# Cyclic Monoterpene–Aromatic Hybrids from Chiral Pool Terpenoid Ketones: Practical Synthetic Methodology for Accessing Them and In Silico Assessment as Cannabinoid Receptor CB1/CB2 Ligands

**DOI:** 10.3390/molecules31152599

**Published:** 2026-07-25

**Authors:** Vasiliki Kaikiti, Andrea Jaksic, Basharat Ali, Savvas N. Georgiades

**Affiliations:** 1 Department of Chemistry, University of Cyprus, 1 Panepistimiou Avenue, Nicosia 2109, Cyprus; kaikiti.vasiliki@ucy.ac.cy (V.K.); a.jaksic@students.uu.nl (A.J.); 2 Institute of Chemistry, Khawaja Fareed University of Engineering and Information Technology, Rahim Yar Khan 64200, Pakistan; basharat.ali@kfueit.edu.pk

**Keywords:** cyclic monoterpenes, aromatic terpenoids, enol triflate, Suzuki–Miyaura C-C cross-coupling, stereoselective hydrogenation, cannabinoid mimics

## Abstract

Natural products featuring a direct σ-bond between a cyclic monoterpene and an aromatic moiety provide a vast source of biological activities, such as antimicrobial, anticancer, antiviral, anticoagulant and cannabinoid regulatory, among others. Only few methods exist for synthetically accessing such hybrid structures and their analogs, all of which are prone to limitations, most notably the reliance on sensitive organometallic intermediates and the difficulty in furnishing certain stereoisomers. An efficient, three-stage synthetic methodology is described herein, that enables the production of hybrid structures featuring a C(sp^3^)-C(sp^2^) bond between six-membered cyclic monoterpenes and aromatic moieties. This process combines: enol triflate formation from a terpenoid ketone precursor, that introduces most of the stereochemical information; Suzuki–Miyaura C-C cross-coupling of the enol triflate with a pool of (hetero)arylboronic acids, to establish the terpene–aromatic link, initially in the form of a C(sp^2^)-C(sp^2^) bond; and a stereoselective hydrogenation of the resulting adducts to afford the target compounds, establishing the stereoconfiguration of the last chiral center. Enantiomeric terpenoid scaffolds derived from menthone and *trans*-tetrahydrocarvone have been combined with six (6) (hetero)arylboronic acids, including medicinally relevant moieties, such as methoxyphenyl, pyridine, quinoline and benzofuran. The power of this method, apart from circumventing the need for in situ-formed sensitive organometallic intermediates, resides in providing access, for the first time, to menthyl- and *trans*-tetrahydrocarvoneyl-type stereoisomers, that were unattainable by any previously described method. The resulting compound library members exhibit drug-like features, based on the computational assessment of 11 selected physicochemical parameters (molecular weight, polarity, aqueous solubility, degree of unsaturation, conformational flexibility, lipophilicity, BBB permeability, skin permeability, gastrointestinal absorption, P-glycoprotein substrate behavior and Lipinski compatibility), using the platforms SwissADME, ADMETLab 3.0 and pkCSM. A computational docking study employing AutoDock Vina further identified promising candidates for targeting the known binding sites of human cannabinoid receptors CB1 and CB2, with calculated binding affinities comparable to those of established ligands.

## 1. Introduction

Terpenes and terpenoids constitute the most extensive and structurally diverse class of natural products, exceeding 55,000 known members. Despite their structural variation, all are biosynthetically derived from a common isoprene (C5) precursor unit, with their sub-classification being determined by the exact number of precursor molecules incorporated in their structure. Terpenes and terpenoids have well-established roles in almost all fundamental plant processes, including growth and development, reproduction, defense and chemical ecology [1]. Lower terpenes and terpenoids find applications beyond their natural roles, including uses as flavors in foods, fragrance agents in perfumes, and raw materials in the pharmaceuticals industry [2].

**Figure 1:**
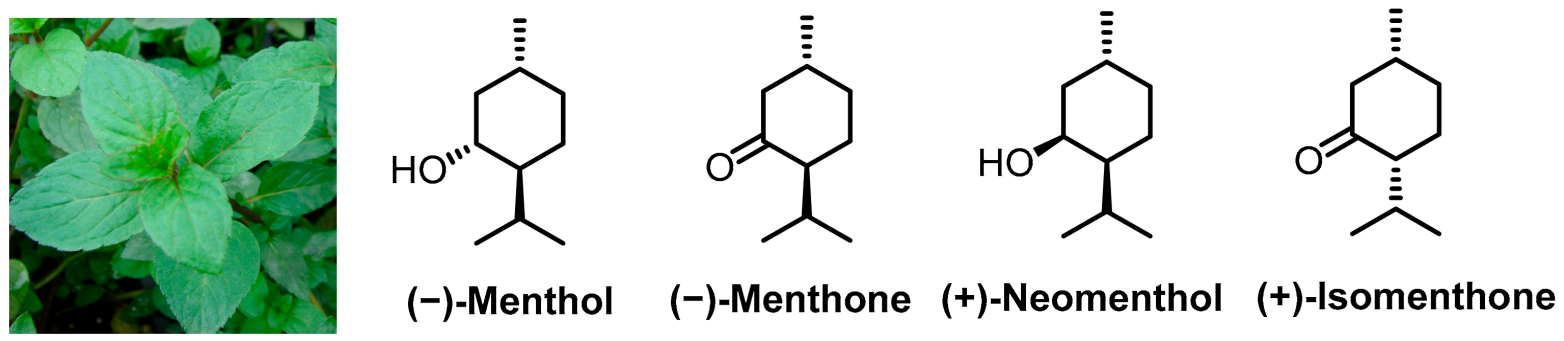
Representative examples of cyclic monoterpenoid components of peppermint essential oil (PEO), exhibiting biological activities: (−)-menthol = (1*R*,2*S*,5*R*)-2-isopropyl-5-methylcyclohexan-1-ol; (−)-menthone = (2*S*,5*R*)-2-isopropyl-5-methylcyclohexan-1-one; (+)-neomenthol = (1*S*,2*S*,5*R*)-2-isopropyl-5-methylcyclohexan-1-ol; (+)-isomenthone = (2*R*,5*R*)-2-isopropyl-5-methylcyclohexan-1-one.

Cyclic monoterpenes (C10 skeleton), in particular, have been associated with significant pharmacological properties. For example, peppermint essential oil (PEO), extracted from *Mentha piperita* (commonly known as peppermint), which contains menthone, menthol, neomenthol and iso-menthone (Figure 1), finds applications in the treatment of flu, headache, fever, sore throat and red eye [3]. Furthermore, menthol possesses radical-scavenging and antioxidant functions, rendering it effective against inflammatory bowel disease (IBD), caused by neutrophil infiltration, free radical formation and increased oxidative stress [4]. Menthol reduces oxidative stress in the colon tissue, while decreasing the levels of both malondialdehyde and the products of lipid peroxidation [4]. Moreover, PEO reduces melanin synthesis in spindle-shaped, epithelial-like melanoma mouse cells by attenuating the expression of microphthalmia-associated transcription factor (MITF), tyrosinase-related proteins TRP−1 and TRP-2, and tyrosinase [4]. Importantly, menthol is a known modulator for various receptors. It acts as an agonist of the transient receptor potential melastatin 8 (TRPM8) channels, which can be activated by cold, membrane depolarization and chemical ligands [5]. The cooling effect of menthol can be attributed to this activation, which leads to an analgesic effect by disrupting nociceptive nerve signaling. Menthol can also block voltage-gated Na^+^ channels and lead to inactivation of the high-voltage-activated Ca^2+^ channel [5]. It activates the transient receptor potential vanilloid 3 (TRPV3) in the skin and neural tissues, contributing to skin sensitization [5]. It can also lead to the activation of the TRPV1 and transient receptor potential ankyrin 1 (TRPA1) channel, effects which overlap with the activation of TRPM8 receptors in the skin [5].

**Figure 2:**
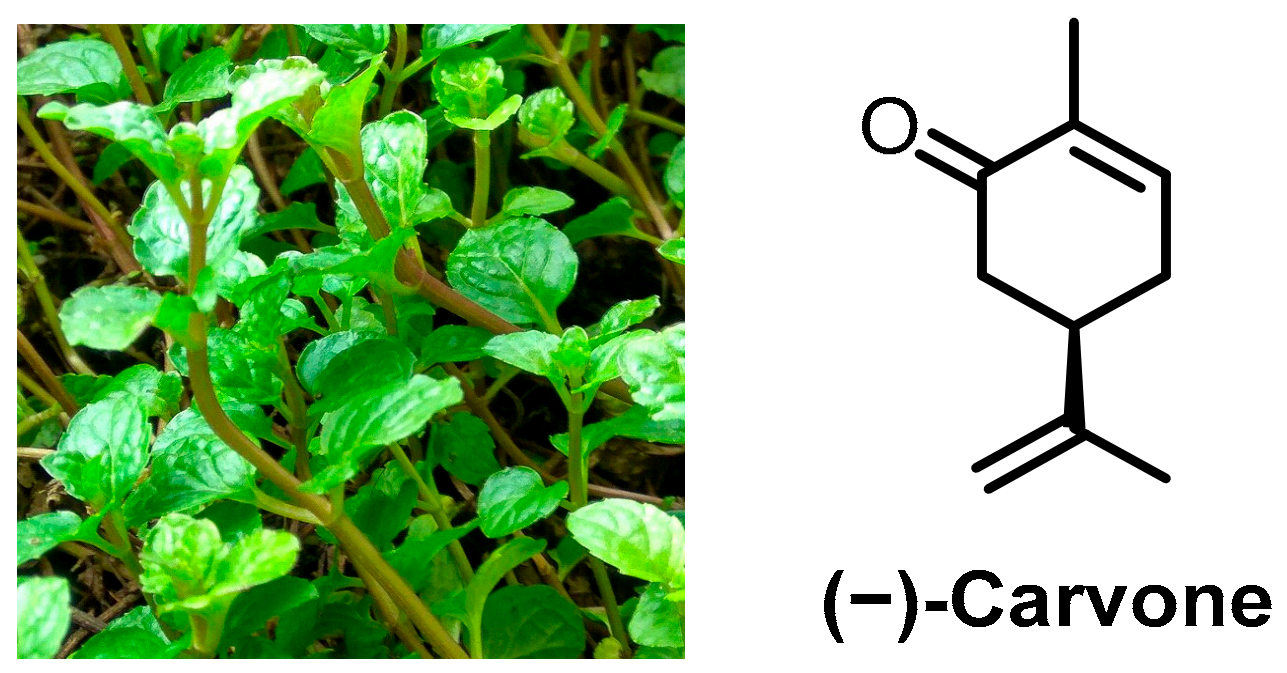
(−)-Carvone, a medicinally active cyclic monoterpenoid that originates from spearmint. IUPAC name: (*R*)-2-methyl-5-(prop-1-en-2-yl)cyclohex-2-en-1-one.

On the other hand, the spearmint-derived (*R*)-(−)-carvone (Figure 2) has shown bactericidal effects on strains of Gram-positive bacteria such as *S. aureus*, *E. faecium* and *M. smegmatis* [6]. Despite exhibiting resistance to certain classes of antibiotics, some Gram-negative bacteria are also sensitive to carvone, owing to its ability to penetrate their outer protective capsule [6]. Carvone further exhibits anti-inflammatory properties by reducing the production levels of pro-inflammatory molecules and increasing the levels of anti-inflammatory cytokines [7,8]. Specifically, in lipopolysaccharide (LPS)-sensitized macrophages, (*R*)-(−)-carvone leads to a reduction in pro-inflammatory cytokines IL-6, IL-1α and IL-1β, and reduces production of tumor necrosis factor-alpha (TNF-α) and nitrogen monoxide (NO), while enhancing the levels of cytokine IL-10, also involved in inflammatory processes [7,8]. It also inhibits the nuclear factor kappa B (NF-κB), a transcription factor with a crucial role in immune response, inflammation and cell growth [7,8]. (*R*)-(−)-carvone has shown a promising antioxidant profile in relevant assays including DPPH, ABTS, ORAC, superoxide and nitric oxide production, as well as iron chelation-based assays [6]. Cardioprotective effects have also been demonstrated for (*R*)-(−)-carvone in cases of doxorubicine-induced toxicity in mice, where treatment with carvone has resulted in a significant increase in antioxidant defense-related catalase activity and a decrease in the levels of creatine kinase and lactate dehydrogenase enzymes [6]. Notably, several in vitro studies have reported the antiproliferative/antitumor potential of carvone in a variety of cancer cell lines, including human leukemia (HL-60) [9], human prostate (LNCaP and PC-3) [9], human melanoma (A375) [10] and human breast (MDA-MB468) [10].

**Figure 3:**
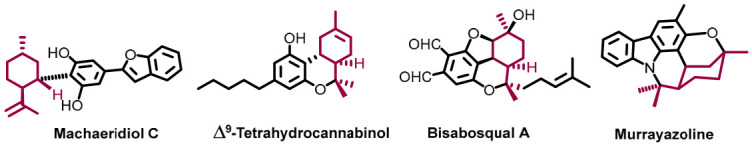
Representative examples of natural products comprising a cyclic monoterpene-aryl C(sp^3^)-C(sp^2^) connection. The terpene fragment is highlighted in red.

In addition to engaging in multivalent hydrophobic contacts with biological targets—the main component of their interaction—cyclic terpenes afford unique 3D skeletal scaffolds for the organization and display of peripheral functional groups, enabling them to enhance binding affinity, as well as selectivity. Substituted aryl and heteroaryl moieties constitute ideal building blocks for expanding the organic framework of terpenes, due to their limited space occupancy, a result of planarity, and their ability to contribute to hydrogen bonding, stemming from the presence of heteroatoms. Nature provides numerous examples of terpene–aromatic hybrid structures, in which the aromatic is frequently of phenolic origin. These hybrids are biologically significant, exhibiting activities as diverse as cannabinoid receptor modulating (e.g., machaeridiol C, Δ^9^-THC) [11,12], broad-spectrum antifungal (e.g., bisabosqual A) [13] and mTOR/AKT regulatory (e.g., murrayazoline) [14] (Figure 3).

**Scheme 1:**
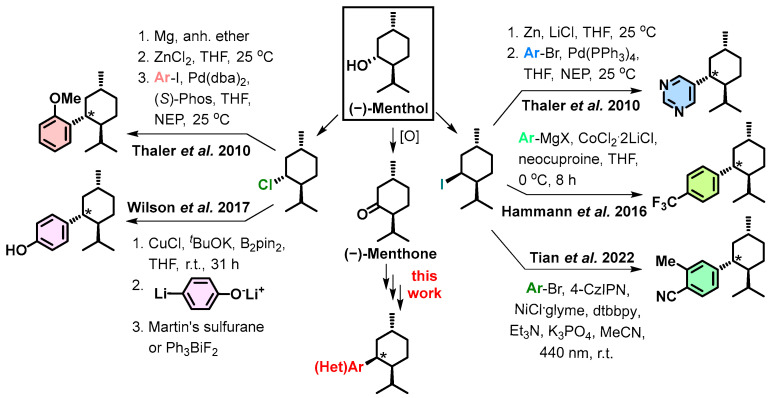
Literature methods for accessing terpene–aromatic hybrid structures from terpenoid alcohols or halides [15,16,17,18]. Existing methodologies rely on sensitive organometallic intermediates in stoichiometric fashion or complex photoredox catalysis conditions, which deliver equatorially connected aryl derivatives from menthyl precursors. The current work provides stereoselective access to derivatives with the opposite configuration on the newly generated stereocenter (marked with an asterisk) in the case of menthyl substrates, which were previously unattainable.

Synthetically, the formation of a direct C(sp^3^)-C(sp^2^) connection between a cyclic terpene scaffold and an (hetero)aromatic ring, respectively, to obtain hybrid derivatives, has been previously explored by other research teams, leading to methodologies that employ boranes, alkali (lithium), alkaline earth (magnesium) or first-row transition metals (zinc) in stoichiometric fashion, applied on terpenoid alcohol-derived halides (Scheme 1) [15,16,17]. Such reagents are moisture-sensitive and often require transmetallation steps, in combination with palladium, copper or cobalt catalysts. Dictated by conformational stereoelectronic limitations on a (metal-based) intermediate, these transformations have consistently afforded an equatorially connected newly installed aryl moiety on the menthol scaffold, while axially connected counterparts have remained largely unattainable by the same methods, until now. A nickel-mediated photoredox method, featuring a radical intermediate, also leads to the same stereoisomer type for the menthyl system, albeit requiring a complex reaction mix [18].

The current study aims to develop an alternative methodology that eliminates the need for sensitive stoichiometric organometallic reagents or complex reaction conditions, while exploiting the cyclic monoterpene’s pre-existing stereocenters, to direct the stereoconfiguration in the final (hetero)aryl–terpene hybrid product, in order to obtain, with high stereoselectivity, unprecedented diastereoisomers in the case of menthyl- and *trans*-tetrahydrocarvoneyl-type scaffolds. The current study has overall yielded 24 intermediates and 24 final derivatives starting from both enantiomers of menthone and *trans*-tetrahydrocarvone, which compose a new compound library that will serve as a pool of potential bioactives, for pharmacological evaluation. We demonstrate computationally, by examining eleven (11) key parameters, that the synthesized derivatives possess drug-like physicochemical features, while their notable structural similarity to known cannabinoid ligands, a promising class of bioactives, has prompted us to evaluate, via a molecular docking study, their potential to serve as ligands of human cannabinoid receptors CB1 and CB2.

## 2. Results and Discussion

### 2.1. Synthesis of Cyclic Monoterpene–Aromatic Hybrid Derivatives

The need to overcome some of the limitations associated with previous synthetic protocols, namely the stoichiometric use of humidity-sensitive organometallic species (organo-lithium, -magnesium, -zinc), the incompatibility of reaction protocols with certain heteroaromatic building blocks, and the inability to access specific diastereomers from the initial terpenoids for biological evaluation led to the development of an alternative, robust and substrate nature-independent methodology towards cyclic terpene–aromatic hybrid derivatives. Specifically, a short and practical synthetic route was explored for the construction of derivatives comprising a direct C(sp^3^)-C(sp^2^) σ-bond between a six-membered cyclic monoterpene scaffold and a (hetero)aromatic moiety.

**Scheme 2:**
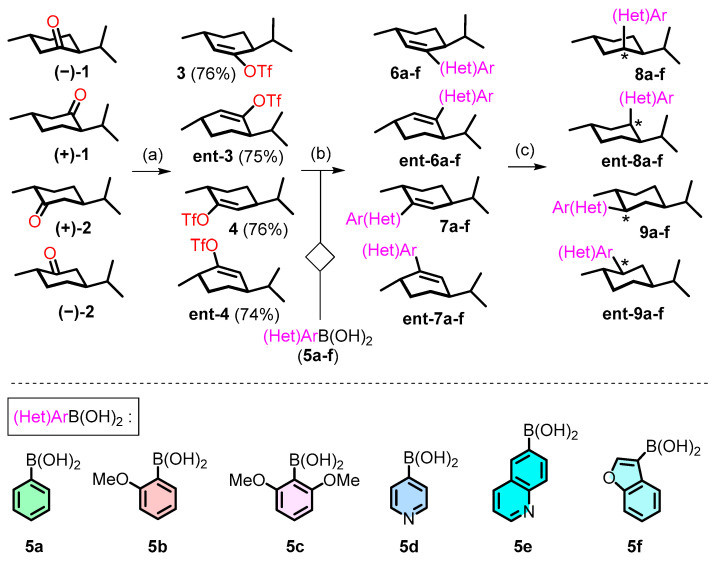
Three-stage synthetic route for the generation of cyclic monoterpene–aromatic hybrid derivatives from chiral pool terpenoid ketones **1** and **2** (both enantiomers of the terpenoid substrates were included in the study). Key: (**a**) THF, PhNTf_2_, LiHMDS, −85 °C to 0 °C, 0.5–1.5 h; (**b**) PhMe-EtOH (2:1), Pd(PPh_3_)_4_, K_2_CO_3_, 85 °C, 24 h; (**c**) EtOH, H_2_, Pd/C, r.t., 24 h. An asterisk marks the stereocenter established in the hydrogenation step. Last row: (Hetero)aryl boronic acid building blocks employed in step (**b**), a set of parallel reactions, in which all terpenoid trifluoromethanesulfonate substrates were combined with six boronic acids to afford all possible combinations. [ent = enantiomer].

The synthetic methodology described herein initiates from chiral pool cyclic monoterpene ketone precursors with a saturated carbon framework, and comprises three stages: (a) conversion of the terpenoid ketone to an enol trifluoromethanesulfonate derivative; (b) palladium-catalyzed C-C cross-coupling to establish a C(sp^2^)-C(sp^2^) connection between the terpene framework and a (hetero)aromatic moiety, with the latter introduced as a boronic acid building block; and (c) heterogeneous catalytic hydrogenation, whose stereochemical outcome is controlled by the pre-existing stereocenters, to afford one major hybrid product featuring C(sp^3^)-C(sp^2^) connectivity (Scheme 2).

Two six-membered cyclic monoterpene scaffolds, each in the form of individual enantiomers, were employed in the current study: (−)- and (+)-menthone (**1**) were separately obtained, either commercially or by applying a literature PCC oxidation protocol on the corresponding menthol enantiomer [19]; and (+)- and (−)-*trans*-tetrahydrocarvone (**2**) were separately obtained from the corresponding pure enantiomers of carvone, an abundant ketone with a higher degree of unsaturation, via reduction of the unsaturated carbon framework that leaves the carbonyl moiety intact, as previously described [20]. These were employed as the precursors for the synthesis of monoterpene-aryl derivatives.

Ketones (−)/(+)-**1** and (+)/(−)-**2** underwent α-deprotonation, at the less hindered secondary position, with lithium hexamethyldisilazane (LiHMDS) in THF, under kinetic control (−85 °C), and the resulting enolate was trapped by addition of excess bis(trifluoromethanesulfonyl)aniline reagent at 0 °C [21], thus affording good yields of the corresponding enol trifluoromethanesulfonyl esters, **3** (76%)/**ent-3** (75%) and **4** (76%)/**ent-4** (74%), respectively (Scheme 2).

**Figure 4:**
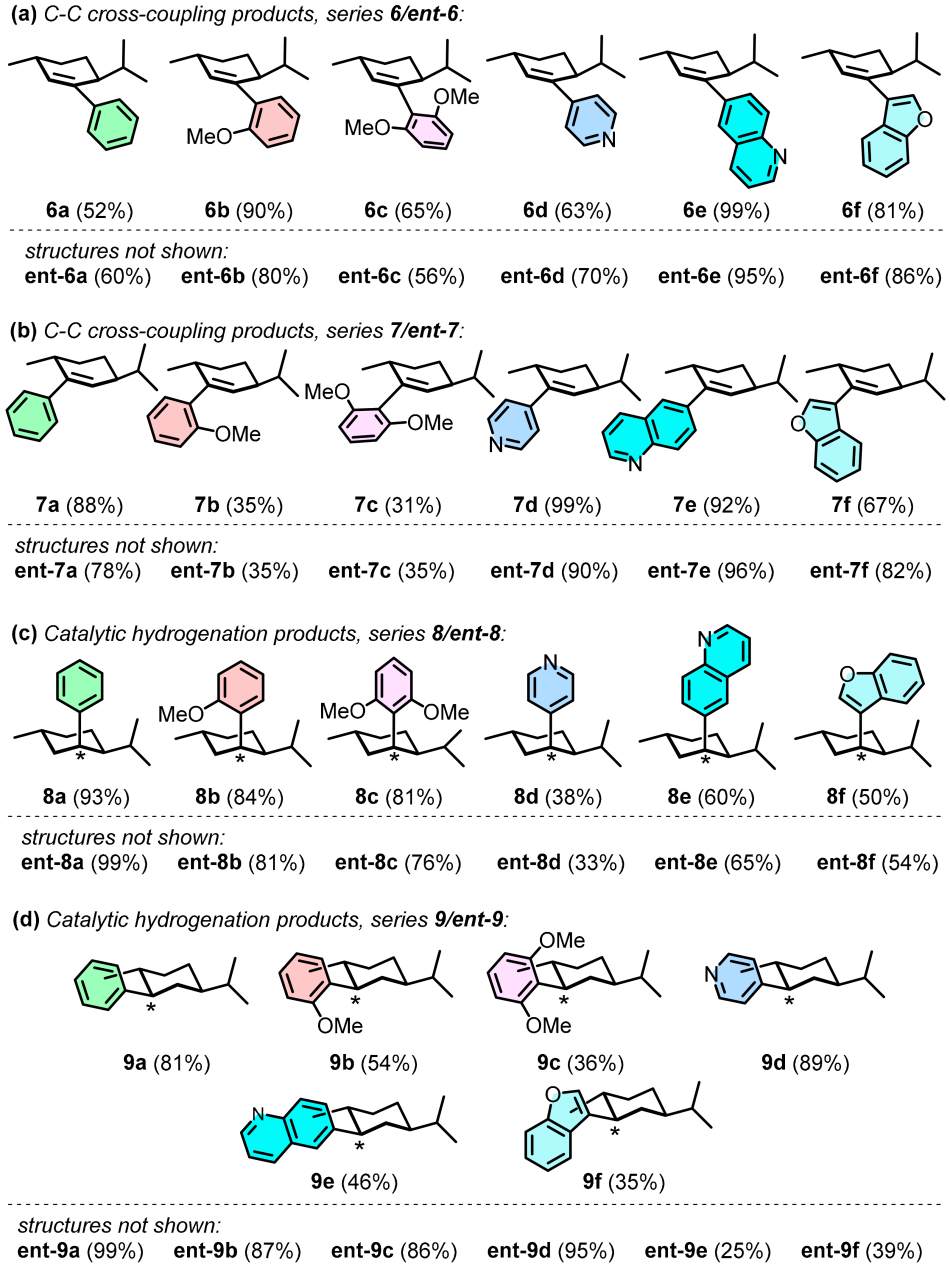
(**a**,**b**) Structures of C-C cross-coupled products from series **6**/**ent-6** and series **7**/**ent-7**, respectively. (**c**,**d**) Structures of catalytic hydrogenation products from series **8**/**ent-8** and series **9**/**ent-9**, respectively. An asterisk marks the stereocenter established in the hydrogenation step.

Intermediates **3**/**ent-3** and **4**/**ent-4** served as substrates for a palladium-catalyzed C-C cross-coupling under Suzuki–Miyaura conditions, using Pd(PPh_3_)_4_ as the catalyst and K_2_CO_3_ as the base, in a mixed toluene–ethanol (2:1) solvent [22]. In this stage, the four substrates were combined with six (6) aryl and heteroaryl boronic acid building blocks: simple phenyl (**5a**), 2-methoxyphenyl (**5b**), 2,6-dimethoxyphenyl (**5c**), 4-pyridyl (**5d**), 6-quinolinyl (**5e**) and 3-benzofuryl (**5f**) (Scheme 2). Oxygen *ortho*-substituted aromatics on cyclic monoterpenes are reminiscent of plant-derived cannabinoids [23], while the selected heteroaromatic moieties (pyridine [24], quinoline [25], benzofuran [26]) are considered privileged motifs in medicinal chemistry, due to their ability to enhance interactions with biological targets and increase water solubility. Cross-coupled products **6a-f**/**ent-6a-f** from the menthone series and **7a-f**/**ent-7a-f** from the *trans*-tetrahydrocarvone series were isolated in satisfactory yields (Figure 4a,b).

Finally, double bond reduction at the arylated site was established via heterogeneous catalytic hydrogenation with H_2_/Pd-C in EtOH [21], at room temperature, with the pre-existing stereocenters dictating stereoselectivity. Literature precedents suggest that the choice of solvent is important for tuning stereoselectivity [27,28,29,30] and, in this case, ethanol proved to be optimal in yielding one major diastereomer. Alkenes of series **6a–f** and series **7a–f** afforded products of series **8a–f** and **9a–f**, respectively. Only the dominant diastereomer, as determined by meticulous TLC analysis, was isolated in each case in pure form, while a minor, trace diastereomer (<5%) was detected, but always obtained as an inseparable mixture with an amount of the major diastereomeric product. The extensive overlap of signals between the two did not allow the ^1^H NMR characterization of the minor product or the determination of the diastereomeric ratio in the hydrogenations. Notably, in certain cases associated with poor yields, evidence of degradation products was observed. The latter exhibited an R_f_ value equal to zero on TLC eluted with the solvent systems employed for chromatography (see Synthetic Methods, Section 3.2.3), suggesting a partial reduction of sensitive heteroaromatic rings to more polar derivatives, likely due to the long reaction times. Attempts to reduce reaction times were accompanied by a reduction in yield of the major product. Hydrogenation yields varied from moderate to high, depending on the combination of terpene scaffold and (hetero)aromatic moiety (Figure 4c,d).

### 2.2. Compound Characterization and Stereochemical Analysis

Each enol trifluoromethanesulfonate intermediate, C-C cross-coupled intermediate and final hydrogenation product was isolated by flash column chromatography in high purity (>95%) and fully characterized by ^1^H NMR, ^13^C NMR, 2D (COSY, HSQC) NMR, FT-IR, and polarimetry (see Supplementary Materials). ^1^H and ^13^C NMR provided unambiguous evidence for the success of both the C-C cross-coupling stage, that attaches the aromatic moieties to the terpene scaffolds, and the catalytic hydrogenation stage, that affords saturated terpene frameworks in the final derivatives. All spectra were in good agreement with the respective proposed structures. Key proton and carbon assignments were performed by ^1^H-^1^H relay (COSY) and ^1^H-^13^C relay (HSQC). For the final hydrogenation products, 2D NOESY NMR spectra were also obtained. Polarimetry confirmed that all enantiomers had comparable optical rotation absolute values but opposite signs.

**Figure 5:**
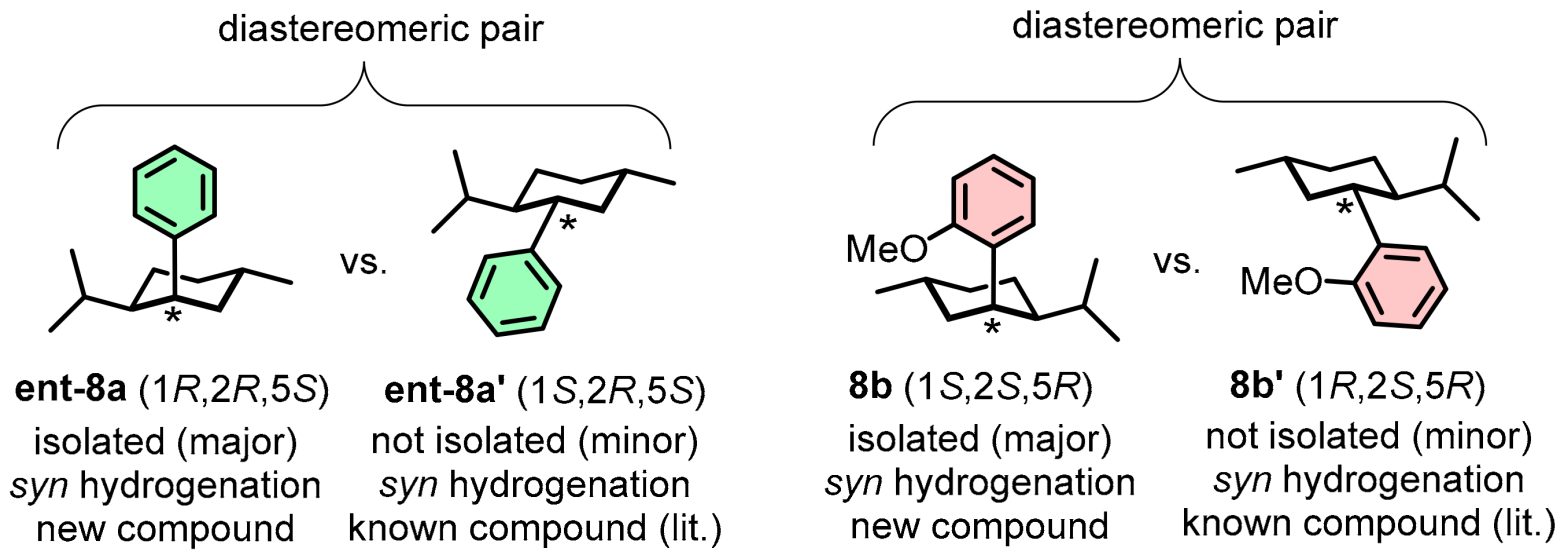
Comparison of isolated diastereomers (left compound in each diastereomeric pair) with non-isolated (right compound in each diastereomeric pair). The isolated ones are new compounds, while the non-isolated ones are known literature compounds. Their ^1^H NMR spectra differ significantly in the chemical shift (*δ*) of the H-atom connected to the carbon center marked with an asterisk. The asterisk shows the stereocenter established in the hydrogenation step.

The stereochemical assignment of the isolated, major products (single diastereomers) of the hydrogenation was based on a combination of comparison with literature compounds, NMR spectra computational prediction and 2D NOESY NMR analysis. While all hydrogenation products of series **8**/**ent-8** isolated in the current study are new compounds, two of their diastereomers were previously prepared by independent methods and reported in the literature, namely the (1*S*,2*R*,5*S*) diastereomer of compound **ent-8a** (referred to herein as **ent-8a’**) [31] and the (1*R*,2*S*,5*R*) diastereomer of compound **8b** (referred to herein as **8b’**) [16,32] (Figure 5). We propose that these two known compounds are, in fact, the minor *syn* hydrogenation diastereomers that occurred in our reaction mixtures in traces, alongside **ent-8a** and **8b**, respectively, but could not be isolated. However, the availability of their literature NMR data enabled the comparison with our own original data, recorded for the isolated, major diastereomers **ent-8a** and **8b**, and led to the conclusion that they were different sets of compounds. Specifically, in each pair under comparison (Figure 5), apart from differences in the aromatic region, the spectra of the isolated compounds, **ent-8a** and **8b**, exhibited a characteristic broadened peak in the region 3.2–3.8 ppm, which is absent from the literature spectra of respective diastereomers **ent-8a’** and **8b’**. This can be attributed to the H-atom connected to the newly established chiral center. In both literature diastereomers, this signal is shifted below 2.8 ppm. This signal is a trend in the entire series **8**/**ent-8**, suggesting that the isolated diastereomers obtained by using our methodology are consistent with the proposed structures, and different from those accessible by alternative methods [15,16,17,18].

To obtain further support for this finding, we have conducted a computational prediction of ^1^H and ^13^C NMR spectra using SPARTAN’24 Quantum Mechanics Driver 1.3.0 (Windows 64-bit) (see Supplementary Materials). We employed a single-point job type, the RWB97X-D method and the 6-31G(D) basis set, with 288 basis functions and 120 electrons. A SCF model was applied to perform a restricted hybrid HF-DFT SCF calculation, using Pulay DIIS + Geometric Direct Minimization. The obtained computational spectra for compound **8a**, which was studied as a model for its series, compared well with the experimentally obtained spectra, and notably correctly predicted the H-atom signal both in terms of chemical shift (*δ*), around 3.26 ppm, as well as the small value of the *J* coupling constant, corresponding to the 1,2-*cis* (equatorial-axial) and/or 1,2-*trans* (equatorial-equatorial) relationship with its neighbor protons.

Compounds of series **9**/**ent-9** and their diastereomers are entirely unprecedented in the literature; therefore, we had to turn to the computational model for stereochemistry assignment of the new stereocenter. In the case of compound **9a**, used as a model for its series, the simulation failed to accurately predict the chemical shift (*δ*) of the distinctive H-atom in the region 2.8–3.4 ppm that is observed in the experimentally obtained ^1^H NMR spectra across all compounds of this series. However, it reliably predicted a high value of the *J* coupling constant for the same proton, in the order of 12–13 Hz, very characteristic of 1,2-*trans* (axial-axial) neighbor proton coupling, as would be expected for the proposed diastereomers based on the Karplus equation [33]. Indeed, in the experimentally obtained ^1^H NMR spectra of all compounds of series **9**/**ent-9**, the signal of the axial H-atom connected to the new chiral center exhibits an extremely high *J* value, in contrast to compounds of series **8**/**ent-8**, where the respective H-atom is equatorial.

**Figure 6:**
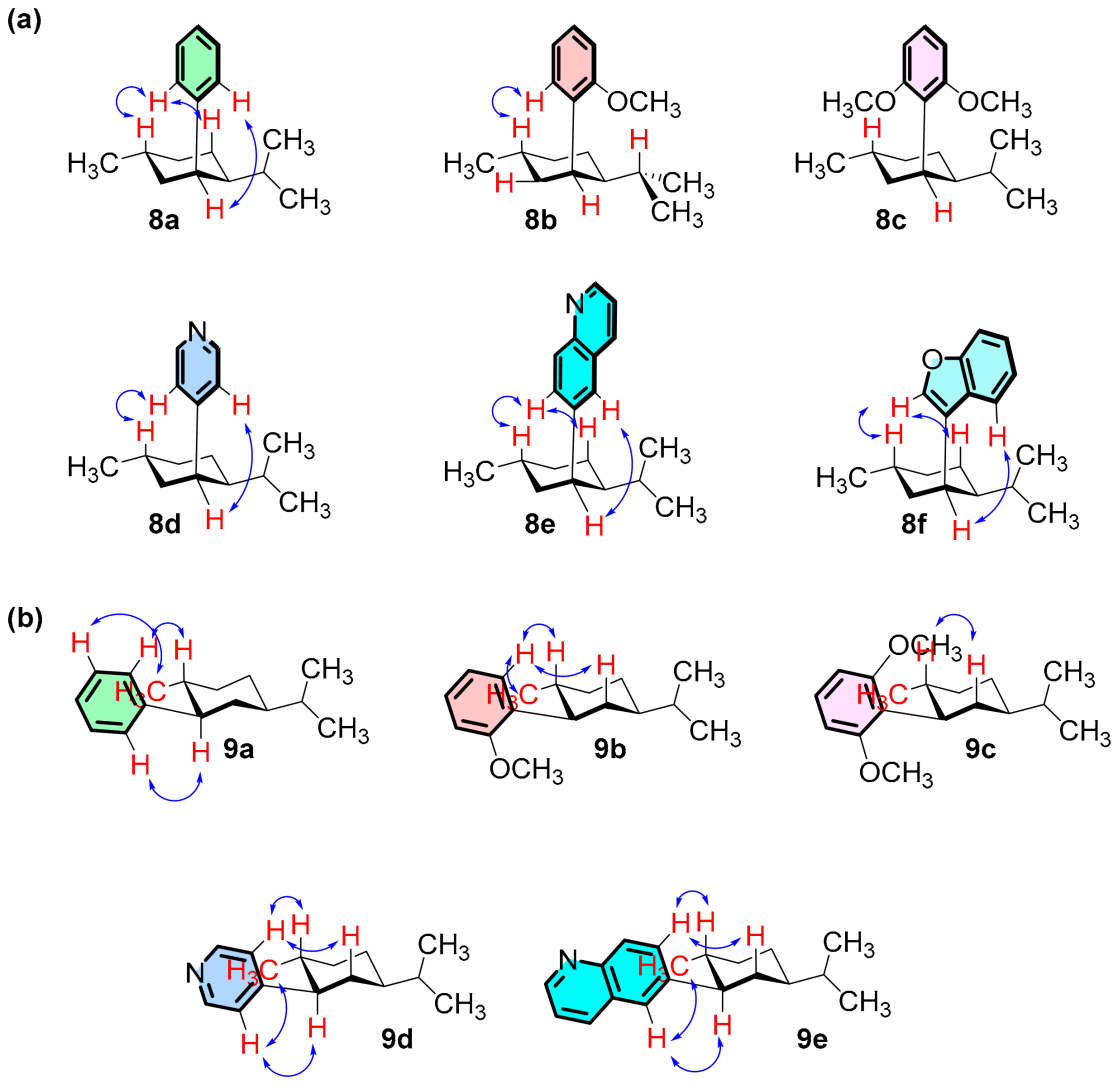
^1^H-^1^H NOESY NMR relays between the newly incorporated (hetero)aryl moieties and key terpene scaffold aliphatic protons, that supported the stereochemical assignment of the new stereocenter in the products of series **8** (**a**) and **9** (**b**). Identical relays were observed for their enantiomers, of series **ent-8** and **ent-9**.

In addition to the above, 2D NOESY NMR spectra were systematically analyzed to identify characteristic through-space ^1^H-^1^H relays, consistent with the above-proposed structures. Such relays were identified for the majority of compounds from series **8**/**ent-8** and **9**/**ent-9**, and are highlighted in Figure 6. They suggest that, in the case of products from series **8**/**ent-8** (menthyl scaffold), the incorporated (hetero)aryl substituent is *trans* to the methyl and *cis* to the isopropyl substituent. This is an important finding, suggesting a unique path to previously unattainable diastereomers of a menthyl scaffold, since all existing protocols are known to furnish the opposite stereoconfiguration at this new chiral center [15,16,17,18]. In the case of products from series **9**/**ent-9** (*trans*-tetrahydrocarvone scaffold), the new (hetero)aryl substituent is also found to be *trans* to the methyl and *cis* to the isopropyl group. It is noted that products of this connectivity are unprecedented, and no alternative methods currently exist for accessing their diastereomers either.

**Scheme 3:**
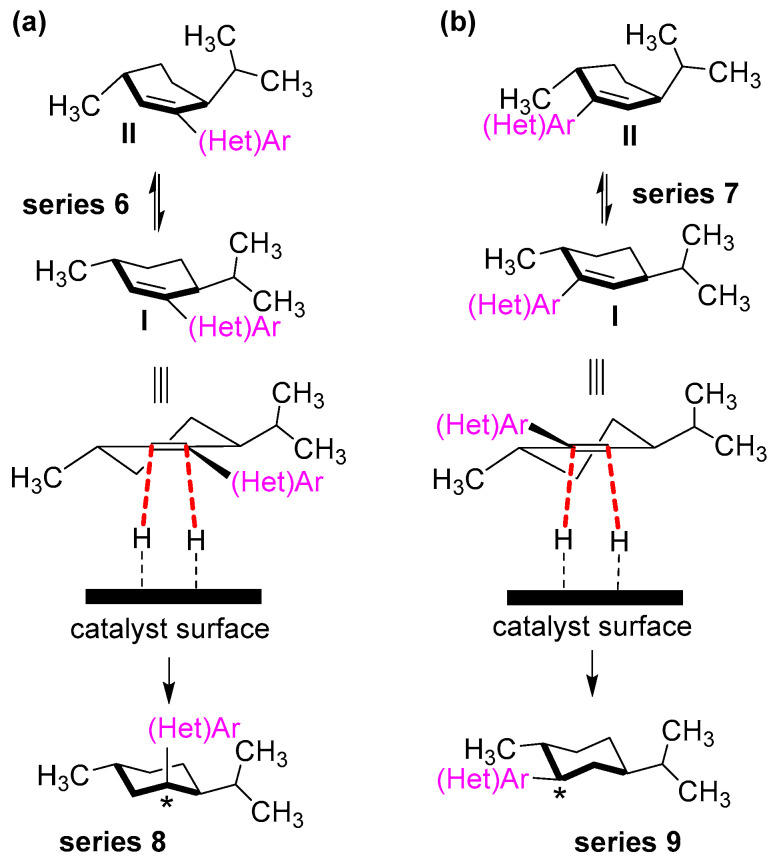
Heterogeneous catalytic hydrogenation at the most accessible face of the six-membered terpene ring, leading to control of stereoconfiguration of final terpene–aromatic hybrid products, for the two scaffolds employed in this study. Substrate conformation is a contribution of two forms, I and II. (**a**) Hydrogenation of menthyl derivatives (series **6**), affording unprecedented diastereomers (series **8**). (**b**) Hydrogenation of *trans*-tetrahydrocarvoneyl derivatives (series **7**), affording unprecedented products of series **9**. The asterisk marks the stereocenter established in the hydrogenation step.

The observed stereoselectivity in the catalytic hydrogenation of substrate types **6**/**ent-6** and **7**/**ent-7** can be rationalized based on a *syn* hydrogenation mode, as is well-established under similar heterogeneous conditions, with the substrate’s conformation being dictated by the two pre-existing substituents. This comprises unequal contributions from a bis(pseudoequatorial) (I) and a bis(pseudoaxial) (II) form (Scheme 3), overall rendering the face bearing the isopropyl group significantly more sterically hindered than the one bearing the methyl group. Consequently, we propose that the hydrogenation proceeds from the more accessible face, resulting in the aromatic substituent being positioned *trans* relative to the methyl group and *cis* relative to the isopropyl group, in the final products of both series **8**/**ent-8** and **9**/**ent-9**.

### 2.3. Computational ADMET Assessment to Identify Drug-like Compounds

The molecular library generated by the described synthetic effort can be considered biased towards bioactive compound discovery, owing to combining recognizable six-membered cyclic monoterpene scaffolds, evolved by nature to interact with a plethora of biomolecular binding sites, and small (hetero)aromatic ring systems, typically employed in medicinal chemistry as privileged motifs, because of their size, planarity and hydrogen-bonding ability. These unique combinations, further leveraged by the exploitation of stereochemical diversity in the current study, are expected to enhance the ability of this compound library to populate pharmacologically relevant areas of 3D chemical space and, consequently, its potential for modulation of diverse biomolecular targets.

As the members of this molecular library are intended for evaluation in the context of future drug discovery efforts, it was necessary to assess their drug-like behavior computationally, as a first level of candidate validation. This computational study included: four (4) terpenoid ketones; four (4) enol trifluoromethanesulfonates; twenty-four (24) C-C cross-coupling intermediates; and twenty-four (24) final hydrogenation products (terpenoid–aromatic hybrids with saturated carbon framework).

Two widely employed online tools, SwissADME (Swiss Institute of Bioinformatics, http://www.sib.swiss, accessed on 20 July 2026) [34] and ADMETLab 3.0 (Xiangya School of Pharmaceutical Sciences, Central South University, https://admetlab3.scbdd.com/, accessed on 20 July 2026) [35], were used to computationally determine the values of six (6) pharmacologically important physicochemical parameters (molecular weight, polarity—expressed as topological polar surface area, aqueous solubility, degree of unsaturation, conformational flexibility and lipophilicity) for each compound included in the study, by means of comparison against the corresponding values for all known drugs, with the latter defining the threshold or range for what is considered drug-like. The thresholds of the examined physicochemical parameters were: molecular weight 150 to 500 g.mol^−1^; polarity (referring to TPSA) 20 to 130 Å^2^; aqueous solubility score −6 to 0; degree of unsaturation score 0.25 to 1; conformational flexibility score 0 to 9; and lipophilicity score −0.7 to 5.0. It is noted that the selected parameters encode some of the most indispensable structural information of the compounds for enabling the assessment of their pharmacokinetic behavior in a medicinal context.

**Figure 7:**
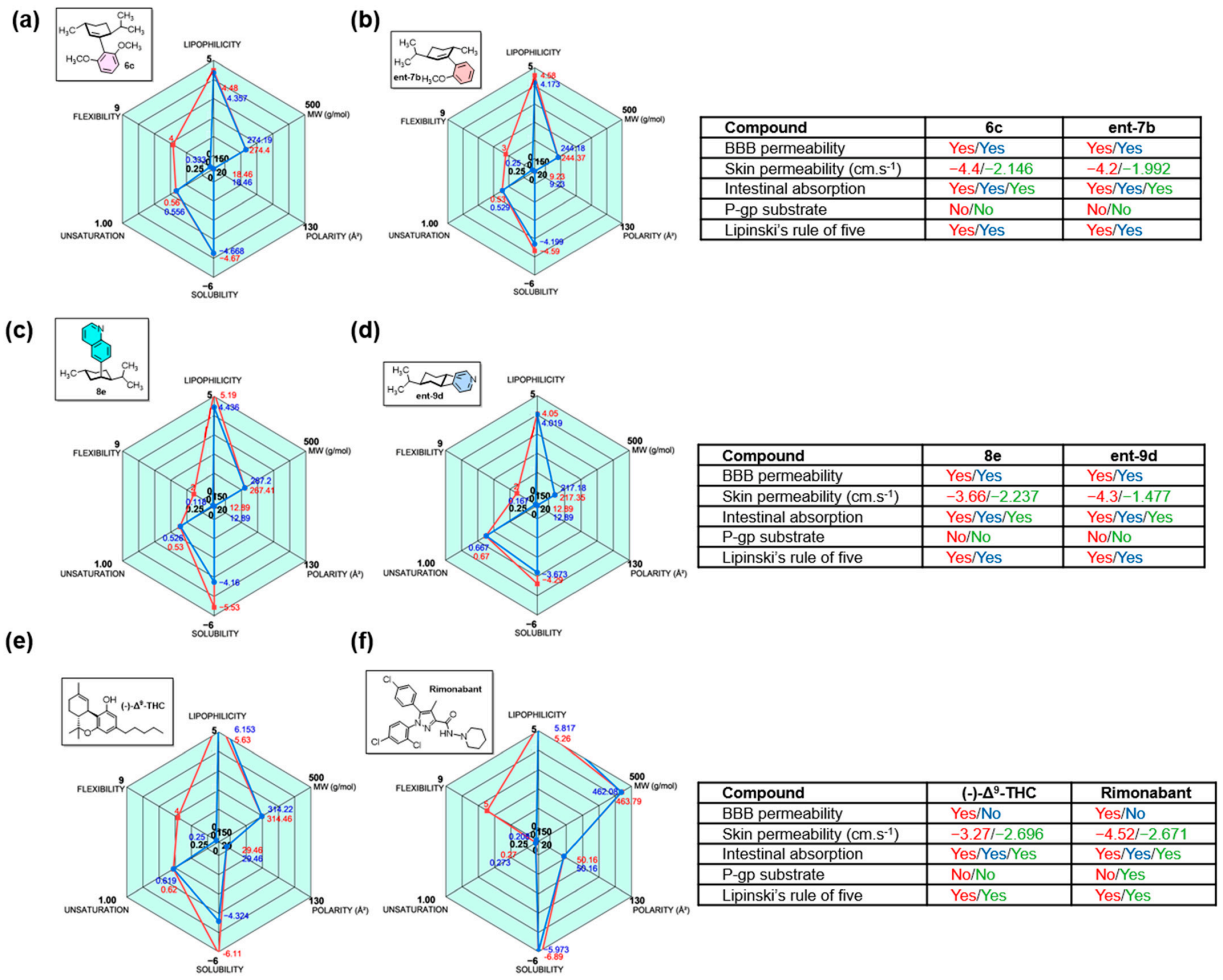
Predicted ADMET profiles of representative examples of hybrid compounds from (**a**) series **6**, (**b**) series **ent-7**, (**c**) series **8** and (**d**) series **ent-9**, in comparison with two well-established cannabinoid receptor ligands, (**e**) Δ^9^-tetrahydrocannabinol (THC) and (**f**) Rimonabant. For calculations we employed three (3) independent online platforms: SwissADME (data in red), ADMETLab 3.0 (data in blue) and pkCSM (data in green). The same color-coding applies to both the radar charts and the tables. The radar charts present six (6) key physicochemical parameters, with the cyan normal hexagon corners corresponding to the upper thresholds, the center of axes corresponding to the lower threshold, and the hexagon outer lines representing the critical lines, within which compounds found are considered drug-like. Lower and upper thresholds are defined based on the structures of all known drugs. Threshold values are: molecular weight 150 to 500 g.mol^−1^; polarity (TPSA) 20 to 130 Å^2^; aqueous solubility score −6 to 0; degree of unsaturation score 0.25 to 1; conformational flexibility score 0 to 9; and lipophilicity −0.7 to 5.0. The corners of the red and blue polygons correspond to the calculated values for the compound under study, obtained by using two different online platforms. Five additional parameters (qualitative or quantitative), obtained from two or three online platforms, are included in the tables.

Compounds that fell within the above numerical limits for all (or most of) the parameters under study were considered suitable for further progression in drug discovery studies. Gratifyingly, for most members of the molecular library, all six (6) calculated parameters were found to fall well within the acceptable thresholds, as predicted by both computational tools, thus satisfying the requirement for drug-likeness. Figure 7 illustrates these values as polygons on a radar chart for two representative examples of compounds from each of the Suzuki–Miyaura (a,b) and catalytic hydrogenation (c,d) synthetic stages. The full set of data can be found in the Supplementary Materials. The cyan background defines the drug-like thresholds, while the angles of the polygons define the calculated values for each compound.

Comparison of the values predicted by SwissADME (red polygon) and ADMETLab 3.0 (blue polygon) generally reveals good alignment for the majority of the parameters studied. Unsurprisingly, the calculated values are identical for MW and unsaturation, which are both directly related to compound structure. Only minor deviations in the predictions of the two platforms are observed in aqueous solubility, polarity and lipophilicity, suggesting similar algorithms for predicting these properties. However, more pronounced deviations are observed in flexibility for some library members, especially those comprising a 2,6-dimethoxyphenyl aromatic and, to a lesser extent those comprising a 2-methoxyphenyl aromatic, implying a susceptibility of this parameter to different mathematical models employed by the two tools. For 2,6-dimethoxy-substituted compounds (**6c**/**ent-6c**, **7c**/**ent-7c**, **8c**/**ent-8c**, **9c**/**ent-9c**), the values of flexibility, as predicted by SwissADME, are the highest in their series, about 1 flexibility unit higher than the corresponding mono-methoxy counterparts and about 2 units higher than the rest of the compounds.

This finding appears to be consistent with our conformational study of the structures in Chem3D 23.0.1, as described in Section 2.4 of this article, which suggests that the presence of a 2,6-dimethoxyphenyl-substituent may be an important conformation-controlling element, both with regard to the terpene ring conformation as well as the methoxy chain conformation, due to engaging in VDW steric repulsions with adjacent bulky terpene substituents, such as the isopropyl group, or 1,3-diaxial interactions. This, in combination with an increased number of rotatable bonds owing to the presence of two methoxy moieties, leads to an increase in overall conformational flexibility, essentially allowing these compounds to adopt alternative stable conformations, rather than just one. Further evidence comes from the ^1^H NMR of the 2,6-dimethoxy derivatives, where the observed broadening or enhanced multiplicity of peaks in the aromatic region may be suggestive of two conformational populations involving this aromatic. This effect is only picked up by SwissADME, indicating a more advanced algorithm that takes into account the above-mentioned interactions.

To obtain an indication of how our synthetic compounds compare with established ligands or known drugs for cannabinoid receptors CB1 and CB2, whose in silico interaction with the compounds will be discussed in Section 2.4, we have also conducted the same ADMET analysis on Δ^9^-tetrahydrocannabinol (THC), a naturally occurring partial agonist of receptors CB1/CB2 and Rimonabant, a synthetic CB1 antagonist (Figure 7e and Figure 7f, respectively). Gratifyingly, many members of this molecular library achieve very comparable solubility and lipophilicity scores to the profiles of both known ligands (positive controls), and in some cases even fit better within the thresholds, as predicted by both computational tools. They differ considerably in molecular weight and polarity, exhibiting both lower MW and TPSA, which, however, cannot be considered prohibitive, as they may be better suited for use as agonists that mostly exploit hydrophobic rather than polar contacts with the intended targets. The docking study described in Section 2.4 indeed highlights the importance of multiple hydrophobic contacts in achieving an overall strong binding effect for selected library members. The unsaturation score of all library members more closely resembles that of THC rather than Rimonabant, likely due to the similar terpene–aromatic hybrid character they share with the first. Finally, the flexibility score of the known ligands is better approached by the 2,6-dimethoxy-substituted examples, suggesting that this substitution pattern should be further explored in combination with larger aromatic moieties in a next-generation synthetic effort.

Intrigued by these findings, we sought to further expand our set of parameters, by including complementary ones that provide information on absorption, distribution, metabolism or excretion, ultimately contributing in drug behavior, safety and suitability, when a compound is used in the body. SwissADME and ADMETLab 3.0 provided qualitative results for compatibility with Lipinski’s rule of five (5) and blood–brain barrier (BBB) permeability, while a third computational tool, pkCSM (University of Queensland, https://biosig.lab.uq.edu.au/pkcsm/, accessed on 20 July 2026) [36], was additionally explored for providing (qualitative or quantitative) predictions of skin permeability, gastrointestinal (GI) absorption and P-glycoprotein (P-gp) substrate behavior (these last three parameters were also calculated by one or both of the previous computational tools, for comparison). These five (5) additional parameters are provided as inset tables in Figure 7 for the example compounds, while the full set of data can be found in the Supplementary Materials.

Comparison of the predictions from the alternative online platforms shows that all library members are Lipinski-compliant, have satisfactory gastrointestinal absorption and exhibit comparable or better skin permeability values compared to the two known cannabinoid ligands included in the study. BBB permeability predictions, conducted by SwissADME and ADMETLab 3.0, suggest that the starting chiral pool ketones as well as all library members featuring a 2,6-dimethoxyphenyl, 4-pyridyl or 6-quinolinyl aromatic on both terpene scaffolds examined, plus a 2-methoxyphenyl aromatic on the *trans*-tetrahydrocarvoneyl scaffold, are likely to permeate the BBB. Some cases identified by one of the two platforms to be problematic with regard to this parameter are the intermediate enol trifluoromethanesulfonates **3**/**ent-3**, **4**/**ent-4**, the Suzuki–Miyaura cross-coupling products **6a**/**ent-6a**, **6b**/**ent-6b**, **6f**/**ent-6f**, **7a**/**ent-7a**, **7f**/**ent-7f**, and the final hydrogenation products **8a**/**ent-8a**, **8b**/**ent-8b**, **8f**/**ent-8f**, **9a**/**ent-9a** and **9f**/**ent-9f**. However, these results should be treated with caution, as a unique combination of structural factors may often be responsible for dictating BBB permeability. Finally, both SwissADME and pkCSM predict that none of the compounds are a likely substrate for P-glycoprotein, a protein transporter responsible for compound efflux from cells. This may offer an advantage to this compound family, by increasing compound absorption, enabling the build-up of high intracellular concentration and enhancing activity against certain drug-resistant cell types, as well as increasing time in the brain, provided that the compounds show no toxicity.

### 2.4. Computational Docking Study to Assess Compound Interaction with Cannabinoid Receptors CB1 and CB2

A computational comparison of the synthesized structures (Suzuki–Miyaura and hydrogenation derivative sub-families) from menthone (series **6**/**ent-6** and **8**/**ent-8**, respectively) and *trans*-tetrahydrocarvone (series **7**/**ent-7** and **9**/**ent-9**), against the structures of known ligands for protein biomolecular targets, using the SwissTargetPrediction online tool from the Swiss Institute of Bioinformatics (http://www.sib.swiss, accessed on 20 July 2026) [34], was conducted. This algorithm enabled the identification of potential biomolecular targets for each compound, based on a high-percentage structural similarity between the synthesized and the established ligands. Two suggested biological targets that featured prominently for several of the synthesized compounds submitted to this profiling process were the cannabinoid receptors CB1 and CB2. CB1/2 are G-protein-coupled receptors and the human targets for the family of phytocannabinoids, isolated from cannabis, including tetrahydrocannabinol (THC), as well as for various synthetic drugs that mimic phytocannabinoid activity.

Given the structural overlap of 2-methoxyphenyl-comprising derivatives of a menthyl-type scaffold with the family of phytocannabinoids, as well as the documented ability of cannabinoid receptors to accommodate diverse synthetic ligands, including ones comprising nitrogen heteraromatics, we decided to computationally explore potential interactions between receptors CB1 and CB2 and the compounds of the series **6**/**ent-6**, **7**/**ent-7**, **8**/**ent-8** and **9**/**ent-9**. Modification of a previous protocol studying the same targets was employed [37]. The AutoDock Vina platform [38] was employed to generate in silico models of the established ligand binding sites of CB1 and CB2 receptors, based on binding site coordinates reported in association to crystal structures from the protein data bank (PDB). These coordinates are reported in Section 3.4. Our study employed CB1 crystal structure 5U09, where the protein is co-crystallized with ligand AM6538 [39], and CB2 crystal structure 5ZTY, where the protein is co-crystallized with ligand AM10257 [40]. The original ligands and any present additives were computationally removed, prior to the docking of the new compounds.

**Figure 8:**
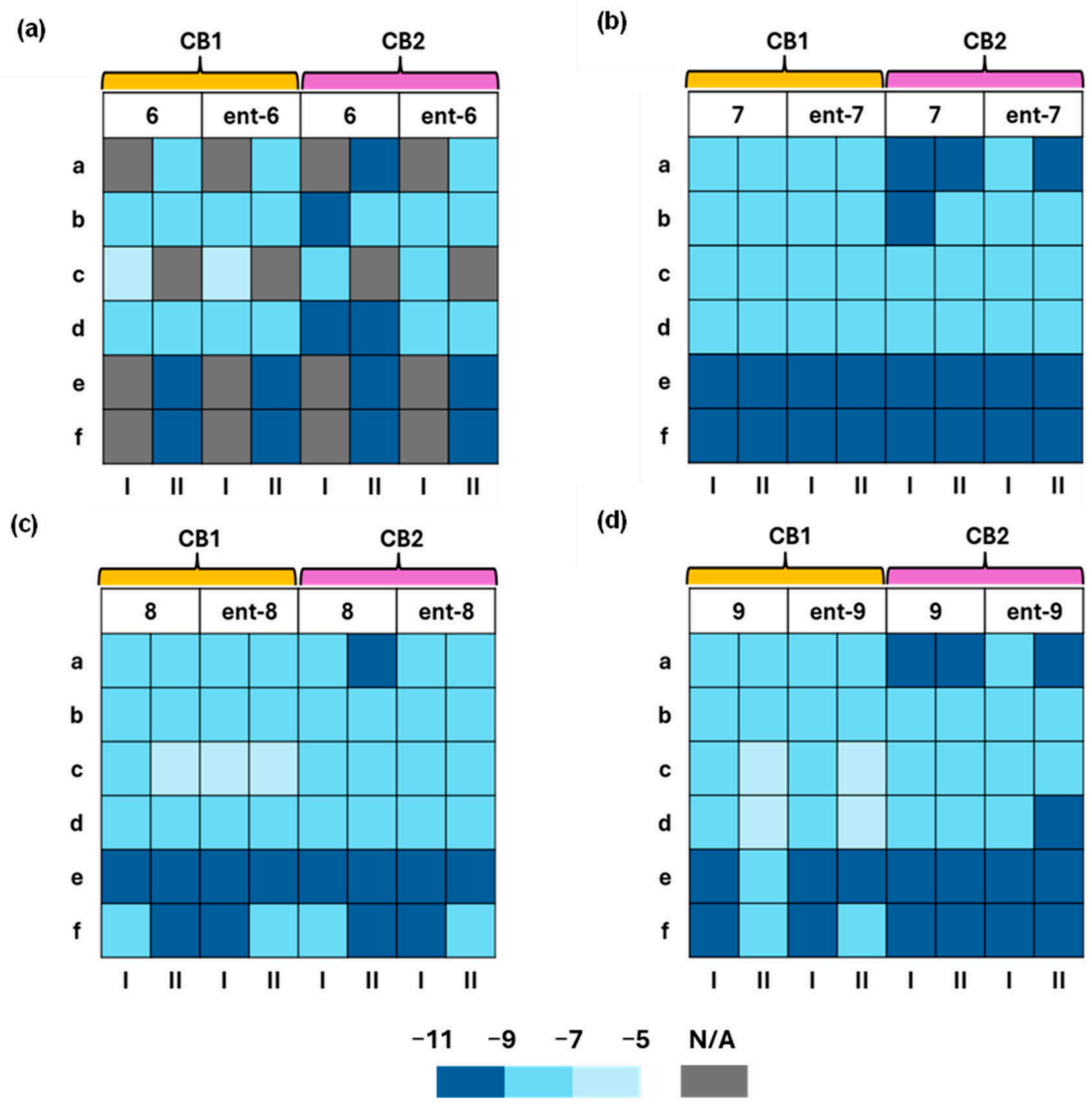
Classification of synthesized compounds as “strong” (−11 to −9 kcal.mol^−1^), “medium” (−9 to −7 kcal.mol^−1^) and “weak” (−7 to −5 kcal.mol^−1^) binders of CB1 (yellow) and CB2 (magenta) receptors, based on the ligand–protein complex energy score, as determined by a computational docking study using the AutoDock Vina 1.2.0 platform. Models of the protein targets were constructed based on crystal structures from PDB: 5U09-CB1 and 5ZTY-CB2. Models of the compounds were energy-minimized in Chem3D 23.0.1. Numerical values are shown in the Supplementary Materials. (**a**) Ligands of series **6**/**ent-6**; (**b**) ligands of series **7**/**ent-7**; (**c**) ligands of series **8**/**ent-8**; (**d**) ligands of series **9**/**ent-9**. Latin numbers I and II correspond to the two lowest-energy conformers of each compound. Where only one conformer resulted from the MM2 energy minimization iterations, the absent conformer is marked as not available (N/A).

An energy-minimized model of each new compound/ligand was constructed using the MM2 molecular mechanics algorithm in Chem3D 23.0.1 and placed in the CB1/2 receptor binding site for docking, within AutoDock Vina 1.2.0 [38]. For each compound, two conformers (termed I and II, see structures in Scheme 3) were studied, which represent the two lowest-energy local minima, and each of these was submitted to docking with the receptor independently. While in the case of hydrogenated compounds from series **8**/**ent-8** and **9**/**ent-9** the di(equatorial) or tri(equatorial) conformer (I), respectively, is considered to be the predominant contributor, in the case of Suzuki–Miyaura derivatives from series **6**/**ent-6** and **7**/**ent-7**, the two conformers (I and II) are found much closer in energy. On the contrary, in some other cases, the energy minimization converged to a single conformer, regardless of which initial conformer was input as the starting point for the MM2 minimization, which we interpreted as an indication of a substantial energy difference between the two, essentially rendering the absent conformer as a non-contributor (marked in Figure 8 as not available, N/A). Energy minimization iterations in AutoDock Vina eventually afforded the ligand–protein complex pose corresponding to the lowest energy state for each conformer examined. The resulting ligand–protein complex poses and the corresponding contacts/interactions are compiled in the Supplementary Materials. Based on their calculated binding affinity scores (in kcal.mol^−1^), the synthesized compounds were ranked in three (3) classes: (a) weak binders (score −5 to −7); (b) medium binders (score −7 to −9); and (c) strong binders (score −9 to −11). The collective results for all compounds included in this computational docking study are shown, color-coded, in Figure 8.

It should be noted that this study needs to be corroborated with experimental findings. Nonetheless, it helps identify some interesting trends, valuable for ligand design:

(a) The menthyl and *trans*-tetrahydrocarvoneyl terpene scaffolds are suitable for peripherally organizing and displaying substituents in 3D, thus affording hybrid terpenoid-(hetero)aromatic ligands with viable structural complementarity to the established binding sites of human cannabinoid receptors CB1 and CB2. This is evidenced by the fact that several of the synthesized compounds show satisfactory binding profiles towards both receptors.
(b) Some of the hybrid derivatives, owing to their diverse (hetero)aromatic substituents, may be suitable to probe the subtle differences between CB1 and CB2 receptors, as different binding affinities are suggested for each receptor variant.
(c) The expanded systems comprising nitrogen or oxygen bicycles (6-quinoline and 3-benzofuran, respectively) appear superior for interacting with the human cannabinoid receptors, across all compound sub-families tested, as can be concluded based on the behavior of ligands **6e**, **6f**, **7e**, **7f**, **8e**, **8f**, **9e**, **9f** and/or their enantiomers, which exhibit solid profiles of excellent binders. Their calculated binding affinities are, in fact, closely comparable to those of clinically used agonists (HU-210), partial agonists (THC) or antagonists (Rimonabant, HY13439) of CB1 and CB2, which were employed as controls in this computational study (see Supplementary Materials). The advantage of these bicyclic ring systems is mainly attributed to their enhanced propensity for π-π stacking, in conjunction with their ability for achieving diverse conformational poses, due to free rotation around their σ-bond with the terpene scaffold.
(d) Although good binders, according to the computational docking study, 2-methoxy- or 2,6-dimethoxy-substituted compounds are not excellent, which may suggest that their increased conformational flexibility relative to the conformationally rigid plant cannabinoids, that comprise the same phenolic oxygen substituent cyclized onto a menthyl scaffold, could be detrimental for recognition. Alternatively, a free hydroxy rather than methoxy group may be necessary, to enhance hydrogen-bonding ability.
(e) The parallel computational assessment of both enantiomers of the tested compounds is significant for identifying unique absolute stereochemical motifs, suitable for interaction optimization. While, in most cases, both enantiomers exhibited ability to interact with the targets, indicating the versatility of these receptors to accommodate hybrid small molecules with rotational flexibility pertaining to the σ-bond between terpene scaffold and aromatic moiety, in certain instances this ability was different, suggesting that the alternative binding orientations predicted for pairs of enantiomers can be critical for achieving optimal complementarity with the chiral target under investigation. Indicative examples include: **6a**, **6b** and **6d** vs. **ent-6a**, **ent-6b** and **ent-6d**, respectively, for CB2; **7a** and **7b** vs. **ent-7a** and **ent-7b**, respectively, for CB2; **8f** vs. **ent-8f** for CB1/2; and **9a** vs. **ent-9a** for CB2. The unequal binding affinities of enantiomers provide a window for further development into highly specific modulators for a particular target.

The computational docking simulation is revealing, not only for the ligand orientation within the binding sites of CB1 and CB2 receptors, but also for the types of interactions possible (see Supplementary Materials). Analyses of the docking complexes of the synthesized compounds against CB1 (PDB: 5U09) reveal a predominantly hydrophobic and aliphatic binding signature. Val196 is engaged in most cases, with its aliphatic side-chain packed against the ligand’s ring system, to provide VDW stabilization, critical for anchoring the hydrophobic core within the receptor’s transmembrane cavity. Met103 and Phe108 often contribute π-alkyl and π-sigma contacts. The flexible thioether side chain of Met103 conforms around the ligand, to maximize surface contact, while the aromatic ring of Phe108 provides directional stacking, useful for dictating ligand orientation within the binding pocket. Ala380, present in most complexes, forms compact alkyl contacts, restricting ligand mobility, while Ile105, Cys386 and Leu359 form a secondary hydrophobic wall that further encloses the ligand and limits solvent exposure. Phe268 contributes occasional π-π T-shaped and π-sigma interactions, important for stabilizing an alternative ligand orientation, and Ser383 appears in a single pose, corresponding to the polar residue known to hydrogen-bond with the native antagonist AM6538 in the original 5U09 structure [39], together with an aromatic cluster (Phe170, Phe174, Phe200, Trp279, Trp356, Phe379) that anchors the ligand head group and stabilizes the inactive receptor conformation. The overlap between our compounds’ contacts and this native aromatic cage/polar anchor system is limited to Phe268 and Ser383, meaning most of the binding energy in our docked compounds derives from a distinct hydrophobic sub-region (Val196, Met103, Phe108, Ala380, Ile105, Cys386, Leu359) rather than a full replication of the native ligand’s stabilizing network, thus identifying Val196, Met103, Phe108 and Ala380 as the priority residues driving binding in this compound series.

On the other hand, an analysis of the docking complexes generated for our synthesized ligands against CB2 (PDB: 5ZTY) reveals a consistent hydrophobic and aromatic binding profile. Phe94 and Phe183 function as central aromatic anchors, with their phenyl rings forming π-π stacking and π-alkyl interactions with the ligand, constraining its orientation within the binding pocket. Ile110 and Phe106, in most cases, reinforce this anchoring through hydrophobic and π-sigma contacts that pack against the ligand’s alkyl substituents, while His95 and Lys109 contribute additional π-alkyl interactions that help stabilize the ligand’s peripheral isopropyl and methyl groups, extending the contact surface toward the pocket entrance. A secondary network of Trp194, Trp258, Phe117 and Cys288 is engaged in poses adopting an alternative orientation. These large aromatic side chains, which provide strong π-π T-shaped and π-sigma stabilization, are structurally significant because Trp258 has been identified as a conformational “toggle switch”, that couples ligand engagement to the receptor-activation state. Hence, its involvement here may influence not just the binding affinity but also receptor downstream signaling behavior. Comparison with the native co-crystallized antagonist AM10257 [40] shows that Phe94, Phe117, Trp194, Trp258 and Phe183 are all established contact residues for the reference ligand, indicating that our compounds occupy the authentic orthosteric cavity and benefit from the same core-stabilizing aromatic cage that maintains ligand affinity in the native structure. In contrast, His95, Lys109, Ile110 and Phe106 are contacts more specific to our compound series, suggesting that these residues provide a supplementary stabilization mode, potentially compensating for reduced engagement elsewhere, and represent promising targets for structure–activity relationship optimization aimed at enhancing CB2 selectivity and potency.

**Figure 9:**
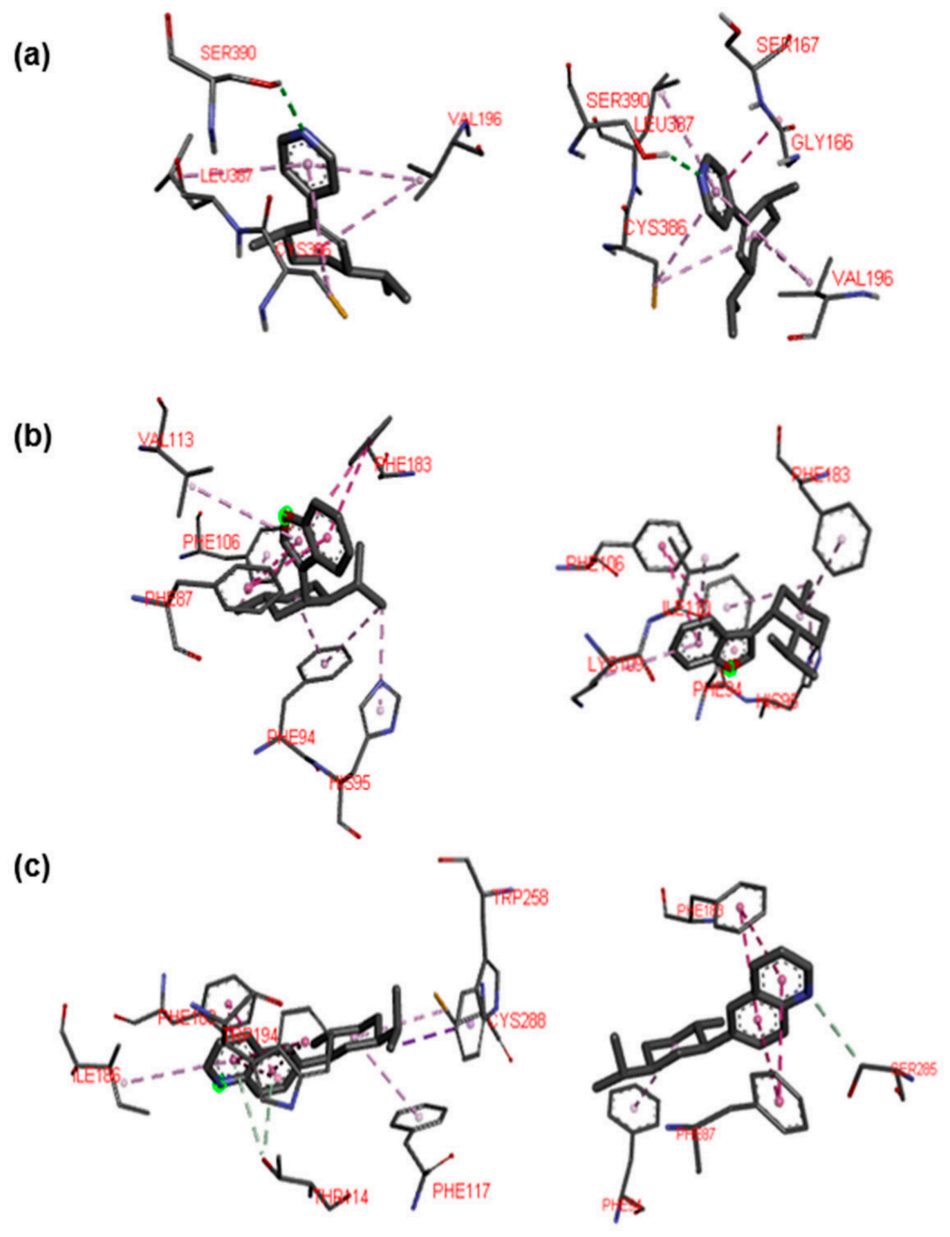
Representative energy-optimized AutoDock Vina-generated complexes of selected terpene–heteroaromatic enantiomeric ligands with human cannabinoid receptors: (**a**) Ligand **7d** (I) (**left**) vs. **ent-7d** (I) (**right**) in CB1; (**b**) ligand **8f** (I) (**left**) vs. **ent-8f** (I) (**right**) in CB2; and (**c**) ligand **9e** (I) (**left**) vs. **ent-9e** (I) (**right**) in CB2. Color-coding of atoms: C = grey; O = red; N = blue; S = yellow.

The predicted interactions are a direct consequence of ligand design, that combines the hydrophobicity of the 3D terpene scaffold with the absolute stereoconfiguration of the planar (heteroaromatic) substituent, to control its orientation, and the hydrogen bonding capability of the selected heteroaromatics or methoxy-substituted aromatic moieties. Overall, ligand design appears to be suitable for targeting CB1/2, allowing adaptability to the geometrical requirements of the binding sites. Representative pairs of enantiomers, in complex with the target receptors, are shown in Figure 9.

## 3. Materials and Methods

### 3.1. General

Organic chemicals and metal catalysts were purchased from TCI Europe N.V (Zwijndrecht, Belgium). or Sigma-Aldrich (St. Louis, MO, USA), anhydrous organic solvents from Carlo Erba Reagents (Val de Reuil, France) and NMR deuterated solvents from TCI Europe N.V. All reactions were performed under a nitrogen atmosphere, in anhydrous solvents, unless otherwise stated.

Thin-layer chromatography (TLC) was carried out on aluminum plates coated with silica gel 60 (F254) from Merck (Darmstadt, Germany), which allows fluorescence under a UV lamp (254 nm), with substances absorbing in the UV spectrum appearing as dark spots on a green background. For detection of substances lacking (strong) UV-absorbing chromophores, we employed vanillin dye for staining. For flash column chromatography, silica gel 60 (SiO_2_) 0.063–0.2 mm was used, acquired from Merck.

NMR spectra were obtained on a Bruker Avance III 500 Ultrashield Plus spectrometer (Billerica, MA, USA), at 500 MHz for ^1^H NMR and 125 MHz for ^13^C, at 25 °C. ^1^H and ^13^C NMR spectra were calibrated using the solvent’s residual peak, that served as an internal reference. Chemical shifts (*δ*) are reported in parts per million (ppm). FT-IR data were collected on ATR in the 4000–500 cm^−1^ range, using a Shimadzu Prestige-21 spectrometer (Kyoto, Japan). For determination of the specific optical rotation of chiral compounds using polarimetry, a Jasco P-2000 polarimeter (Tokyo, Japan) with a sodium vapor lamp (λ = 589 nm) was employed.

### 3.2. Synthetic Methods

#### 3.2.1. General Method for Synthesis of Enol Trifluoromethanesulfonate from Terpenoid Ketone (Synthesis of Compounds **3**/**ent-3**, **4**/**ent-4**)

A 25 mL round-bottom flask was charged with the terpenoid ketone (compound **1** or **2**) (1.6 mmol, 1 eq.) and PhNTf_2_ (2.1 mmol, 1.3 eq.), sealed with a rubber septum and, after purging out the air, set under a N_2_ atmosphere (ballon pressure). Anhydrous THF (3.2 mL) was added via syringe and the flask was cooled to −85 °C (EtOAc/liquid N_2_ mixture). LiHMDS (3.2 mL of a 1 M solution in THF, 2 eq.) was then added dropwise via syringe. Subsequently, the mixture was brought up to 0 °C (placement in an ice bath) and stirred for 30 min. After consumption of the starting material, as indicated by TLC, the mixture was quenched with aqueous saturated NH_4_Cl solution (50 mL) and extracted (3 × 50 mL) with EtOAc. The combined organic phase was dried over anhydrous Na_2_SO_4_ and, after removing the drying agent by filtration, the solvent was removed under vacuum on a rotary evaporator. The crude sample was redissolved in DCM (3 mL) and purified by silica gel flash chromatography, using hexane-DCM 20:1 as the elution solvent, to afford the enol trifluoromethanesulfonate product. Yields shown in Scheme 2.

#### 3.2.2. General Method for Suzuki–Miyaura C-C Cross-Coupling of Enol Trifluoromethanesulfonate and Arylboronic Acid (Synthesis of Compound Series **6**/**ent-6**, **7**/**ent-7**)

A 25 mL round-bottom flask was charged with enol trifluoromethanesulfonate (0.24 mmol, 1 eq.), aryl-boronic acid (compound of series **5**) (0.36 mmol, 1.5 eq.), K_2_CO_3_ (0.6 mmol, 2.5 eq.) and Pd(PPh_3_)_4_ (0.012 mmol, 0.05 eq.), fitted with a vertical condenser, sealed with a rubber septum and, after purging out the air, set under a N_2_ atmosphere (ballon pressure). A toluene–ethanol mixture (2:1, total of 4.5 mL) was added via syringe and the reaction was refluxed at 85 °C in an oil bath for 24 h, then allowed to cool down to room temperature. The mixture was diluted with EtOAc (50 mL) and washed, first with brine (50 mL) and then with water (50 mL). The aqueous phase was back-extracted with EtOAc (2 × 50 mL) and the combined organic phase was dried over anhydrous Na_2_SO_4_. After removal of the drying agent by filtration, the solvent was evaporated under vacuum on a rotary evaporator. The crude product was redissolved in DCM (3 mL) and purified by silica gel flash chromatography, to afford the C-C cross-coupling product. For compounds **6a**/**ent-6a**, **6f**/**ent-6f**, **7a**/**ent-7a**, and **7f**/**ent-7f**, the eluent was hexane; for compounds **6b**/**ent-6b**, **6c**/**ent-6c**, **7b**/**ent-7b**, and **7c**/**ent-7c**, the eluent was hexane-DCM step gradient (from 20:1 to 5:1); for compounds **6d**/**ent-6d**, **6e**/**ent-6e**, **7d**/**ent-7d**, and **7e**/**ent-7e**, the eluent was hexane-ethyl acetate 4:1. Yields are shown in Figure 4a,b.

#### 3.2.3. General Method for Obtaining Terpene–Aromatic Hybrid Compound via Catalytic Hydrogenation of C-C Cross-Coupled Substrate (Synthesis of Compound Series **8**/**ent-8**, **9**/**ent-9**)

In a 10 mL round-bottom flask, Pd/C powder (25 mg) was added to a solution of the C-C cross-coupled substrate (0.25 mmol, 1 eq.) in absolute EtOH (8.3 mL). The flask was sealed with a rubber septum and, by carefully bubbling H_2_ gas through the mixture with a long needle for 2–3 min at low pressure in a fume hood, it was purged of air through an exit needle. It was subsequently set under a H_2_ atmosphere (balloon pressure), with the gas inlet needle submerged in the liquid and vigorous stirring taking place for 24 h, at room temperature. After the mixture underwent filtration to remove the catalyst, the solvent was evaporated under vacuum on a rotary evaporator. The crude sample was redissolved in DCM (3 mL) and purified by silica gel flash chromatography, to afford the terpene–aromatic hybrid product. For compounds **8a**/**ent-8a**, **8f**/**ent-8f**, **9a**/**ent-9a**, and **9f**/**ent-9f**, the eluent was hexane; for compounds **8b**/**ent-8b**, **8c**/**ent-8c**, **9b**/**ent-9b**, and **9c**/**ent-9c**, the eluent was hexane-DCM step gradient (from 20:1 to 5:1); for compounds **8d**/**ent-8d** and **9d**/**ent-9d**, the eluent was hexane-ethyl acetate 4:1; for compounds **8e**/**ent-8e** and **9e**/**ent-9e**, the eluent was hexane-ethyl acetate step gradient (from 6:1 to 4:1). Yields are shown in Figure 4c,d.

### 3.3. ADMET Assessment of Precursors and Synthesized Compounds

SwissADME (https://www.swissadme.ch/, accessed on 20 July 2026) [34], ADMETLab 3.0 (https://admetlab3.scbdd.com/, accessed on 20 July 2026) [35] and pkCSM (https://biosig.lab.uq.edu.au/pkcsm/, accessed on 20 July 2026) [36] were used as web-based platforms/computational tools to evaluate the physicochemical, pharmacokinetic, and drug-likeness properties of small molecules during early-stage drug discovery. The structure of each compound, drawn in ChemDraw 23.0.1 (64-bit), was entered in each platform as input, transformed into SMILES string, selected parameters were calculated and the resulting predictions were used to prioritize candidates for further analysis.

### 3.4. Molecular Docking Study to Assess Ligand Binding Affinity for CB1/CB2 Receptors

The three-dimensional crystal structures of the human cannabinoid receptor types 1 and 2 (CB1 and CB2) were retrieved from the RCSB Protein Data Bank (PDB ID: 5U09 [39] and 5ZTY [40], respectively), having been selected based on high resolution criteria (2.6 Å and 2.8 Å, respectively). Prior to docking, the receptor structures were prepared using AutoDockToolsref (MGLTools 1.5.7, The Scripps Research Institute, La Jolla, CA, USA) [38]. Specifically, co-crystallized ligands, water molecules, buffer molecules, crystallographic lipids, and any fusion protein constructs were removed, polar hydrogens were added, and Gasteiger partial charges were assigned, before the structure was saved in a PDBQT format.

Three-dimensional structures of the small-molecule ligands from series **6**/**ent-6**, **7**/**ent-7**, **8**/**ent-8** and **9**/**ent-9** were constructed in ChemDraw 23.0.1 (64-bit), transferred to Chem3D 23.0.1 (64-bit) for geometry optimization using an MM2 force field, and exported as PDB files. Two alternative (flip) conformers of the aliphatic six-membered ring of each ligand (termed I and II) were investigated, corresponding to energy local minima, whose energy difference was either small or significant, depending on the actual ligand structure, except in those cases where MM2 iterations converged to one single conformer. Each ligand structure was subsequently prepared in AutoDockTools [38], by assigning rotatable bonds and Gasteiger charges, before conversion to a PDBQT format.

Molecular docking was performed using AutoDock Vina 1.2.0 (The Scripps Research Institute) [38], to investigate binding interactions between the synthesized ligands of series **6**/**ent-6**, 7/**ent-7**, **8**/**ent-8** and **9**/**ent-9**, and receptors CB1 and CB2. For receptor CB1, the docking search space was defined with a grid box centered at coordinates x = 22.509 Å, y = 0.285 Å, z = −4.502 Å, encompassing the orthosteric binding site of CB1, with grid dimensions of 27.29 × 29.79 × 31.85 Å along the x, y and z axes, respectively. For receptor CB2, the docking search space was defined with a grid box centered at coordinates x = 9.09 Å, y = −0.17 Å, z = −55.72 Å, encompassing the orthosteric binding site of CB2, with grid dimensions of 28.18 × 32.39 × 35.18 Å along the x, y and z axes, respectively. Docking was carried out with an exhaustiveness parameter of 8 and a maximum of 9 binding poses generated. The resulting docked conformations were extracted from the output PDBQT file using the Vina_Split utility [38] and converted to a PDB format, using Open Babel 3.1.1 for downstream analysis. Protein–ligand interaction analysis and visualization were performed in BIOVIA Discovery Studio Visualizer 2025, where the best-ranked docking pose (lowest energy, kcal.mol^−1^) was selected and evaluated for intermolecular interactions using the Show Interactions function. Publication-quality images were exported at 300 DPI with a white background (see Supplementary Materials).

## 4. Conclusions

The current approach exploits pharmacologically relevant, optically active, chiral pool monoterpenoids as starting precursors for the assembly of a focused compound library, whose members combine the cyclic terpene scaffolds with a series of pharmacologically privileged (hetero)aromatic ring systems, via a single σ-bond connection. Their molecular design allows the resulting structures to behave as modular ligands upon biomolecule binding.

The proposed synthetic path for accessing these hybrid structures entails novelty, since it leads, for menthyl-based compounds, to stereoisomers not previously attainable by any of the existing methods, and for *trans*-tetrahydrocarvoneyl-based compounds to the only methodology up to date for accessing them. Therefore, this process provides a practical and robust alternative to untapping the hidden biological potential of rare stereoisomers, while at the same time manages to circumvent the reliance on sensitive in situ-formed organometallic species that limits previous synthetic methods. Applying the proposed methodology affords enantiomerically pure products from chiral precursors, while eliminating the need for chiral separations, a necessary drawback of racemic syntheses.

The obtained molecular library is a novel entry in the area of terpenoid research, intended for systematic biological screening. In addition to including unprecedented hybrid-aromatic derivatives, with a 3D display of substituents on the periphery of the central terpene scaffold, it further benefits from the inclusion of pairs of enantiomers. Comparison of the enantiomers in their binding to specific targets will allow us to identify stereochemical motifs that are essential to activity enhancement.

A systematic computational study, based on three independent platforms, established the drug-like nature of the synthesized compounds, by assessing 11 essential physicochemical parameters, and highlighted compounds comprising 2,6-dimethoxyphenyl, 4-pyridyl or 6-quinolinyl aromatic on both terpene scaffolds as the ones most closely fulfilling the required pharmacological profile for cannabinoid receptor targeting. Furthermore, the docking study led to the identification of 6-quinolinyl-based compounds as optimal candidates for further development, based on calculated binding energy scores, and provided a structural basis for selection of suitable fragments for optimizing affinity and selectivity. In conjunction with the in silico ADMET assessment, the docking study could be a useful supporting tool for prioritizing compound candidates with viable target engagement profiles, for further pharmacological and pharmacokinetic advancement.

Plans for future work will expand the range of terpene scaffolds and heteroaromatic moieties, will attempt to combine dimethoxy-substitution and bicyclic aromatics to achieve synergies between pharmacokinetic properties and target affinity, and will evaluate the synthesized compounds in cells overexpressing the human cannabinoid receptors, in order to obtain and cross-reference experimental results with computational predictions.

## Data Availability

Data supporting reported results can be found in the Supplementary Materials.

## Supplementary Materials

The following supplementary materials can be downloaded at https://www.mdpi.com/article/10.3390/molecules31152599/s1: I. Synthesized (Isolated) Compound inventory; II. Compound characterization data; III. Compound 1D and 2D NMR spectra; IV. Calculated ^1^H and ^13^C NMR spectra; V. ADMET Computational study of synthesized compounds and known CB1/2 ligands; VI. Molecular docking of ketone precursors and synthesized compounds against cannabinoid receptors CB1 and CB2: optimal complex poses and intermolecular interactions; VII. Molecular docking of known modulators of cannabinoid receptors CB1 and CB2.

## Abbreviations

The following abbreviations are used in this manuscript:

ABTS	2,2′-Azino-bis(3-ethylbenzothiazoline-6-sulfonic acid)
Ac	Acetyl
ADME(T)	Absorption, distribution, metabolism, excretion, (toxicity)
AKT	Protein kinase B
ATR	Attenuated total reflectance
CB	Cannabinoid receptor
CBD	Cannabidiol
D	Dimension
DCM	Dichloromethane
DPI	Dots per inch
DPPH	2,2-Diphenyl-1-picrylhydrazyl
Ent	Enantiomer
Et	Ethyl
FT-IR	Fourier transform infrared
GI	Gastrointestinal
HF-DFT	Hartree-Fock density functional theory
HL	Human leukemia cell line
IBD	Inflammatory bowel disease
ID	Identification code
IL	Interleukin cytokine
LHMDS	Lithium hexamethyldisilazane
LNCaP	Lymph node carcinoma of the prostate cell line
LPS	Lipopolysaccharide
MGL	Molecular graphics laboratory
MITF	Microphthalmia-associated transcription factor
MM	Molecular mechanics
MRSA	Methicillin-resistant *Staphylococcus aureus*
mTOR	Mechanistic target of rapamycin
MW	Molecular weight
N/A	Not available
NF-κB	Nuclear factor kappa B
NMR	Nuclear magnetic resonance
ORAC	Oxygen radical absorbance capacity
P-gp	P-glycoprotein
PC	Prostate cancer cell line
PDB	Protein data bank
PDBQT	Protein data bank + partial charge (Q) + atom type (T)
PEO	Peppermint essential oil
Ph	Phenyl
ppm	Parts per million
RCSB	Research collaboratory for structural bioinformatics
SCF	Secure controls framework
SMILES	Simplified molecular input line entry system
Tf	Trifluoromethanesulfonyl
THC	Δ^9^-Tetrahydrocannabinol
THCBD	Tetrahydrocannabidiol
THF	Tetrahydrofuran
TLC	Thin layer chromatography
TNF-α	Tumor necrosis factor-alpha
TPSA	Topological polar surface area
TRPA	Transient receptor potential ankyrin channel
TRPM	Transient receptor potential melastatin channel
TRPV	Transient receptor potential vanilloid channel
UV	Ultraviolet
VDW	Van der Waals interactions
VLA	Very late activation antigen
VRE	Vancomycin-resistant enterococcus
