# Supplementary material for: Cyclic Monoterpene–Aromatic Hybrids from Chiral Pool Terpenoid Ketones: Practical Synthetic Methodology for Accessing Them and In Silico Assessment as Cannabinoid Receptor CB1/CB2 Ligands
Source: Molecules. 2026 Jul 25;31(15):2599. doi: 10.3390/molecules31152599 (PMC13467945; doi:10.3390/molecules31152599)
Supplement: Supplementary file 1 [file molecules-31-02599-s001.zip › molecules-4407798-SI update.pdf]

# Supplementary Materials

## Cyclic Monoterpene–Aromatic Hybrids from Chiral Pool Terpenoid Ketones: Practical Synthetic Methodology for Accessing Them and In Silico Assessment as Cannabinoid Receptor CB1/CB2 Ligands

Vasiliki Kaikiti,[1] Andrea Jaksic,[1,†] Basharat Ali,[2] Savvas N. Georgiades[1,*]

* Corresponding author: Dr Savvas N. Georgiades; e-mail: georgiades.savvas@ucy.ac.cy

[1] Department of Chemistry, University of Cyprus, 1 Panepistimiou Avenue, Nicosia 2109, Cyprus

[2] Institute of Chemistry, Khawaja Fareed University of Engineering and Information Technology, Rahim Yar Khan 64200, Pakistan

[†] Current address: Department of Chemistry, Utrecht University, 3584 CS Utrecht, The Netherlands

**CONTENTS**

I. Synthesized (Isolated) Compound Inventory S-2

II. Compound Characterization Data S-3

III. Compound 1D and 2D NMR Spectra S-21

IV. Calculated $^1$H and $^{13}$C NMR Spectra S-84

V. ADMET Computational Study of Synthesized Compounds and Known CB1/2 Ligands S-88

VI. Molecular Docking of Ketone Precursors and Synthesized Compounds Against Cannabinoid Receptors CB1 and CB2: Optimal Complex Poses and Intermolecular Interactions S-103

VII. Molecular Docking of Known Modulators of Cannabinoid Receptors CB1 and CB2 S-195

### I. Synthesized (Isolated) Compound Inventory

| Compound | Known/New | Compound | Known/New |
|---|---|---|---|
| | | | |
| **(-)-1** | known | **(+)-1** | known |
| **(-)-2** | known | **(+)-2** | known |
| **3** | known | **ent-3** | known |
| **4** | known | **ent-4** | new |
| **6a** | new | **ent-6a** | new |
| **6b** | new | **ent-6b** | new |
| **6c** | new | **ent-6c** | new |
| **6d** | new | **ent-6d** | new |
| **6e** | new | **ent-6e** | new |
| **6f** | new | **ent-6f** | new |
| **7a** | new | **ent-7a** | new |
| **7b** | new | **ent-7b** | new |
| **7c** | new | **ent-7c** | new |
| **7d** | new | **ent-7d** | new |
| **7e** | new | **ent-7e** | new |
| **7f** | new | **ent-7f** | new |
| **8a** | new | **ent-8a*** | new |
| **8b**** | new | **ent-8b** | new |
| **8c** | new | **ent-8c** | new |
| **8d** | new | **ent-8d** | new |
| **8e** | new | **ent-8e** | new |
| **8f** | new | **ent-8f** | new |
| **9a** | new | **ent-9a** | new |
| **9b** | new | **ent-9b** | new |
| **9c** | new | **ent-9c** | new |
| **9d** | new | **ent-9d** | new |
| **9e** | new | **ent-9e** | new |
| **9f** | new | **ent-9f** | new |

* (1*S*,2*R*,5*S*) diastereomer known

** (1*R*,2*S*,5*R*) diastereomer known

Notes:

- Enantiomers are formed in independent reactions, each starting from an optically pure precursor.
- Only the major diastereomer was isolated from each hydrogenation reaction (series **8**, **ent-8**, **9**, **ent-9**). The minor diastereomer could not be isolated or characterized.

## II. Compound Characterization Data

**• (3*R*,6*S*)-6-Isopropyl-3-methylcyclohex-1-en-1-yl trifluoromethanesulfonate (3):**

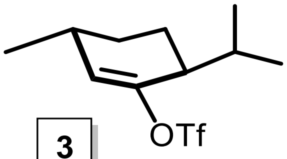

**3**

Produced from substrate **(-)-1**. Colorless oil. Yield: 76%. $^{1}$H NMR (500 MHz, $CDCl_3$): δ (ppm) 5.64 (bs, 1H), 2.48 (m, 1H), 2.33 (m, 1H), 2.15 (m, 1H), 1.81 (m, 2H, 2 signals overlapping), 1.42 (m, 1H), 1.13 (m, 1H), 1.04 (d, 3H, *J* = 7.2 Hz), 0.95 (d, 3H, *J* = 6.9 Hz), 0.82 (d, 3H, *J* = 6.9 Hz). $^{13}$C NMR (125 MHz, $CDCl_3$): δ (ppm) 151.8, 125.9, 118.6 (q, $CF_3$, *J* = 319.9 Hz), 43.1, 30.6, 29.9, 27.3, 22.5, 21.3, 19.9, 16.3. FT-IR: v ($cm^{-1}$) 2962 (m), 2932 (m), 2875 (m), 1674 (w), 1415 (s), 1246 (s), 1203 (s), 1142 (s), 1045 (w), 974 (m), 944 (m), 893 (s), 873 (m), 817 (m). $[a]^{23}_{D}$ +4.8 (*c* 0.7, $CH_2Cl_2$).

**• (3*S*,6*R*)-6-Isopropyl-3-methylcyclohex-1-en-1-yl trifluoromethanesulfonate (ent-3):**

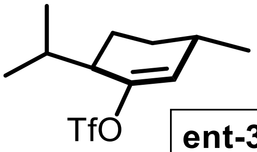

**ent-3**

Produced from substrate **(+)-1**. Colorless oil. Yield: 75%. $^{1}$H NMR (500 MHz, $CDCl_3$): δ (ppm) 5.64 (bs, 1H), 2.48 (m, 1H), 2.33 (m, 1H), 2.15 (m, 1H), 1.81 (m, 2H, 2 signals overlapping), 1.42 (m, 1H), 1.13 (m, 1H), 1.04 (d, 3H, *J* = 7.2 Hz), 0.95 (d, 3H, *J* = 6.9 Hz), 0.82 (d, 3H, *J* = 6.9 Hz). $^{13}$C NMR (125 MHz, $CDCl_3$): δ (ppm) 151.8, 125.9, 118.6 (q, $CF_3$, *J* = 319.9 Hz), 43.1, 30.6, 29.9, 27.3, 22.5, 21.3, 19.9, 16.3. FT-IR: v ($cm^{-1}$) 2962 (m), 2932 (m), 2875 (m), 1674 (w), 1415 (s), 1246 (s), 1203 (s), 1142 (s), 1045 (w), 974 (m), 944 (m), 893 (s), 873 (m), 817 (m). $[a]^{23}_{D}$ -5.3 (*c* 0.8, $CH_2Cl_2$).

**• (3*S*,6*R*)-3-Isopropyl-6-methylcyclohex-1-en-1-yl trifluoromethanesulfonate (4):**

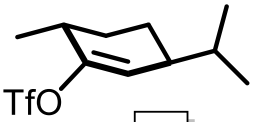

**4**

Produced from substrate **(+)-2**. Colorless oil. Yield: 76%. $^{1}$H NMR (500 MHz, $CDCl_3$): δ (ppm) 5.64 (d, 1H, *J* = 2.7 Hz), 2.49 (m, 1H), 2.15 (m, 1H), 1.84 (ddt, 1H, $J_1$ = 12.7 Hz, $J_2$ = 5.9 Hz, $J_3$ = 3.1 Hz), 1.60-1.70 (m, 2H, 2 signals overlapping), 1.56 (m, 1H), 1.39 (m, 1H), 1.14 (d, 3H, *J* = 7.0 Hz), 0.92 (d, 3H, *J* = 6.6 Hz), 0.90 (d, 3H, *J* = 6.6 Hz). $^{13}$C NMR (125 MHz, $CDCl_3$): δ (ppm) 153.7, 121.2, 118.5 (q, $CF_3$, *J* = 319.2 Hz), 42.0, 31.9, 31.7, 29.5, 20.3, 19.6, 19.4, 17.9. FT-IR: v ($cm^{-1}$) 2963 (w), 2875 (w), 1652 (w), 1576 (w), 1560 (w), 1541 (w), 1508 (w), 1457 (w), 1414 (w), 1246 (w), 1203 (m), 1141 (m), 1038 (w), 1004 (w), 938 (w), 882 (m), 863 (w), 811 (w). $[a]^{23}_{D}$ -29.7 (*c* 1.4, $CH_2Cl_2$).

• **(3*R*,6*S*)-3-Isopropyl-6-methylcyclohex-1-en-1-yl trifluoromethanesulfonate (ent-4):**

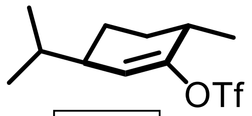


**ent-4**

Produced from substrate **(-)-2**. Colorless oil. Yield: 74%. $^1$H NMR (500 MHz, $CDCl_3$): δ (ppm) 5.64 (d, 1H, *J* = 2.7 Hz), 2.49 (m, 1H), 2.15 (m, 1H), 1.84 (ddt, 1H, $J_1$ = 12.7 Hz, $J_2$ = 5.9 Hz, $J_3$ = 3.1 Hz), 1.60-1.70 (m, 2H, 2 signals overlapping), 1.56 (m, 1H), 1.39 (m, 1H), 1.14 (d, 3H, *J* = 7.0 Hz), 0.92 (d, 3H, *J* = 6.6 Hz), 0.90 (d, 3H, *J* = 6.6 Hz). $^{13}$C NMR (125 MHz, $CDCl_3$): δ (ppm) 153.7, 121.2, 118.5 (q, $CF_3$, *J* = 319.2 Hz), 42.0, 31.9, 31.7, 29.5, 20.3, 19.6, 19.4, 17.9. FT-IR: ν ($cm^{-1}$) 2963 (w), 2875 (w), 1652 (w), 1576 (w), 1560 (w), 1541 (w), 1508 (w), 1457 (w), 1414 (w), 1246 (w), 1203 (m), 1141 (m), 1038 (w), 1004 (w), 938 (w), 882 (m), 863 (w), 811 (w). $[a]^{23}_D$ +26.5 (*c* 1.2, $CH_2Cl_2$).

• **(2*S*,5*R*)-2-Isopropyl-5-methyl-2,3,4,5-tetrahydro-1,1'-biphenyl (6a):**

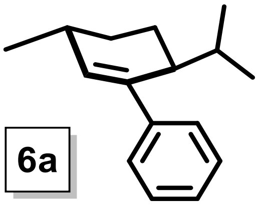

**6a**

Produced from substrates **3** and **5a**. Colorless oil. Yield: 52%. $^1$H NMR (500 MHz, $CDCl_3$): δ (ppm) 7.24-7.32 (m, 4H, 2 signals overlapping), 7.20 (tt, 1H, $J_1$ = 7.1 Hz, $J_2$ = 1.9 Hz), 5.67 (bs, 1H), 2.71 (m, 1H), 2.22 (m, 1H), 1.80-1.90 (m, 3H, 3 signals overlapping), 1.47 (m, 1H), 1.17 (m, 1H), 1.01 (d, 3H, *J* = 7.1 Hz), 0.88 (d, 3H, *J* = 6.9 Hz), 0.58 (d, 3H, *J* = 6.9 Hz). $^{13}$C NMR (125 MHz, $CDCl_3$): δ (ppm) 143.5, 141.6, 135.0, 128.0, 126.6, 126.1, 41.6, 31.3, 31.0, 28.4, 22.1, 21.6, 20.7, 15.6. FT-IR: ν ($cm^{-1}$) 2954 (m), 2918 (s), 2850 (m), 2360 (m), 2344 (w), 2162 (w), 2032 (w), 1738 (w), 1647 (w), 1639 (w), 1563 (w), 1048 (w). $[a]^{23}_D$ +16.5 (*c* 0.3, $CH_2Cl_2$).

• **(2*S*,5*R*)-2-Isopropyl-2'-methoxy-5-methyl-2,3,4,5-tetrahydro-1,1'-biphenyl (6b):**

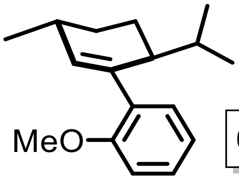


**6b**

Produced from substrates **3** and **5b**. Colorless oil. Yield: 90%. $^1$H NMR (500 MHz, $CDCl_3$): δ (ppm) 7.20 (dt, 1H, $J_1$ = 7.9 Hz, $J_2$ = 1.8 Hz), 7.13 (dd, 1H, $J_1$ = 7.3 Hz, $J_2$= 1.8 Hz), 6.90 (t, 1H, *J* = 7.5 Hz), 6.82 (d, 1H, *J* = 8.1 Hz), 5.56 (bs, 1H), 3.80 (s, 3H), 2.86 (m, 1H), 2.24 (m, 1H), 1.87 (m, 1H), 1.77 (m, 1H), 1.58 (m, 1H), 1.38 (m, 1H), 1.22 (m, 1H), 1.01 (d, 3H, *J* = 7.2 Hz), 0.85 (d, 3H, *J* = 6.9 Hz), 0.57 (d, 3H, *J* = 6.9 Hz). $^{13}$C NMR (125 MHz, $CDCl_3$): δ (ppm) 156.7, 141.1, 135.4, 132.9, 130.3, 127.4, 120.6, 110.5, 55.4, 42.2, 31.7, 31.5, 28.7, 22.1, 21.9, 21.0, 15.8. FT-IR: ν ($cm^{-1}$) 2953 (s), 2926 (s), 2867 (m), 2834 (w), 1597 (w), 1577 (w), 1488 (m), 1460 (m), 1434 (m), 1383 (w), 1365 (w), 1290 (w), 1246 (s), 1230 (m), 1179 (w), 1115 (m), 1052 (w), 1029 (m), 863 (w), 780 (w), 748 (s). $[a]^{23}_D$ +3.5 (*c* 0.6, $CH_2Cl_2$).

• **(2*S*,5*R*)-2-Isopropyl-2',6'-dimethoxy-5-methyl-2,3,4,5-tetrahydro-1,1'-biphenyl (6c):**

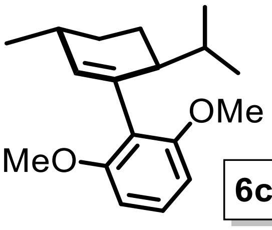

**6c**

Produced from substrates **3** and **5c**. Colorless oil. Yield 65%. $^{1}$H NMR (500 MHz, $CDCl_3$): δ (ppm) 7.13 (t, 1H, *J* = 8.3 Hz), 6.54 (bd, 2H, *J* = 8.3 Hz), 5.47 (bs, 1H), 3.76 (s, 6H), 2.68 (m, 1H), 2.32 (m, 1H), 1.88 (m, 1H), 1.75 (m, 1H), 1.50 (m, 1H), 1.46 (m, 1H), 1.25 (m, 1H), 0.99 (d, 3H, *J* = 7.1 Hz), 0.82 (m, 3H, *J* = 6.9 Hz), 0.67 (d, 3H, *J* = 6.9 Hz). $^{13}$C NMR (125 MHz, $CDCl_3$): δ (ppm) 158.0, 135.9, 134.3, 127.1, 121.3, 104.4 (app. d), 55.9 (app. d), 43.0, 31.6, 31.4, 29.0, 22.1, 21.9, 21.5, 16.3. FT-IR: ν ($cm^{-1}$) 2952 (m), 2932 (m), 2869 (m), 2836 (m), 1587 (s), 1465 (s), 1442 (m), 1431 (s), 1382 (w), 1280 (w), 1240 (s), 1117 (w), 1102 (s), 1035 (m), 956 (w), 862 (w), 782 (s), 756 (w), 732 (s). $[a]^{23}_{D}$ +3.2 (*c* 1.1, $CH_2Cl_2$).

• **4-((3*R*,6*S*)-6-Isopropyl-3-methylcyclohex-1-en-1-yl)pyridine (6d):**

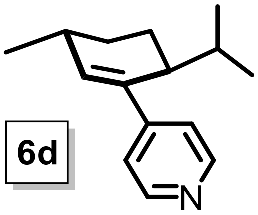

**6d**

Produced from substrates **3** and **5d**. Colorless oil. Yield: 63%. $^{1}$H NMR (500 MHz, $CDCl_3$): δ (ppm) 8.50 (d, 2H, *J* = 5.2 Hz), 7.20 (d, 2H, *J* = 5.2 Hz), 5.85 (bs, 1H), 2.69 (m, 1H), 2.24 (m, 1H), 1.78-1.88 (m, 3H, 3 signals overlapping), 1.47 (m, 1H), 1.15 (m, 1H), 1.03 (d, 3H, *J* = 7.1 Hz), 0.88 (d, 3H, *J* = 6.9 Hz), 0.56 (d, 3H, *J* = 6.9 Hz). $^{13}$C NMR (125 MHz, $CDCl_3$): δ (ppm) 152.1, 148.5, 139.6, 138.3, 121.8, 41.0, 31.4, 30.5, 28.4, 21.7, 21.3, 20.6, 15.7. FT-IR: ν ($cm^{-1}$) 2959 (s), 2936 (s), 2872 (m), 2850 (m), 1715 (w), 1597 (s), 1544 (w), 1465 (m), 1412 (m), 1366 (m), 1219 (w), 1108 (w), 1062 (w), 992 (w), 819 (m). $[a]^{23}_{D}$ +8.5 (*c* 0.9, $CH_2Cl_2$).

• **6-((3*R*,6*S*)-6-Isopropyl-3-methylcyclohex-1-en-1-yl)quinoline (6e):**

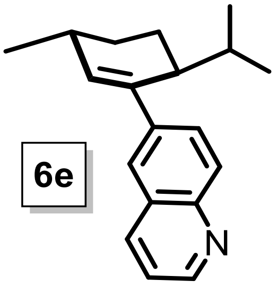

**6e**

Produced from substrates **3** and **5e**. White crystals. Yield: 99%. $^{1}$H NMR (500 MHz, $CDCl_3$): δ (ppm) 8.86 (d, 1H, *J* = 4.3 Hz), 8.16 (d, 1H, *J* = 8.4 Hz), 8.08 (d, 1H, *J* = 8.4 Hz), 7.65-7.71 (m, 2H, 2 peaks overlapping), 7.40 (dd, 1H, $J_1$ = 8.4 Hz, $J_2$ = 4.3 Hz), 5.83 (bs, 1H), 2.82 (m, 1H), 2.28 (m, 1H), 1.80-1.94 (m, 3H, 3 signals overlapping), 1.51 (m, 1H), 1.21 (m, 1H), 1.04 (d, 3H, *J* = 7.1 Hz), 0.89 (d, 3H, *J* = 6.9 Hz), 0.59 (d, 3H, *J* = 6.9 Hz). $^{13}$C NMR (125 MHz, $CDCl_3$): δ (ppm) 149.0, 146.6, 142.2, 141.0, 136.7, 136.5, 129.7, 128.4, 128.3, 124.4, 121.1, 41.6, 31.5, 30.9, 28.6, 22.0, 21.6, 20.7, 15.7. FT-IR: ν ($cm^{-1}$) 2956 (m), 2923 (m), 2885 (m), 2867 (m), 2846 (m), 2831 (m), 1720 (w), 1589 (m), 1494 (m), 1385 (m), 1366 (m), 1120 (w), 945 (w), 883 (w), 875 (m), 840 (s), 794 (m), 777 (m). $[a]^{23}_{D}$ +2.6 (*c* 0.4, $CH_2Cl_2$).

• **3-((3*R*,6*S*)-6-Isopropyl-3-methylcyclohex-1-en-1-yl)benzofuran (6f):**

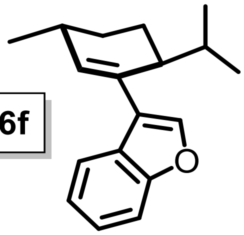

**6f**

Produced from substrates **3** and **5f**. Colorless oil. Yield: 81%. $^{1}$H NMR (500 MHz, $CDCl_3$): δ (ppm) 7.66 (d, 1H, *J* = 7.7 Hz), 7.48 (s, 1H), 7.47 (d, 1H, *J* = 7.5 Hz), 7.28 (t, 1H, *J* = 7.4 Hz), 7.24 (t, 1H, *J* = 7.5 Hz), 5.90 (bs, 1H), 2.66 (m, 1H), 2.29 (m, 1H), 1.80-2.00 (m, 3H, 3 signals overlapping), 1.50 (m, 1H), 1.24 (m, 1H), 1.02 (d, 3H, *J* = 7.1 Hz), 0.88 (d, 3H, *J* = 6.9 Hz), 0.64 (d, 3H, *J* = 6.9 Hz). $^{13}$C NMR (125 MHz, $CDCl_3$): δ (ppm) 155.3, 140.7, 135.4, 132.2, 127.4, 124.0, 122.9, 122.5, 120.7, 111.5, 42.4, 31.0, 30.9, 28.9, 22.0, 21.6, 20.8, 16.1. FT-IR: v ($cm^{-1}$) 2961 (s), 2928 (s), 2870 (s), 1763 (m), 1604 (m), 1460 (s), 1382 (w), 1329 (w), 1242 (w), 1077 (s), 1047 (s), 879 (m), 746 (s). $[a]^{23}_{D}$ +5.7 (*c* 0.2, $CH_2Cl_2$).

• **(2*R*,5*S*)-2-Isopropyl-5-methyl-2,3,4,5-tetrahydro-1,1'-biphenyl (ent-6a):**

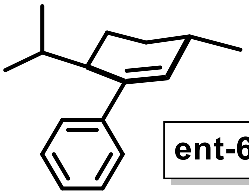

**ent-6a**

Produced from substrates **ent-3** and **5a**. Colorless oil. Yield: 60%. $^{1}$H NMR (500 MHz, $CDCl_3$): δ (ppm) 7.24-7.32 (m, 4H, 2 signals overlapping), 7.20 (tt, 1H, $J_1$ = 7.1 Hz, $J_2$ = 1.9 Hz), 5.67 (bs, 1H), 2.71 (m, 1H), 2.22 (m, 1H), 1.80-1.90 (m, 3H, 3 signals overlapping), 1.47 (m, 1H), 1.17 (m, 1H), 1.01 (d, 3H, *J* = 7.1 Hz), 0.88 (d, 3H, *J* = 6.9 Hz), 0.58 (d, 3H, *J* = 6.9 Hz). $^{13}$C NMR (125 MHz, $CDCl_3$): δ (ppm) 143.5, 141.6, 135.0, 128.0, 126.6, 126.1, 41.6, 31.3, 31.0, 28.4, 22.1, 21.6, 20.7, 15.6. FT-IR: v ($cm^{-1}$) 2954 (m), 2918 (s), 2850 (m), 2360 (m), 2344 (w), 2162 (w), 2032 (w), 1738 (w), 1647 (w), 1639 (w), 1563 (w), 1048 (w). $[a]^{23}_{D}$ -17.2 (*c* 1.01, $CH_2Cl_2$).

• **(2*R*,5*S*)-2-Isopropyl-2'-methoxy-5-methyl-2,3,4,5-tetrahydro-1,1'-biphenyl (ent-6b):**

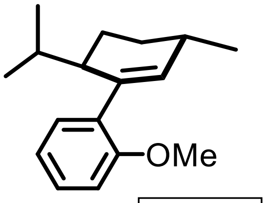


**ent-6b**

Produced from substrate **ent-3** and **5b**. Yellow oil. Yield: 80%. $^{1}$H NMR (500 MHz, $CDCl_3$): δ (ppm) 7.20 (dt, 1H, $J_1$ = 7.9 Hz, $J_2$ = 1.9 Hz), 7.13 (dd, 1H, $J_1$ = 7.4 Hz, $J_2$= 1.7 Hz), 6.90 (t, 1H, *J* = 7.5 Hz), 6.82 (d, 1H, *J* = 8.1 Hz), 5.56 (bs, 1H), 3.80 (s, 3H), 2.86 (m, 1H), 2.24 (m, 1H), 1.87 (m, 1H), 1.77 (m, 1H), 1.58 (m, 1H), 1.38 (m, 1H), 1.22 (m, 1H), 1.01 (d, 3H, *J* = 7.2 Hz), 0.85 (d, 3H, *J* = 6.9 Hz), 0.57 (d, 3H, *J* = 6.9 Hz). $^{13}$C NMR (125 MHz, $CDCl_3$): δ (ppm) 156.7, 141.1, 135.4, 132.9, 130.3, 127.4, 120.6, 110.5, 55.4, 42.2, 31.7, 31.5, 28.7, 22.1, 21.9, 21.0, 15.8. FT-IR: v ($cm^{-1}$) 2955 (s), 2923 (s), 2869 (m), 2850 (m), 1597 (w), 1488 (m), 1461 (s), 1434 (m), 1378 (m), 1247 (s), 1230 (m), 1178 (w), 1115 (m), 1097 (w), 1049 (m), 1029 (s), 803 (m) 748 (s). $[a]^{23}_{D}$ -4.0 (*c* 0.5, $CH_2Cl_2$).

- **(2*R*,5*S*)-2-Isopropyl-2',6'-dimethoxy-5-methyl-2,3,4,5-tetrahydro-1,1'-biphenyl (ent-6c):**

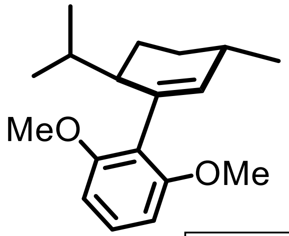

**ent-6c**

Produced from substrates **ent-3** and **5c**. White solid. Yield: 56%. $^1$H NMR (500 MHz, $CDCl_3$): δ (ppm) 7.13 (t, 1H, *J* = 8.3 Hz), 6.54 (bd, 2H, *J* = 8.3 Hz), 5.47 (bs, 1H), 3.76 (s, 6H), 2.68 (m, 1H), 2.32 (m, 1H), 1.88 (m, 1H), 1.75 (m, 1H), 1.50 (m, 1H), 1.46 (m, 1H), 1.25 (m, 1H), 0.99 (d, 3H, *J* = 7.1 Hz), 0.82 (m, 3H, *J* = 6.9 Hz), 0.67 (d, 3H, *J* = 6.9 Hz). $^{13}$C NMR (125 MHz, $CDCl_3$): δ (ppm) 158.0, 135.9, 134.3, 127.1, 121.3, 104.4 (app. d), 55.9 (app. d), 43.0, 31.6, 31.4, 29.0, 22.1, 21.9, 21.5, 16.3. FT-IR: ν ($cm^{-1}$) 2952 (m), 2934 (m), 2870 (w), 2836 (w), 1589 (s), 1465 (s), 1442 (m), 1431 (s), 1382 (w), 1280 (w), 1240 (s), 1171 (w), 1128 (w), 1103 (s), 1037 (m), 958 (w), 863 (w). 783 (s), 733 (s). $[a]^{23}_D$ -3.9 (*c* 0.7, $CH_2Cl_2$).

- **4-((3*S*,6*R*)-6-Isopropyl-3-methylcyclohex-1-en-1-yl)pyridine (ent-6d):**

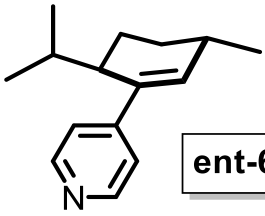

**ent-6d**

Produced from substrates **ent-3** and **5d**. Colorless oil. Yield 70%. $^1$H NMR (500 MHz, $CDCl_3$): δ (ppm) 8.50 (d, 2H, *J* = 5.2 Hz), 7.20 (d, 2H, *J* = 5.2 Hz), 5.85 (bs, 1H), 2.69 (m, 1H), 2.24 (m, 1H), 1.78-1.88 (m, 3H, 3 signals overlapping), 1.47 (m, 1H), 1.15 (m, 1H), 1.03 (d, 3H, *J* = 7.1 Hz), 0.88 (d, 3H, *J* = 6.9 Hz), 0.56 (d, 3H, *J* = 6.9 Hz). $^{13}$C NMR (125 MHz, $CDCl_3$): δ (ppm) 152.1, 148.5, 139.6, 138.3, 121.8, 41.0, 31.4, 30.5, 28.4, 21.7, 21.3, 20.6, 15.7. FT-IR: ν ($cm^{-1}$) 2960 (s), 2933 (s), 2871 (s), 1713 (m), 1687 (m), 1597 (s), 1544 (w), 1465 (m), 1411 (s), 1386 (w), 1366 (s), 1219 (m), 1065 (m), 994 (m), 819 (m). $[a]^{23}_D$ -7.6 (*c* 1, $CH_2Cl_2$).

- **6-((3*S*,6*R*)-6-Isopropyl-3-methylcyclohex-1-en-1-yl)quinoline (ent-6e):**

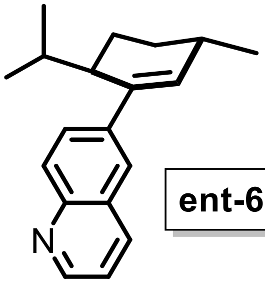

**ent-6e**

Produced from substrates **ent-3** and **5e**. White crystals. Yield: 95%. $^1$H NMR (500 MHz, $CDCl_3$): δ (ppm) 8.86 (d, 1H, *J* = 4.3 Hz), 8.16 (d, 1H, *J* = 8.4 Hz), 8.08 (d, 1H, *J* = 8.4 Hz), 7.65-7.71 (m, 2H, 2 peaks overlapping), 7.40 (dd, 1H, $J_1$ = 8.4 Hz, $J_2$ = 4.3 Hz), 5.83 (bs, 1H), 2.82 (m, 1H), 2.28 (m, 1H), 1.80-1.94 (m, 3H, 3 signals overlapping), 1.51 (m, 1H), 1.21 (m, 1H), 1.04 (d, 3H, *J* = 7.1 Hz), 0.89 (d, 3H, *J* = 6.9 Hz), 0.59 (d, 3H, *J* = 6.9 Hz). $^{13}$C NMR (125 MHz, $CDCl_3$): δ (ppm) 149.0, 146.6, 142.2, 141.0, 136.7, 136.5, 129.7, 128.4, 128.3, 124.4, 121.1, 41.6, 31.5, 30.9, 28.6, 22.0, 21.6, 20.7, 15.7. FT-IR: ν ($cm^{-1}$) 2956 (s), 2925 (s), 2867 (m), 2832 (w), 1656 (w), 1629 (w), 1588 (w), 1494 (m), 1460 (m), 1451 (w), 1386 (m), 1366 (m), 1322 (w), 1181 (m), 883 (w) 875 (m), 840 (s), 794 (m), 777 (m). $[a]^{23}_D$ -3.0 (*c* 0.7, $CH_2Cl_2$).

• **3-((3*S*,6*R*)-6-Isopropyl-3-methylcyclohex-1-en-1-yl)benzofuran (ent-6f):**

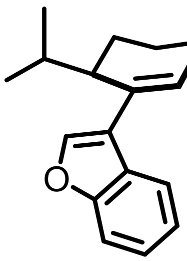

**ent-6f**

Produced from substrates **ent-3** and **5f**. Yellow oil. Yield: 86%. $^{1}$H NMR (500 MHz, $CDCl_3$): δ (ppm) 7.66 (d, 1H, *J* = 7.7 Hz), 7.48 (s, 1H), 7.47 (d, 1H, *J* = 7.5 Hz), 7.28 (t, 1H, *J* = 7.4 Hz), 7.24 (t, 1H, *J* = 7.5 Hz), 5.90 (bs, 1H), 2.66 (m, 1H), 2.29 (m, 1H), 1.80-2.00 (m, 3H, 3 signals overlapping), 1.50 (m, 1H), 1.24 (m, 1H), 1.02 (d, 3H, *J* = 7.1 Hz), 0.88 (d, 3H, *J* = 6.9 Hz), 0.64 (d, 3H, *J* = 6.9 Hz). $^{13}$C NMR (125 MHz, $CDCl_3$): δ (ppm) 155.3, 140.7, 135.4, 132.2, 127.4, 124.0, 122.9, 122.5, 120.7, 111.5, 42.4, 31.0, 30.9, 28.9, 22.0, 21.6, 20.8, 16.1. FT-IR: ν ($cm^{-1}$) 2956 (s), 2923 (s), 2869 (m), 1765 (m), 1614 (m), 1460 (s), 1382 (w), 1329 (w), 1242 (w), 1127 (w), 1068 (m), 1029 (m), 936 (w), 791 (w), 746 (s). $[a]^{23}_{D}$ -6.3 (*c* 0.6, $CH_2Cl_2$).

• **(2*R*,5*S*)-5-Isopropyl-2-methyl-2,3,4,5-tetrahydro-1,1'-biphenyl (7a):**

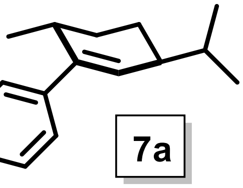

**7a**

Produced from substrates **4** and **5a**. Colourless oil. Yield: 88%. $^{1}$H NMR (500 MHz, $CDCl_3$): δ (ppm) 7.34 (d, 2H, *J* = 7.9 Hz), 7.30 (app. t, 2H, *J* = 7.6 Hz), 7.22 (tt, 1H, $J_1$ = 7.3 Hz, $J_2$ = 1.8 Hz), 5.79 (bs, 1H), 2.84 (m, 1H), 2.12 (m, 1H), 1.84 (m, 1H), 1.68-1.76 (m, 2H, 2 signals overlapping), 1.58 (m, 1H), 1.44 (m, 1H), 0.98 (d, 3H, *J* = 6.9 Hz), 0.94(9) (d, 3H, *J* = 6.9 Hz), 0.94(6) (d, 3H, *J* = 7.1 Hz). $^{13}$C NMR (125 MHz, $CDCl_3$): δ (ppm) 143.2, 129.0, 128.1, 127.9, 126.5, 126.2, 42.7, 32.3, 30.3, 29.9, 20.0, 19.9, 19.8, 19.3. FT-IR: ν ($cm^{-1}$) 2961 (s), 2929 (s), 2873 (m), 1705 (w), 1682 (m), 1599 (w), 1493 (w), 1458 (m), 1448 (m), 1366 (m), 973 (w), 761 (s). $[a]^{23}_{D}$ +4.3 (*c* 0.5, $CH_2Cl_2$).

• **(2*R*,5*S*)-5-Isopropyl-2'-methoxy-2-methyl-2,3,4,5-tetrahydro-1,1'-biphenyl (7b):**

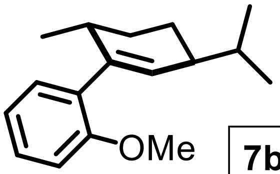

**7b**

Produced from substrates **4** and **5b**. Colorless oil. Yield: 35%. $^{1}$H NMR (500 MHz, $CDCl_3$): δ (ppm) 7.22 (dt, 1H, $J_1$ = 7.8 Hz, $J_2$ = 1.7 Hz), 7.06 (dd, 1H, $J_1$ = 7.4 Hz, $J_2$ = 1.7 Hz), 6.90 (dt, 1H, $J_1$ = 7.4 Hz, $J_2$ = 1.0 Hz), 6.85 (dd, 1H, $J_1$ = 8.2 Hz, $J_2$ = 1.0 Hz), 5.51 (bs, 1H), 3.80 (s, 3H), 2.80 (m, 1H), 2.13 (m, 1H), 1.88 (m, 1H), 1.69 (m, 1H), 1.54-1.62 (m, 2H, 2 signals overlapping), 1.49 (m, 1H), 0.96 (d, 3H, *J* = 6.8 Hz), 0.94 (d, 3H, *J* = 6.8 Hz), 0.84 (d, 3H, *J* = 7.1 Hz). $^{13}$C NMR (125 MHz, $CDCl_3$): δ (ppm) 156.7, 143.1, 133.2, 130.8, 129.7, 127.7, 120.3, 110.5, 55.5, 42.0, 32.3, 31.0, 29.6, 20.6, 19.8, 19.7, 19.4. FT-IR: ν ($cm^{-1}$) 2956 (m), 2920 (m), 2869 (m), 2831 (w), 1726 (w), 1597 (m), 1579 (w), 1488 (m), 1462 (m), 1435 (m), 1378 (w), 1365 (w), 1289 (m), 1244 (s), 1179 (w), 1115 (m), 1052 (m), 1028 (m), 751 (s). $[a]^{23}_{D}$ +5.3 (*c* 0.6, $CH_2Cl_2$).

• **(2*R*,5*S*)-5-Isopropyl-2',6'-dimethoxy-2-methyl-2,3,4,5-tetrahydro-1,1'-biphenyl (7c):**

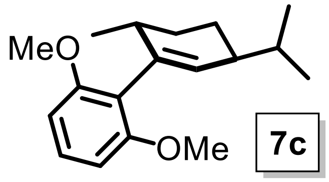

**7c**

Produced from substrates **4** and **5c**. Colorless oil. Yield: 31%. $^{1}$H NMR (500 MHz, $CDCl_3$): δ (ppm) 7.16 (t, 1H, *J* = 8.3 Hz), 6.55 (d, 2H, *J* = 8.3 Hz), 5.37 (bs, 1H), 3.76 (s, 6H), 2.52 (m, 1H), 2.18 (m, 1H), 1.88 (m, 1H), 1.71 (m, 1H), 1.59 (m, 1H), 1.52-1.58 (m, 2H, 2 signals overlapping), 0.95 (d, 3H, *J* = 6.9 Hz), 0.94 (d, 3H, *J* = 6.9 Hz), 0.84 (d, 3H, *J* = 6.9 Hz). $^{13}$C NMR (125 MHz, $CDCl_3$): δ (ppm) 158.1, 136.2, 130.5, 127.4, 121.8, 104.0, 55.8, 42.0, 32.3, 31.8, 29.7, 20.2, 19.5, 19.3, 19.1. FT-IR: ν ($cm^{-1}$) 2958 (m), 2932 (m), 2870 (w), 2834 (w), 1709 (w), 1585 (m), 1468 (s), 1432 (m), 1245 (s), 1107 (s), 778 (w), 726 (m). $[a]^{23}_{D}$ -14.3 (*c* 0.3, $CH_2Cl_2$).

• **4-((3*S*,6*R*)-3-Isopropyl-6-methylcyclohex-1-en-1-yl)pyridine (7d):**

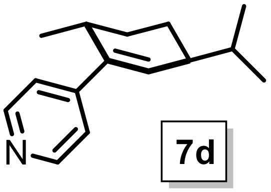

**7d**

Produced from substrates **4** and **5d**. Yellow oil. Yield: 99%. $^{1}$H NMR (500 MHz, $CDCl_3$): δ (ppm) 8.51 (d, 2H, *J* = 5.5 Hz), 7.27 (d, 2H, *J* = 5.5 Hz), 6.05 (bs, 1H), 2.82 (m, 1H), 2.14 (m, 1H), 1.81 (ddt, 1H, $J_1$ = 12.9 Hz, $J_2$ = 5.5 Hz, $J_3$ = 3.2 Hz), 1.69-1.79 (m, 2H, 2 signals overlapping), 1.60 (m, 1H), 1.45 (m, 1H), 0.98 (d, 3H, *J* = 6.7 Hz), 0.96 (d, 3H, *J* = 6.7 Hz), 0.94 (d, 3H, *J* = 6.5 Hz). $^{13}$C NMR (125 MHz, $CDCl_3$): δ (ppm) 150.1, 149.2, 140.8, 132.8, 120.9, 42.8, 32.2, 29.6, 29.3, 19.7(9), 19.7(7), 19.7(0), 19.2. FT-IR: ν ($cm^{-1}$) 2960 (m), 2930 (m), 2872 (m), 1601 (m), 1458 (m), 1418 (w), 1382 (w), 1364 (w), 1220 (w), 1197 (w), 1069 (w), 1028 (w), 1006 (w), 852 (w), 827 (m). $[a]^{23}_{D}$ +8.3 (*c* 0.7, $CH_2Cl_2$).

• **6-((3*S*,6*R*)-3-Isopropyl-6-methylcyclohex-1-en-1-yl)quinoline (7e):**

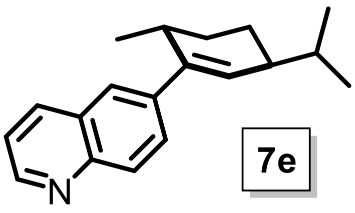

**7e**

Produced from substrates **4** and **5e**. Colorless oil. Yield: 92%. $^{1}$H NMR (500 MHz, $CDCl_3$): δ (ppm) 8.86 (dd, 1H, $J_1$ = 4.3 Hz, $J_2$ = 1.5 Hz), 8.19 (d, 1H, *J* = 8.2 Hz), 8.10 (d, 1H, *J* = 8.8 Hz), 7.79 (dd, 1H, $J_1$ = 8.8 Hz, $J_2$ = 2.0 Hz), 7.73 (d, 1H, *J* = 2.0 Hz), 7.42 (dd, 1H, $J_1$ = 8.2 Hz, $J_2$= 4.3 Hz), 5.98 (bs, 1H), 2.97 (m, 1H), 2.18 (m, 1H), 1.88 (ddt, 1H, $J_1$ = 13.0 Hz, $J_2$ = 5.5 Hz, $J_3$ = 3.1 Hz), 1.73-1.83 (m, 2H, 2 signals overlapping), 1.63 (m, 1H), 1.48 (m, 1H), 1.02 (d, 3H, *J* = 6.9 Hz), 0.99 (d, 6H, *J* = 6.9 Hz). $^{13}$C NMR (125 MHz, $CDCl_3$): δ (ppm) 149.0, 142.4, 141.1, 136.9, 131.1, 129.3, 128.4, 128.3, 126.5, 124.0, 121.1, 42.9, 32.3, 30.2, 29.9, 20.0, 19.9, 19.8, 19.3. FT-IR: ν ($cm^{-1}$) 2962 (m), 2871 (m), 1706 (s), 1677 (s), 1654 (m), 1620 (m), 1591 (w), 1577 (w), 1560 (w), 1498 (m), 1460 (s), 1368 (w), 1322 (w), 1167 (w), 1116 (w), 982 (w), 898 (w), 839 (m), 800 (w), 771 (w). $[a]^{23}_{D}$ +16.7 (*c* 0.6, $CH_2Cl_2$).

• **3-((3*S*,6*R*)-3-Isopropyl-6-methylcyclohex-1-en-1-yl)benzofuran (7f):**

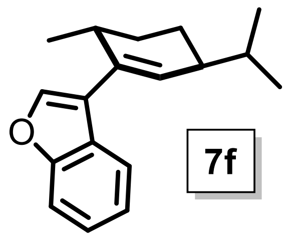

**7f**

Produced from substrates **4** and **5f**. Colorless oil. Yield: 67%. $^1$H NMR (500 MHz, $CDCl_3$): δ (ppm) 7.75 (dd, 1H, $J_1$ = 7.6 Hz, $J_2$ = 1.3 Hz), 7.57 (s, 1H), 7.48 (d, 1H, $J$ = 8.0 Hz), 7.30 (dt, 1H, $J_1$ = 7.4 Hz, $J_2$ = 1.3 Hz), 7.26 (dt, 1H, $J_1$ = 7.6 Hz, $J_2$ = 1.3 Hz), 6.08 (bs, 1H), 2.68 (m, 1H), 2.19 (m, 1H), 1.88 (ddd, 1H, $J_1$ = 13.0 Hz, $J_2$ = 5.4 Hz, $J_3$ = 3.2 Hz), 1.77 (m, 1H), 1.71 (m, 1H), 1.63 (m, 1H), 1.52 (m, 1H), 1.09 (d, 3H, $J$ = 6.8 Hz) 1.03 (d, 3H, $J$ = 6.8 Hz), 1.00 (d, 3H, $J$ = 7.1 Hz). $^{13}$C NMR (125 MHz, $CDCl_3$): δ (ppm) 155.7, 140.9, 134.3, 129.1, 126.9, 124.1, 122.5, 122.2, 121.2, 111.6, 42.8, 32.3, 31.4, 29.7, 20.2, 19.9, 19.8, 19.4. FT-IR: ν ($cm^{-1}$) 2956 (m), 2928 (m), 2869 (m), 1814 (w), 1613 (w), 1475 (w), 1461 (s), 1451 (s), 1384 (w), 1366 (w), 1067 (w), 744 (s). $[a]^{23}_D$ +8.5 (*c* 1.5, $CH_2Cl_2$).

• **(2*S*,5*R*)-5-Isopropyl-2-methyl-2,3,4,5-tetrahydro-1,1'-biphenyl (ent-7a):**

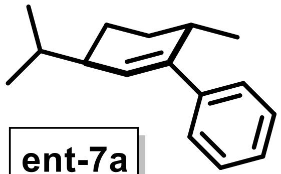

**ent-7a**

Produced from substrates **ent-4** and **5a**. Colourless oil. Yield: 78%. $^1$H NMR (500 MHz, $CDCl_3$): δ (ppm) 7.34 (d, 2H, $J$ = 7.9 Hz), 7.30 (app. t, 2H, $J$ = 7.6 Hz), 7.22 (tt, 1H, $J_1$ = 7.3 Hz, $J_2$ = 1.8 Hz), 5.79 (bs, 1H), 2.84 (m, 1H), 2.12 (m, 1H), 1.84 (m, 1H), 1.68-1.76 (m, 2H, 2 signals overlapping), 1.58 (m, 1H), 1.44 (m, 1H), 0.98 (d, 3H, $J$ = 6.9 Hz), 0.94(9) (d, 3H, $J$ = 6.9 Hz), 0.94(5) (d, 3H, $J$ = 7.1 Hz). $^{13}$C NMR (125 MHz, $CDCl_3$): δ (ppm) 143.2, 129.0, 128.1, 127.9, 126.5, 126.2, 42.7, 32.3, 30.3, 29.9, 20.0, 19.9, 19.8, 19.3. FT-IR: ν ($cm^{-1}$) 2965 (m), 2933 (m), 2875 (w), 1709 (s), 1680 (s), 1649 (w), 1597 (w), 1578 (w), 1459 (m), 1448 (m), 1382 (w), 1368 (w), 973 (w), 762 (m). $[a]^{23}_D$ -4.8 (*c* 0.6, $CH_2Cl_2$).

• **(2*S*,5*R*)-5-Isopropyl-2'-methoxy-2-methyl-2,3,4,5-tetrahydro-1,1'-biphenyl (ent-7b):**

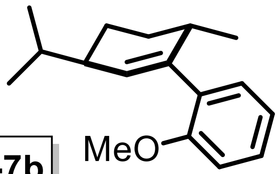


**ent-7b**

Produced from substrates **ent-4** and **5b**. Colorless oil. Yield: 35%. $^1$H NMR (500 MHz, $CDCl_3$): δ (ppm) 7.22 (dt, 1H, $J_1$ = 7.8 Hz, $J_2$ = 1.7 Hz), 7.06 (dd, 1H, $J_1$ = 7.4 Hz, $J_2$ = 1.7 Hz), 6.90 (dt, 1H, $J_1$ = 7.4 Hz, $J_2$ = 1.0 Hz), 6.85 (dd, 1H, $J_1$ = 8.2 Hz, $J_2$ = 1.0 Hz), 5.51 (bs, 1H), 3.80 (s, 3H), 2.80 (m, 1H), 2.13 (m, 1H), 1.88 (m, 1H), 1.69 (m, 1H), 1.54-1.62 (m, 2H, 2 signals overlapping), 1.49 (m, 1H), 0.96 (d, 3H, $J$ = 6.8 Hz), 0.94 (d, 3H, $J$ = 6.8 Hz), 0.84 (d, 3H, $J$ = 7.1 Hz). $^{13}$C NMR (125 MHz, $CDCl_3$): δ (ppm) 156.7, 143.1, 133.2, 130.8, 129.7, 127.7, 120.3, 110.5, 55.5, 42.0, 32.3, 31.0, 29.6, 20.6, 19.8, 19.7, 19.4. FT-IR: ν ($cm^{-1}$) 2959 (m), 2918 (m), 2872 (m), 2829 (m), 1729 (w), 1597 (m), 1580 (w), 1488 (m), 1464 (m), 1436 (m), 1378 (w), 1366 (w), 1286 (m), 1243 (m), 1179 (m), 1115 (w), 1052 (m), 1027 (m), 753 (s). $[a]^{23}_D$ -5.8 (*c* 0.2, $CH_2Cl_2$).

• **(2*S*,5*R*)-5-Isopropyl-2',6'-dimethoxy-2-methyl-2,3,4,5-tetrahydro-1,1'-biphenyl (ent-7c):**

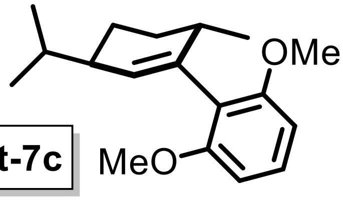


**ent-7c**

Produced from substrates **ent-4** and **5c**. Colorless oil. Yield: 35%. $^{1}$H NMR (500 MHz, $CDCl_3$): δ (ppm) 7.16 (t, 1H, *J* = 8.3 Hz), 6.55 (d, 2H, *J* = 8.3 Hz), 5.37 (bs, 1H), 3.76 (s, 6H), 2.52 (m, 1H), 2.18 (m, 1H), 1.88 (m, 1H), 1.71 (m, 1H), 1.59 (m, 1H), 1.52-1.58 (m, 2H, 2 signals overlapping), 0.95 (d, 3H, *J* = 6.9 Hz), 0.94 (d, 3H, *J* = 6.9 Hz), 0.84 (d, 3H, *J* = 6.9 Hz). $^{13}$C NMR (125 MHz, $CDCl_3$): δ (ppm) 158.1, 136.2, 130.5, 127.4, 121.8, 104.0, 55.8, 42.0, 32.3, 31.8, 29.7, 20.2, 19.5, 19.3, 19.1. FT-IR: ν ($cm^{-1}$) 2956 (m), 2931 (m), 2869 (w), 2834 (w), 1708 (w), 1586 (m), 1468 (s), 1432 (m), 1244 (s), 1107 (s), 778 (w), 725 (m). $[a]^{23}_{D}$ +13.8 (*c* 0.3, $CH_2Cl_2$).

• **4-((3*R*,6*S*)-3-Isopropyl-6-methylcyclohex-1-en-1-yl)pyridine (ent-7d):**

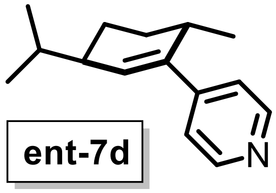


**ent-7d**

Produced from substrates **ent-4** and **5d**. Yellow oil. Yield: 90%. $^{1}$H NMR (500 MHz, $CDCl_3$): δ (ppm) 8.51 (d, 2H, *J* = 5.5 Hz), 7.27 (d, 2H, *J* = 5.5 Hz), 6.05 (bs, 1H), 2.82 (m, 1H), 2.14 (m, 1H), 1.81 (ddt, 1H, $J_1$ = 12.9 Hz, $J_2$ = 5.5 Hz, $J_3$ = 3.2 Hz), 1.69-1.79 (m, 2H, 2 signals overlapping), 1.60 (m, 1H), 1.45 (m, 1H), 0.98 (d, 3H, *J* = 6.7 Hz), 0.96 (d, 3H, *J* = 6.7 Hz), 0.94 (d, 3H, *J* = 6.5 Hz). $^{13}$C NMR (125 MHz, $CDCl_3$): δ (ppm) 150.1, 149.2, 140.8, 132.8, 120.9, 42.8, 32.2, 29.6, 29.3, 19.7(9), 19.7(7), 19.7(0), 19.2. FT-IR: ν ($cm^{-1}$) 2959 (m), 2932 (m), 2872 (m), 1600 (m), 1458 (m), 1418 (w), 1382 (w), 1363 (w), 1220 (w), 1197 (w), 1068 (w), 1028 (w), 1006 (w), 850 (w), 827 (m). $[a]^{23}_{D}$ -9.0 (*c* 0.4, $CH_2Cl_2$).

• **6-((3*R*,6*S*)-3-Isopropyl-6-methylcyclohex-1-en-1-yl)quinoline (ent-7e):**

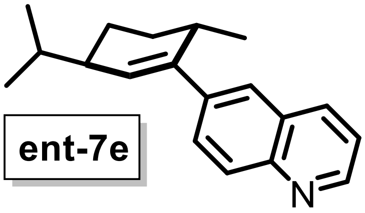


**ent-7e**

Produced from substrates **ent-4** and **5e**. Colorless oil. Yield: 96%. $^{1}$H NMR (500 MHz, $CDCl_3$): δ (ppm) 8.86 (dd, 1H, $J_1$ = 4.3 Hz, $J_2$ = 1.5 Hz), 8.19 (d, 1H, *J* = 8.2 Hz), 8.10 (d, 1H, *J* = 8.8 Hz), 7.79 (dd, 1H, $J_1$ = 8.8 Hz, $J_2$ = 2.0 Hz), 7.73 (d, 1H, *J* = 2.0 Hz), 7.42 (dd, 1H, $J_1$ = 8.2 Hz, $J_2$= 4.3 Hz), 5.98 (bs, 1H), 2.97 (m, 1H), 2.18 (m, 1H), 1.88 (ddt, 1H, $J_1$ = 13.0 Hz, $J_2$ = 5.5 Hz, $J_3$ = 3.1 Hz), 1.73-1.83 (m, 2H, 2 signals overlapping), 1.63 (m, 1H), 1.48 (m, 1H), 1.02 (d, 3H, *J* = 6.9 Hz), 0.99 (d, 6H, *J* = 6.9 Hz). $^{13}$C NMR (125 MHz, $CDCl_3$): δ (ppm) 149.0, 142.4, 141.1, 136.9, 131.1, 129.3, 128.4, 128.3, 126.5, 124.0, 121.1, 42.9, 32.3, 30.2, 29.9, 20.0, 19.9, 19.8, 19.3. FT-IR: ν ($cm^{-1}$) 2963 (m), 2872 (m), 1706 (s), 1676 (s), 1654 (m), 1619 (m), 1591 (w), 1577 (w), 1560 (w), 1498 (m), 1459 (s), 1368 (w), 1320 (w), 1167 (w), 1118 (w), 982 (w), 897 (w), 839 (m), 800 (w), 772 (w). $[a]^{23}_{D}$ - 17.4 (*c* 0.4, $CH_2Cl_2$).

**• 3-((3*R*,6*S*)-3-Isopropyl-6-methylcyclohex-1-en-1-yl)benzofuran (ent-7f):**

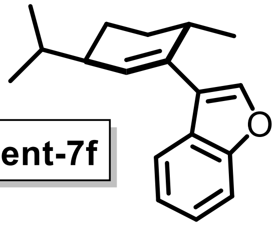

**ent-7f**

Produced from substrates **ent-4** and **5f**. Colorless oil. Yield: 82%. $^{1}$H NMR (500 MHz, $CDCl_3$): δ (ppm) 7.75 (dd, 1H, $J_1$ = 7.6 Hz, $J_2$ = 1.3 Hz), 7.57 (s, 1H), 7.48 (d, 1H, $J$ = 8.0 Hz), 7.30 (dt, 1H, $J_1$ = 7.4 Hz, $J_2$ = 1.3 Hz), 7.26 (dt, 1H, $J_1$ = 7.6 Hz, $J_2$ = 1.3 Hz), 6.08 (bs, 1H), 2.68 (m, 1H), 2.19 (m, 1H), 1.88 (ddd, 1H, $J_1$ = 13.0 Hz, $J_2$ = 5.4 Hz, $J_3$ = 3.2 Hz), 1.77 (m, 1H), 1.71 (m, 1H), 1.63 (m, 1H), 1.52 (m, 1H), 1.09 (d, 3H, $J$ = 6.8 Hz) 1.03 (d, 3H, $J$ = 6.8 Hz), 1.00 (d, 3H, $J$ = 7.1 Hz). $^{13}$C NMR (125 MHz, $CDCl_3$): δ (ppm) 155.7, 140.9, 134.3, 129.1, 126.9, 124.1, 122.5, 122.2, 121.2, 111.6, 42.8, 32.3, 31.4, 29.7, 20.2, 19.9, 19.8, 19.4. FT-IR: v ($cm^{-1}$) 2958 (m), 2929 (m), 2870 (m), 1814 (m), 1610 (w), 1475 (m), 1461 (s), 1452 (s), 1385 (w), 1368 (w), 1230 (w), 1137 (w), 1065 (w), 747 (s). $[a]^{23}_{D}$ -9.4 (*c* 0.5, $CH_2Cl_2$).

**• ((1*S*,2*S*,5*R*)-2-Isopropyl-5-methylcyclohexyl)benzene (8a):**

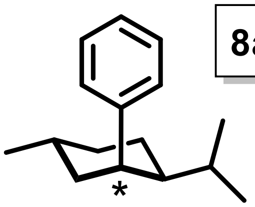

**8a**

Produced from substrate **6a**. Colorless oil. Yield: 93%. $^{1}$H NMR (500 MHz, $CDCl_3$): δ (ppm) 7.36 (d, 2H, $J$ = 7.7 Hz), 7.26 (app. t, 2H, $J$ = 7.5 Hz), 7.18 (t, 1H, $J$ = 7.3 Hz), 3.18 (bs, 1H), 1.84-1.94 (m, 2H, 2 signals overlapping), 1.60-1.76 (m, 3H, 3 signals overlapping), 1.27-1.43 (m, 3H, 3 signals overlapping), 1.01 (ddd, 1H, $J_1$ = 26.0 Hz, $J_2$ = 12.0 Hz, $J_3$ = 4.5 Hz), 0.83 (d, 3H, $J$ = 6.3 Hz), 0.81 (d, 3H, $J$ = 6.3 Hz), 0.77 (d, 3H, $J$ = 6.3 Hz). $^{13}$C NMR (125 MHz, $CDCl_3$): δ (ppm) 145.7, 130.1, 127.8, 125.4, 47.2, 43.1, 43.0, 35.4, 30.1, 26.6, 25.8, 22.8, 21.3, 21.2. FT-IR: v ($cm^{-1}$) 2922 (s), 2853 (s), 2358 (w), 1742 (w), 1458 (m), 1378 (w), 767 (w). $[a]^{23}_{D}$ +5.9 (*c* 0.8, $CH_2Cl_2$).

**• 1-((1*S*,2*S*,5*R*)-2-Isopropyl-5-methylcyclohexyl)-2-methoxybenzene (8b):**

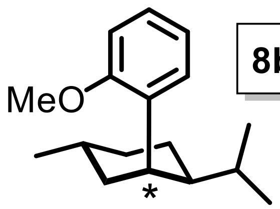


**8b**

Produced from substrate **6b**. Yellow oil. Yield: 84%. $^{1}$H NMR (500 MHz, $CDCl_3$): δ (ppm) 7.49 (dd, 1H, $J_1$ = 7.6 Hz, $J_2$ = 1.5 Hz), 7.16 (dt, 1H, $J_1$ = 7.7 Hz, $J_2$ = 1.5 Hz), 6.88 (d, 1H, $J$ = 8.8 Hz), 6.85 (t, 1H, $J$ = 7.2 Hz), 3.82 (s, 3H), 3.73 (bs, 1H), 1.80-1.96 (m, 2H, 2 signals overlapping), 1.67 (m, 2H, 2 signals overlapping), 1.54 (m, 1H), 1.44 (m, 1H), 1.28 (m, 2H, 2 signals overlapping), 1.00 (m, 1H), 0.87 (d, 3H, $J$ = 6.6 Hz), 0.79 (d, 3H, $J$ = 6.6 Hz), 0.68 (d, 3H, $J$ = 6.6 Hz). $^{13}$C NMR (125 MHz, $CDCl_3$): δ (ppm) 157.3, 133.7, 130.2, 126.1, 119.2, 110.6, 55.5, 46.8, 41.2, 35.4, 32.3, 29.9, 27.4, 25.9, 22.7, 21.4, 21.2. FT-IR: v ($cm^{-1}$) 2945 (s), 2923 (s), 2867 (m), 1600 (w), 1490 (m), 1453 (m), 1438 (w), 1259 (m), 1236 (s), 1107 (m), 1032 (w), 799 (m), 747 (s). $[a]^{23}_{D}$ +14.4 (*c* 0.6, $CH_2Cl_2$).

**• 2-((1*S*,2*S*,5*R*)-2-Isopropyl-5-methylcyclohexyl)-1,3-dimethoxybenzene (8c):**

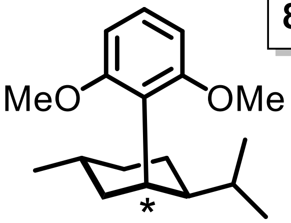

**8c**

Produced from substrate **6c**. Colorless oil. Yield: 81%. $^1$H NMR (500 MHz, $CDCl_3$): δ (ppm) 7.10 (t, 1H, $J$ = 8.2 Hz), 6.53 (app. t, 2H, 2 signals overlapping, $J$ = 8.6 Hz), 3.79 (s, 3H), 3.78 (s, 3H), 3.21 (dt, 1H, $J_1$ = 11.5 Hz, $J_2$ = 3.4 Hz), 2.11 (tt, 1H, $J_1$ = 11.4 Hz, $J_2$ = 2.8 Hz), 1.77 (m, 1H), 1.63-1.71 (m, 2H, 2 signals overlapping), 1.54 (m, 1H), 1.49 (m, 1H), 1.34 (m, 1H), 1.05 (m, 2H, 2 signals overlapping), 0.89 (d, 3H, $J$ = 6.4 Hz), 0.80 (d, 3H, $J$ = 7.0 Hz), 0.64 (d, 3H, $J$ = 7.0 Hz). $^{13}$C NMR (125 MHz, $CDCl_3$): δ (ppm) 159.7, 158.2, 126.4, 122.2, 104.7, 104.2, 56.0, 55.3, 43.9, 39.9, 38.0, 35.6, 33.6, 28.7, 25.4, 22.6, 21.7, 15.7. FT-IR: ν (cm$^{-1}$) 2949 (s), 2921 (s), 2853 (s), 2838 (m), 1590 (s), 1470 (s), 1433 (m), 1249 (m), 1220 (w), 1140 (w), 1105 (s), 1093 (s), 780 (w), 724 (m). $[a]^{23}_D$ +3.7 (*c* 1.3, $CH_2Cl_2$).

**• 4-((1*S*,2*S*,5*R*)-2-Isopropyl-5-methylcyclohexyl)pyridine (8d):**

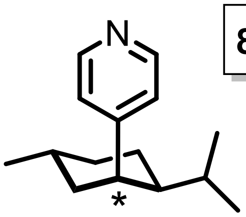

**8d**

Produced from substrate **6d**. Colorless oil. Yield: 38%. $^1$H NMR (500 MHz, $CDCl_3$): δ (ppm) 8.47 (d, 2H, $J$ = 5.3 Hz), 7.32 (d, 2H, $J$ = 5.3 Hz), 3.18 (bs, 1H), 1.86-1.96 (m, 2H, 2 signals overlapping), 1.69 (ddd, 1H, $J_1$ = 13.1 Hz, $J_2$ = 6.4 Hz, $J_3$ = 2.5 Hz), 1.57 (m, 2H, 2 signals overlapping), 1.42 (dt, 1H, $J_1$ = 12.8 Hz, $J_2$ = 5.6 Hz), 1.31 (m, 2H, 2 signals overlapping), 1.01 (ddd, 1H, $J_1$ = 25.4 Hz, $J_2$ = 13.0 Hz, $J_3$ = 4.8 Hz), 0.86 (d, 3H, $J$ = 6.1 Hz), 0.81 (d, 3H, $J$ = 6.3 Hz), 0.74 (d, 3H, $J$ = 6.1 Hz). $^{13}$C NMR (125 MHz, $CDCl_3$): δ (ppm) 155.9, 148.2, 125.8, 46.8, 42.8, 42.3, 35.0, 30.0, 26.7, 25.9, 22.6, 21.2, 21.1. FT-IR: ν (cm$^{-1}$) 2953 (s), 2923 (s), 2869 (s), 2852 (s), 1719 (m), 1639 (w), 1585 (m), 1459 (m), 1414 (w), 1377 (w), 1366 (w), 820 (m). $[a]^{23}_D$ +20.2 (*c* 0.5, $CH_2Cl_2$).

**• 6-((1*S*,2*S*,5*R*)-2-Isopropyl-5-methylcyclohexyl)quinoline (8e):**

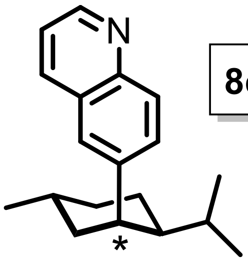

**8e**

Produced from substrate **6e**. Yellow oil. Yield: 60%. $^1$H NMR ($CDCl_3$): δ (500 MHz, ppm) 8.85 (dd, 1H, $J_1$ = 4.2 Hz, $J_2$ = 1.4 Hz), 8.14 (d, 1H, $J$ = 8.0 Hz), 8.04 (d, 1H, $J$ = 8.7 Hz), 7.80 (d, 1H, $J$ = 2.0 Hz), 7.74 (dd, 1H, $J_1$ = 8.7 Hz, $J_2$ = 2.0 Hz), 7.39 (dd, 1H, $J_1$ = 8.2 Hz, $J_2$ = 4.2 Hz), 3.38 (bs, 1H), 1.90-2.00 (m, 2H, 2 signals overlapping), 1.72-1.84 (m, 2H, 2 signals overlapping), 1.69 (m, 1H), 1.32-1.52 (m, 3H, 3 signals overlapping), 1.06 (m, 1H), 0.87 (d, 3H, $J$ = 6.3 Hz), 0.81 (d, 3H, $J$ = 6.3 Hz), 0.77 (d, 3H, $J$ = 6.3 Hz). $^{13}$C NMR (125 MHz, $CDCl_3$): δ (ppm) 149.2, 146.3, 144.4, 136.3, 133.2, 128.3, 128.0, 127.6, 120.9, 47.2, 43.0, 42.9, 35.3, 30.1, 26.8,

25.9, 22.7, 21.3, 21.2. FT-IR: v ($cm^{-1}$) 2924 (s), 2866 (m), 2839 (m), 2089 (w), 1613 (w), 1508 (s), 1454 (m), 836 (s), 799 (m), 775 (m). $[a]^{23}_{D}$ +2.2 (*c* 2.0, $CH_2Cl_2$).

**• 3-((1*S*,2*S*,5*R*)-2-Isopropyl-5-methylcyclohexyl)benzofuran (8f):**

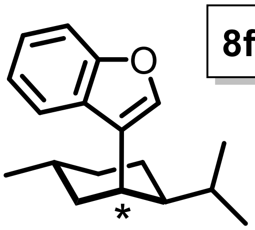

**8f**

Produced from substrate **6f**. Colorless oil. Yield: 50%. $^{1}$H NMR (500 MHz, $CDCl_3$): δ (ppm) 7.56 (dd, 1H, $J_1$ = 7.8 Hz, $J_2$ = 1.1 Hz), 7.54 (bs, 1H), 7.47 (d, 1H, *J* = 8.0 Hz), 7.29 (dt, 1H, $J_1$ = 7.4 Hz, $J_2$ = 1.4 Hz), 7.24 (dt, 1H, $J_1$ = 7.5 Hz, $J_2$ = 1.1 Hz), 3.51 (bs, 1H), 1.96 (m, 1H), 1.77-1.85 (m, 2H, 2 signals overlapping), 1.42-1.52 (m, 2H, 2 signals overlapping), 1.23-1.39 (m, 3H, 3 signals overlapping), 1.00 (ddd, $J_1$ = 26.6 Hz, $J_2$ = 12.5 Hz, $J_3$ = 4.0 Hz), 0.89 (d, 3H, *J* = 6.5 Hz), 0.80 (d, 3H, *J* = 6.5 Hz), 0.74 (d, 3H, *J* = 6.5 Hz). $^{13}$C NMR (125 MHz, $CDCl_3$): δ (ppm) 154.3, 141.4, 129.1, 124.0, 122.1, 120.9, 119.4, 111.2, 47.3, 40.5, 35.3, 32.5, 30.2, 27.2, 26.8, 22.3, 21.3, 21.2. FT-IR: v ($cm^{-1}$) 2922 (s), 2867 (m), 2843 (m), 1454 (s), 1272 (w), 1179 (w), 1098 (w), 1085 (m), 859 (w), 743 (s). $[a]^{23}_{D}$ +10.2 (*c* 0.1, $CH_2Cl_2$).

**• ((1*R*,2*R*,5*S*)-2-Isopropyl-5-methylcyclohexyl)benzene (ent-8a):**

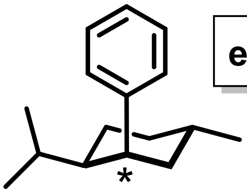

**ent-8a**

Produced from substrate **ent-6a**. Colorless oil. Yield: 99%. $^{1}$H NMR (500 MHz, $CDCl_3$): δ (ppm) 7.36 (d, 2H, *J* = 7.7 Hz), 7.26 (app. t, 2H, *J* = 7.5 Hz), 7.18 (t, 1H, *J* = 7.3 Hz), 3.18 (bs, 1H), 1.84-1.94 (m, 2H, 2 signals overlapping), 1.60-1.76 (m, 3H, 3 signals overlapping), 1.27-1.43 (m, 3H, 3 signals overlapping), 1.01 (ddd, 1H, $J_1$ = 26.0 Hz, $J_2$ = 12.0 Hz, $J_3$ = 4.5 Hz), 0.83 (d, 3H, *J* = 6.3 Hz), 0.81 (d, 3H, *J* = 6.3 Hz), 0.77 (d, 3H, *J* = 6.3 Hz). $^{13}$C NMR (125 MHz, $CDCl_3$): δ (ppm) 145.7, 130.1, 127.8, 125.4, 47.2, 43.1, 43.0, 35.4, 30.1, 26.6, 25.8, 22.8, 21.3, 21.2. FT-IR: v ($cm^{-1}$) 2922 (s), 2853 (s), 2358 (w), 1742 (w), 1458 (m), 1378 (w), 767 (w). $[a]^{23}_{D}$ -5.4 (*c* 1.4, $CH_2Cl_2$).

**• 1-((1*R*,2*R*,5*S*)-2-Isopropyl-5-methylcyclohexyl)-2-methoxybenzene (ent-8b):**

**ent-8b**

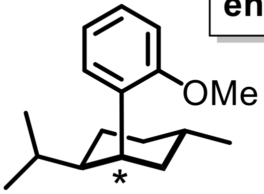


Produced from substrate **ent-6b**. Colorless oil. Yield: 81%. $^{1}$H NMR (500 MHz, $CDCl_3$): δ (ppm) 7.49 (dd, 1H, $J_1$ = 7.6 Hz, $J_2$ = 1.5 Hz), 7.16 (dt, 1H, $J_1$ = 7.7 Hz, $J_2$ = 1.5 Hz), 6.88 (d, 1H, *J* = 8.8 Hz), 6.85 (t, 1H, *J* = 7.2 Hz), 3.82 (s, 3H), 3.73 (bs, 1H), 1.80-1.96 (m, 2H, 2 signals overlapping), 1.67 (m, 2H, 2 signals overlapping), 1.54 (m, 1H), 1.44 (m, 1H), 1.28 (m, 2H, 2 signals overlapping), 1.00 (m, 1H), 0.87 (d, 3H, *J* = 6.6 Hz), 0.79 (d, 3H, *J* = 6.6 Hz), 0.68

(d, 3H, *J* = 6.6 Hz). $^{13}$C NMR (125 MHz, CDCl$_3$): δ (ppm) 157.3, 133.7, 130.2, 126.1, 119.2, 110.6, 55.5, 46.8, 41.2, 35.4, 32.3, 29.9, 27.4, 25.9, 22.7, 21.4, 21.2. FT-IR: ν (cm$^{-1}$) 2946 (s), 2923 (s), 2866 (m), 1600 (w), 1491 (m), 1450 (m), 1438 (m), 1258 (m), 1235 (s), 1107 (m), 1034 (m), 800 (m), 748 (s). [a]$^{23}_{D}$ -14.9 (*c* 0.3, CH$_2$Cl$_2$).

**• 2-((1*R*,2*R*,5*S*)-2-Isopropyl-5-methylcyclohexyl)-1,3-dimethoxybenzene (ent-8c):**

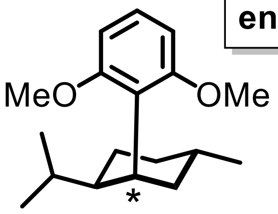


**ent-8c**

Produced from substrate **ent-6c**. White solid. Yield: 76%. $^{1}$H NMR (500 MHz, CDCl$_3$): δ (ppm) 7.10 (t, 1H, *J* = 8.2 Hz), 6.53 (app. t, 2H, 2 signals overlapping, *J* = 8.6 Hz), 3.79 (s, 3H), 3.78 (s, 3H), 3.21 (dt, 1H, $J_1$ = 11.5 Hz, $J_2$ = 3.4 Hz), 2.11 (tt, 1H, $J_1$ = 11.4 Hz, $J_2$ = 2.8 Hz), 1.77 (m, 1H), 1.63-1.71 (m, 2H, 2 signals overlapping), 1.54 (m, 1H), 1.49 (m, 1H), 1.34 (m, 1H), 1.05 (m, 2H, 2 signals overlapping), 0.89 (d, 3H, *J* = 6.4 Hz), 0.80 (d, 3H, *J* = 7.0 Hz), 0.64 (d, 3H, *J* = 7.0 Hz). $^{13}$C NMR (125 MHz, CDCl$_3$): δ (ppm) 159.7, 158.2, 126.4, 122.2, 104.7, 104.2, 56.0, 55.3, 43.9, 39.9, 38.0, 35.6, 33.6, 28.7, 25.4, 22.6, 21.7, 15.7. FT-IR: ν (cm$^{-1}$) 2949 (m), 2923 (m), 2857 (s), 2836 (m), 1590 (s), 1470 (s), 1435 (m), 1249 (m), 1220 (m), 1140 (m), 1104 (s), 1094 (s), 780 (m), 725 (s). [a]$^{23}_{D}$ -3.4 (*c* 1.1, CH$_2$Cl$_2$).

**• 4-((1*R*,2*R*,5*S*)-2-Isopropyl-5-methylcyclohexyl)pyridine (ent-8d):**

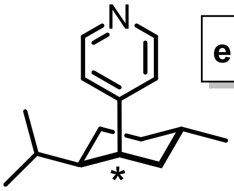


**ent-8d**

Produced from substrate **ent-6d**. Yellow oil. Yield: 33%. $^{1}$H NMR (500 MHz, CDCl$_3$): δ (ppm) 8.47 (d, 2H, *J* = 5.3 Hz), 7.32 (d, 2H, *J* = 5.3 Hz), 3.18 (bs, 1H), 1.86-1.96 (m, 2H, 2 signals overlapping), 1.69 (ddd, 1H, $J_1$ = 13.1 Hz, $J_2$ = 6.4 Hz, $J_3$ = 2.5 Hz), 1.57 (m, 2H, 2 signals overlapping), 1.42 (dt, 1H, $J_1$ = 12.8 Hz, $J_2$ = 5.6 Hz), 1.31 (m, 2H, 2 signals overlapping), 1.01 (ddd, 1H, $J_1$ = 25.4 Hz, $J_2$ = 13.0 Hz, $J_3$ = 4.8 Hz), 0.86 (d, 3H, *J* = 6.1 Hz), 0.81 (d, 3H, *J* = 6.3 Hz), 0.74 (d, 3H, *J* = 6.1 Hz). $^{13}$C NMR (125 MHz, CDCl$_3$): δ (ppm) 155.9, 148.2, 125.8, 46.8, 42.8, 42.3, 35.0, 30.0, 26.7, 25.9, 22.6, 21.2, 21.1. FT-IR: ν (cm$^{-1}$) 2953 (s), 2924 (s), 2869 (m), 2854 (m), 1719 (m), 1639 (w), 1587 (m), 1459 (m), 1414 (w), 1378 (w), 1366 (w), 821 (m). [a]$^{23}_{D}$ -21.3 (*c* 0.3, CH$_2$Cl$_2$).

**• 6-((1*R*,2*R*,5*S*)-2-Isopropyl-5-methylcyclohexyl)quinoline (ent-8e):**

Produced from substrate **ent-6e**. Yellow crystals. Yield: 65%. $^{1}$H NMR (500 MHz, CDCl$_3$): δ (ppm) 8.85 (dd, 1H, $J_1$ = 4.2 Hz, $J_2$ = 1.4 Hz), 8.14 (d, 1H, *J* = 8.0 Hz), 8.04 (d, 1H, *J* = 8.7 Hz), 7.80 (d, 1H, *J* = 2.0 Hz), 7.74 (dd, 1H, $J_1$ = 8.7 Hz, $J_2$ = 2.0 Hz), 7.39 (dd, 1H, $J_1$ = 8.2 Hz, $J_2$ = 4.2 Hz), 3.38 (bs, 1H), 1.90-2.00 (m, 2H, 2 signals overlapping), 1.72-1.84 (m, 2H, 2 signals overlapping), 1.69 (m, 1H), 1.32-1.52 (m, 3H, 3 signals overlapping), 1.06 (m, 1H), 0.87 (d,

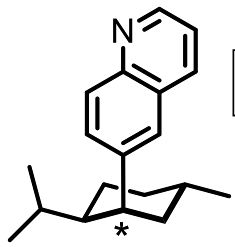

**ent-8e**

3H, $J$ = 6.3 Hz), 0.81 (d, 3H, $J$ = 6.3 Hz), 0.77 (d, 3H, $J$ = 6.3 Hz). $^{13}$C NMR (125 MHz, $CDCl_3$): δ (ppm) 149.2, 146.3, 144.4, 136.3, 133.2, 128.3, 128.0, 127.6, 120.9, 47.2, 43.0, 42.9, 35.3, 30.1, 26.8, 25.9, 22.7, 21.3, 21.2. FT-IR: ν ($cm^{-1}$) 2922 (s), 2867 (s), 2840 (m), 1592 (w), 1505 (m), 1455 (m), 836 (s), 796 (m), 773 (m). $[a]^{23}_{D}$ -2.5 (*c* 1.1, $CH_2Cl_2$).

- **3-((1*R*,2*R*,5*S*)-2-Isopropyl-5-methylcyclohexyl)benzofuran (ent-8f):**

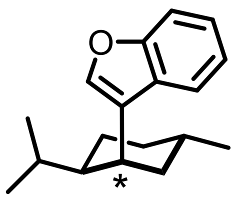

**ent-8f**

Produced from substrate **ent-6f**. Colorless oil. Yield: 54%. $^{1}$H NMR (500 MHz, $CDCl_3$): δ (ppm) 7.56 (dd, 1H, $J_1$ = 7.8 Hz, $J_2$ = 1.1 Hz), 7.54 (bs, 1H), 7.47 (d, 1H, $J$ = 8.0 Hz), 7.29 (dt, 1H, $J_1$ = 7.4 Hz, $J_2$ = 1.4 Hz), 7.24 (dt, 1H, $J_1$ = 7.5 Hz, $J_2$ = 1.1 Hz), 3.51 (bs, 1H), 1.96 (m, 1H), 1.77-1.85 (m, 2H, 2 signals overlapping), 1.42-1.52 (m, 2H, 2 signals overlapping), 1.23-1.39 (m, 3H, 3 signals overlapping), 1.00 (ddd, $J_1$ = 26.6 Hz, $J_2$ = 12.5 Hz, $J_3$ = 4.0 Hz), 0.89 (d, 3H, $J$ = 6.5 Hz), 0.80 (d, 3H, $J$ = 6.5 Hz), 0.74 (d, 3H, $J$ = 6.5 Hz). $^{13}$C NMR (125 MHz, $CDCl_3$): δ (ppm) 154.3, 141.4, 129.1, 124.0, 122.1, 120.9, 119.4, 111.2, 47.3, 40.5, 35.3, 32.5, 30.2, 27.2, 26.8, 22.3, 21.3, 21.2. FT-IR: ν ($cm^{-1}$) 2920 (s), 2867 (m), 2843 (m), 1452 (s), 1383 (w), 1271 (w), 1179 (w), 1098 (m), 1085 (m), 1011 (w), 859 (m), 741 (s). $[a]^{23}_{D}$ -9.3 (*c* 0.7, $CH_2Cl_2$).

- **((1*R*,2*R*,5*R*)-5-Isopropyl-2-methylcyclohexyl)benzene (9a):**

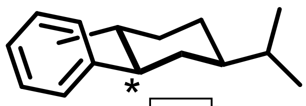

**9a**

Produced from substrate **7a**. Colorless oil. Yield: 81%. $^{1}$H NMR (500 MHz, $CDCl_3$): δ (ppm) 7.37 (d, 2H, $J$ = 8.1 Hz), 7.22-7.28 (m, 3H, 2 signals overlapping), 2.92 (td, 1H, $J_1$ = 12.7 Hz, $J_2$ = 4.0 Hz), 2.16 (m, 1H), 1.75-1.85 (m, 2H, 2 signals overlapping), 1.72 (m, 1H), 1.66 (m, 1H), 1.56-1.62 (m, 2H, 2 signals overlapping), 1.27-1.33 (m, 2H, 2 signals overlapping), 1.00 (d, 6H, $J$ = 6.9 Hz), 0.72 (d, 3H, $J$ = 7.1 Hz). $^{13}$C NMR (125 MHz, $CDCl_3$): δ (ppm) 146.3, 127.9, 127.6, 125.5, 46.6, 44.8, 34.3, 33.6, 33.1, 27.6, 23.3, 19.9, 19.8(5), 12.1. FT-IR: ν ($cm^{-1}$) 1646 (w), 1060 (s), 1032 (w), 871 (w). $[a]^{23}_{D}$ +3.5 (*c* 1.0, $CH_2Cl_2$).

- **1-((1*R*,2*R*,5*R*)-5-Isopropyl-2-methylcyclohexyl)-2-methoxybenzene (9b):**

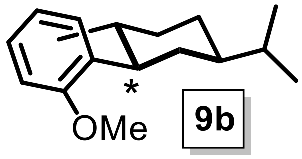

**9b**

Produced from substrate **7b**. Colorless oil. Yield: 54%. $^{1}$H NMR (500 MHz, $CDCl_3$): δ (ppm) 7.17 (td, 1H, $J_1$ = 7.5 Hz, $J_2$ = 1.7 Hz), 7.10 (dd, 1H, $J_1$ = 7.7 Hz, $J_2$ = 1.5 Hz), 6.92 (td, 1H, $J_1$ = 7.4, $J_2$ = 0.9 Hz), 6.84 (dd, 1H, $J_1$ = 8.3 Hz, $J_2$ = 0.9 Hz), 3.82 (s, 3H), 3.23 (td, 1H, $J_1$ = 13.1 Hz, $J_2$ = 3.5 Hz), 2.18 (m, 1H), 1.75 (m, 1H), 1.66 (m, 1H), 1.60 (m, 1H), 1.47-1.53 (m, 3H, 3 signals overlapping), 1.26 (m, 2H, 2 signals overlapping), 0.92 (d, 6H, $J$ =

6.9 Hz), 0.64 (d, 3H, *J* = 7.1 Hz). $^{13}$C NMR (125 MHz, $CDCl_3$): δ (ppm) 156.8, 134.5, 127.8, 126.3, 119.9, 110.0, 55.2, 45.1, 39.5, 33.4, 33.2, 30.7, 27.8, 23.6, 20.0, 19.8, 12.6. FT-IR: ν ($cm^{-1}$) 2962 (m), 2925 (m), 2870 (m), 1601 (s), 1489 (m), 1459 (m), 1419 (m), 1230 (m), 1088 (m), 1068 (m), 1040 (s), 748 (s), 727 (m). $[a]^{23}_D$ +11.6 (*c* 0.4 $CH_2Cl_2$).

• **2-((1*R*,2*R*,5*R*)-5-Isopropyl-2-methylcyclohexyl)-1,3-dimethoxybenzene (9c):**

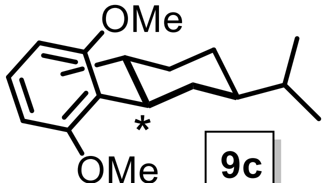


**9c**

Produced from substrate **7c**. Yellow oil. Yield: 36%. $^{1}$H NMR (500 MHz, $CDCl_3$): δ (ppm) 7.11 (t, 1H, *J* = 8.3 Hz), 6.54 (d, 2H, *J* = 8.3 Hz), 3.77 (s, 6H), 3.39 (dt, 1H, $J_1$ = 13.0 Hz, $J_2$ = 3.3 Hz), 2.26 (q, 1H, *J* = 12.8 Hz), 1.96 (m, 1H), 1.72 (tt, 1H, $J_1$ = 13.3 Hz, $J_2$ = 4.4 Hz), 1.60 (dq, 1H, $J_1$ = 13.0 Hz, $J_2$ = 3.0 Hz), 1.48 (m, 3H, 3 signals overlapping), 1.27 (qd, 1H, $J_1$ = 12.8 Hz, $J_2$ = 3.7 Hz), 1.12 (m, 1H), 0.90 (d, 3H, *J* = 6.9 Hz), 0.89 (d, 3H, *J* = 6.9 Hz), 0.83 (d, 3H, *J* = 7.1 Hz). $^{13}$C NMR (125 MHz, $CDCl_3$): δ (ppm) 159.2, 126.5, 122.6, 104.5, 55.7, 46.1, 39.7, 34.0, 33.2, 33.1, 28.5, 24.1, 20.0(4), 20.0(0), 14.0. FT-IR: ν ($cm^{-1}$) 2955 (m), 2925 (m), 2869 (m), 2834 (m), 1587 (s), 1468 (s), 1434 (m), 1376 (m), 1273 (w), 1260 (m), 1246 (s), 1230 (s), 1167 (w), 1141 (s), 1101 (s), 1041 (w), 806 (w), 784 (m), 760 (m), 724 (s), 708 (w). $[a]^{23}_D$ +6.1 (*c* 0.5, $CH_2Cl_2$).

• **4-((1*R*,2*R*,5*R*)-5-Isopropyl-2-methylcyclohexyl)pyridine (9d):**

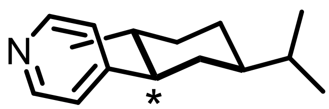


**9d**

Produced from substrate **7d**. Yellow oil. Yield: 89%. $^{1}$H NMR (500 MHz, $CDCl_3$): δ (ppm) 8.48 (d, 2H, *J* = 6.1 Hz), 7.10 (d, 2H, *J* = 6.1 Hz), 2.81 (dt, 1H, $J_1$ = 12.7 Hz, $J_2$ = 3.8 Hz), 2.10 (oct, 1H, *J* = 3.7 Hz), 1.70-1.76 (m, 2H, 2 signals overlapping), 1.63 (m, 1H), 1.47-1.57 (m, 3H, 3 signals overlapping), 1.16-1.28 (m, 2H, 2 signals overlapping), 0.92 (d, 6H, *J* = 6.9 Hz), 0.61 (d, 3H, *J* = 7.1 Hz). $^{13}$C NMR (125 MHz, $CDCl_3$): δ (ppm) 155.4, 149.2, 123.1, 45.9, 44.4, 33.7, 33.4, 33.0, 26.7, 23.1, 19.8, 12.0. FT-IR: ν ($cm^{-1}$) 2956 (s), 2928 (s), 2856 (s), 1597 (s), 1558 (m), 1508 (m), 1466 (s), 1418 (m), 1365 (m), 1288 (m), 1069 (w), 990 (w), 822 (m), 764 (w). $[a]^{23}_D$ +2.0 (*c* 1.3, $CH_2Cl_2$).

• **6-((1*R*,2*R*,5*R*)-5-Isopropyl-2-methylcyclohexyl)quinoline (9e):**

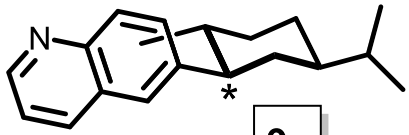


**9e**

Produced from substrate **7e**. Yellow oil. Yield: 46%. $^{1}$H NMR (500 MHz, $CDCl_3$): δ (ppm) 8.86 (dd, 1H, $J_1$ = 4.4 Hz, $J_2$ = 1.6 Hz), 8.16 (d, 1H, *J* = 8.3 Hz), 8.07 (d, 1H, *J* = 8.7 Hz), 7.61 (dd, 1H, $J_1$ = 8.8 Hz, $J_2$ = 1.9 Hz), 7.55 (s, 1H), 7.40 (dd, 1H, $J_1$ = 8.1 Hz, $J_2$ = 4.1 Hz), 3.02 (td, 1H, $J_1$ = 12.6, $J_2$ = 3.7 Hz), 2.21 (m, 1H), 1.72-1.82 (m, 3H, 3 signals overlapping), 1.68 (q, 1H, *J*

= 11.7 Hz), 1.51-1.61 (m, 2H, 2 signals overlapping), 1.24-1.30 (m, 2H, 2 signals overlapping), 0.96 (d, 3H, *J* = 6.9 Hz), 0.96 (d, 3H, *J* = 6.9 Hz), 0.63 (d, 3H, *J* = 7.1 Hz). $^{13}$C NMR (125 MHz, $CDCl_3$): δ (ppm) 149.1, 146.5, 145.1, 136.3, 130.9, 128.3, 128.2, 124.9, 120.9, 46.4, 44.7, 34.0, 33.6, 33.1, 27.6, 23.2, 19.8(9), 19.8(7), 12.1. FT-IR: v (cm$^{-1}$) 2955 (m), 2919 (s), 2867 (m), 1593 (w), 1571 (w), 1498 (s), 1464 (m), 1439 (w), 1378 (m), 1366 (m), 1170 (w), 1117 (w), 883 (m), 834 (s), 796 (m), 777 (m). $[a]^{23}_D$ +15.8 (*c* 1.1, $CH_2Cl_2$).

**• 3-((1*R*,2*R*,5*R*)-5-Isopropyl-2-methylcyclohexyl)benzofuran (9f):**

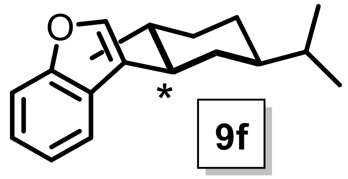

**9f**

Produced from substrate **7f**. Colorless oil. Yield: 35%. $^1$H NMR (500 MHz, $C_6D_6$): δ (ppm) 7.47-7.43 (m, 2H, 2 signals overlapping), 7.13-7.10 (m, 2H, 2 signals overlapping), 7.05 (d, 1H, *J* = 1.18 Hz), 2.85 (dt, 1H, $J_1$ = 12.6 Hz, $J_2$ = 4.3 Hz), 2.29 (m, 1H), 1.64-1.59 (m, 2H, 2 signals overlapping), 1.57 (m, 1H), 1.38 (m, 1H), 1.33 (m, 2H, 2 signals overlapping), 1.14 (m, 1H), 1.08 (m, 1H), 0.90 (d, 6H, *J* = 6.8 Hz), 0.62 (d, 3H, *J* = 7.2 Hz). $^{13}$C NMR (125 MHz, $C_6D_6$): δ (ppm) 155.9, 154.8, 140.8, 124.1, 122.1, 120.2, 119.6, 111.6, 44.5, 37.5, 32.9, 32.7, 31.1, 27.8, 23.2, 19.7, 19.6, 12.3. FT-IR: v (cm$^{-1}$) 2956 (m), 2925 (m), 1726 (m), 1452 (s), 1382 (w), 1366 (w), 1277 (m), 1190 (w), 1120 (w), 1090 (m), 1072 (w), 857 (m), 741 (s). $[a]^{23}_D$ +31.6 (*c* 0.2, $CH_2Cl_2$).

**• ((1*S*,2*S*,5*S*)-5-Isopropyl-2-methylcyclohexyl)benzene (ent-9a):**

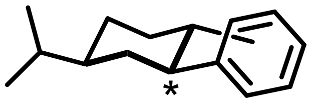

**ent-9a**

Produced from substrate **ent-7a**. Colorless oil. Yield: 99%. $^1$H NMR (500 MHz, $CDCl_3$): δ (ppm) 7.37 (d, 2H, *J* = 8.1 Hz), 7.22-7.28 (m, 3H, 2 signals overlapping), 2.92 (td, 1H, $J_1$ = 12.7 Hz, $J_2$ = 4.0 Hz), 2.16 (m, 1H), 1.75-1.85 (m, 2H, 2 signals overlapping), 1.72 (m, 1H), 1.66 (m, 1H), 1.56-1.62 (m, 2H, 2 signals overlapping), 1.27-1.33 (m, 2H, 2 signals overlapping), 1.00 (d, 6H, *J* = 6.9 Hz), 0.72 (d, 3H, *J* = 7.1 Hz). $^{13}$C NMR (125 MHz, $CDCl_3$): δ (ppm) 146.3, 127.9, 127.6, 125.5, 46.6, 44.8, 34.3, 33.6, 33.1, 27.6, 23.3, 19.9, 19.8(5), 12.1. FT-IR: v (cm$^{-1}$) 1646 (w), 1061 (s), 1034 (w), 870 (w). $[a]^{23}_D$ -3.1 (*c* 0.5, $CH_2Cl_2$).

**• 1-((1*S*,2*S*,5*S*)-5-Isopropyl-2-methylcyclohexyl)-2-methoxybenzene (ent-9b):**

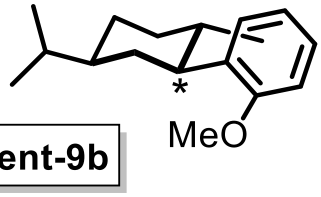


**ent-9b**

Produced from substrate **ent-7b**. Colorless oil. Yield: 87%. $^1$H NMR (500 MHz, $CDCl_3$): δ (ppm) 7.17 (td, 1H, $J_1$ = 7.5 Hz, $J_2$ = 1.7 Hz), 7.10 (dd, 1H, $J_1$ = 7.7 Hz, $J_2$ = 1.5 Hz), 6.92 (td, 1H, $J_1$ = 7.4, $J_2$ = 0.9 Hz), 6.84 (dd, 1H, $J_1$ = 8.3 Hz, $J_2$ = 0.9 Hz), 3.82 (s, 3H), 3.23 (td, 1H, $J_1$ = 13.1 Hz, $J_2$ = 3.5 Hz), 2.18 (m, 1H), 1.75 (m, 1H), 1.66 (m, 1H), 1.60 (m, 1H), 1.47-1.53 (m, 3H, 3 signals overlapping), 1.26 (m, 2H, 2 signals overlapping), 0.92 (d, 6H, *J* = 6.9 Hz), 0.64 (d, 3H, *J* = 7.1 Hz). $^{13}$C NMR (125 MHz, $CDCl_3$): δ

(ppm) 156.8, 134.5, 127.8, 126.3, 119.9, 110.0, 55.2, 45.1, 39.5, 33.4, 33.2, 30.7, 27.8, 23.6, 20.0, 19.8, 12.6. FT-IR: ν ($cm^{-1}$) 2965 (m), 2923 (s), 2869 (m), 1600 (m), 1491 (s), 1462 (m), 1418 (w), 1233 (s), 1086 (m), 1069 (m), 1038 (s), 748 (s), 727 (m). $[a]^{23}_D$ -12.9 (*c* 0.6, $CH_2Cl_2$).

**• 2-((1*S*,2*S*,5*S*)-5-Isopropyl-2-methylcyclohexyl)-1,3-dimethoxybenzene (ent-9c):**

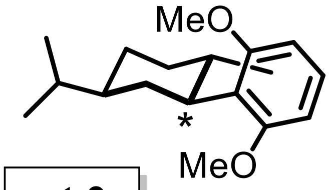


**ent-9c**

Produced from substrate **ent-7c**. Yellow oil. Yield: 86%. $^1$H NMR (500 MHz, $CDCl_3$): δ (ppm) 7.11 (t, 1H, *J* = 8.3 Hz), 6.54 (d, 2H, *J* = 8.3 Hz), 3.77 (s, 6H), 3.39 (dt, 1H, $J_1$ = 13.0 Hz, $J_2$ = 3.3 Hz), 2.26 (q, 1H, *J* = 12.8 Hz), 1.96 (m, 1H), 1.72 (tt, 1H, $J_1$ = 13.3 Hz, $J_2$ = 4.4 Hz), 1.60 (dq, 1H, $J_1$ = 13.0 Hz, $J_2$ = 3.0 Hz), 1.48 (m, 3H, 3 signals overlapping), 1.27 (qd, 1H, $J_1$ = 12.8 Hz, $J_2$ = 3.7 Hz), 1.12 (m, 1H), 0.90 (d, 3H, *J* = 6.9 Hz), 0.89 (d, 3H, *J* = 6.9 Hz), 0.83 (d, 3H, *J* = 7.1 Hz). $^{13}$C NMR (125 MHz, $CDCl_3$): δ (ppm) 159.2, 126.5, 122.6, 104.5, 55.7, 46.1, 39.7, 34.0, 33.2, 33.1, 28.5, 24.1, 20.0(4), 20.0(0), 14.0. FT-IR: ν ($cm^{-1}$) 2955 (m), 2924 (s), 2869 (m), 2834 (m), 1587 (s), 1491 (w), 1468 (s), 1434 (m), 1376 (w), 1244 (s), 1232 (s), 1141 (s), 1102 (s), 785 (m), 751 (m), 724 (m). $[a]^{23}_D$ -5.6 (*c* 0.6, $CH_2Cl_2$).

**• 4-((1*S*,2*S*,5*S*)-5-Isopropyl-2-methylcyclohexyl)pyridine (ent-9d):**

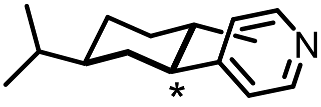


**ent-9d**

Produced from substrate **ent-7d**. Yellow oil. Yield: 95%. $^1$H NMR (500 MHz, $CDCl_3$): δ (ppm) 8.48 (d, 2H, *J* = 6.1 Hz), 7.10 (d, 2H, *J* = 6.1 Hz), 2.81 (dt, 1H, $J_1$ = 12.7 Hz, $J_2$ = 3.8 Hz), 2.10 (oct, 1H, *J* = 3.7 Hz), 1.70-1.76 (m, 2H, 2 signals overlapping), 1.63 (m, 1H), 1.47-1.57 (m, 3H, 3 signals overlapping), 1.16-1.28 (m, 2H, 2 signals overlapping), 0.92 (d, 6H, *J* = 6.9 Hz), 0.61 (d, 3H, *J* = 7.1 Hz). $^{13}$C NMR (125 MHz, $CDCl_3$): δ (ppm) 155.4, 149.2, 123.1, 45.9, 44.4, 33.7, 33.4, 33.0, 26.7, 23.1, 19.8, 12.0. FT-IR: ν ($cm^{-1}$) 2956 (s), 2929 (s), 2859 (s), 1597 (s), 1557 (w), 1465 (m), 1415 (m), 1381 (w), 1368 (w), 1287 (m), 1071 (w), 994 (w), 824 (m), 763 (w). $[a]^{23}_D$ -1.5 (*c* 1.1, $CH_2Cl_2$).

**• 6-((1*S*,2*S*,5*S*)-5-Isopropyl-2-methylcyclohexyl)quinoline (ent-9e):**

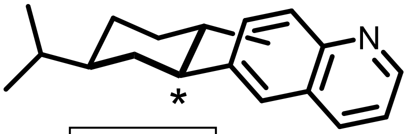


**ent-9e**

Produced from substrate **ent-7e**. Yellow oil. Yield: 25%. $^1$H NMR (500 MHz, $CDCl_3$): δ (ppm) 8.86 (dd, 1H, $J_1$ = 4.4 Hz, $J_2$ = 1.6 Hz), 8.16 (d, 1H, *J* = 8.3 Hz), 8.07 (d, 1H, *J* = 8.7 Hz), 7.61 (dd, 1H, $J_1$ = 8.8 Hz, $J_2$ = 1.9 Hz), 7.55 (s, 1H), 7.40 (dd, 1H, $J_1$ = 8.1 Hz, $J_2$ = 4.1 Hz), 3.02 (td, 1H, $J_1$ = 12.6, $J_2$ = 3.7 Hz), 2.21 (m, 1H), 1.72-1.82 (m, 3H, 3 signals overlapping), 1.68 (q, 1H, *J* = 11.7 Hz), 1.51-1.61 (m, 2H, 2 signals overlapping), 1.24-1.30 (m, 2H, 2 signals

overlapping), 0.96 (d, 3H, $J$ = 6.9 Hz), 0.96 (d, 3H, $J$ = 6.9 Hz), 0.63 (d, 3H, $J$ = 7.1 Hz). $^{13}$C NMR (125 MHz, $CDCl_3$): δ (ppm) 149.1, 146.5, 145.1, 136.3, 130.9, 128.3, 128.2, 124.9, 120.9, 46.4, 44.7, 34.0, 33.6, 33.1, 27.6, 23.2, 19.8(9), 19.8(7), 12.1. FT-IR: ν (cm$^{-1}$) 2954 (m), 2918 (s), 2867 (m), 1593 (w), 1571 (w), 1498 (s), 1464 (m), 1439 (w), 1378 (m), 1366 (m), 1171 (w), 1115 (w), 883 (m), 834 (s), 796 (m), 778 (m). [a]$^{23}_{D}$ -16.6 ($c$ 0.5, $CH_2Cl_2$).

**• 3-((1*S*,2*S*,5*S*)-5-Isopropyl-2-methylcyclohexyl)benzofuran (ent-9f):**

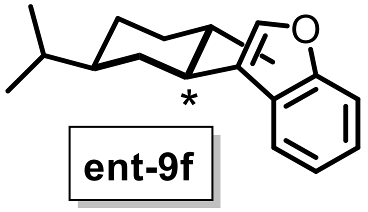

**ent-9f**

Produced from substrate **ent-7f**. Colorless oil. Yield: 39%. $^1$H NMR (500 MHz, $C_6D_6$): δ (ppm) 7.47-7.43 (m, 2H, 2 signals overlapping), 7.13-7.10 (m, 2H, 2 signals overlapping), 7.05 (d, 1H, $J$ = 1.18 Hz), 2.85 (dt, 1H, $J_1$ = 12.6 Hz, $J_2$ = 4.3 Hz), 2.29 (m, 1H), 1.64-1.59 (m, 2H, 2 signals overlapping), 1.57 (m, 1H), 1.38 (m, 1H), 1.33 (m, 2H, 2 signals overlapping), 1.14 (m, 1H), 1.08 (m, 1H), 0.90 (d, 6H, $J$ = 6.8 Hz), 0.62 (d, 3H, $J$ = 7.2 Hz). $^{13}$C NMR (125 MHz, $C_6D_6$): δ (ppm) 155.9, 154.8, 140.8, 124.1, 122.1, 120.2, 119.6, 111.6, 44.5, 37.5, 32.9, 32.7, 31.1, 27.8, 23.2, 19.7, 19.6, 12.3. FT-IR: ν (cm$^{-1}$) 2954 (m), 2923 (m), 1726 (m), 1450 (s), 1382 (w), 1366 (w), 1277 (m), 1190 (w), 1121 (w), 1090 (m), 1072 (w), 857 (m), 740 (s). [a]$^{23}_{D}$ -32.8 ($c$ 0.3, $CH_2Cl_2$).

## III. Compound 1D and 2D NMR Spectra

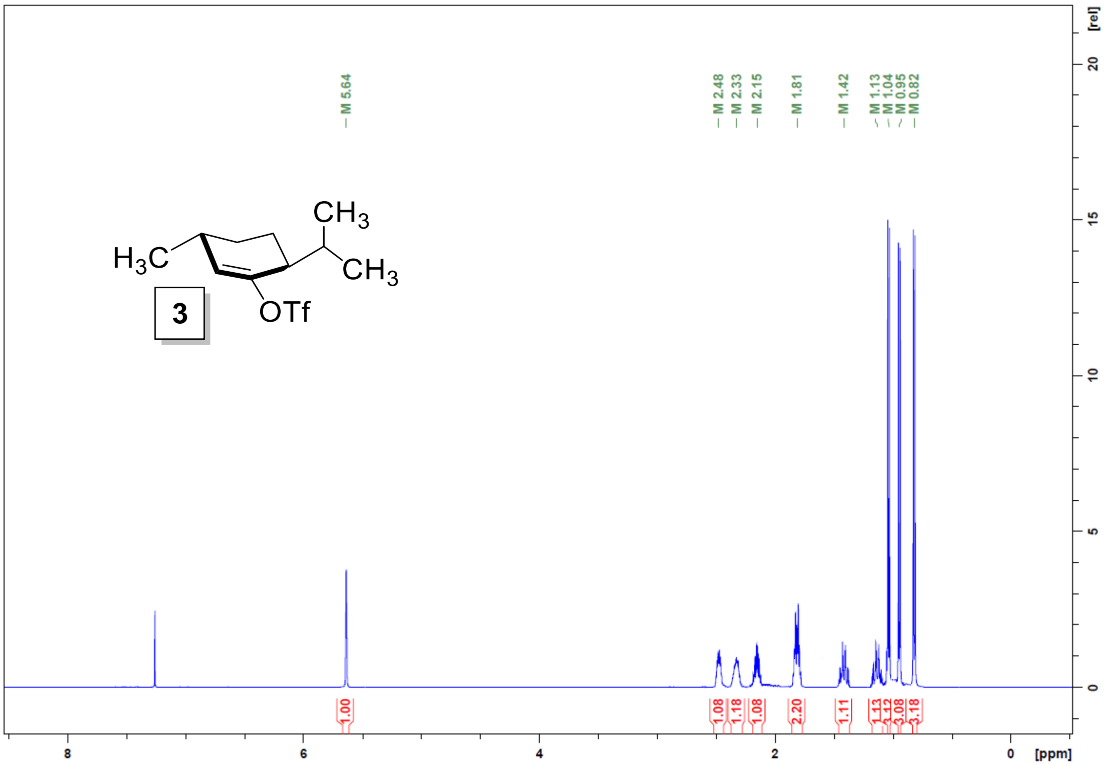


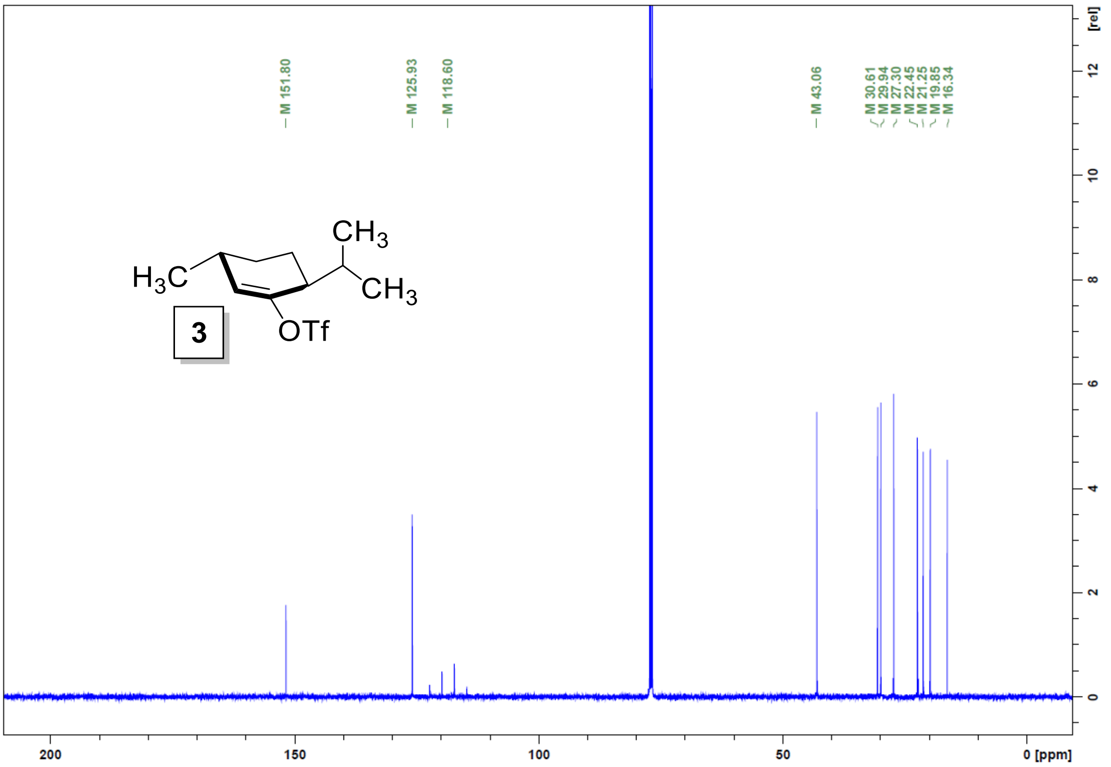

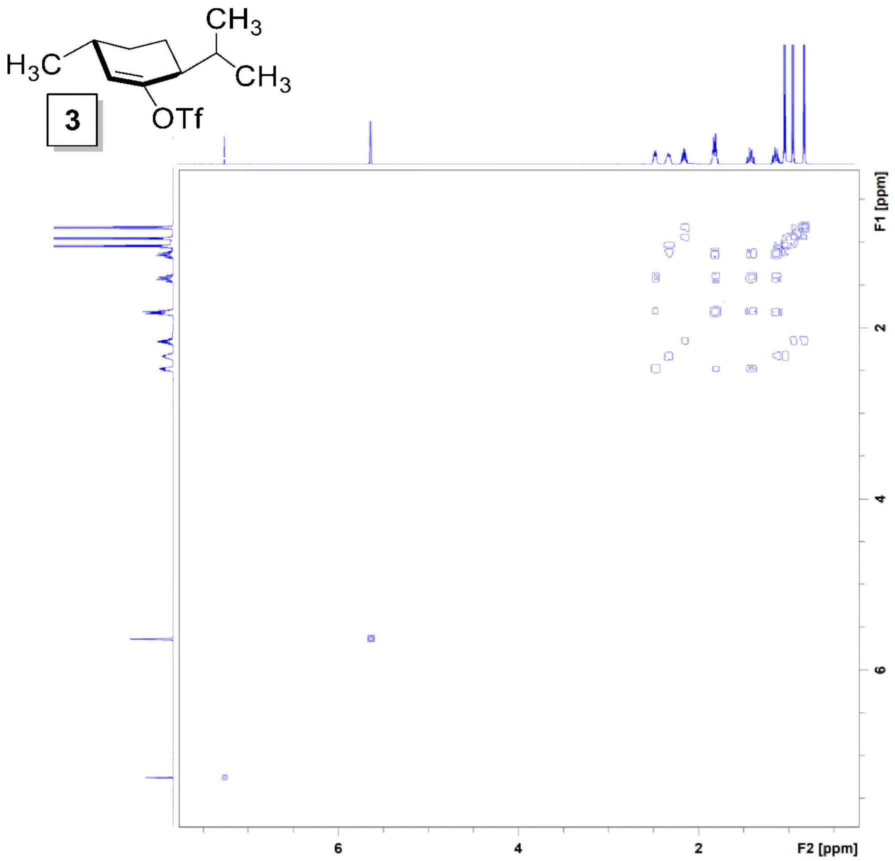
CH3
H3C
CH3
OTf
3
F1 [ppm]
2
4
6
6
4
2
F2 [ppm]

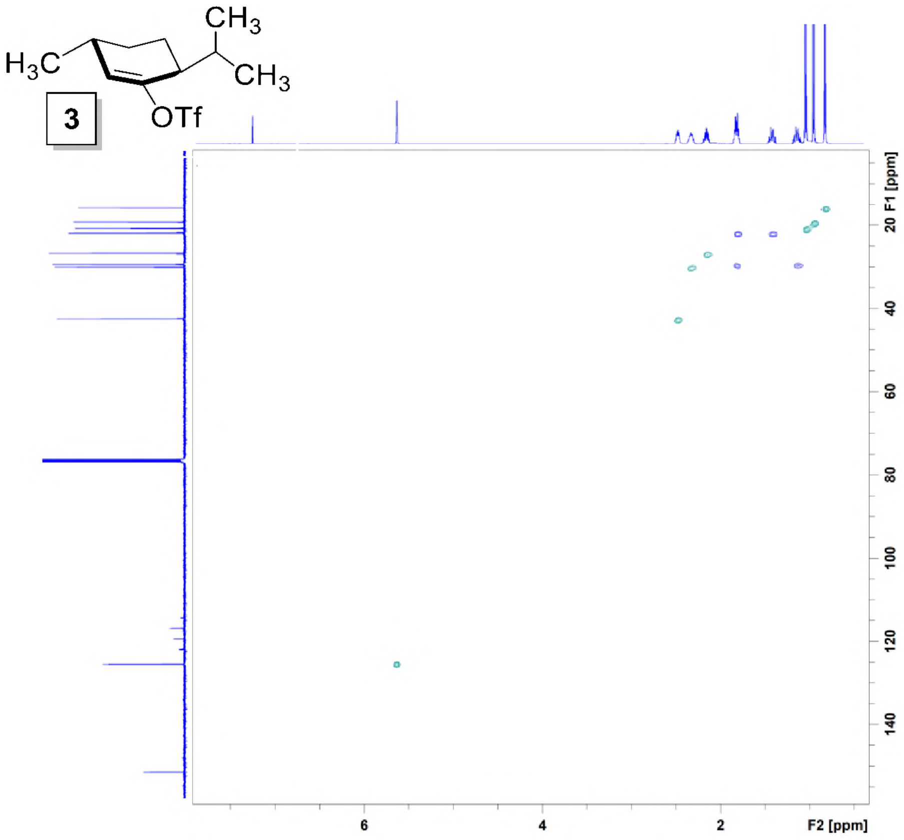
CH3
H3C
CH3
OTf
3
20 F1 [ppm]
40
60
80
100
120
140
6
4
2
F2 [ppm]

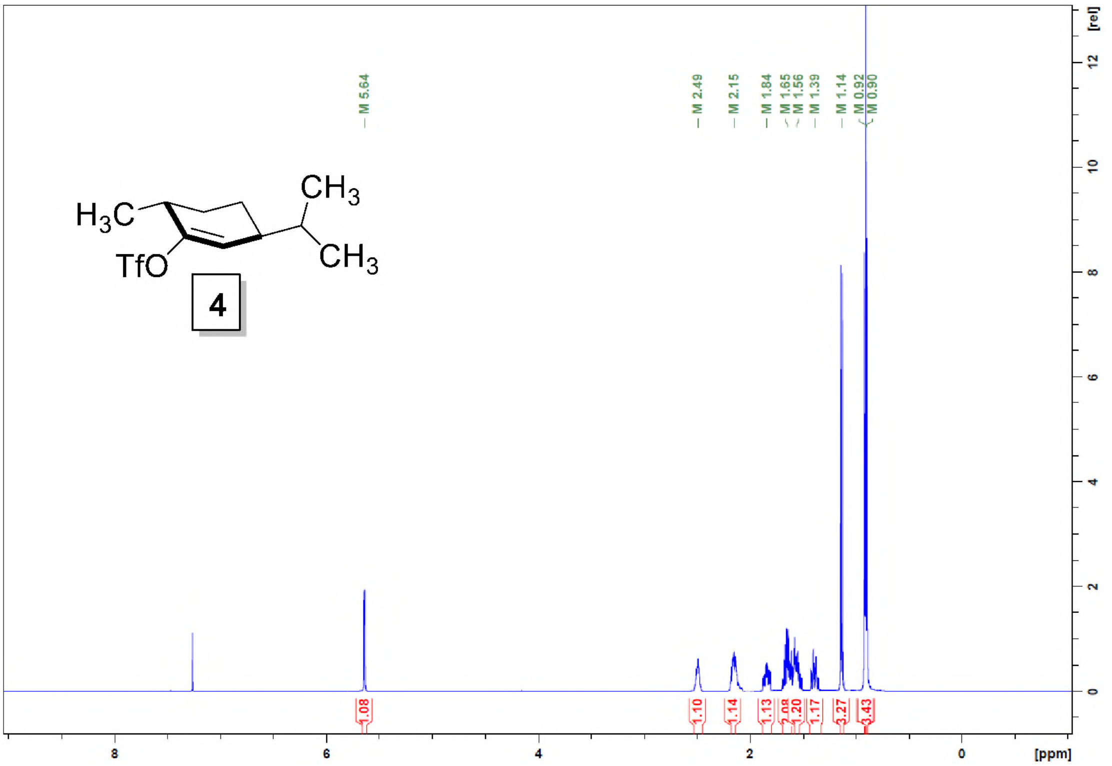
M 5.64
M 2.49
M 2.15
M 1.84
M 1.65
M 1.56
M 1.39
M 1.14
M 0.92
M 0.90
1.08
1.10
1.14
1.13
2.08
2.20
1.17
3.27
3.43
H3C
TfO
CH3
CH3
4
[rel]
[ppm]

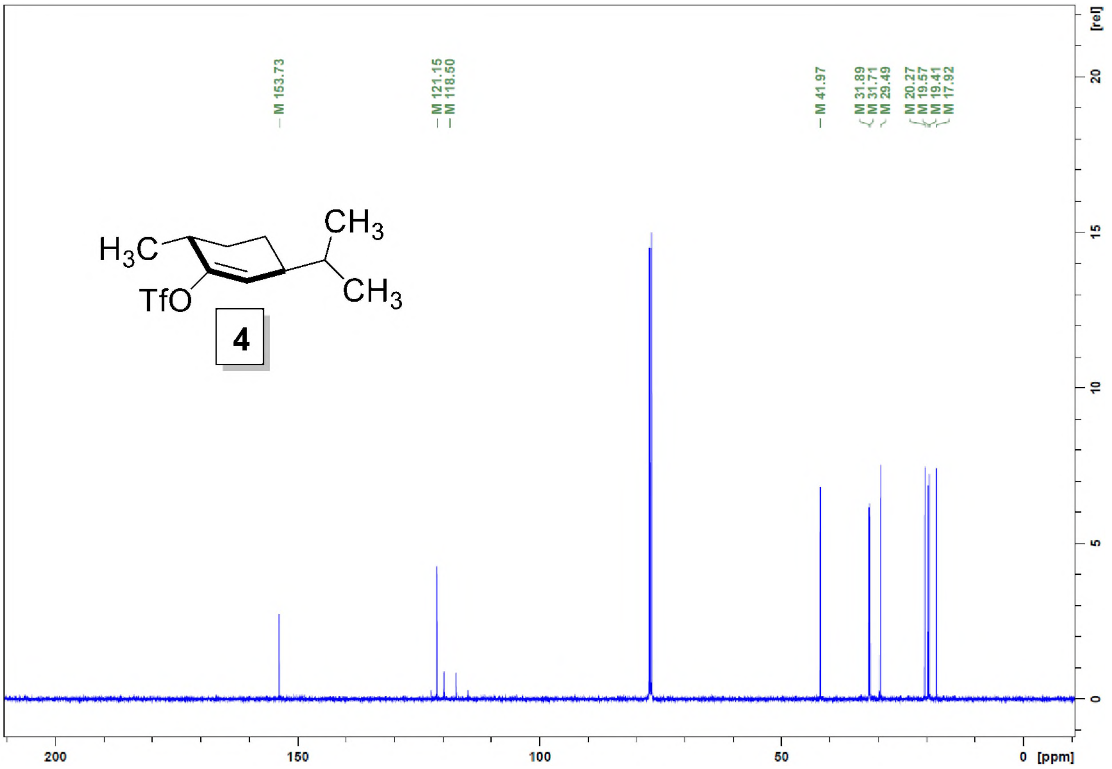
M 153.73
M 121.15
M 118.50
M 41.97
M 31.89
M 31.71
M 29.49
M 20.27
M 19.57
M 19.41
M 17.92
H3C
TfO
CH3
CH3
4
[rel]
[ppm]

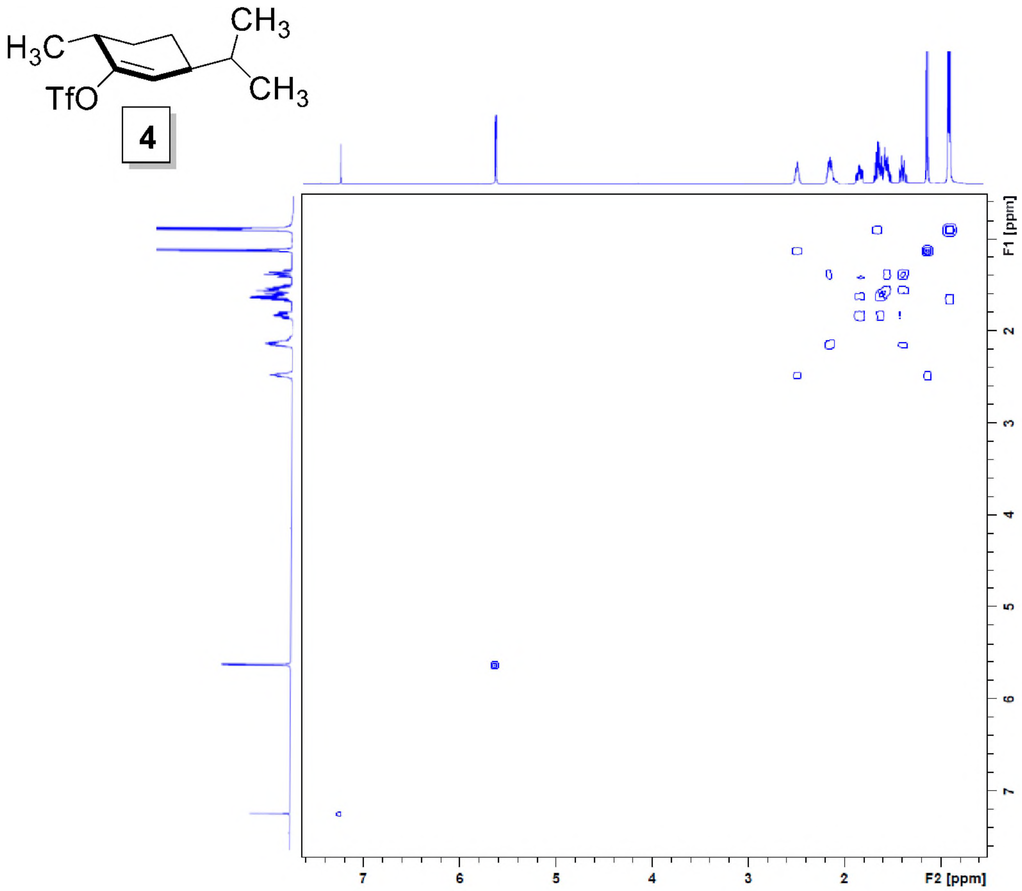
H3C
CH3
TfO
CH3
4
F1 [ppm]
F2 [ppm]


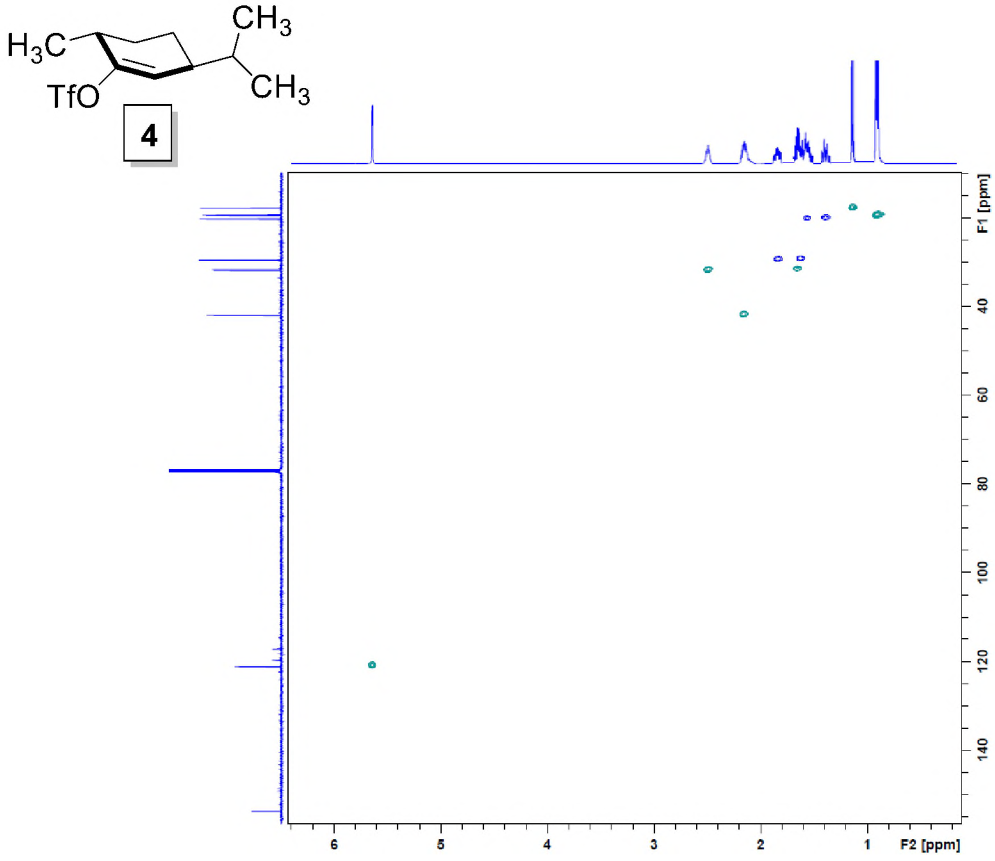
H3C
CH3
TfO
CH3
4
F1 [ppm]
F2 [ppm]

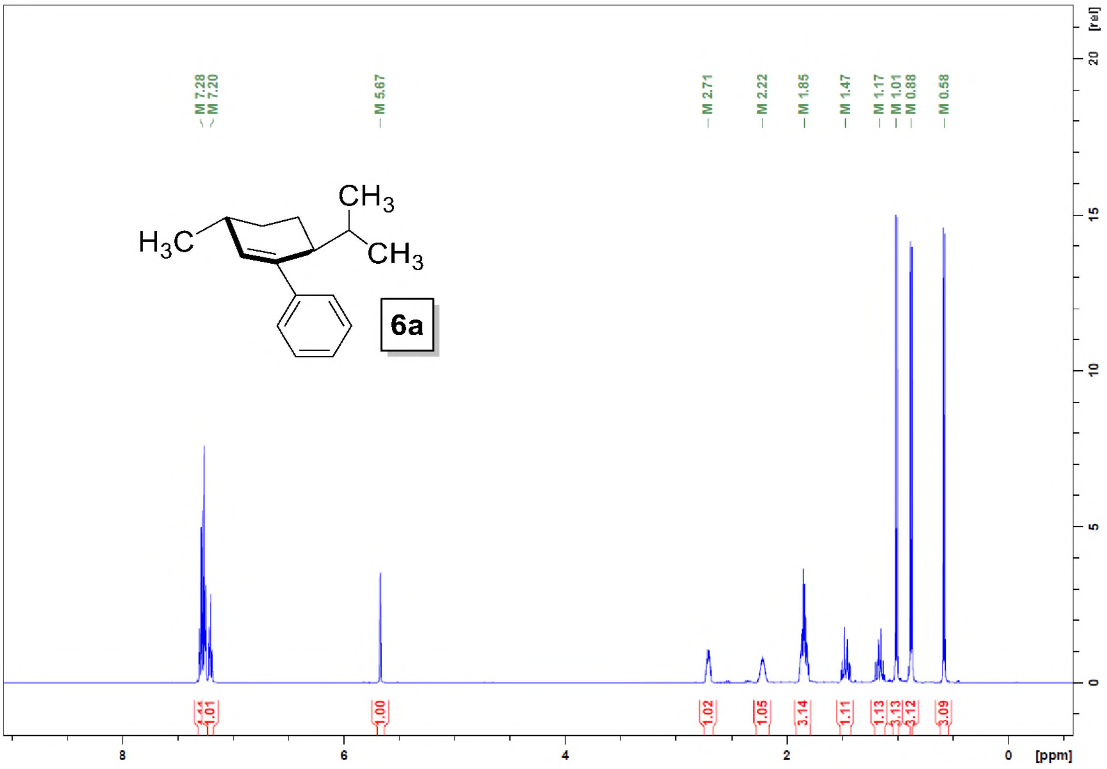
M 7.28
M 7.20
M 5.67
M 2.71
M 2.22
M 1.85
M 1.47
M 1.17
M 1.01
M 0.88
M 0.58
$H_3C$
$CH_3$
$CH_3$
6a
4.44
1.01
1.00
1.02
1.05
3.14
1.11
1.13
3.13
3.12
3.09
[ppm]
[rel]

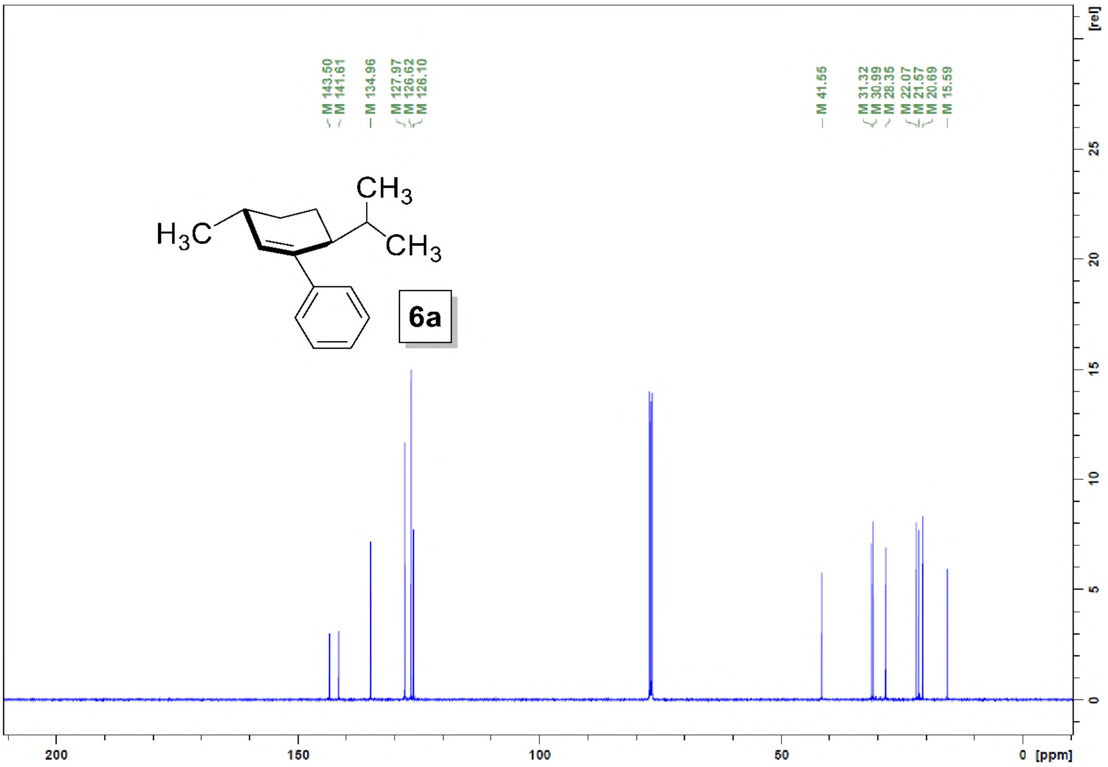
M 143.50
M 141.61
M 134.96
M 127.97
M 126.62
M 126.10
M 41.55
M 31.32
M 30.99
M 28.35
M 22.07
M 21.57
M 20.69
M 15.59
$H_3C$
$CH_3$
$CH_3$
6a
[ppm]
[rel]

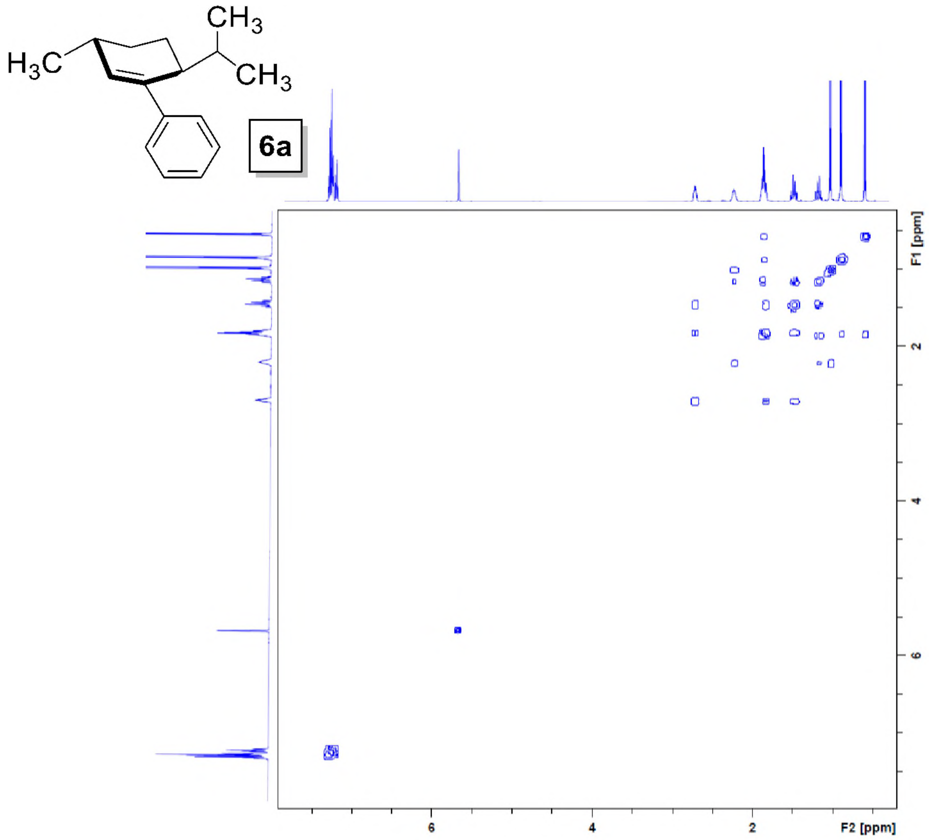

$H_3C$
$CH_3$
$CH_3$
6a
F1 [ppm]
2
4
6
F2 [ppm]


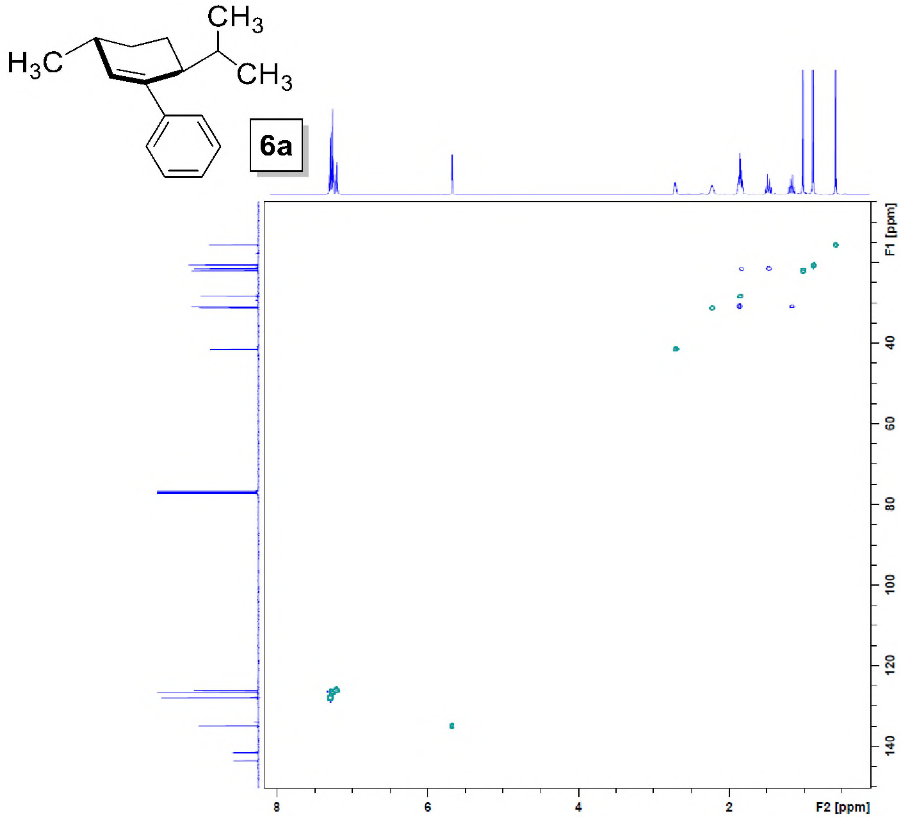

$H_3C$
$CH_3$
$CH_3$
6a
F1 [ppm]
40
60
80
100
120
140
8
6
4
2
F2 [ppm]

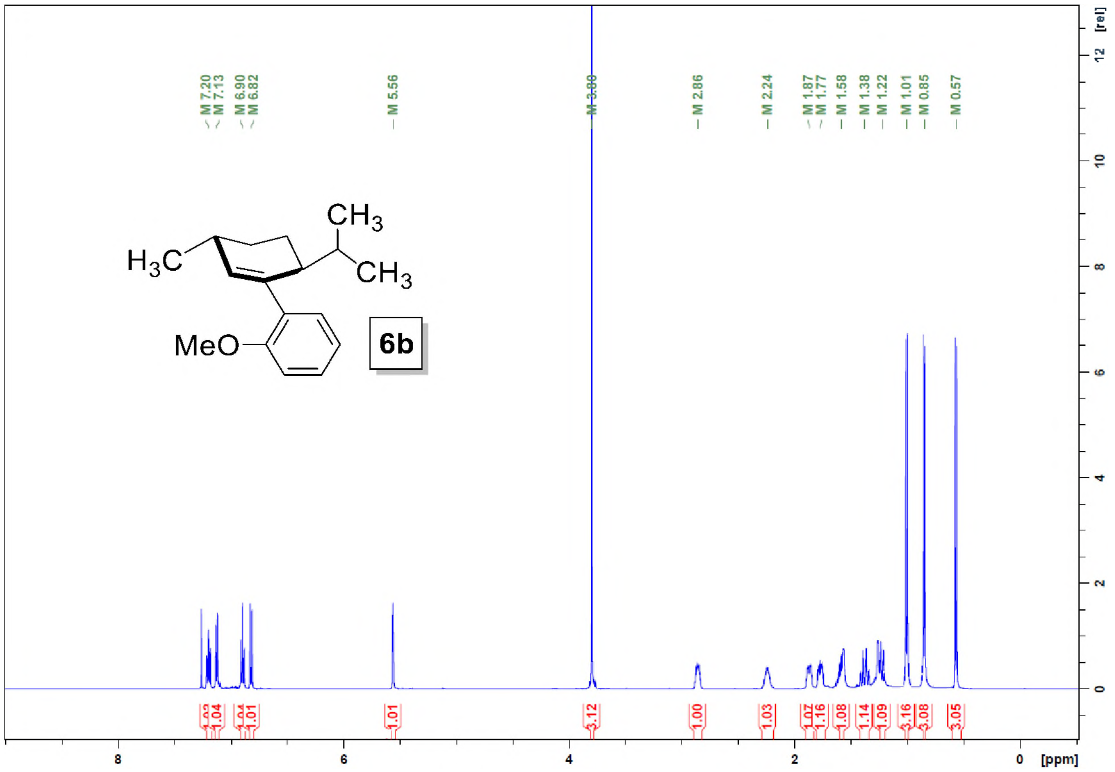
M 7.20
M 7.13
M 6.90
M 6.82
M 5.56
M 3.80
M 2.86
M 2.24
M 1.87
M 1.77
M 1.58
M 1.38
M 1.22
M 1.01
M 0.85
M 0.57
$CH_3$
$H_3C$
$CH_3$
MeO
6b
1.04
1.01
1.01
3.12
1.00
1.03
1.07
1.16
1.08
1.14
1.09
3.16
3.08
3.05
8
6
4
2
0
[ppm]
[rel]

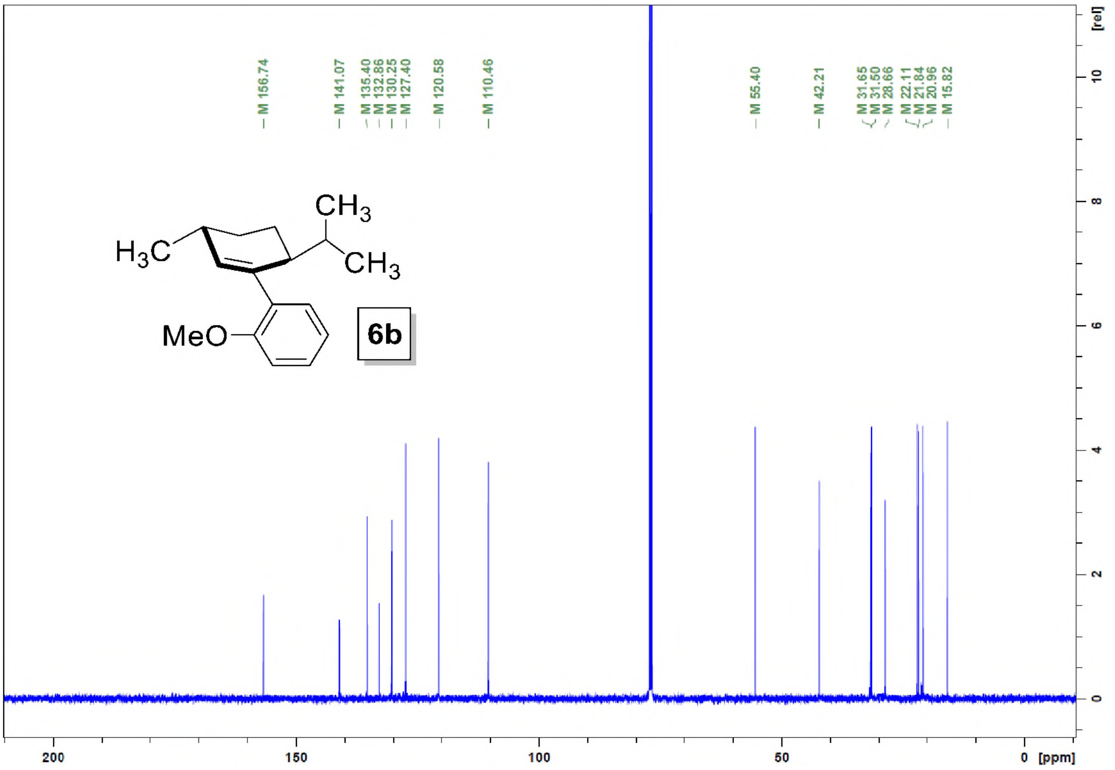
M 156.74
M 141.07
M 135.40
M 132.86
M 130.25
M 127.40
M 120.58
M 110.46
M 55.40
M 42.21
M 31.65
M 31.50
M 28.66
M 22.11
M 21.84
M 20.96
M 15.82
$CH_3$
$H_3C$
$CH_3$
MeO
6b
200
150
100
50
0
[ppm]
[rel]

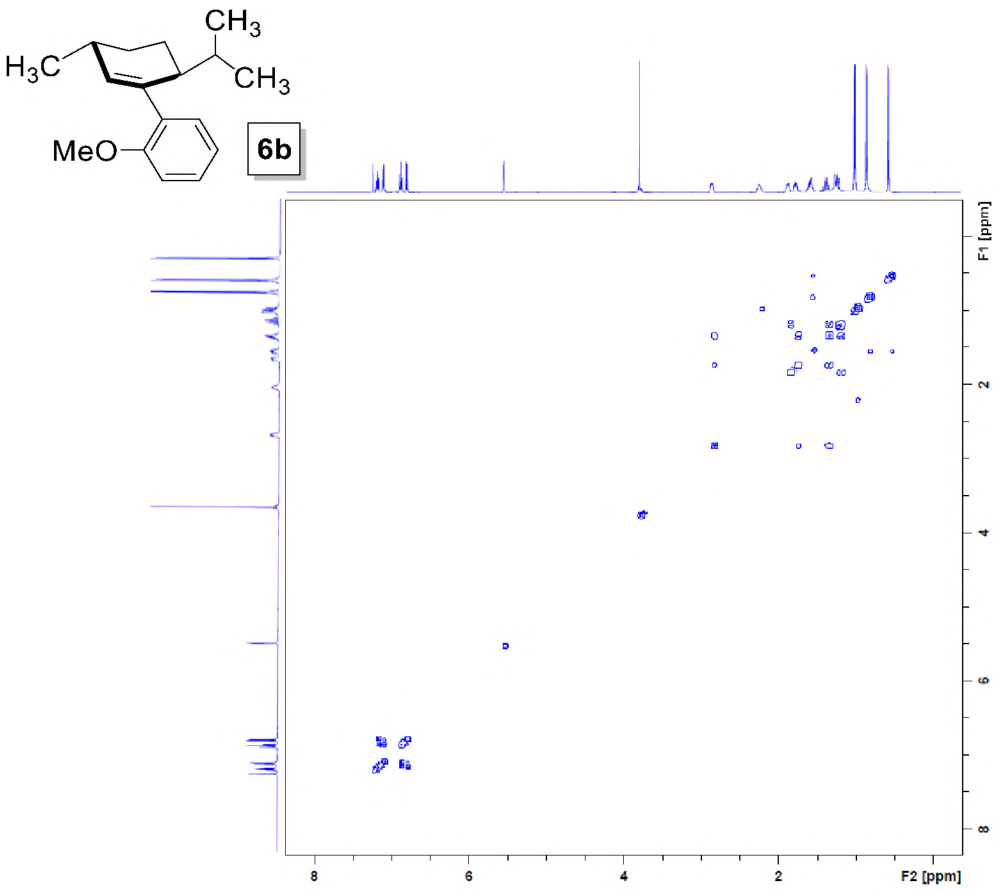


6b

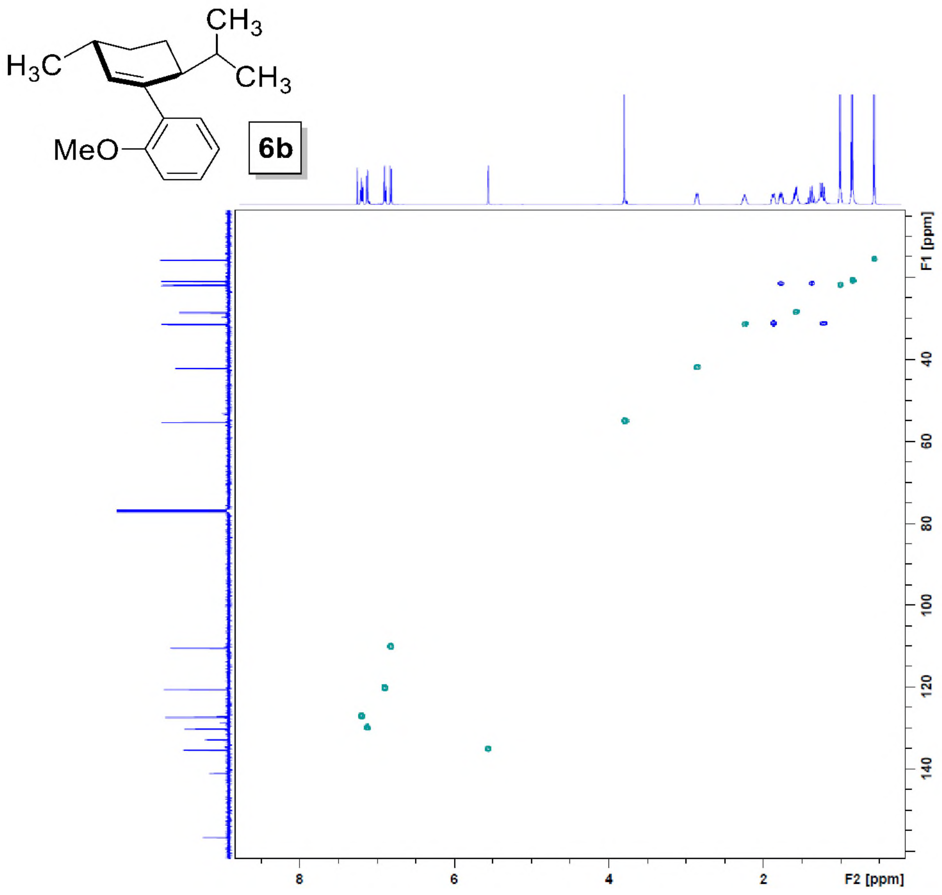


6b

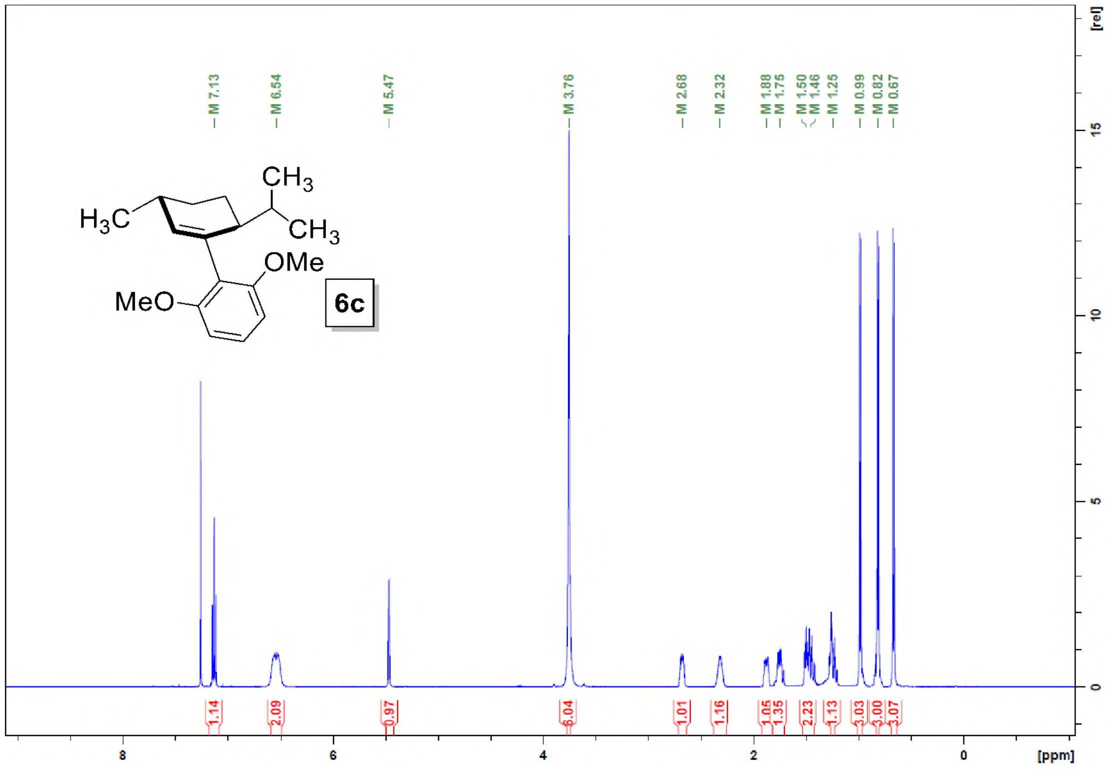

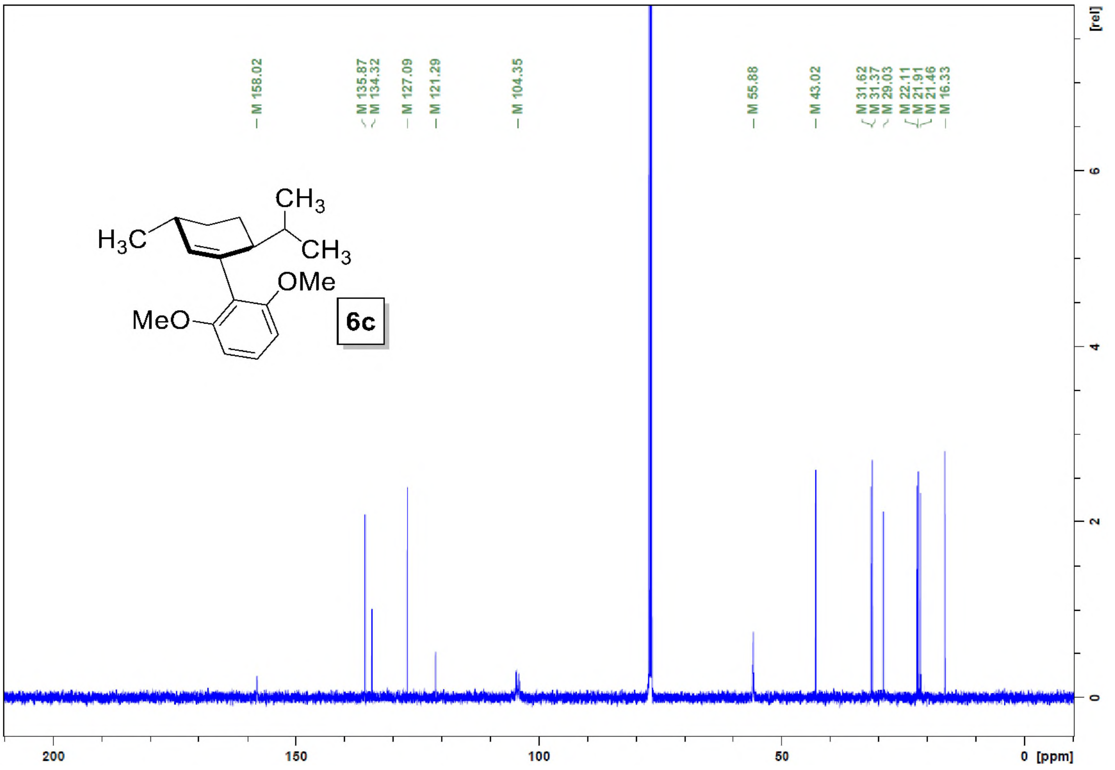

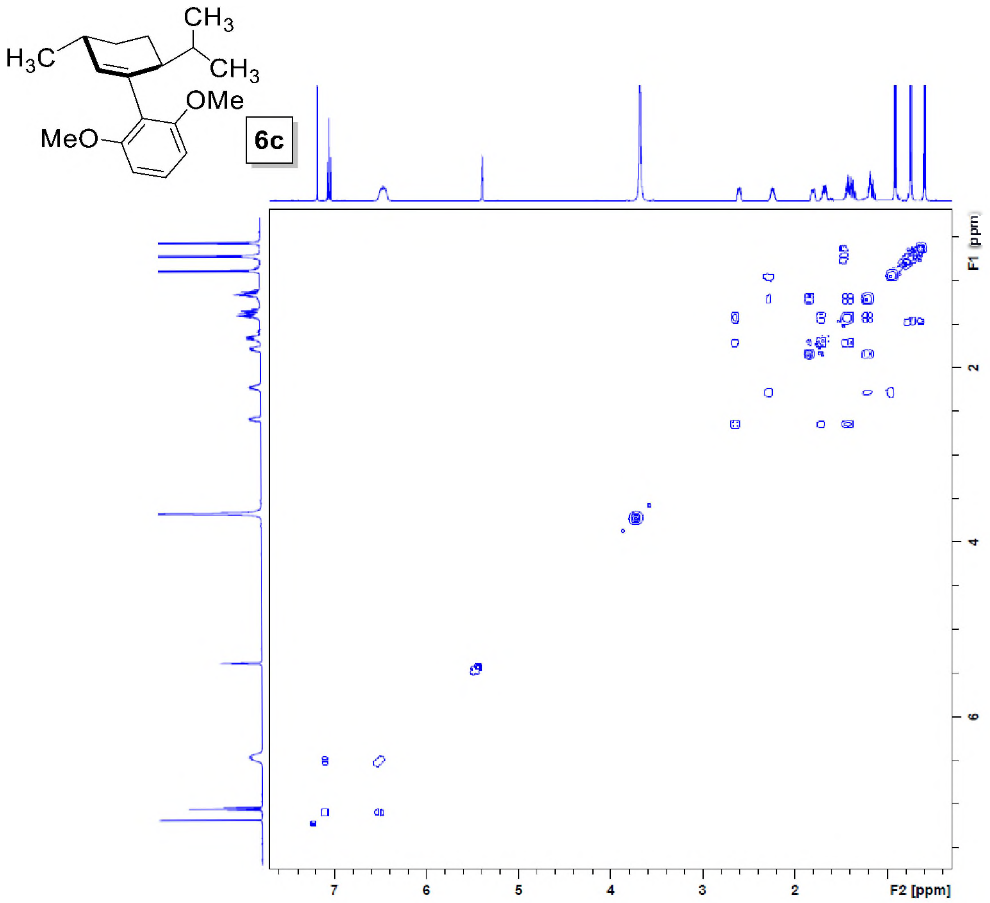

$H_3C$
$CH_3$
$CH_3$
OMe
MeO
6c
F1 [ppm]
F2 [ppm]
2
4
6
7
6
5
4
3
2


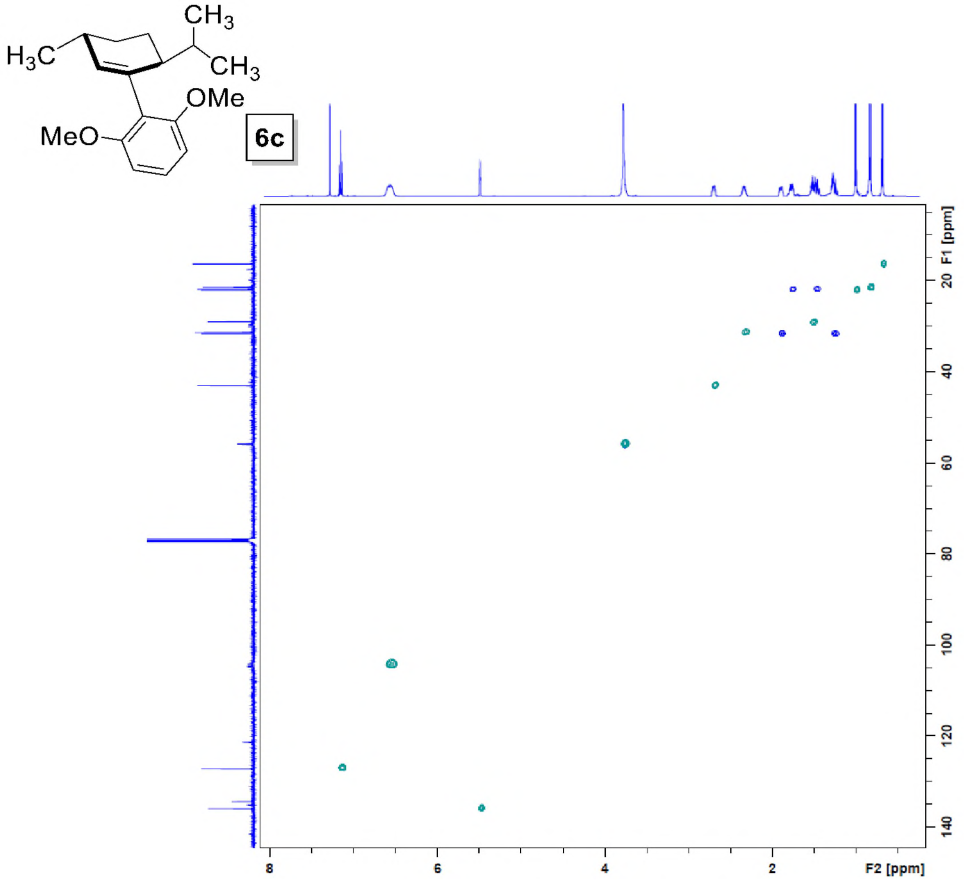

$H_3C$
$CH_3$
$CH_3$
OMe
MeO
6c
F1 [ppm]
F2 [ppm]
20
40
60
80
100
120
140
8
6
4
2

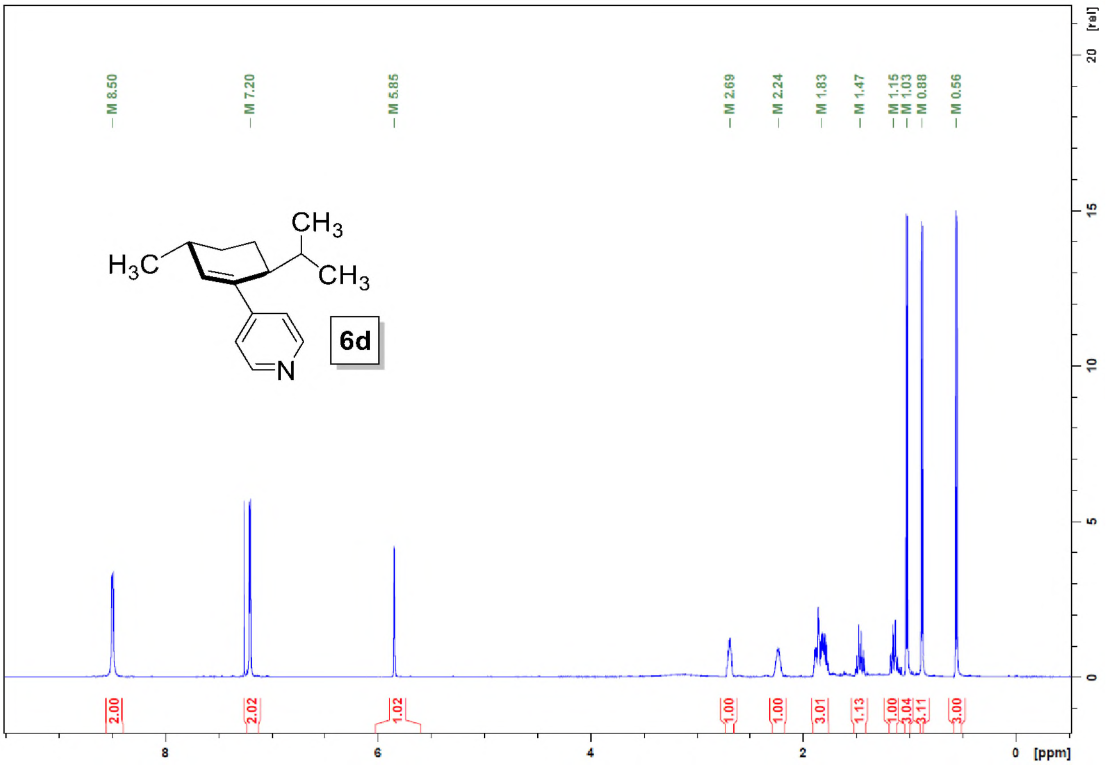
M 8.50
M 7.20
M 5.85
M 2.69
M 2.24
M 1.83
M 1.47
M 1.15
M 1.03
M 0.88
M 0.56
$H_3C$
$CH_3$
$CH_3$
N
6d
2.00
2.02
1.02
1.00
1.00
3.01
1.13
1.00
3.04
3.11
3.00
[ppm]
[rel]

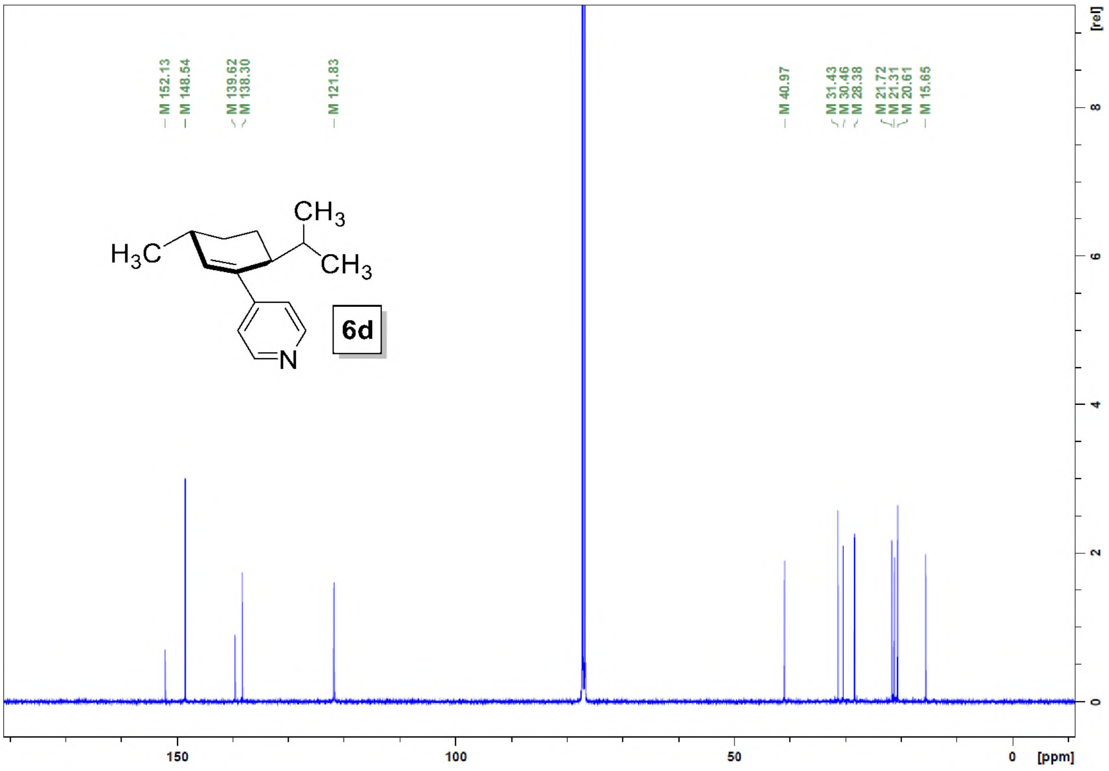
M 152.13
M 148.54
M 139.62
M 138.30
M 121.83
M 40.97
M 31.43
M 30.46
M 28.38
M 21.72
M 21.31
M 20.61
M 15.65
$H_3C$
$CH_3$
$CH_3$
N
6d
[ppm]
[rel]

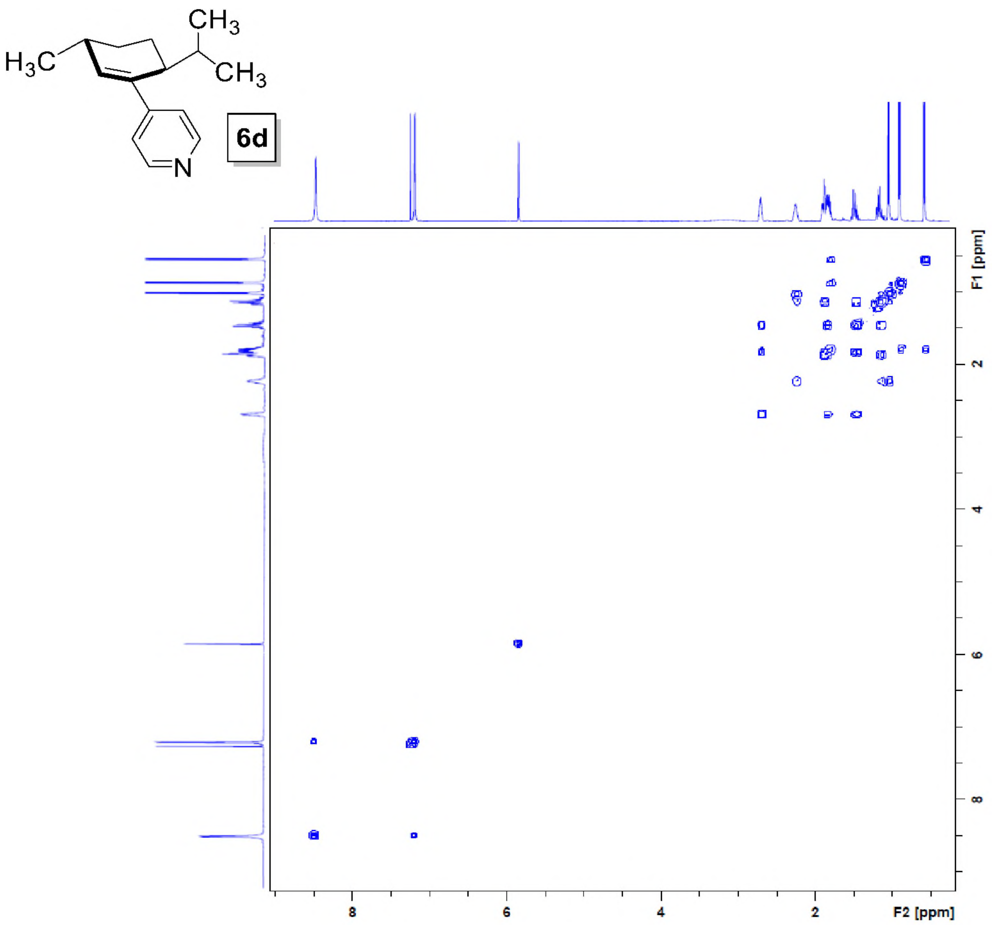
H3C
CH3
CH3
N
6d
F1 [ppm]
F2 [ppm]
2
4
6
8

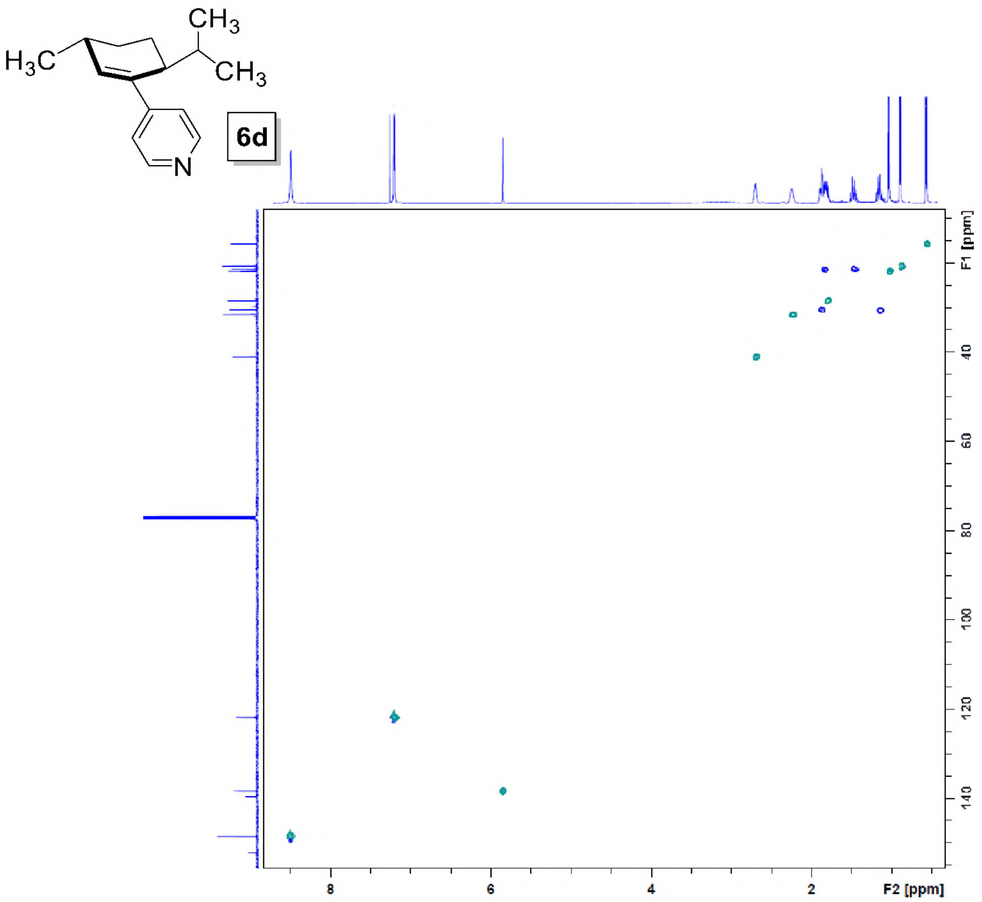
H3C
CH3
CH3
N
6d
F1 [ppm]
40
60
80
100
120
140
F2 [ppm]
8
6
4
2

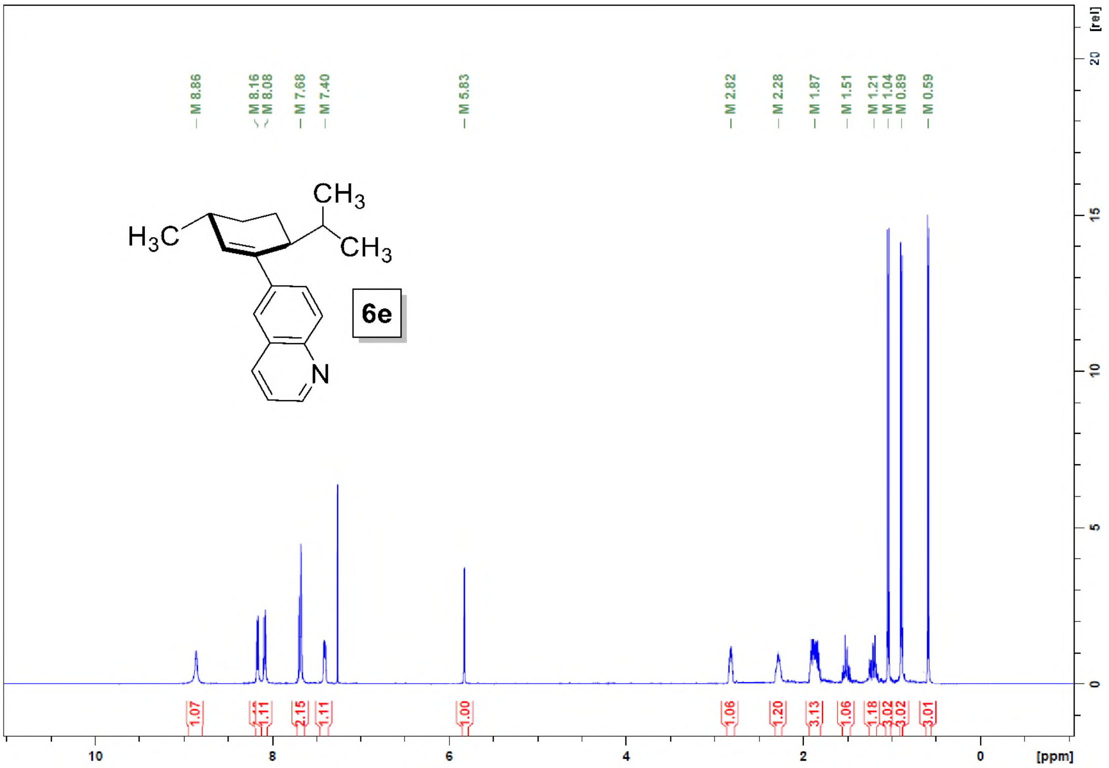
M 8.86
M 8.16
M 8.08
M 7.68
M 7.40
M 5.83
M 2.82
M 2.28
M 1.87
M 1.51
M 1.21
M 1.04
M 0.89
M 0.59
6e
[ppm]
[rel]

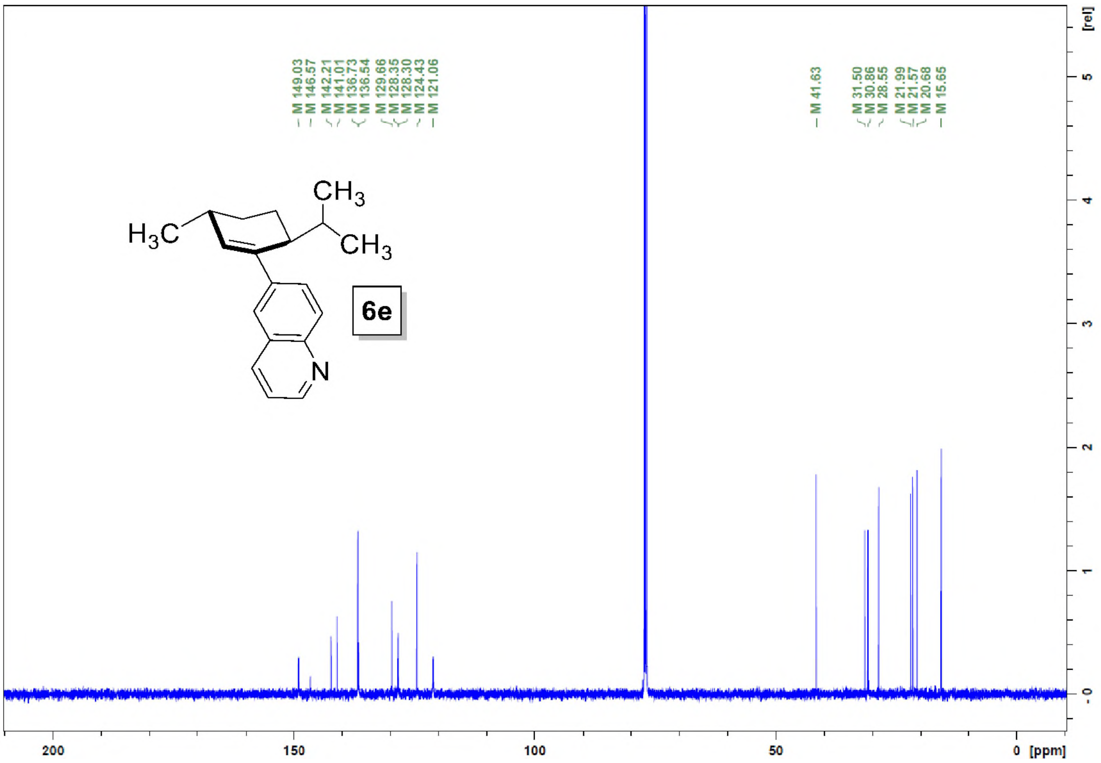
M 149.03
M 146.57
M 142.21
M 141.01
M 136.73
M 136.54
M 129.66
M 128.35
M 128.30
M 124.43
M 121.06
M 41.63
M 31.50
M 30.86
M 28.55
M 21.99
M 21.57
M 20.68
M 15.65
6e
[ppm]
[rel]

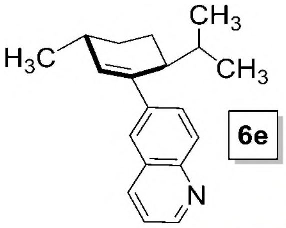

**6e**

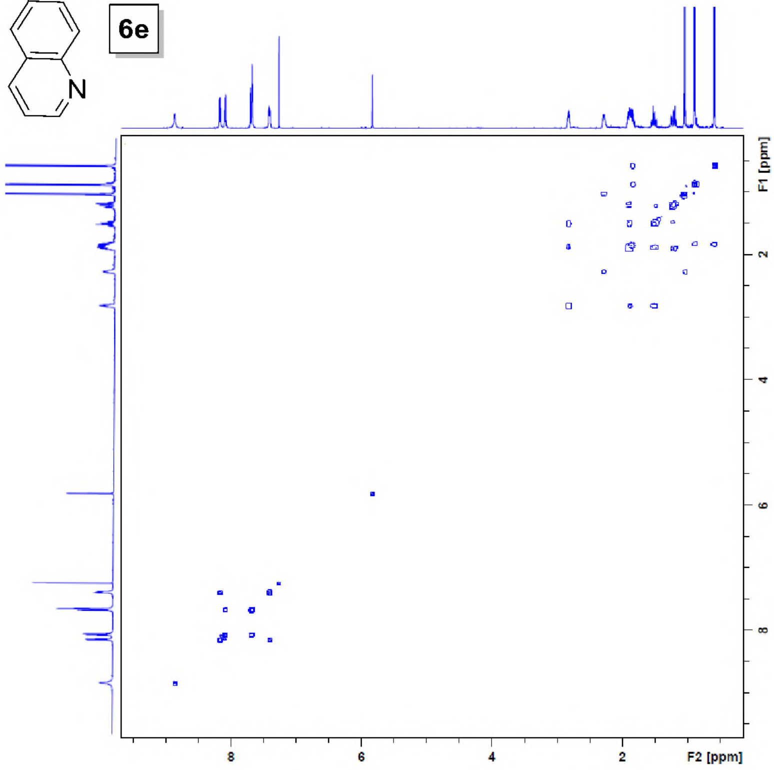


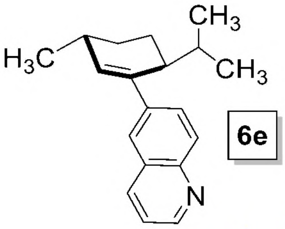

**6e**

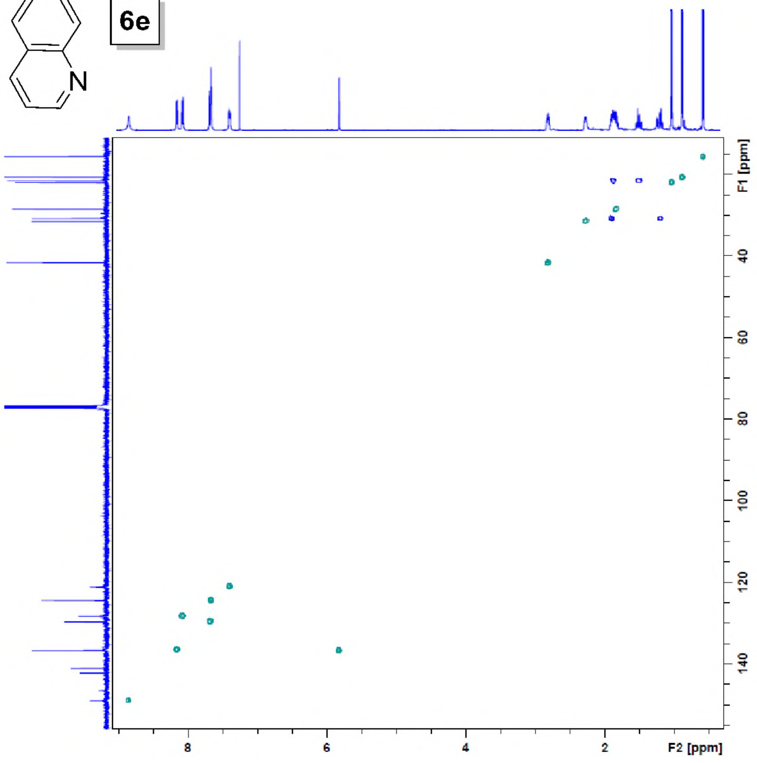

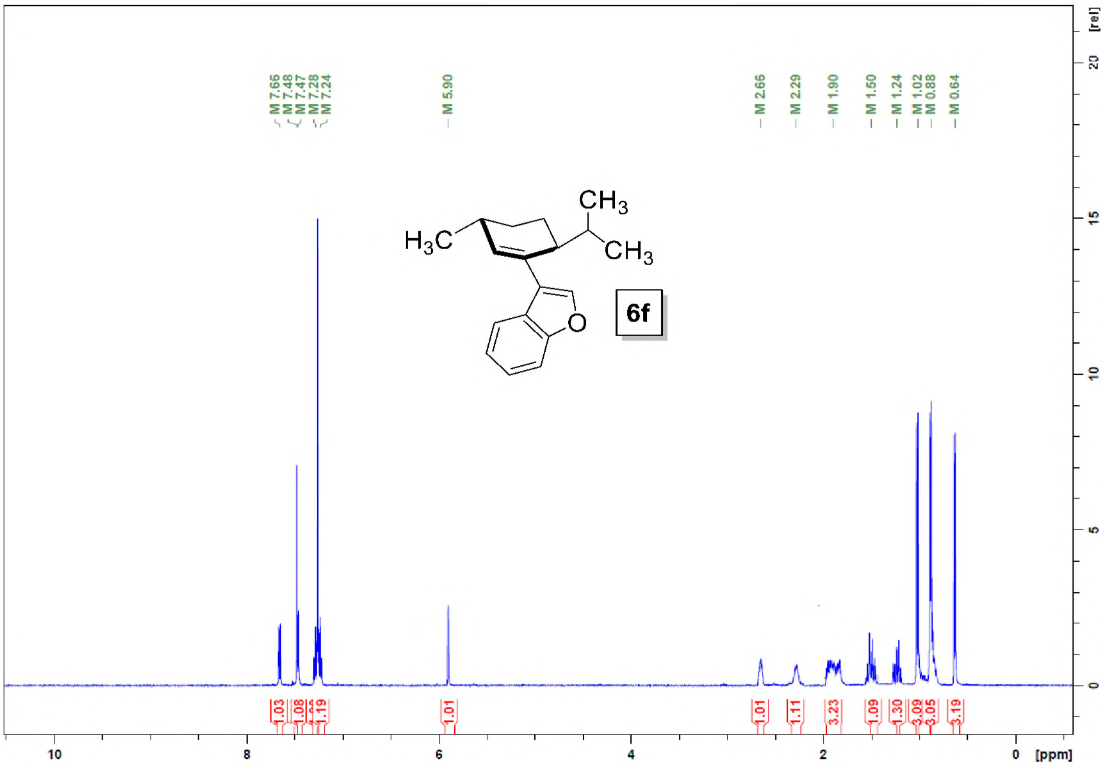
M 7.66
M 7.48
M 7.47
M 7.28
M 7.24
M 5.90
M 2.66
M 2.29
M 1.90
M 1.50
M 1.24
M 1.02
M 0.88
M 0.64
H3C
CH3
CH3
O
6f
1.03
1.08
1.19
1.01
1.01
1.11
3.23
1.09
1.30
3.09
3.05
3.19
10
8
6
4
2
0
[ppm]
[rel]
20
15
10
5
0

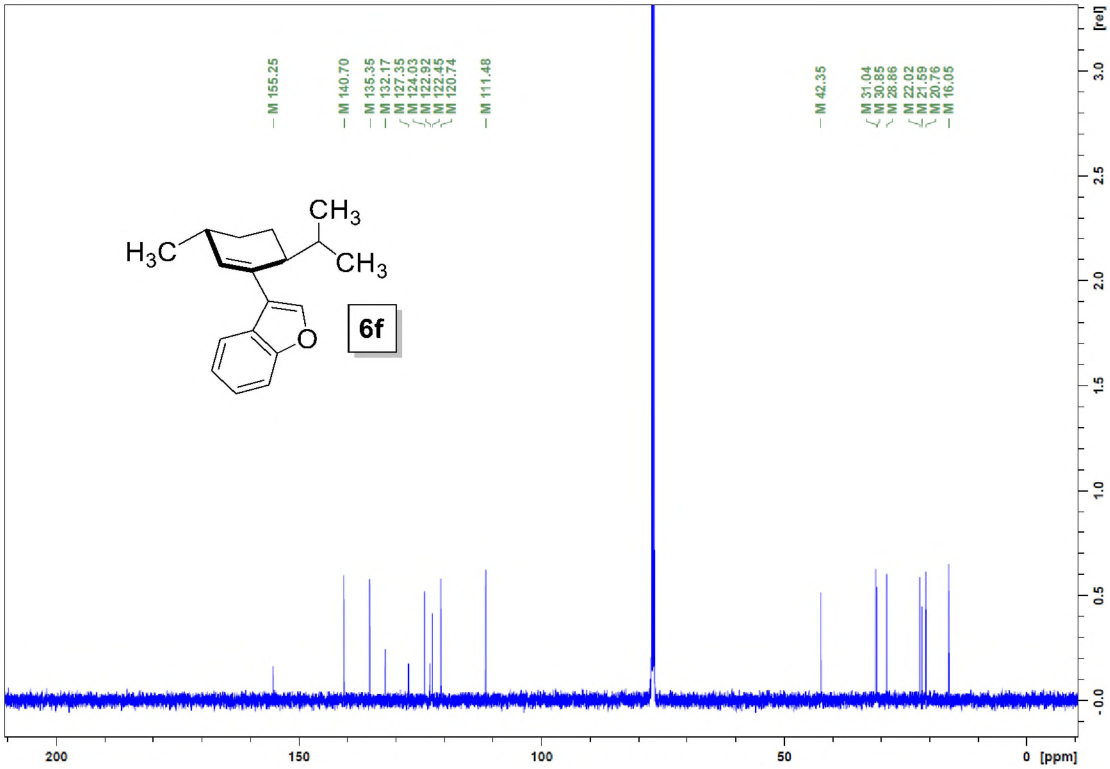
M 155.25
M 140.70
M 135.35
M 132.17
M 127.35
M 124.03
M 122.92
M 122.45
M 120.74
M 111.48
M 42.35
M 31.04
M 30.85
M 28.86
M 22.02
M 21.59
M 20.76
M 16.05
H3C
CH3
CH3
O
6f
200
150
100
50
0
[ppm]
[rel]
3.0
2.5
2.0
1.5
1.0
0.5
-0.0

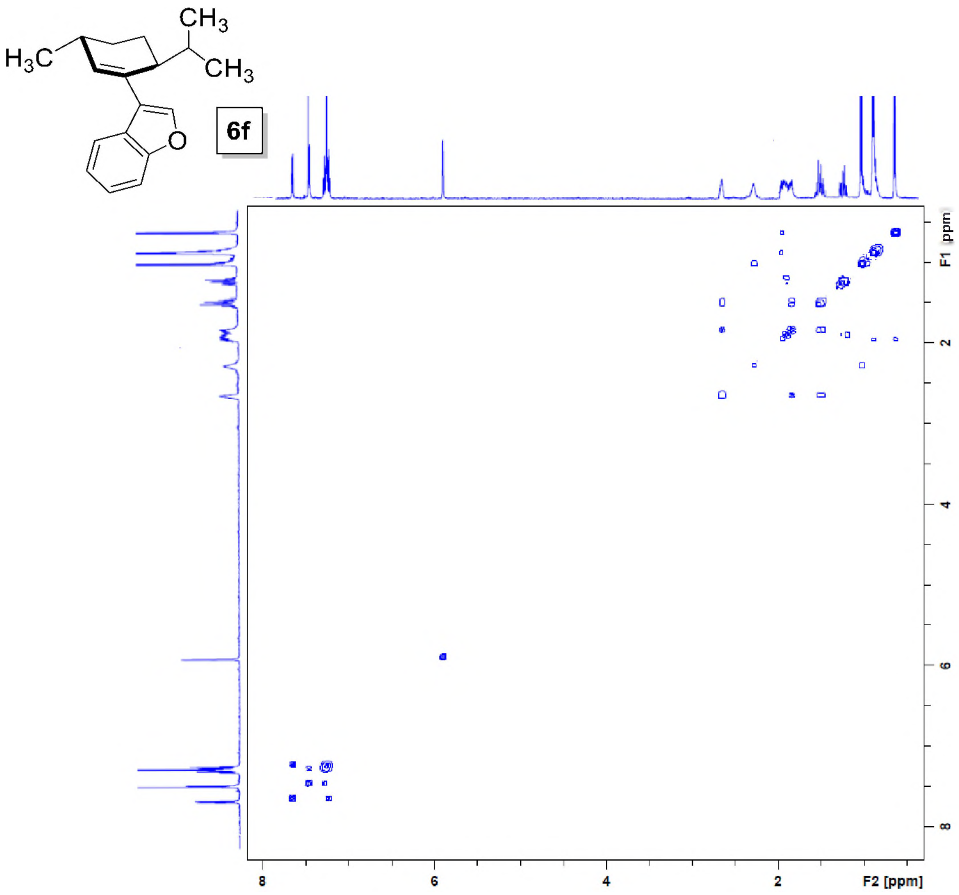


6f

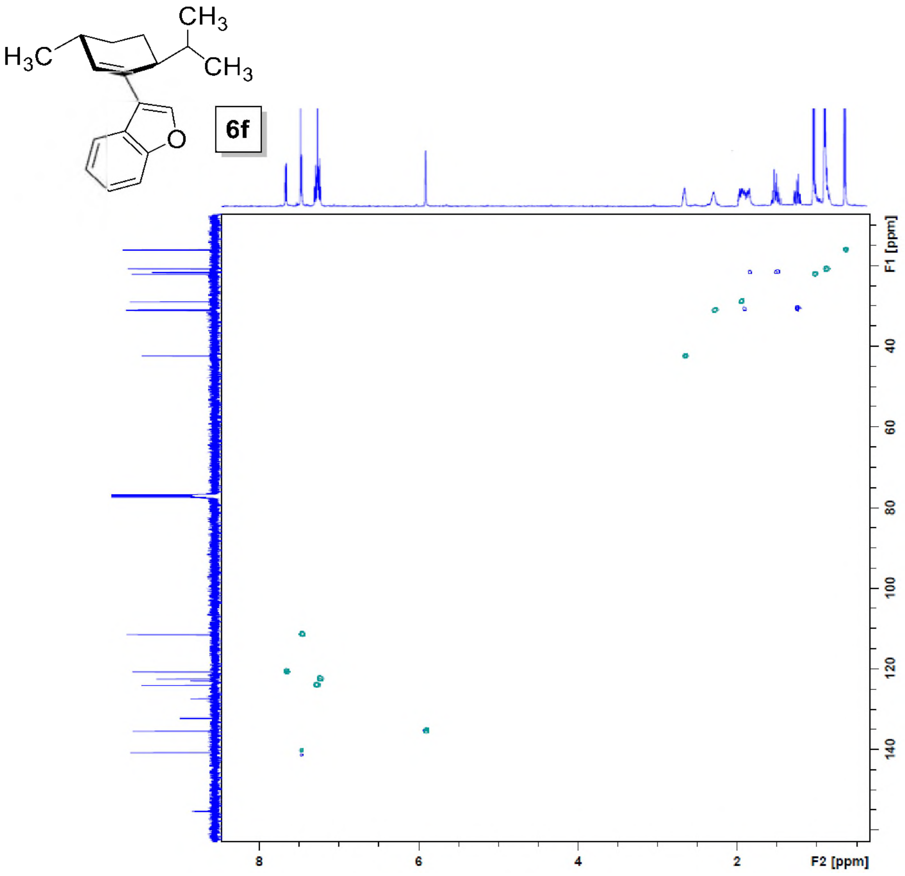


6f

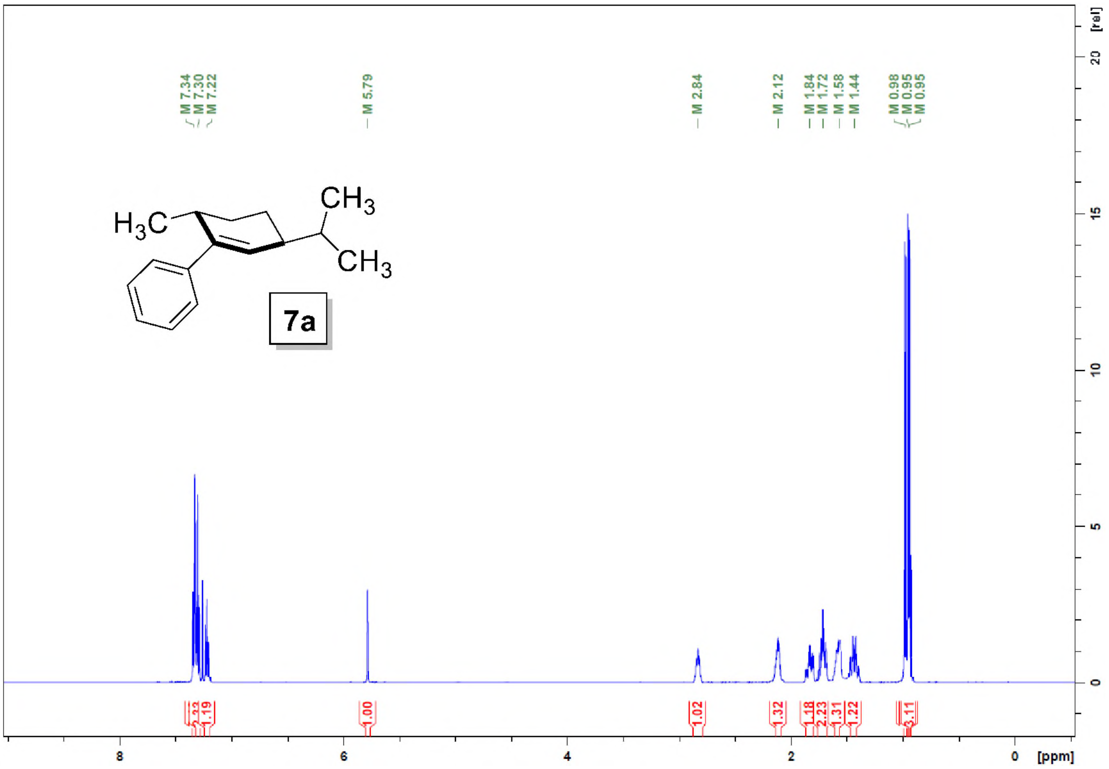
M 7.34
M 7.30
M 7.22
M 5.79
M 2.84
M 2.12
M 1.84
M 1.72
M 1.58
M 1.44
M 0.98
M 0.95
M 0.95
$H_3C$
$CH_3$
$CH_3$
7a
1.19
1.00
1.02
1.32
1.18
2.23
1.31
1.22
3.11
[rel]
[ppm]

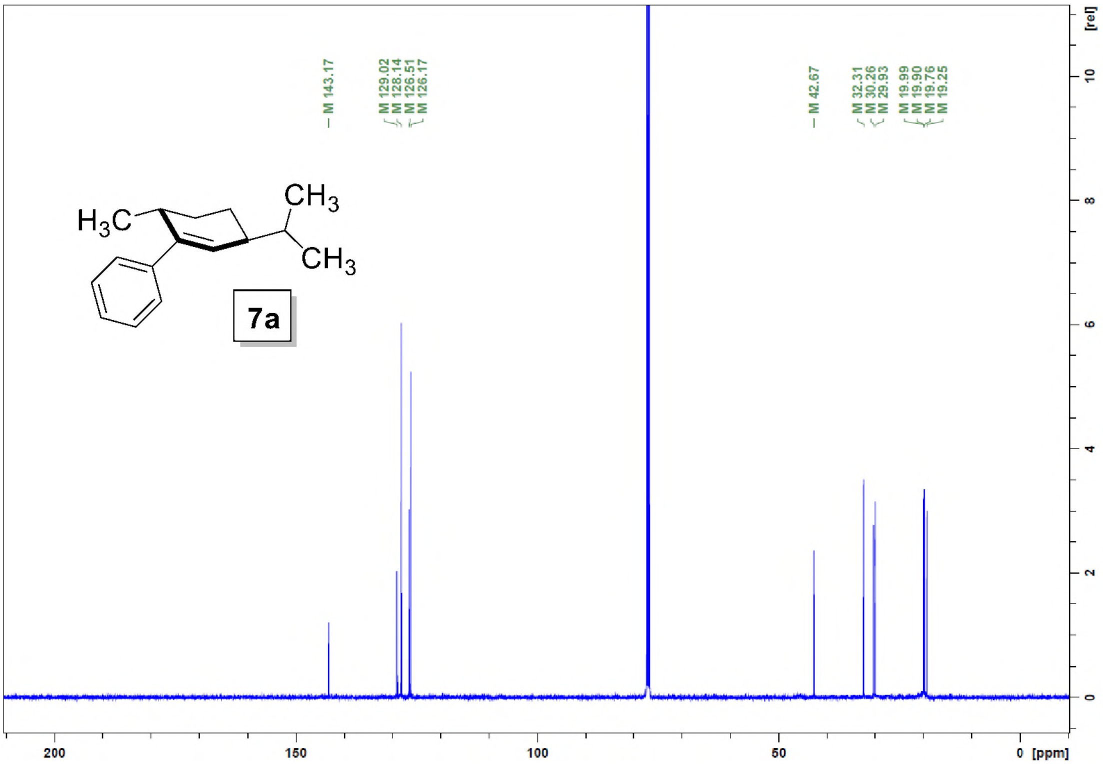
M 143.17
M 129.02
M 128.14
M 126.51
M 126.17
M 42.67
M 32.31
M 30.26
M 29.93
M 19.99
M 19.90
M 19.76
M 19.25
$H_3C$
$CH_3$
$CH_3$
7a
[rel]
[ppm]

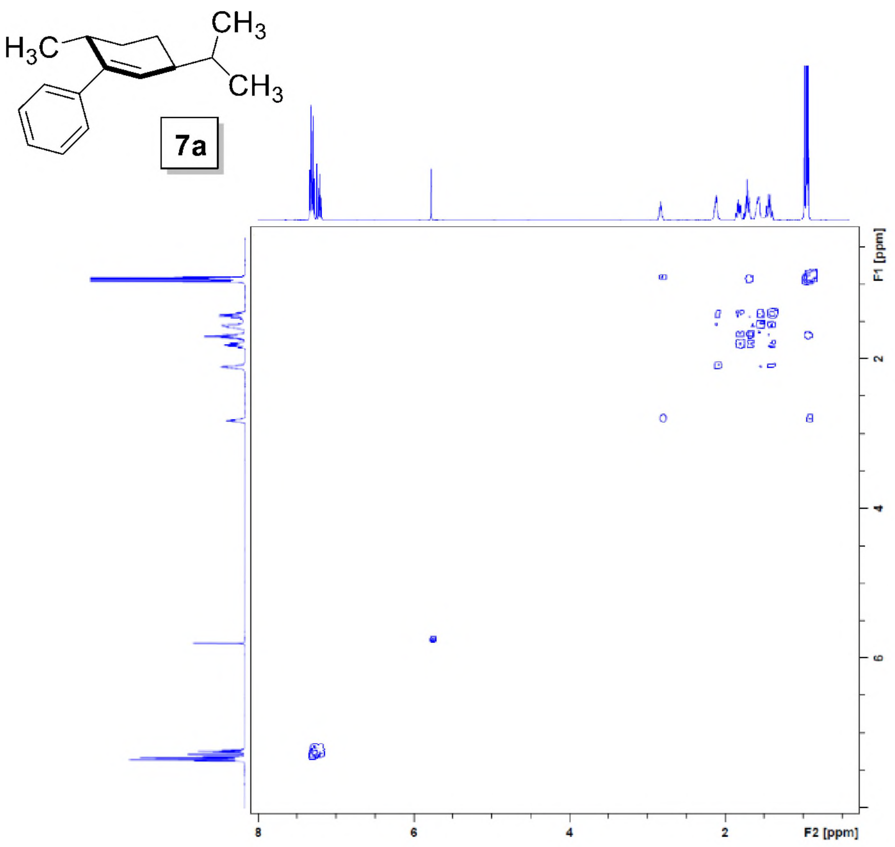
CH3
H3C
CH3
7a
F1 [ppm]
F2 [ppm]

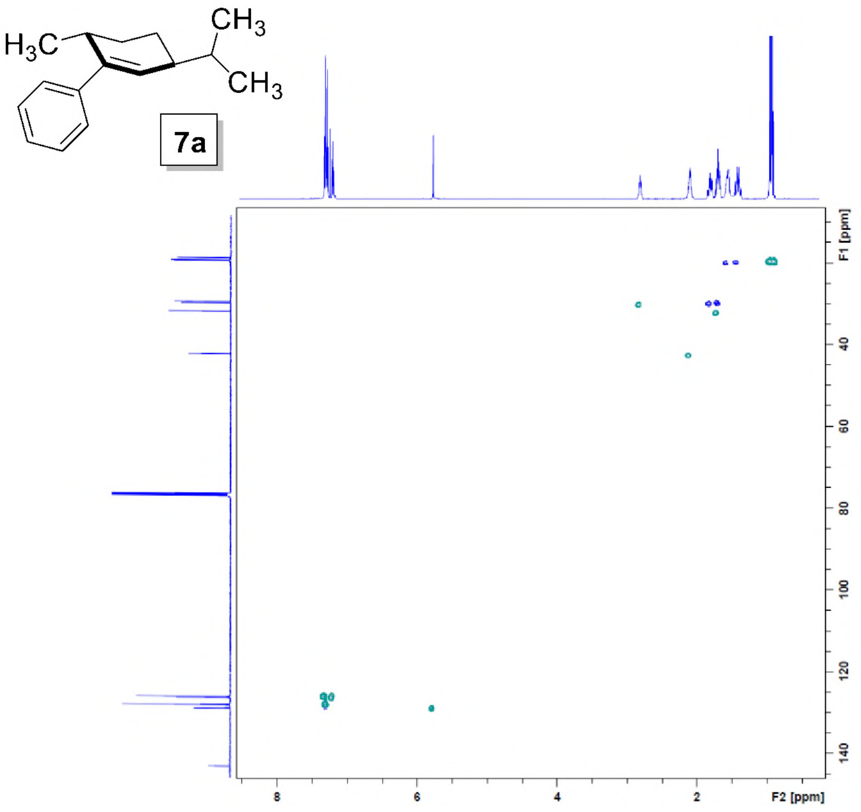
CH3
H3C
CH3
7a
F1 [ppm]
F2 [ppm]

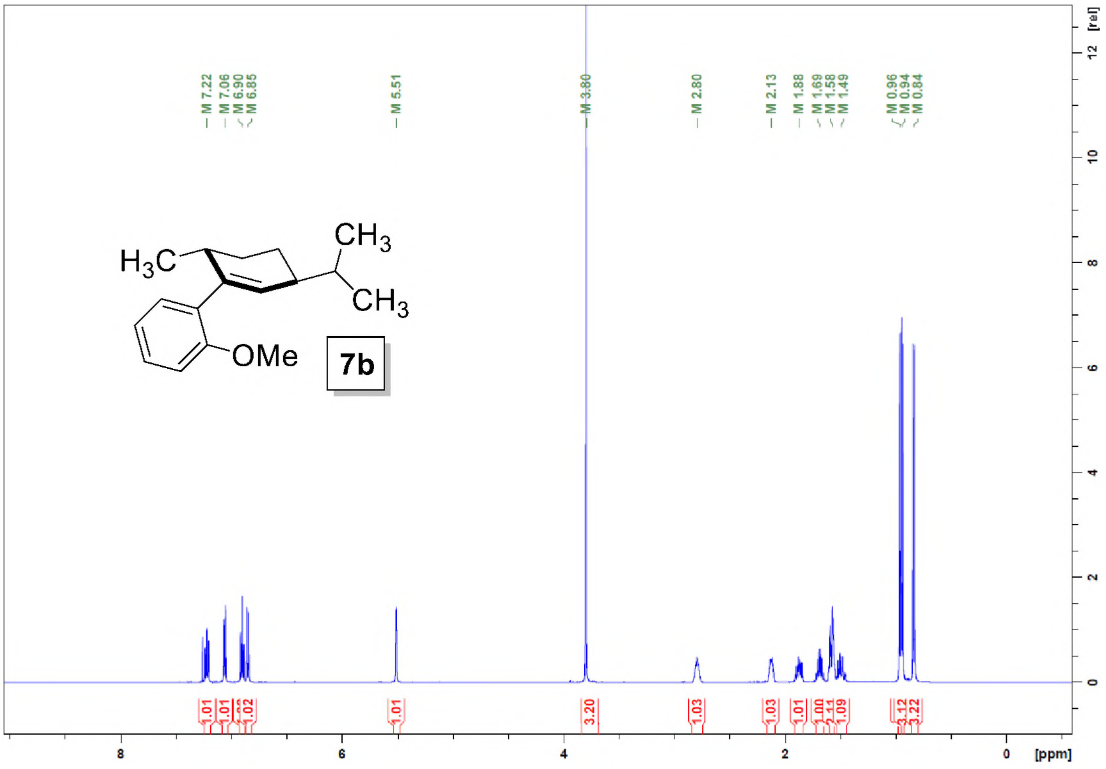
M 7.22
M 7.06
M 6.90
M 6.85
M 5.51
M 3.80
M 2.80
M 2.13
M 1.88
M 1.69
M 1.58
M 1.49
M 0.96
M 0.94
M 0.84
1.01
1.01
1.02
1.01
3.20
1.03
1.03
1.01
1.00
2.11
1.09
3.12
3.22
H3C
CH3
CH3
OMe
7b
[rel]
[ppm]

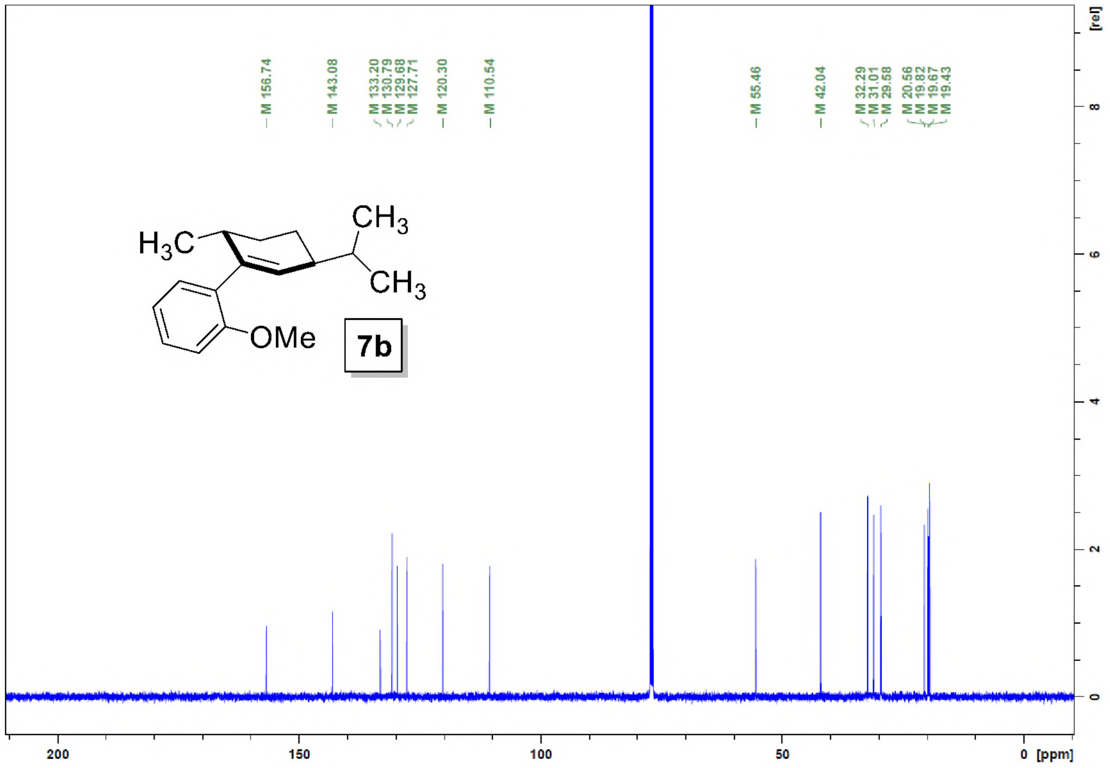
M 156.74
M 143.08
M 133.20
M 130.79
M 129.68
M 127.71
M 120.30
M 110.54
M 55.46
M 42.04
M 32.29
M 31.01
M 29.58
M 20.56
M 19.82
M 19.67
M 19.43
H3C
CH3
CH3
OMe
7b
[rel]
[ppm]

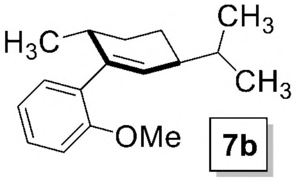
H3C
CH3
CH3
OMe

**7b**

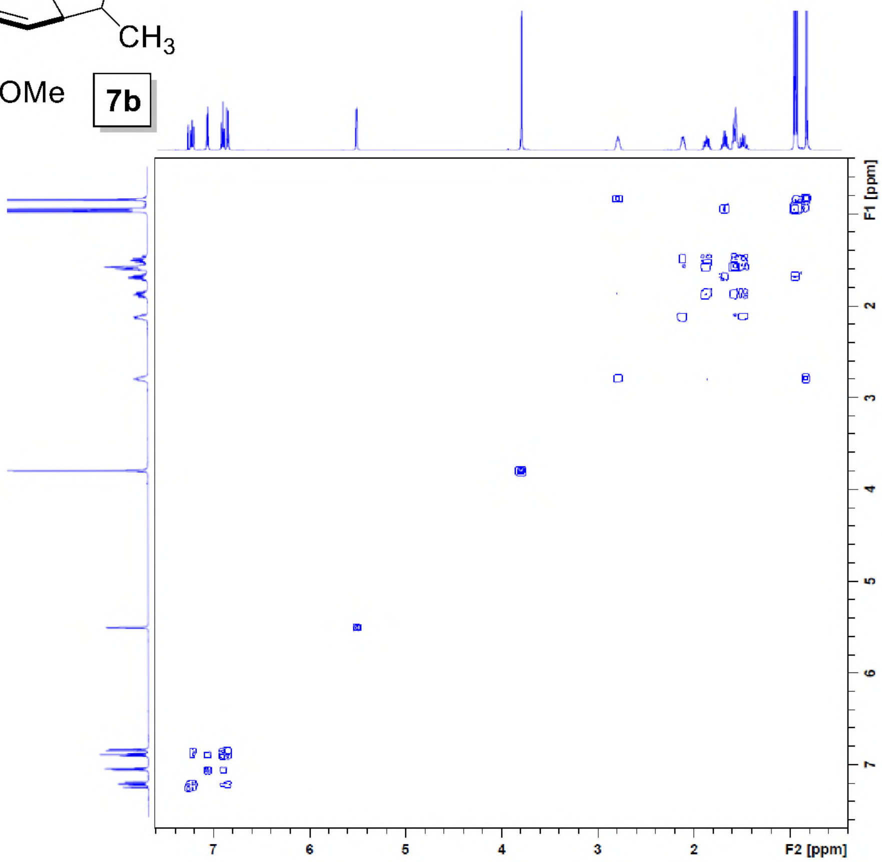
F1 [ppm]
1
2
3
4
5
6
7
F2 [ppm]
7
6
5
4
3
2

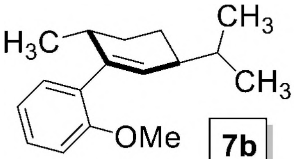
H3C
CH3
CH3
OMe

**7b**

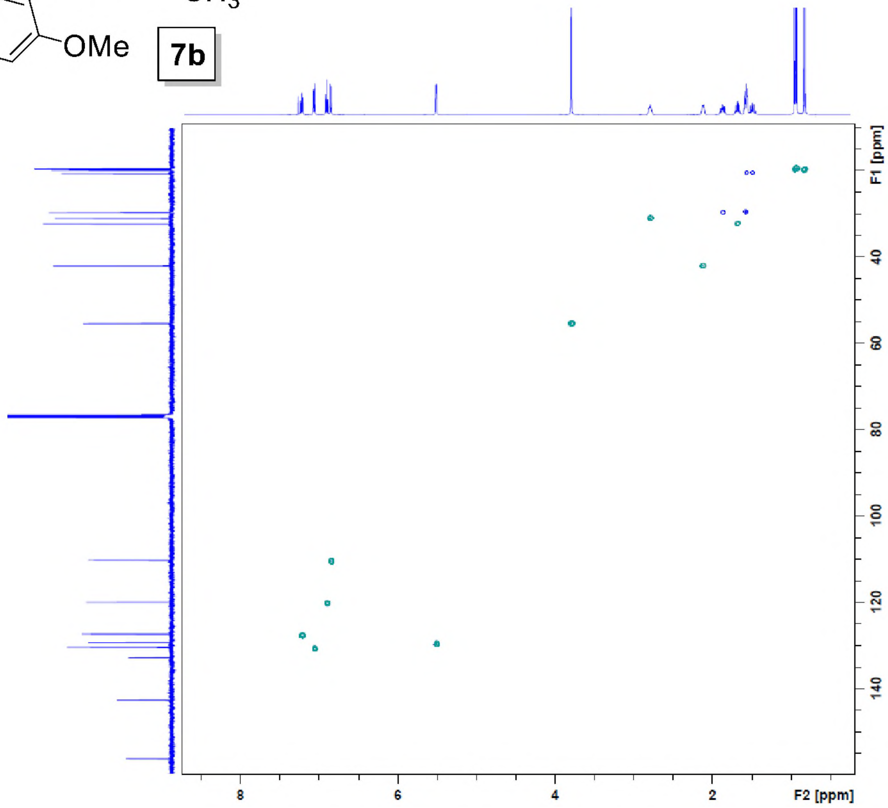
F1 [ppm]
40
60
80
100
120
140
F2 [ppm]
8
6
4
2

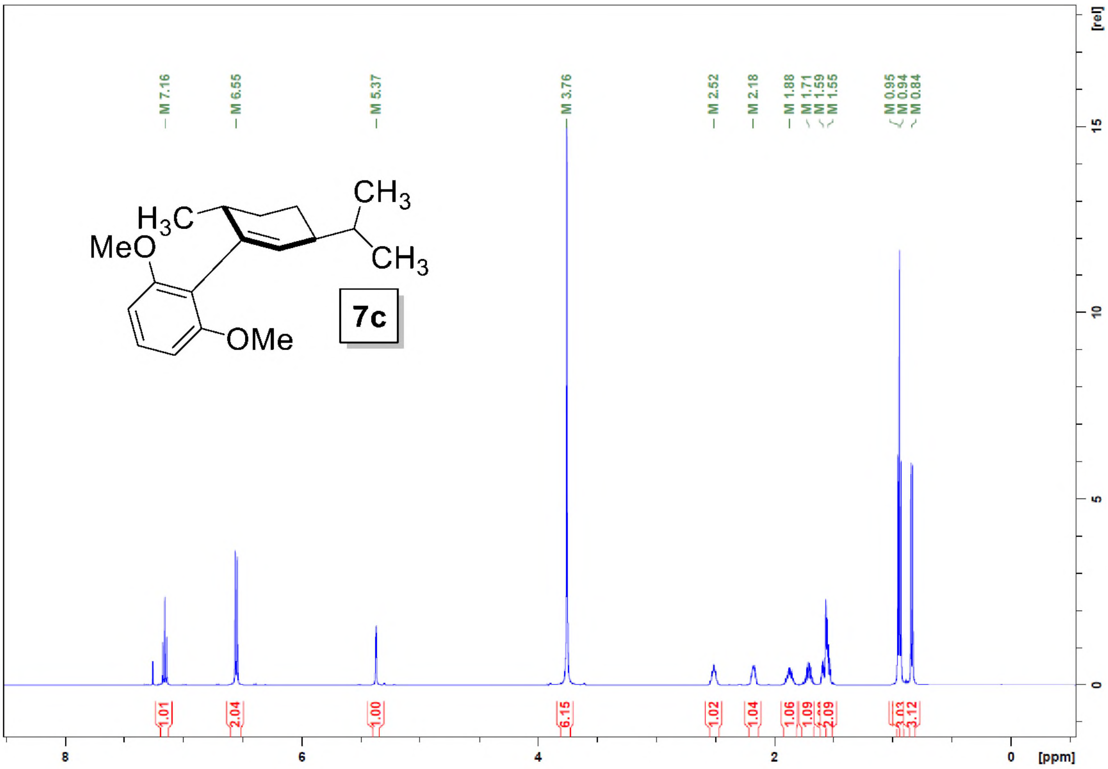

M 7.16
M 6.55
M 5.37
M 3.76
M 2.52
M 2.18
M 1.88
M 1.71
M 1.59
M 1.55
M 0.95
M 0.94
M 0.84
$H_3C$
$CH_3$
$CH_3$
MeO
OMe
7c
1.01
2.04
1.00
6.15
1.02
1.04
1.06
1.09
2.09
3.03
3.12
[rel]
[ppm]


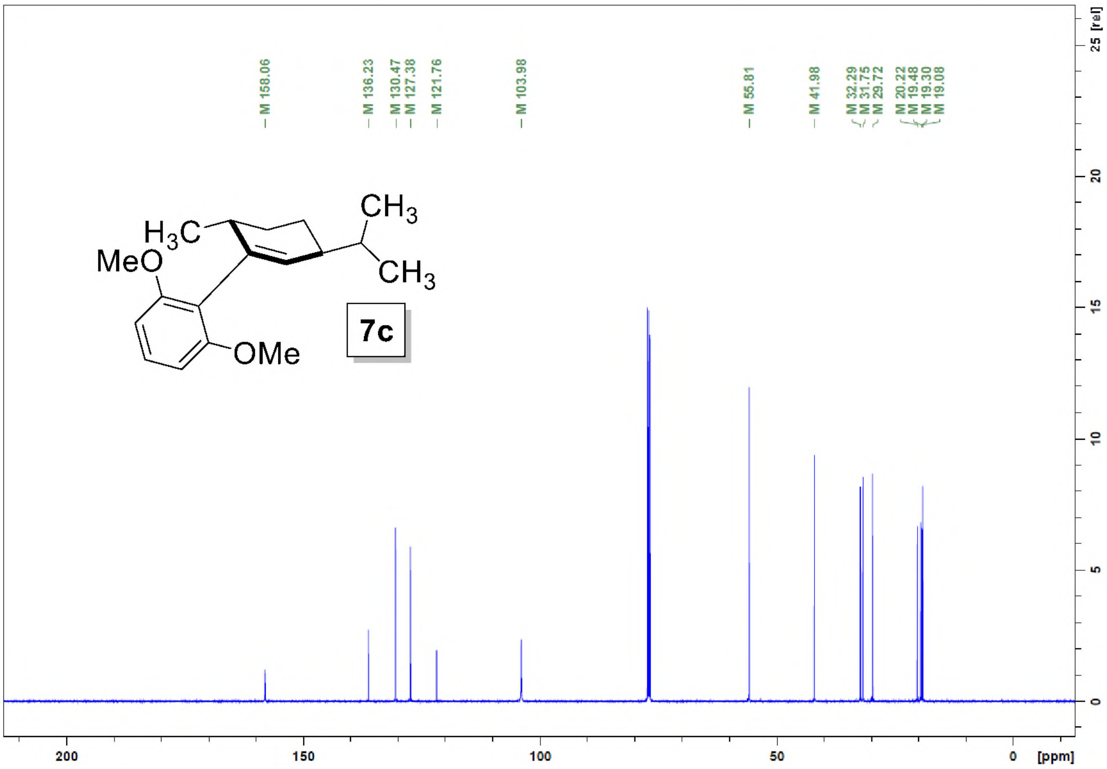

M 158.06
M 136.23
M 130.47
M 127.38
M 121.76
M 103.98
M 55.81
M 41.98
M 32.29
M 31.75
M 29.72
M 20.22
M 19.48
M 19.30
M 19.08
$H_3C$
$CH_3$
$CH_3$
MeO
OMe
7c
[rel]
[ppm]

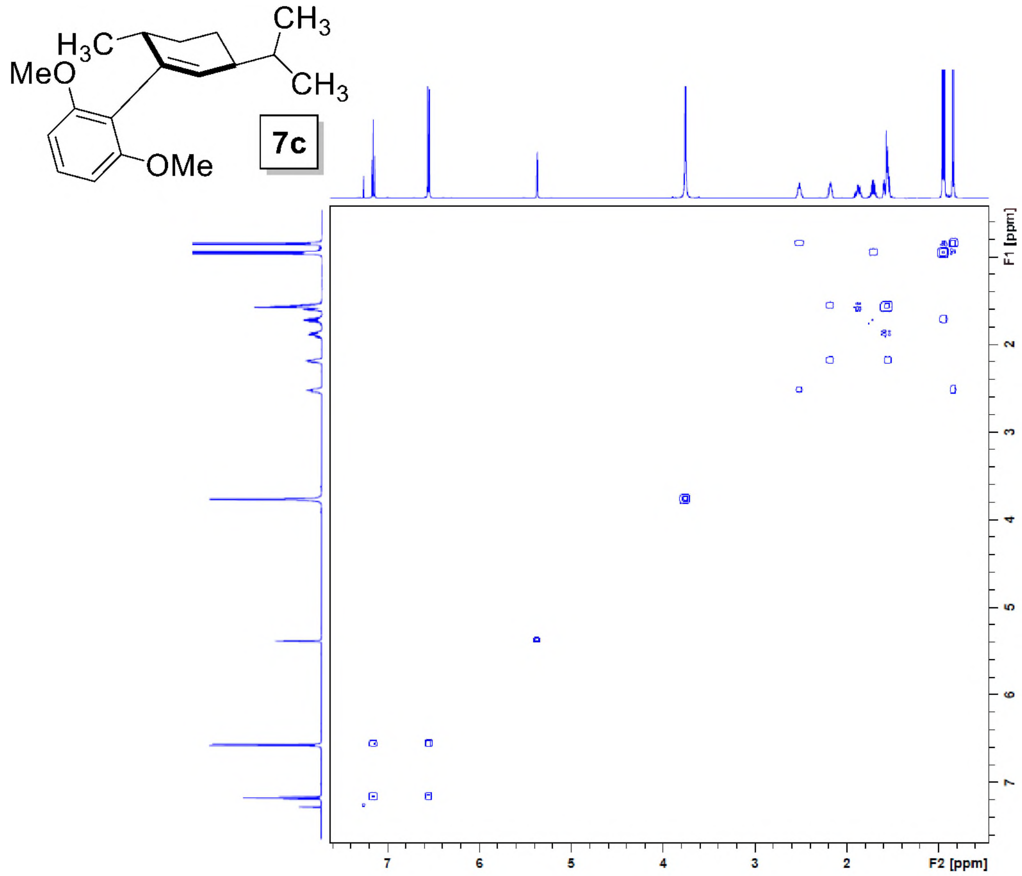
H3C
MeO
CH3
CH3
OMe
7c
F1 [ppm]
F2 [ppm]

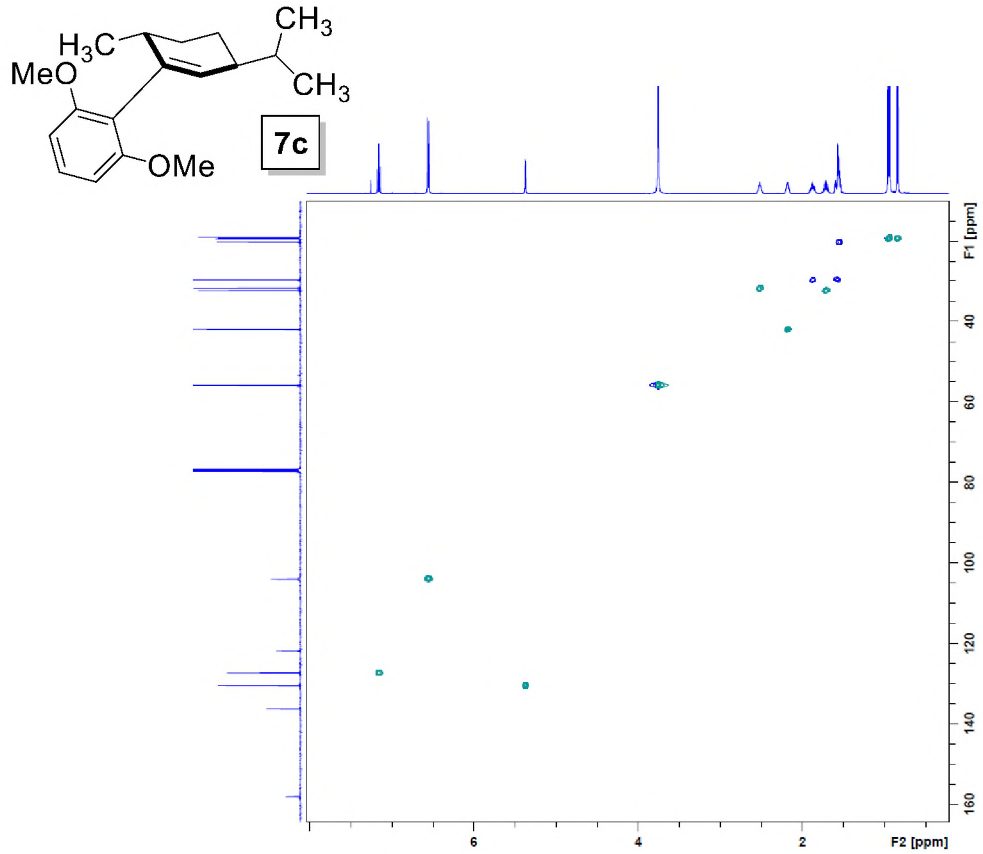
H3C
MeO
CH3
CH3
OMe
7c
F1 [ppm]
F2 [ppm]

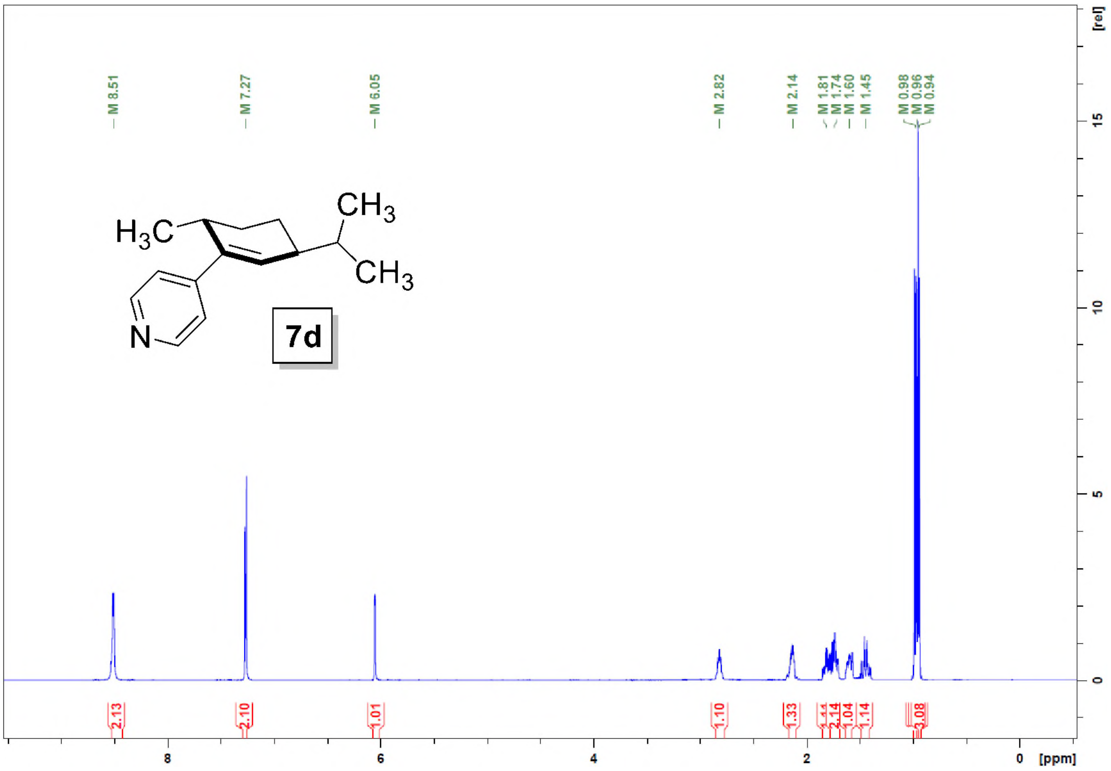
M 8.51
M 7.27
M 6.05
M 2.82
M 2.14
M 1.81
M 1.74
M 1.60
M 1.45
M 0.98
M 0.96
M 0.94
H3C
CH3
CH3
N
7d
2.13
2.10
1.01
1.10
1.33
2.14
1.04
1.14
3.08
[rel]
[ppm]

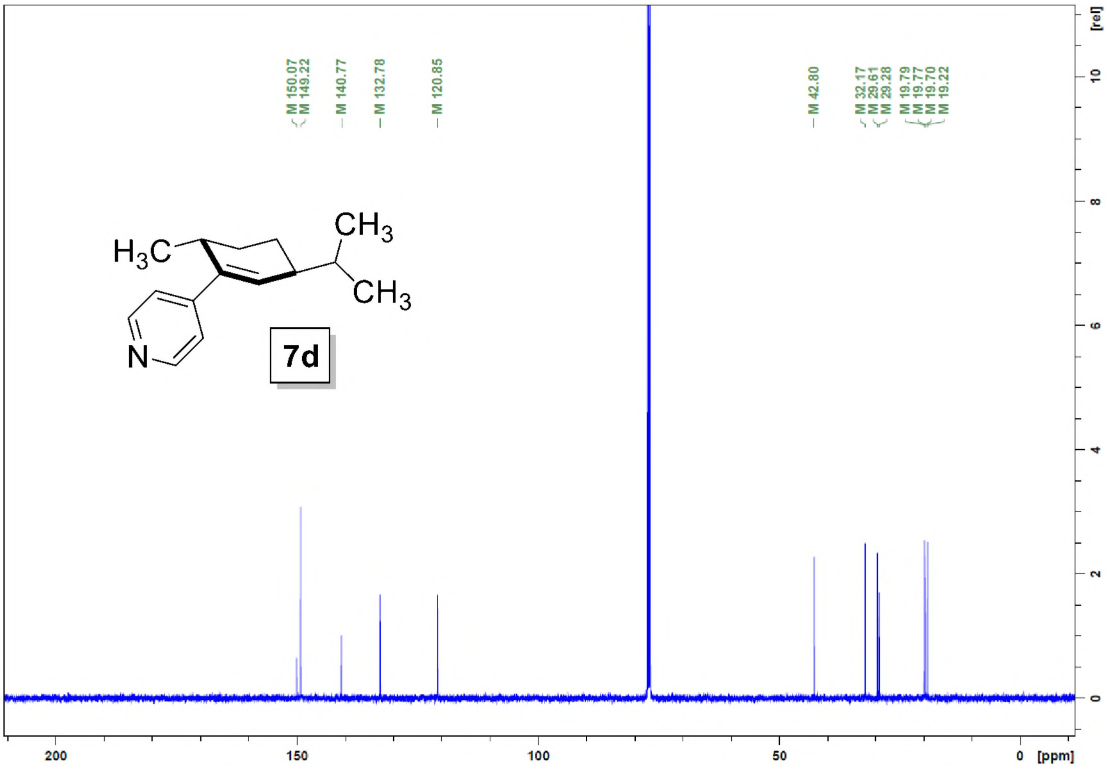
M 150.07
M 149.22
M 140.77
M 132.78
M 120.85
M 42.80
M 32.17
M 29.61
M 29.28
M 19.79
M 19.77
M 19.70
M 19.22
H3C
CH3
CH3
N
7d
[rel]
[ppm]

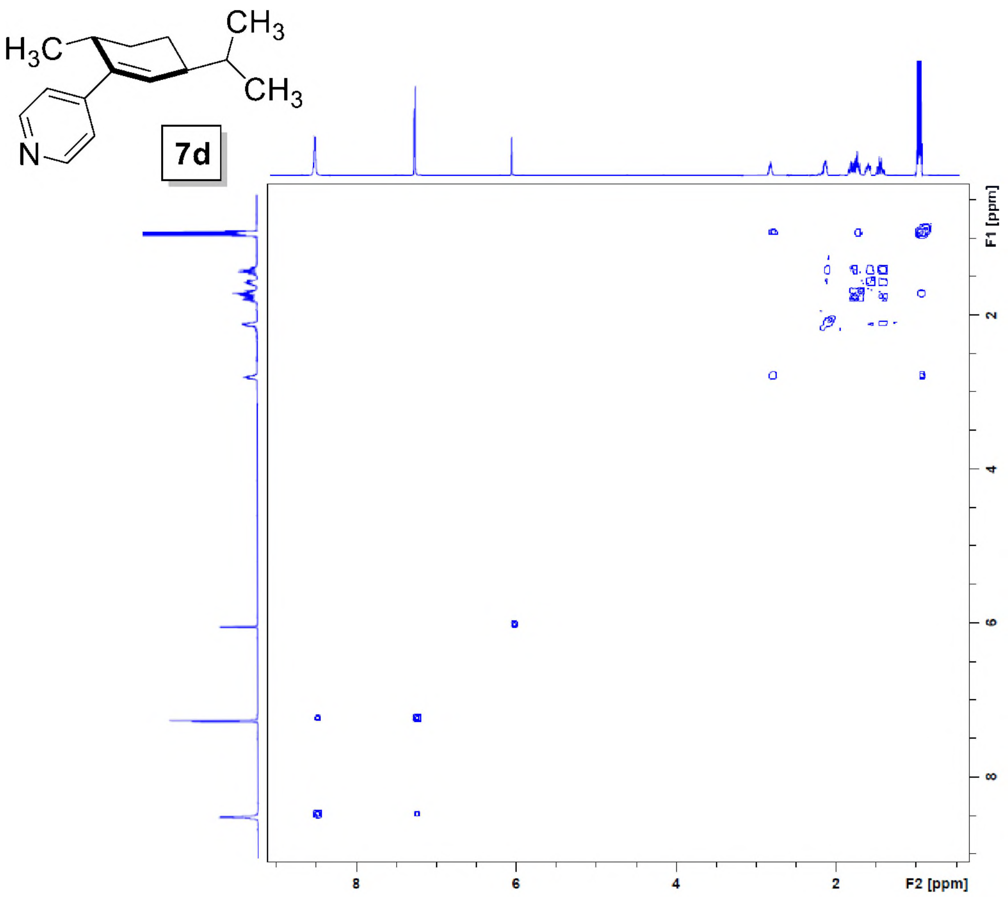
7d
F1 [ppm]
F2 [ppm]
2
4
6
8

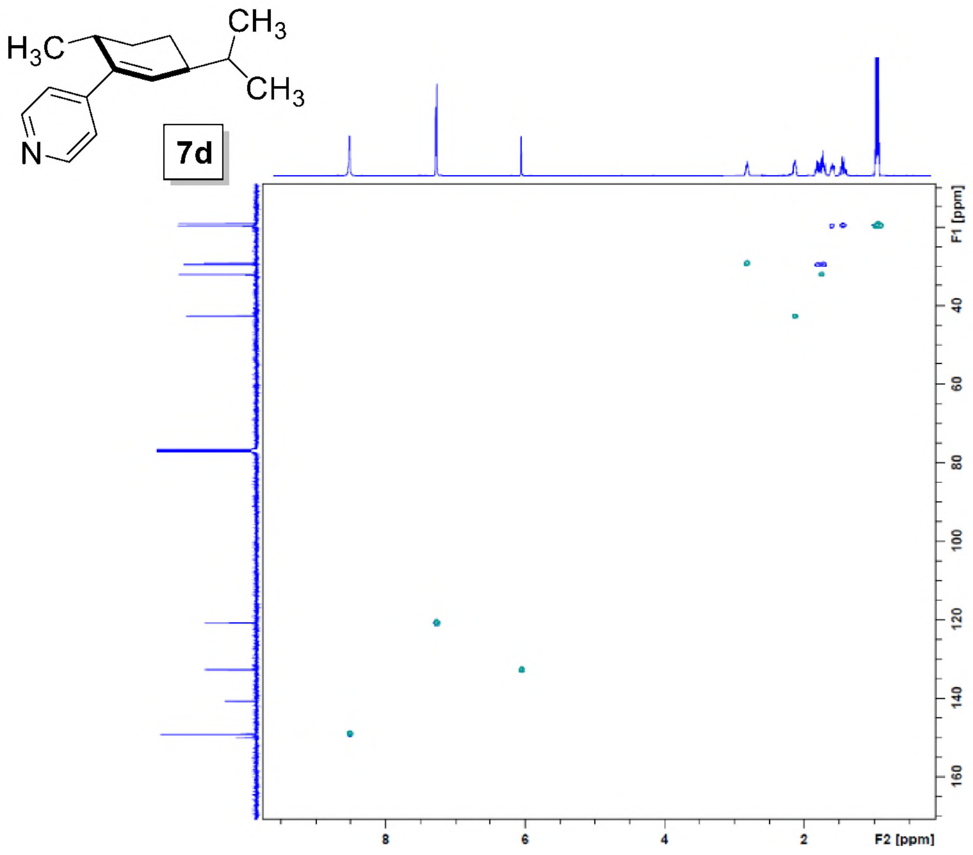
7d
F1 [ppm]
40
60
80
100
120
140
160
F2 [ppm]
8
6
4
2

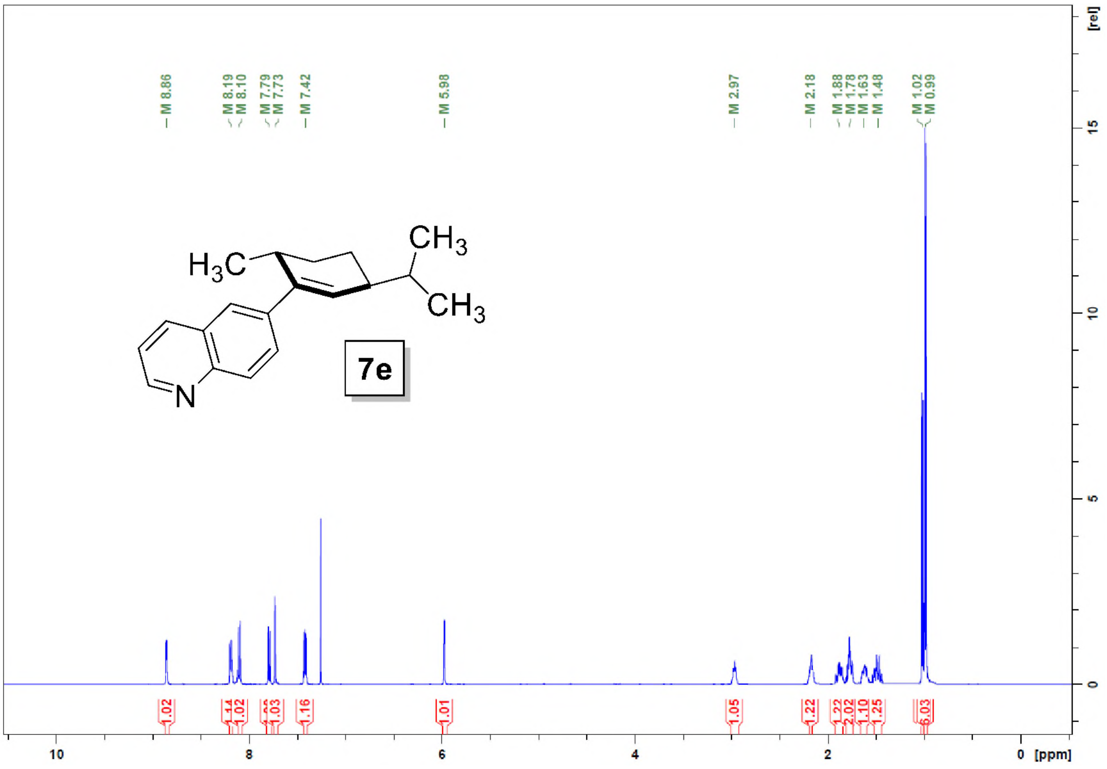
M 8.86
M 8.19
M 8.10
M 7.79
M 7.73
M 7.42
M 5.98
M 2.97
M 2.18
M 1.88
M 1.78
M 1.63
M 1.48
M 1.02
M 0.99
7e
[rel]
[ppm]

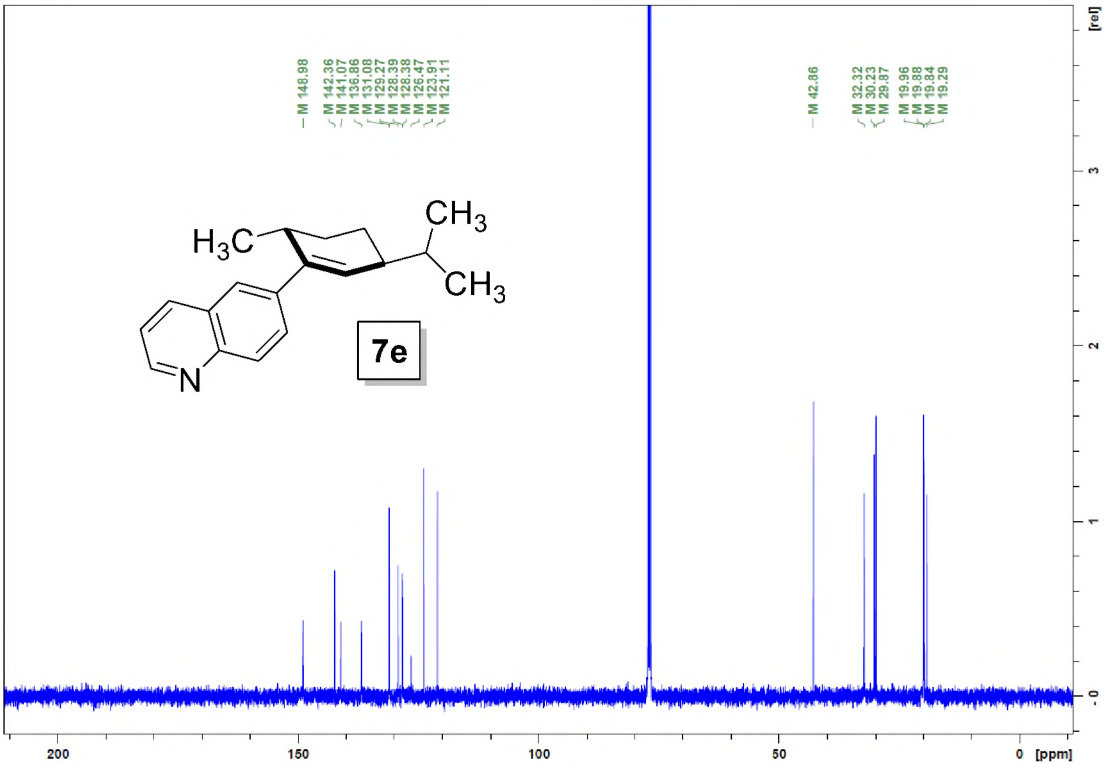
M 148.98
M 142.36
M 141.07
M 136.86
M 131.08
M 129.27
M 128.39
M 128.38
M 126.47
M 123.91
M 121.11
M 42.86
M 32.32
M 30.23
M 29.87
M 19.96
M 19.88
M 19.84
M 19.29
7e
[rel]
[ppm]

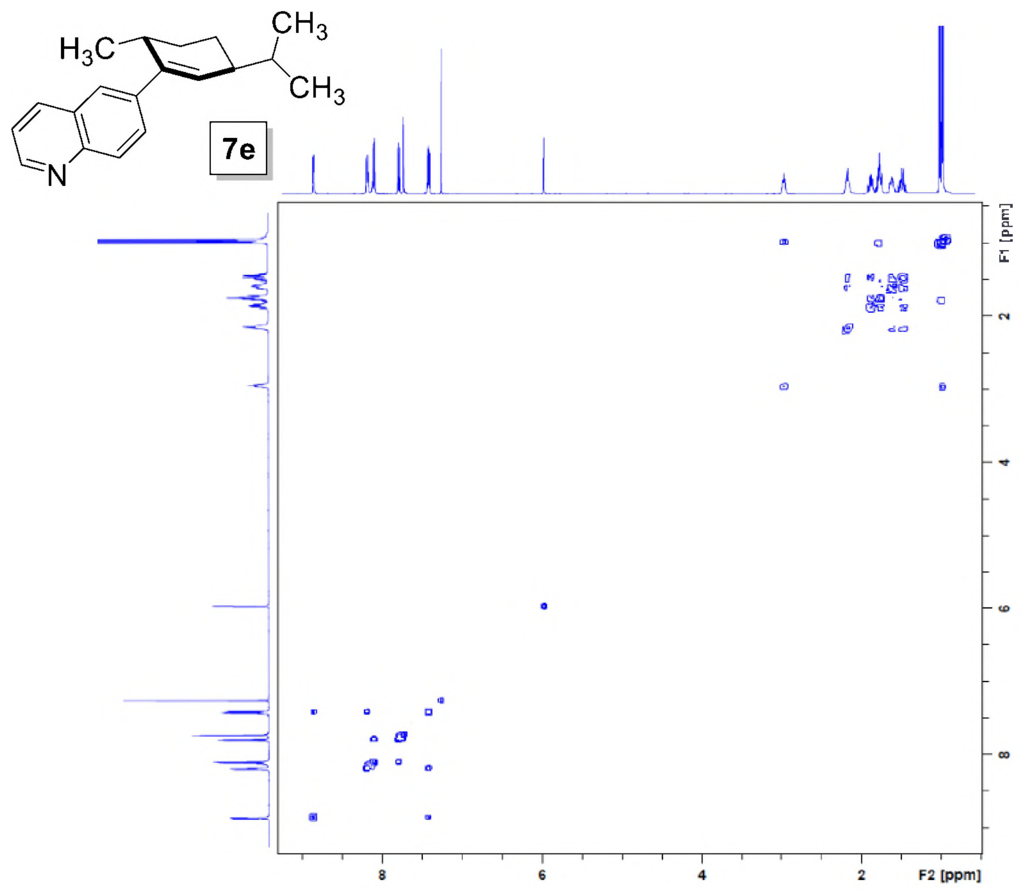
H3C
CH3
CH3
N
7e
F1 [ppm]
F2 [ppm]
8
6
4
2

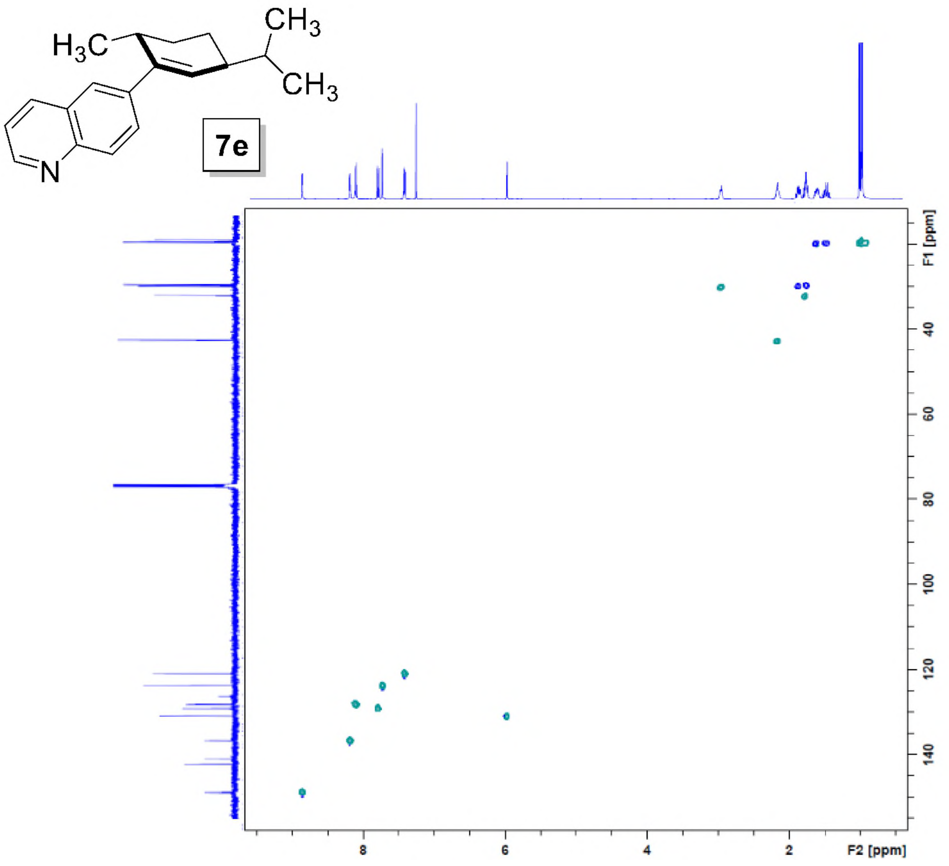
H3C
CH3
CH3
N
7e
F1 [ppm]
40
60
80
100
120
140
F2 [ppm]
8
6
4
2

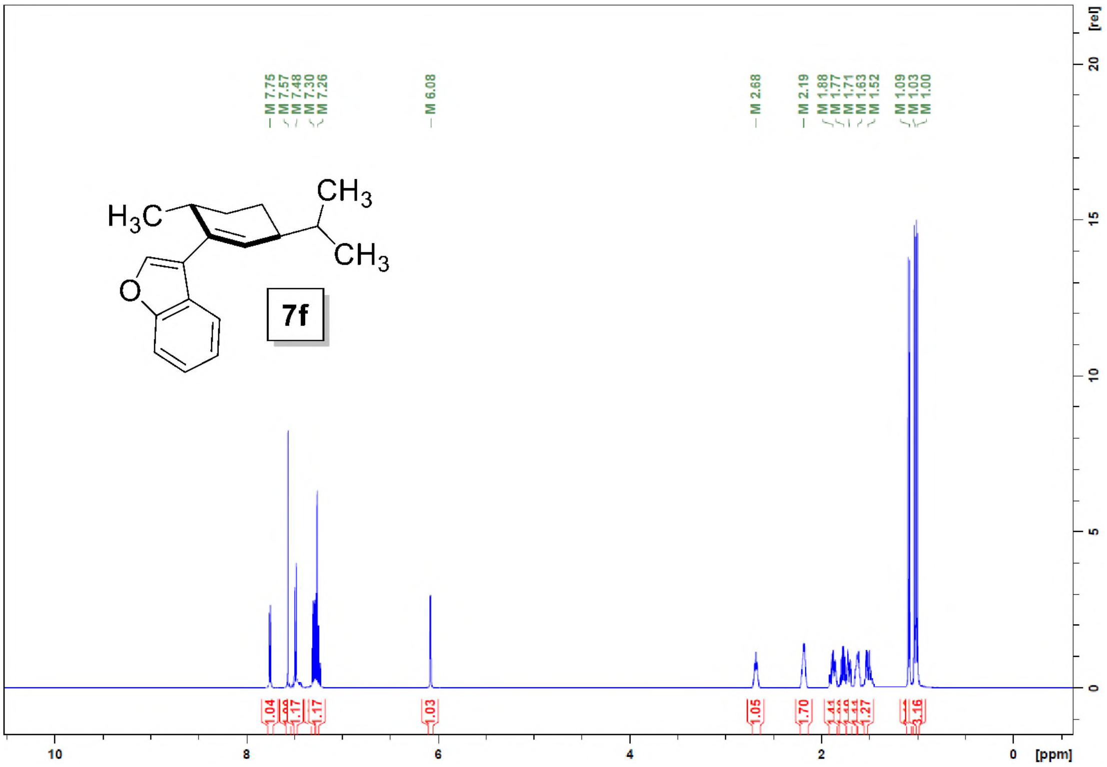
M 7.75
M 7.57
M 7.48
M 7.30
M 7.26
M 6.08
M 2.68
M 2.19
M 1.88
M 1.77
M 1.71
M 1.63
M 1.52
M 1.09
M 1.03
M 1.00
$H_3C$
$CH_3$
$CH_3$
O
7f
1.04
1.17
1.17
1.03
1.05
1.70
1.27
3.16
[rel]
[ppm]

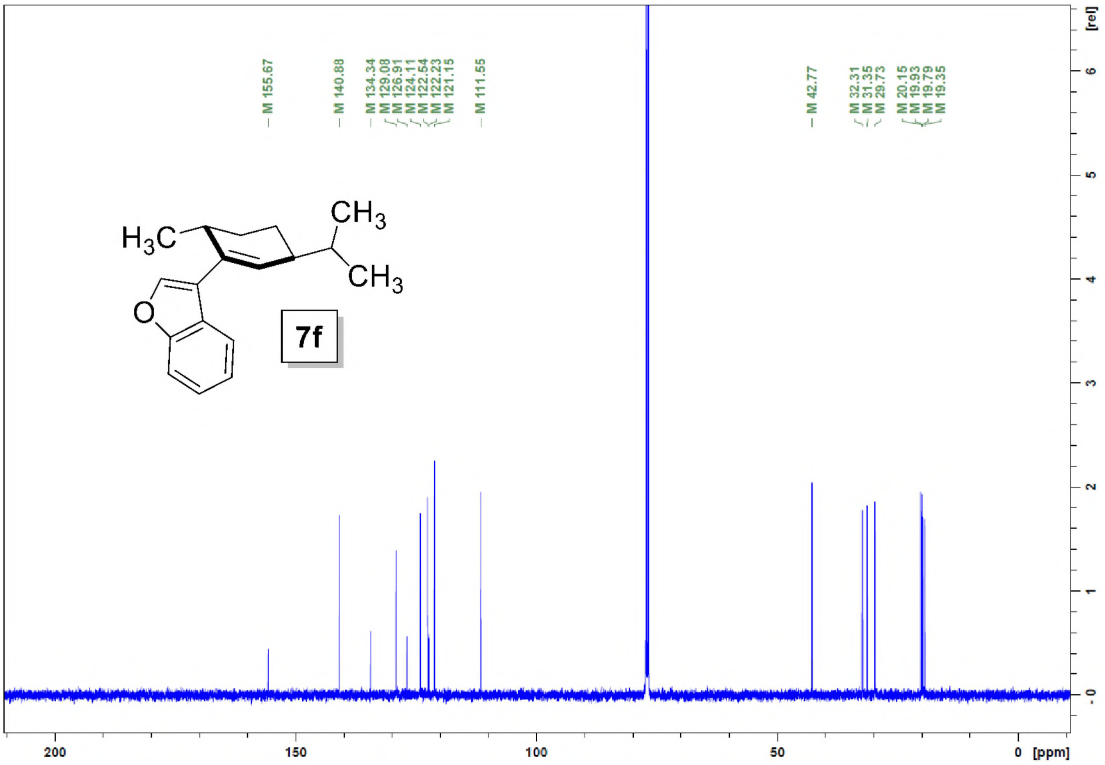
M 155.67
M 140.88
M 134.34
M 129.08
M 126.91
M 124.11
M 122.54
M 122.23
M 121.15
M 111.55
M 42.77
M 32.31
M 31.35
M 29.73
M 20.15
M 19.93
M 19.79
M 19.35
$H_3C$
$CH_3$
$CH_3$
O
7f
[rel]
[ppm]

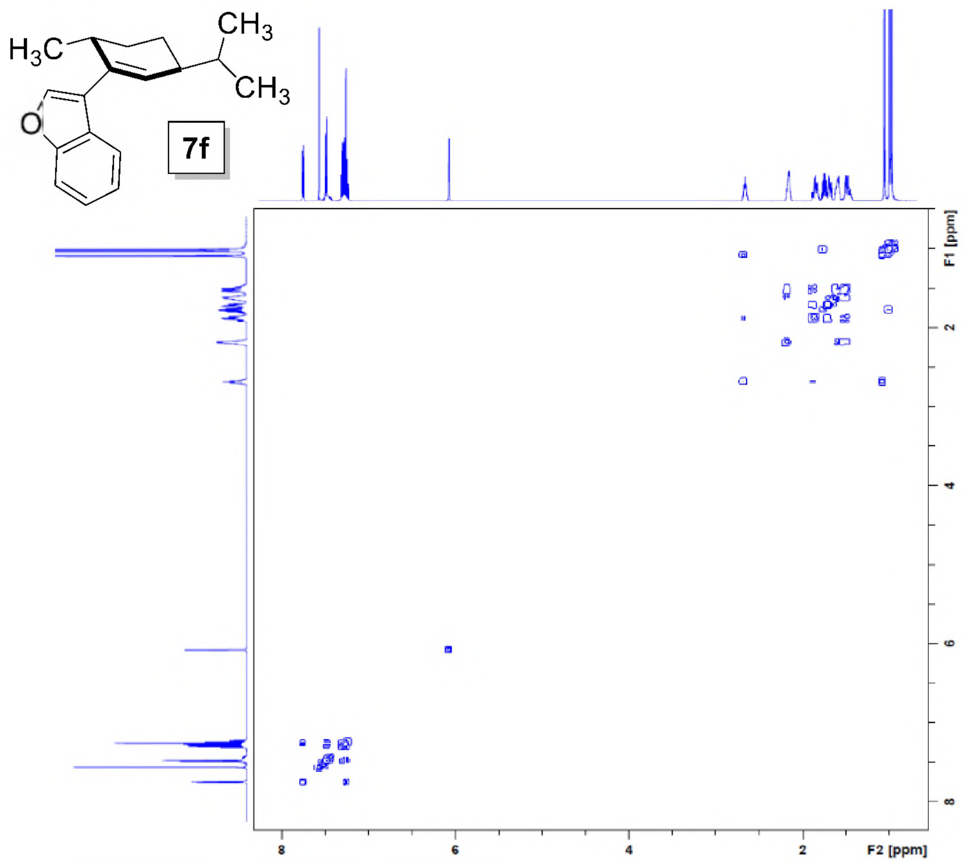
H3C
CH3
CH3
O
7f
F1 [ppm]
F2 [ppm]
2
4
6
8

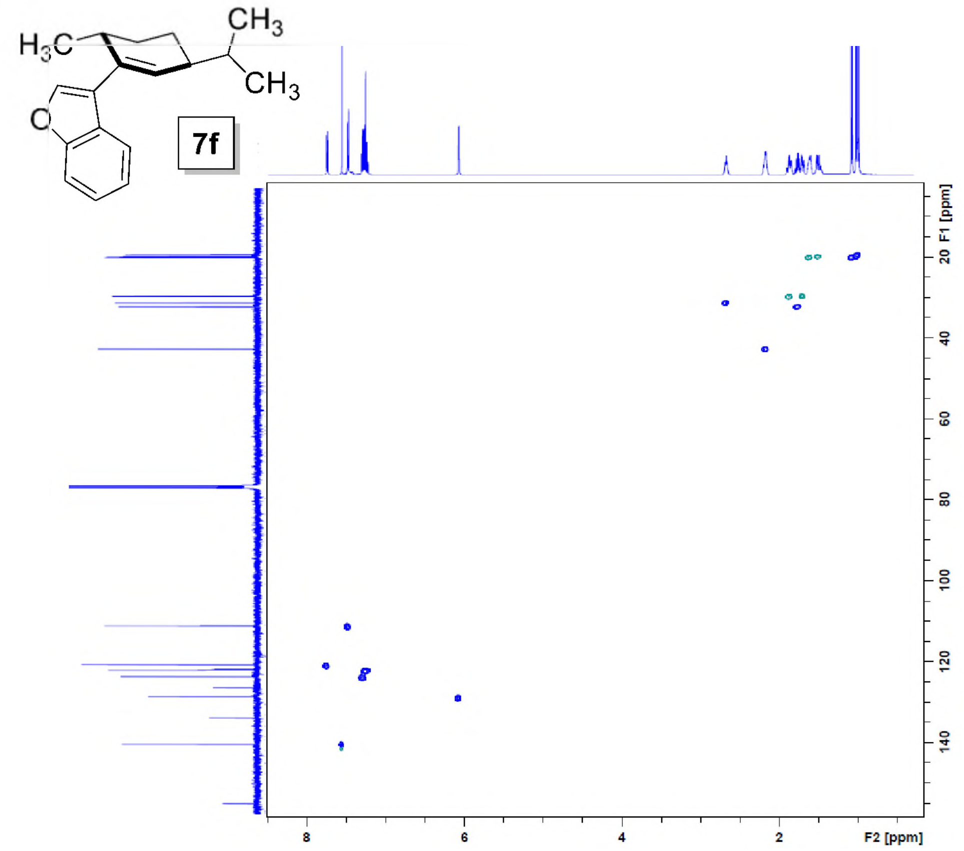
H3C
CH3
CH3
O
7f
F1 [ppm]
F2 [ppm]
20
40
60
80
100
120
140
8
6
4
2

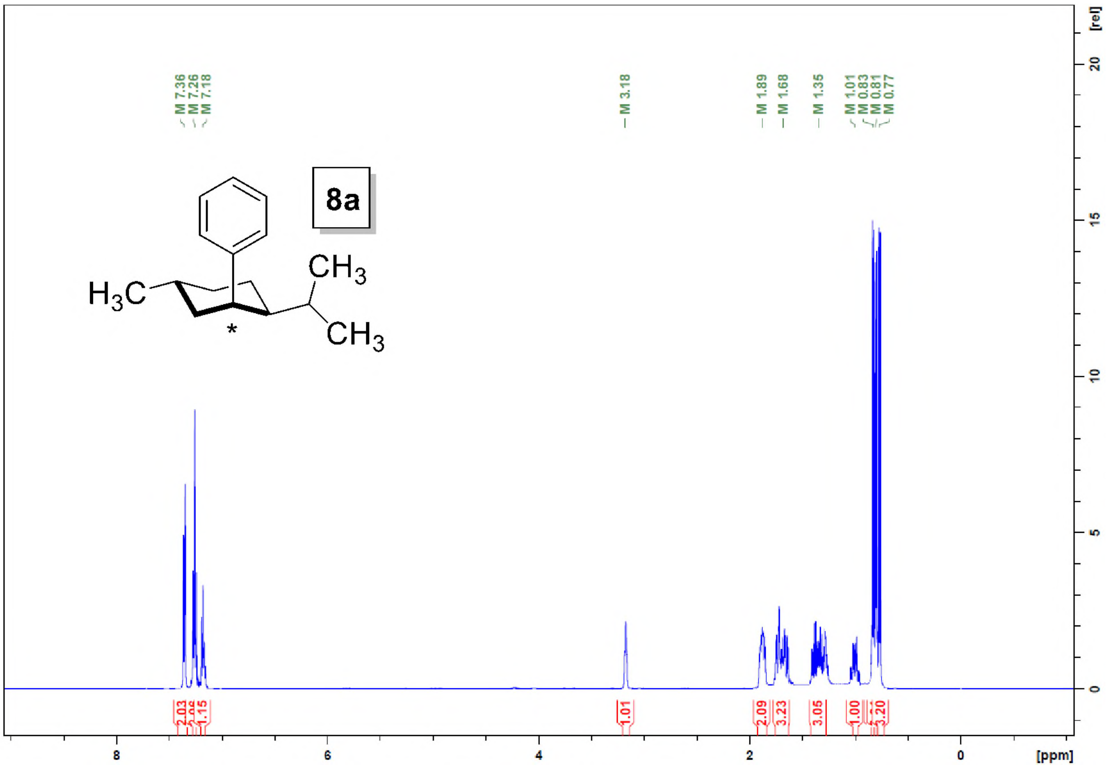

8a
H3C
CH3
CH3
*
M 7.36
M 7.26
M 7.18
M 3.18
M 1.89
M 1.68
M 1.35
M 1.01
M 0.83
M 0.81
M 0.77
[ppm]
[rel]


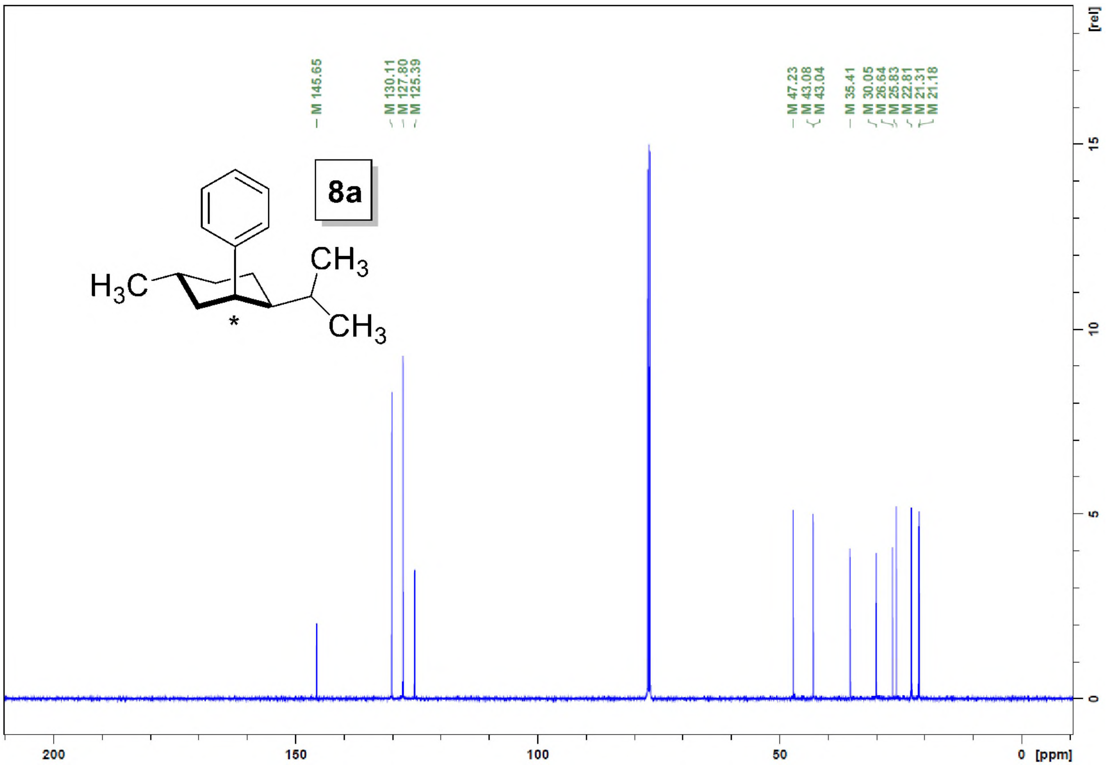

8a
H3C
CH3
CH3
*
M 145.65
M 130.11
M 127.80
M 125.39
M 47.23
M 43.08
M 43.04
M 35.41
M 30.05
M 26.64
M 25.83
M 22.81
M 21.31
M 21.18
[ppm]
[rel]

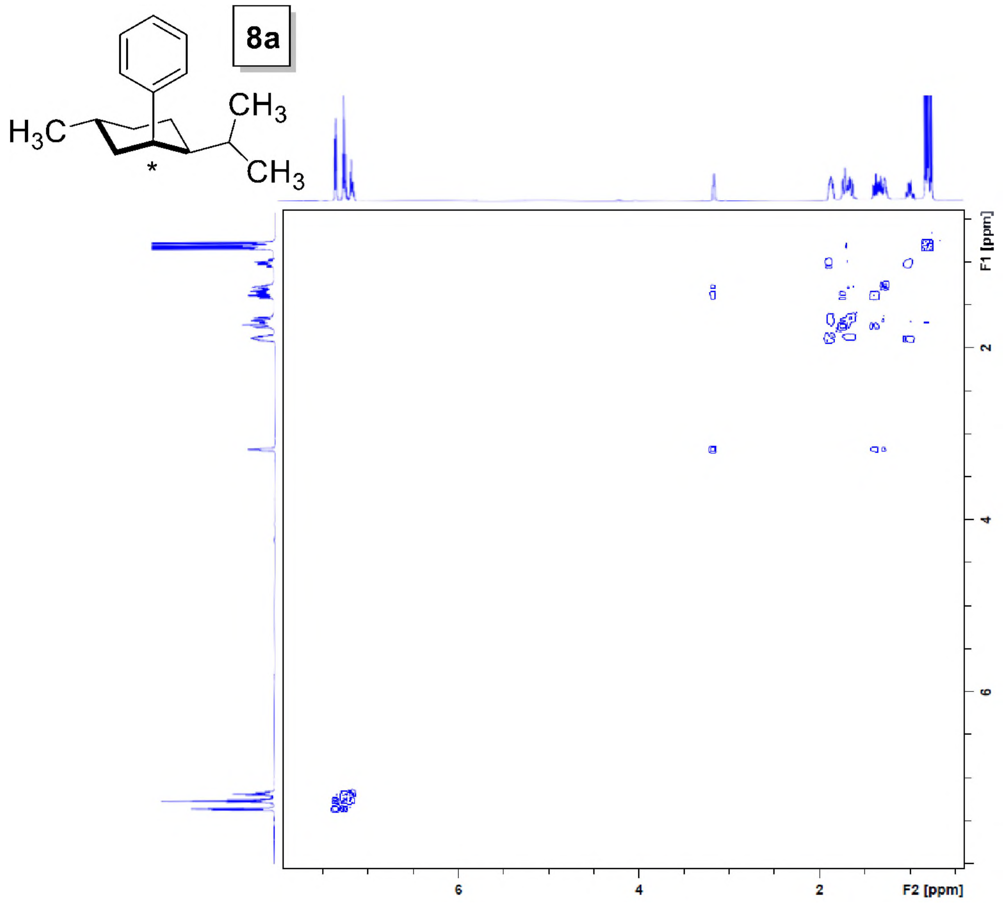
8a
H3C
CH3
CH3
*
F1 [ppm]
F2 [ppm]
2
4
6

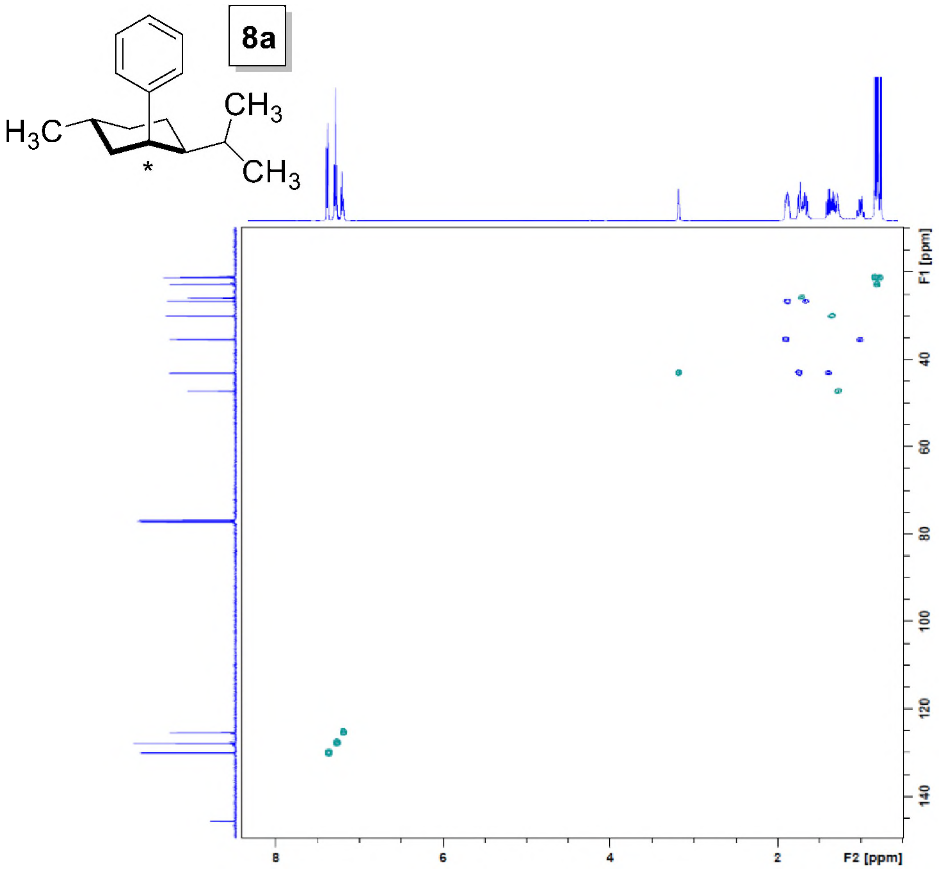
8a
H3C
CH3
CH3
*
F1 [ppm]
F2 [ppm]
40
60
80
100
120
140
2
4
6
8

**8a**

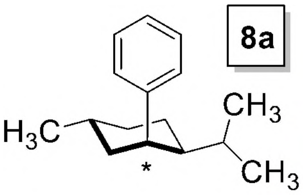


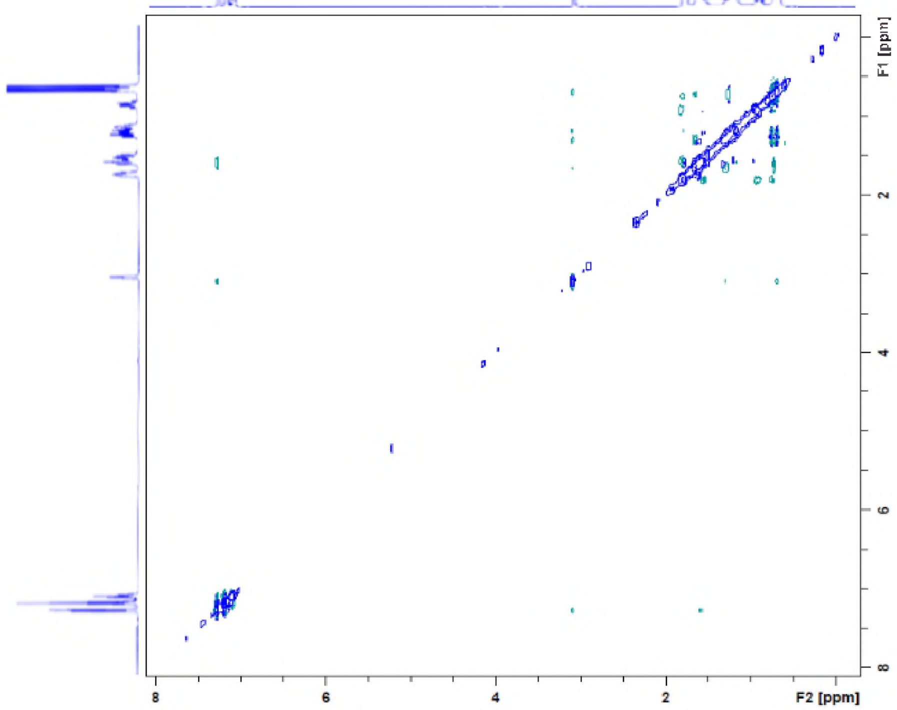

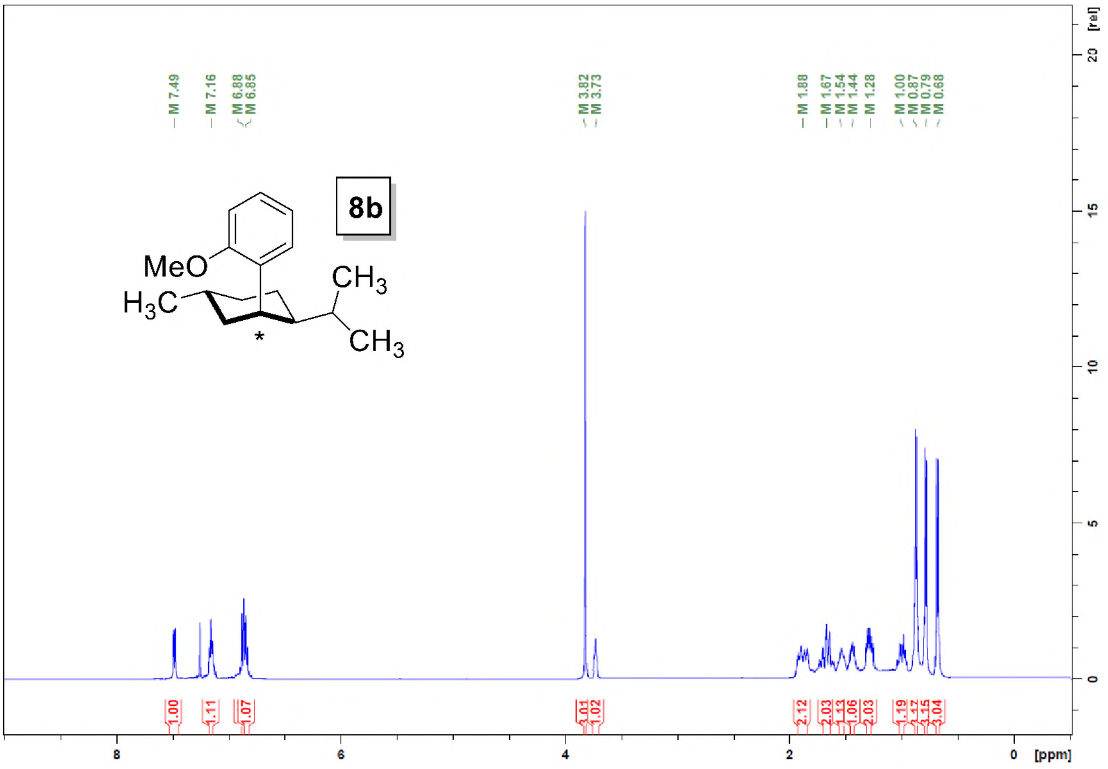
8b
MeO
H3C
CH3
CH3
[ppm]
[rel]

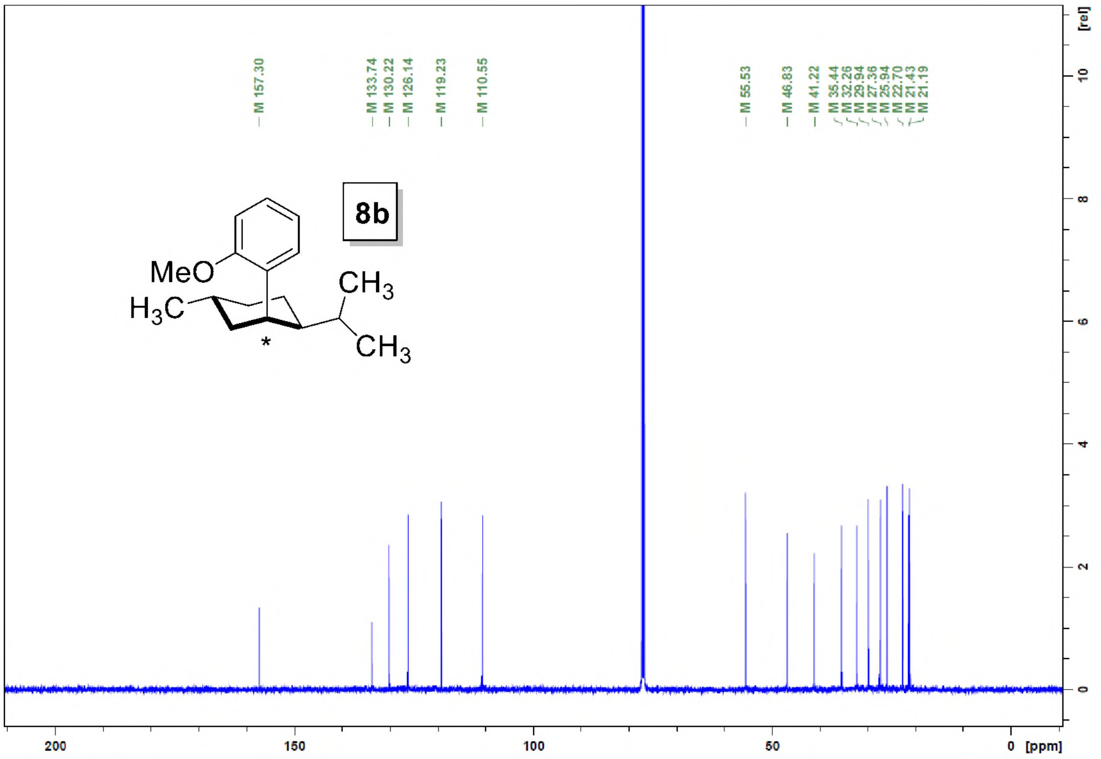
8b
MeO
H3C
CH3
CH3
[ppm]
[rel]

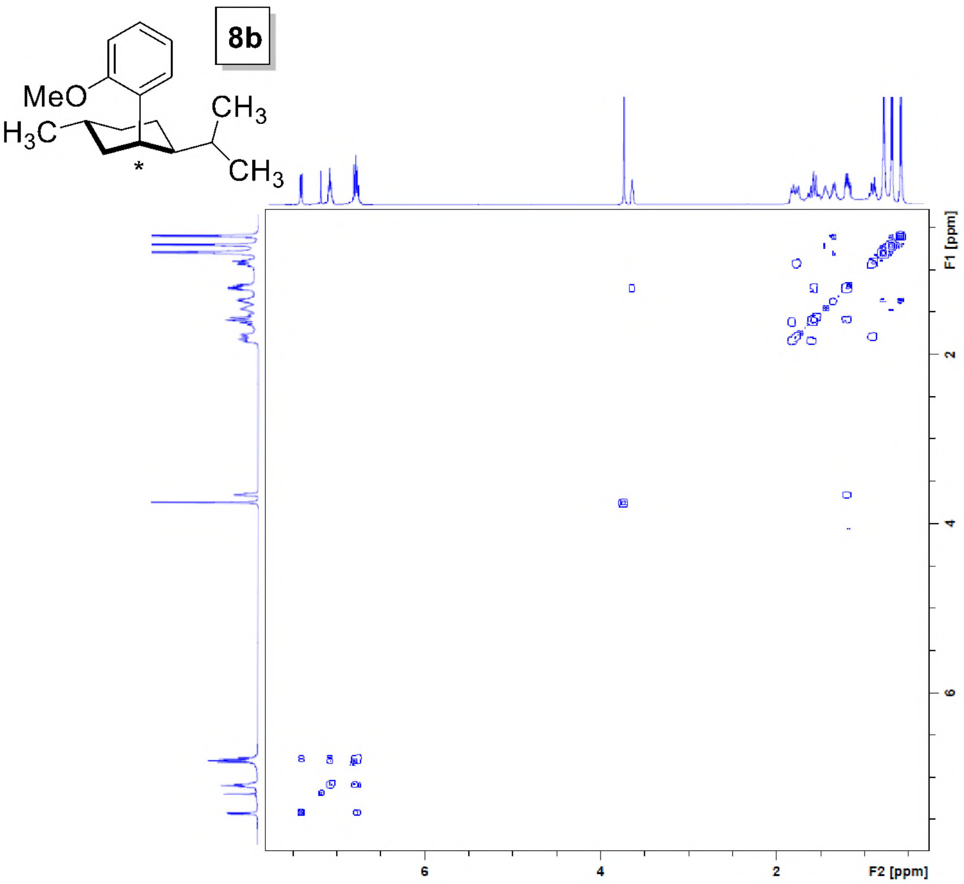
8b
MeO
$H_3C$
$CH_3$
$CH_3$
*
F1 [ppm]
2
4
6
F2 [ppm]

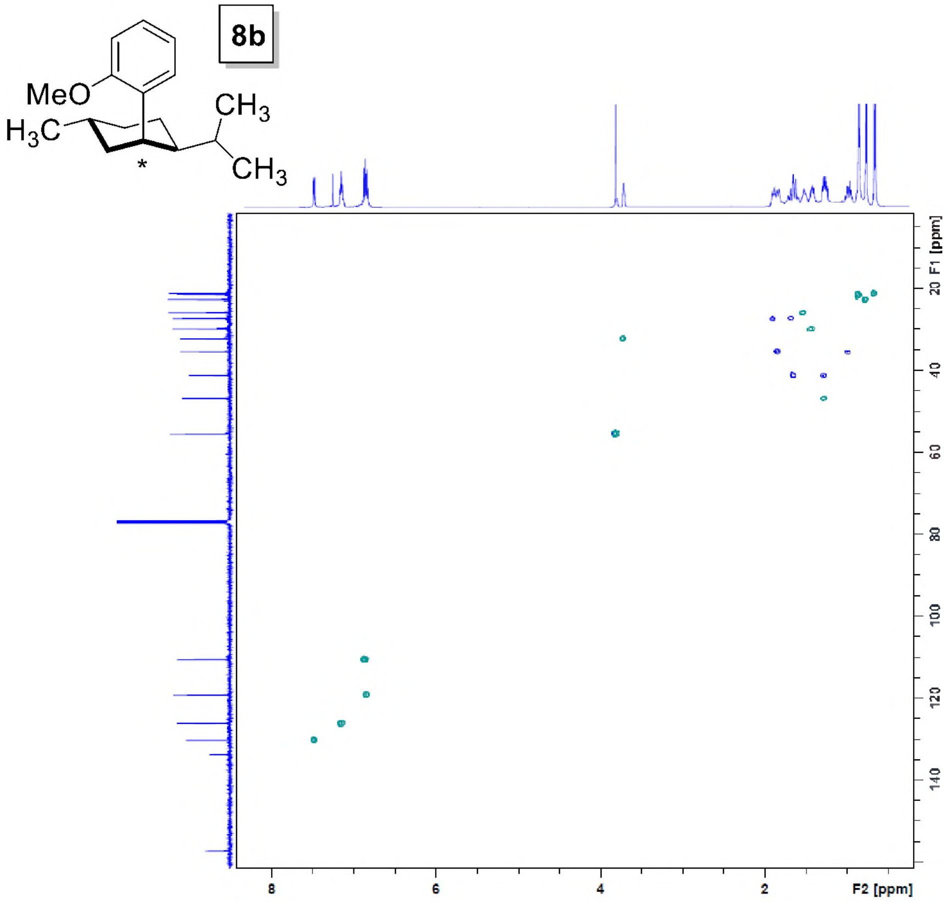
8b
MeO
$H_3C$
$CH_3$
$CH_3$
*
F1 [ppm]
20
40
60
80
100
120
140
8
6
4
2
F2 [ppm]

**8b**

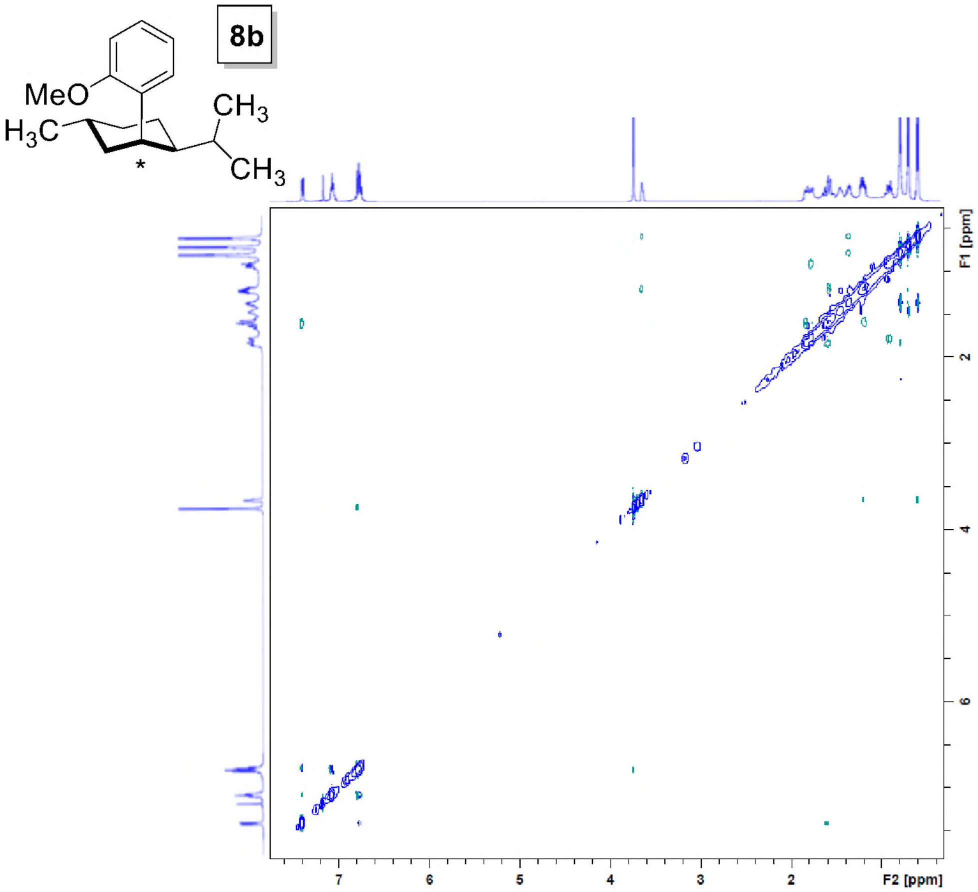

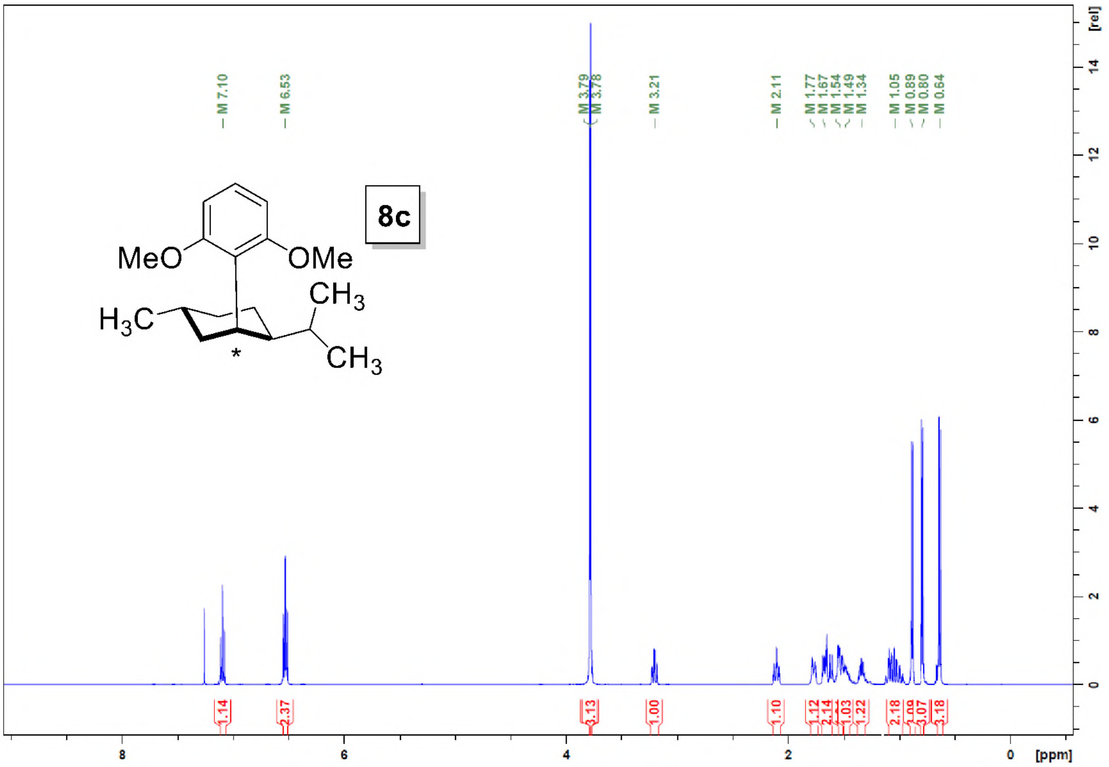

8c
MeO
OMe
$H_3C$
$CH_3$
$CH_3$
*
M 7.10
M 6.53
M 3.79
M 3.78
M 3.21
M 2.11
M 1.77
M 1.67
M 1.54
M 1.49
M 1.34
M 1.05
M 0.89
M 0.80
M 0.64
1.14
2.37
3.13
1.00
1.10
1.12
2.14
1.03
1.22
2.18
3.07
3.18
[rel]
[ppm]


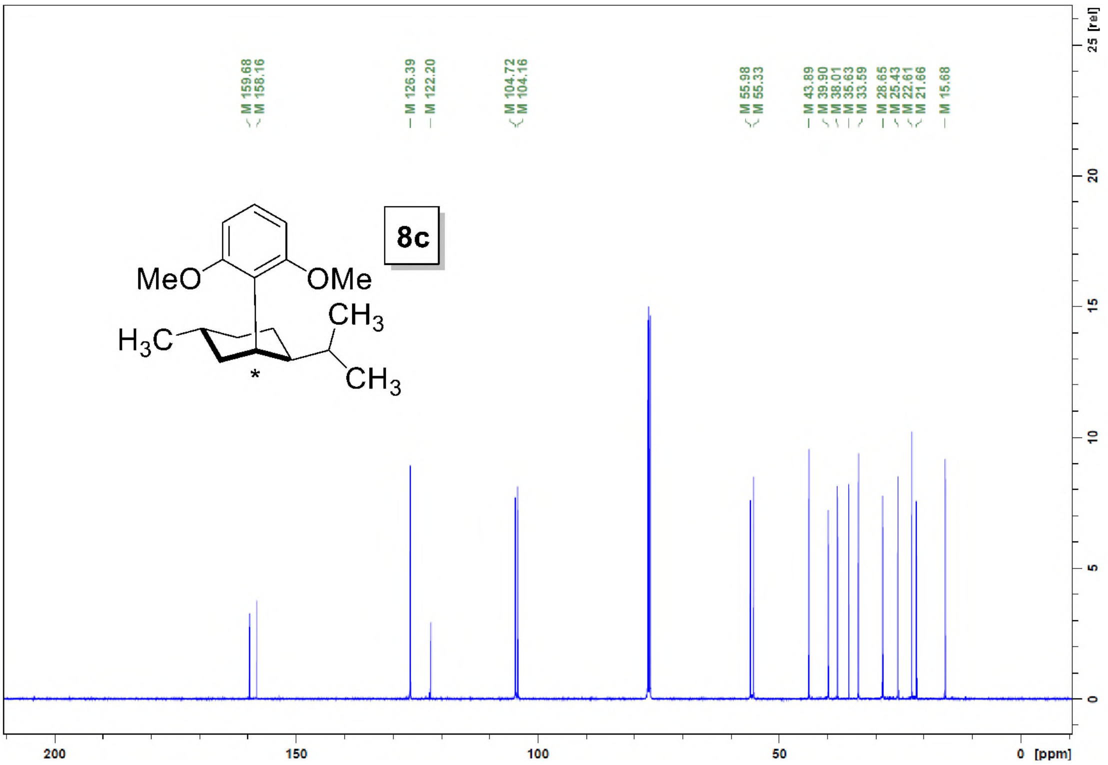

8c
MeO
OMe
$H_3C$
$CH_3$
$CH_3$
*
M 159.68
M 158.16
M 126.39
M 122.20
M 104.72
M 104.16
M 55.38
M 55.33
M 43.89
M 39.90
M 38.01
M 35.63
M 33.59
M 28.65
M 25.43
M 22.61
M 21.66
M 15.68
[rel]
[ppm]

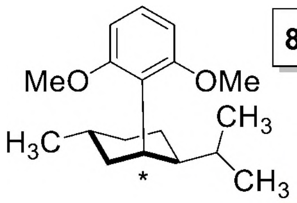
MeO
OMe
H3C
CH3
CH3
*

**8c**

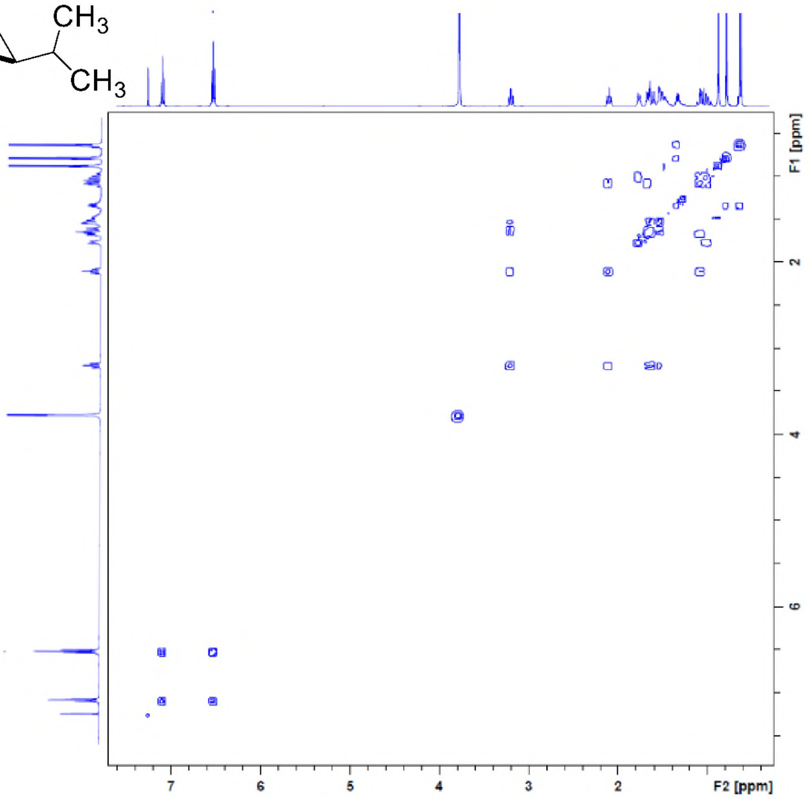
F1 [ppm]
2
4
6
7
6
5
4
3
2
F2 [ppm]

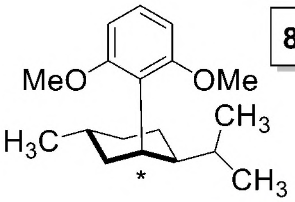
MeO
OMe
H3C
CH3
CH3
*

**8c**

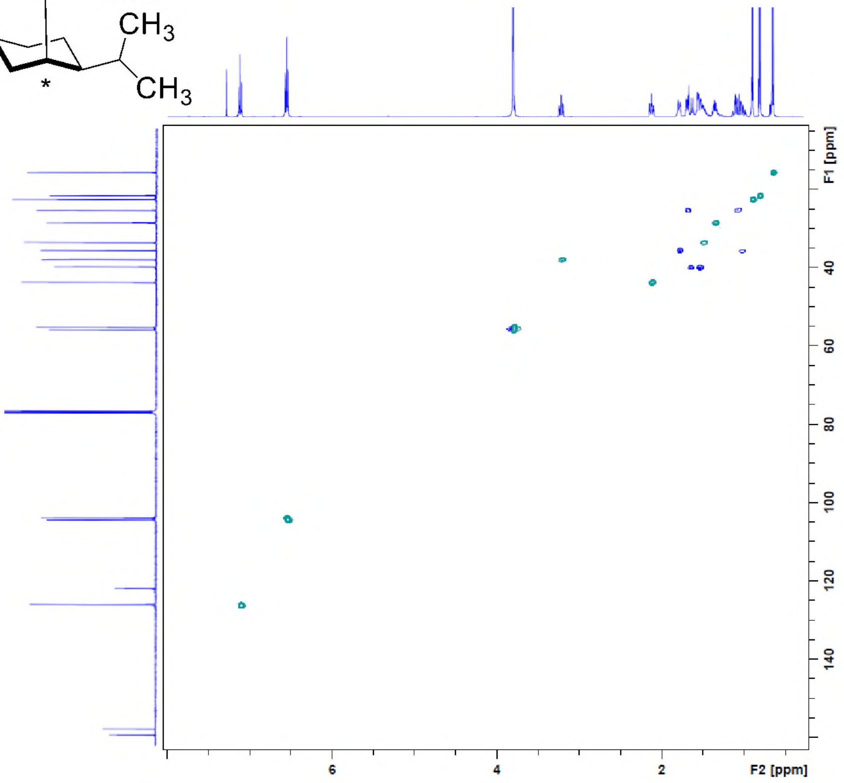
F1 [ppm]
40
60
80
100
120
140
6
4
2
F2 [ppm]

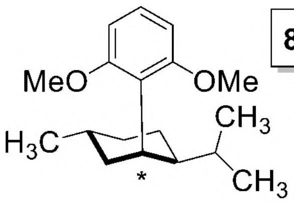


**8c**

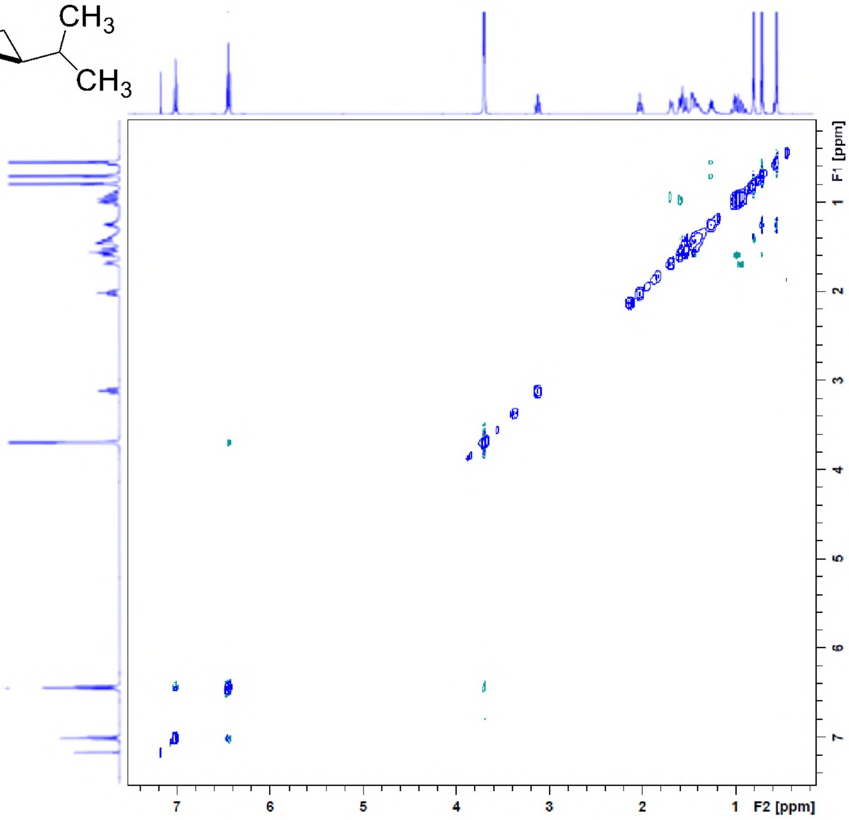

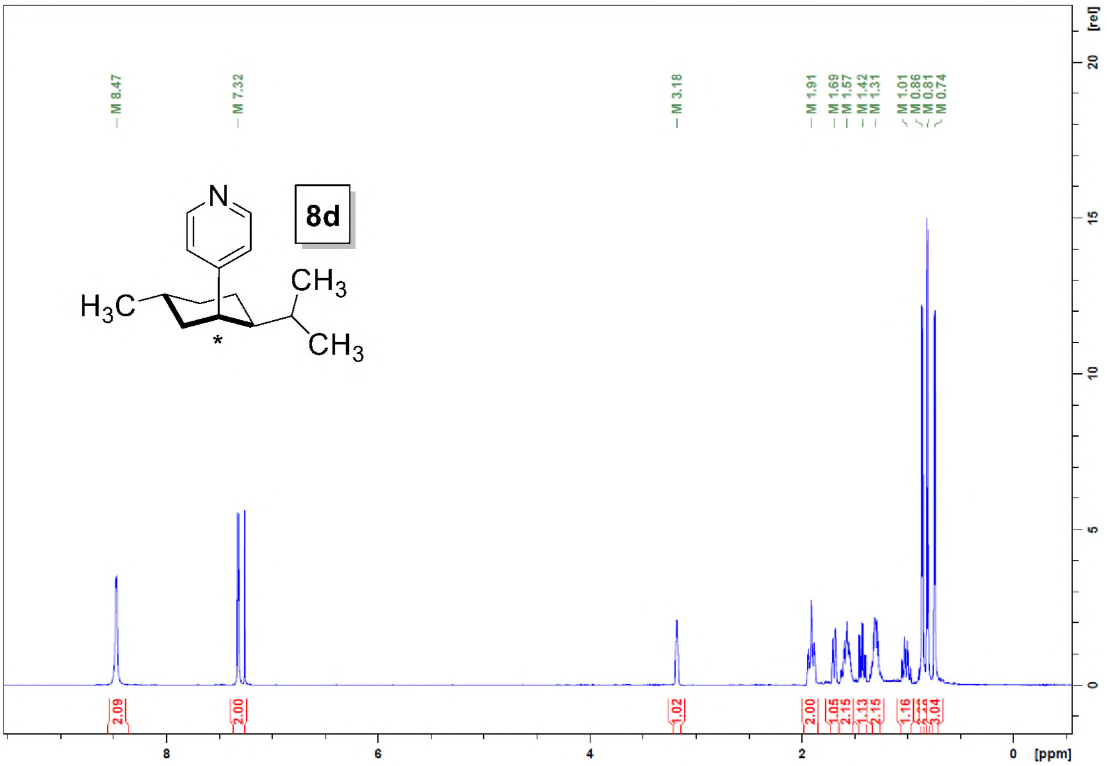
8d
H3C
CH3
CH3
M 8.47
M 7.32
M 3.18
M 1.91
M 1.69
M 1.57
M 1.42
M 1.31
M 1.01
M 0.86
M 0.81
M 0.74
[rel]
[ppm]

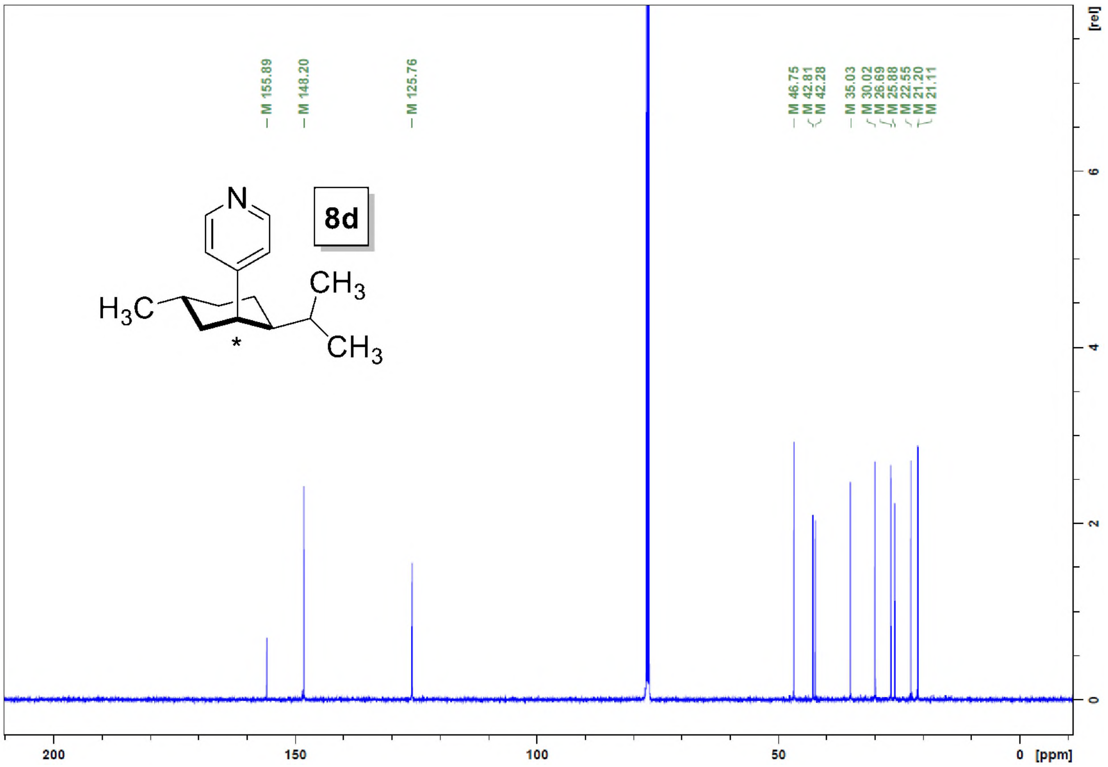
8d
H3C
CH3
CH3
M 155.89
M 148.20
M 125.76
M 46.75
M 42.81
M 42.28
M 35.03
M 30.02
M 28.69
M 25.88
M 22.55
M 21.20
M 21.11
[rel]
[ppm]

**8d**

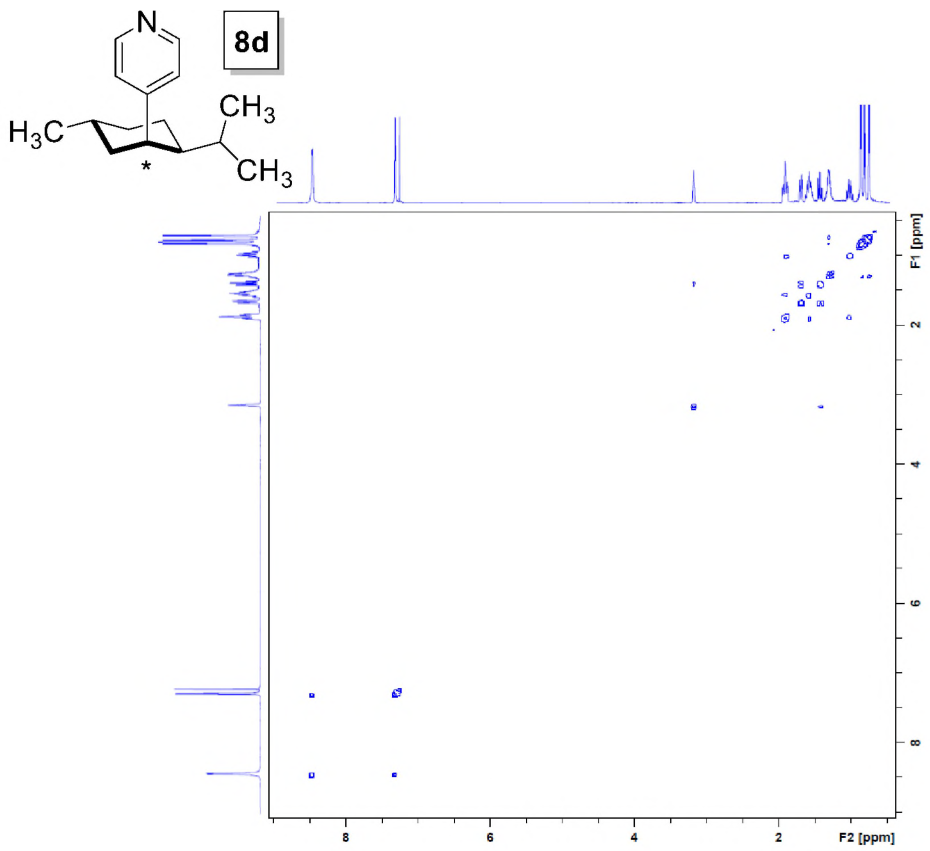
H3C
CH3
CH3
N
*
F1 [ppm]
F2 [ppm]
2
4
6
8

**8d**

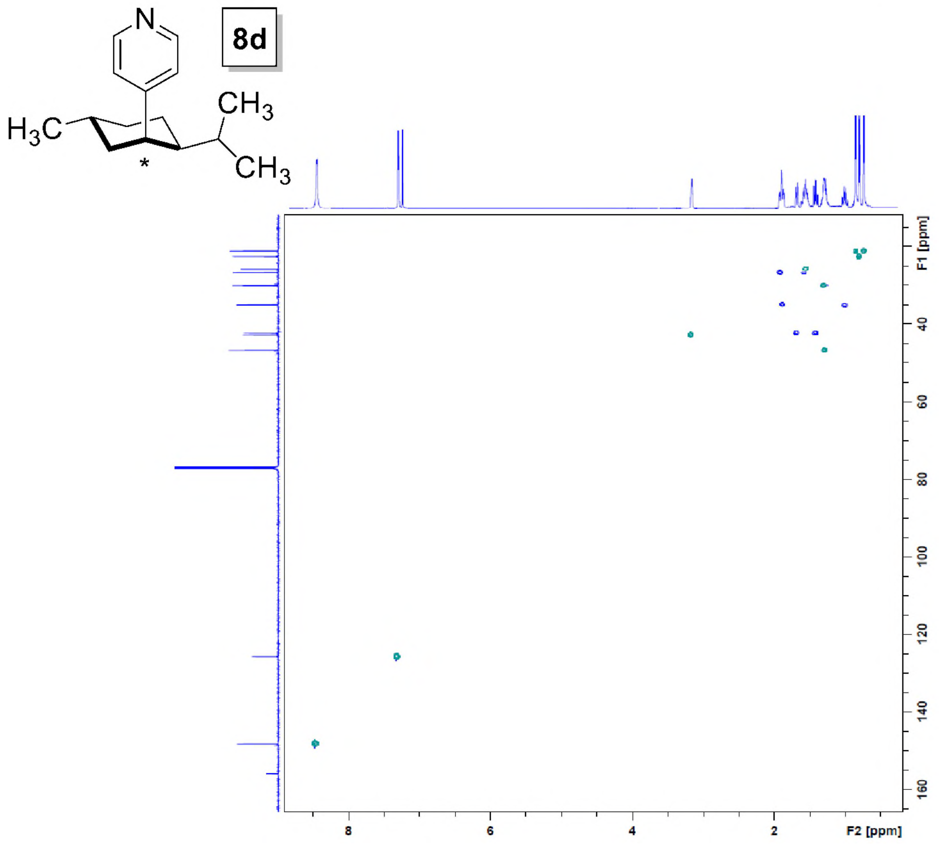
H3C
CH3
CH3
N
*
F1 [ppm]
F2 [ppm]
40
60
80
100
120
140
160
8
6
4
2

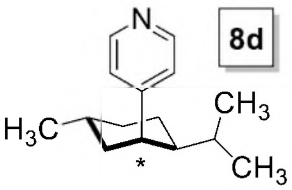

**8d**

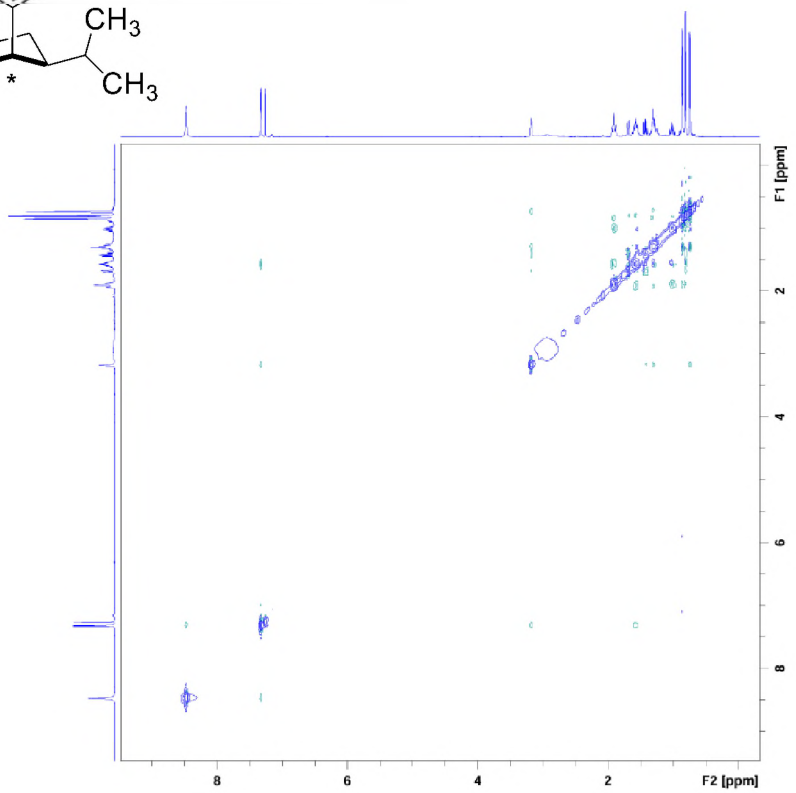

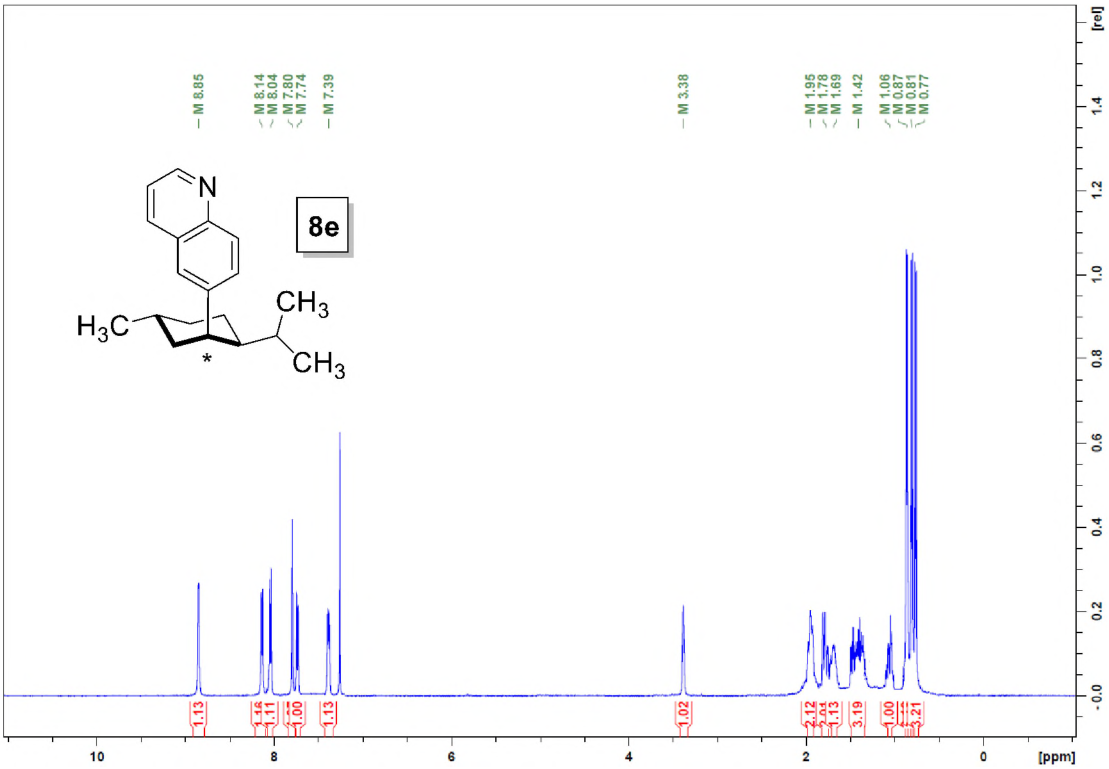

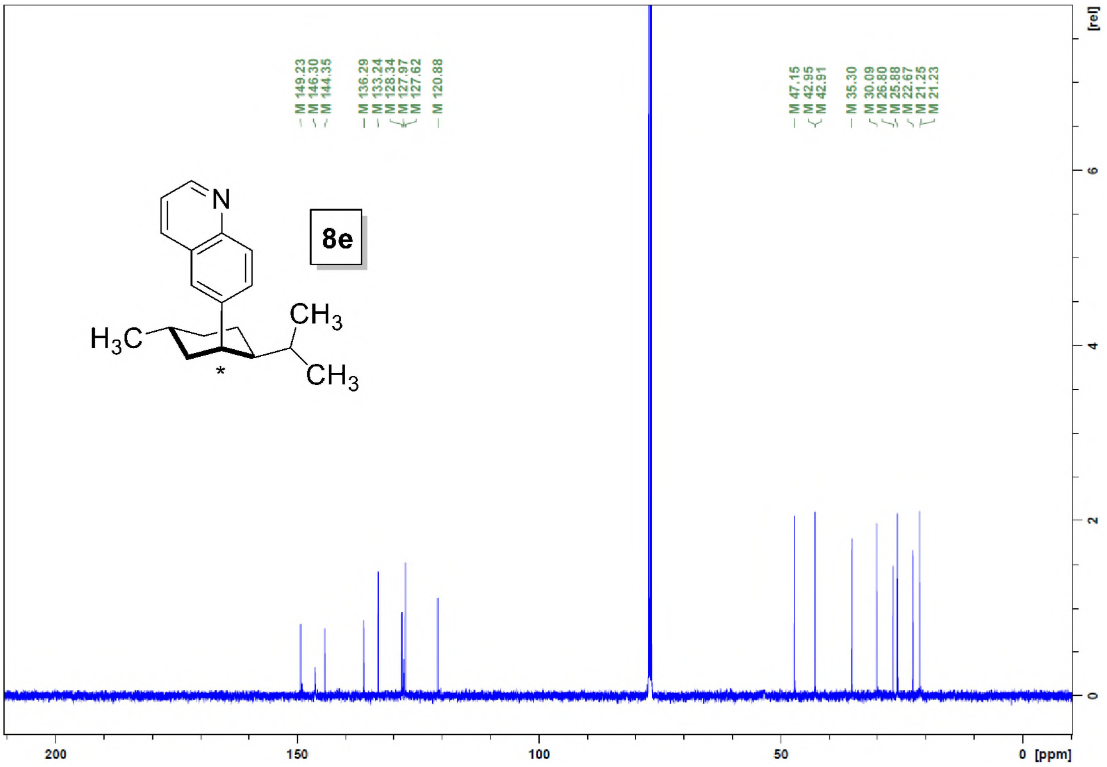

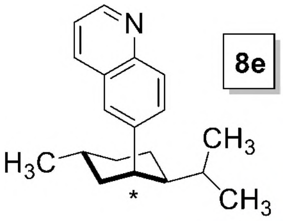


**8e**

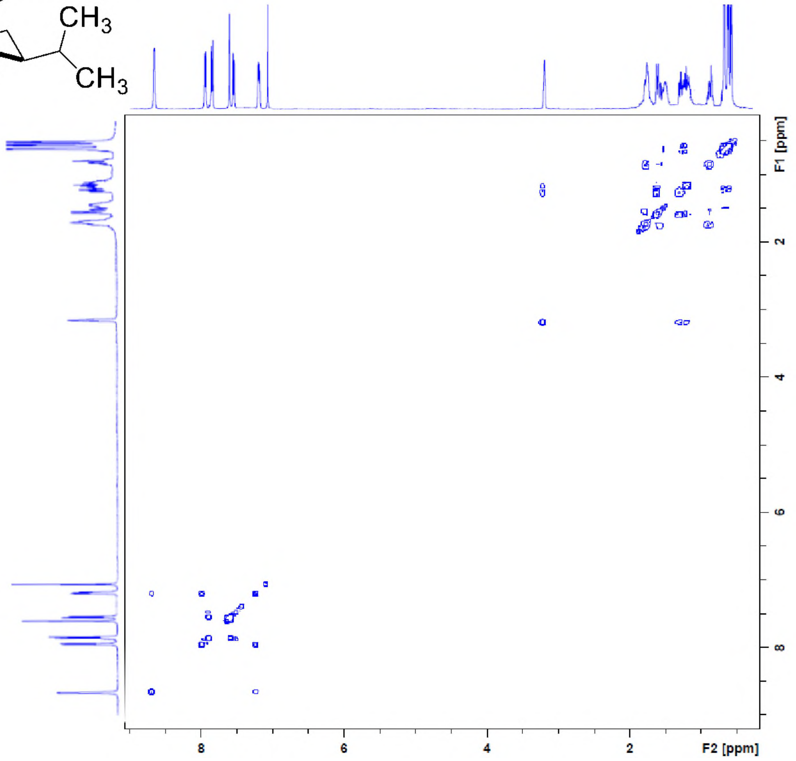


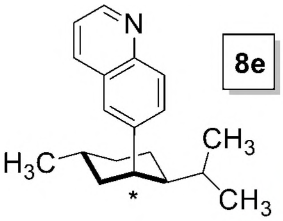


**8e**

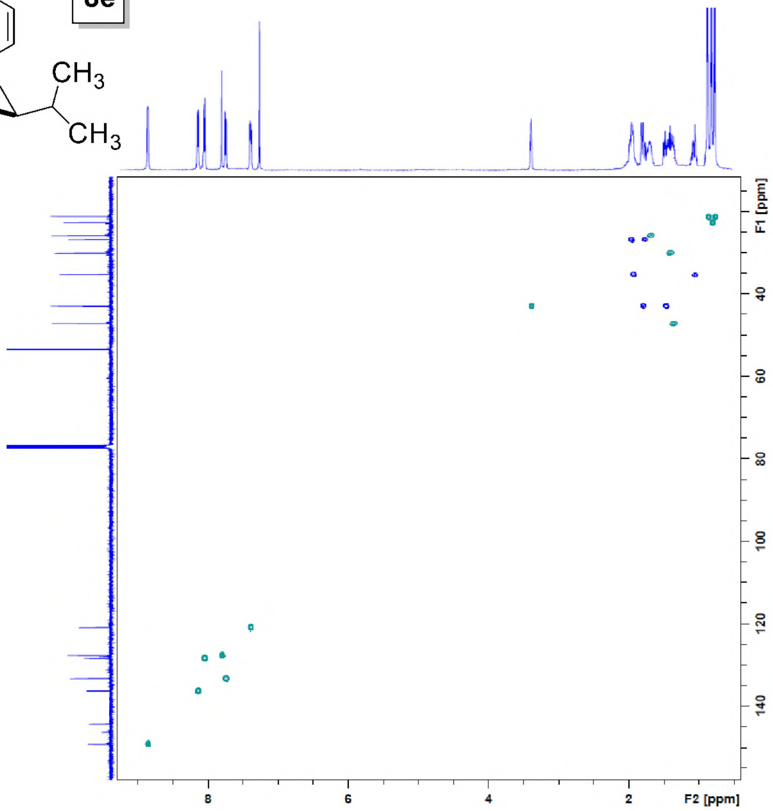

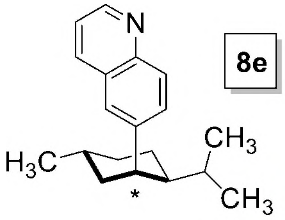

**8e**

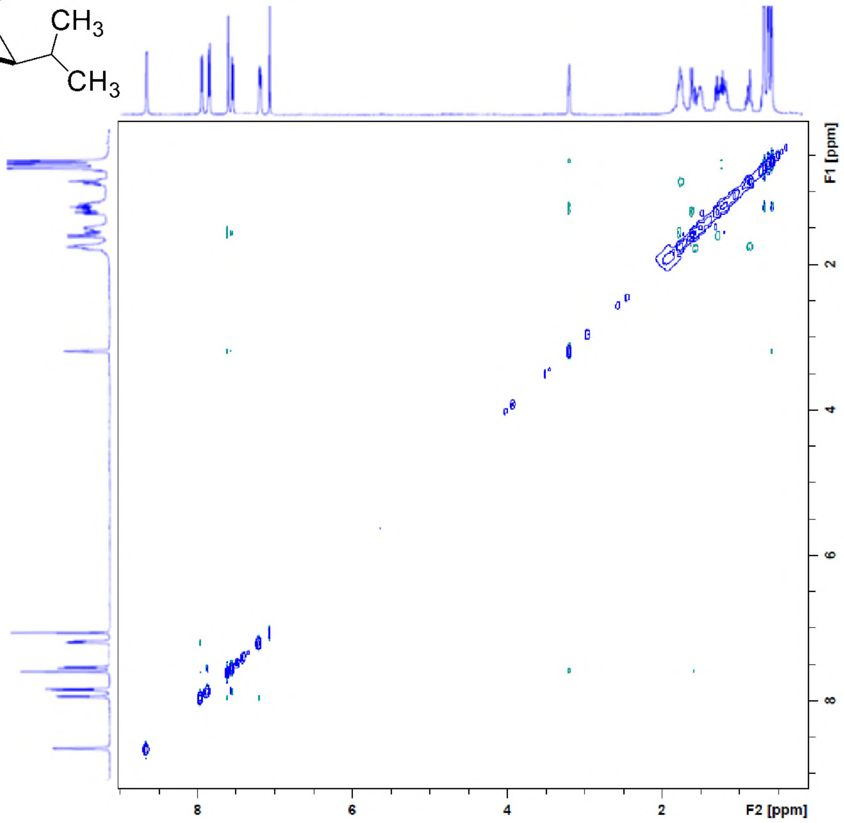

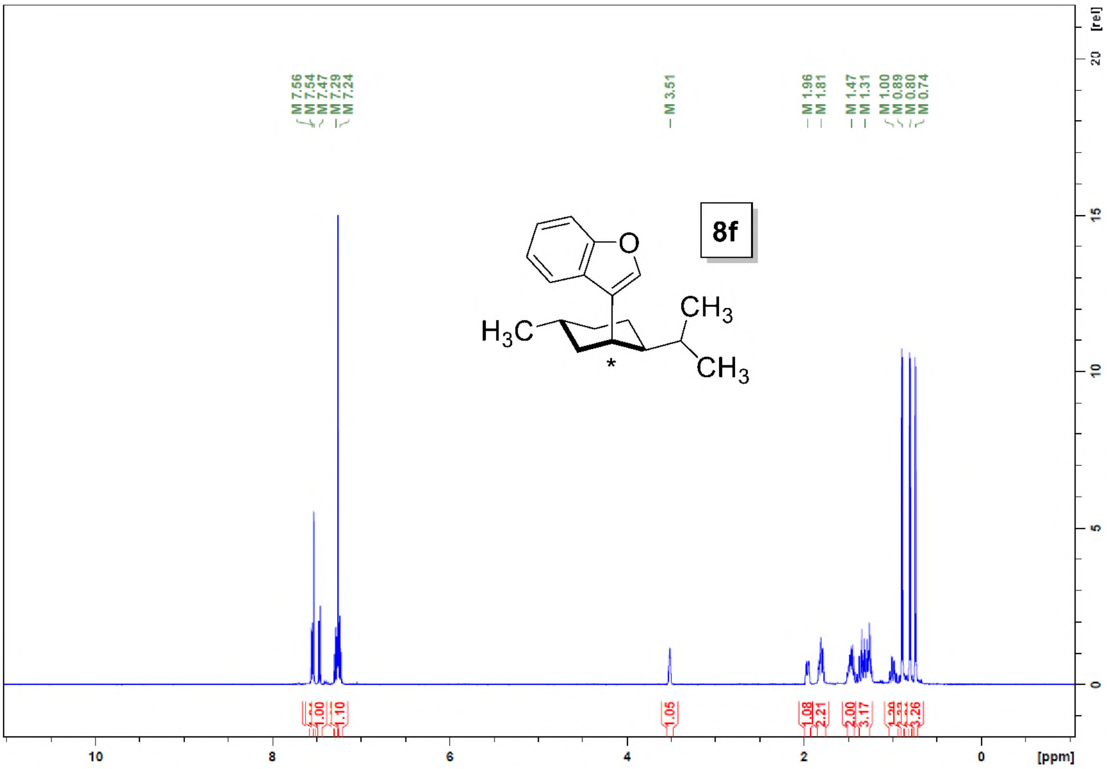
8f
H3C
CH3
CH3
M 7.56
M 7.54
M 7.47
M 7.29
M 7.24
M 3.51
M 1.96
M 1.81
M 1.47
M 1.31
M 1.00
M 0.89
M 0.80
M 0.74
1.00
1.10
1.05
1.08
2.21
2.00
3.17
3.26
[ppm]
[rel]

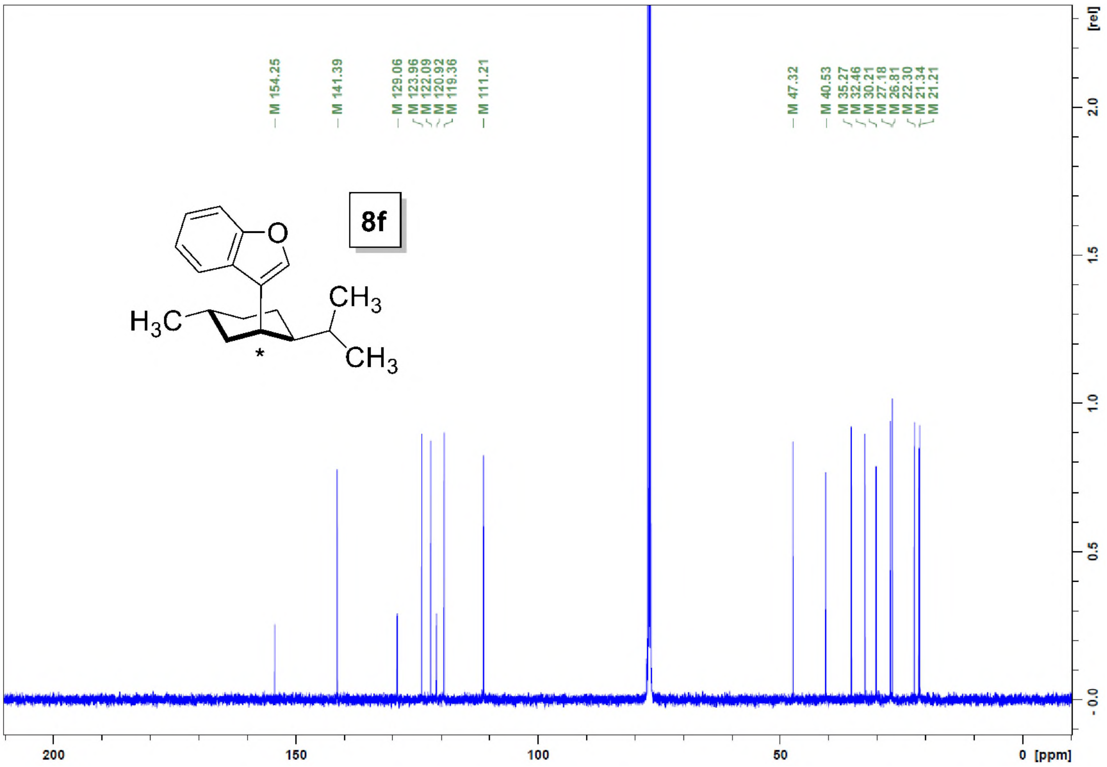
8f
H3C
CH3
CH3
M 154.25
M 141.39
M 129.06
M 123.96
M 122.09
M 120.92
M 119.36
M 111.21
M 47.32
M 40.53
M 35.27
M 32.46
M 30.21
M 27.18
M 26.81
M 22.30
M 21.34
M 21.21
[ppm]
[rel]

**8f**

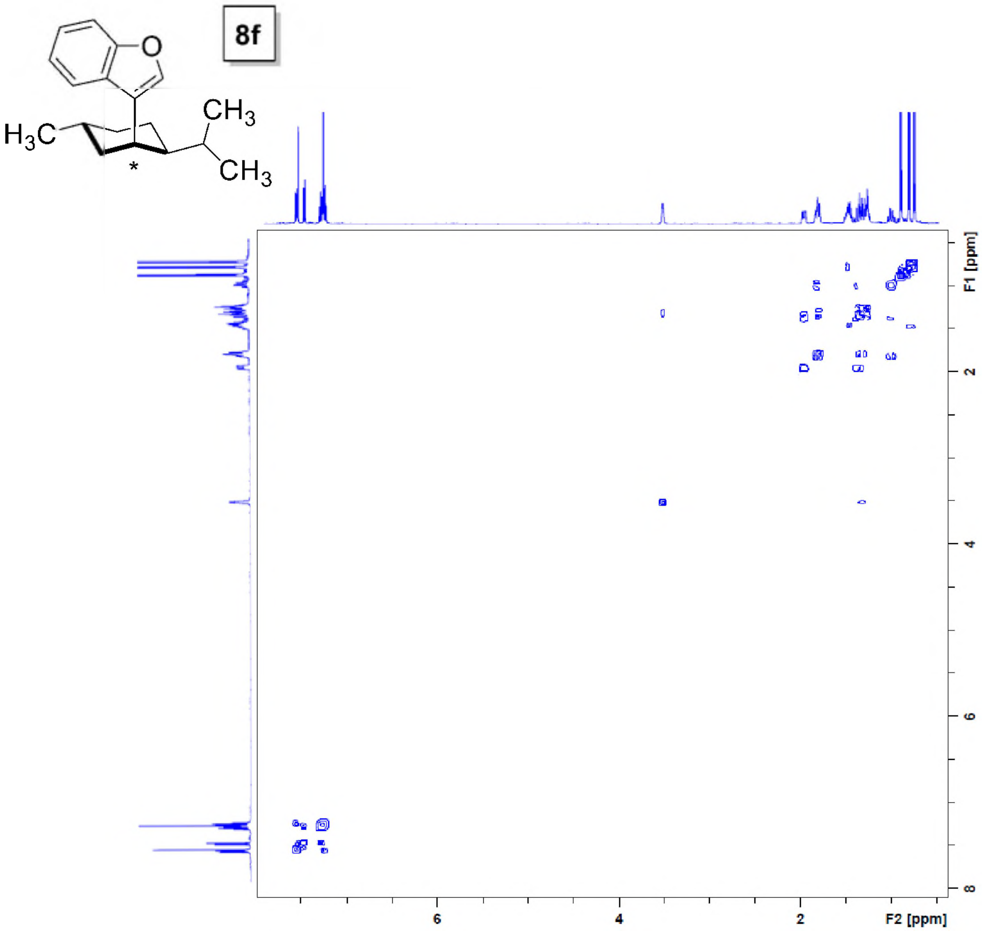
H3C
CH3
CH3
*
F1 [ppm]
F2 [ppm]
2
4
6
8

**8f**

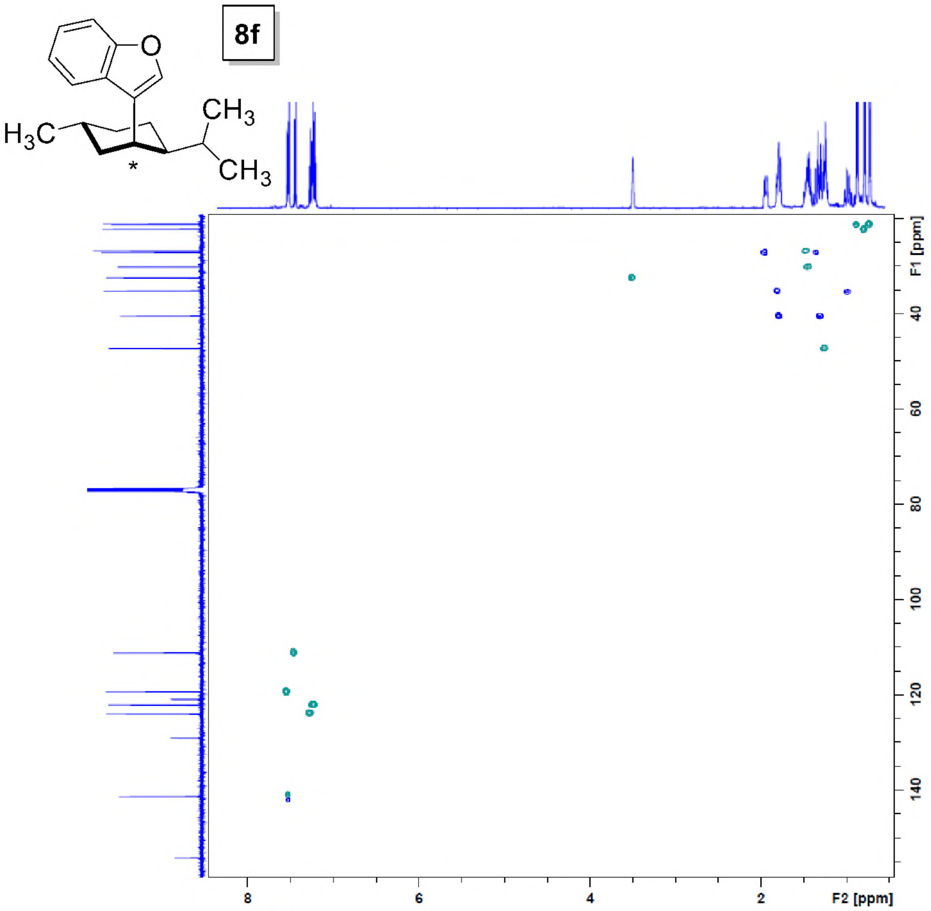
H3C
CH3
CH3
*
F1 [ppm]
F2 [ppm]
40
60
80
100
120
140
2
4
6
8

**8f**

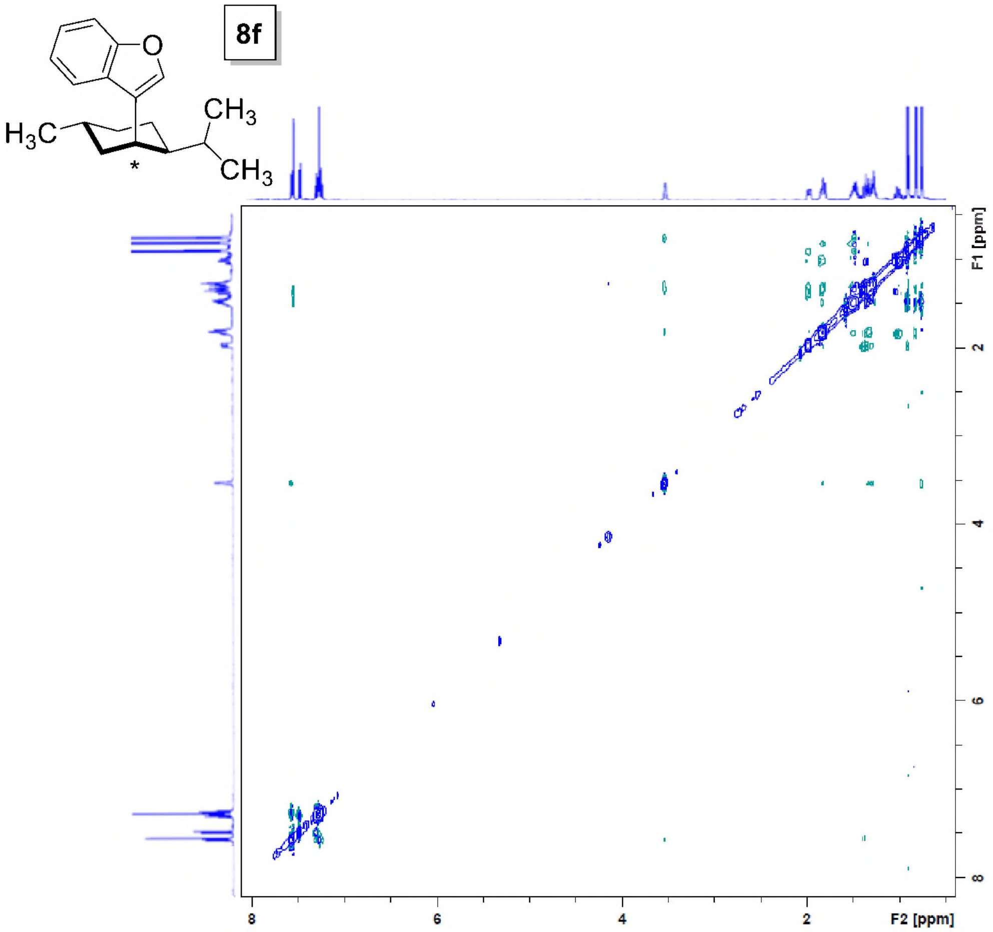

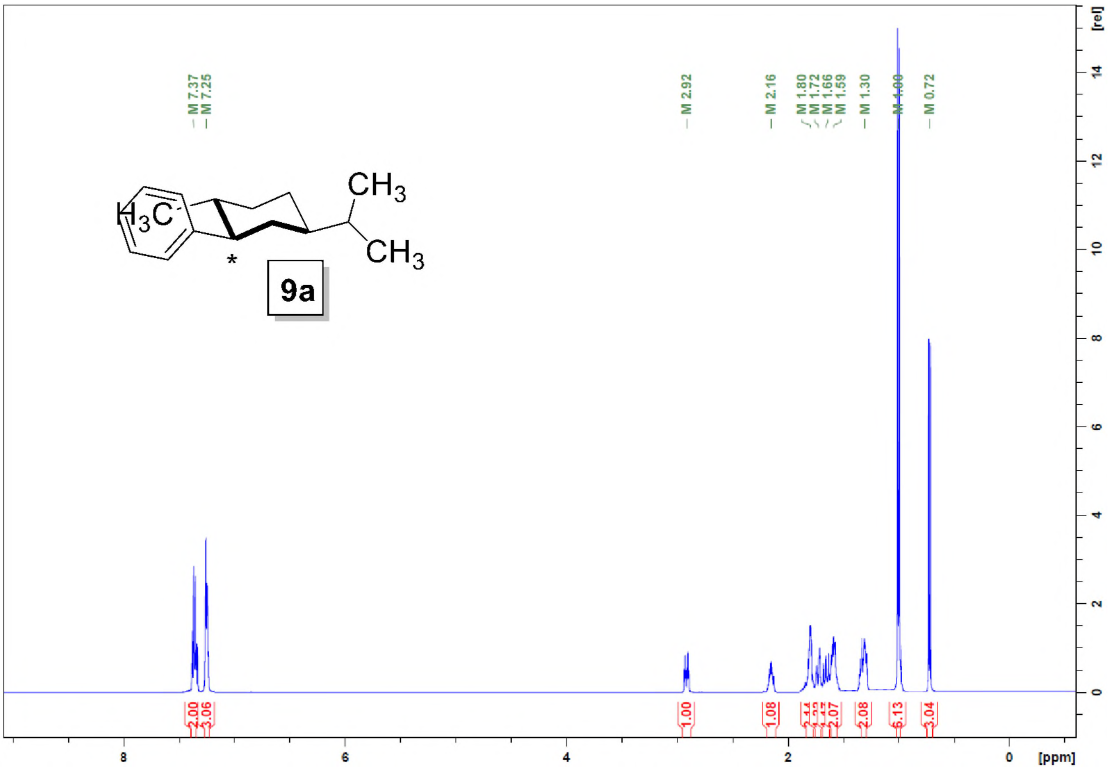
M 7.37
M 7.25
M 2.92
M 2.16
M 1.80
M 1.72
M 1.66
M 1.59
M 1.30
M 0.72
2.00
3.06
1.00
1.08
2.08
6.13
3.04
9a
[rel]
[ppm]

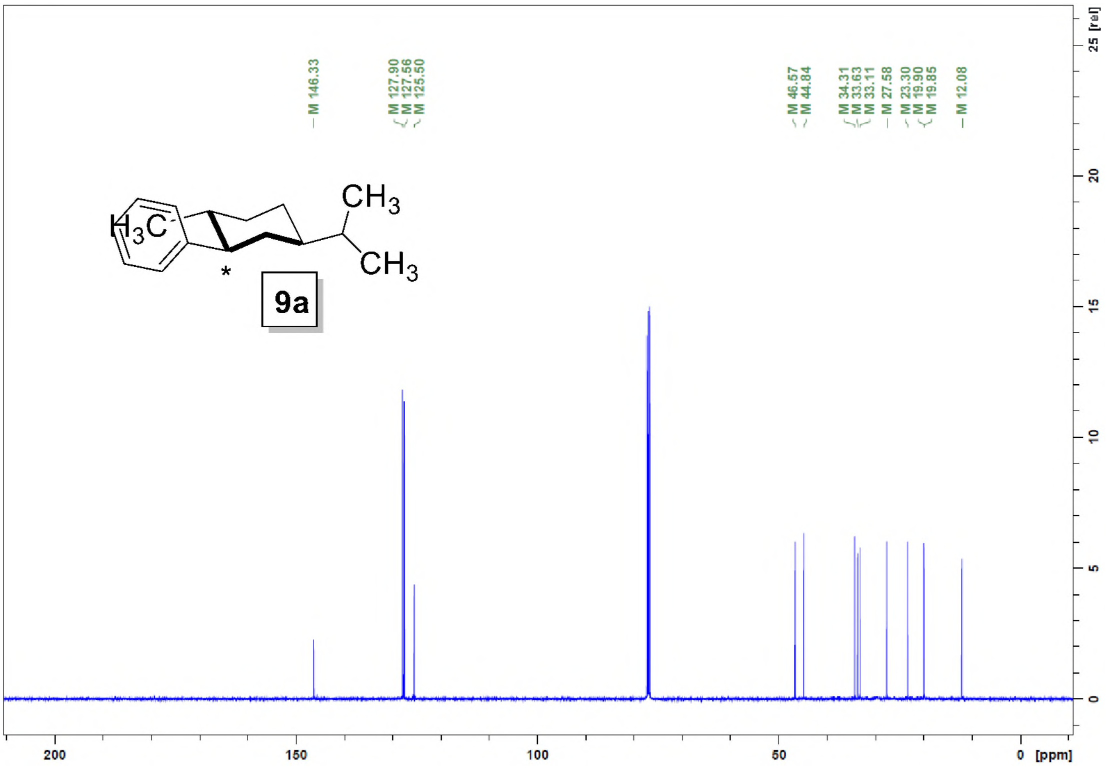
M 146.33
M 127.90
M 127.56
M 125.50
M 46.57
M 44.84
M 34.31
M 33.63
M 33.11
M 27.58
M 23.30
M 19.90
M 19.85
M 12.08
9a
[rel]
[ppm]

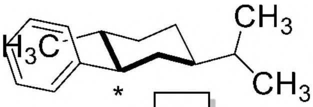

CH3
H3C
CH3
*


**9a**

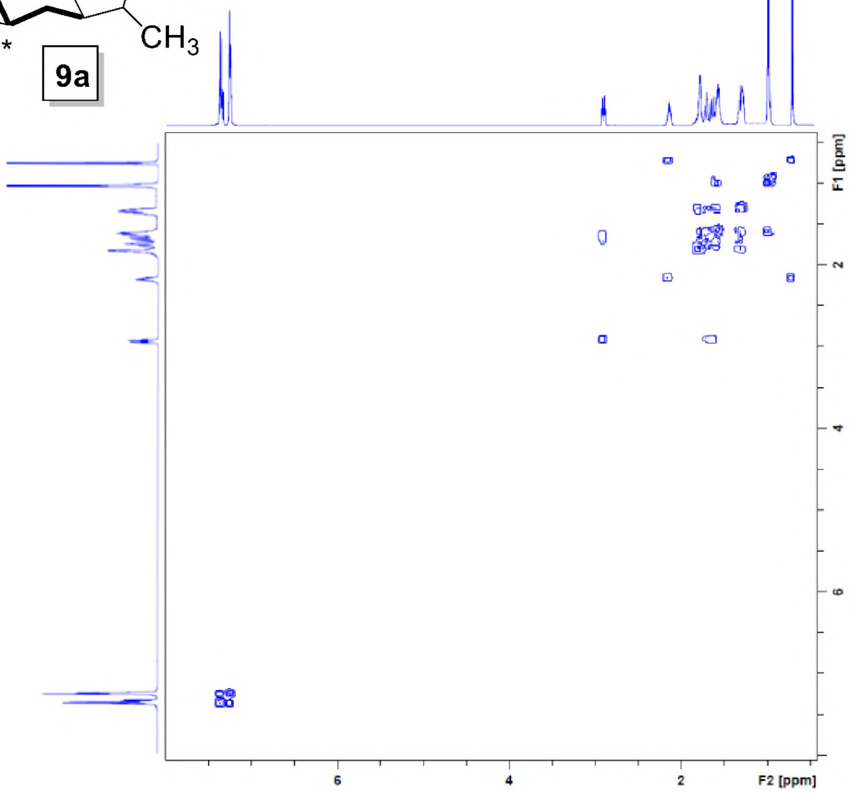

F1 [ppm]
F2 [ppm]
2
4
6


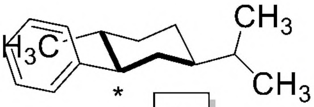

CH3
H3C
CH3
*


**9a**

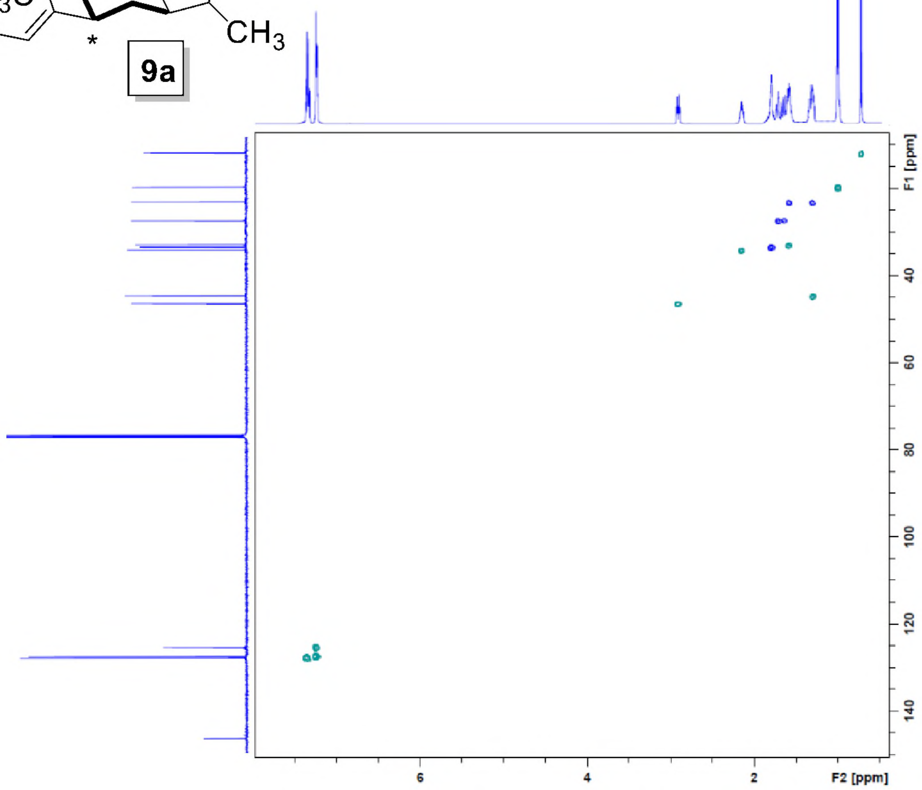

F1 [ppm]
F2 [ppm]
40
60
80
100
120
140
2
4
6

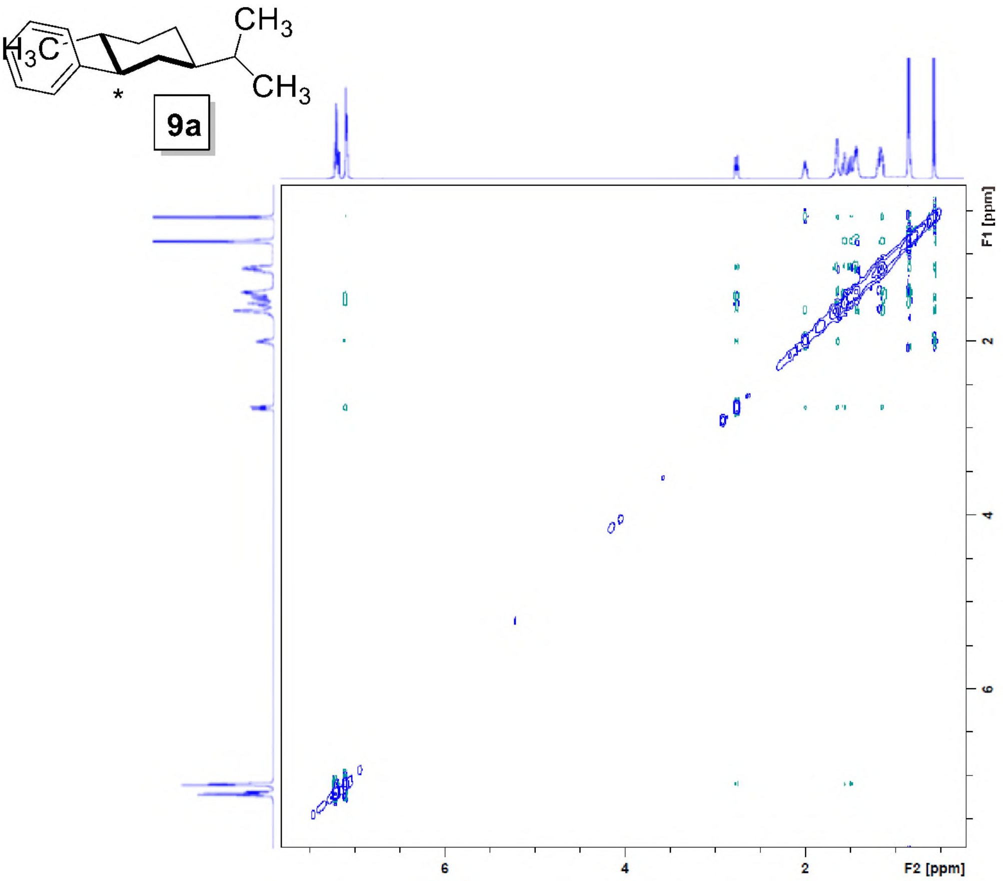


9a

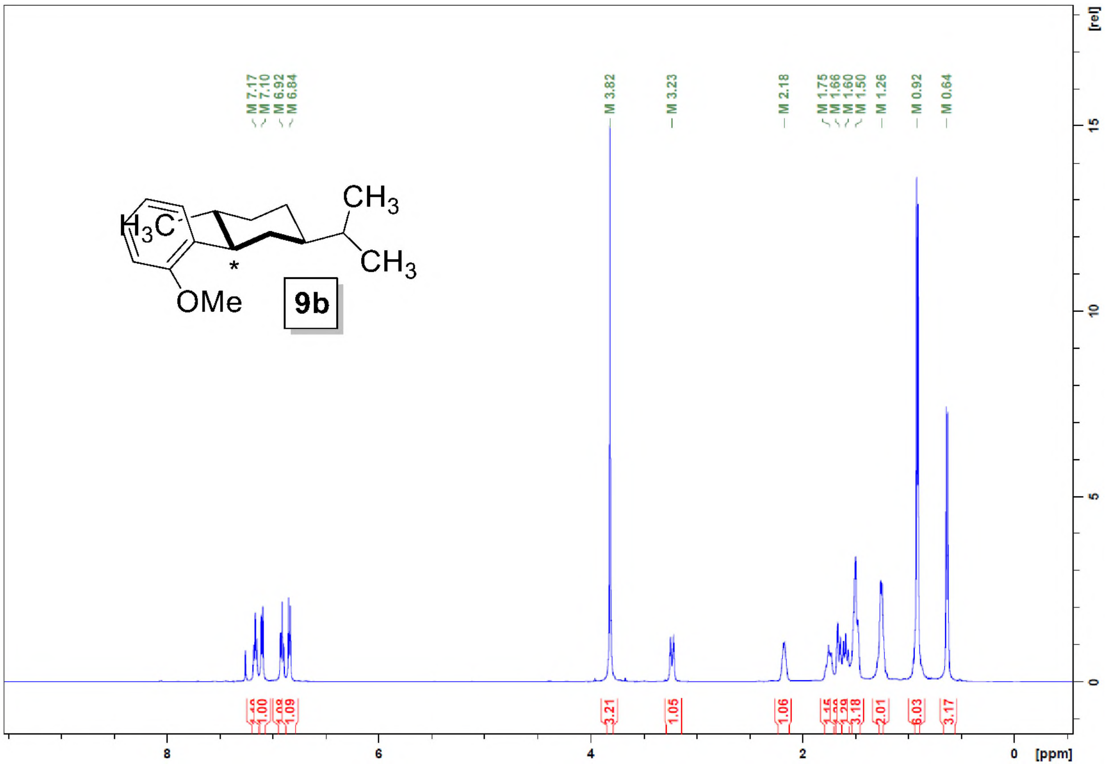
9b
M 7.17
M 7.10
M 6.92
M 6.84
M 3.82
M 3.23
M 2.18
M 1.75
M 1.66
M 1.60
M 1.50
M 1.26
M 0.92
M 0.64
[ppm]
[rel]

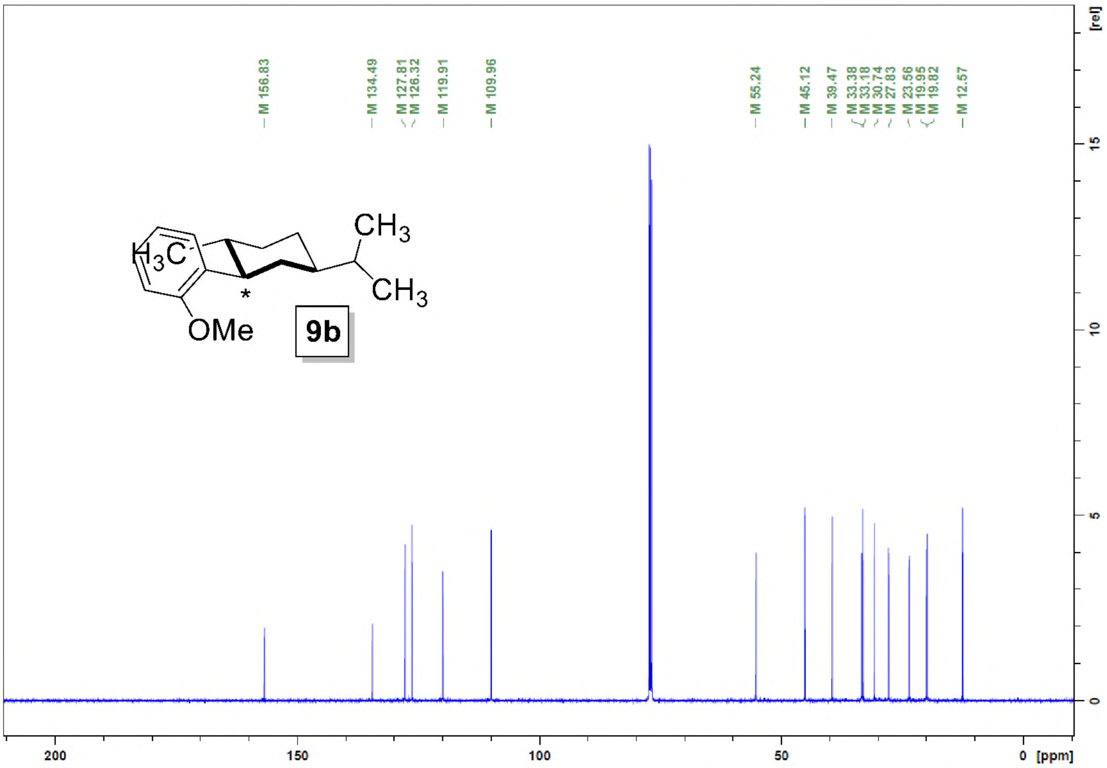
9b
M 156.83
M 134.49
M 127.81
M 126.32
M 119.91
M 109.96
M 55.24
M 45.12
M 39.47
M 33.38
M 33.18
M 30.74
M 27.83
M 23.56
M 19.95
M 19.82
M 12.57
[ppm]
[rel]

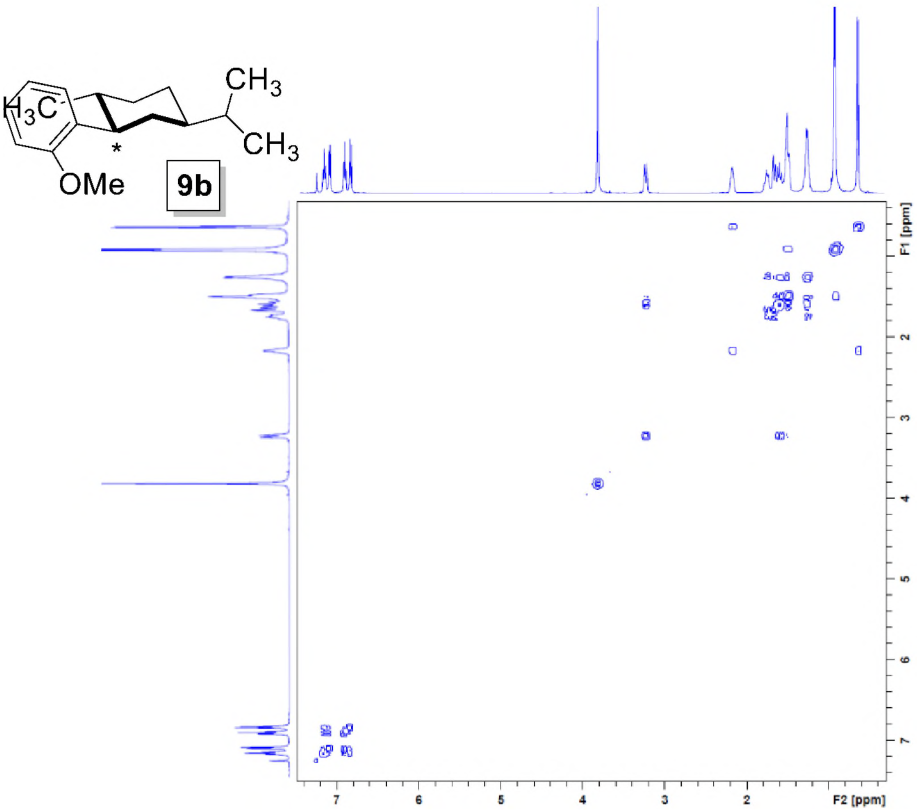

H3C
CH3
CH3
OMe
*
9b
F1 [ppm]
F2 [ppm]


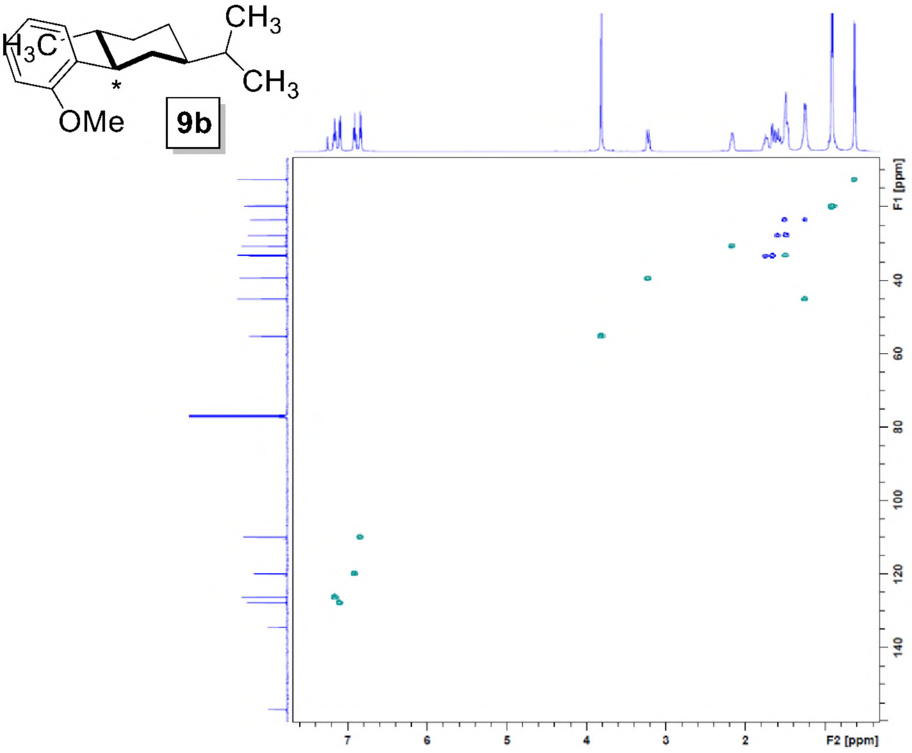

H3C
CH3
CH3
OMe
*
9b
F1 [ppm]
F2 [ppm]

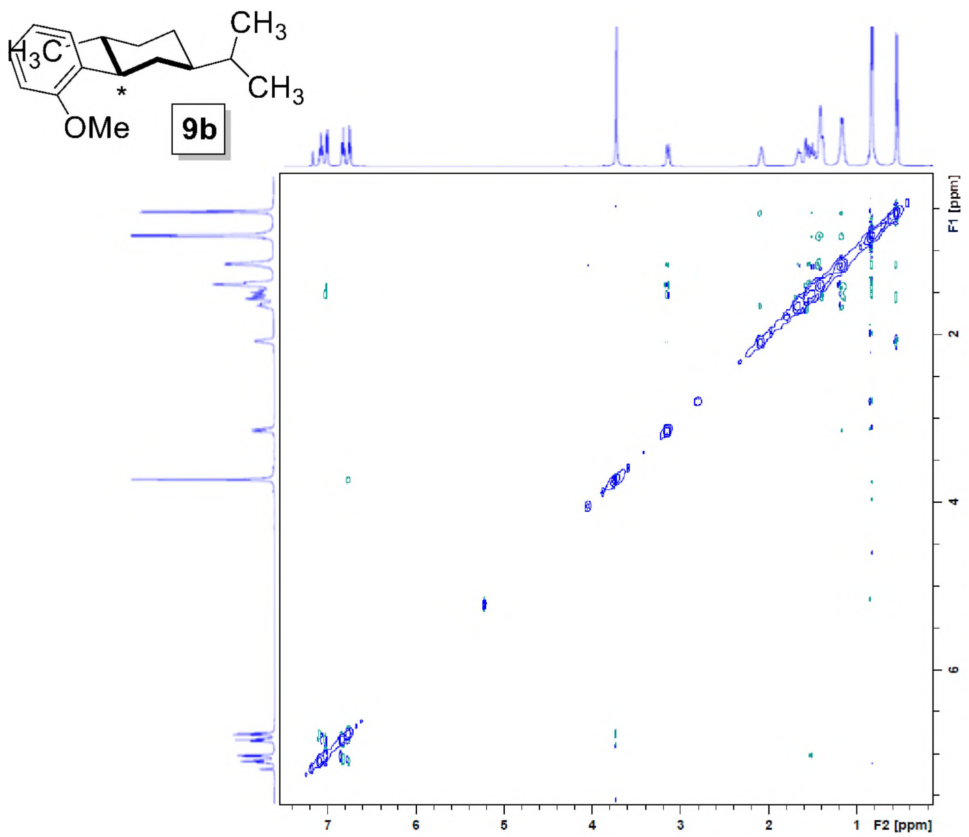
H3C
CH3
CH3
OMe
*
9b
F1 [ppm]
F2 [ppm]
7
6
5
4
3
2
1
2
4
6

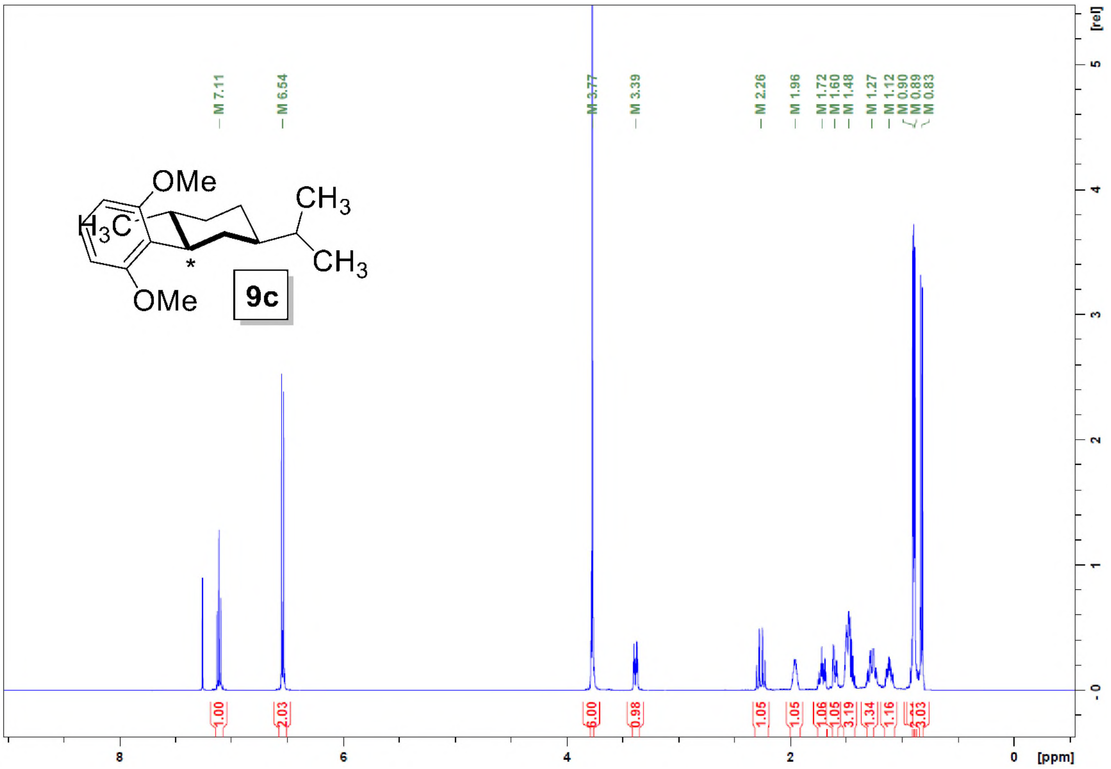
M 7.11
M 6.54
M 3.77
M 3.39
M 2.26
M 1.96
M 1.72
M 1.60
M 1.48
M 1.27
M 1.12
M 0.90
M 0.89
M 0.83
OMe
CH3
H3C
OMe
CH3
9c
1.00
2.03
6.00
0.98
1.05
1.05
1.06
1.05
3.19
1.34
1.16
3.03
[rel]
[ppm]

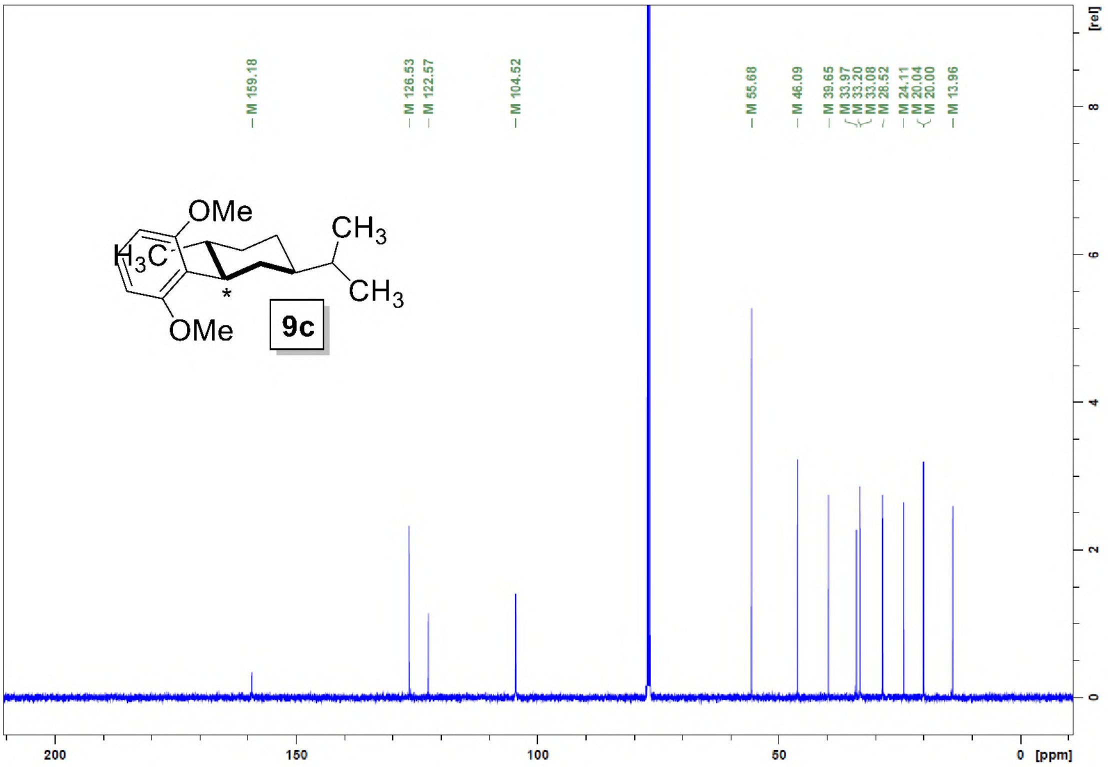
M 159.18
M 126.53
M 122.57
M 104.52
M 55.68
M 46.09
M 39.65
M 33.97
M 33.20
M 33.08
M 28.52
M 24.11
M 20.04
M 20.00
M 13.96
OMe
CH3
H3C
OMe
CH3
9c
[rel]
[ppm]

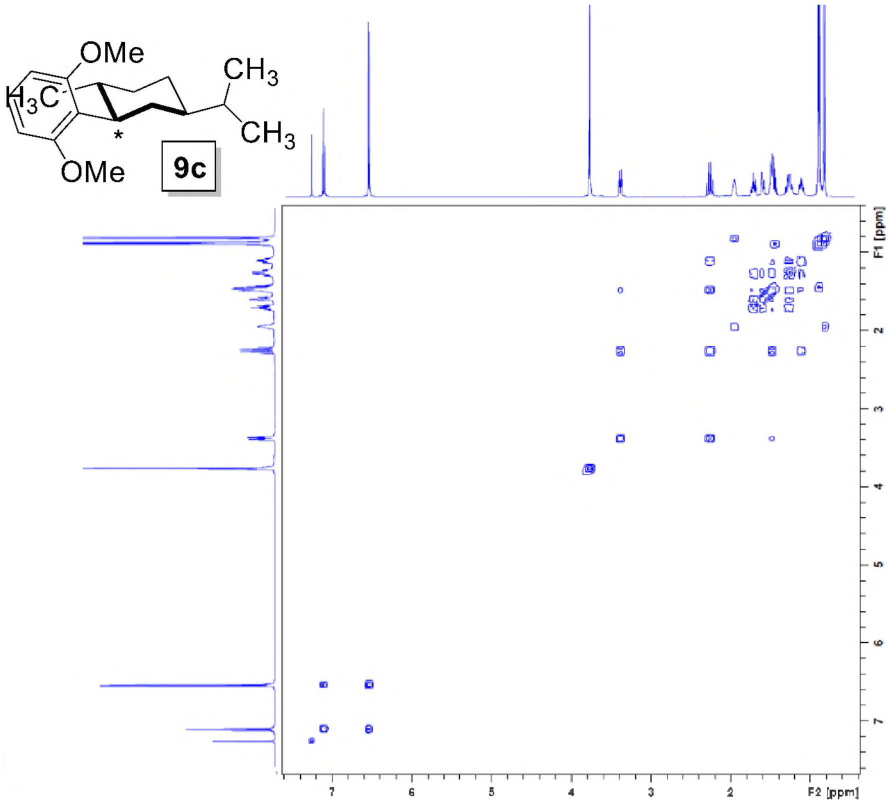
OMe
H3C
CH3
CH3
OMe
9c
F1 [ppm]
F2 [ppm]

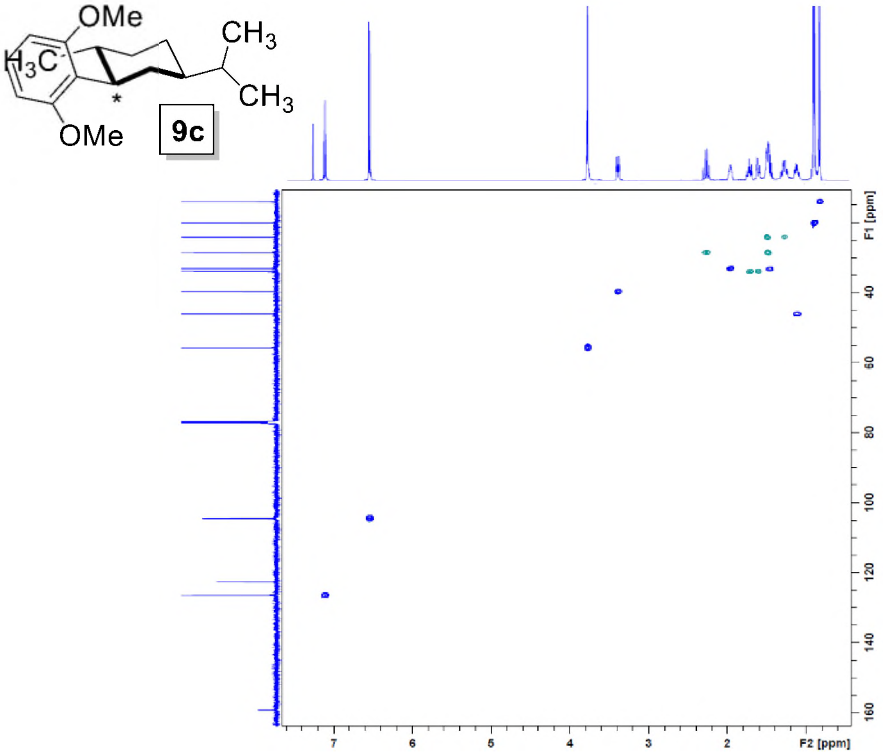
OMe
H3C
CH3
CH3
OMe
9c
F1 [ppm]
F2 [ppm]

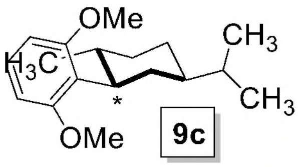


**9c**

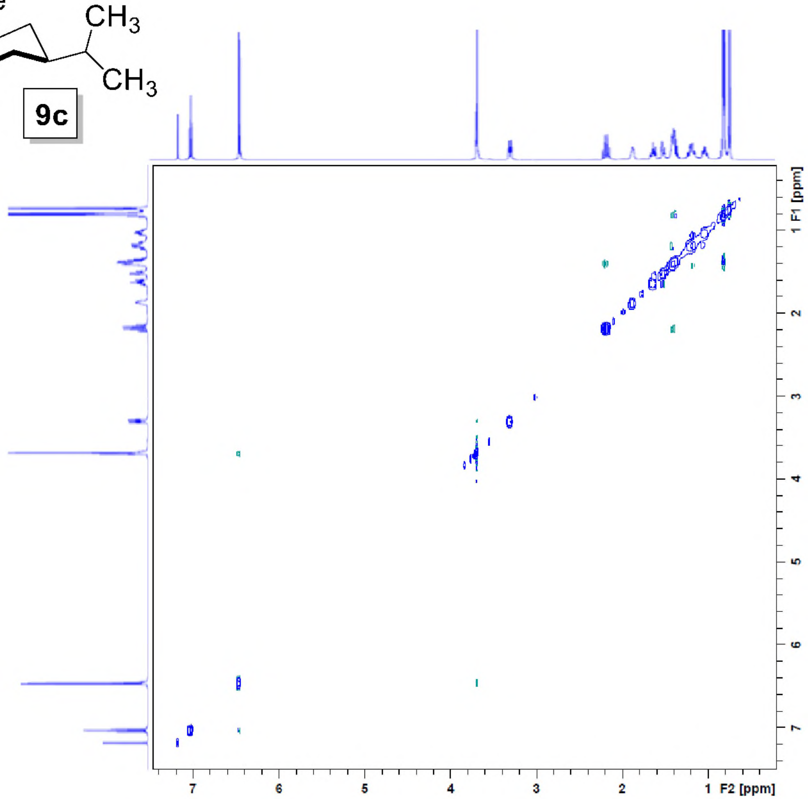

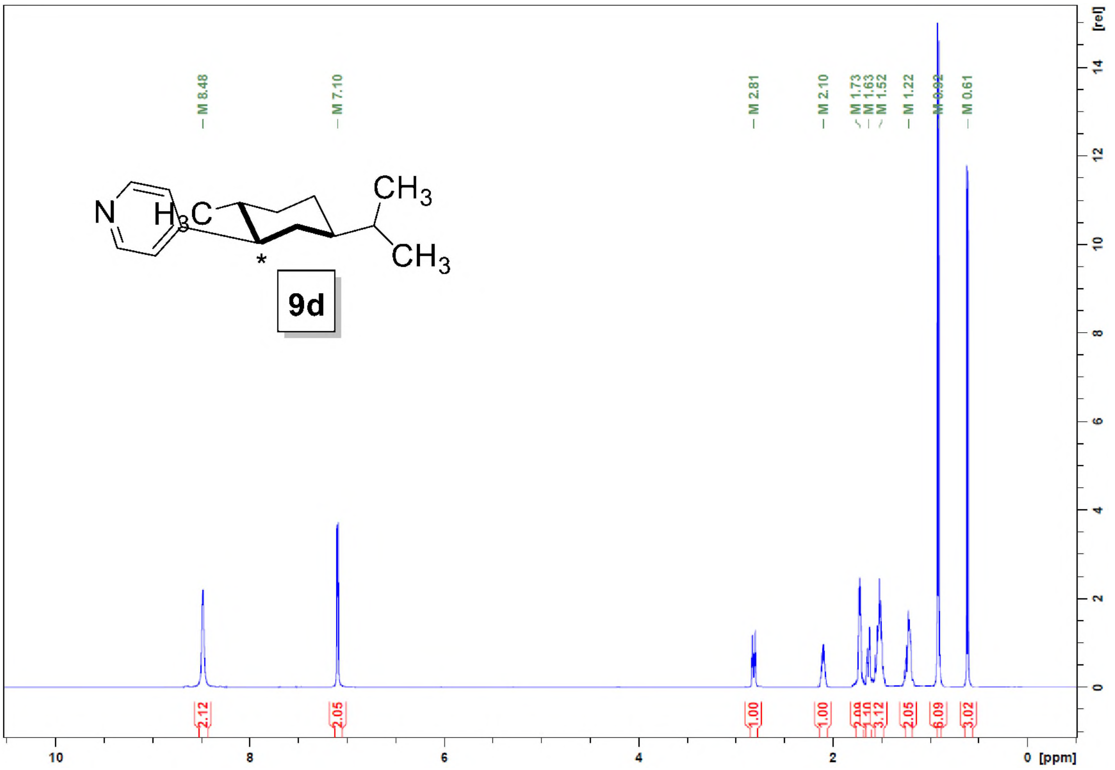

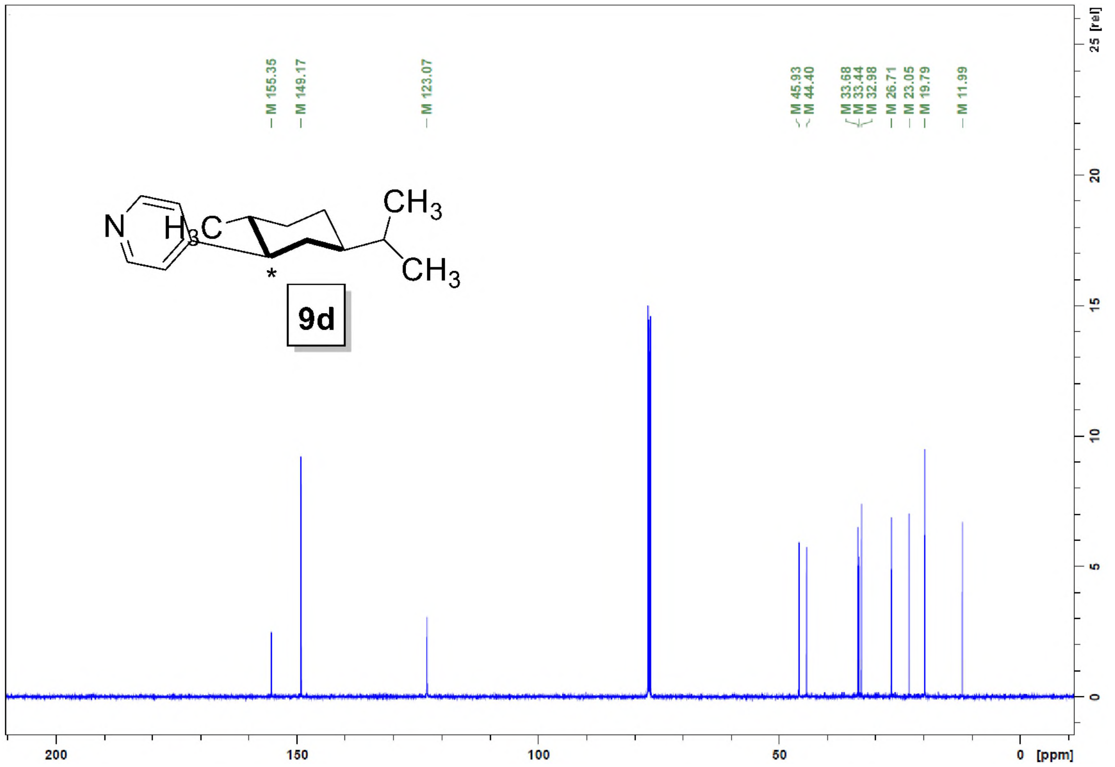

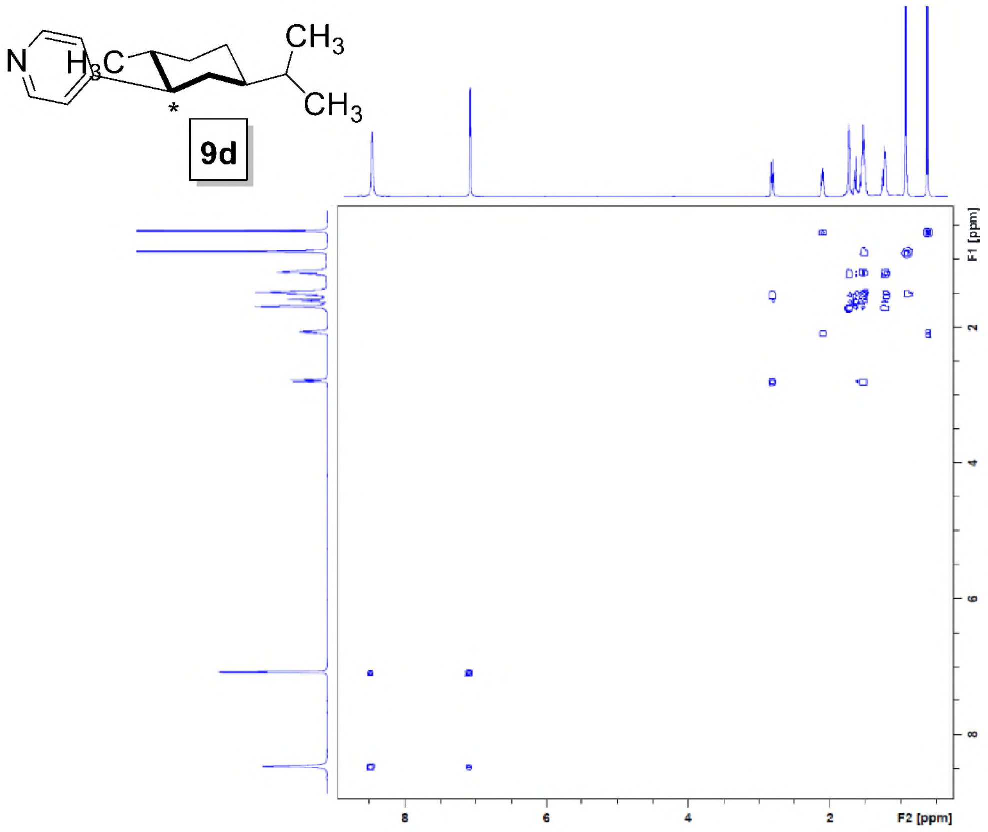
CH3
N
H3C
CH3
9d
F1 [ppm]
F2 [ppm]

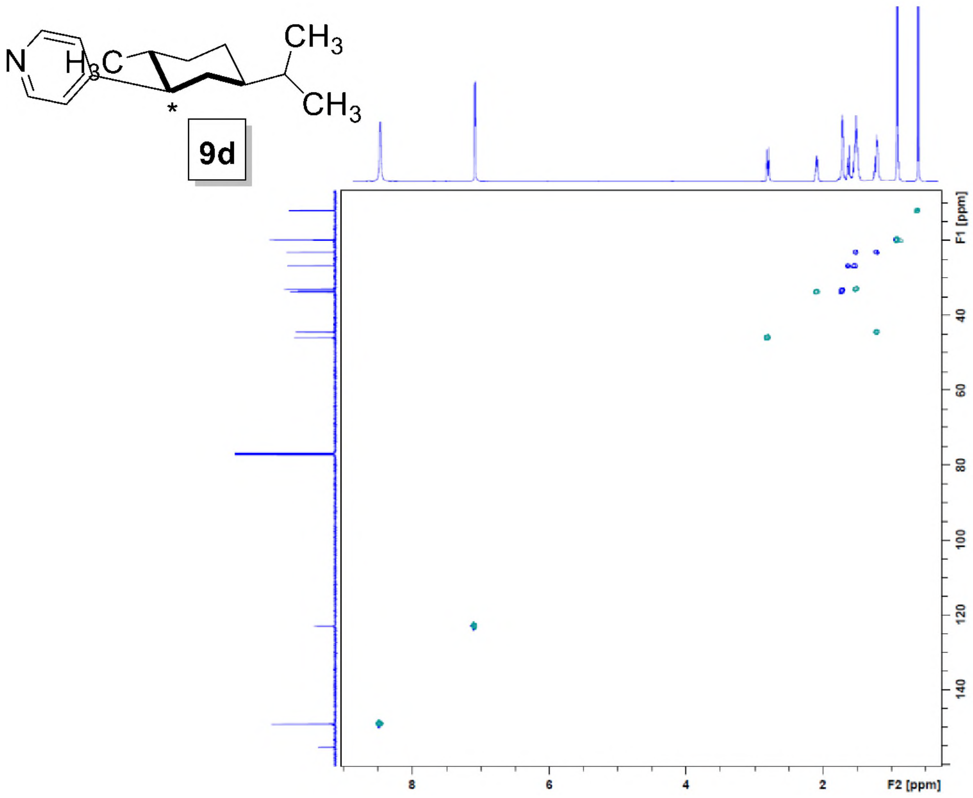
CH3
N
H3C
CH3
9d
F1 [ppm]
F2 [ppm]

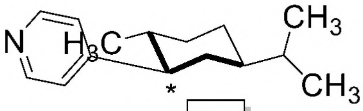


**9d**

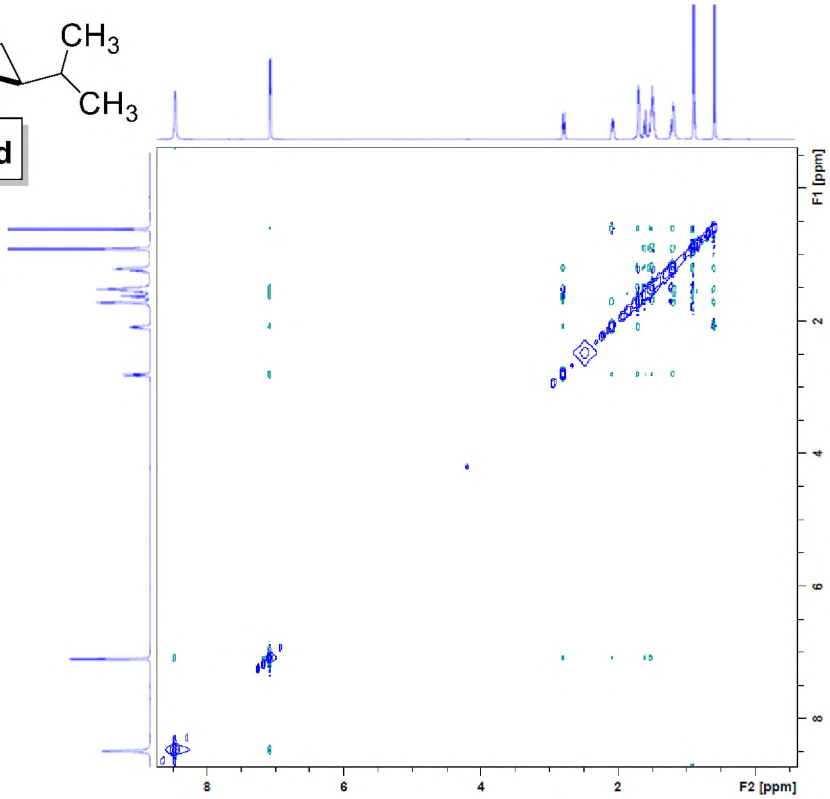

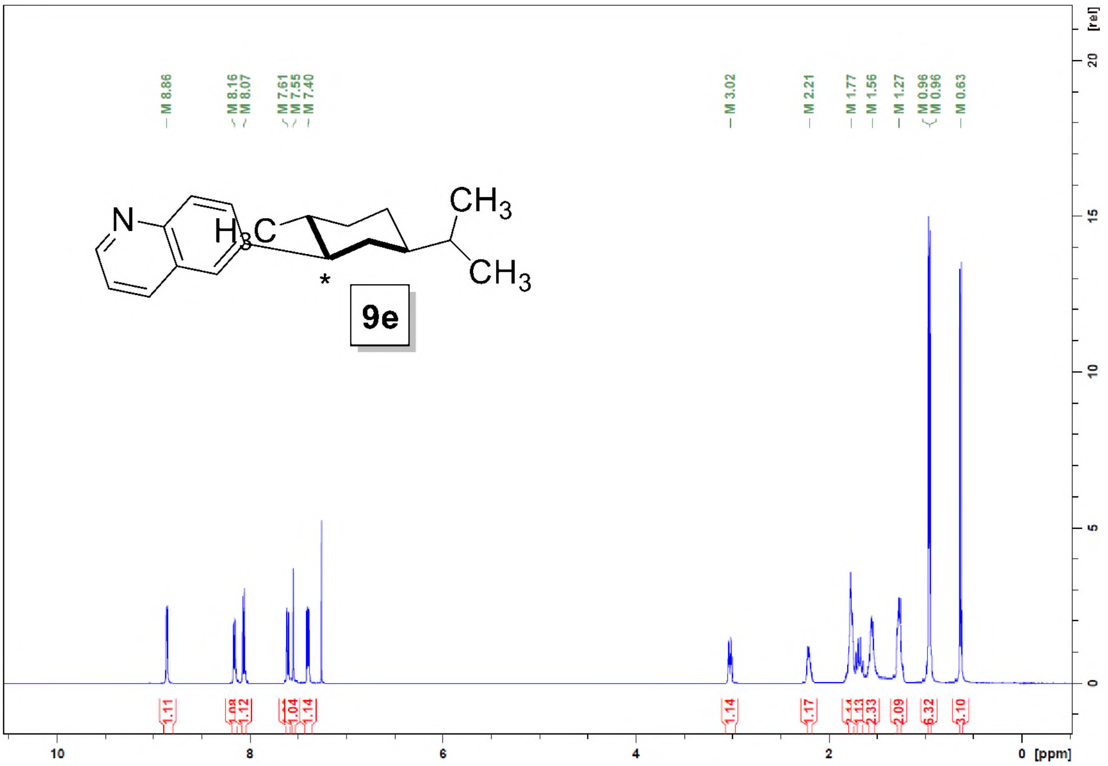
M 8.86
M 8.16
M 8.07
M 7.61
M 7.55
M 7.40
M 3.02
M 2.21
M 1.77
M 1.56
M 1.27
M 0.96
M 0.96
M 0.63
N
$H_3C$
$CH_3$
$CH_3$
*
9e
1.11
1.08
1.12
1.04
1.14
1.14
1.17
2.14
1.13
2.33
2.09
6.32
3.10
[ppm]
[rel]

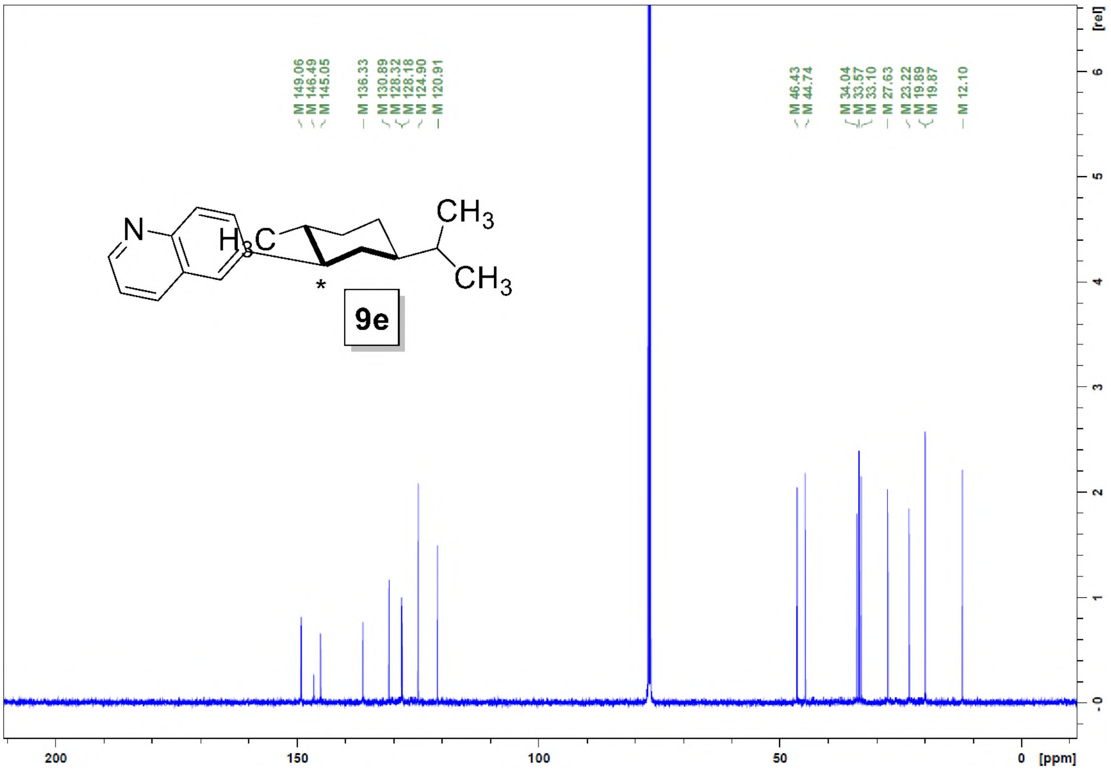
M 149.06
M 146.49
M 145.05
M 136.33
M 130.89
M 128.32
M 128.18
M 124.90
M 120.91
M 46.43
M 44.74
M 34.04
M 33.57
M 33.10
M 27.63
M 23.22
M 19.89
M 19.87
M 12.10
N
$H_3C$
$CH_3$
$CH_3$
*
9e
[ppm]
[rel]

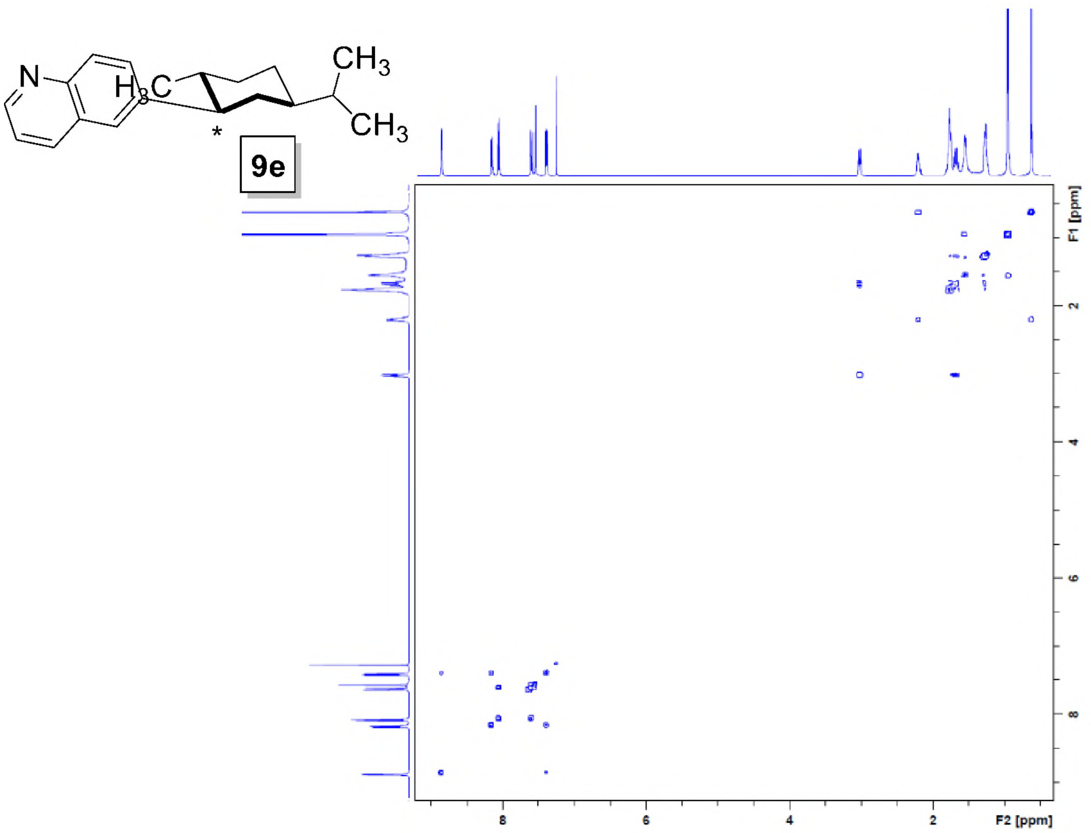
N
H3C
CH3
CH3
*
9e
F1 [ppm]
F2 [ppm]
8
6
4
2

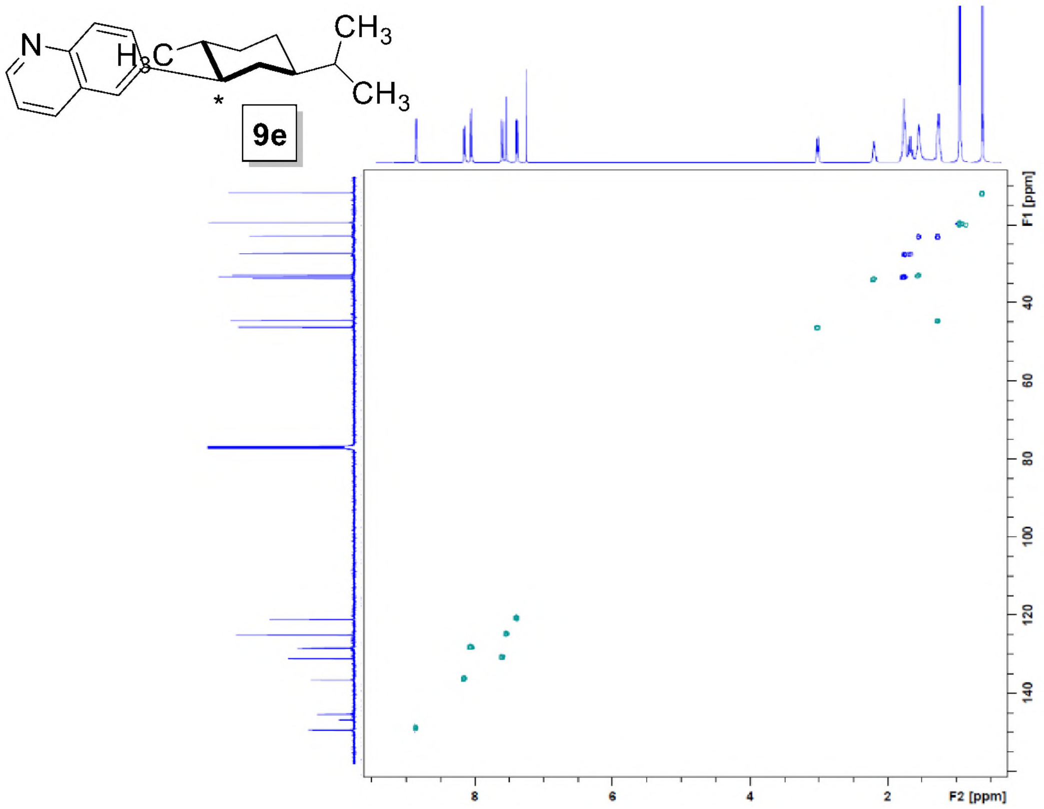
N
H3C
CH3
CH3
*
9e
F1 [ppm]
F2 [ppm]
40
60
80
100
120
140
8
6
4
2

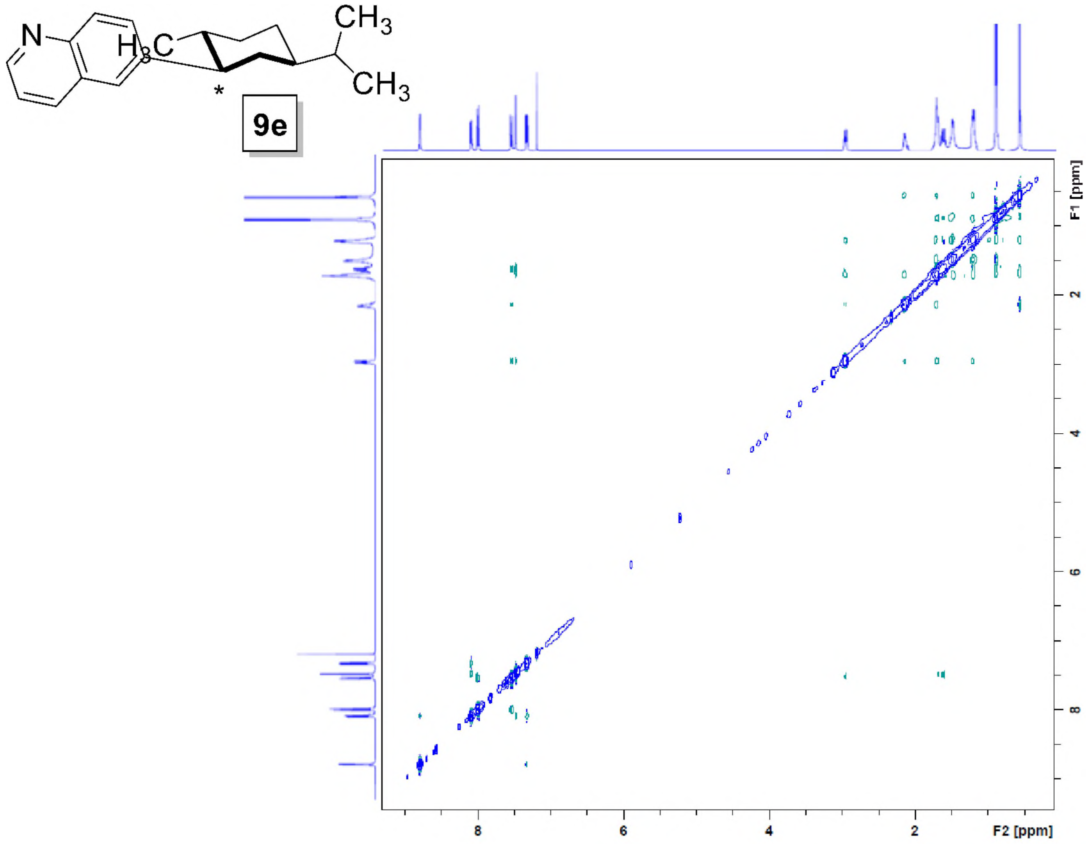

N
H3C
CH3
CH3
*
9e
F1 [ppm]
F2 [ppm]

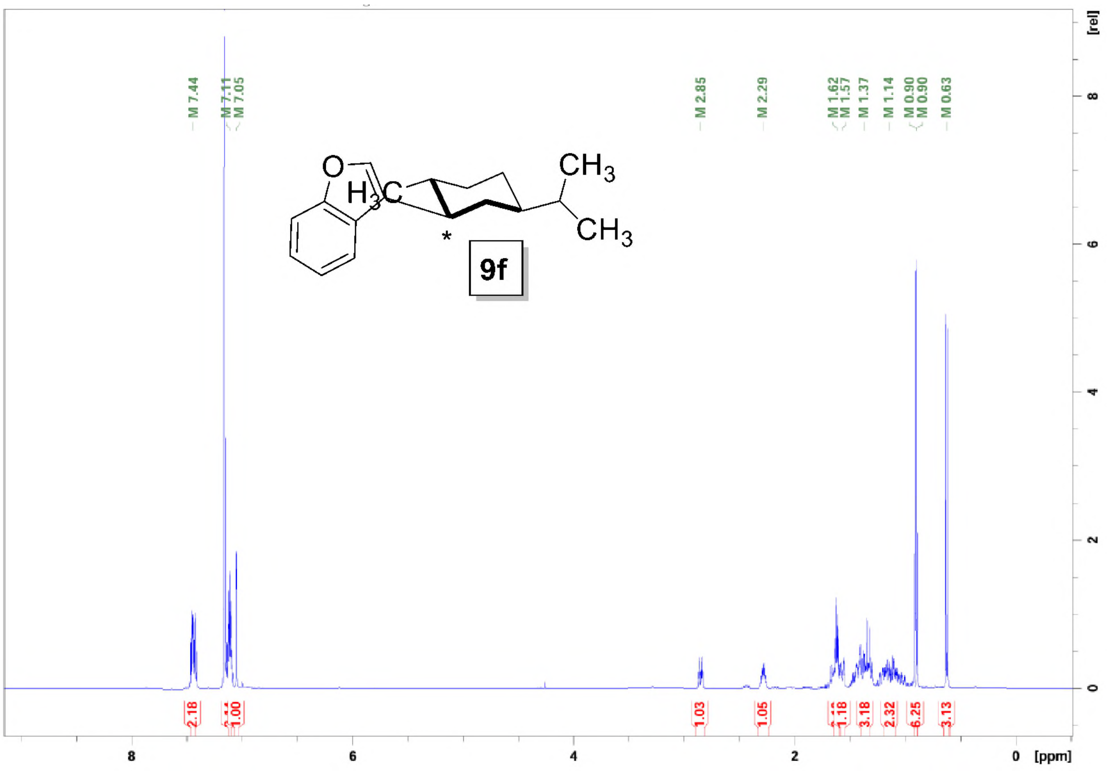

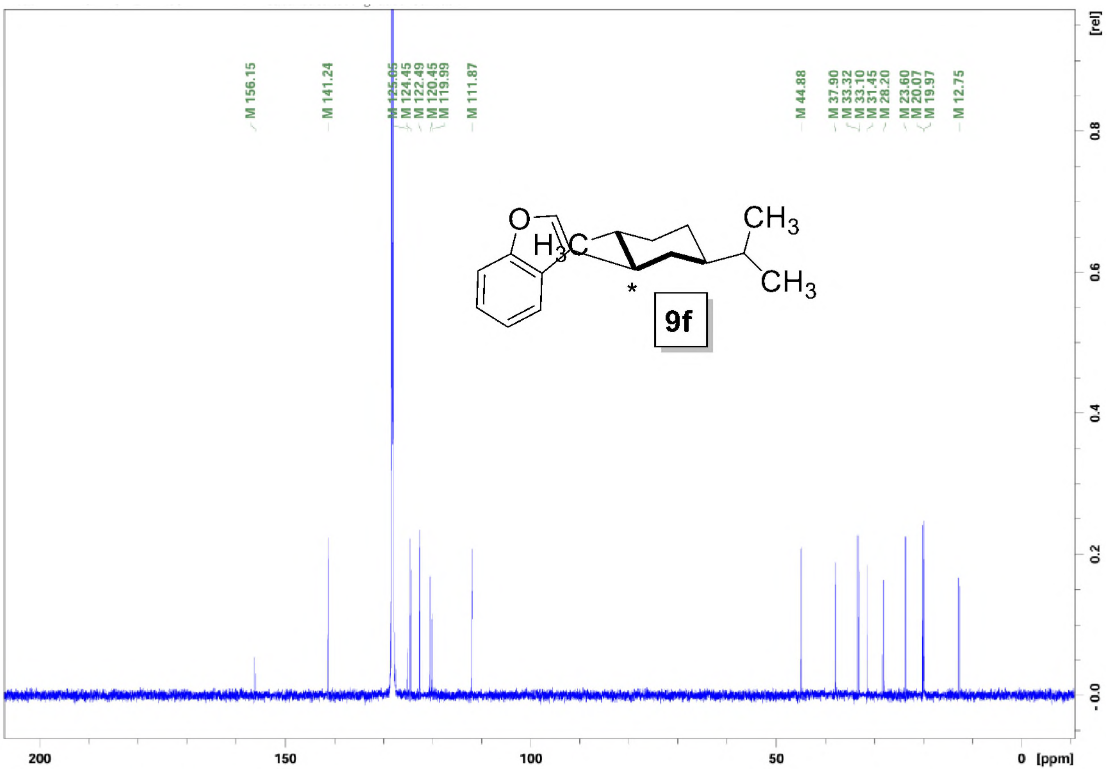

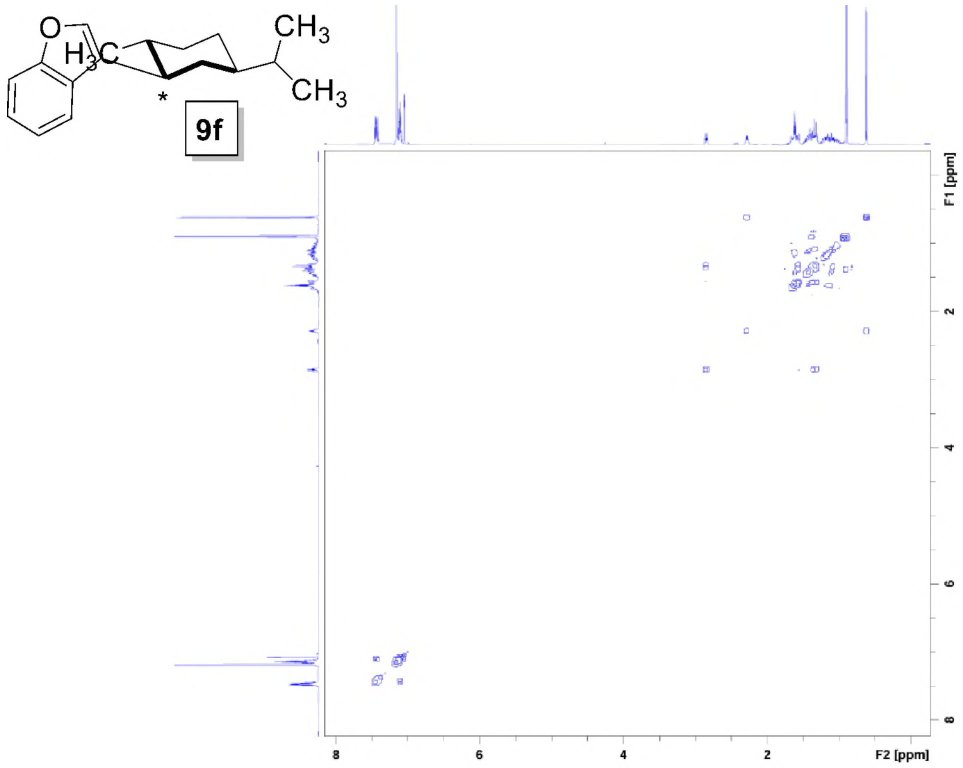

9f
F1 [ppm]
F2 [ppm]


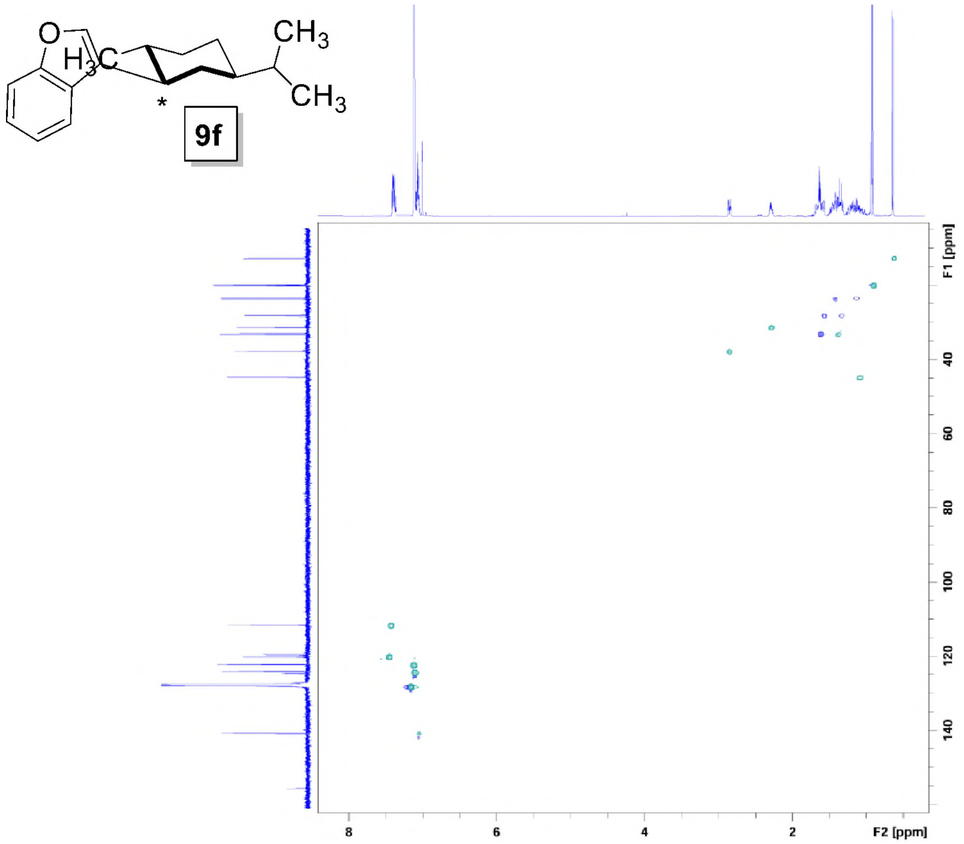

9f
F1 [ppm]
F2 [ppm]

## IV. Calculated $^1$H and $^{13}$C NMR Spectra

The calculations to obtain NMR spectra computationally for two model compounds, **8a** and **9a**, and their diastereomers, **8a'** and **9a'**, believed to be the corresponding minor products in the hydrogenation (not isolated), were conducted using SPARTAN'24 Quantum Mechanics Driver (Win/64), Release 1.3.0, single-point job type, method RWB97X-D, basis set 6-31G(D), number of basis functions 288, number of electrons 120. A SCF model was applied to perform a restricted hybrid HF-DFT SCF calculation, using Pulay DIIS + Geometric Direct Minimization.

- Compound **8a** (isolated) – Calculated NMR chemical shifts

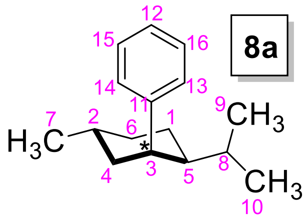


**8a**

| position | δ C | mult | δ H | splitting (Hz) | HMBC(H→C) (2,3-bond) |
|---|---|---|---|---|---|
| 1 | 28.0 | $CH_2$ | 1.93 | dd (12.9, 12.3) | 2, 3, 5, 6, 8 |
| . | | | 2.01 | dd (4.3, 4.1) | 2, 3, 5, 6, 8 |
| 2 | 24.3 | CH | 2.07 | m (12.5, 12.4, $6.4_{x3}$, 2.7) | 1, 3, 4, 6, 7 |
| 3 | 43.6 | CH | 3.26 | ddd (5.3, 5.3, 1.7) | 1, 2, 4, 5, 8, 11, 13, 14 |
| 4 | 41.5 | $CH_2$ | 1.80 | ddd (-13.1, 2.7, 1.7) | 2, 3, 5, 6, 7, 11 |
| . | | | 1.57 | ddd (-13.1, 12.5, 5.3) | 2, 3, 5, 6, 7, 11 |
| 5 | 44.9 | CH | 1.57 | ddd (12.3, 5.3, 4.1) | 1, 3, 4, 6, 8, 9, 10, 11 |
| 6 | 33.6 | $CH_2$ | 1.98 | d (-13.0) | 1, 2, 4, 5, 7 |
| . | | | 1.29 | m (- 13.0, 12.9, 12.4, 4.3) | 1, 2, 4, 5, 7 |
| 7 | 22.7 | $CH_3$ | 0.86 | d (6.4) | 2, 4, 6 |
| 8 | 29.8 | CH | 1.63 | m ($6.4_{x6}$) | 1, 3, 5, 9, 10 |
| 9 | 21.3 | $CH_3$ | 0.94 | d (6.4) | 5, 8, 10 |
| 10 | 20.9 | $CH_3$ | 0.82 | d (6.4) | 5, 8, 9 |
| 11 | 144.1 | C | | | ... |
| 12 | 125.3 | CH | 7.26 | s | 13, 14, 15, 16 |
| 13 | 129.5 | CH | 7.42 | s | 3, 11, 12, 14, 16 |
| 14 | 129.5 | CH | 7.42 | s | 3, 11, 12, 13, 15 |
| 15 | 127.7 | CH | 7.34 | s | 11, 12, 14, 16 |
| 16 | 127.7 | CH | 7.34 | s | 11, 12, 13, 15 |

- Diastereomer **8a'** (not isolated) – Calculated NMR chemical shifts

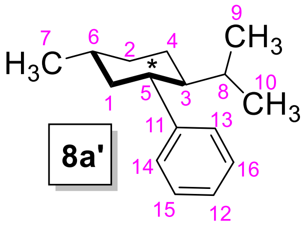

**8a'**

| position | δ C | mult | δ H | splitting (Hz) | HMBC(H→C) (2,3-bond) |
|---|---|---|---|---|---|
| 1 | 43.7 | CH₂ | 1.46 | ddd (-13.7, 12.8, 12.4) | 2, 3, 5, 6, 7, 11 |
| . | | | 1.93 | ddd (-13.7, 3.0, 2.7) | 2, 3, 5, 6, 7, 11 |
| 2 | 33.9 | CH₂ | 1.91 | ddd (-12.9, 3.4, 3.0) | 1, 3, 4, 6, 7 |
| . | | | 1.31 | m (13.3, -12.9, 12.4, 3.3) | 1, 3, 4, 6, 7 |
| 3 | 47.0 | CH | 1.85 | ddd (12.7, 12.0, 2.9) | 1, 2, 4, 5, 8, 9, 10, 11 |
| 4 | 24.4 | CH₂ | 1.87 | dd (-13.0, 3.3) | 2, 3, 5, 6, 8 |
| . | | | 1.47 | m (13.3, -13.0, 12.7, 3.4) | 2, 3, 5, 6, 8 |
| 5 | 48.1 | CH | 2.71 | ddd (12.8, 12.0, 2.7) | 1, 3, 4, 6, 8, 11, 13, 14 |
| 6 | 32.0 | CH | 1.79 | m (12.4, 12.4, 6.4$_{x3}$, 3.0, 3.0) | 1, 2, 4, 5, 7 |
| 7 | 22.5 | CH₃ | 0.98 | d (6.4) | 1, 2, 6 |
| 8 | 27.3 | CH | 1.57 | m (6.4$_{x3}$, 6.4$_{x3}$, 2.9) | 3, 4, 5, 9, 10 |
| 9 | 21.5 | CH₃ | 0.87 | d (6.4) | 3, 8, 10 |
| 10 | 14.9 | CH₃ | 0.78 | d (6.4) | 3, 8, 9 |
| 11 | 144.7 | C | | | ... |
| 12 | 125.5 | CH | 7.24 | s | 13, 14, 15, 16 |
| 13 | 127.0 | CH | 7.27 | s | 5, 11, 12, 14, 16 |
| 14 | 127.0 | CH | 7.27 | s | 5, 11, 12, 13, 15 |
| 15 | 128.0 | CH | 7.33 | s | 11, 12, 14, 16 |
| 16 | 128.0 | CH | 7.33 | s | 11, 12, 13, 15 |

- Compound **9a** (isolated) – Calculated NMR chemical shifts

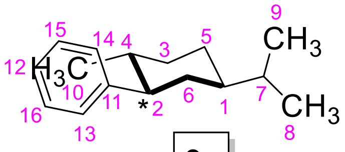


**9a**

| position | δ C | mult | δ H | splitting (Hz) | HMBC(H→C) (2,3-bond) |
|---|---|---|---|---|---|
| **1** | 42.7 | CH | 1.70 | ddd (12.7, 2.6, 2.2) | 2, 3, 5, 6, 7, 8, 9 |
| **2** | 51.7 | CH | 2.25 | ddd (12.8, 11.8, 2.7) | 1, 3, 4, 6, 10, 11, 13, 14 |
| **3** | 34.5 | $CH_2$ | 1.92 | ddd (-13.1, 3.4, 3.1) | 1, 2, 4, 5, 10 |
| . | | | 1.41 | m (13.3, -13.1, 12.4, 3.4) | 1, 2, 4, 5, 10 |
| **4** | 37.5 | CH | 1.91 | m (12.4, 11.8, $6.4_{x3}$) | 2, 3, 5, 6, 10, 11 |
| **5** | 31.8 | $CH_2$ | 1.61 | ddd (-12.5, 3.4, 3.1) | 1, 3, 4, 6, 7 |
| . | | | 1.75 | ddd (13.3, -12.5, 3.4) | 1, 3, 4, 6, 7 |
| **6** | 33.9 | $CH_2$ | 1.89 | ddd (-13.3, 2.7, 2.6) | 1, 2, 4, 5, 7, 11 |
| . | | | 1.54 | ddd (-13.3, 12.8, 12.7) | 1, 2, 4, 5, 7, 11 |
| **7** | 32.4 | CH | 1.85 | m ($6.4_{x3}$, $6.4_{x3}$, 2.2) | 1, 5, 6, 8, 9 |
| **8** | 21.9 | $CH_3$ | 1.01 | d (6.4) | 1, 7, 9 |
| **9** | 15.7 | $CH_3$ | 0.92 | d (6.4) | 1, 7, 8 |
| **10** | 20.7 | $CH_3$ | 0.68 | d (6.4) | 2, 3, 4 |
| **11** | 145.3 | C | | | ... |
| **12** | 125.5 | CH | 7.24 | s | 13, 14, 15, 16 |
| **13** | 127.0 | CH | 7.24 | s | 2, 11, 12, 14, 16 |
| **14** | 127.0 | CH | 7.24 | s | 2, 11, 12, 13, 15 |
| **15** | 128.0 | CH | 7.32 | s | 11, 12, 14, 16 |
| **16** | 128.0 | CH | 7.32 | s | 11, 12, 13, 15 |

- Diastereomer **9a'** (not isolated) – Calculated NMR chemical shifts

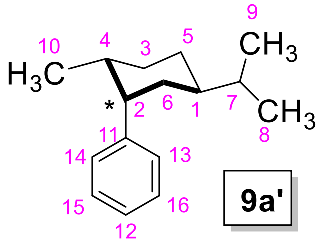


**9a'**

| position | δ C | mult | δ H | splitting (Hz) | HMBC(H→C) (2,3-bond) |
|---|---|---|---|---|---|
| **1** | 37.8 | CH | 1.92 | m (12.4, 12.4, 11.6, $3.9_{x2}$) | 2, 3, 5, 6, 7, 8, 9 |
| **2** | 46.5 | CH | 2.96 | ddd (5.9, 4.7, 1.4) | 1, 3, 4, 6, 10, 11, 13, 14 |
| **3** | 28.4 | $CH_2$ | 1.57 | m (-14.7, 3.8, 3.0, 2.9) | 1, 2, 4, 5, 10 |
| . | | | 1.90 | ddd (-14.7, 13.1, 3.8) | 1, 2, 4, 5, 10 |
| **4** | 34.1 | CH | 1.98 | m ($6.4_{x3}$, 4.7, 3.0) | 2, 3, 5, 6, 10, 11 |
| **5** | 30.3 | $CH_2$ | 2.28 | m (-12.8, 3.9, 3.8, 2.9) | 1, 3, 4, 6, 7 |
| . | | | 1.24 | m (13.1, -12.8, 12.4, 3.8) | 1, 3, 4, 6, 7 |
| **6** | 36.4 | $CH_2$ | 2.28 | ddd (-13.2, 3.9, 1.4) | 1, 2, 4, 5, 7, 11 |
| . | | | 1.50 | ddd (-13.2, 12.4, 5.9) | 1, 2, 4, 5, 7, 11 |
| **7** | 34.7 | CH | 1.40 | m (11.6, $6.4_{x6}$) | 1, 5, 6, 8, 9 |
| **8** | 21.1 | $CH_3$ | 0.91 | d (6.4) | 1, 7, 9 |
| **9** | 21.2 | $CH_3$ | 1.05 | d (6.4) | 1, 7, 8 |
| **10** | 20.6 | $CH_3$ | 0.65 | d (6.4) | 2, 3, 4 |
| **11** | 143.5 | C | | | ... |
| **12** | 125.6 | CH | 7.27 | s | 13, 14, 15, 16 |
| **13** | 129.2 | CH | 7.42 | d (7.7) | 2, 11, 12, 14, 16 |
| **14** | 129.2 | CH | 7.42 | d (7.3) | 2, 11, 12, 13, 15 |
| **15** | 127.6 | CH | 7.32 | d (7.3) | 11, 12, 14, 16 |
| **16** | 127.6 | CH | 7.32 | d (7.7) | 11, 12, 13, 15 |

## V. ADMET Computational Study of Synthesized Compounds and Known CB1/2 Ligands

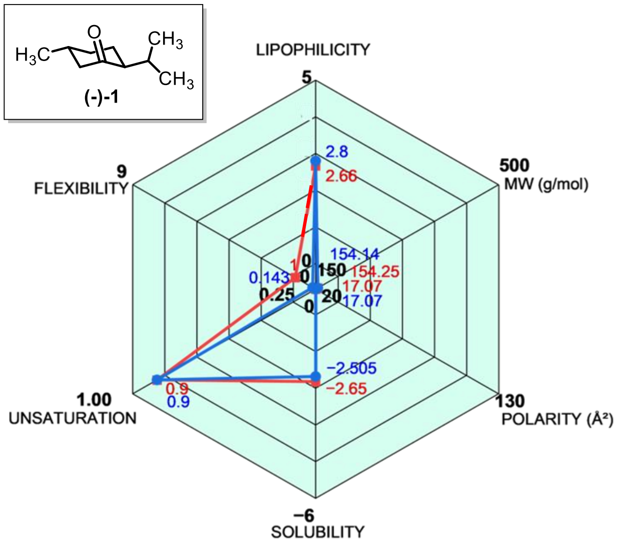


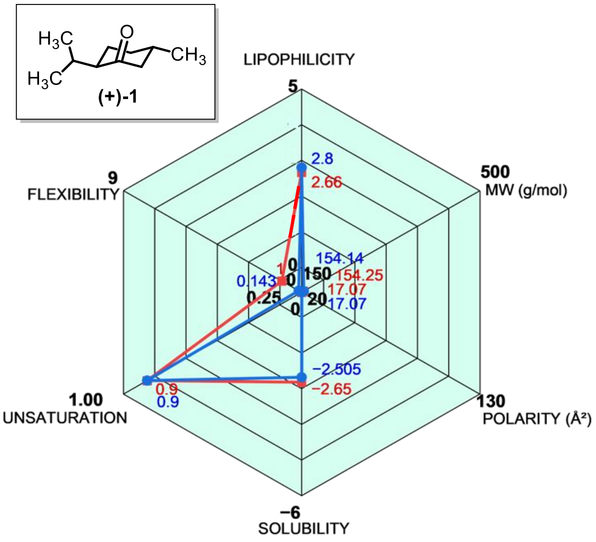


| Compound | (-)-1 | (+)-1 |
|---|---|---|
| BBB permeability | Yes/Yes | Yes/Yes |
| Skin permeability (cm.s$^{-1}$) | -5.08/-1.909 | -5.08/-1.909 |
| Intestinal absorption | Yes/Yes/Yes | Yes/Yes/Yes |
| P-gp substrate | No/No | No/No |
| Lipinski's rule of five | Yes/Yes | Yes/Yes |

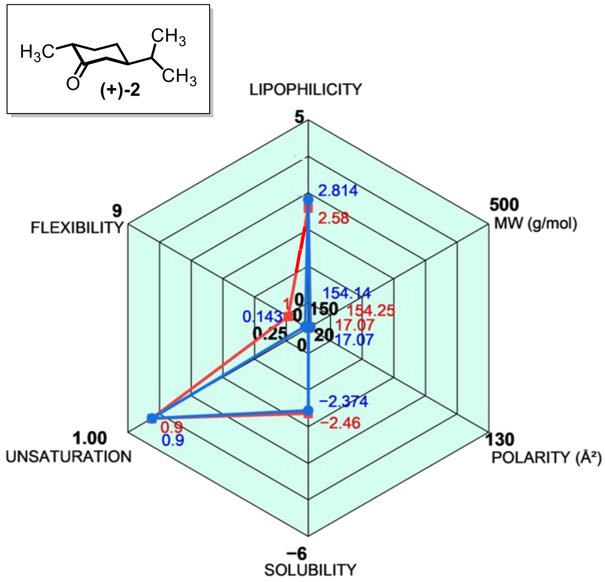


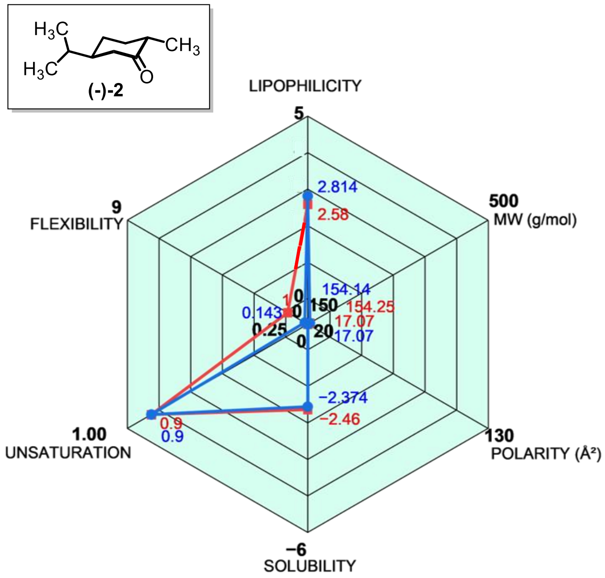


| Compound | (+)-2 | (-)-2 |
|---|---|---|
| BBB permeability | Yes/Yes | Yes/Yes |
| Skin permeability (cm.s$^{-1}$) | -5.30/-1.909 | -5.30/-1.909 |
| Intestinal absorption | Yes/Yes/Yes | Yes/Yes/Yes |
| P-gp substrate | No/No | No/No |
| Lipinski's rule of five | Yes/Yes | Yes/Yes |

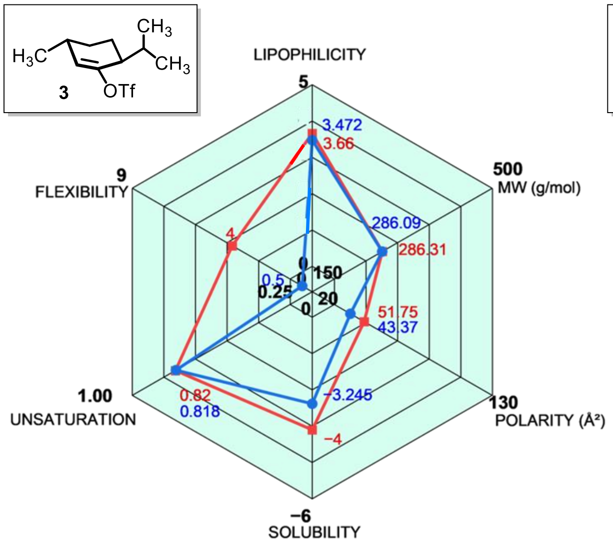


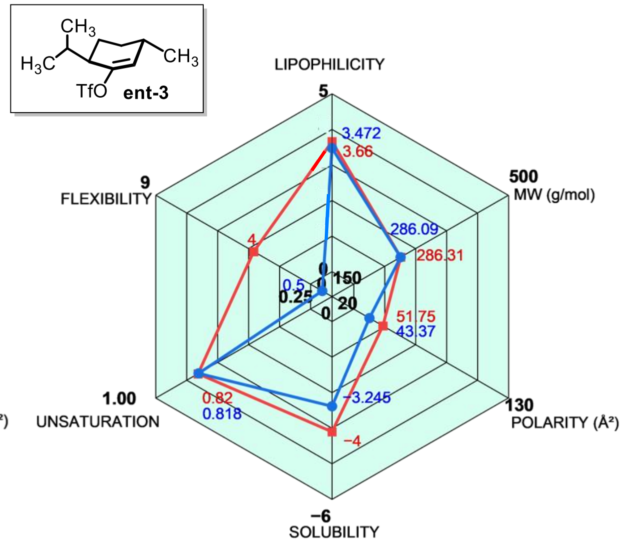


| Compound | 3 | ent-3 |
|---|---|---|
| BBB permeability | No/Yes | No/Yes |
| Skin permeability (cm.s$^{-1}$) | -5.06/-2.343 | -5.06/-2.343 |
| Intestinal absorption | Yes/Yes/Yes | Yes/Yes/Yes |
| P-gp substrate | No/No | No/No |
| Lipinski’s rule of five | Yes/Yes | Yes/Yes |

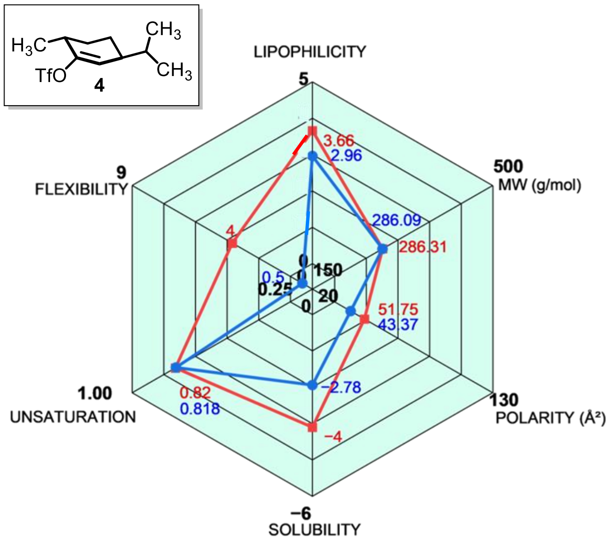


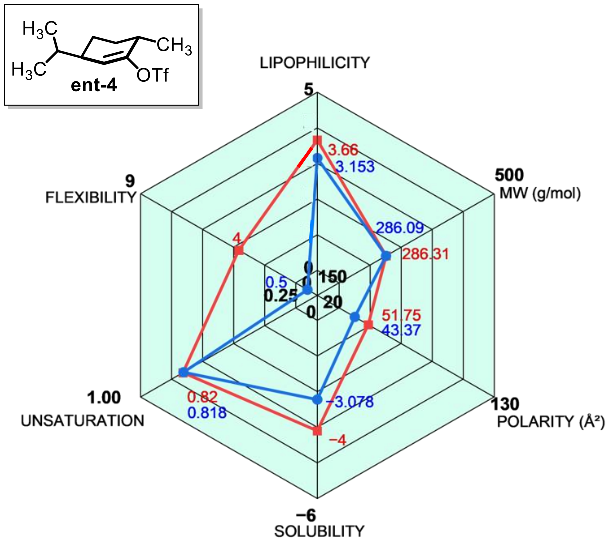


| Compound | 4 | ent-4 |
|---|---|---|
| BBB permeability | No/Yes | No/Yes |
| Skin permeability (cm.s$^{-1}$) | -5.06/-2.339 | -5.06/-2.339 |
| Intestinal absorption | Yes/Yes/Yes | Yes/Yes/Yes |
| P-gp substrate | No/No | No/No |
| Lipinski’s rule of five | Yes/Yes | Yes/Yes |

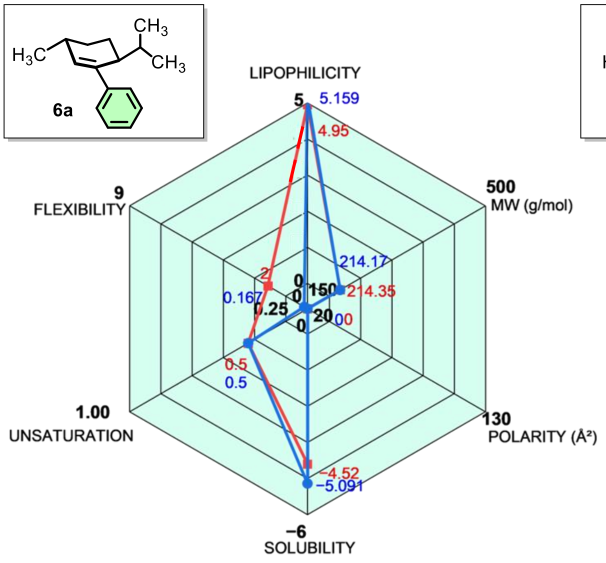


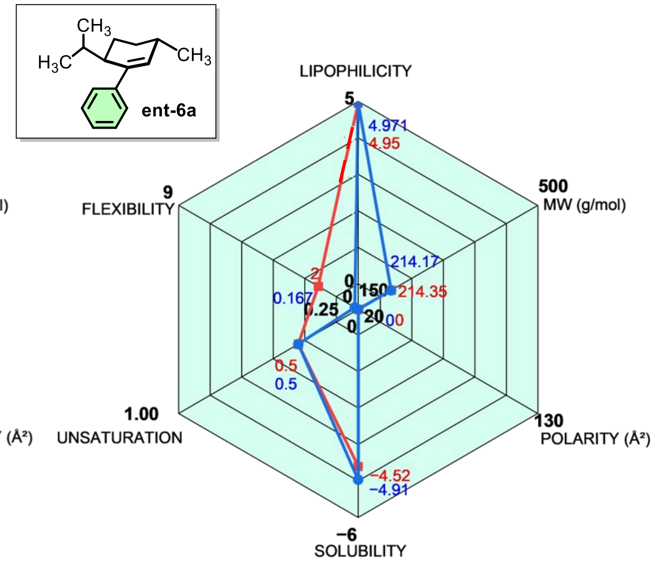


| Compound | 6a | ent-6a |
|---|---|---|
| BBB permeability | No/Yes | No/Yes |
| Skin permeability (cm.s$^{-1}$) | -3.99/-1.506 | -3.99/-1.506 |
| Intestinal absorption | Yes/Yes/Yes | Yes/Yes/Yes |
| P-gp substrate | No/No | No/No |
| Lipinski's rule of five | Yes/Yes | Yes/Yes |

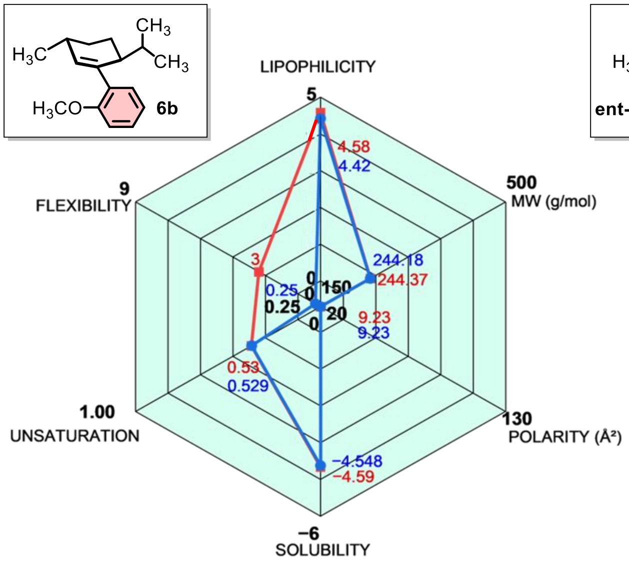


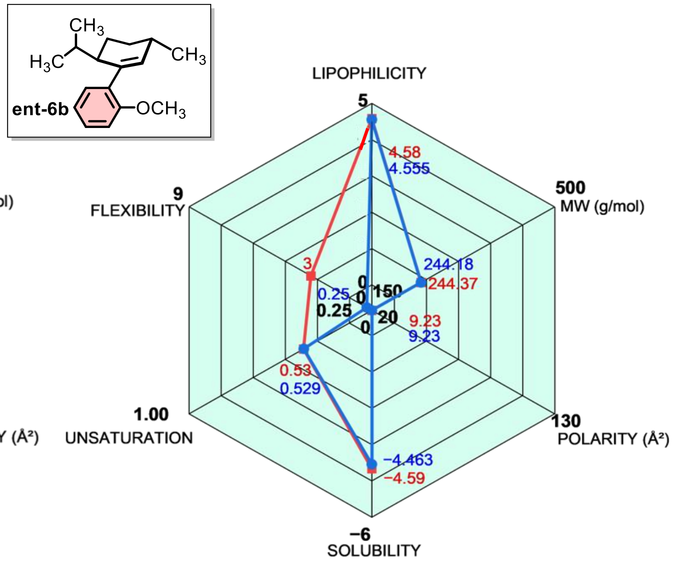


| Compound | 6b | ent-6b |
|---|---|---|
| BBB permeability | Yes/No | Yes/No |
| Skin permeability (cm.s$^{-1}$) | -4.2/-1.7 | -4.2/-1.7 |
| Intestinal absorption | Yes/Yes/Yes | Yes/Yes/Yes |
| P-gp substrate | No/No | No/No |
| Lipinski's rule of five | Yes/Yes | Yes/Yes |

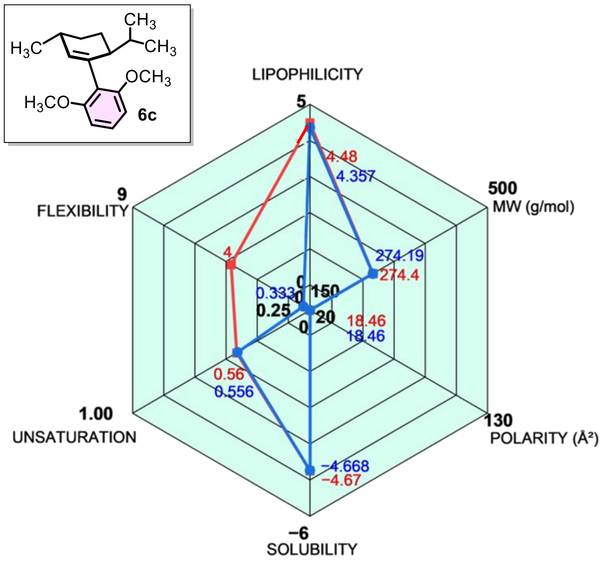


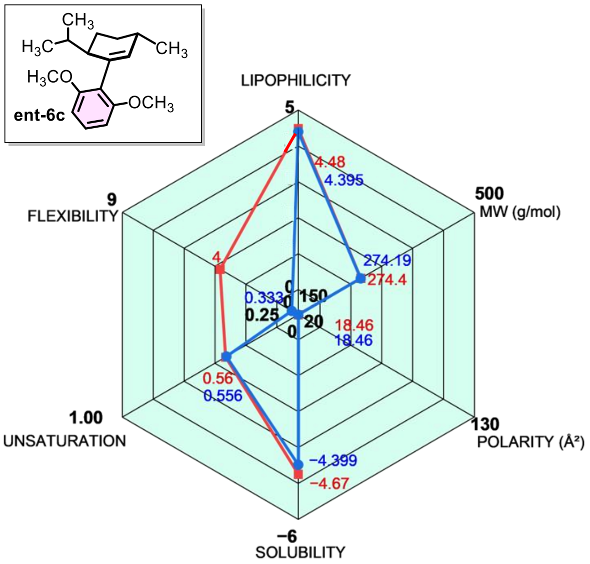


| Compound | 6c | ent-6c |
|---|---|---|
| BBB permeability | Yes/Yes | Yes/Yes |
| Skin permeability (cm.s$^{-1}$) | -4.4/-2.146 | -4.4/-2.146 |
| Intestinal absorption | Yes/Yes/Yes | Yes/Yes/Yes |
| P-gp substrate | No/No | No/No |
| Lipinski's rule of five | Yes/Yes | Yes/Yes |

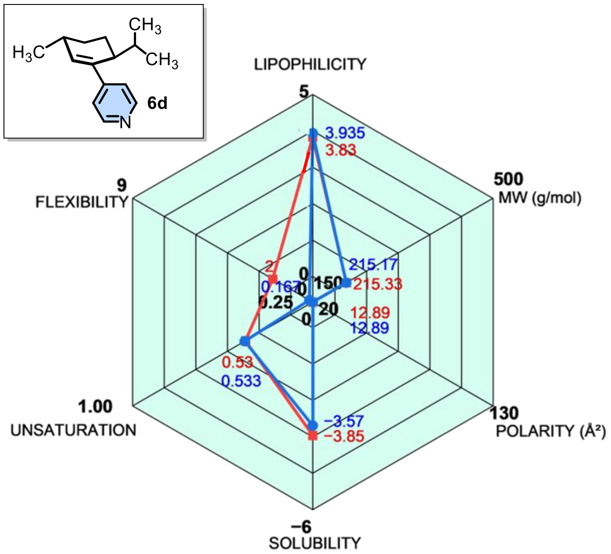


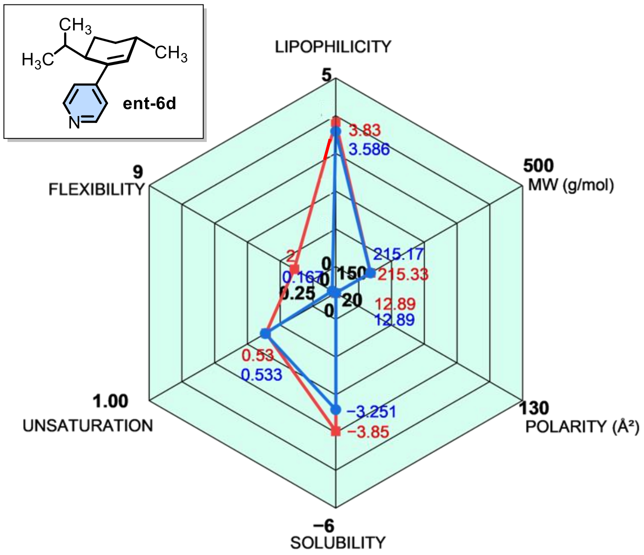


| Compound | 6d | ent-6d |
|---|---|---|
| BBB permeability | Yes/Yes | Yes/Yes |
| Skin permeability (cm.s$^{-1}$) | -4.76/-1.513 | -4.76/-1.513 |
| Intestinal absorption | Yes/Yes/Yes | Yes/Yes/Yes |
| P-gp substrate | No/No | No/No |
| Lipinski's rule of five | Yes/Yes | Yes/Yes |

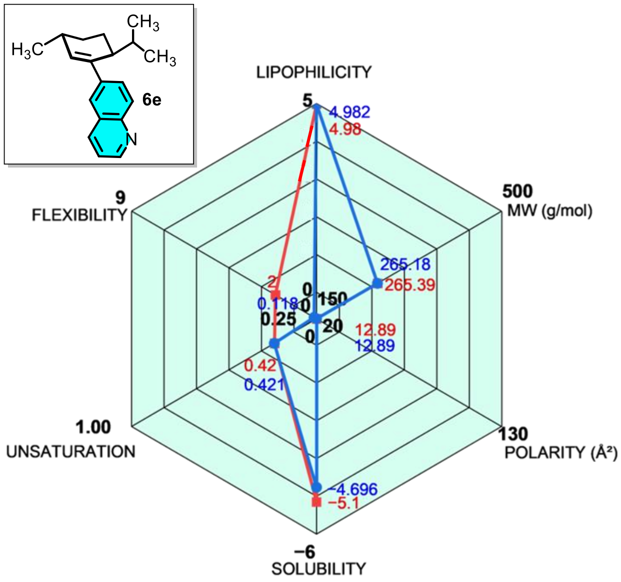


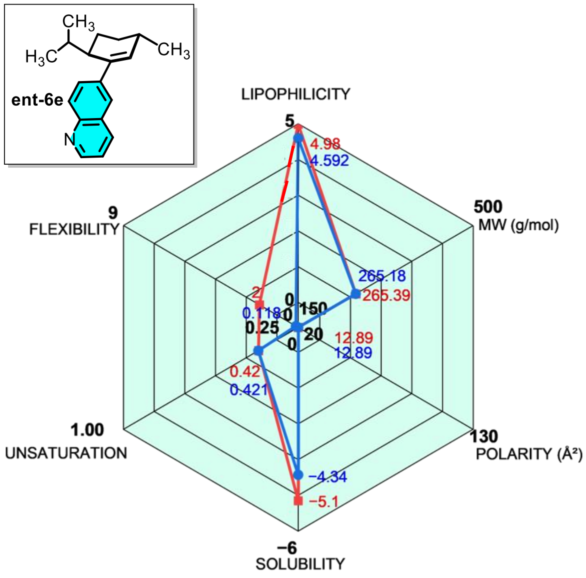


| Compound | 6e | ent-6e |
|---|---|---|
| BBB permeability | Yes/Yes | Yes/Yes |
| Skin permeability (cm.s$^{-1}$) | -4.11/-1.95 | -4.11/-1.95 |
| Intestinal absorption | Yes/Yes/Yes | Yes/Yes/Yes |
| P-gp substrate | No/No | No/No |
| Lipinski's rule of five | Yes/Yes | Yes/Yes |

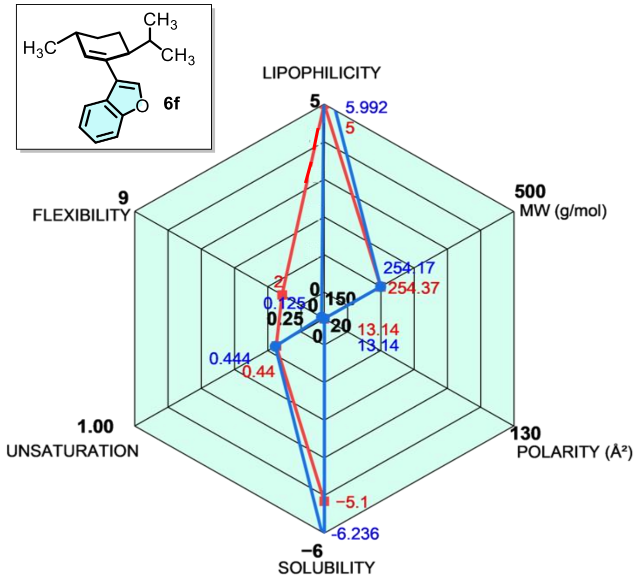


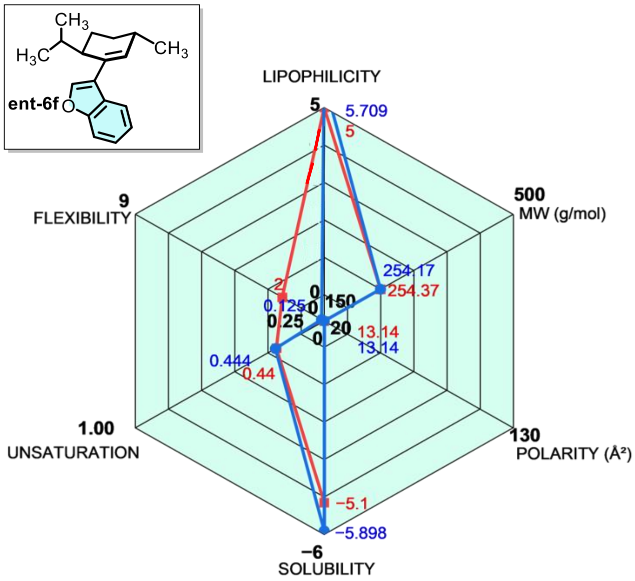


| Compound | 6f | ent-6f |
|---|---|---|
| BBB permeability | No/Yes | No/Yes |
| Skin permeability (cm.s$^{-1}$) | -3.95/-1.854 | -3.95/-1.854 |
| Intestinal absorption | Yes/Yes/Yes | Yes/Yes/Yes |
| P-gp substrate | No/No | No/No |
| Lipinski's rule of five | Yes/Yes | Yes/Yes |

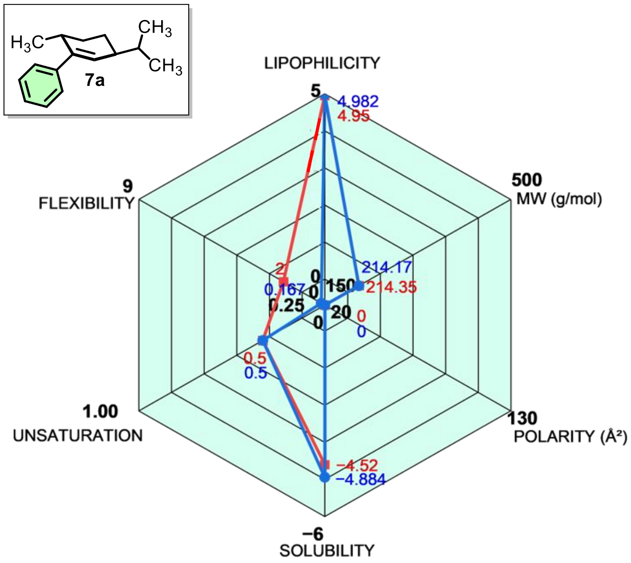


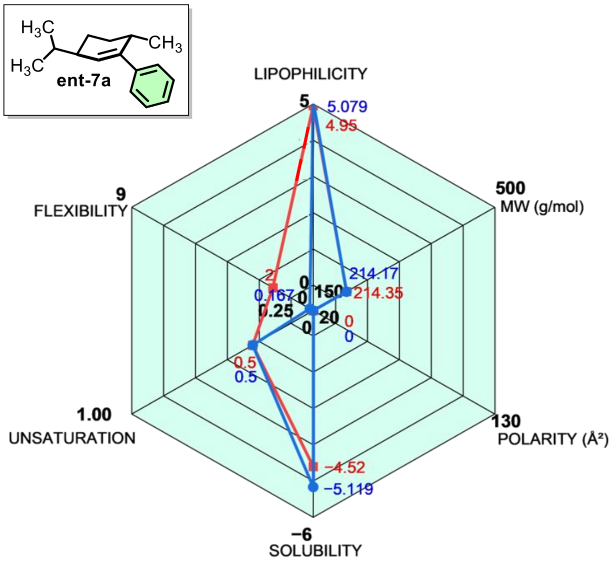


| Compound | 7a | ent-7a |
|---|---|---|
| BBB permeability | No/Maybe | No/Maybe |
| Skin permeability (cm.s$^{-1}$) | -3.99/-1.456 | -3.99/-1.456 |
| Intestinal absorption | Yes/Yes/Yes | Yes/Yes/Yes |
| P-gp substrate | No/No | No/No |
| Lipinski's rule of five | Yes/Yes | Yes/Yes |

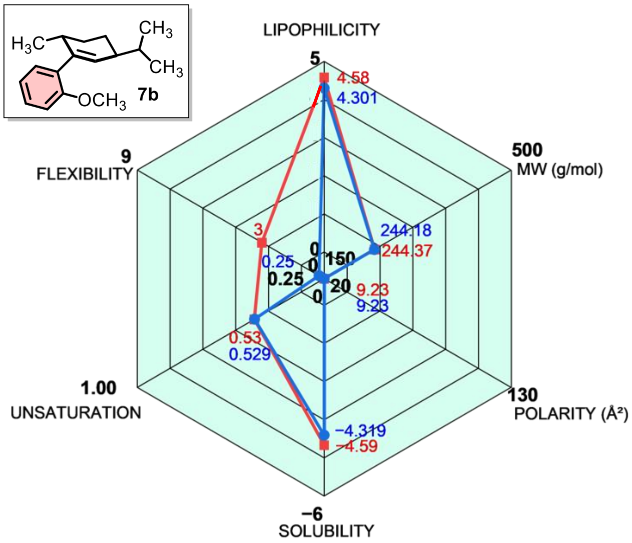


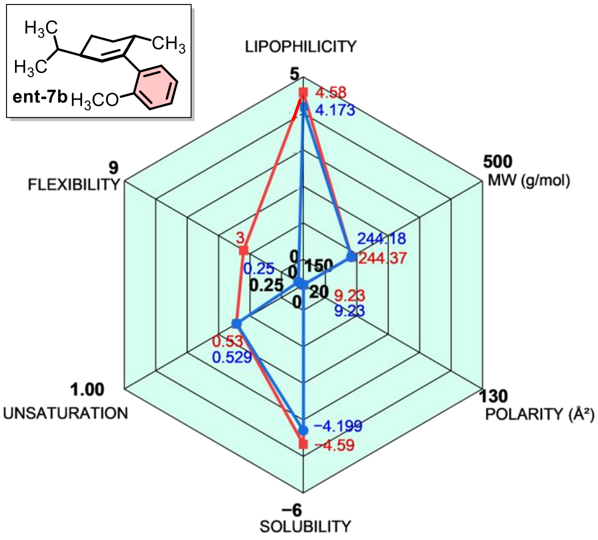


| Compound | 7b | ent-7b |
|---|---|---|
| BBB permeability | Yes/Yes | Yes/Yes |
| Skin permeability (cm.s$^{-1}$) | -4.2/-1.992 | -4.2/-1.992 |
| Intestinal absorption | Yes/Yes/Yes | Yes/Yes/Yes |
| P-gp substrate | No/No | No/No |
| Lipinski's rule of five | Yes/Yes | Yes/Yes |

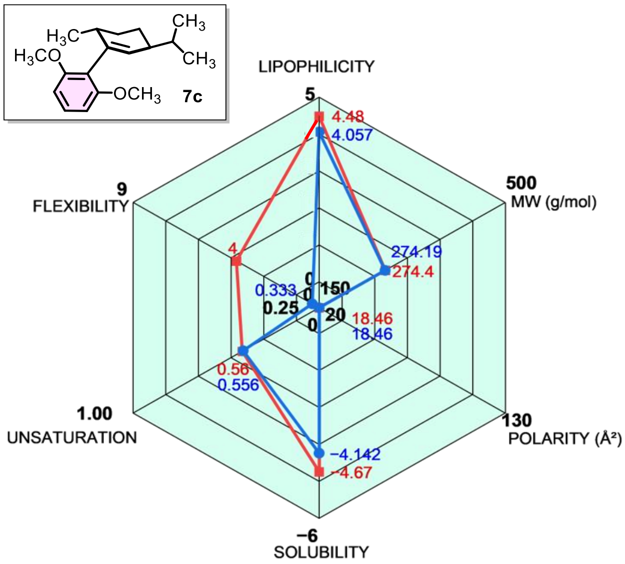


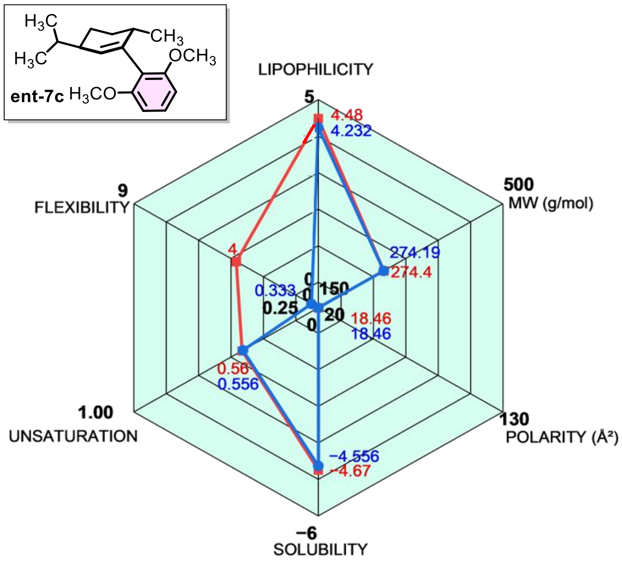


| Compound | 7c | ent-7c |
|---|---|---|
| BBB permeability | Yes/Yes | Yes/Yes |
| Skin permeability (cm.s$^{-1}$) | -4.4/-2.263 | -4.4/-2.263 |
| Intestinal absorption | Yes/Yes/Yes | Yes/Yes/Yes |
| P-gp substrate | No/No | No/No |
| Lipinski's rule of five | Yes/Yes | Yes/Yes |

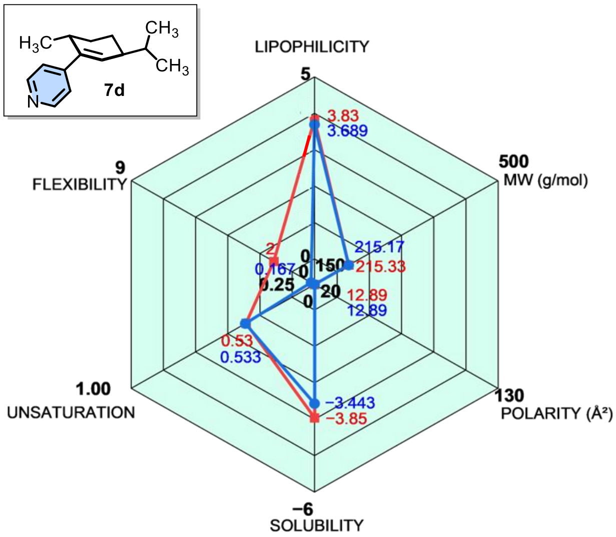


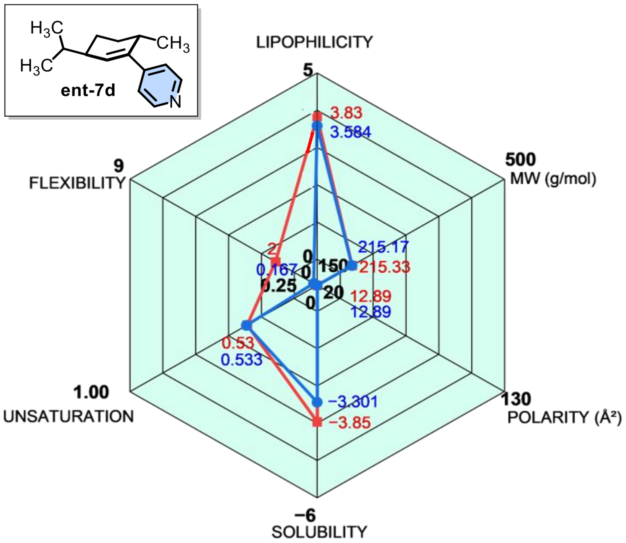


| Compound | 7d | ent-7d |
|---|---|---|
| BBB permeability | Yes/Yes | Yes/Yes |
| Skin permeability (cm.s$^{-1}$) | -4.76/-1.545 | -4.76/-1.545 |
| Intestinal absorption | Yes/Yes/Yes | Yes/Yes/Yes |
| P-gp substrate | No/No | No/No |
| Lipinski's rule of five | Yes/Yes | Yes/Yes |

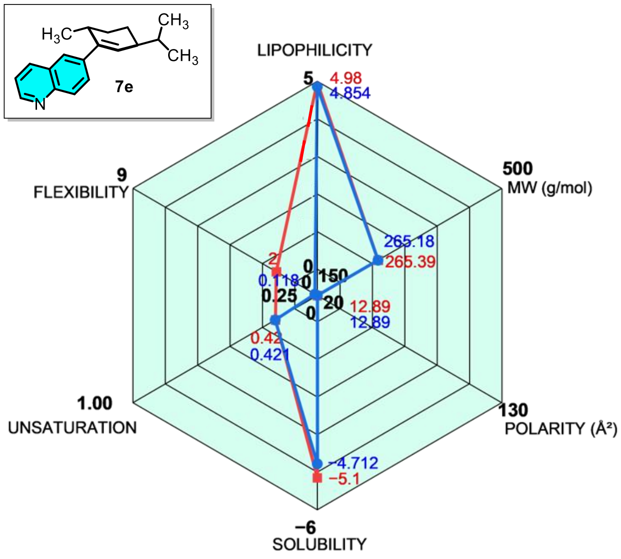


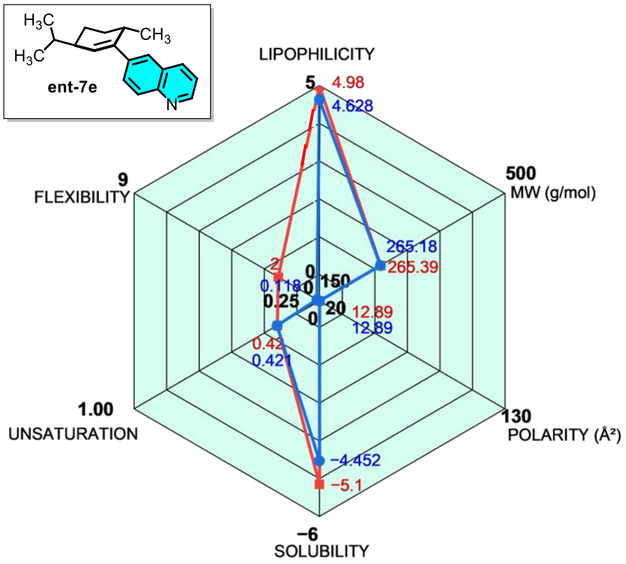


| Compound | 7e | ent-7e |
|---|---|---|
| BBB permeability | Yes/Yes | Yes/Yes |
| Skin permeability (cm.s$^{-1}$) | -4.11/-1.919 | -4.11/-1.919 |
| Intestinal absorption | Yes/Yes/Yes | Yes/Yes/Yes |
| P-gp substrate | No/No | No/No |
| Lipinski's rule of five | Yes/Yes | Yes/Yes |

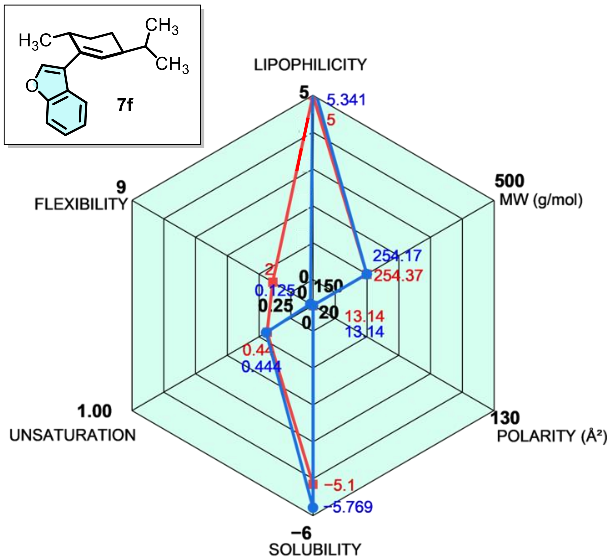


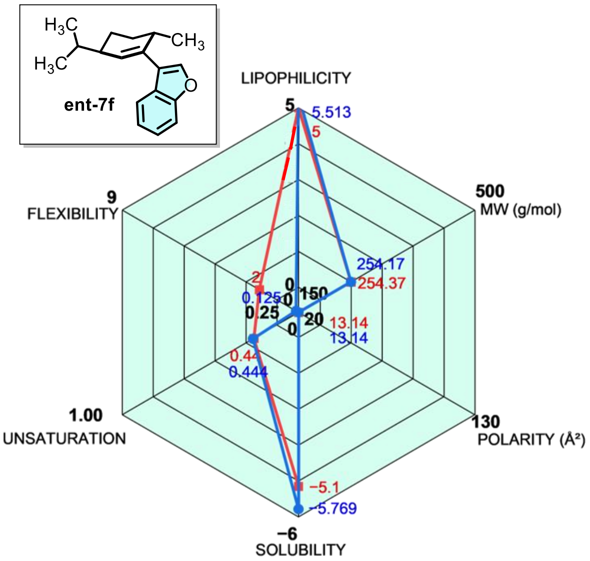


| Compound | 7f | ent-7f |
|---|---|---|
| BBB permeability | No/Maybe | No/Maybe |
| Skin permeability (cm.s$^{-1}$) | -3.95/-1.897 | -3.95/-1.897 |
| Intestinal absorption | Yes/Yes/Yes | Yes/Yes/Yes |
| P-gp substrate | No/No | No/No |
| Lipinski's rule of five | Yes/Yes | Yes/Yes |

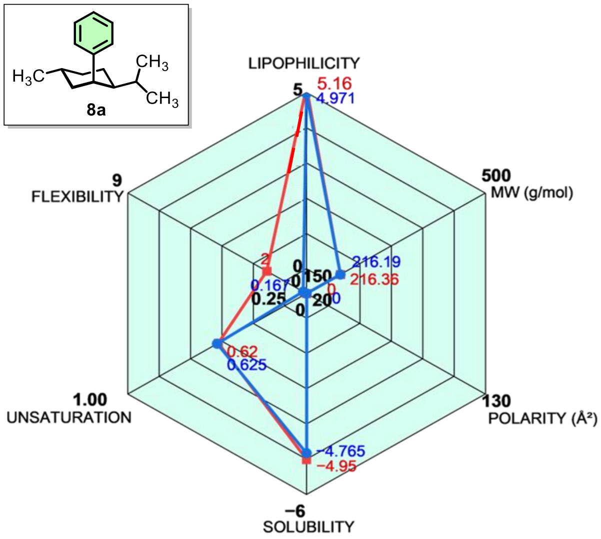


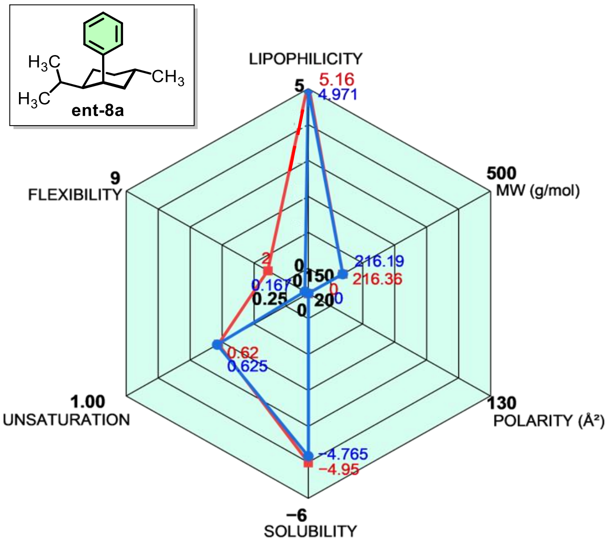


| Compound | 8a | ent-8a |
|---|---|---|
| BBB permeability | No/Maybe | No/Maybe |
| Skin permeability (cm.s$^{-1}$) | -3.54/-2.165 | -3.54/-2.165 |
| Intestinal absorption | Yes/Yes/Yes | Yes/Yes/Yes |
| P-gp substrate | No/No | No/No |
| Lipinski's rule of five | Yes/Yes | Yes/Yes |

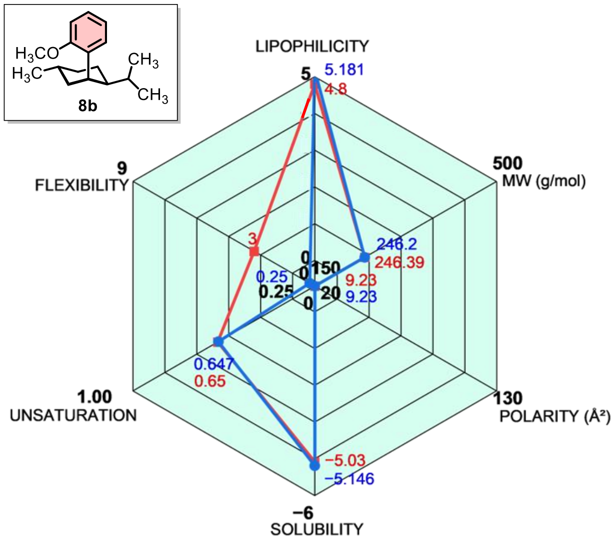


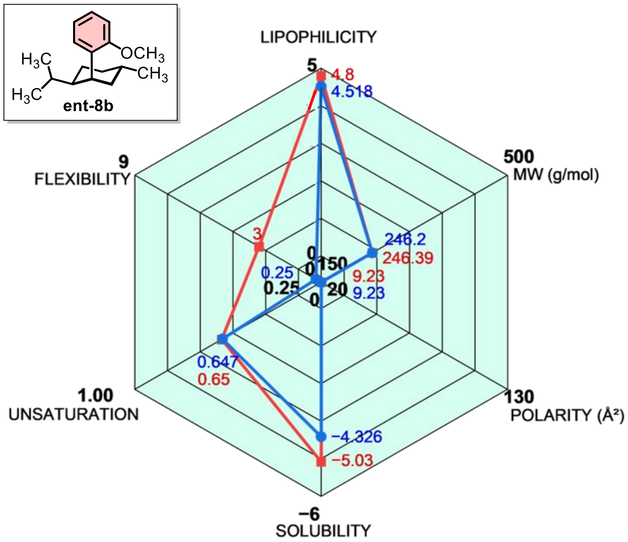


| Compound | 8b | ent-8b |
|---|---|---|
| BBB permeability | Yes/No | Yes/No |
| Skin permeability (cm.s$^{-1}$) | -3.73/-2.069 | -3.73/-2.069 |
| Intestinal absorption | Yes/Yes/Yes | Yes/Yes/Yes |
| P-gp substrate | No/No | No/No |
| Lipinski's rule of five | Yes/Yes | Yes/Yes |

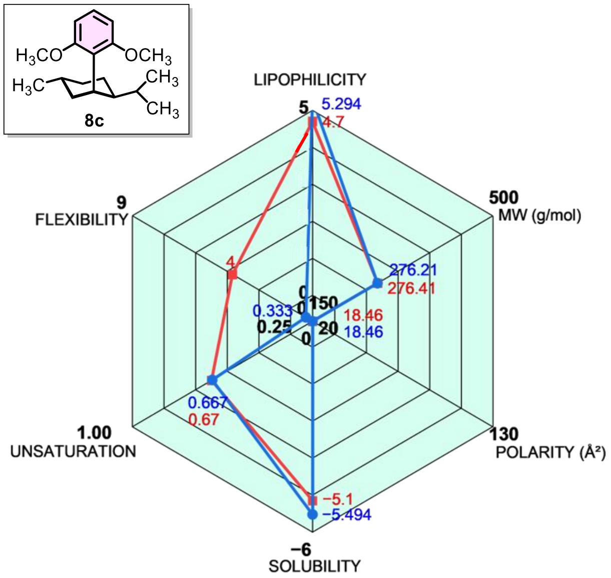


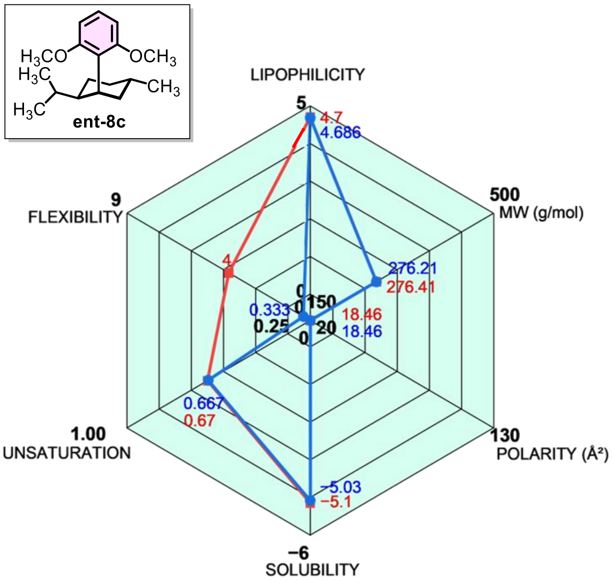


| Compound | 8c | ent-8c |
|---|---|---|
| BBB permeability | Yes/Yes | Yes/Yes |
| Skin permeability (cm.s$^{-1}$) | -3.94/-2.182 | -3.94/-2.182 |
| Intestinal absorption | Yes/Yes/Yes | Yes/Yes/Yes |
| P-gp substrate | No/No | No/No |
| Lipinski's rule of five | Yes/Yes | Yes/Yes |

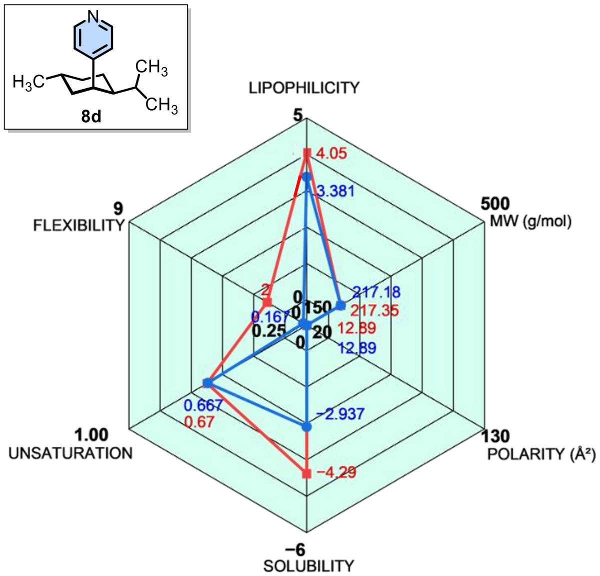


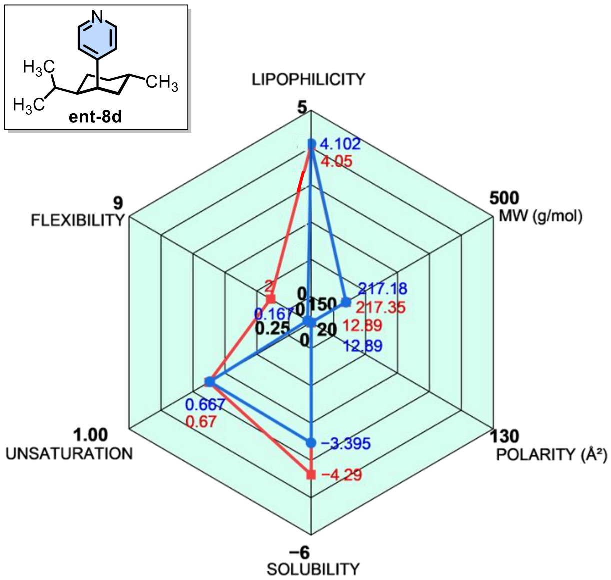


| Compound | 8d | ent-8d |
|---|---|---|
| BBB permeability | Yes/Yes | Yes/Yes |
| Skin permeability (cm.s$^{-1}$) | -4.3/-1.489 | -4.3/-1.489 |
| Intestinal absorption | Yes/Yes/Yes | Yes/Yes/Yes |
| P-gp substrate | No/No | No/No |
| Lipinski's rule of five | Yes/Yes | Yes/Yes |

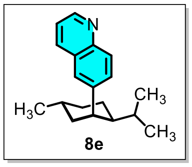


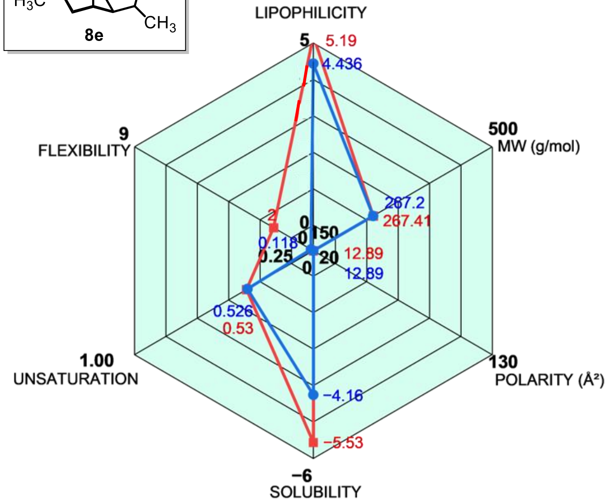


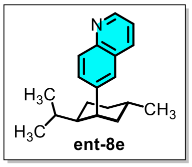


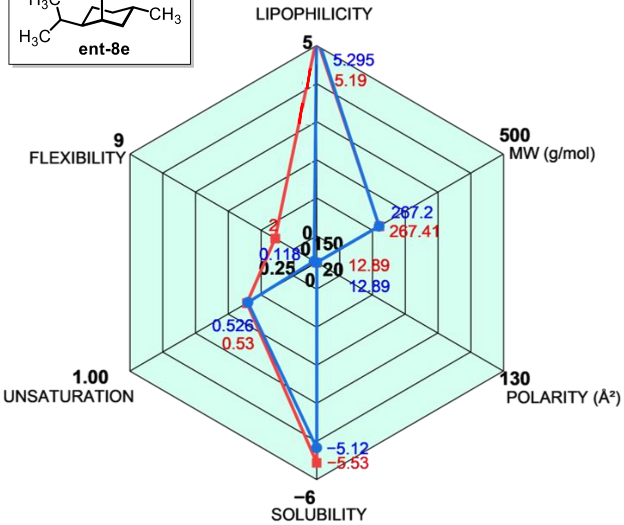


| Compound | 8e | ent-8e |
|---|---|---|
| BBB permeability | Yes/Yes | Yes/Yes |
| Skin permeability (cm.s$^{-1}$) | -3.66/-2.237 | -3.66/-2.237 |
| Intestinal absorption | Yes/Yes/Yes | Yes/Yes/Yes |
| P-gp substrate | No/No | No/No |
| Lipinski's rule of five | Yes/Yes | Yes/Yes |

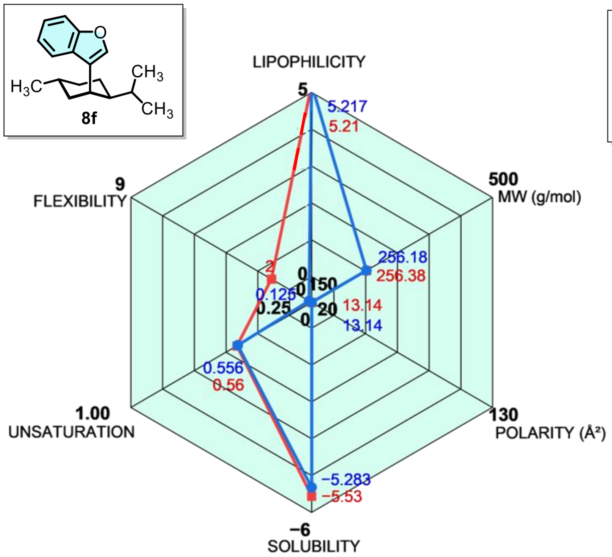


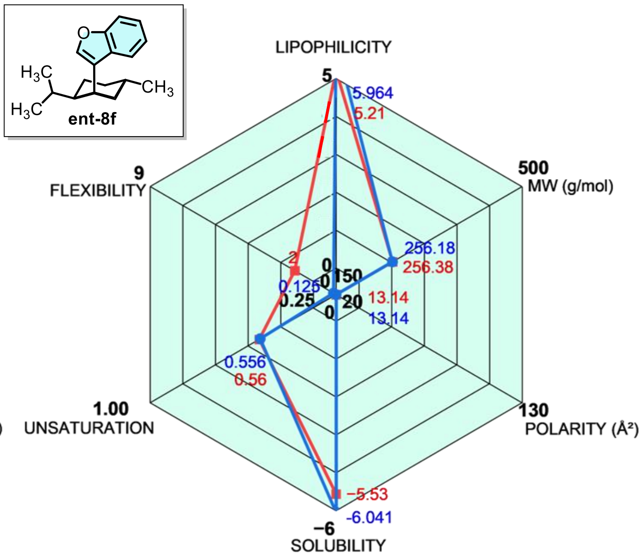


| Compound | 8f | ent-8f |
|---|---|---|
| BBB permeability | No/Yes | No/Yes |
| Skin permeability (cm.s$^{-1}$) | -3.49/-2.173 | -3.49/-2.173 |
| Intestinal absorption | Yes/Yes/Yes | Yes/Yes/Yes |
| P-gp substrate | No/No | No/No |
| Lipinski's rule of five | Yes/Yes | Yes/Yes |

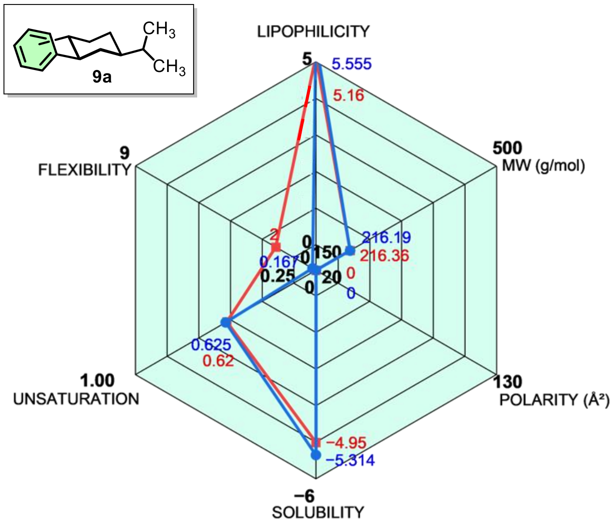


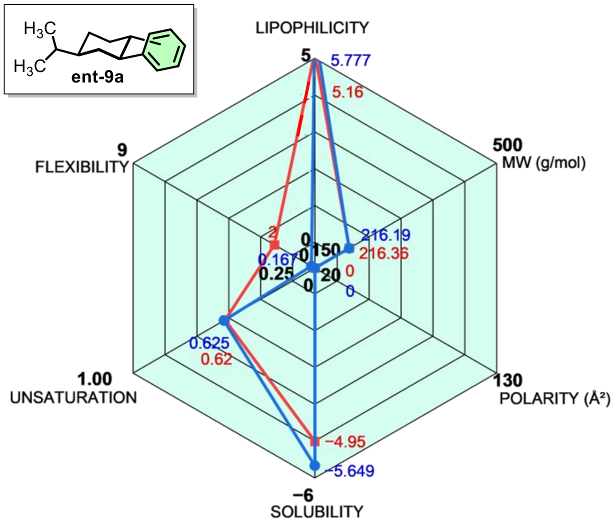


| Compound | 9a | ent-9a |
|---|---|---|
| BBB permeability | No/Maybe | No/Maybe |
| Skin permeability (cm.s$^{-1}$) | -3.54/-1.975 | -3.54/-1.975 |
| Intestinal absorption | Yes/Yes/Yes | Yes/Yes/Yes |
| P-gp substrate | No/No | No/No |
| Lipinski's rule of five | Yes/Yes | Yes/Yes |

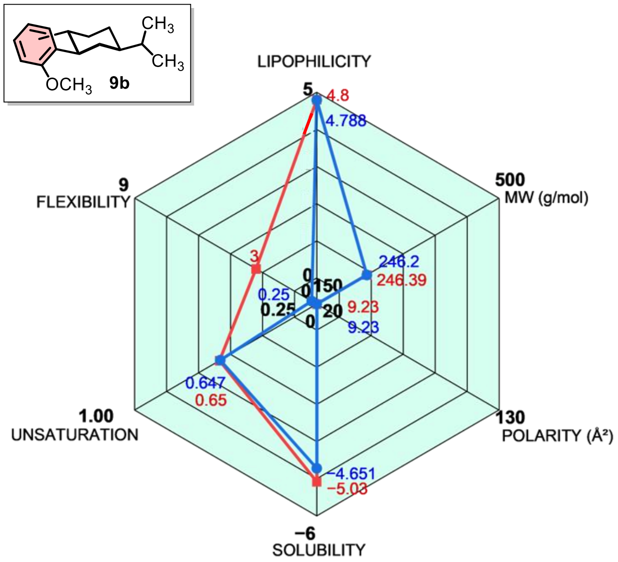


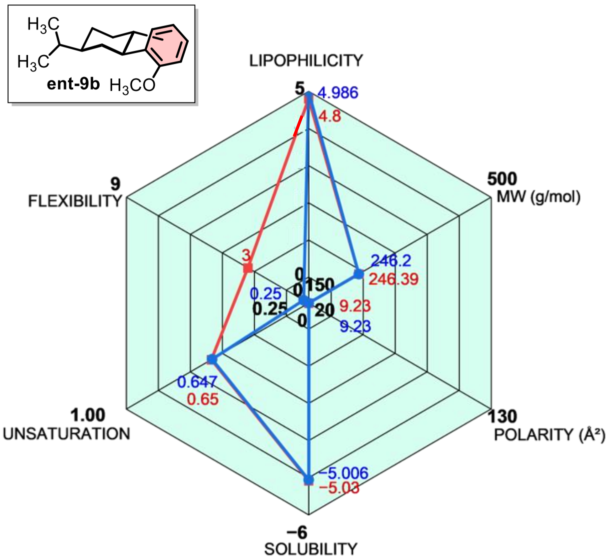


| Compound | 9b | ent-9b |
|---|---|---|
| BBB permeability | Yes/Yes | Yes/Yes |
| Skin permeability (cm.s$^{-1}$) | -3.73/-2.061 | -3.73/-2.061 |
| Intestinal absorption | Yes/Yes/Yes | Yes/Yes/Yes |
| P-gp substrate | No/No | No/No |
| Lipinski's rule of five | Yes/Yes | Yes/Yes |

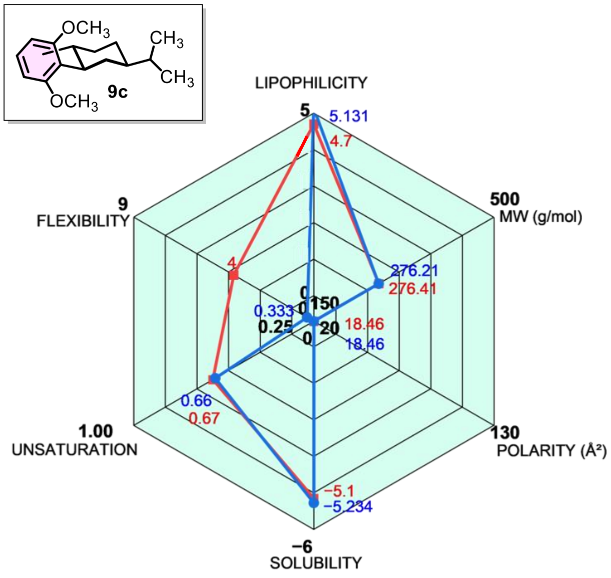


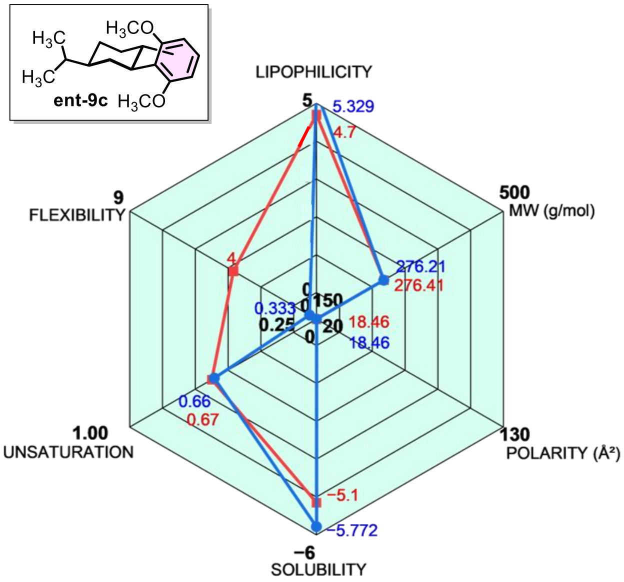


| Compound | 9c | ent-9c |
|---|---|---|
| BBB permeability | Yes/Yes | Yes/Yes |
| Skin permeability (cm.s$^{-1}$) | -3.94/-2.201 | -3.94/-2.201 |
| Intestinal absorption | Yes/Yes/Yes | Yes/Yes/Yes |
| P-gp substrate | No/No | No/No |
| Lipinski's rule of five | Yes/Yes | Yes/Yes |

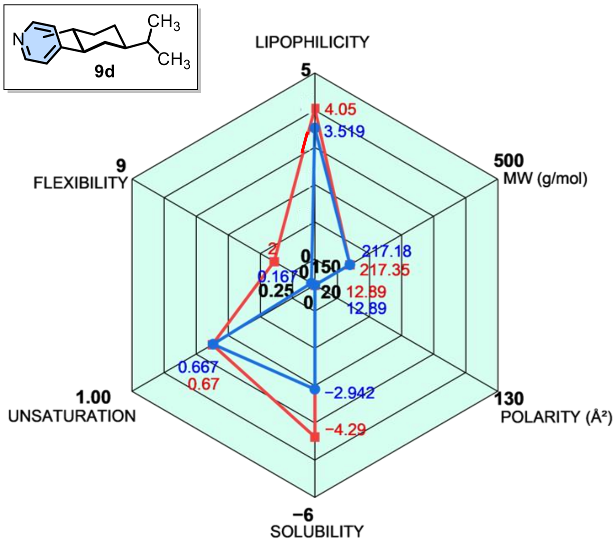


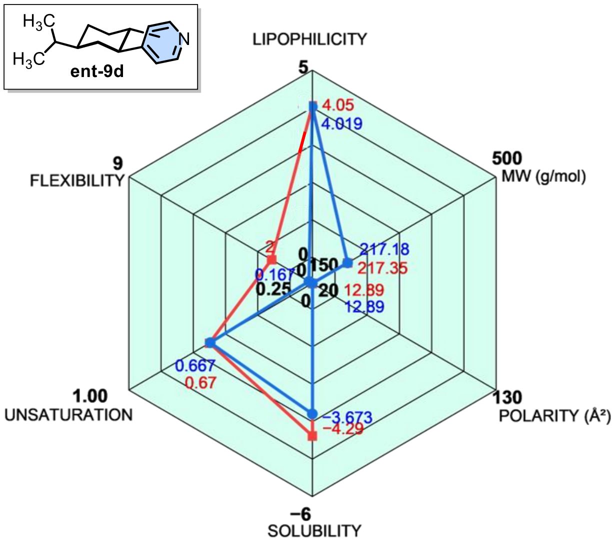


| Compound | 9d | ent-9d |
|---|---|---|
| BBB permeability | Yes/Yes | Yes/Yes |
| Skin permeability (cm.s$^{-1}$) | -4.3/-1.477 | -4.3/-1.477 |
| Intestinal absorption | Yes/Yes/Yes | Yes/Yes/Yes |
| P-gp substrate | No/No | No/No |
| Lipinski's rule of five | Yes/Yes | Yes/Yes |

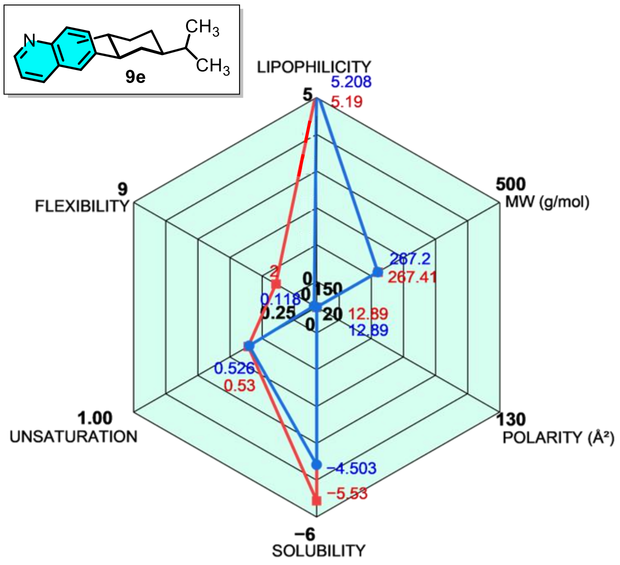


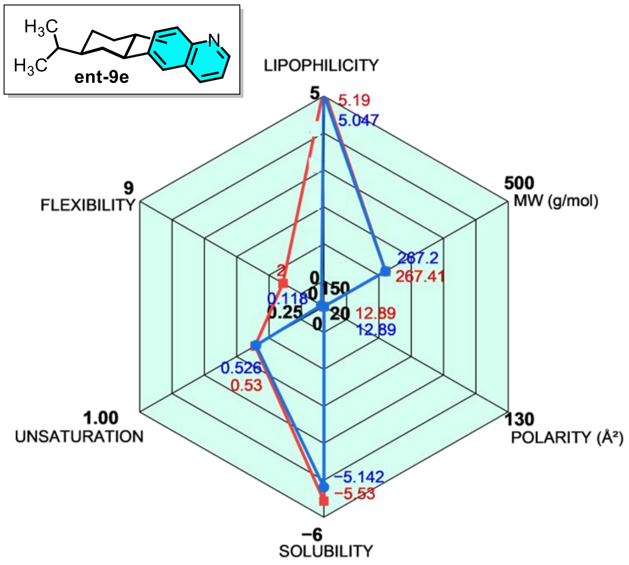


| Compound | 9e | ent-9e |
|---|---|---|
| BBB permeability | Yes/Yes | Yes/Yes |
| Skin permeability (cm.s$^{-1}$) | -3.66/-2.128 | -3.66/-2.128 |
| Intestinal absorption | Yes/Yes/Yes | Yes/Yes/Yes |
| P-gp substrate | No/No | No/No |
| Lipinski's rule of five | Yes/Yes | Yes/Yes |

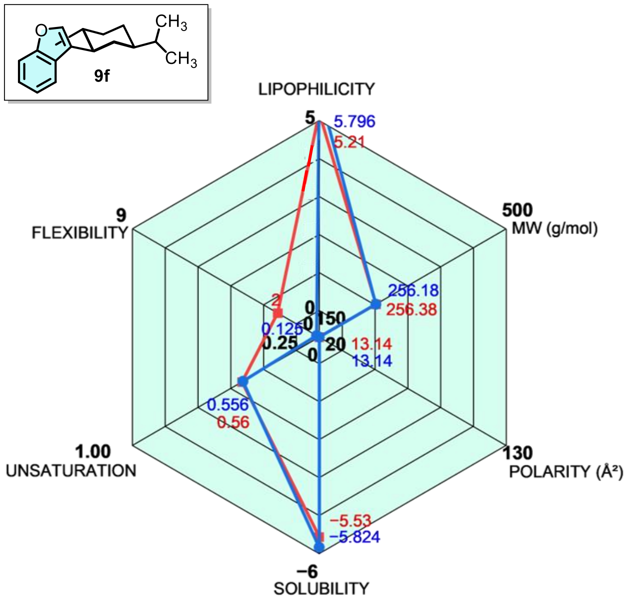


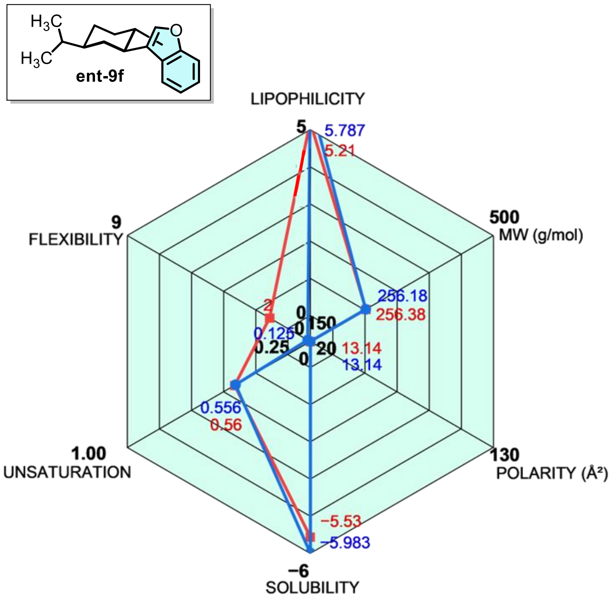


| Compound | 9f | ent-9f |
|---|---|---|
| BBB permeability | No/Yes | No/Yes |
| Skin permeability (cm.s$^{-1}$) | -3.49/-2.151 | -3.49/-2.151 |
| Intestinal absorption | Yes/Yes/Yes | Yes/Yes/Yes |
| P-gp substrate | No/No | No/No |
| Lipinski's rule of five | Yes/Yes | Yes/Yes |

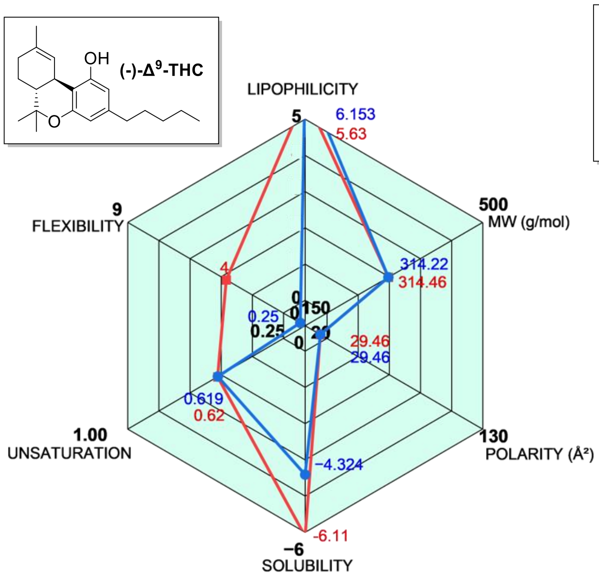


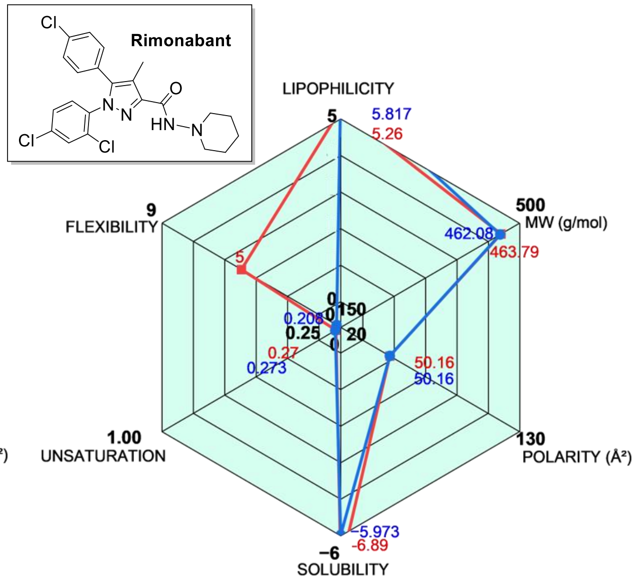


| Compound | (-)-Δ$^{9}$-THC | Rimonabant |
|---|---|---|
| BBB permeability | Yes/No | Yes/No |
| Skin permeability (cm.s$^{-1}$) | -3.27/-2.696 | -4.52/-2.671 |
| Intestinal absorption | Yes/Yes/Yes | Yes/Yes/Yes |
| P-gp substrate | No/No | No/Yes |
| Lipinski's rule of five | Yes/Yes | Yes/Yes |

## VI. Molecular Docking of Ketone Precursors and Synthesized Compounds Against Cannabinoid Receptors CB1 and CB2: Optimal Complex Poses and Intermolecular Interactions

• *Ketone Precursors with CB1 Receptor*

<table>
<tr><th>(-)-1</th><th>(+)-1</th></tr>
<tr><td>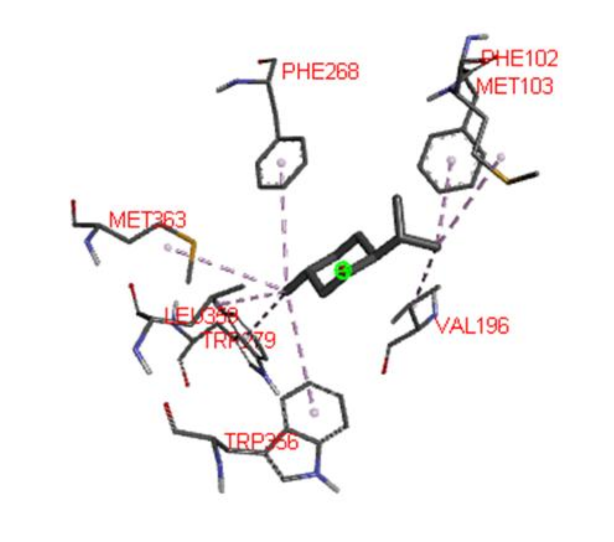
</td><td>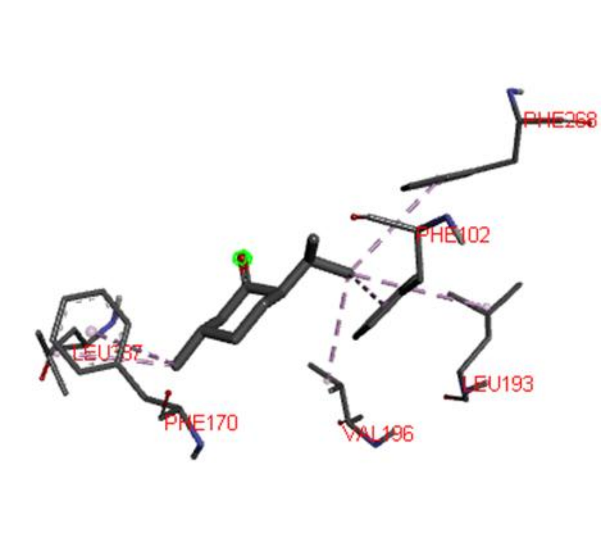
</td></tr>
<tr><td>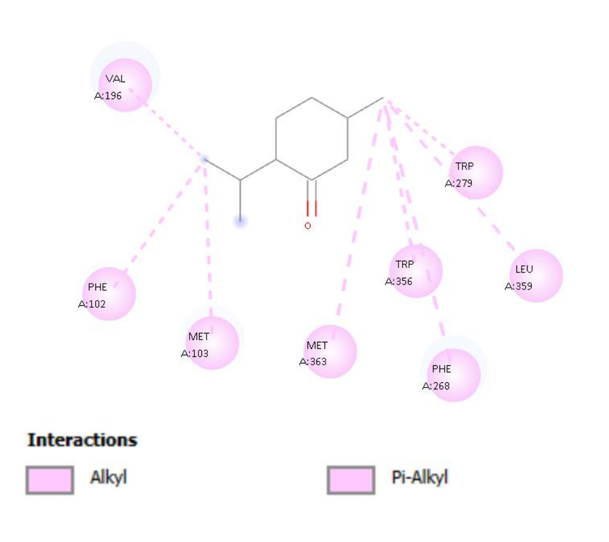
</td><td>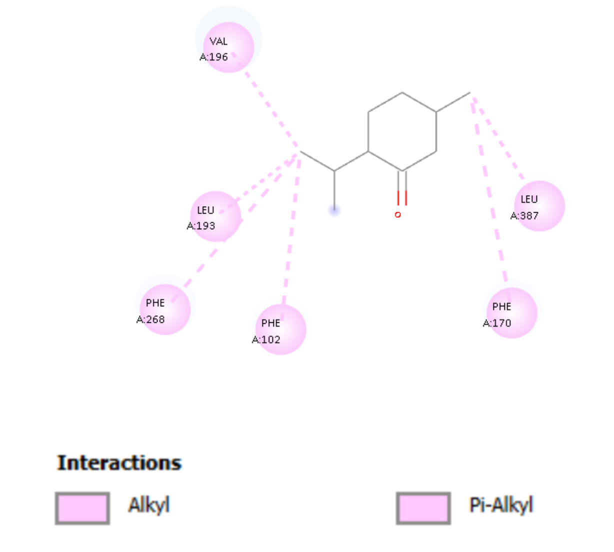
</td></tr>
<tr><td>Binding energy: -6.3</td><td>Binding energy: -6.2</td></tr>
</table>

| (+)-2 | (-)-2 |
|---|---|
| 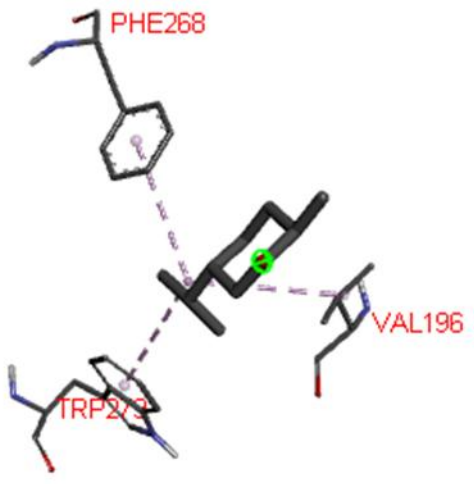 | 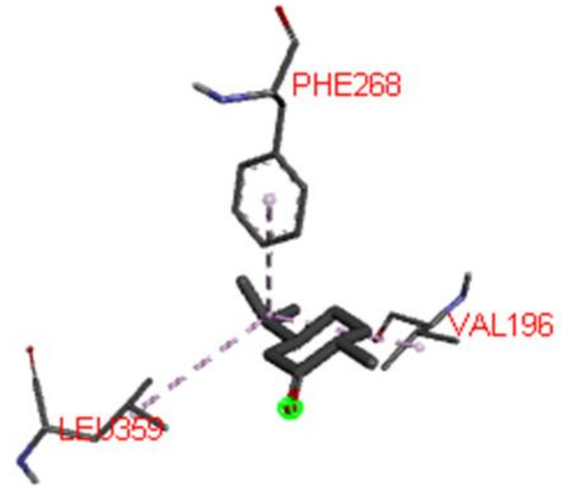 |
| 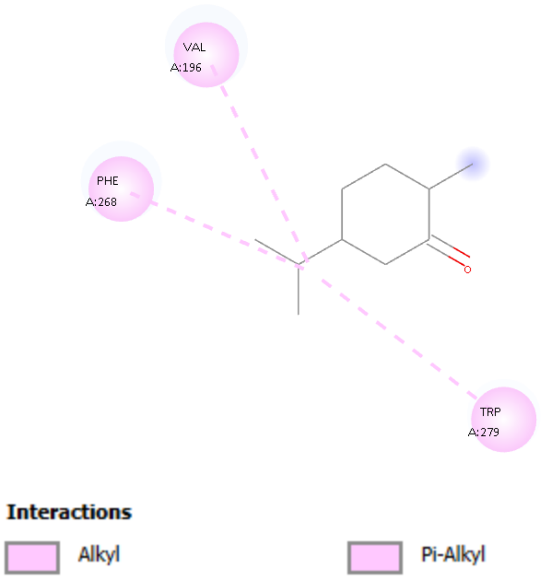 | 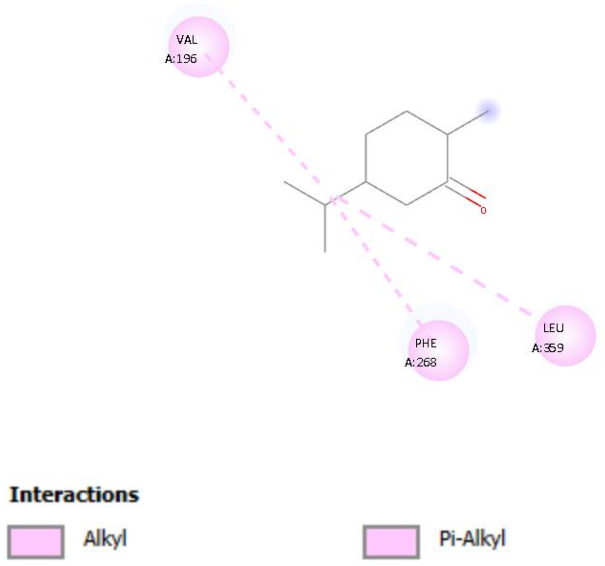 |
| **Binding energy: -6.3** | **Binding energy: -6.2** |

• *Synthesized Ligands of Series **6**/ent-**6** with CB1 Receptor*

<table>
<tr><th>6a (II)</th><th>ent-6a (II)</th></tr>
<tr><td>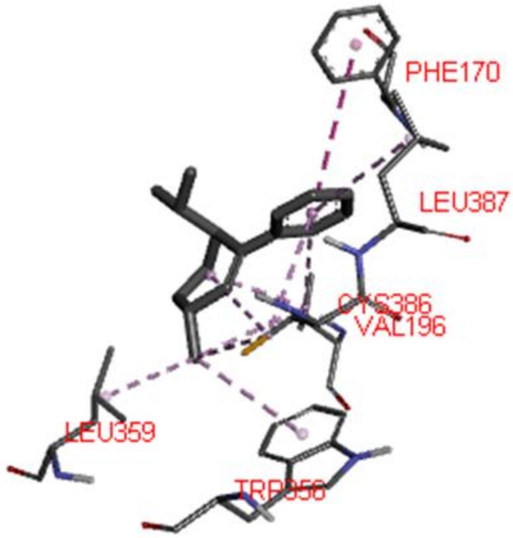
</td><td>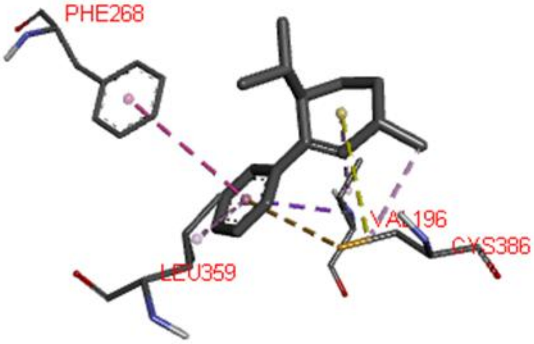
</td></tr>
<tr><td>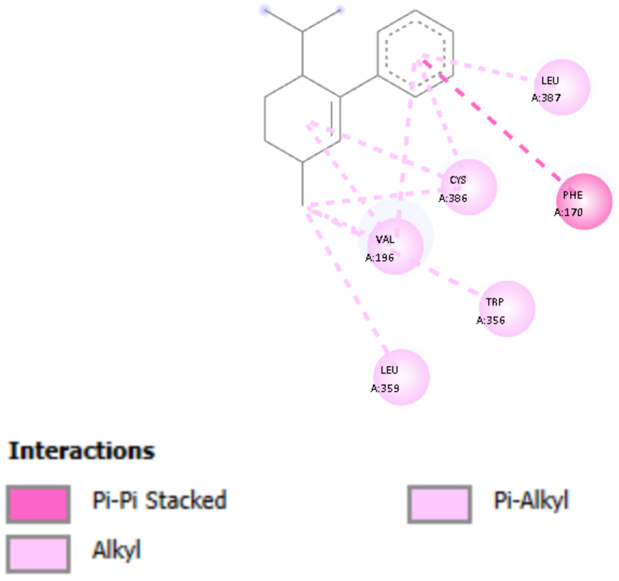
</td><td>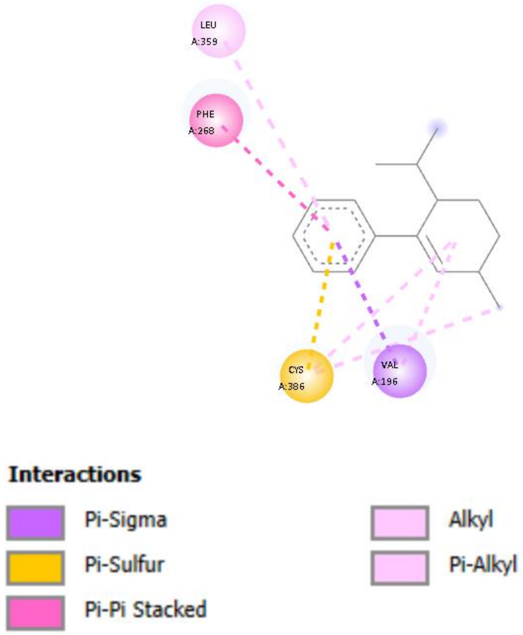
</td></tr>
<tr><td>Binding energy: -8.0</td><td>Binding energy: -7.7</td></tr>
</table>

<table>
<tr><th>6b (I)</th><th>ent-6b (I)</th></tr>
<tr><td>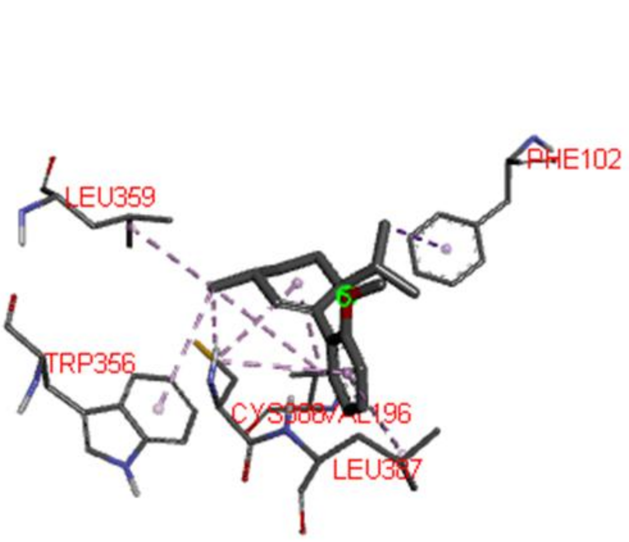
</td><td>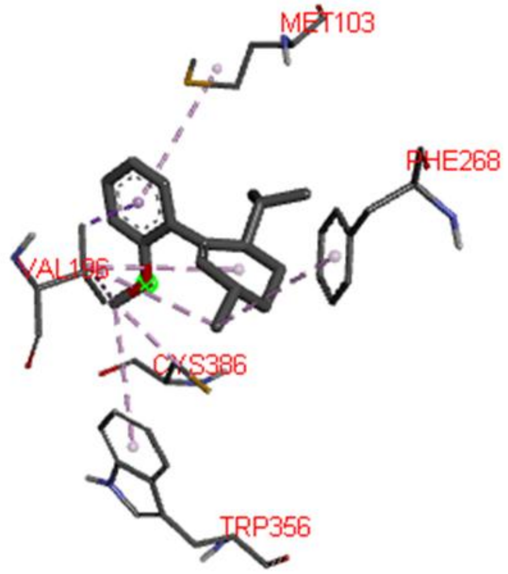
</td></tr>
<tr><td>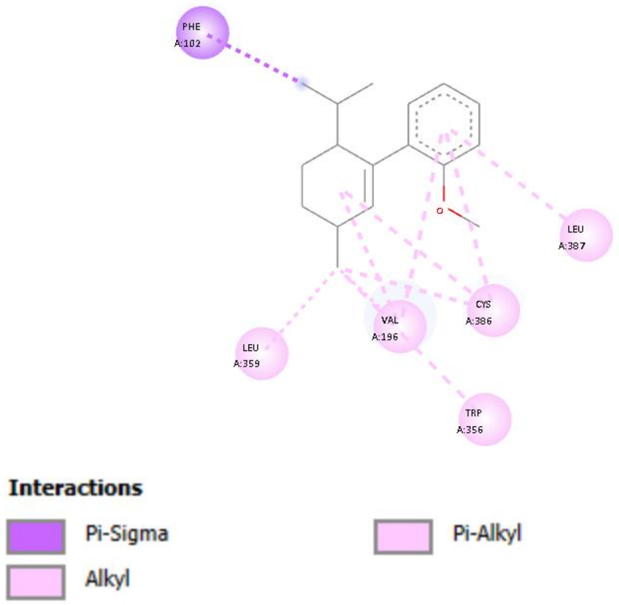
</td><td>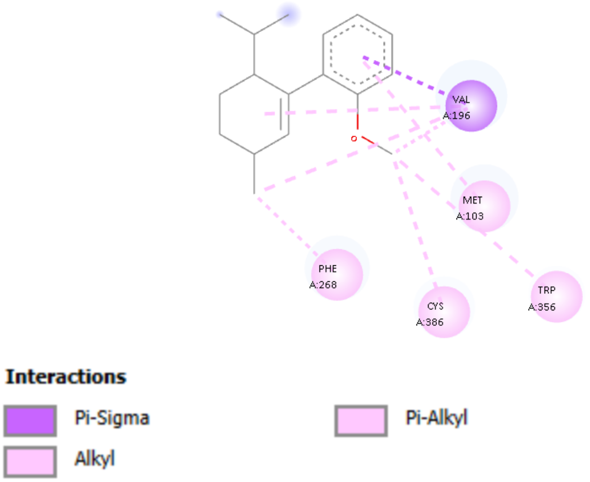
</td></tr>
<tr><td>Binding energy: -7.1</td><td>Binding energy: -7.1</td></tr>
</table>

<table>
<tr><th>6b (II)</th><th>ent-6b (II)</th></tr>
<tr><td>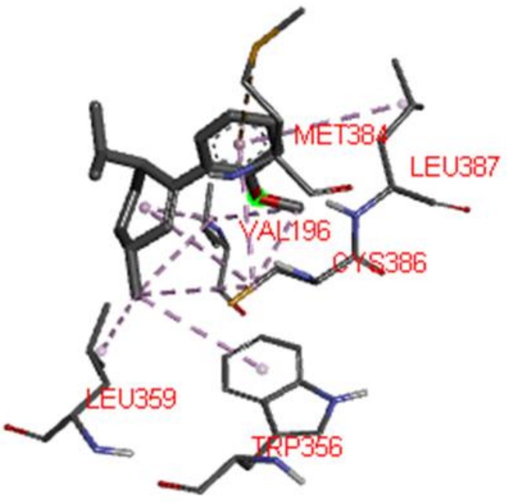
</td><td>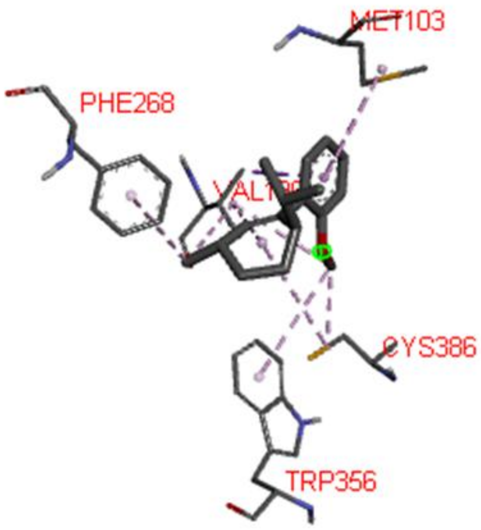
</td></tr>
<tr><td>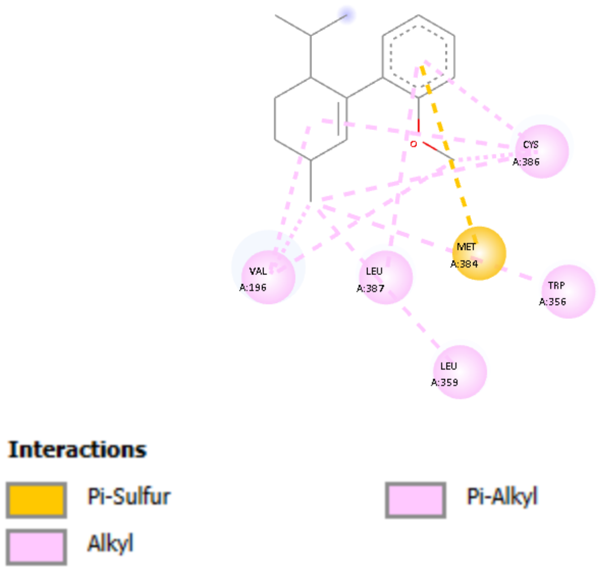
</td><td>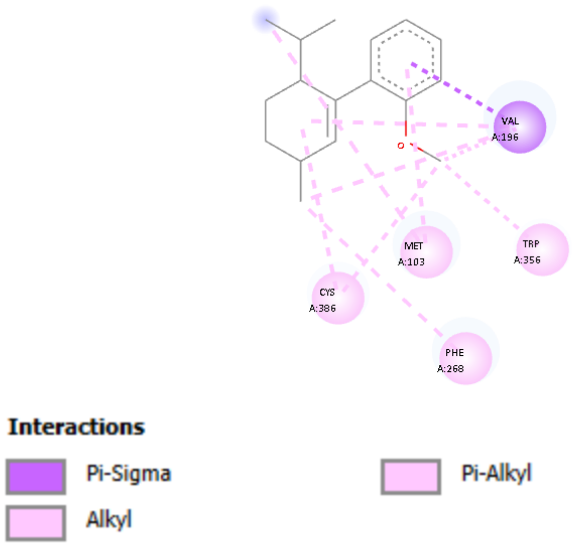
</td></tr>
<tr><td>Binding energy: -8.0</td><td>Binding energy: -7.1</td></tr>
</table>

<table>
<tr><th>6c (I)</th><th>ent-6c (I)</th></tr>
<tr><td>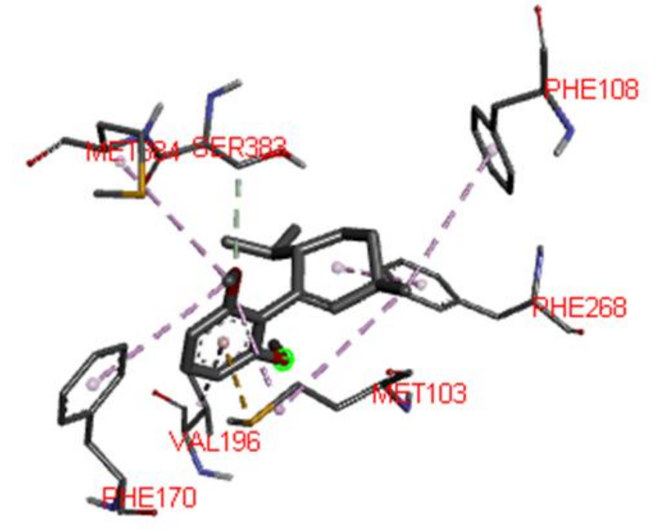
</td><td>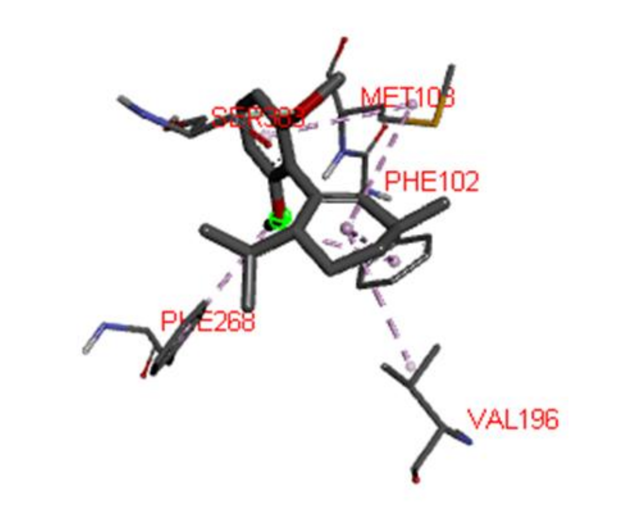
</td></tr>
<tr><td>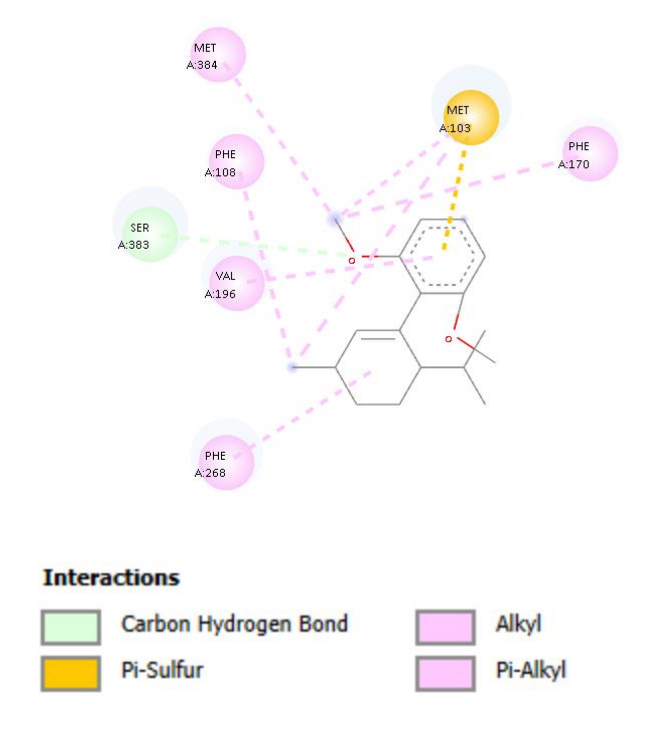
</td><td>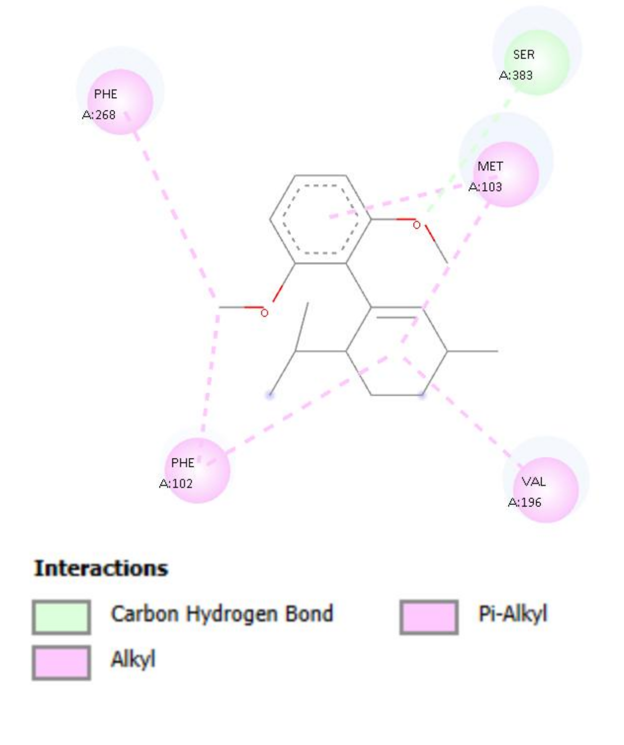
</td></tr>
<tr><td>Binding energy: -6.1</td><td>Binding energy: -5.8</td></tr>
</table>

| 6d (I) | ent-6d (I) |
|---|---|
| 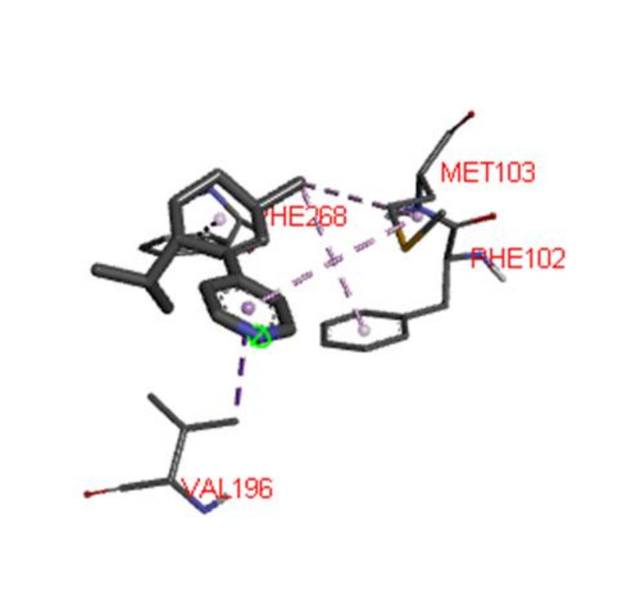 | 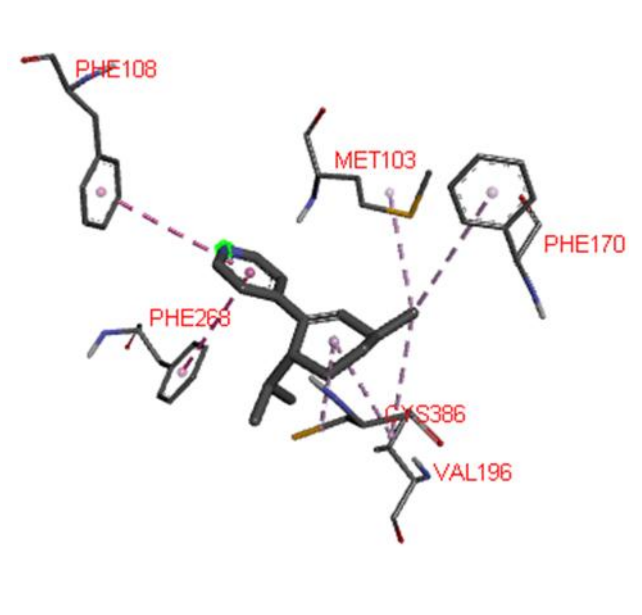 |
| 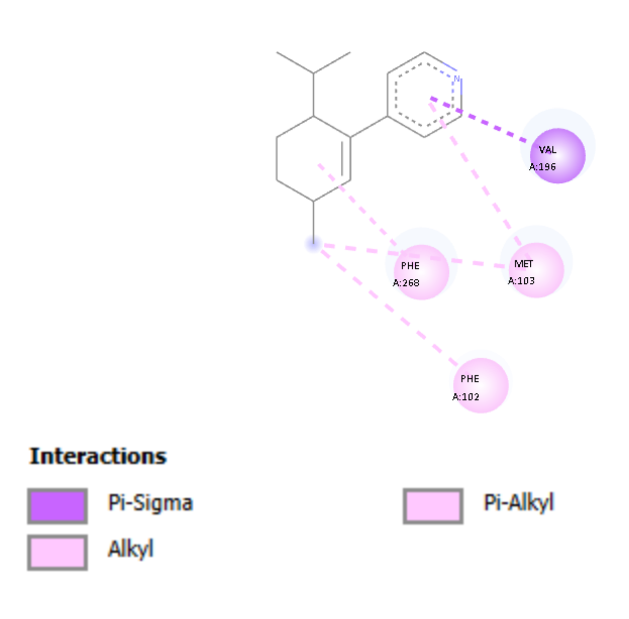 | 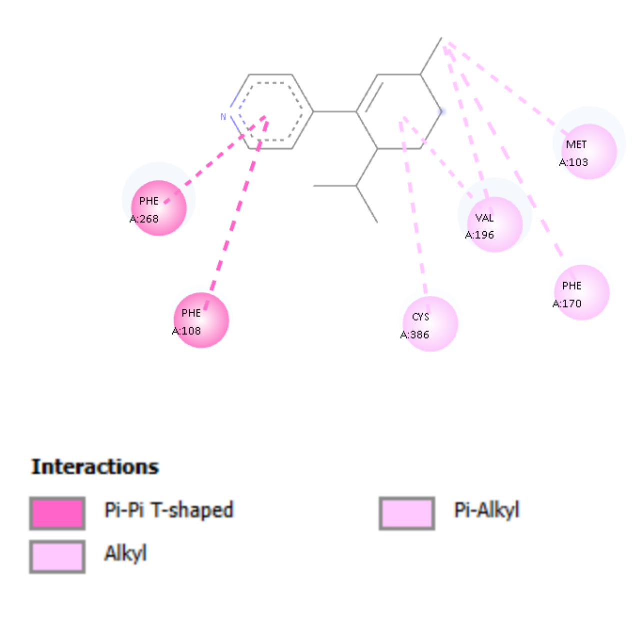 |
| **Binding energy: -8.0** | **Binding energy: -7.8** |

| 6d (II) | ent-6d (II) |
|---|---|
| 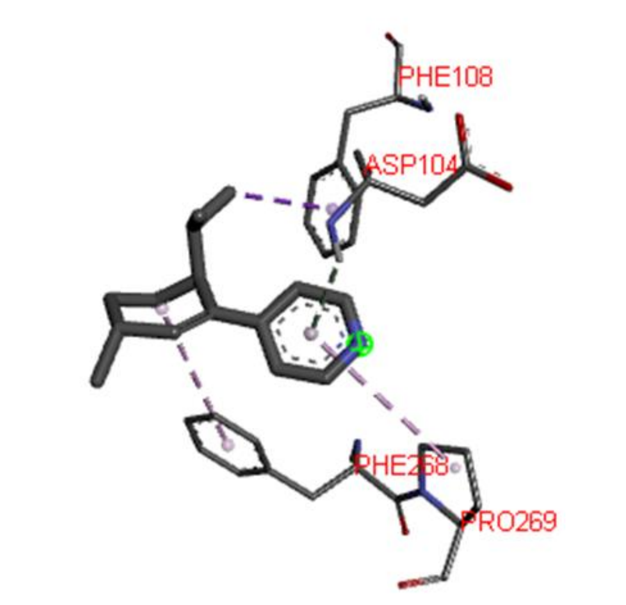 | 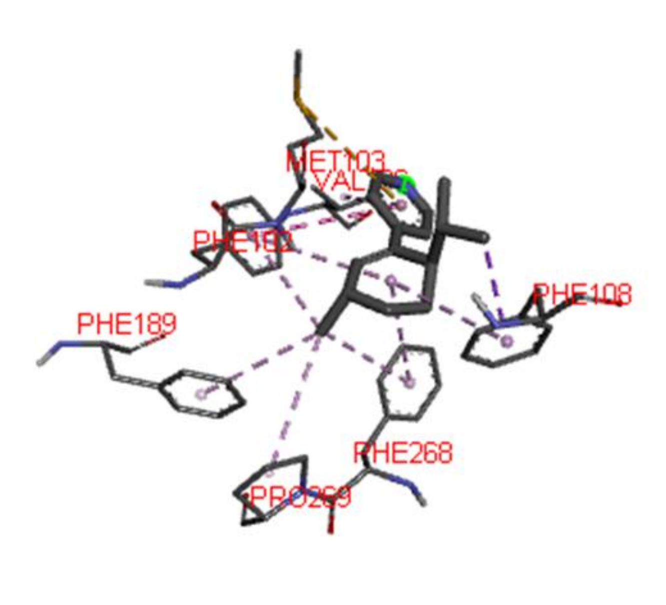 |
| 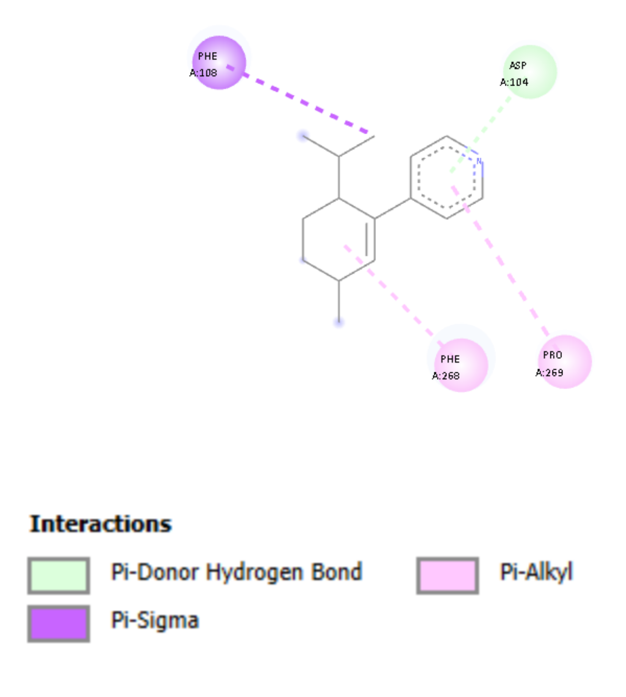 | 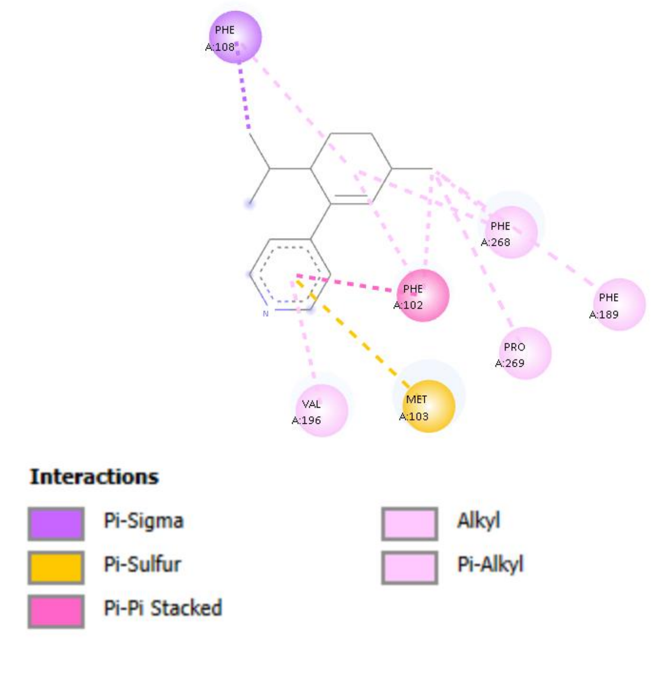 |
| **Binding energy: -8.4** | **Binding energy: -7.7** |

| 6e (II) | ent-6e (II) |
|---|---|
| 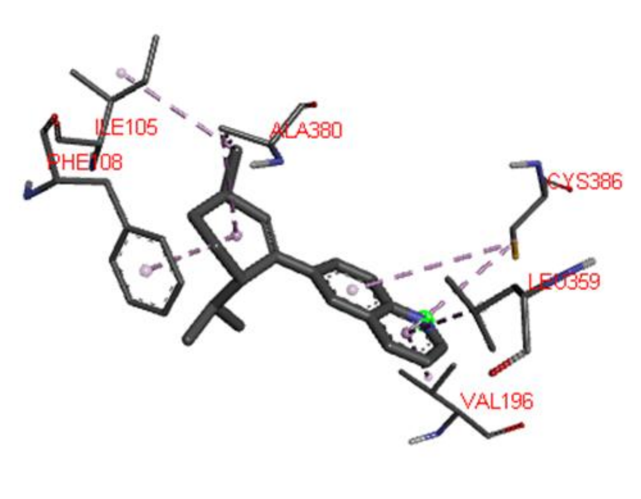 | 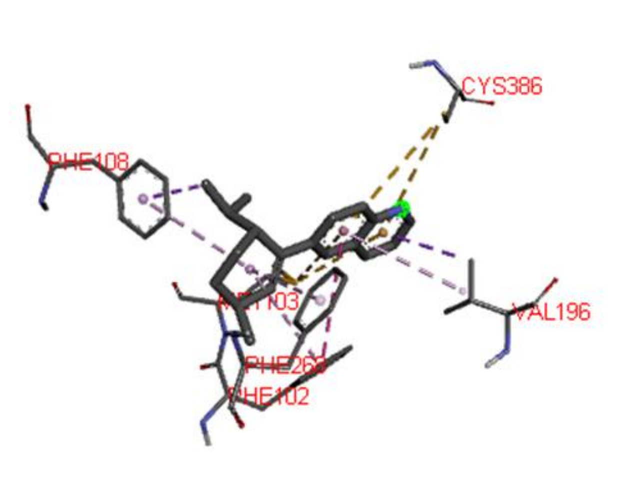 |
| 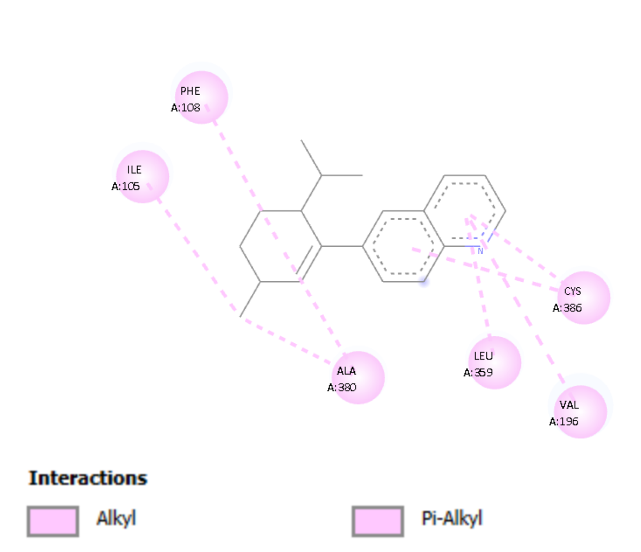 | 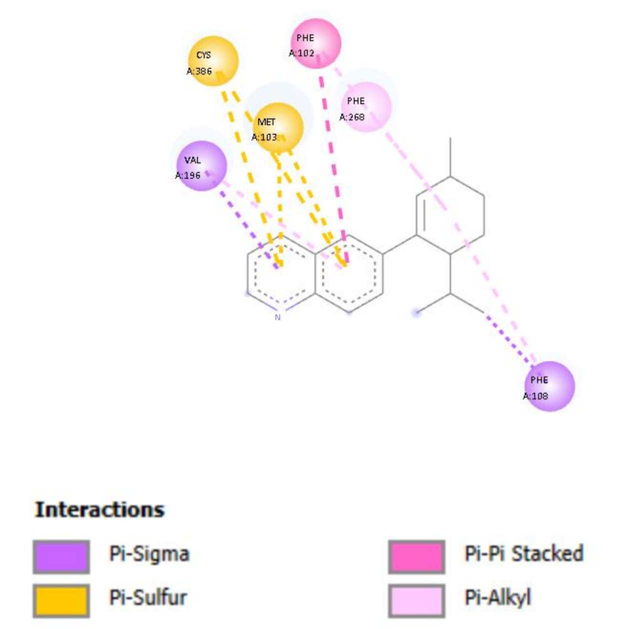 |
| **Binding energy: -9.6** | **Binding energy: -9.6** |

<table>
<tr><th>6f (II)</th><th>ent-6f (II)</th></tr>
<tr><td>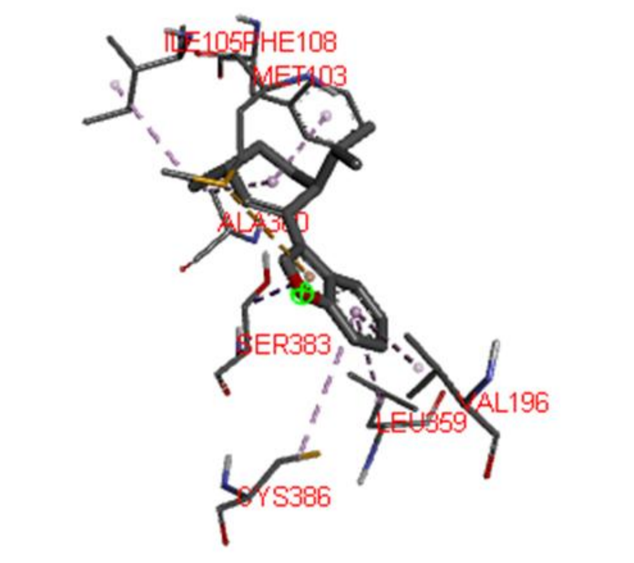
</td><td>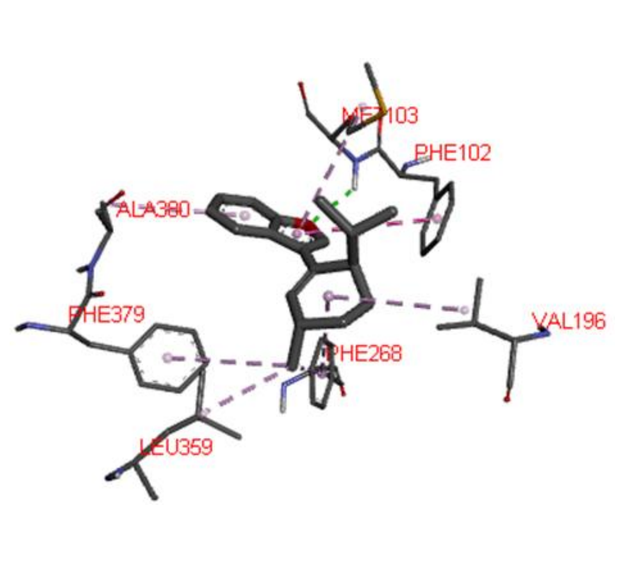
</td></tr>
<tr><td>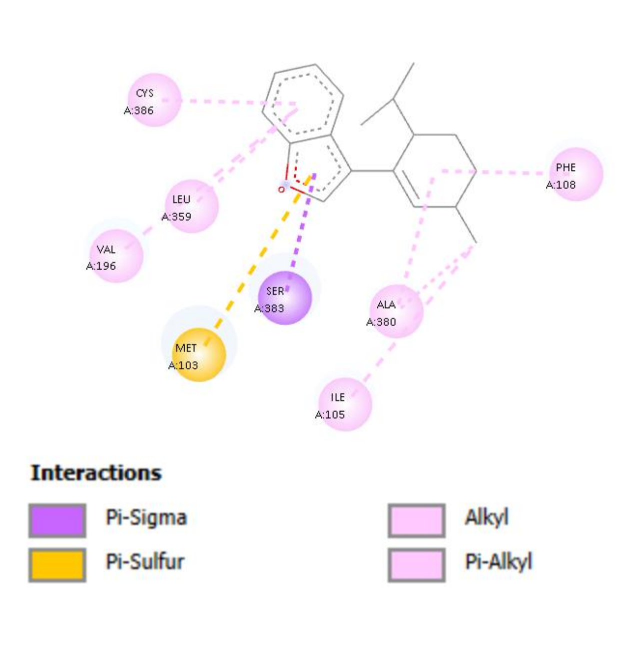
</td><td>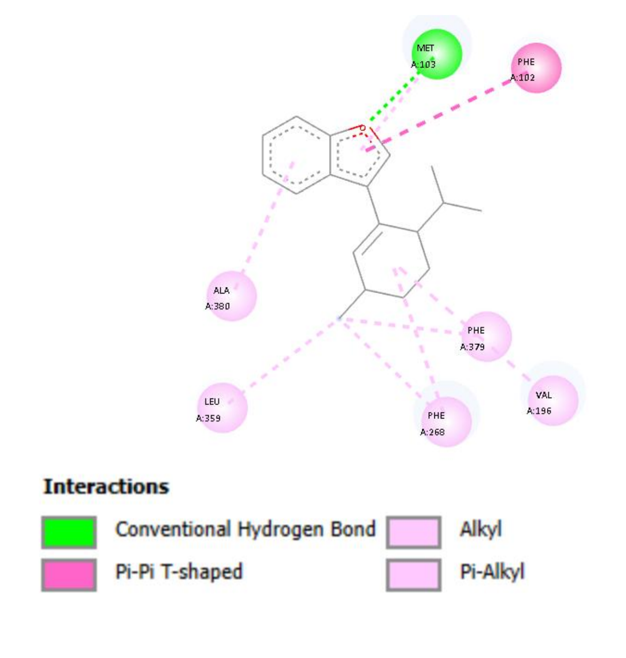
</td></tr>
<tr><td>Binding energy: -9.5</td><td>Binding energy: -9.2</td></tr>
</table>

• *Synthesized Ligands of Series **7**/ent-**7** with CB1 Receptor*

| **7a (I)** | **ent-7a (I)** |
| --- | --- |
| 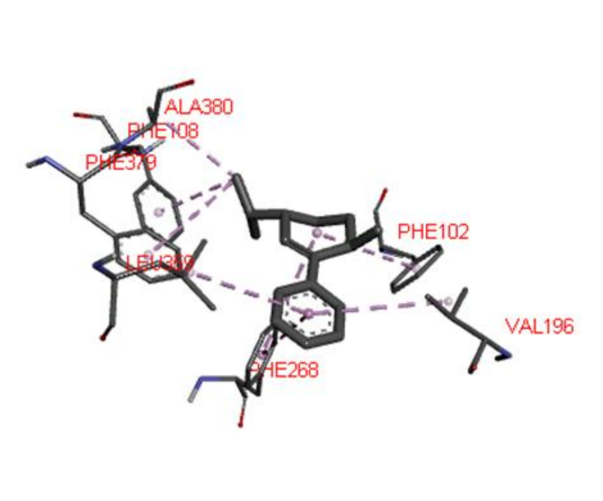 | 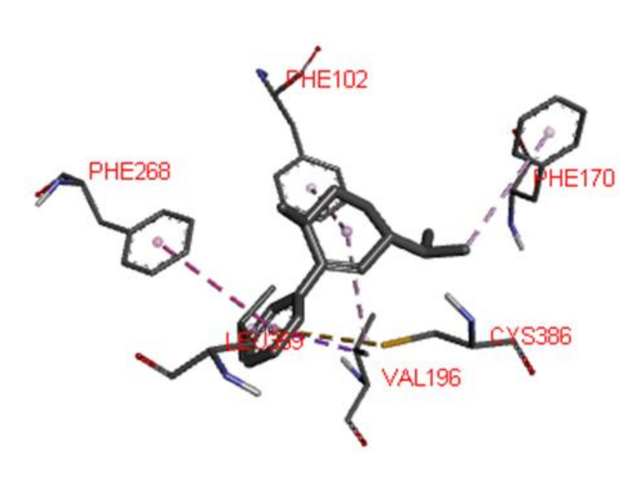 |
| 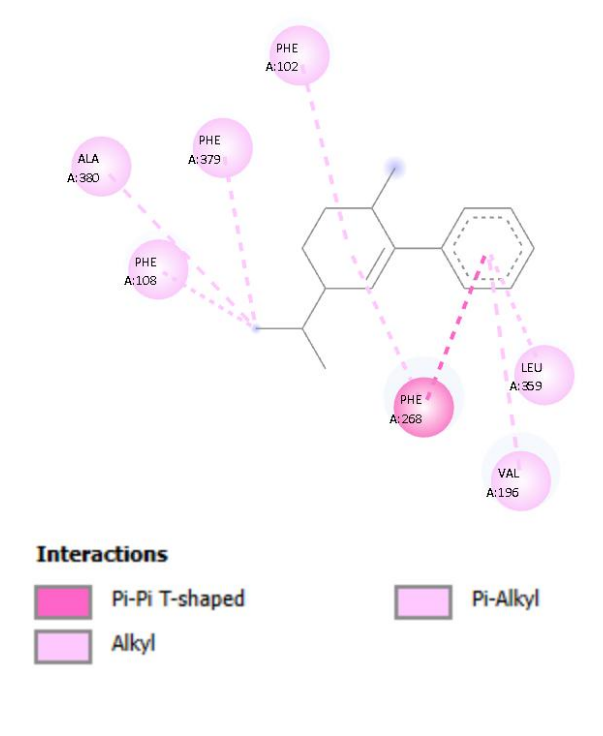 | 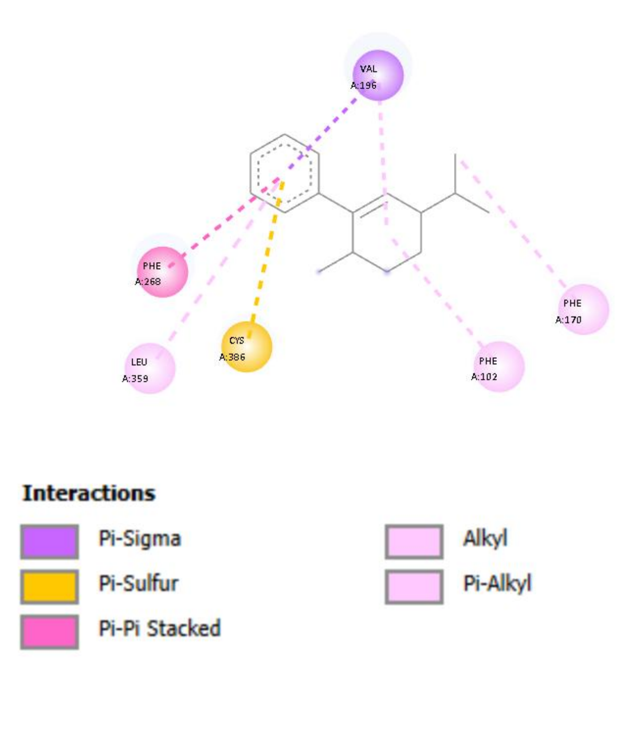 |
| **Binding energy: -8.8** | **Binding energy: -8.4** |

<table>
<tr><th>7a (II)</th><th>ent-7a (II)</th></tr>
<tr><td>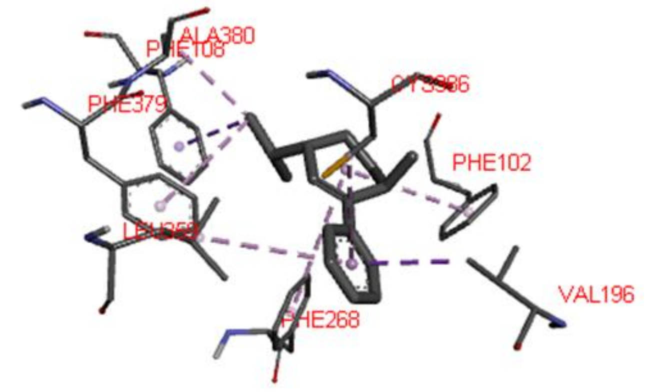
</td><td>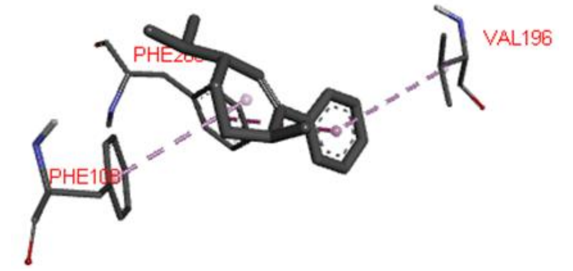
</td></tr>
<tr><td>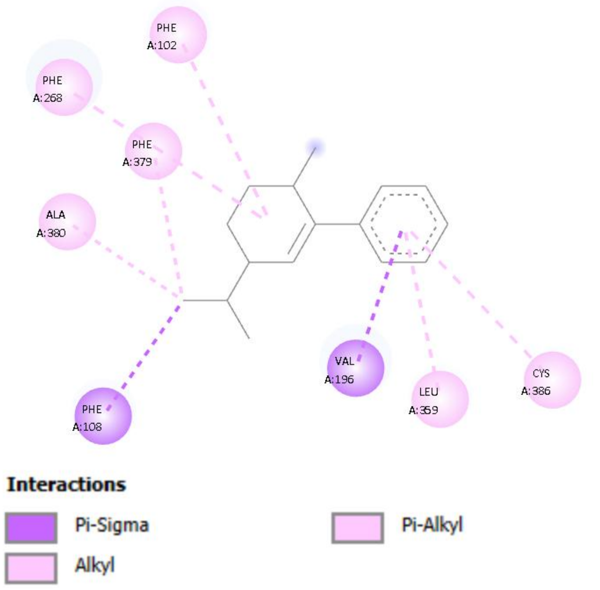
</td><td>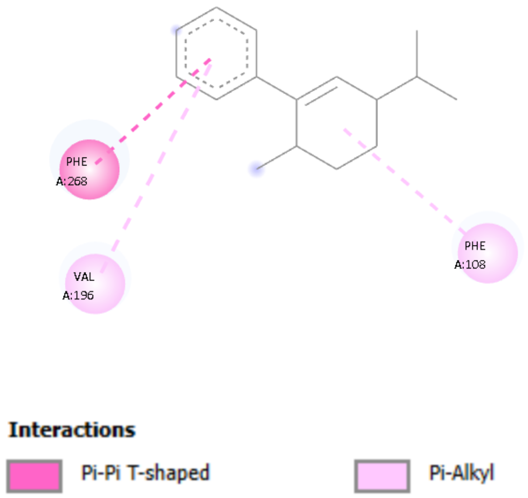
</td></tr>
<tr><td>Binding energy: -8.8</td><td>Binding energy: -8.7</td></tr>
</table>

<table>
<tr><th>7b (I)</th><th>ent-7b (I)</th></tr>
<tr><td>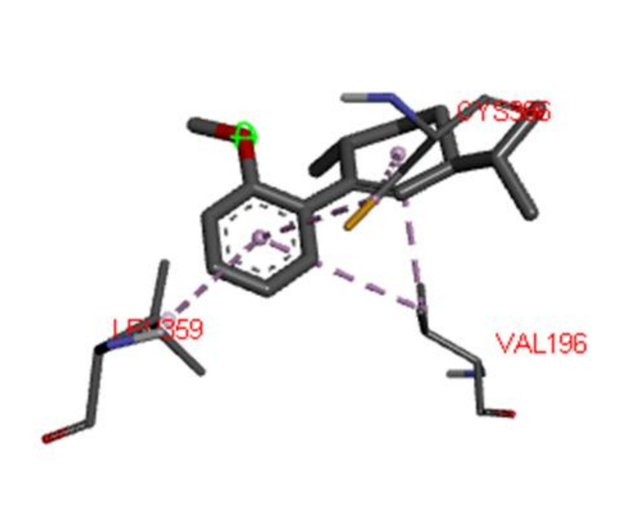
</td><td>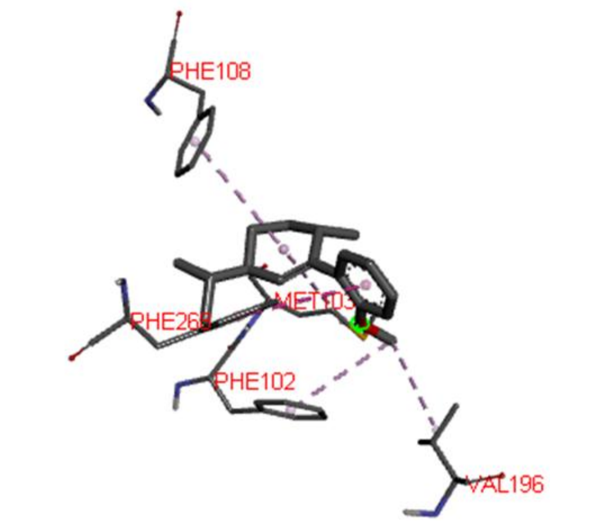
</td></tr>
<tr><td>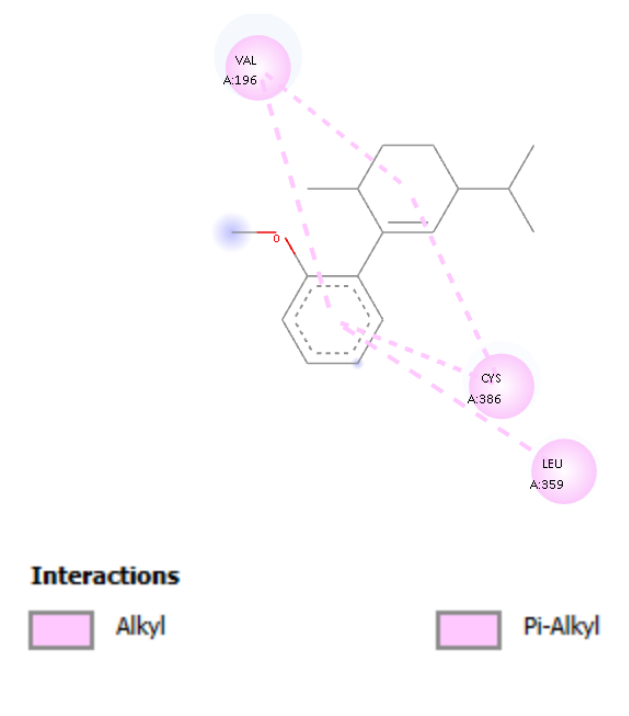
</td><td>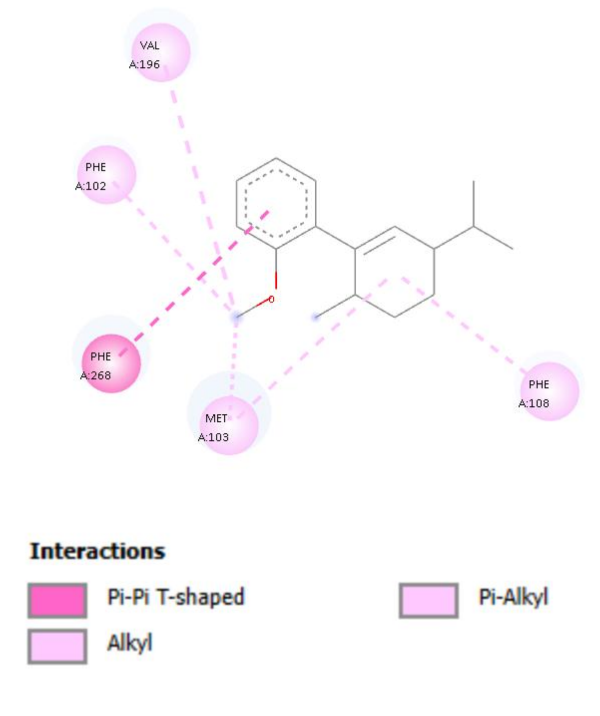
</td></tr>
<tr><td>Binding energy: -8.4</td><td>Binding energy: -8.1</td></tr>
</table>

<table>
<tr><th>7b (II)</th><th>ent-7b (II)</th></tr>
<tr><td>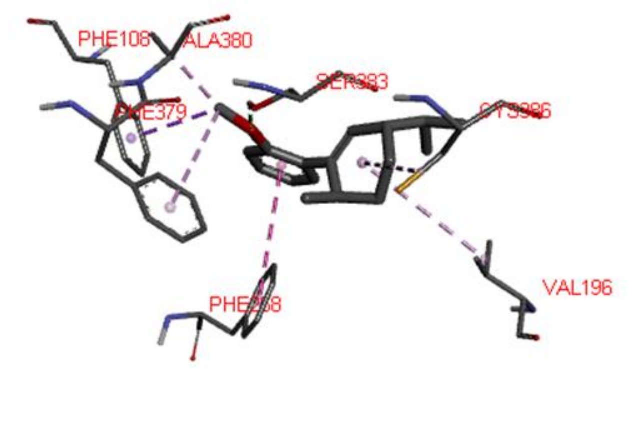
</td><td>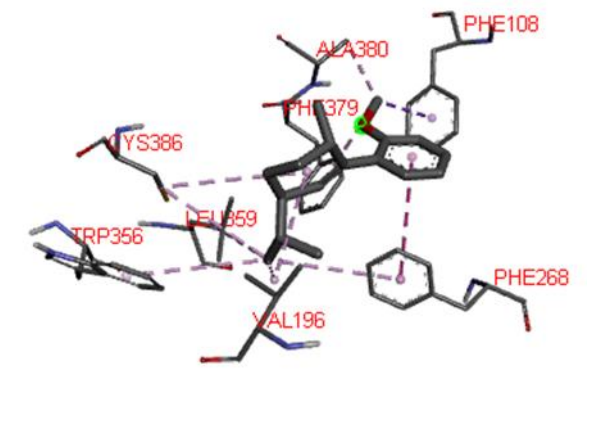
</td></tr>
<tr><td>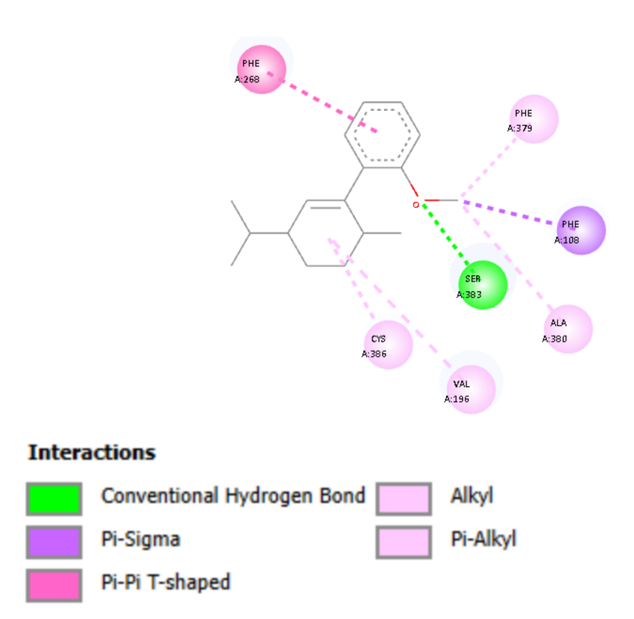
</td><td>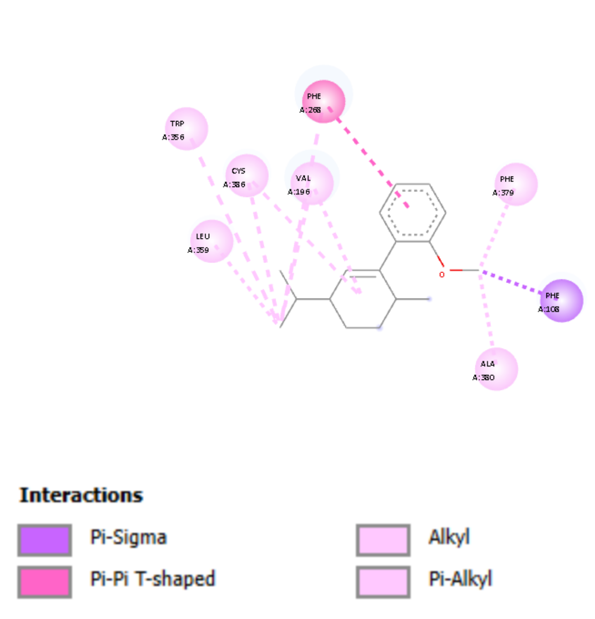
</td></tr>
<tr><td>Binding energy: -8.3</td><td>Binding energy: -8.3</td></tr>
</table>

<table>
<tr><th>7c (I)</th><th>ent-7c (I)</th></tr>
<tr><td>
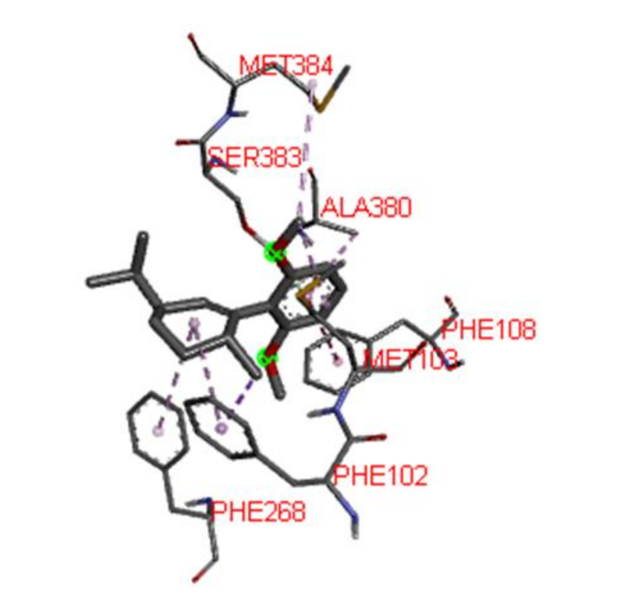

</td><td>
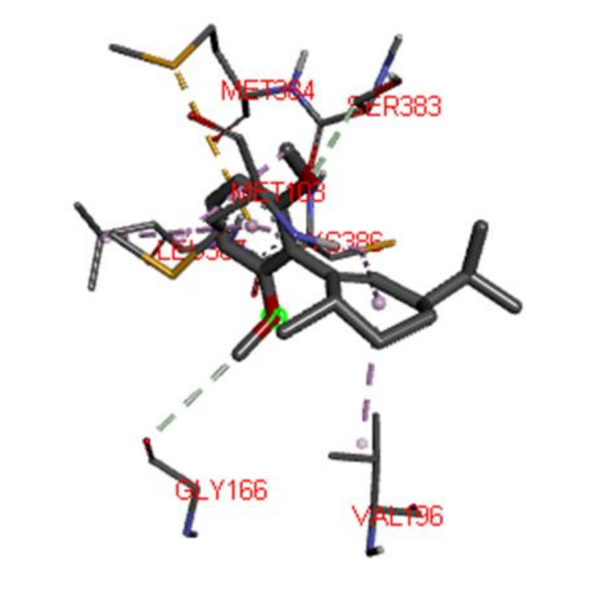

</td></tr>
<tr><td>
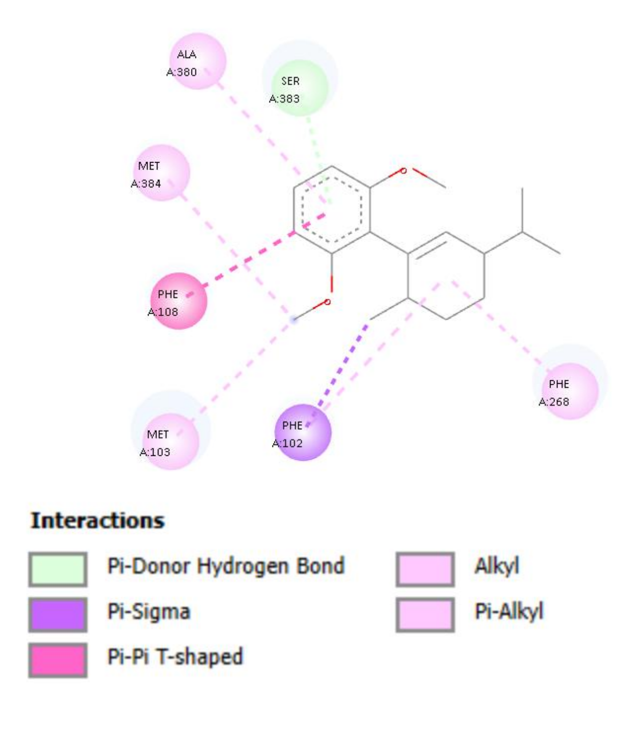

</td><td>
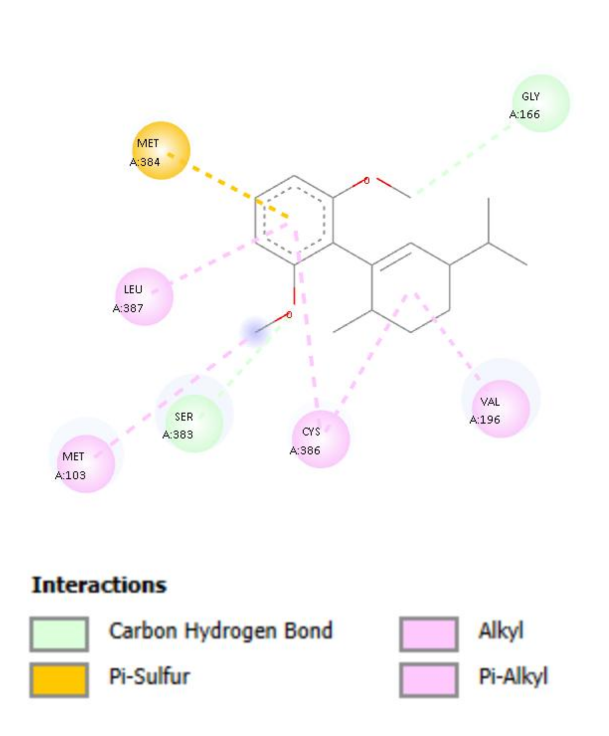

</td></tr>
<tr><td>Binding energy: -7.6</td><td>Binding energy: -7.4</td></tr>
</table>

| 7c (II) | ent-7c (II) |
| --- | --- |
| 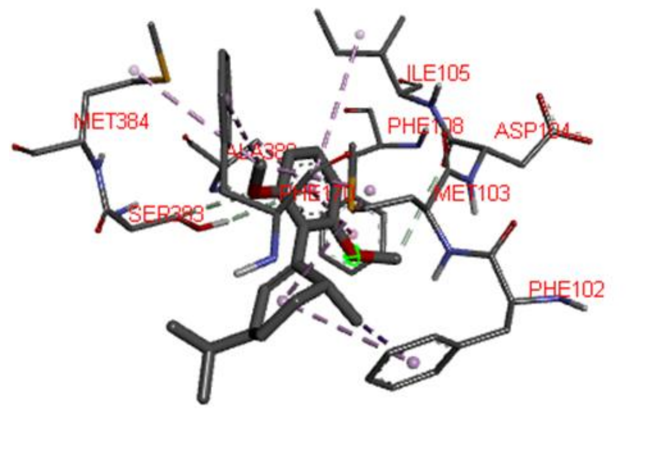<br> | 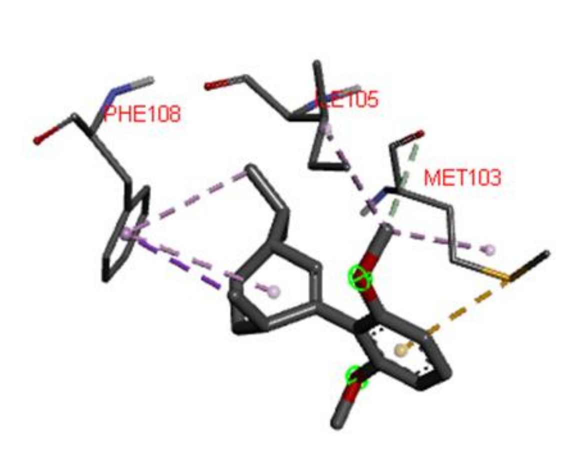<br> |
| 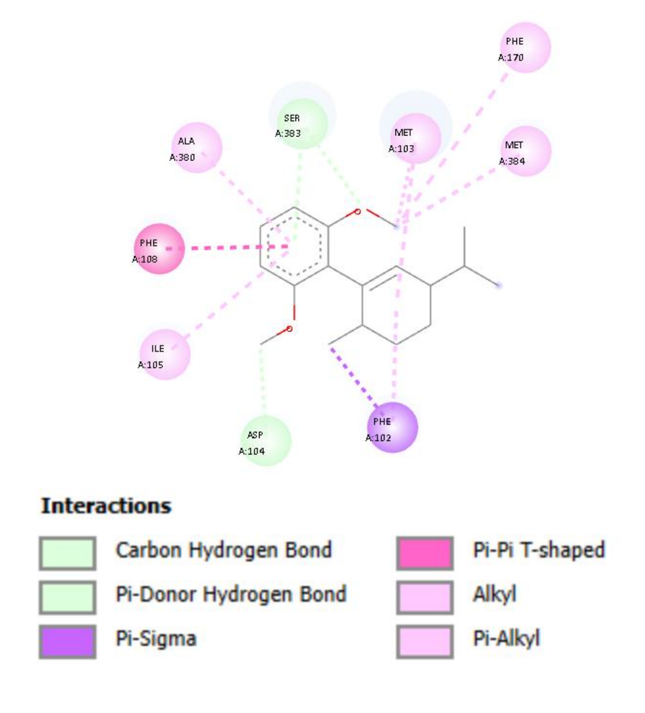<br> | 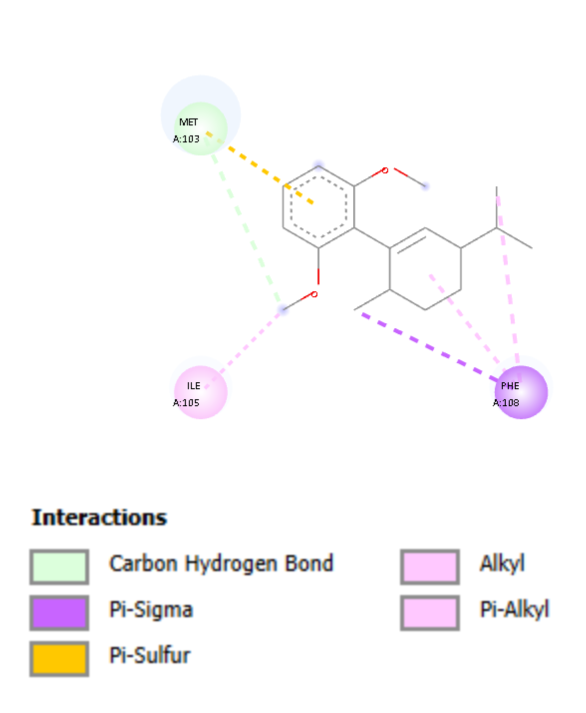<br> |
| **Binding energy: -7.6** | **Binding energy: -7.6** |

| 7d (I) | ent-7d (I) |
| --- | --- |
| 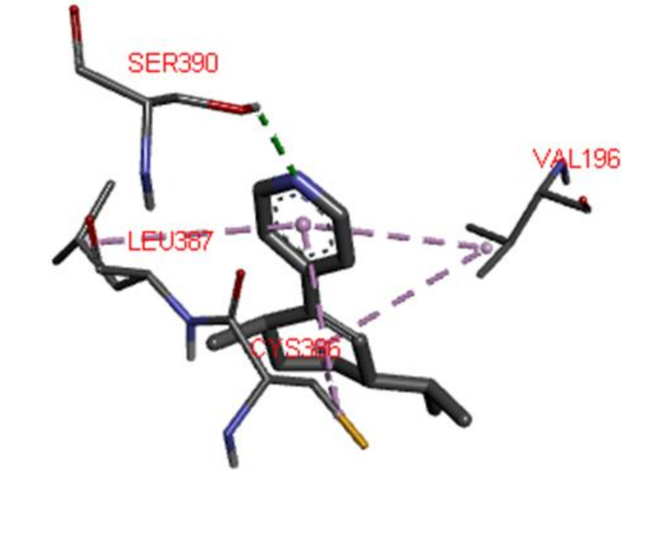 | 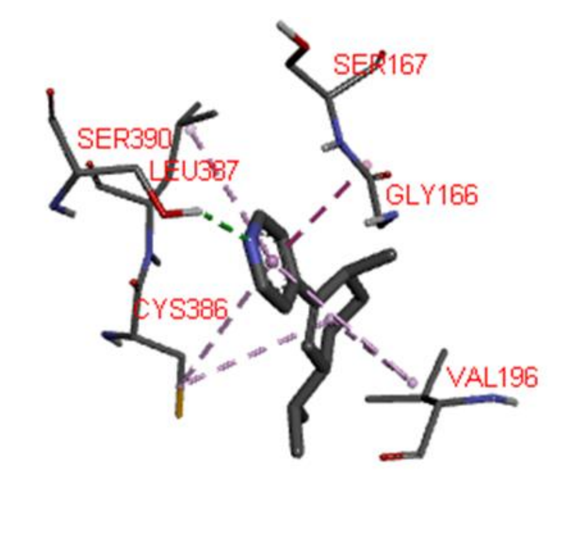 |
| 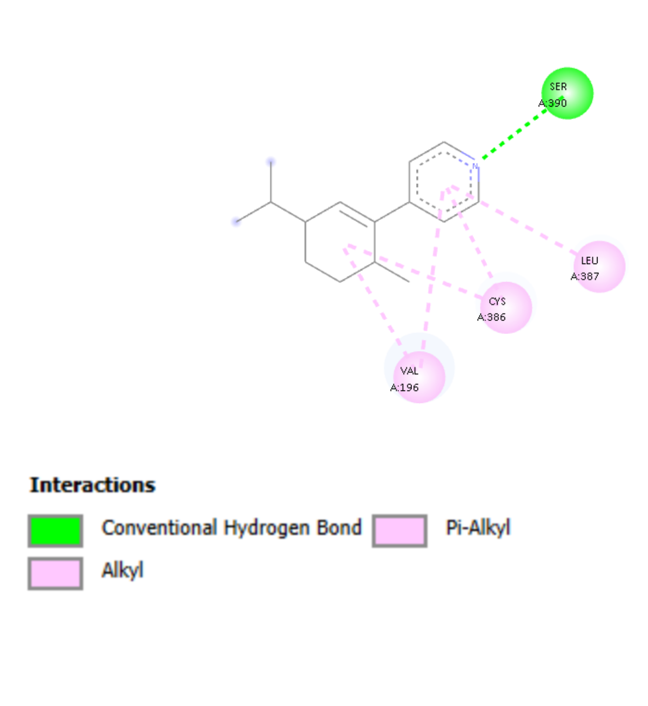 | 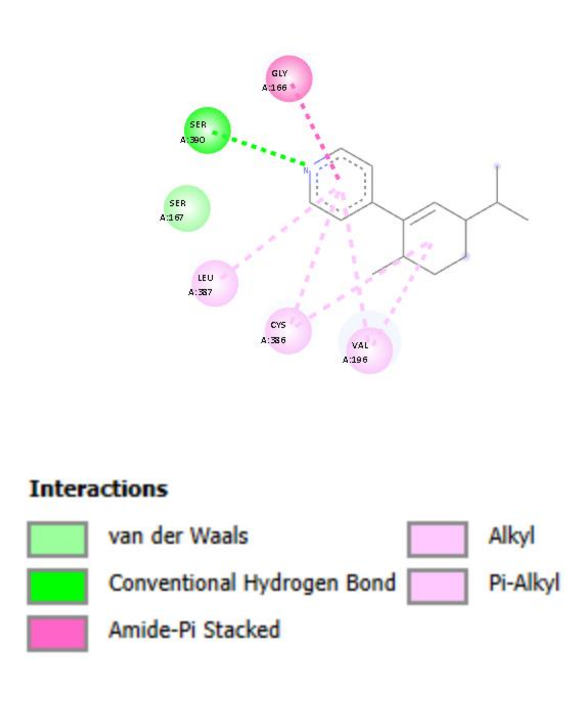 |
| **Binding energy: -8.5** | **Binding energy: -8.3** |

<table>
<tr><th>7d (II)</th><th>ent-7d (II)</th></tr>
<tr><td>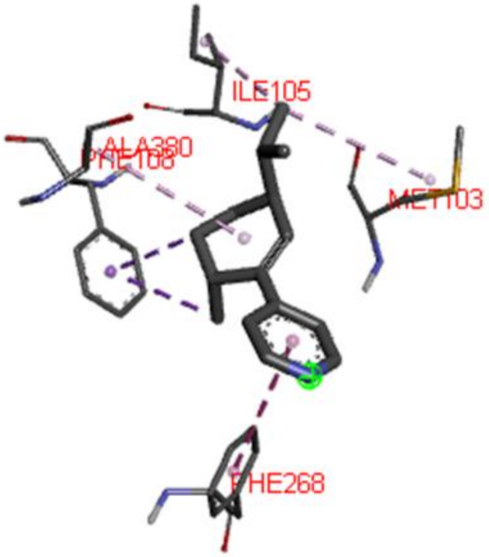
</td><td>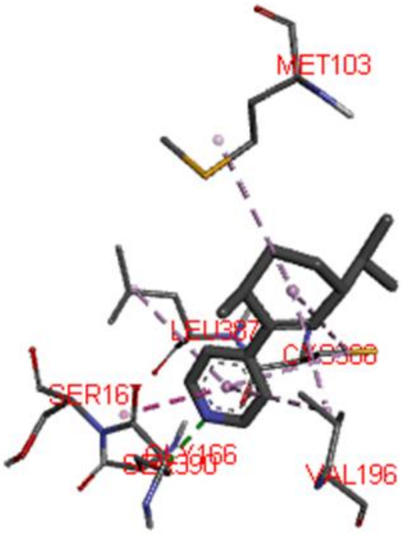
</td></tr>
<tr><td>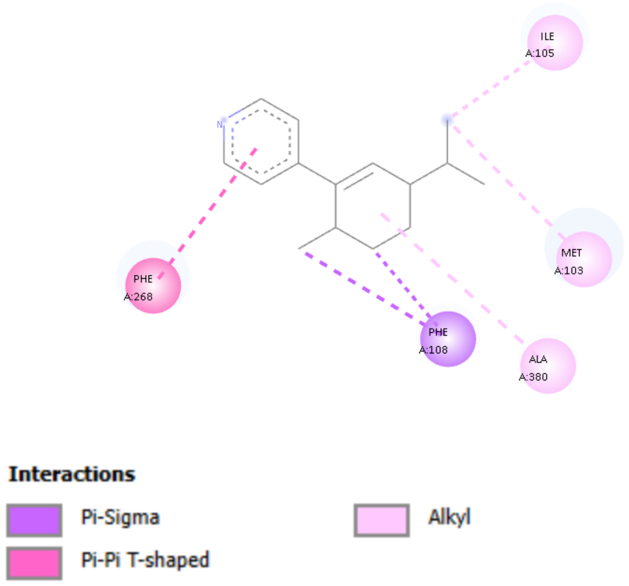
</td><td>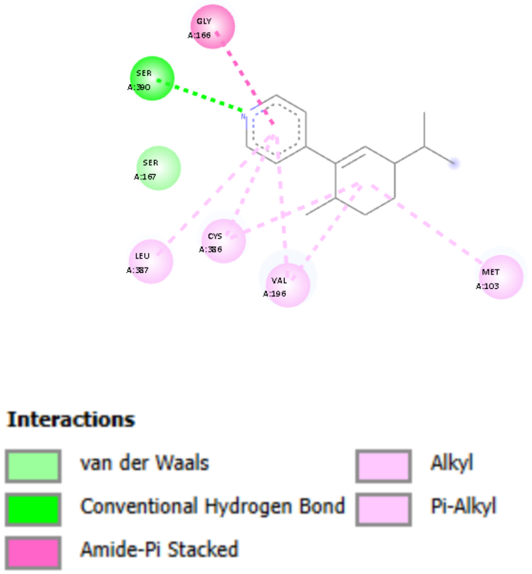
</td></tr>
<tr><td>Binding energy: -7.6</td><td>Binding energy: -7.9</td></tr>
</table>

| 7e (I) | ent-7e (I) |
|---|---|
| 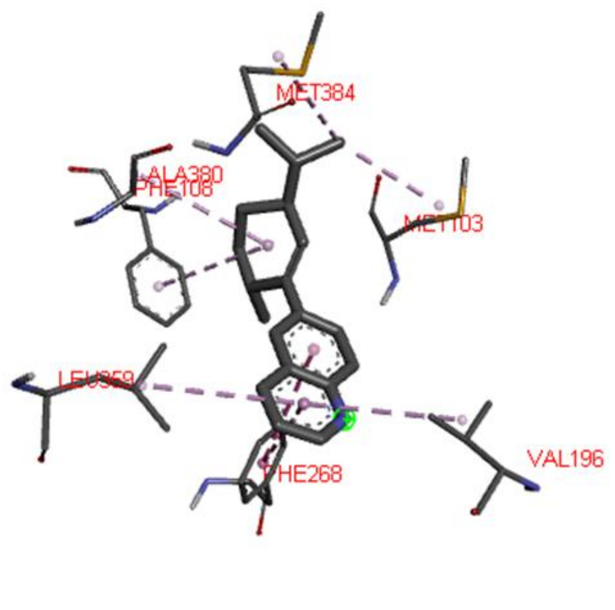  | 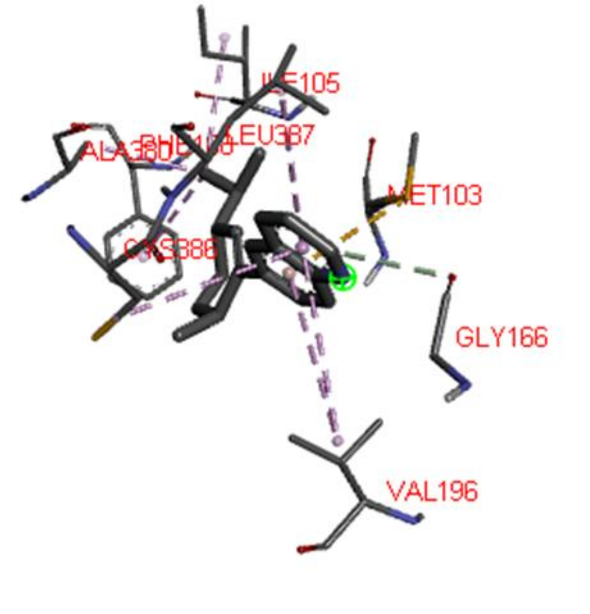  |
| 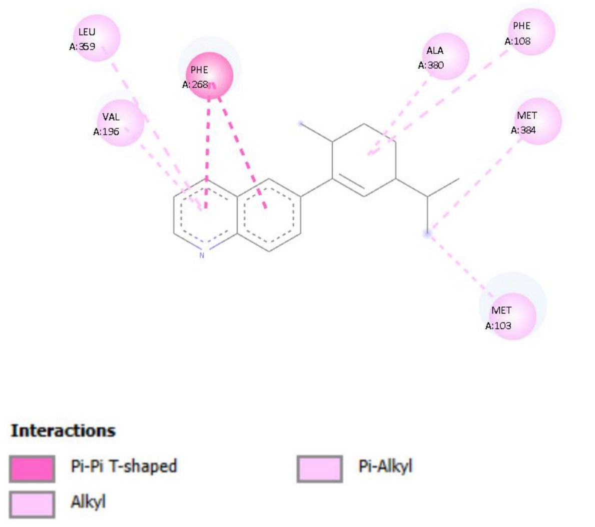  | 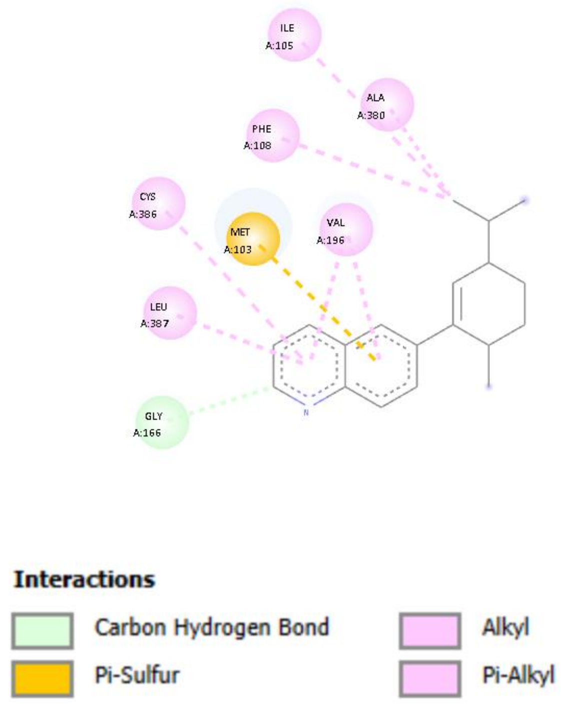  |
| **Binding energy: -9.8** | **Binding energy: -9.5** |

| 7e (II) | ent-7e (II) |
|---|---|
| 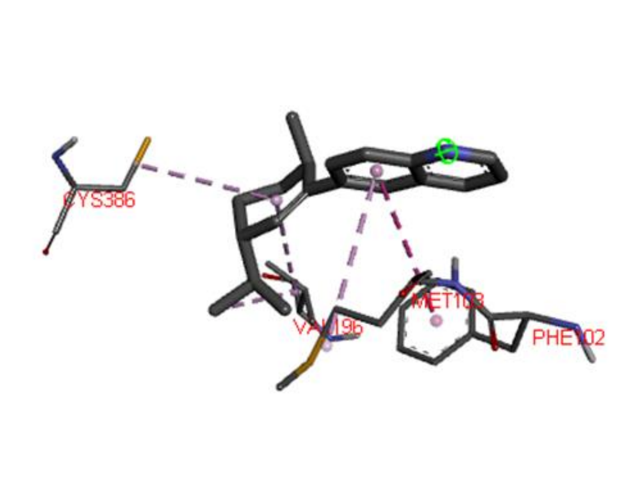 | 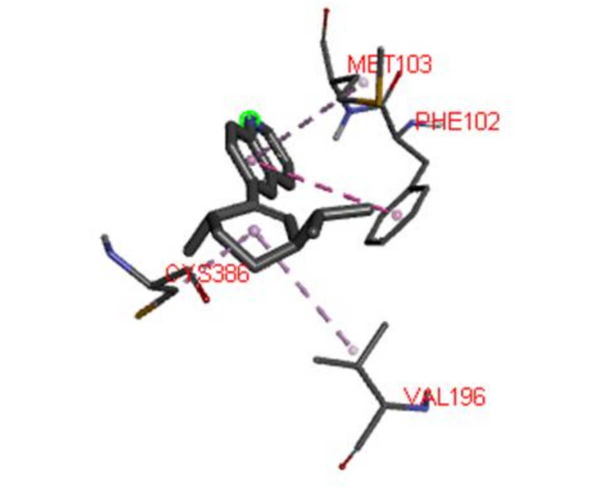 |
| 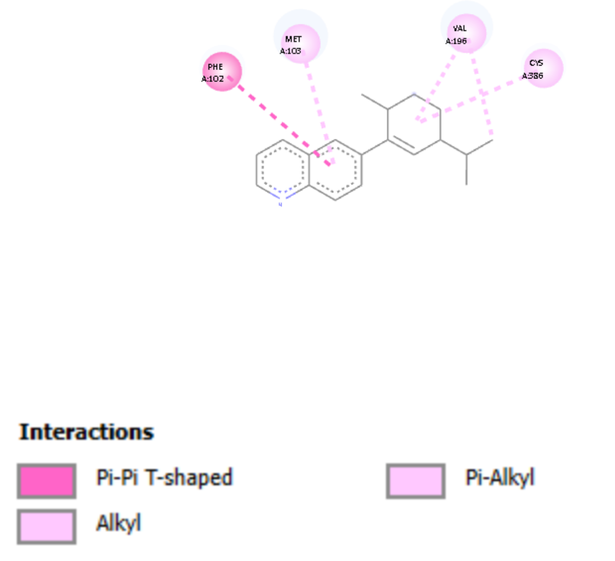 | 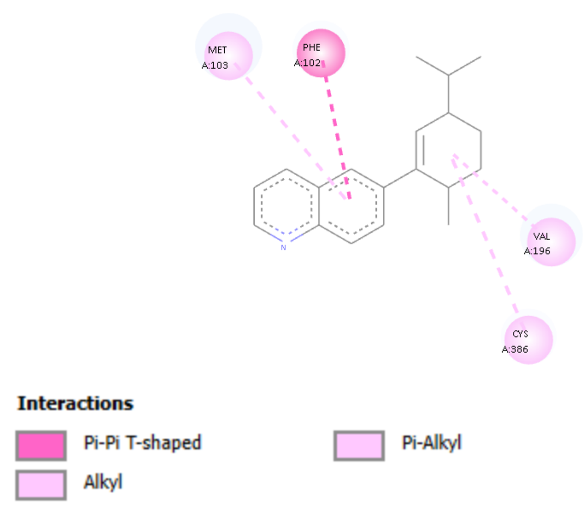 |
| **Binding energy: -9.5** | **Binding energy: -9.9** |

<table>
<tr><th>7f (I)</th><th>ent-7f (I)</th></tr>
<tr><td>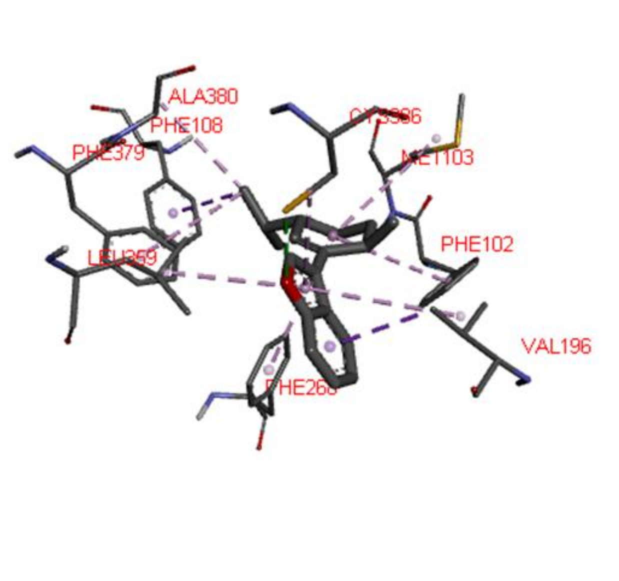
</td><td>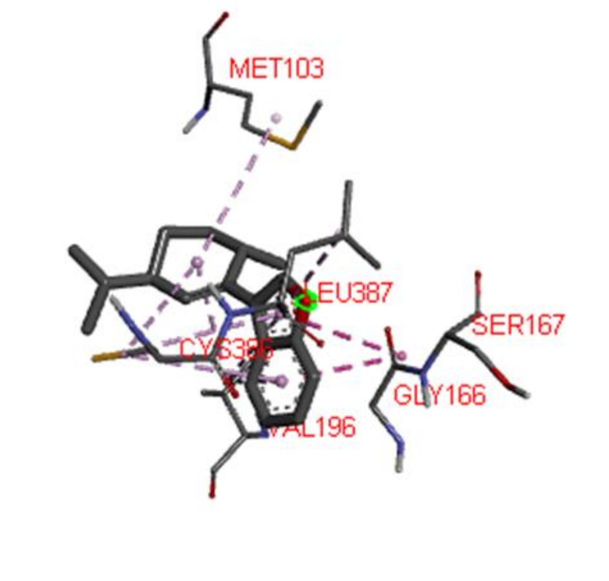
</td></tr>
<tr><td>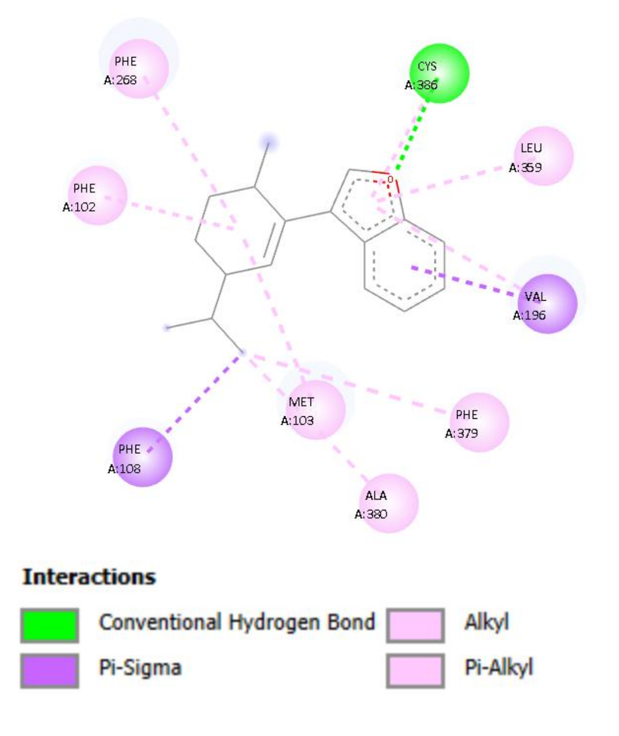
</td><td>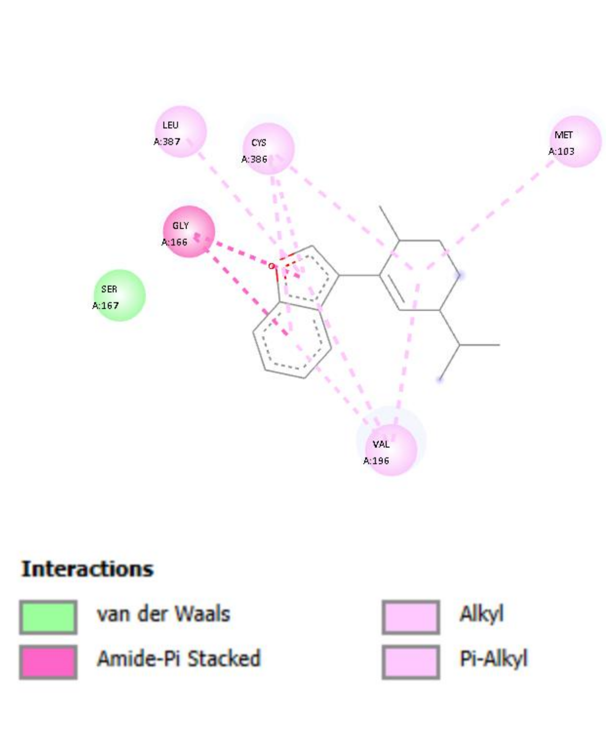
</td></tr>
<tr><td>Binding energy: -9.5</td><td>Binding energy: -9.5</td></tr>
</table>

| 7f (II) | ent-7f (II) |
|---|---|
| 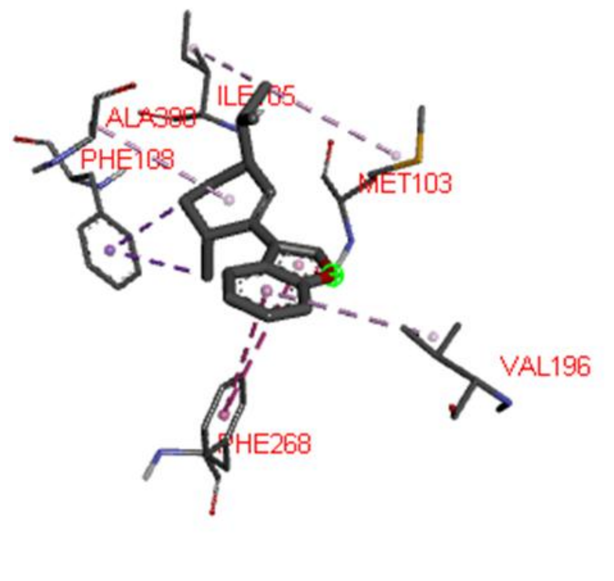 | 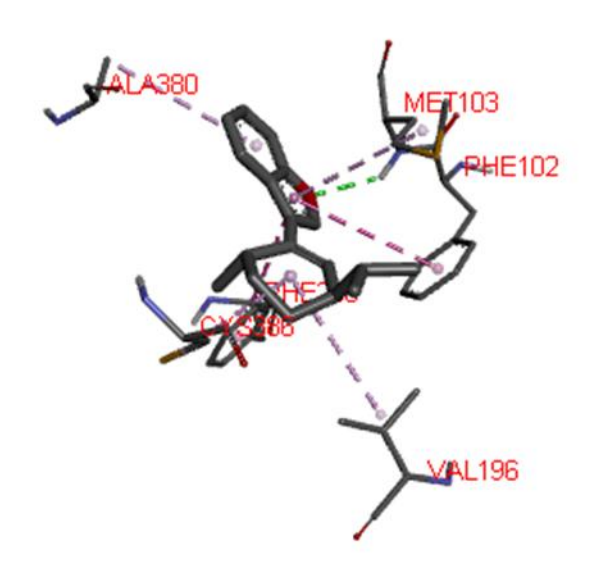 |
| 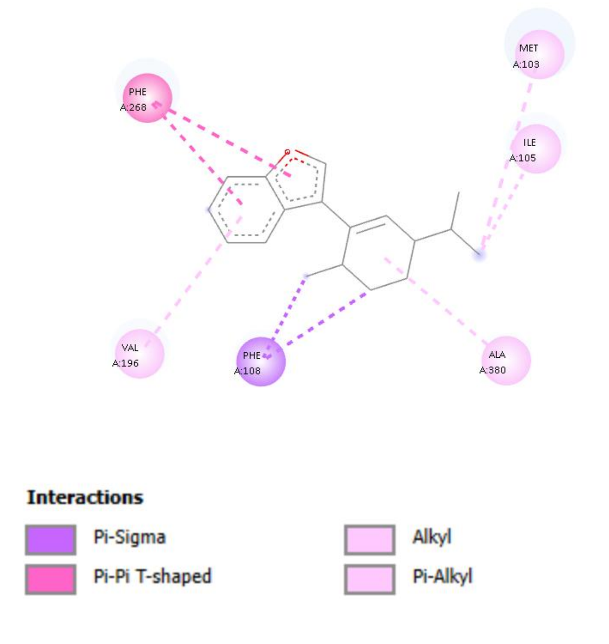 | 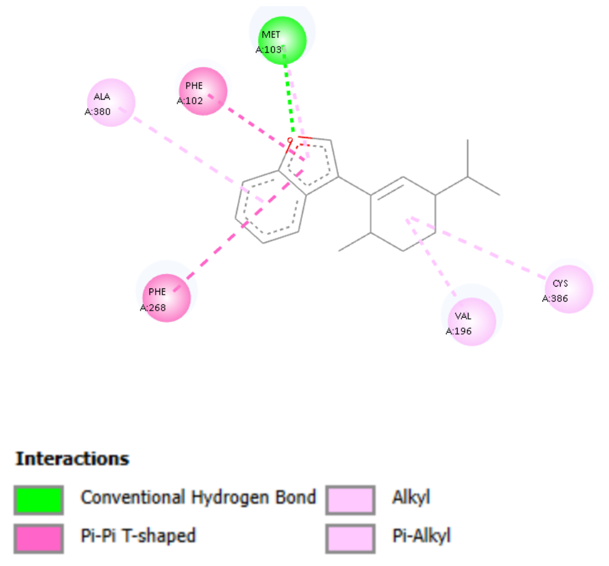 |
| **Binding energy: -9.0** | **Binding energy: -9.1** |

• *Synthesized Ligands of Series **8**/ent-**8** with CB1 Receptor*

<table>
<tr><th>8a (I)</th><th>ent-8a (I)</th></tr>
<tr><td>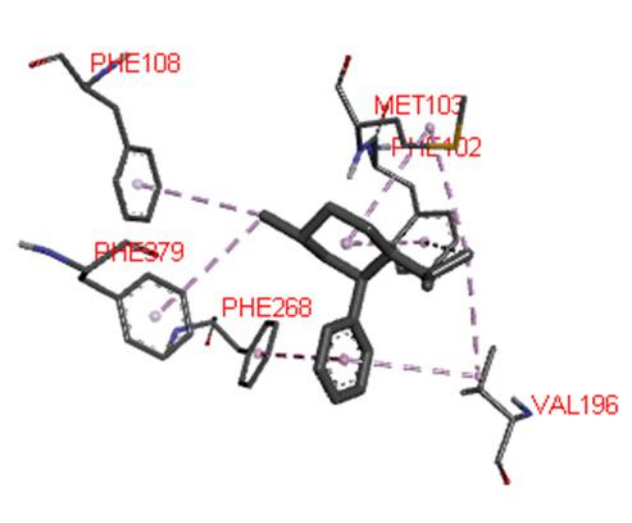
</td><td>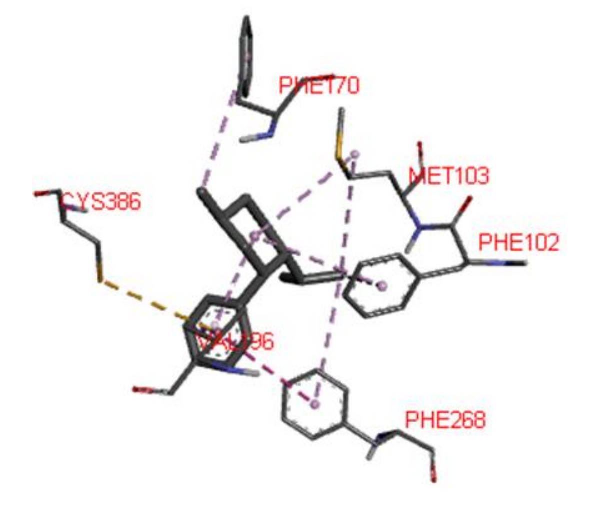
</td></tr>
<tr><td>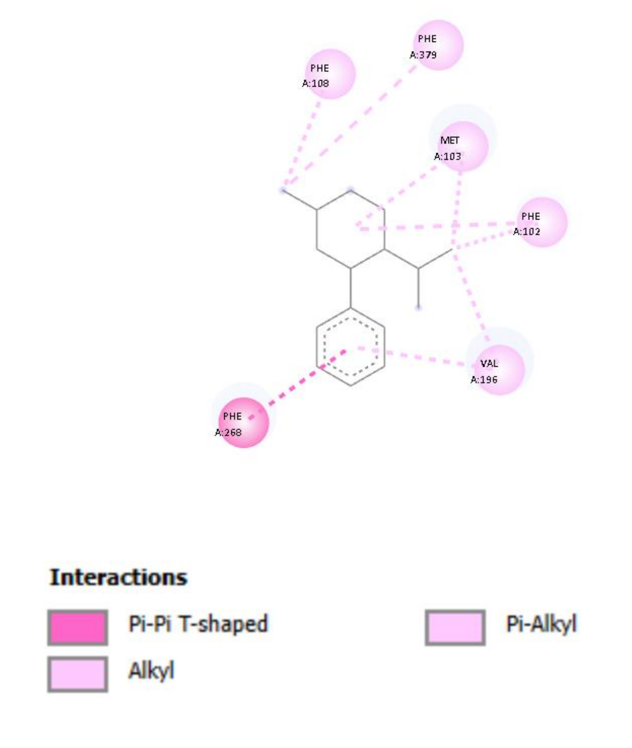
</td><td>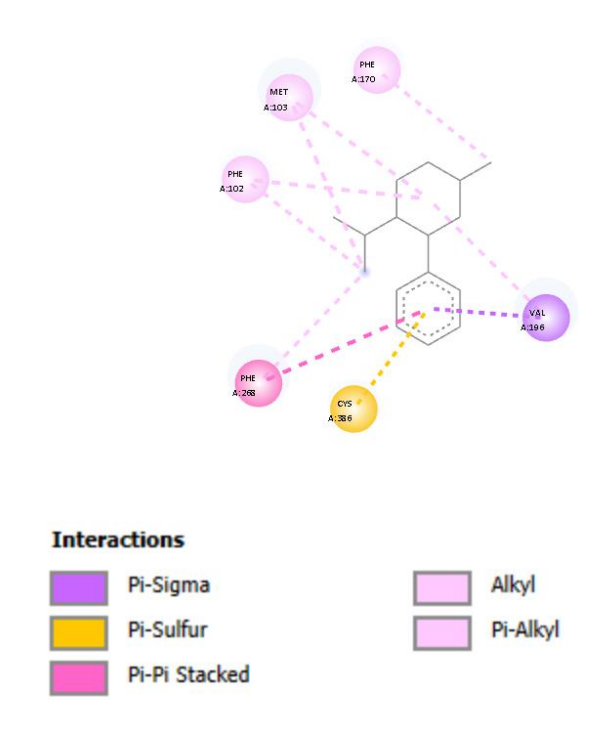
</td></tr>
<tr><td>Binding energy: -8.3</td><td>Binding energy: -8.2</td></tr>
</table>

| 8a (II) | ent-8a (II) |
| --- | --- |
| 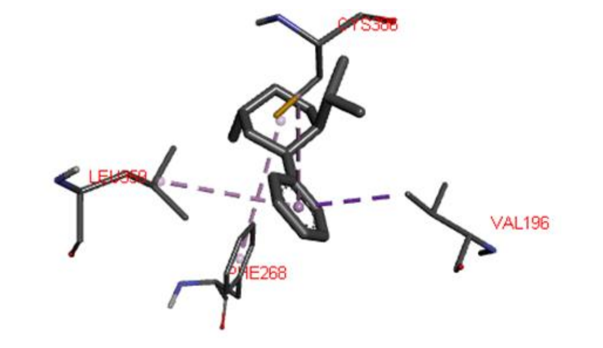  | 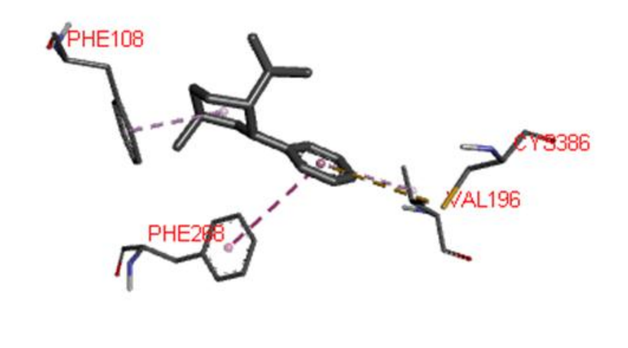  |
| 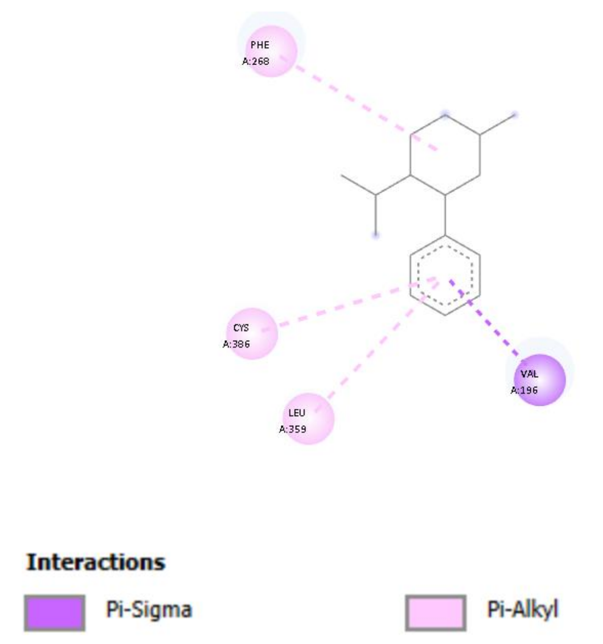  | 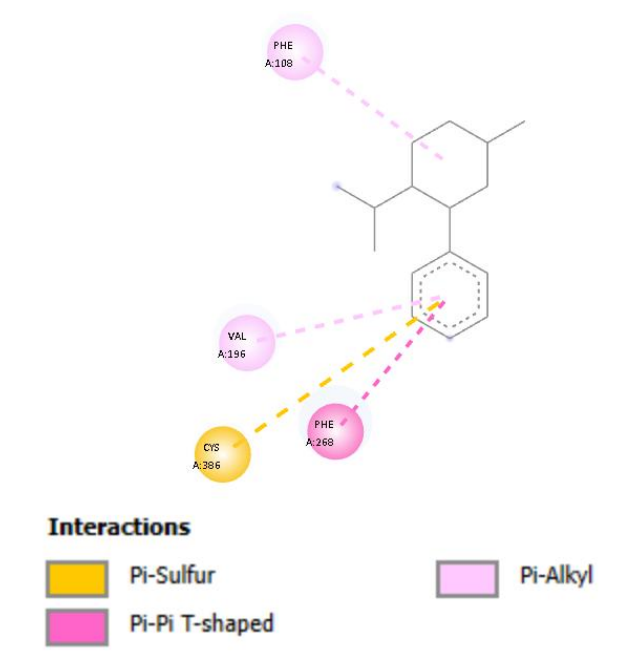  |
| **Binding energy: -7.6** | **Binding energy: -7.8** |

<table>
<tr><th>8b (I)</th><th>ent-8b (I)</th></tr>
<tr><td>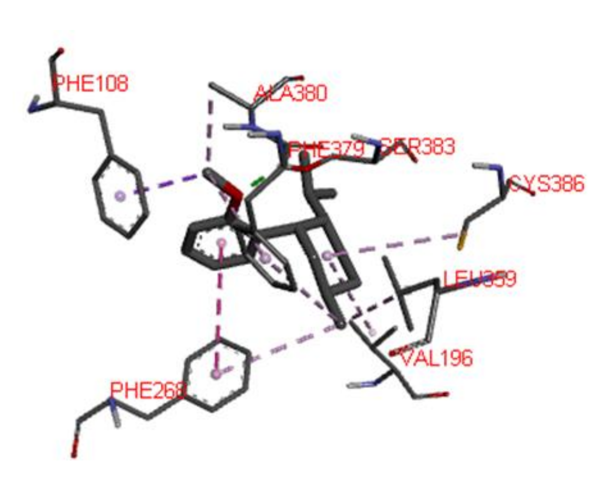
</td><td>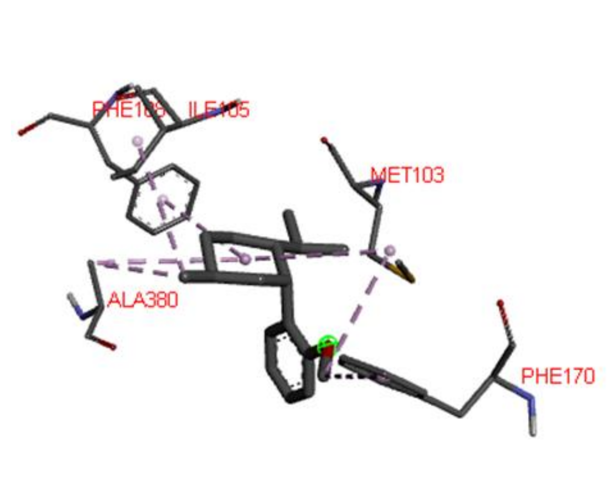
</td></tr>
<tr><td>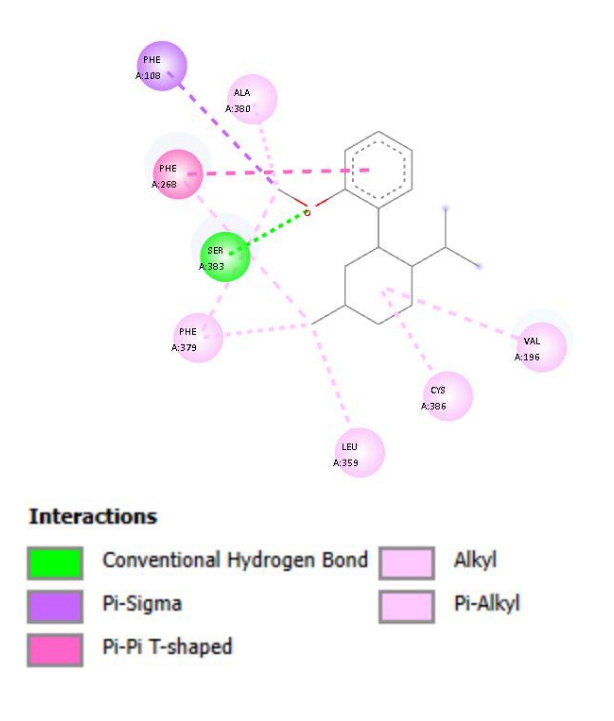
</td><td>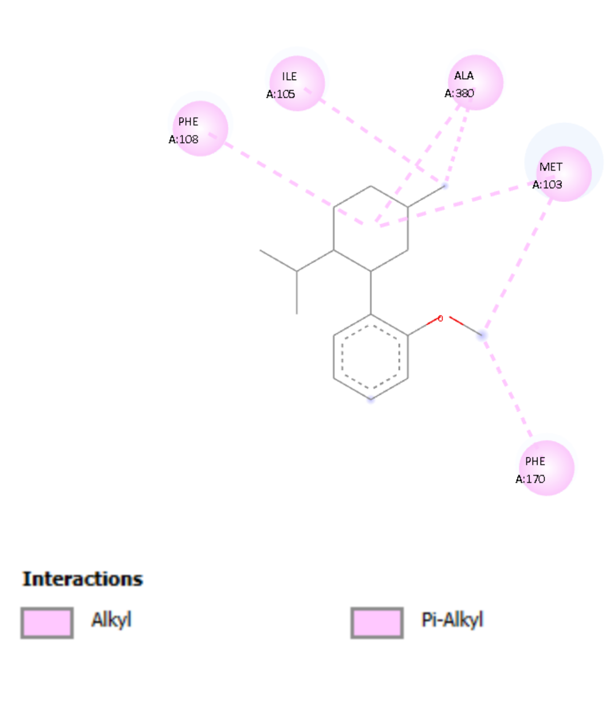
</td></tr>
<tr><td>Binding energy: -7.6</td><td>Binding energy: -7.8</td></tr>
</table>

| 8b (II) | ent-8b (II) |
|---|---|
| 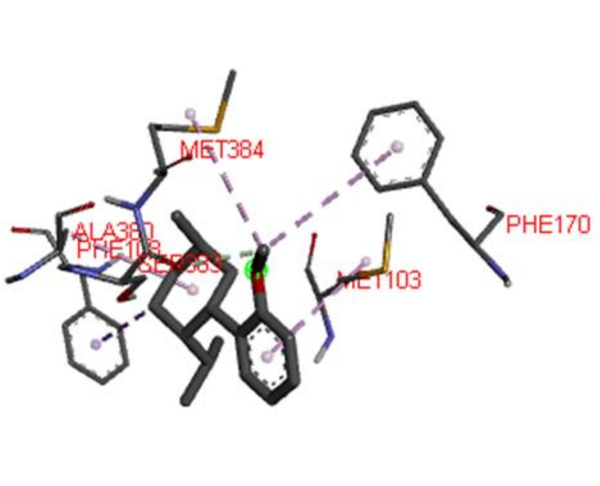 | 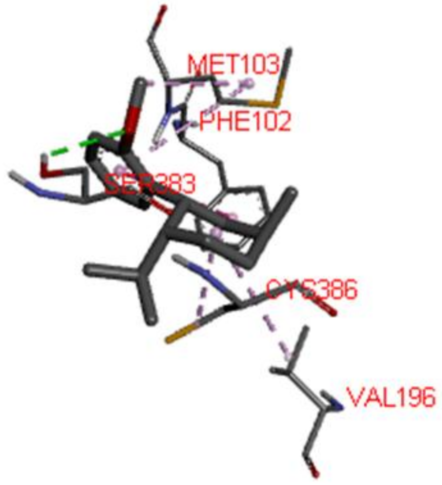 |
| 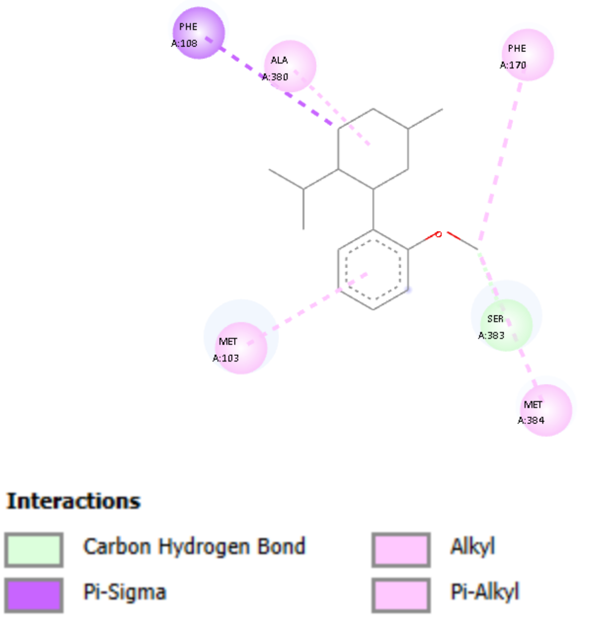 | 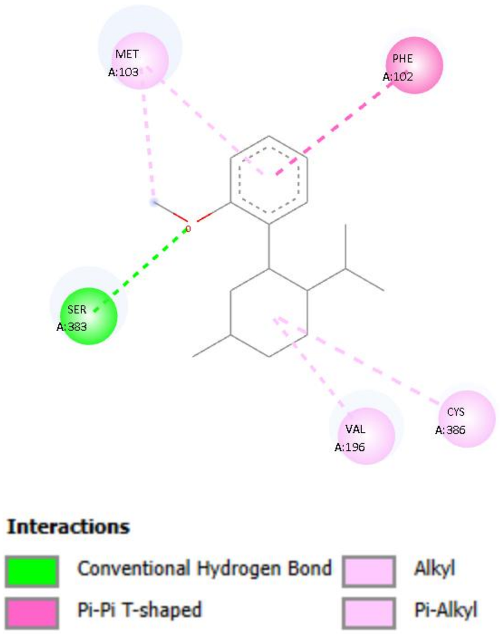 |
| **Binding energy: -8.4** | **Binding energy: -7.6** |

<table>
<tr><th>8c (I)</th><th>ent-8c (I)</th></tr>
<tr><td>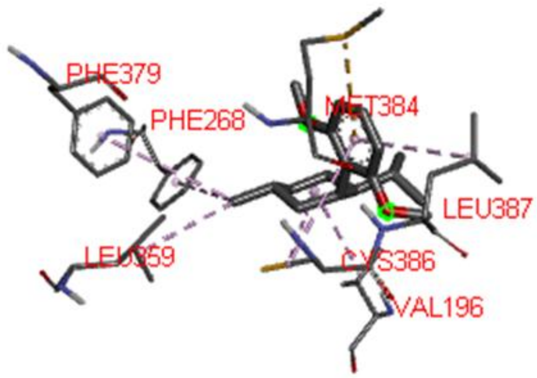
</td><td>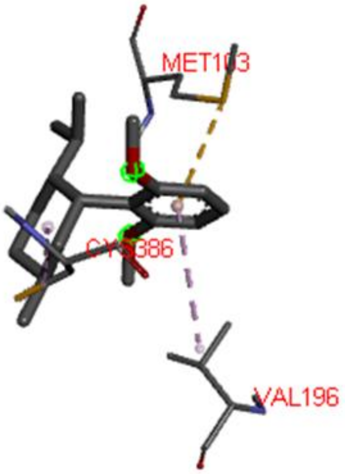
</td></tr>
<tr><td>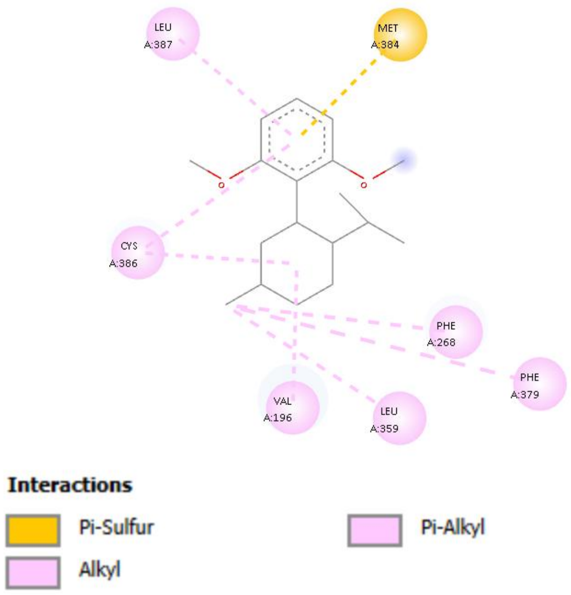
</td><td>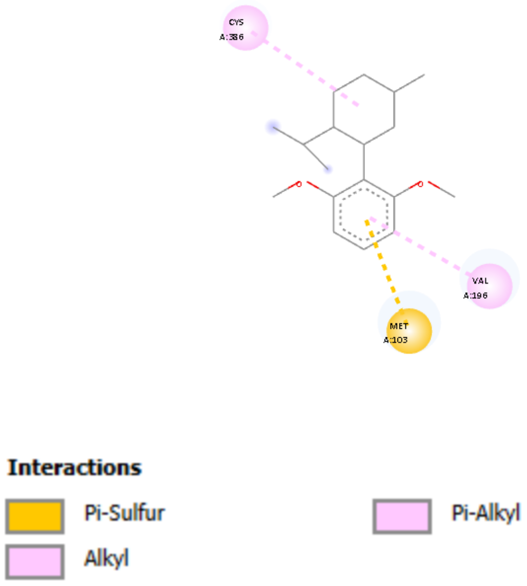
</td></tr>
<tr><td>Binding energy: -7.1</td><td>Binding energy: -6.8</td></tr>
</table>

<table>
<tr><th>8c (II)</th><th>ent-8c (II)</th></tr>
<tr><td>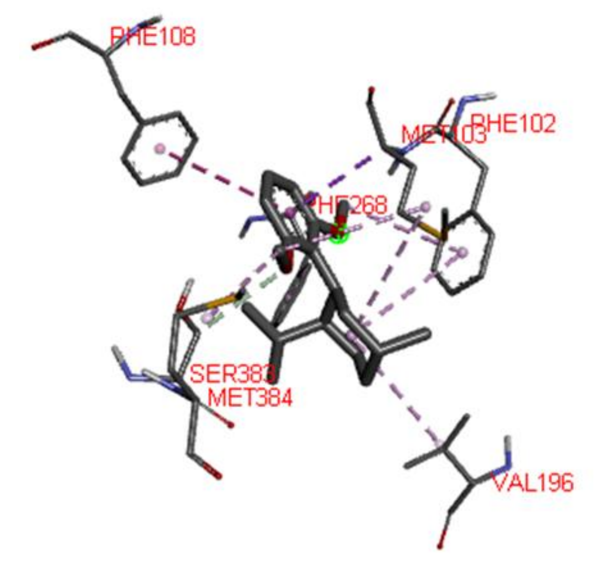
</td><td>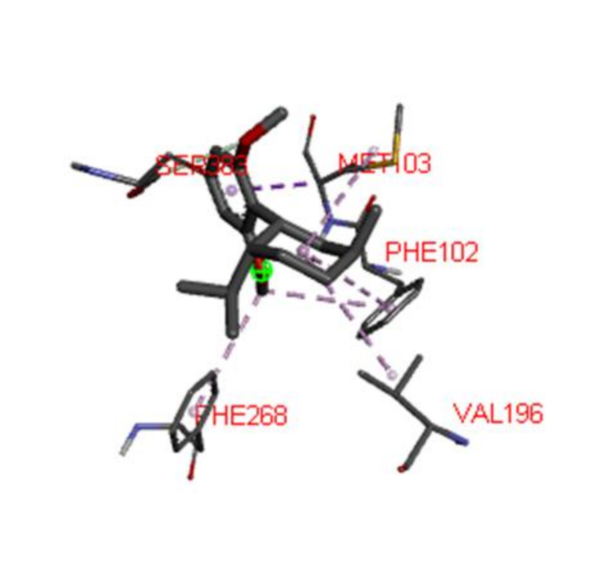
</td></tr>
<tr><td>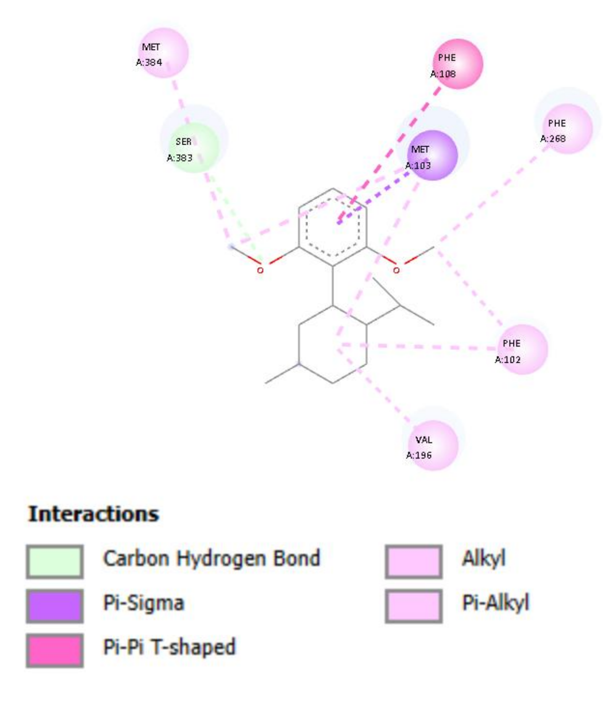
</td><td>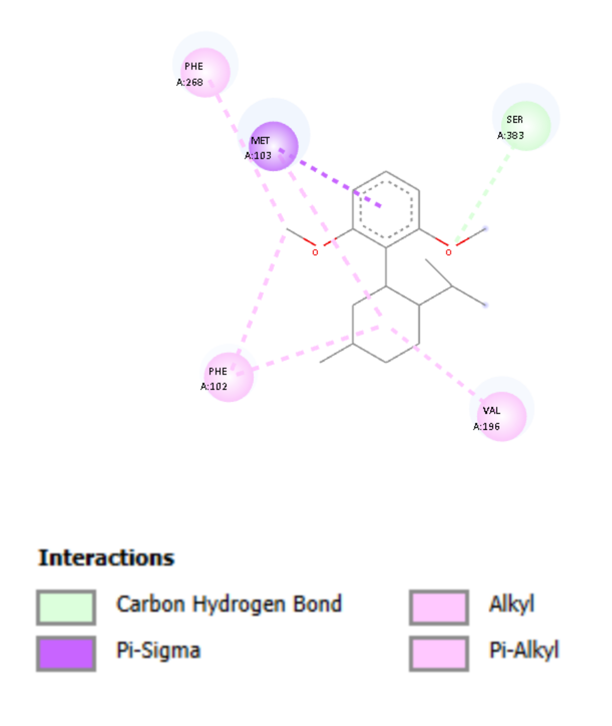
</td></tr>
<tr><td>Binding energy: -6.7</td><td>Binding energy: -6.3</td></tr>
</table>

<table>
<tr><th>8d (I)</th><th>ent-8d (I)</th></tr>
<tr><td>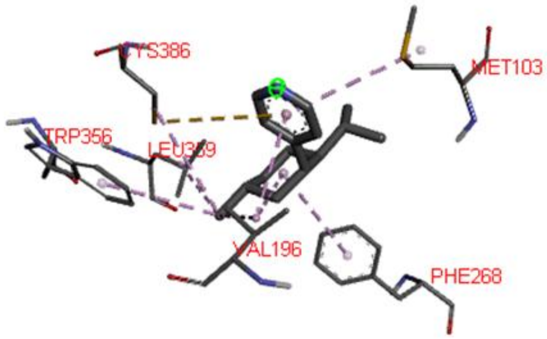
</td><td>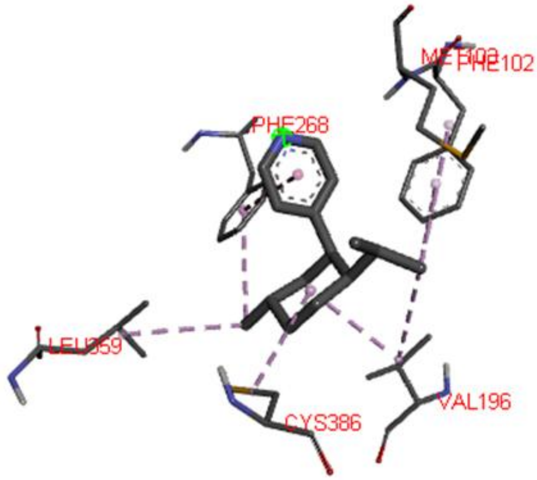
</td></tr>
<tr><td>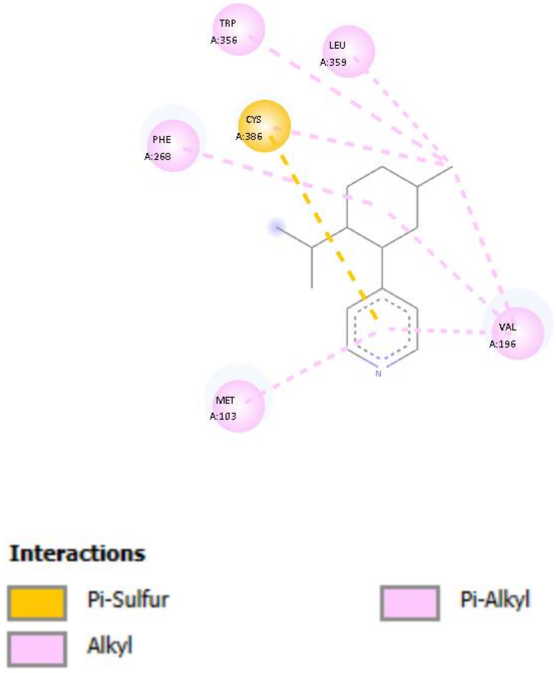
</td><td>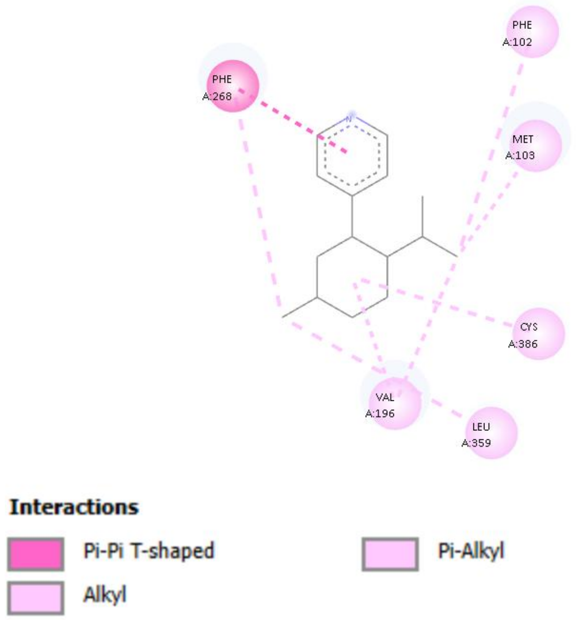
</td></tr>
<tr><td>Binding energy: -7.9</td><td>Binding energy: -7.7</td></tr>
</table>

<table>
<tr><th>8d (II)</th><th>ent-8d (II)</th></tr>
<tr><td>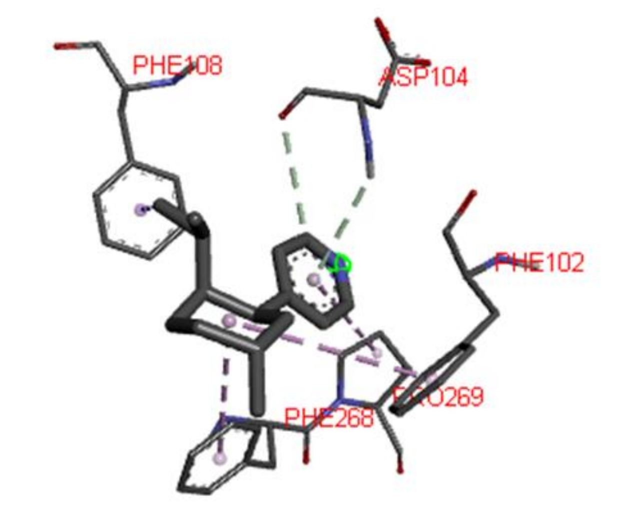
</td><td>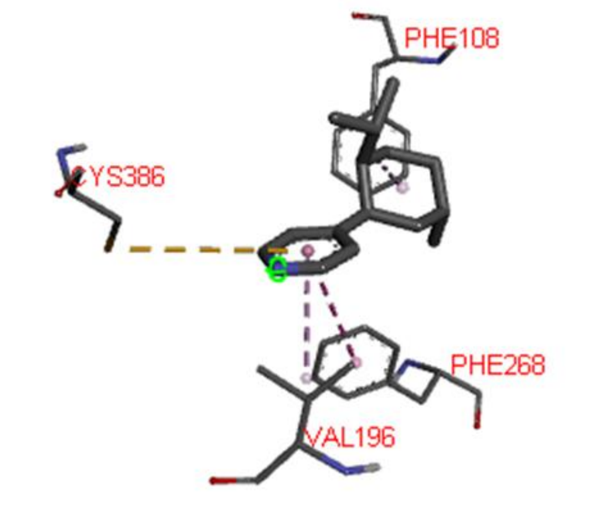
</td></tr>
<tr><td>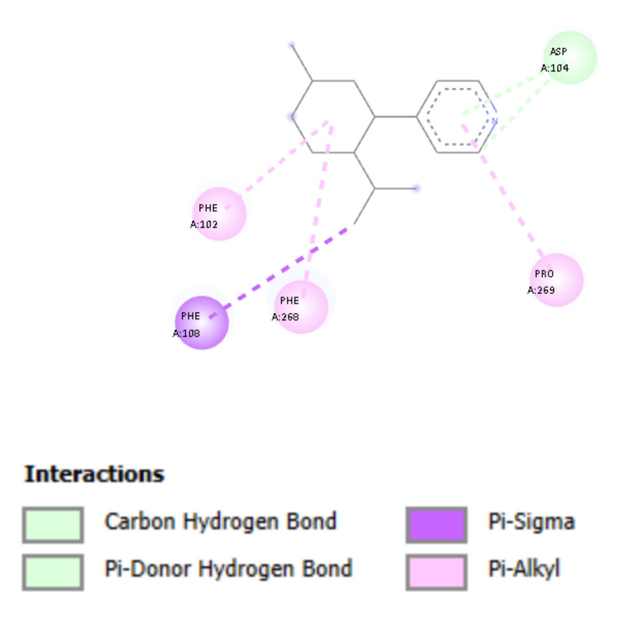
</td><td>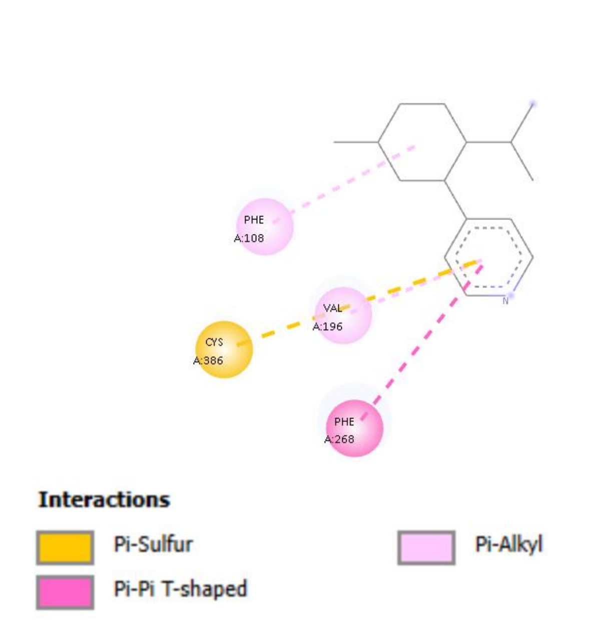
</td></tr>
<tr><td>Binding energy: -8.2</td><td>Binding energy: -7.4</td></tr>
</table>

<table>
<tr><th>8e (I)</th><th>ent-8e (I)</th></tr>
<tr><td>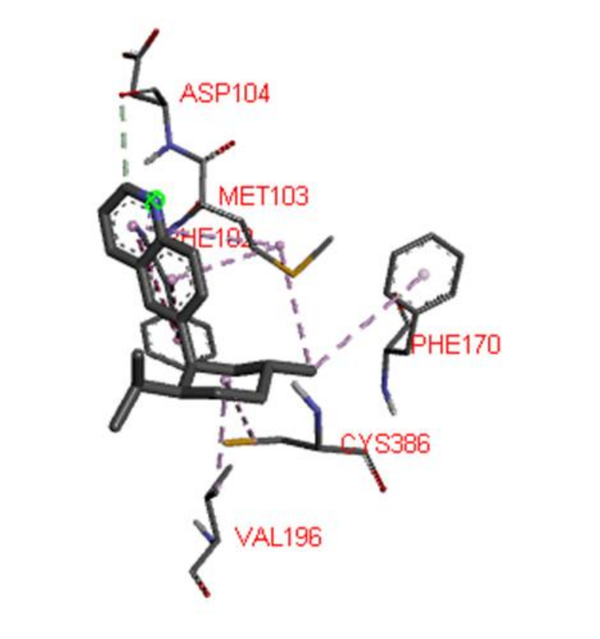</td><td>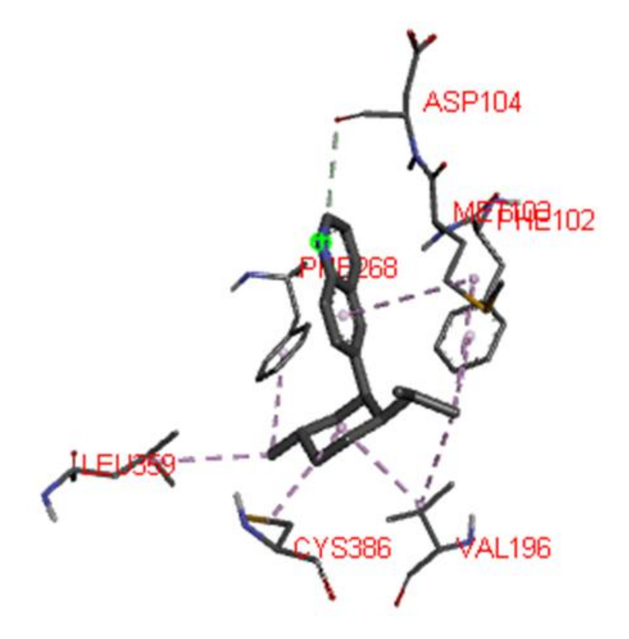</td></tr>
<tr><td>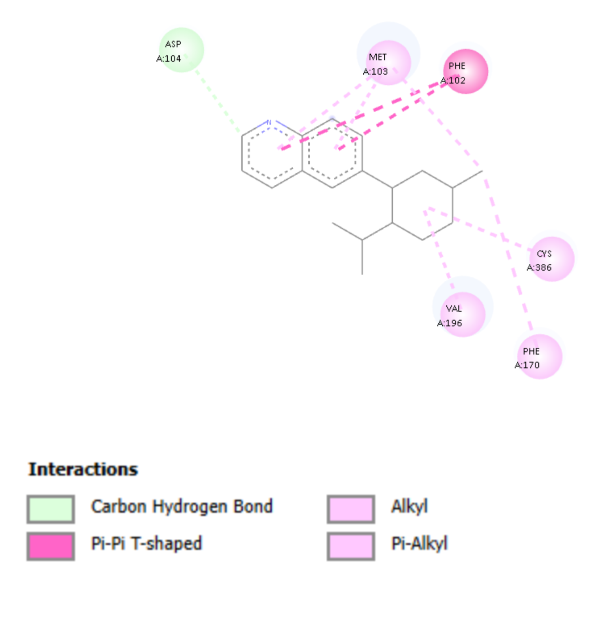</td><td>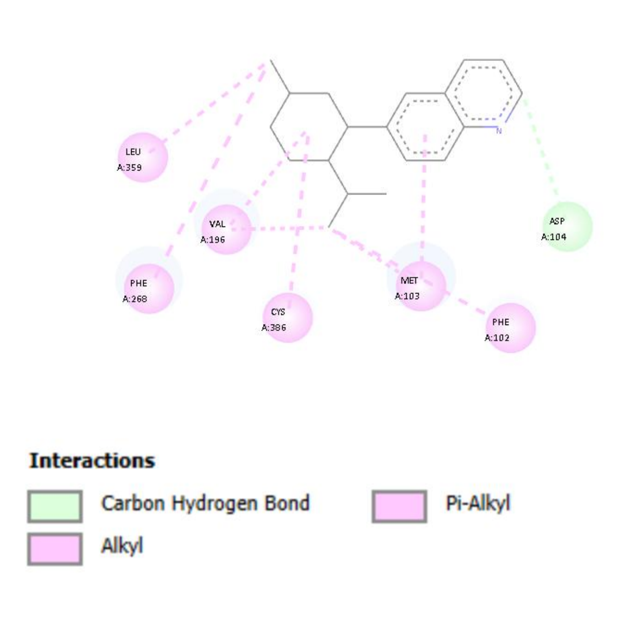</td></tr>
<tr><td>Binding energy: -9.6</td><td>Binding energy: -9.6</td></tr>
</table>

<table>
<tr><th>8e (II)</th><th>ent-8e (II)</th></tr>
<tr><td>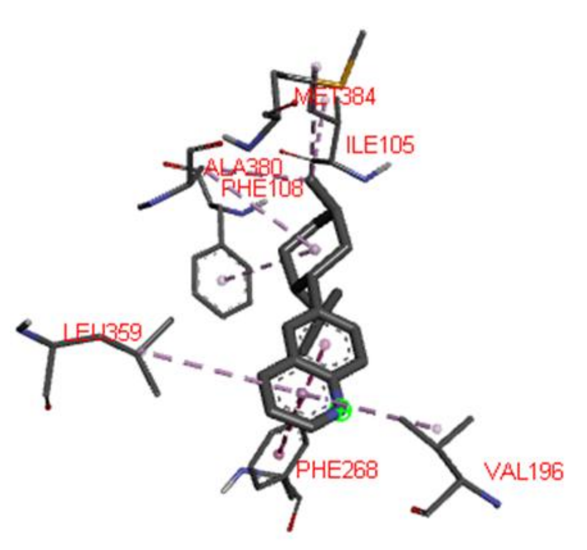
</td><td>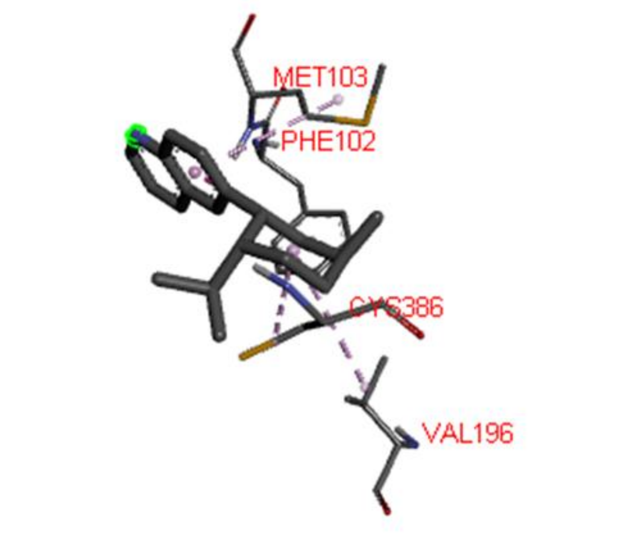
</td></tr>
<tr><td>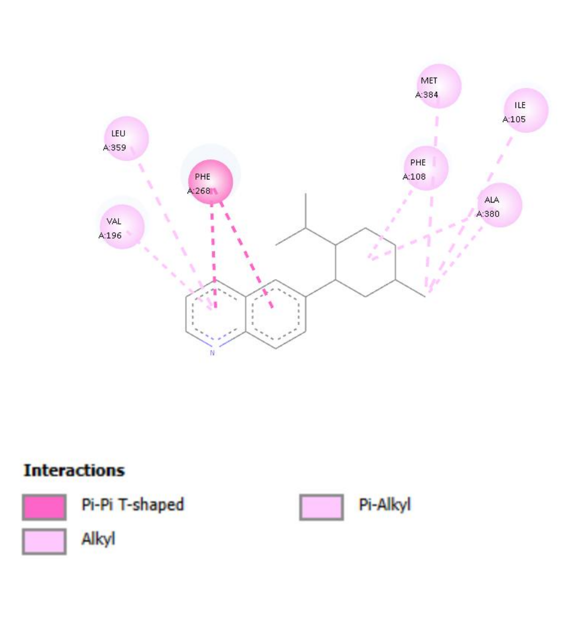
</td><td>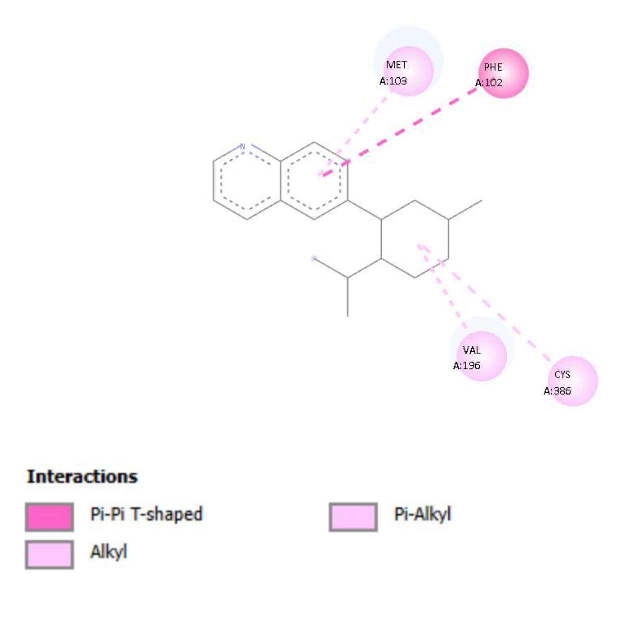
</td></tr>
<tr><td>Binding energy: -9.6</td><td>Binding energy: -9.2</td></tr>
</table>

| 8f (I) | ent-8f (I) |
|---|---|
| 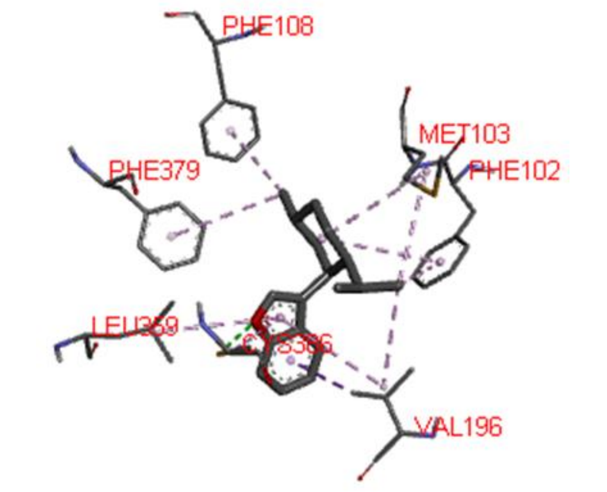 | 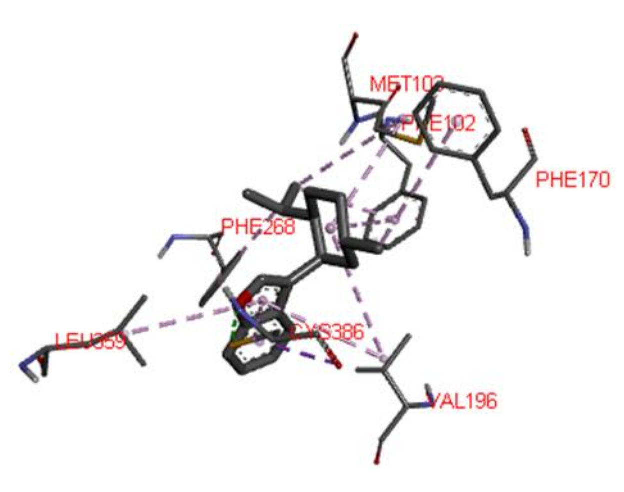 |
| 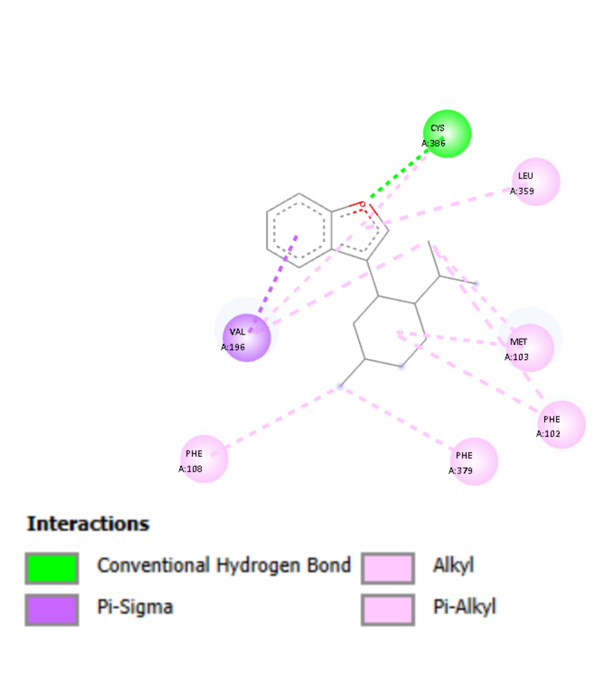 | 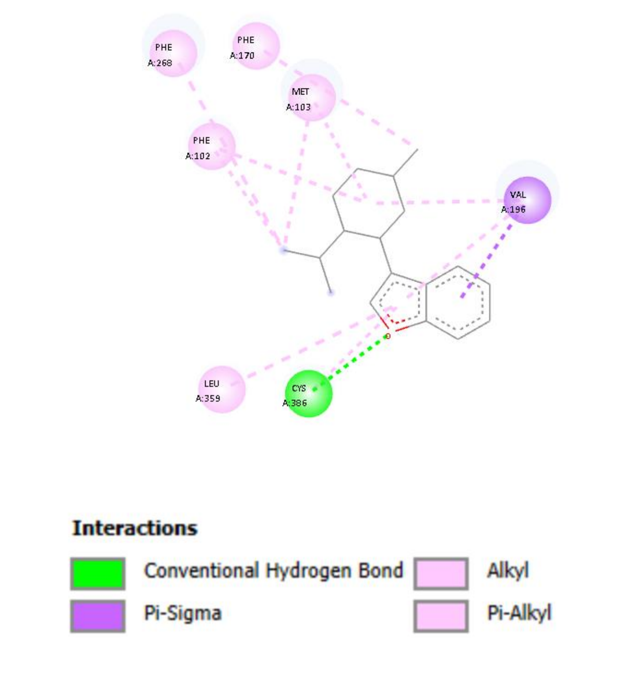 |
| **Binding energy: -8.9** | **Binding energy: -9.0** |

| 8f (II) | ent-8f (II) |
|---|---|
| 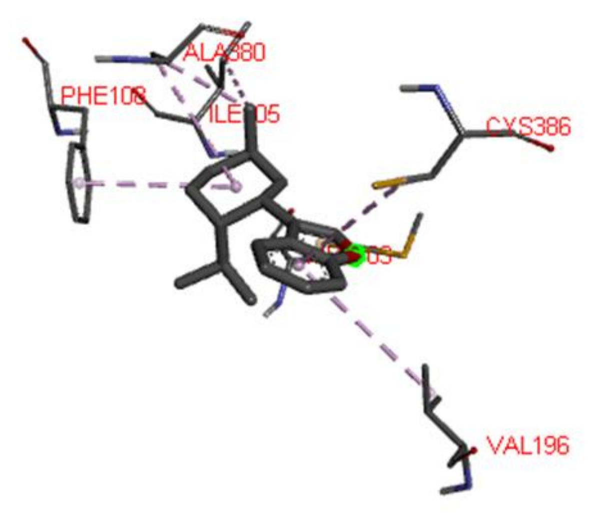 | 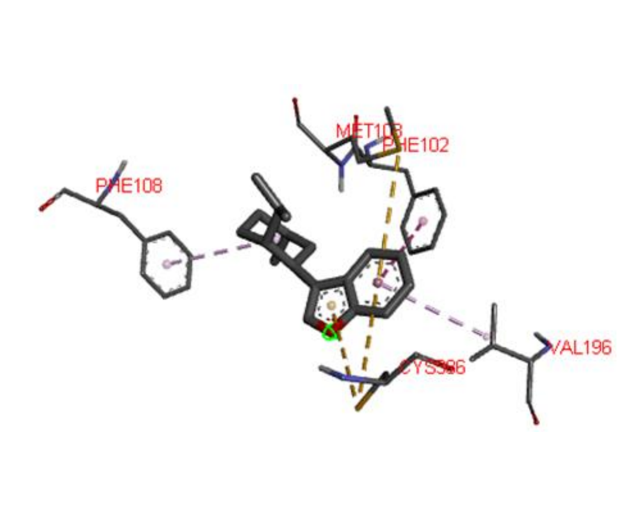 |
| 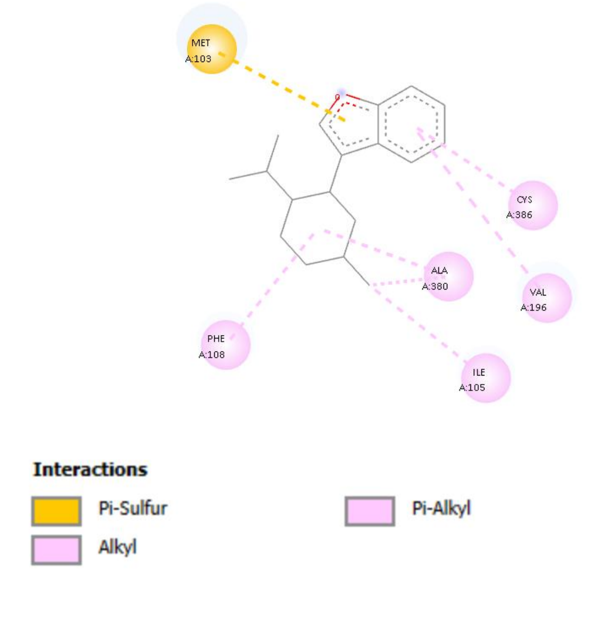 | 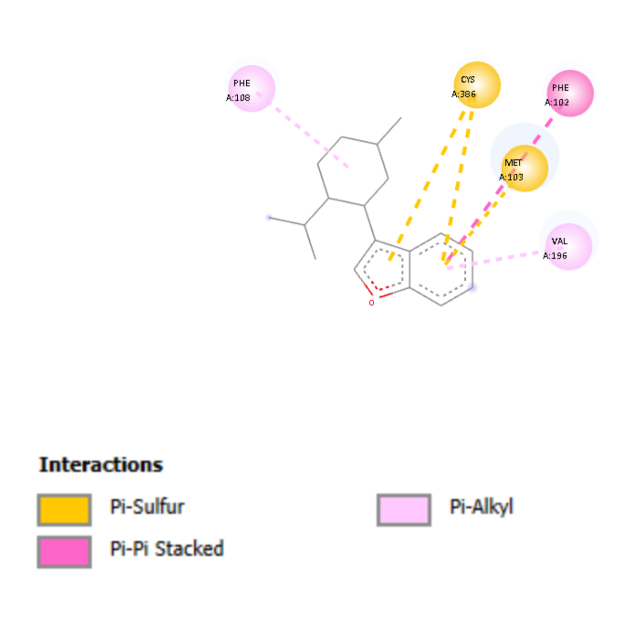 |
| **Binding energy: -9.0** | **Binding energy: -8.7** |

• *Synthesized Ligands of Series **9**/ent-**9** with CB1 Receptor*

<table>
<tr><th>9a (I)</th><th>ent-9a (I)</th></tr>
<tr><td>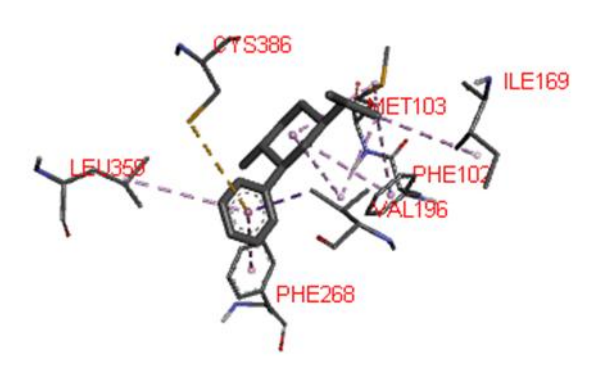
</td><td>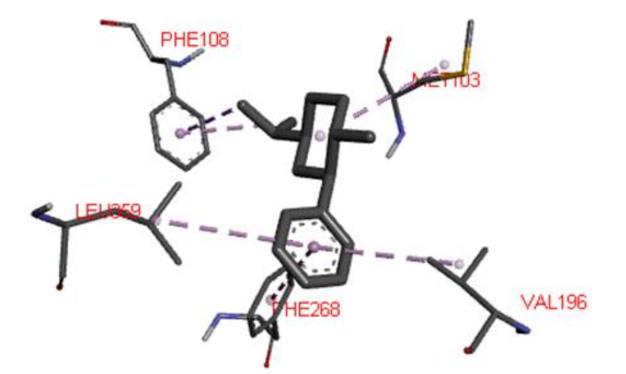
</td></tr>
<tr><td>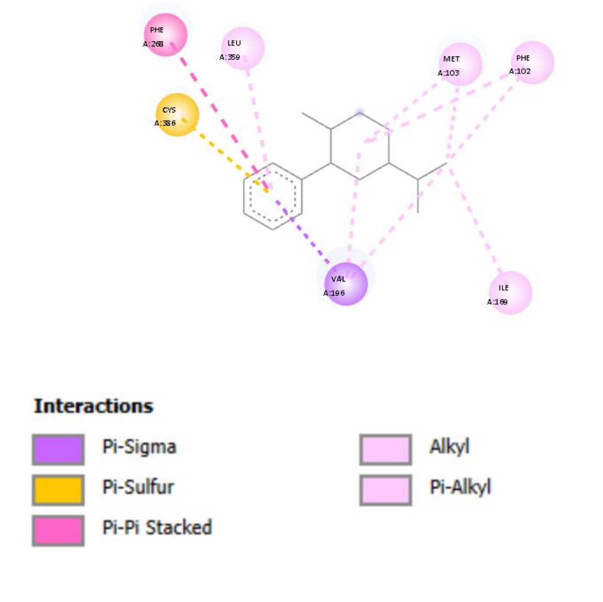
</td><td>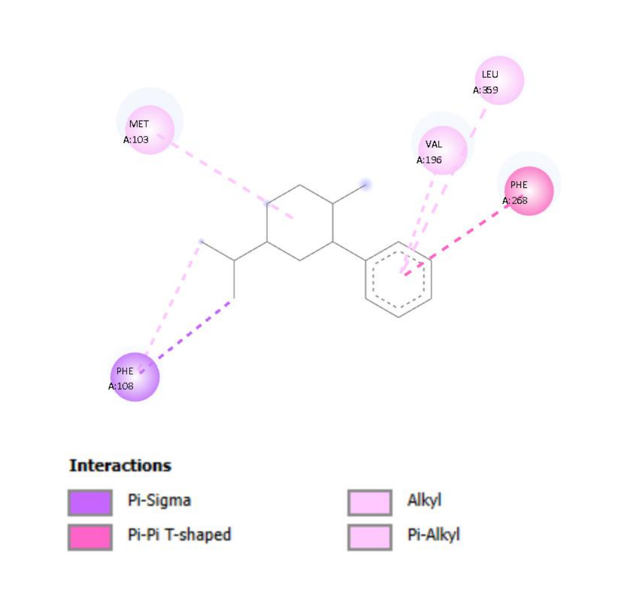
</td></tr>
<tr><td>Binding energy: -8.5</td><td>Binding energy: -8.2</td></tr>
</table>

| 9a (II) | ent-9a (II) |
|---|---|
| 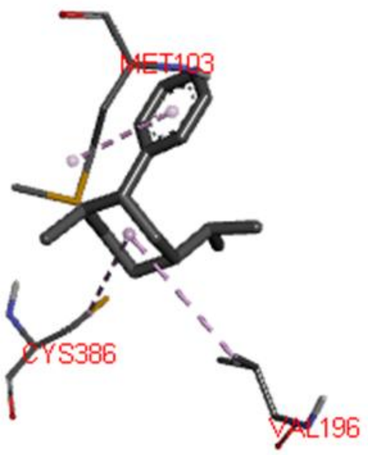  | 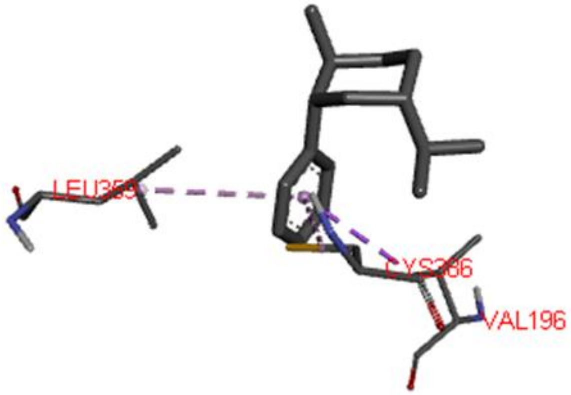  |
| 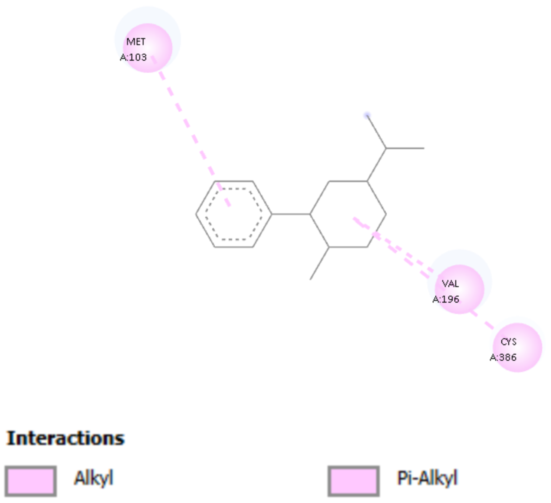  | 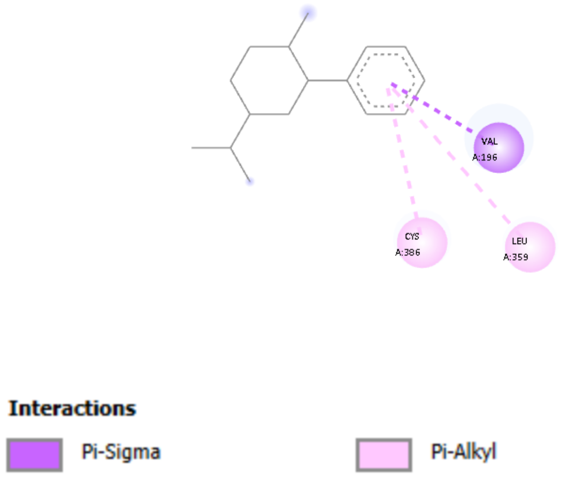  |
| **Binding energy: -7.3** | **Binding energy: -7.2** |

<table>
<tr><th>9b (I)</th><th>ent-9b (I)</th></tr>
<tr><td>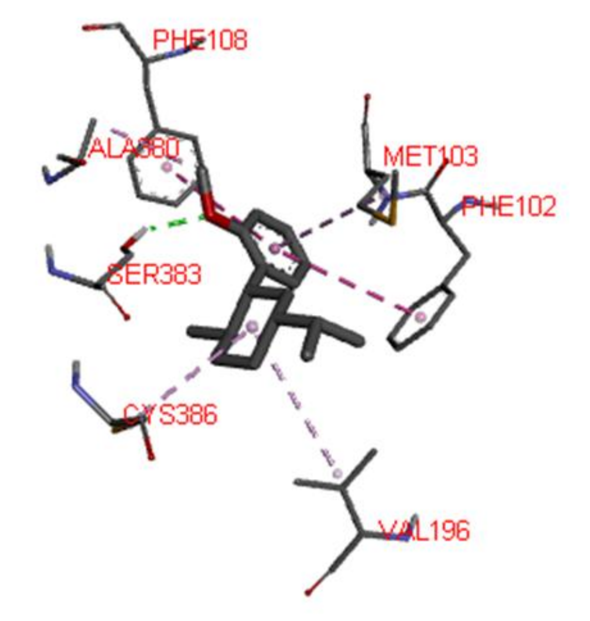
</td><td>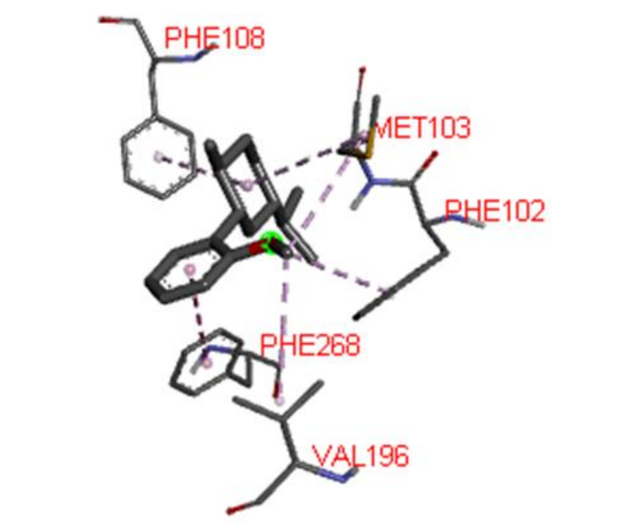
</td></tr>
<tr><td>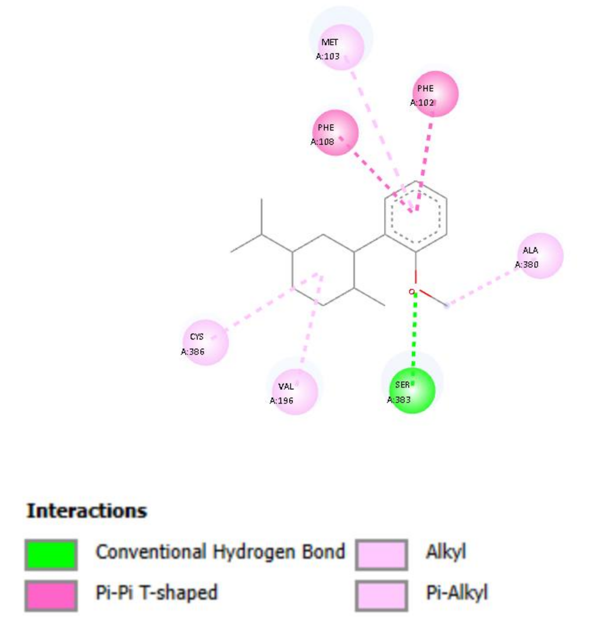
</td><td>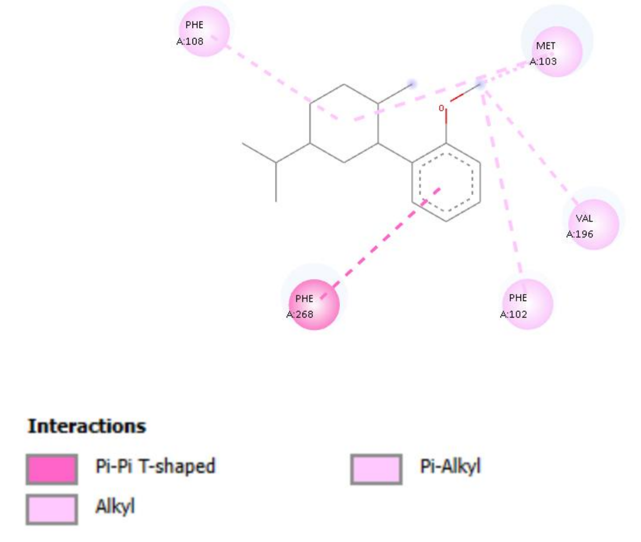
</td></tr>
<tr><td>Binding energy: -8.2</td><td>Binding energy: -8.0</td></tr>
</table>

| 9b (II) | ent-9b (II) |
| --- | --- |
| 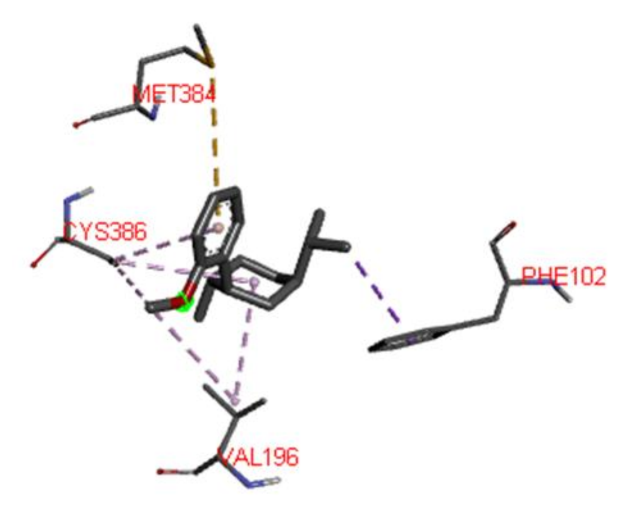 | 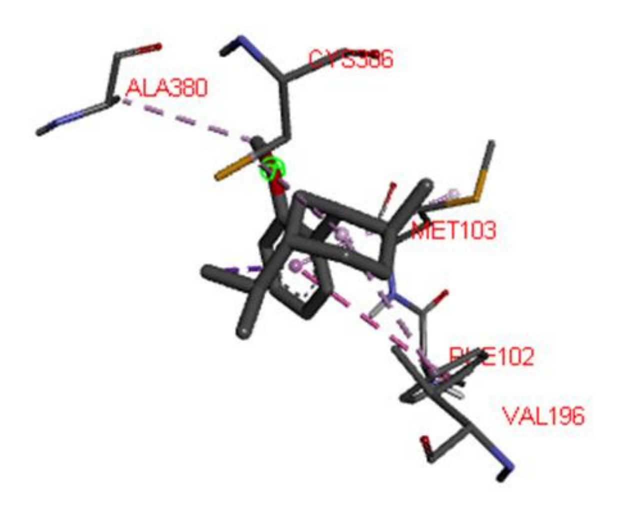 |
| 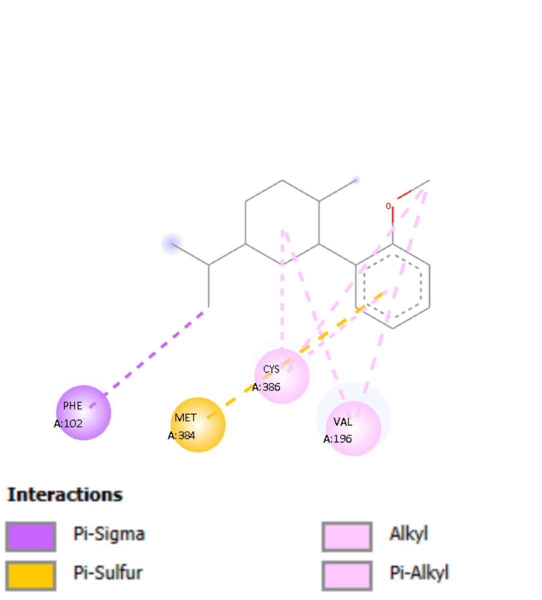 | 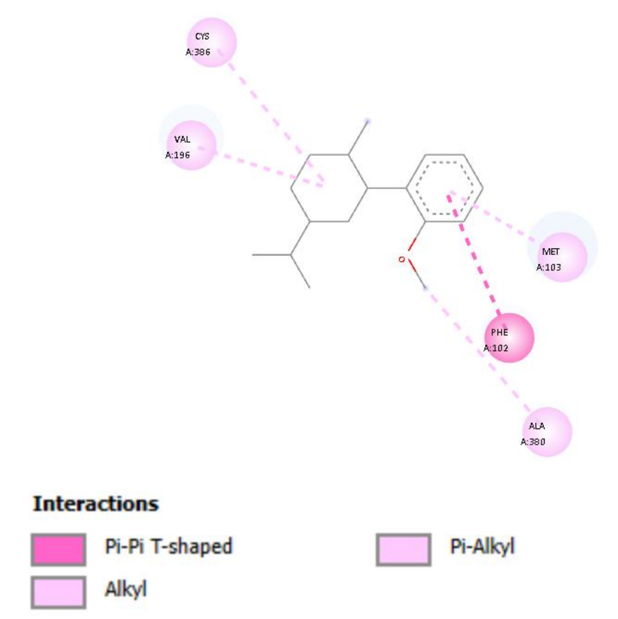 |
| **Binding energy: -7.0** | **Binding energy: -7.2** |

| 9c (I) | ent-9c (I) |
|---|---|
| 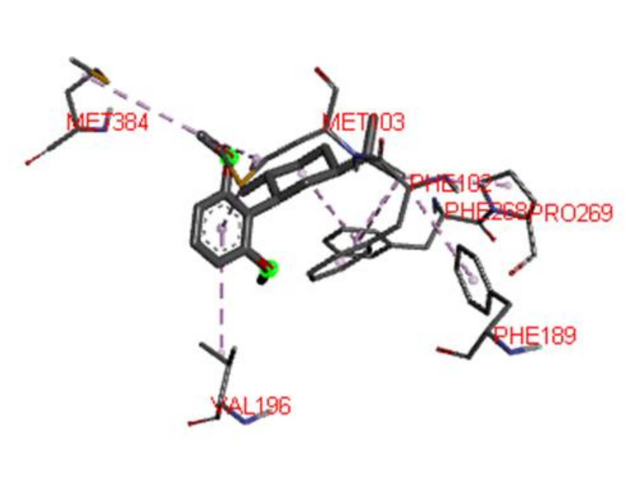 | 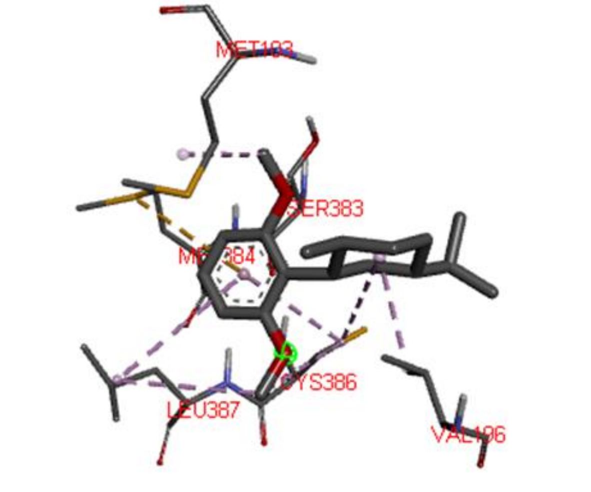 |
| 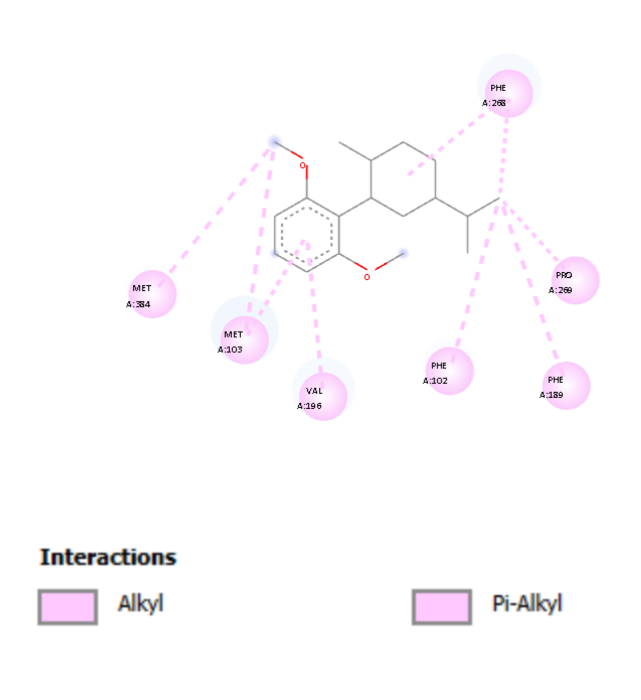 | 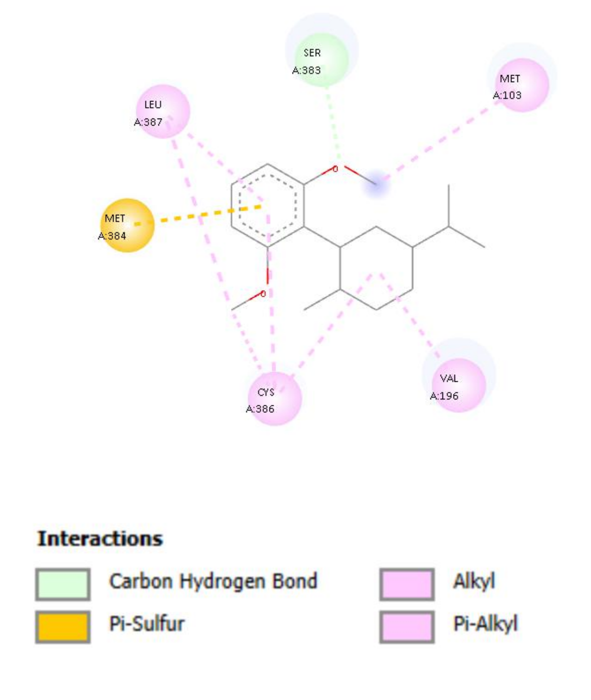 |
| **Binding energy: -7.5** | **Binding energy: -7.7** |

<table>
<tr><th>9c (II)</th><th>ent-9c (II)</th></tr>
<tr><td>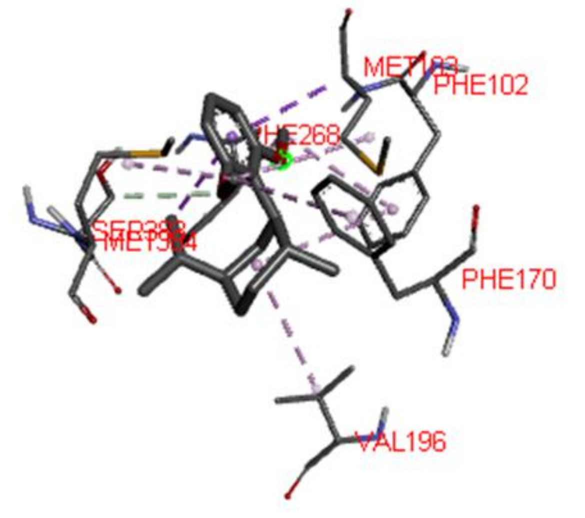
</td><td>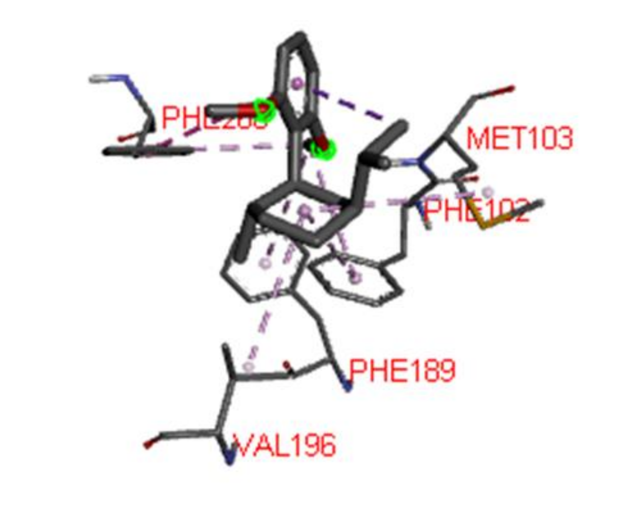
</td></tr>
<tr><td>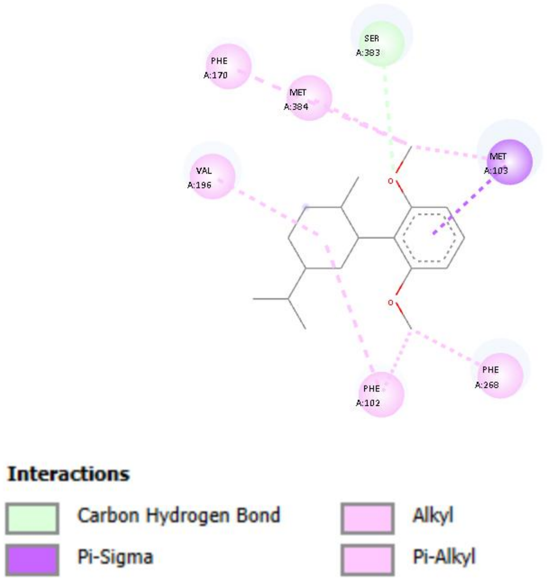
</td><td>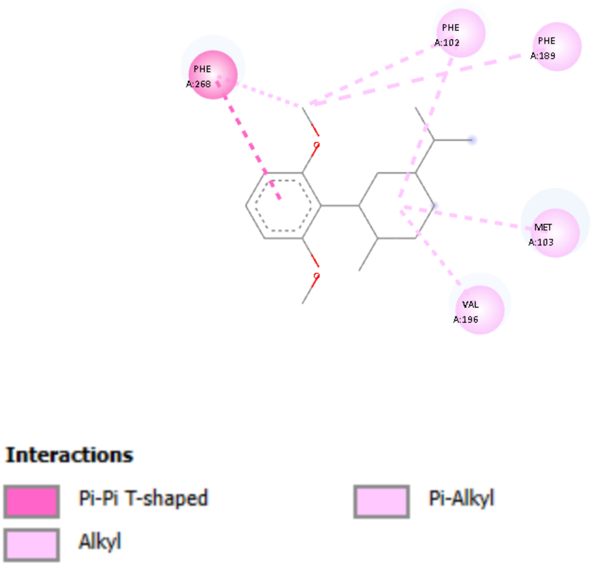
</td></tr>
<tr><td>Binding energy: -6.2</td><td>Binding energy: -6.5</td></tr>
</table>

<table>
<tr><th>9d (I)</th><th>ent-9d (I)</th></tr>
<tr><td>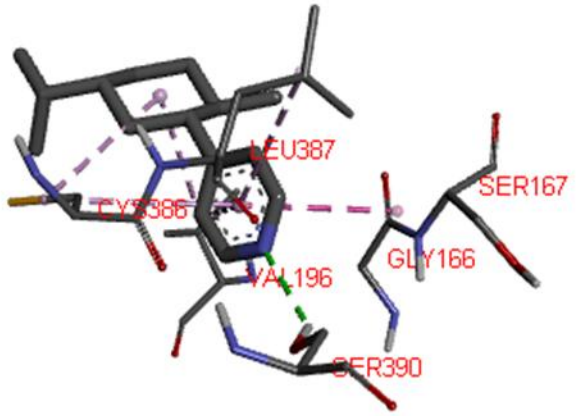
</td><td>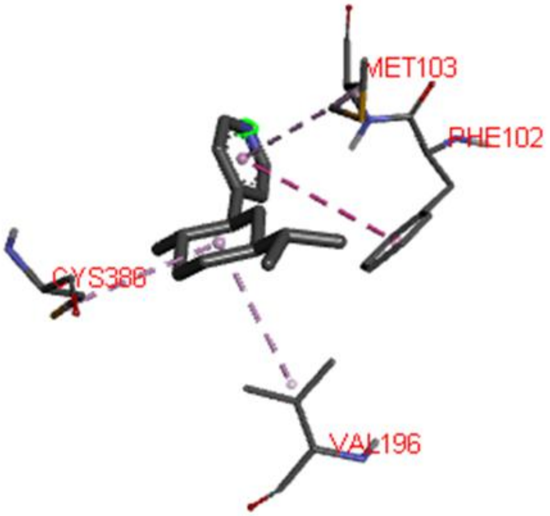
</td></tr>
<tr><td>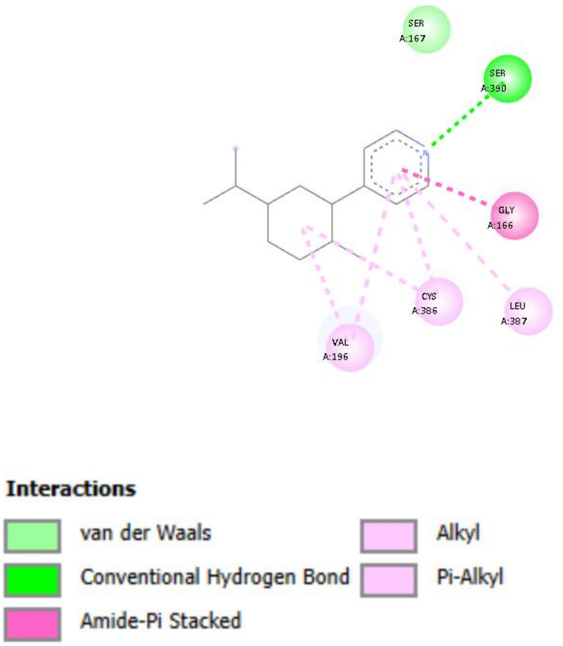
</td><td>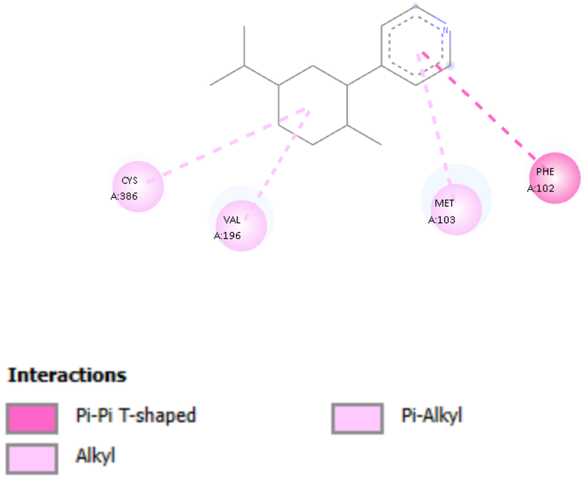
</td></tr>
<tr><td>Binding energy: -8.5</td><td>Binding energy: -7.6</td></tr>
</table>

<table>
<tr><th>9d (II)</th><th>ent-9d (II)</th></tr>
<tr><td>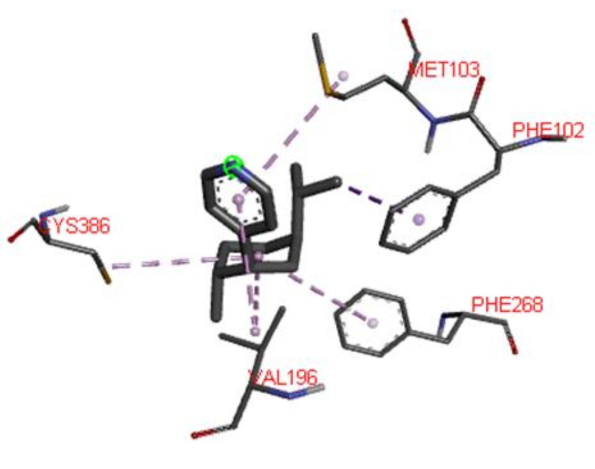
</td><td>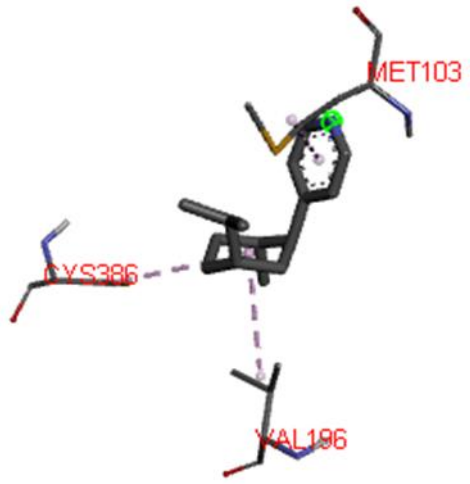
</td></tr>
<tr><td>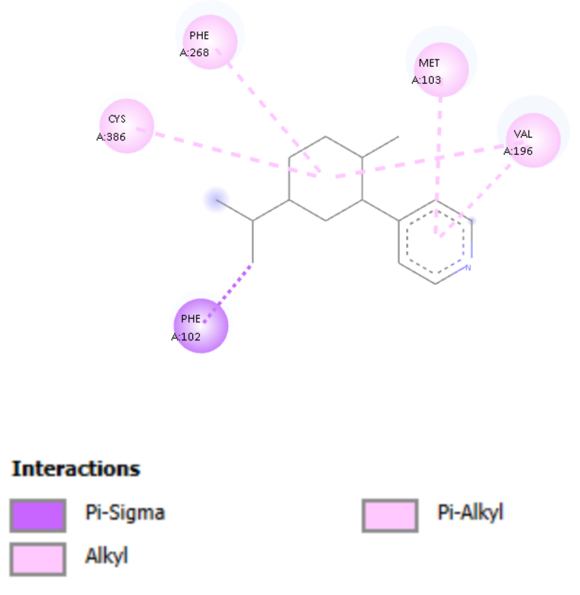
</td><td>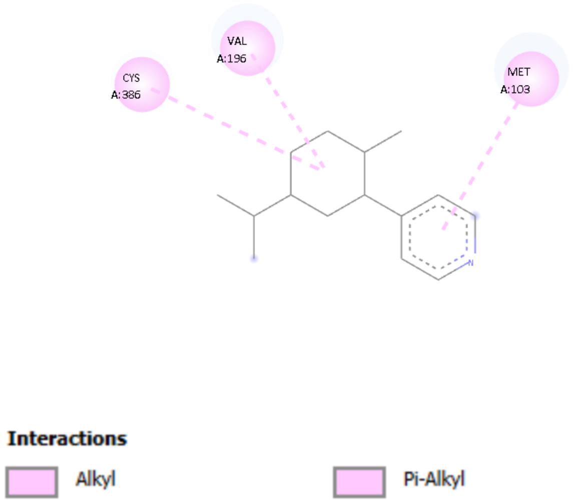
</td></tr>
<tr><td>Binding energy: -6.9</td><td>Binding energy: -6.9</td></tr>
</table>

<table>
<tr><th>9e (I)</th><th>ent-9e (I)</th></tr>
<tr><td>
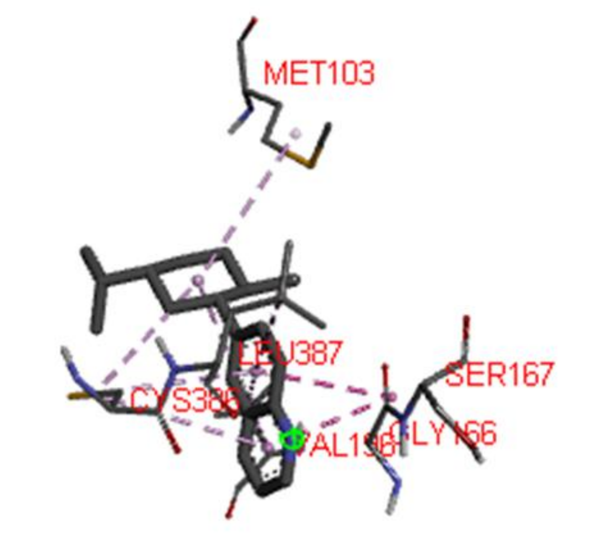

</td><td>
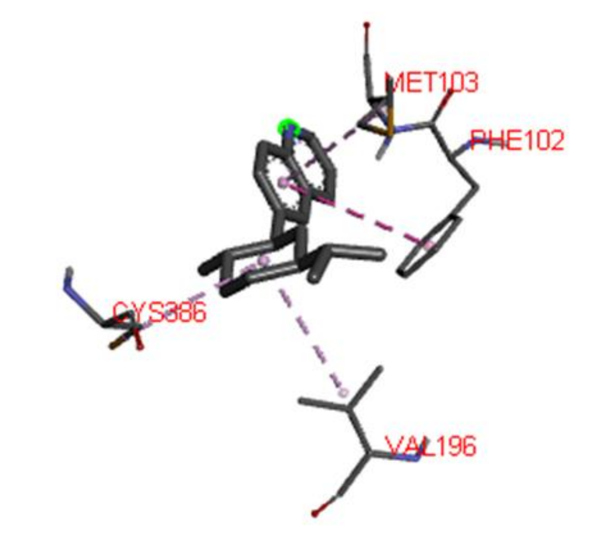

</td></tr>
<tr><td>
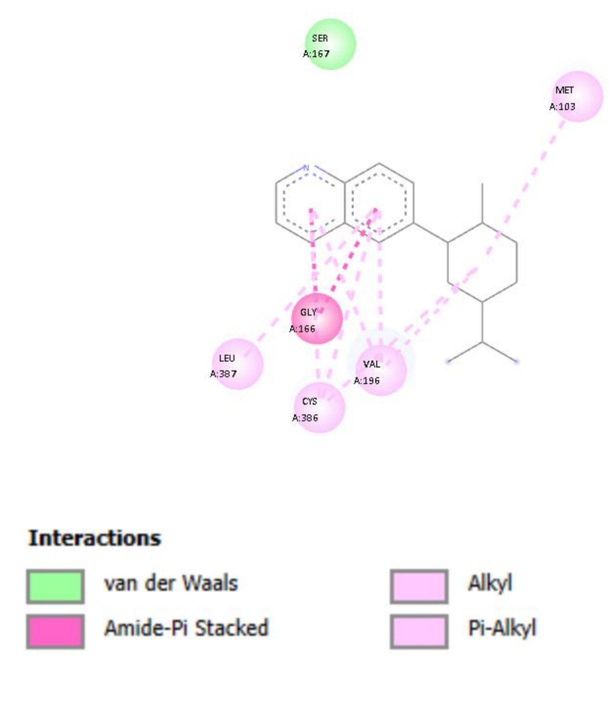

</td><td>
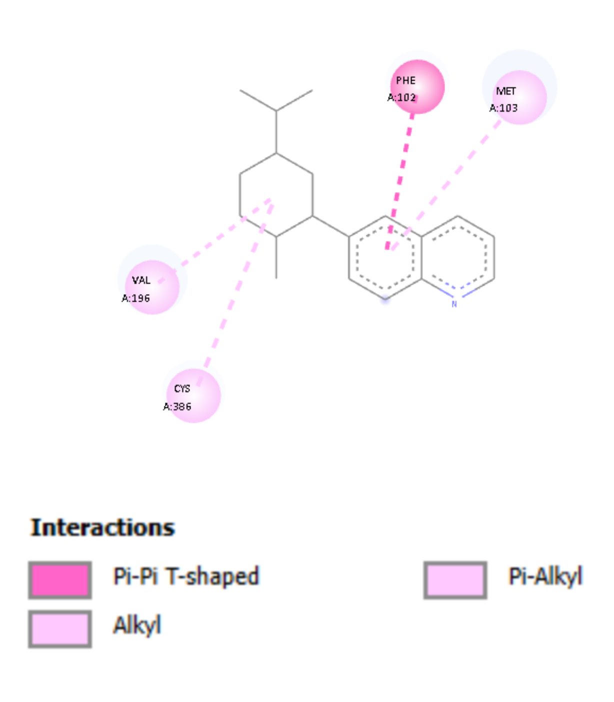

</td></tr>
<tr><td>Binding energy: -9.8</td><td>Binding energy: -10.1</td></tr>
</table>

<table>
<tr><th>9e (II)</th><th>ent-9e (II)</th></tr>
<tr><td>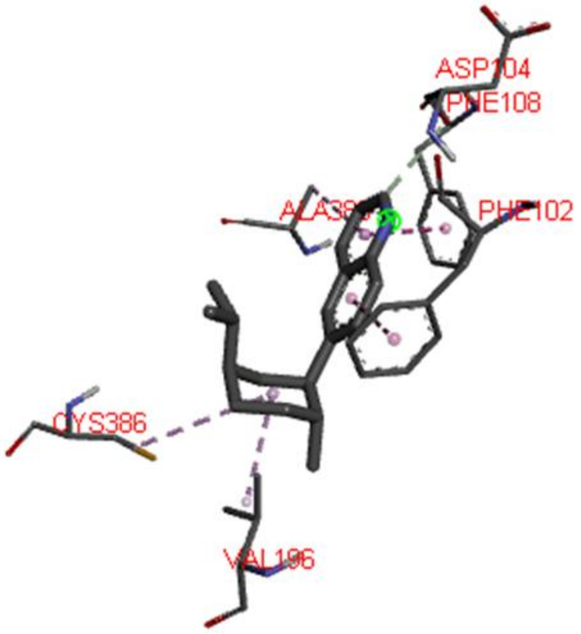
</td><td>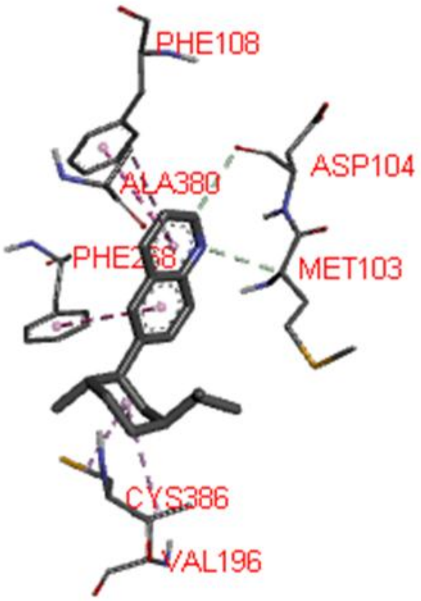
</td></tr>
<tr><td>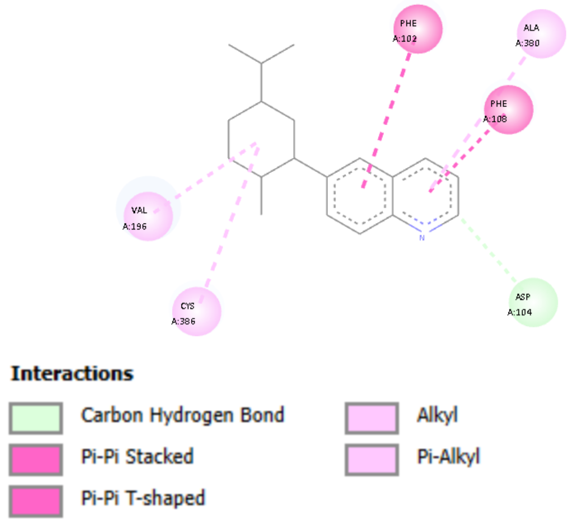
</td><td>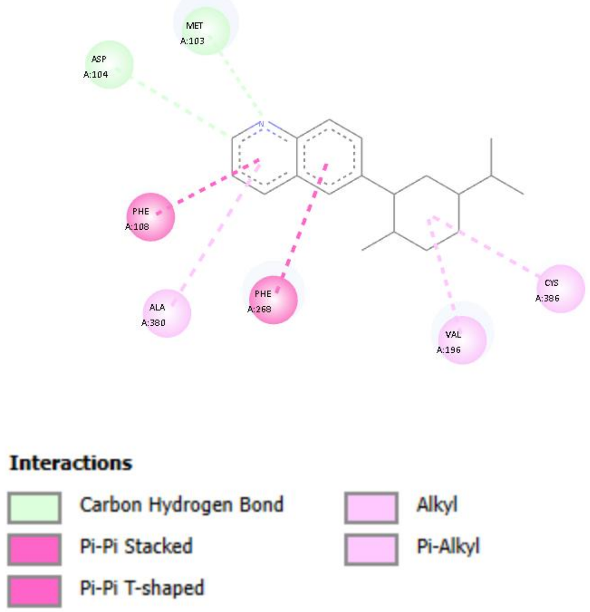
</td></tr>
<tr><td>Binding energy: -8.6</td><td>Binding energy: -9.4</td></tr>
</table>

<table>
<tr><th>9f (I)</th><th>ent-9f (I)</th></tr>
<tr><td>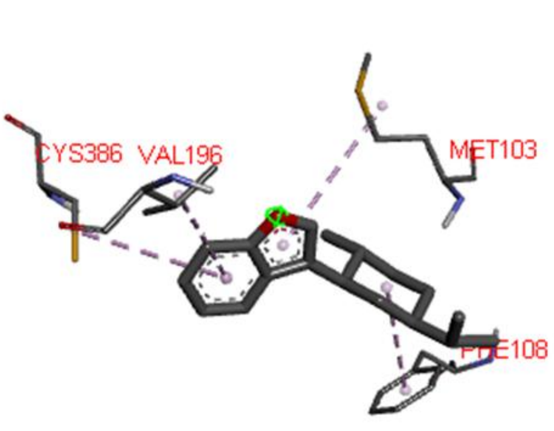
</td><td>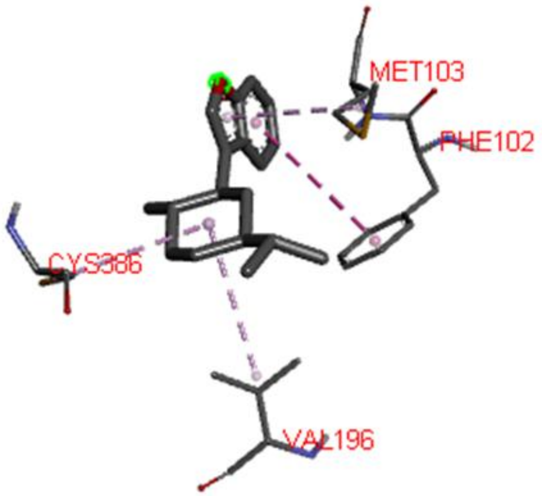
</td></tr>
<tr><td>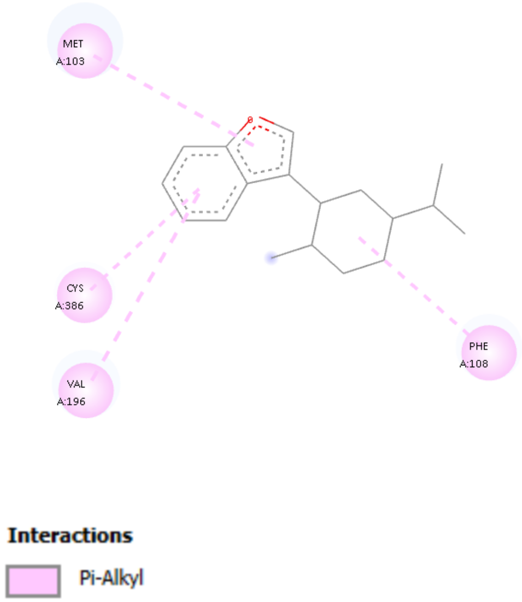
</td><td>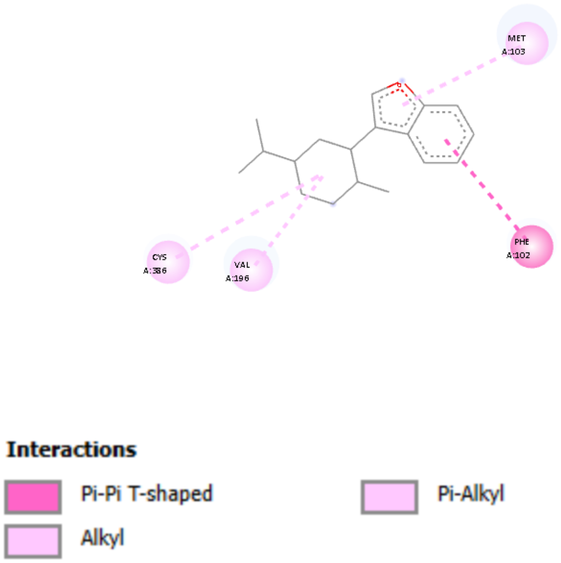
</td></tr>
<tr><td>Binding energy: -9.1</td><td>Binding energy: -9.2</td></tr>
</table>

| 9f (II) | ent-9f (II) |
|---|---|
| 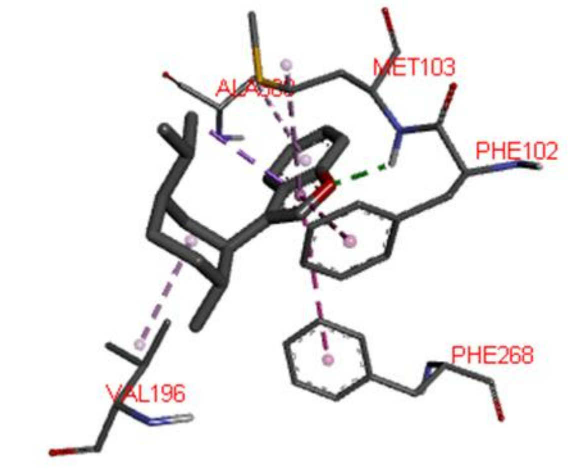  | 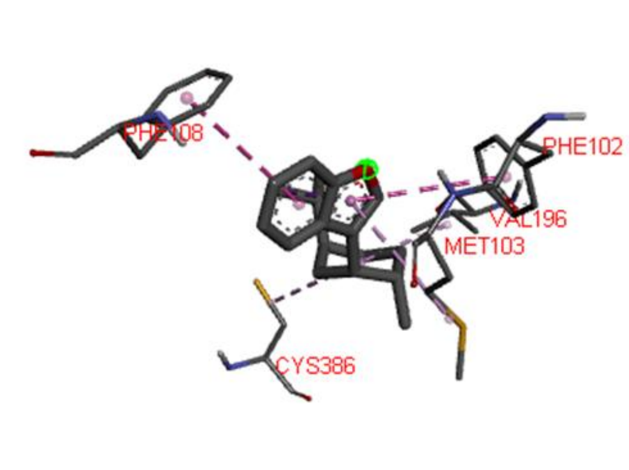  |
| 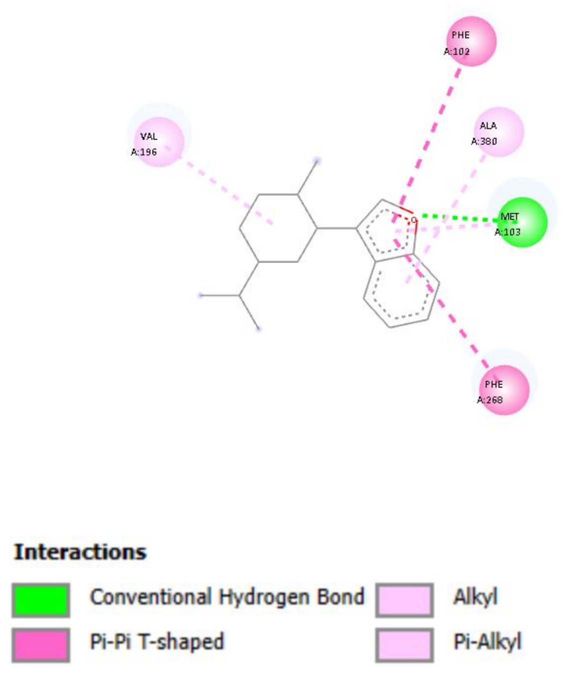  | 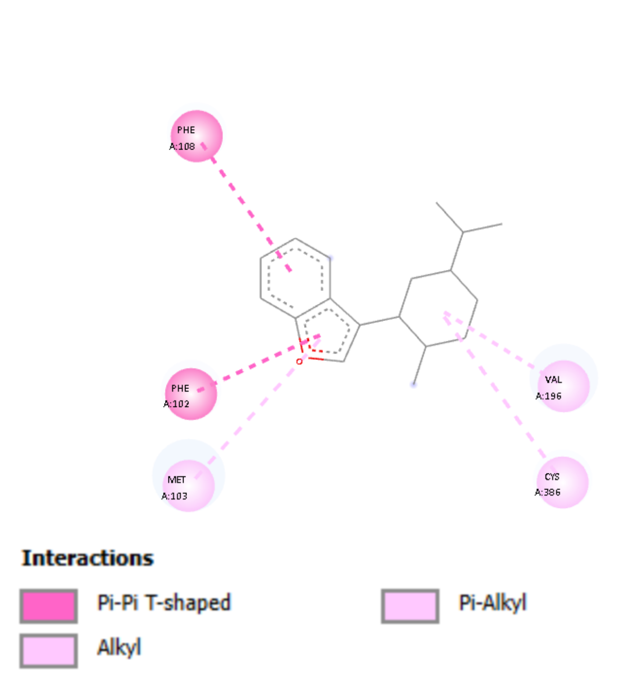  |
| **Binding energy: -8.4** | **Binding energy: -7.9** |

• *Ketone Precursors with CB2 Receptor*

| **(-)-1** | **(+)-1** |
|---|---|
| 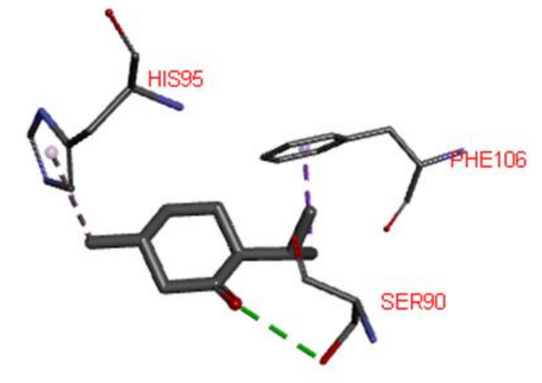 | 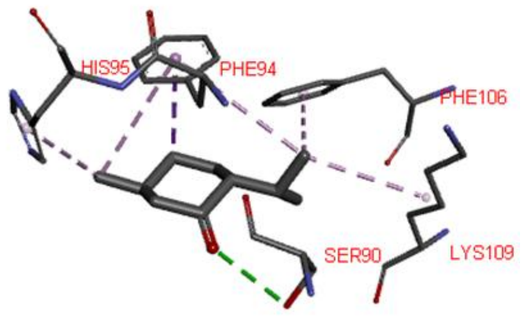 |
| 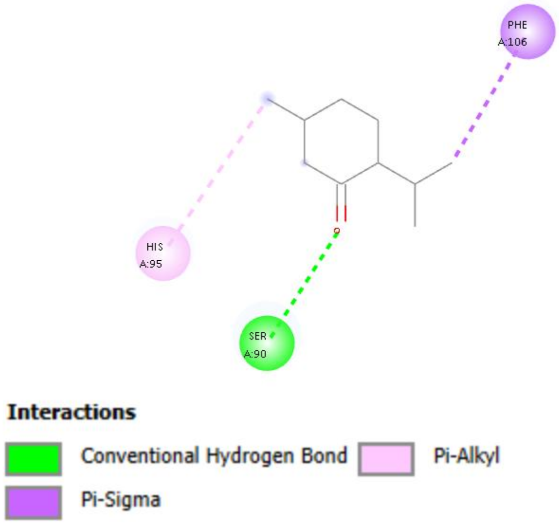 | 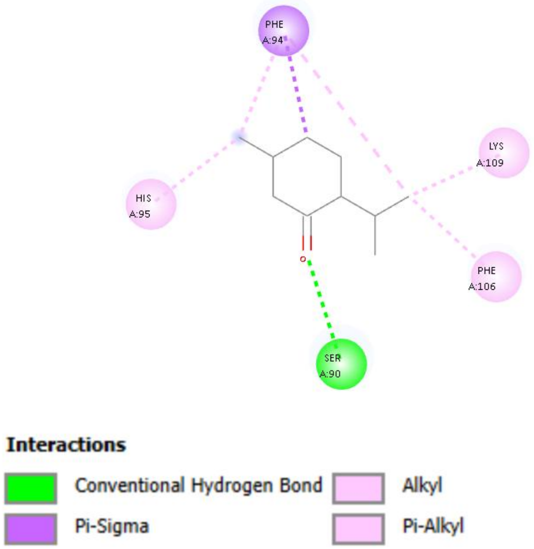 |
| **Binding energy: -6.6** | **Binding energy: -7.0** |

| (+)-2 | (-)-2 |
|---|---|
| 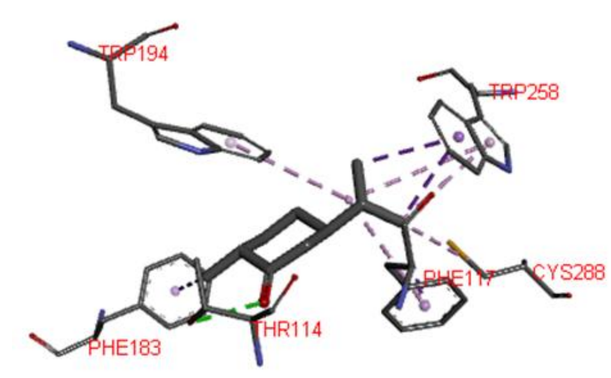 | 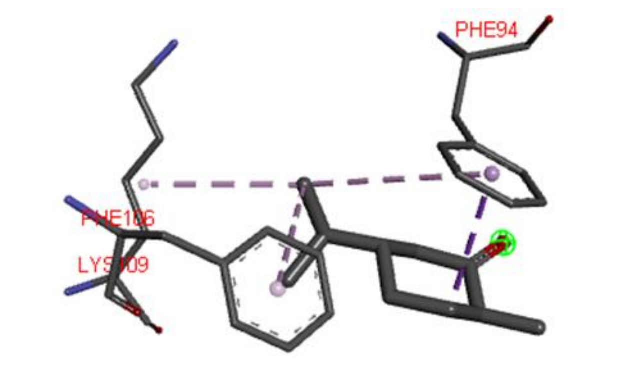 |
| 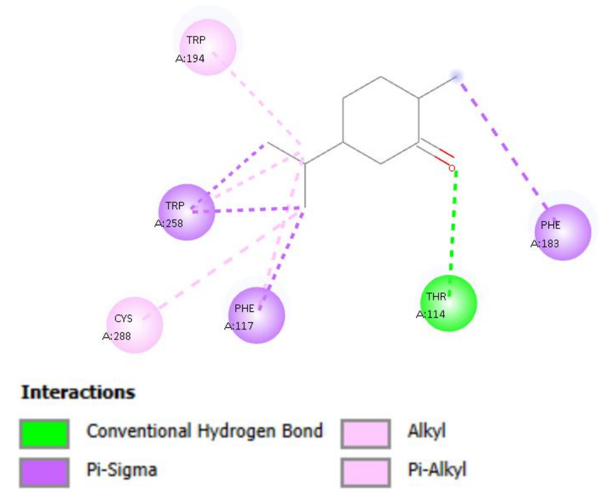 | 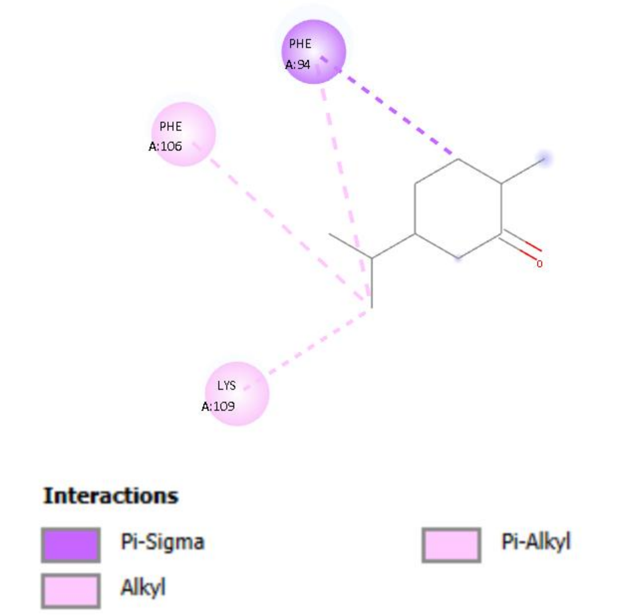 |
| **Binding energy: -6.7** | **Binding energy: -6.8** |

• *Synthesized Ligands of Series **6**/ent-**6** with CB2 Receptor*

| **6a (II)** | **ent-6a (II)** |
|---|---|
| 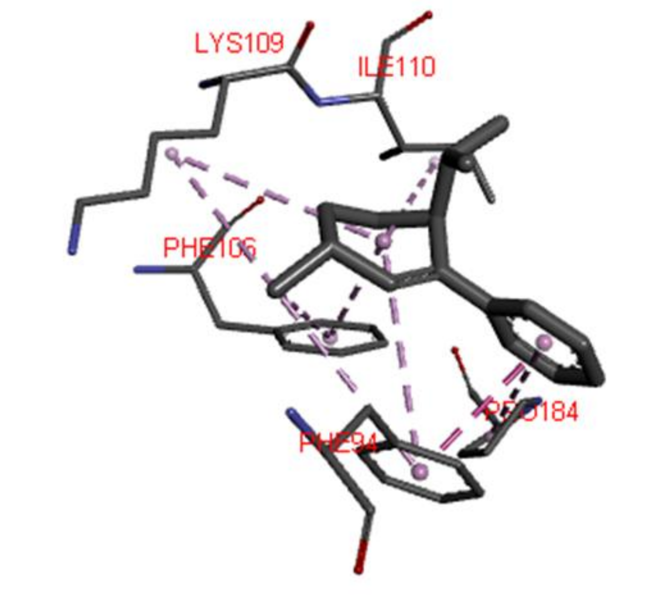  | 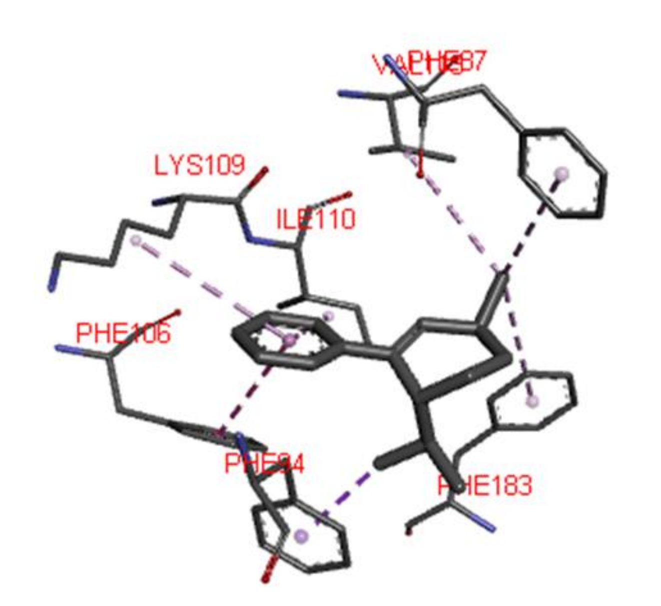  |
| 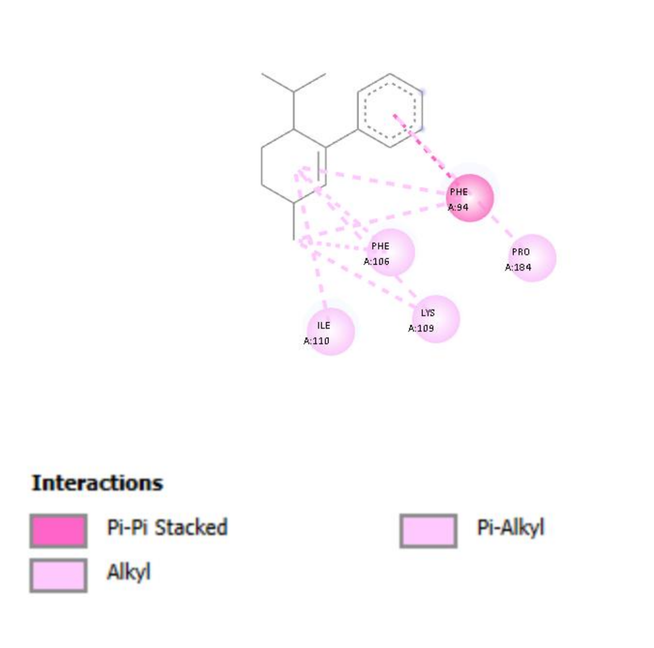  | 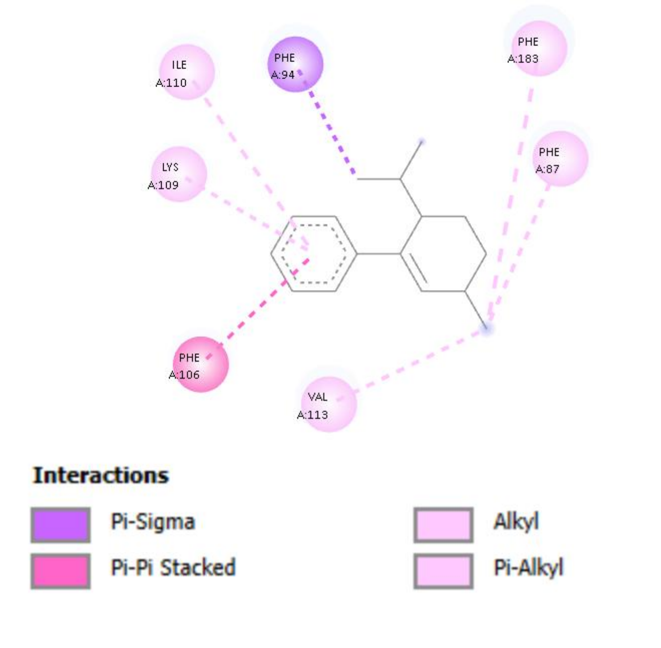  |
| **Binding energy: -9.4** | **Binding energy: -8.6** |

| 6b (I) | ent-6b (I) |
| --- | --- |
| 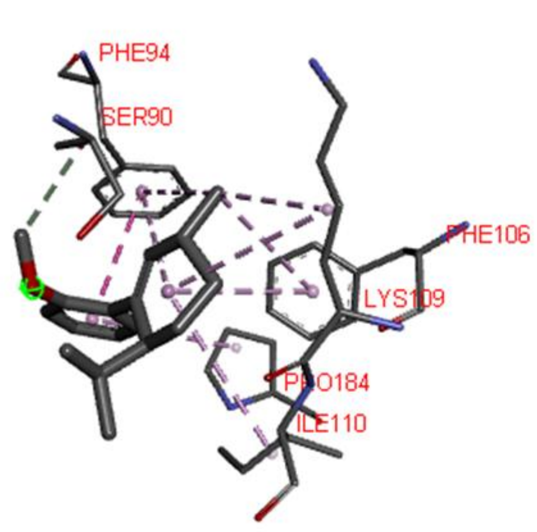 | 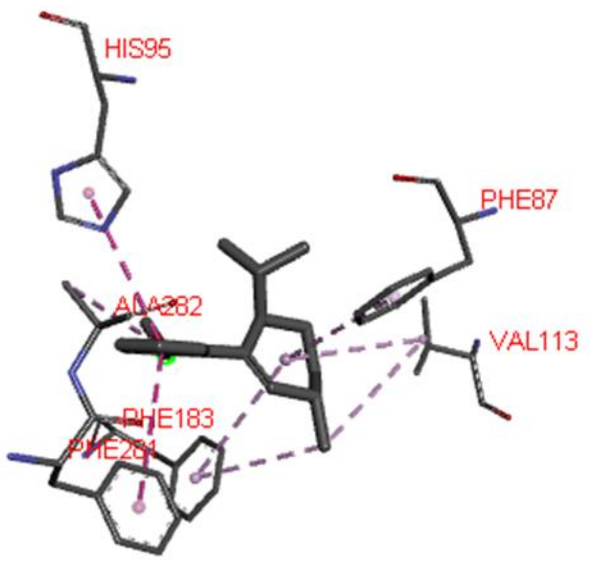 |
| 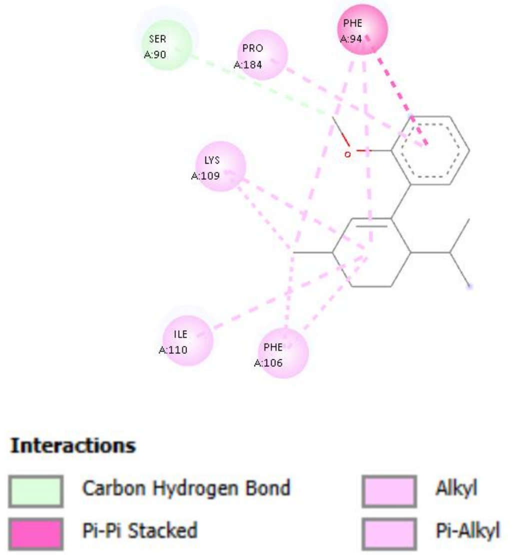 | 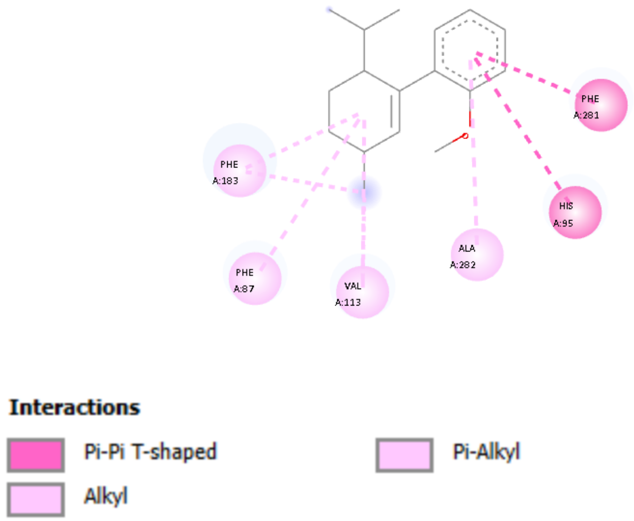 |
| **Binding energy: -9.3** | **Binding energy: -8.3** |

<table>
<tr><th>6b (II)</th><th>ent-6b (II)</th></tr>
<tr><td>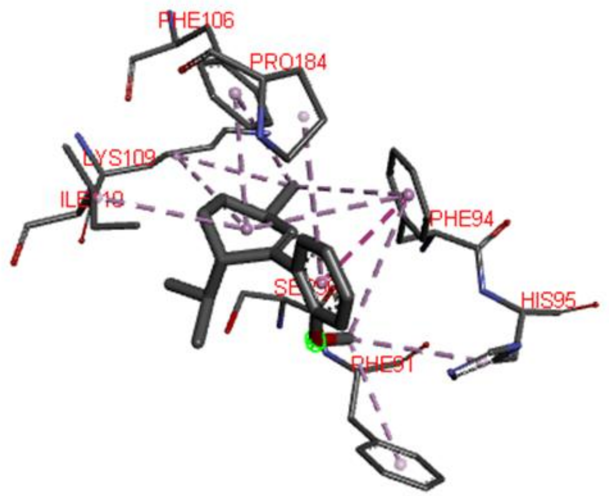
</td><td>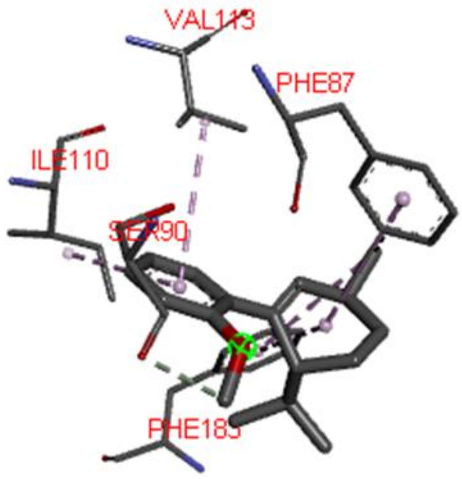
</td></tr>
<tr><td>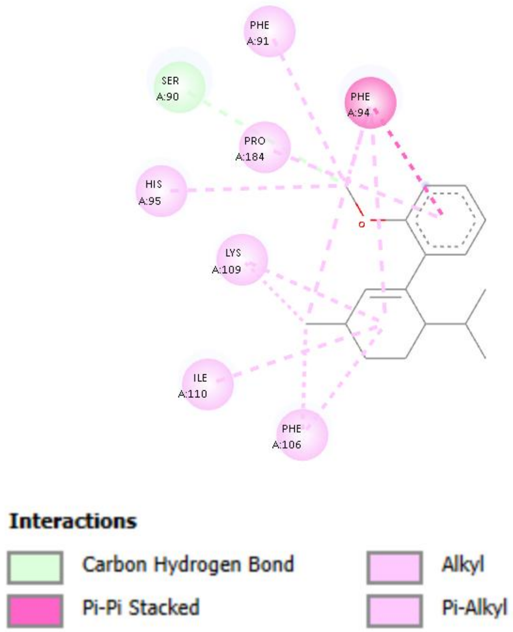
</td><td>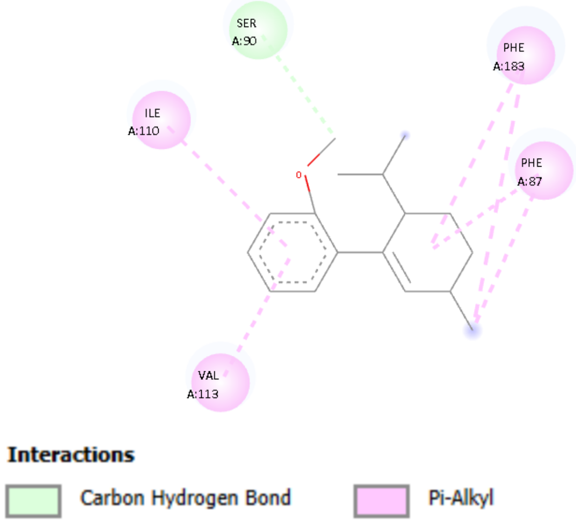
</td></tr>
<tr><td>Binding energy: -8.5</td><td>Binding energy: -8.3</td></tr>
</table>

<table>
<tr><th>6c (I)</th><th>ent-6c (I)</th></tr>
<tr><td>
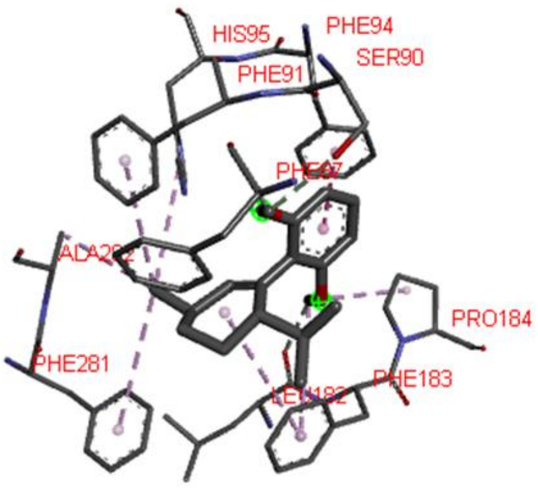

</td><td>
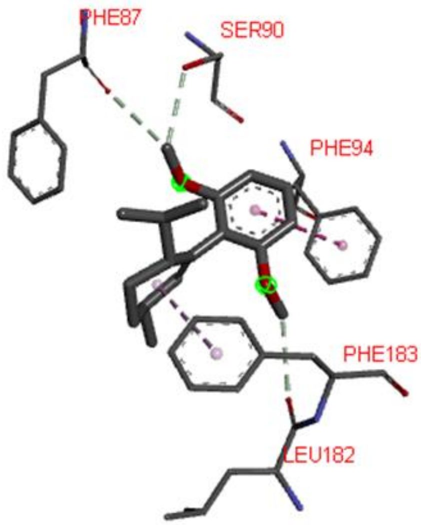

</td></tr>
<tr><td>
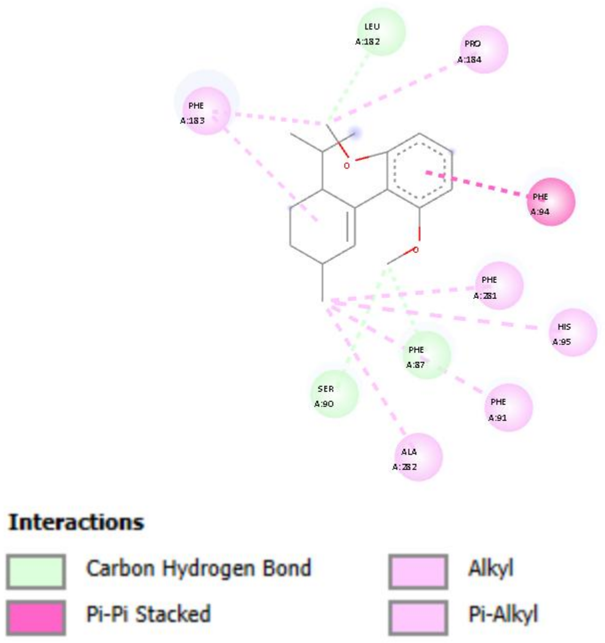

</td><td>
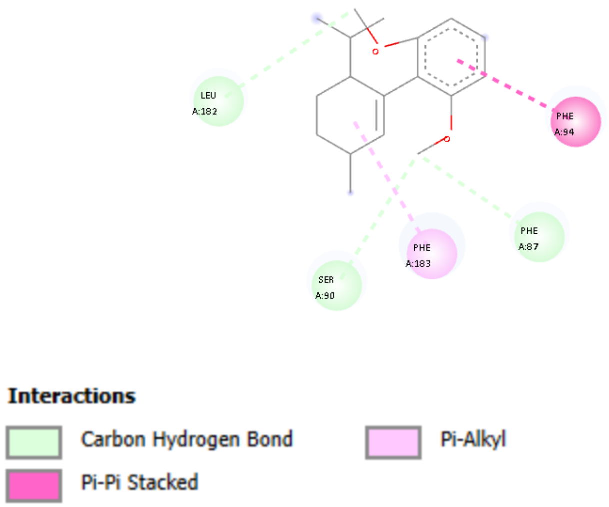

</td></tr>
<tr><td>Binding energy: -7.6</td><td>Binding energy: -7.7</td></tr>
</table>

<table>
<tr><th>6d (I)</th><th>ent-6d (I)</th></tr>
<tr><td>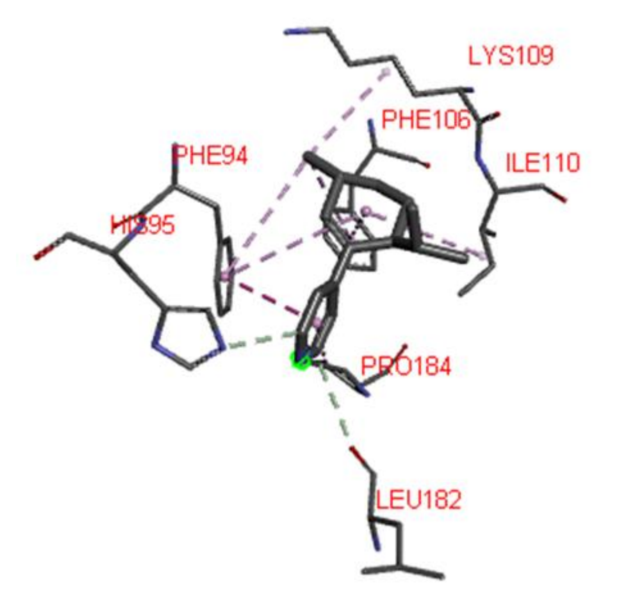
</td><td>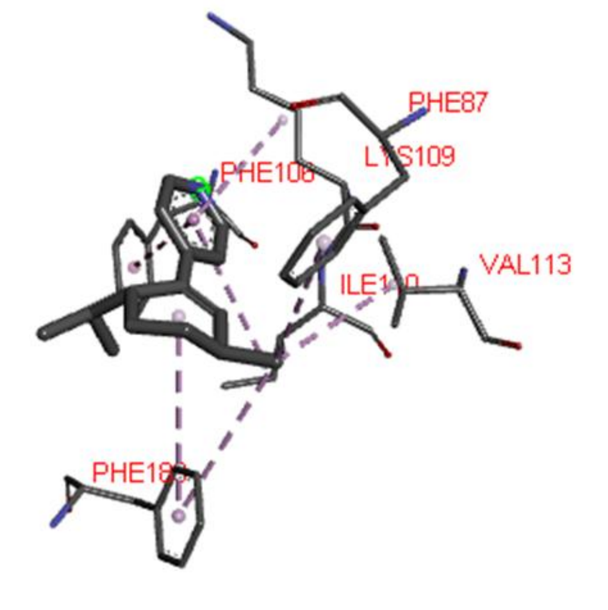
</td></tr>
<tr><td>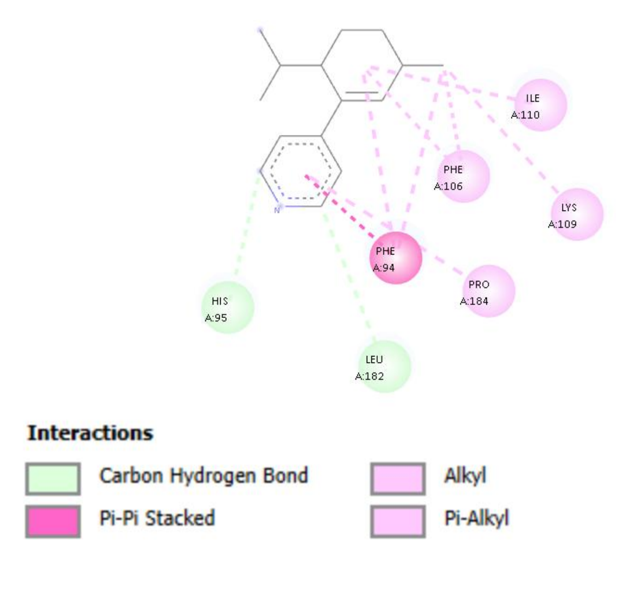
</td><td>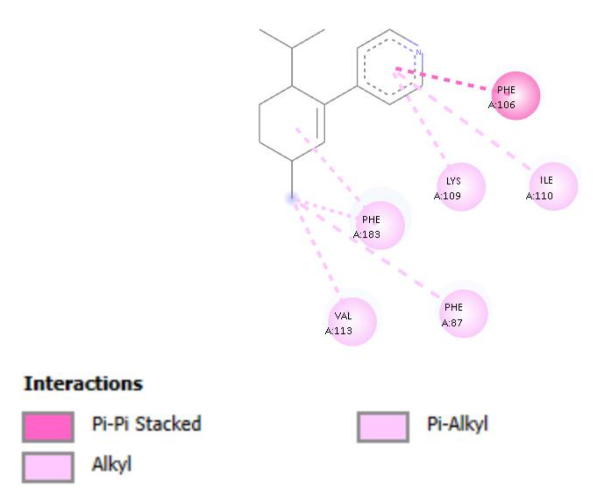
</td></tr>
<tr><td>Binding energy: -9.0</td><td>Binding energy: -8.2</td></tr>
</table>

<table>
<tr><th>6d (II)</th><th>ent-6d (II)</th></tr>
<tr><td>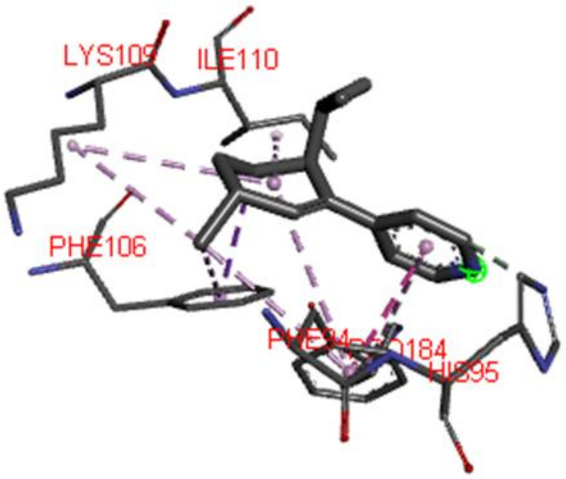
</td><td>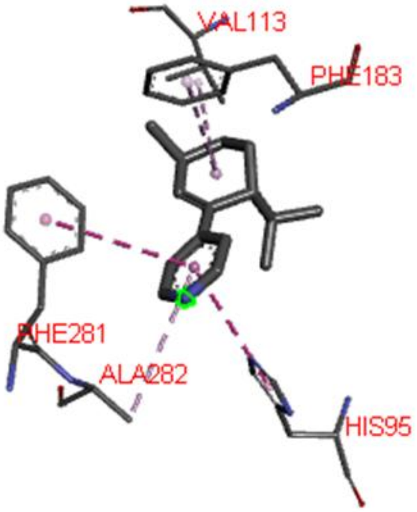
</td></tr>
<tr><td>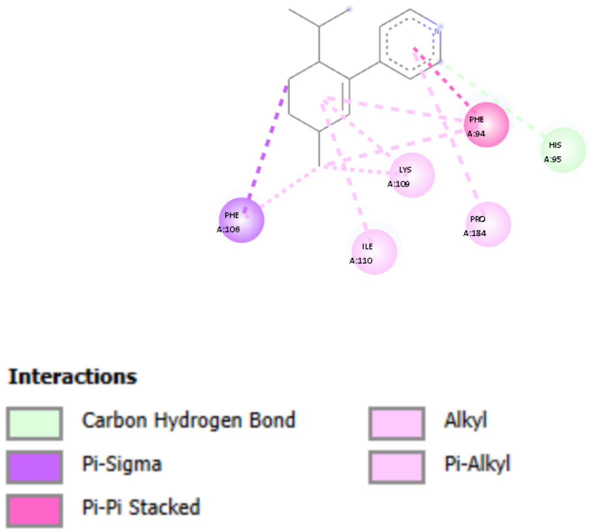
</td><td>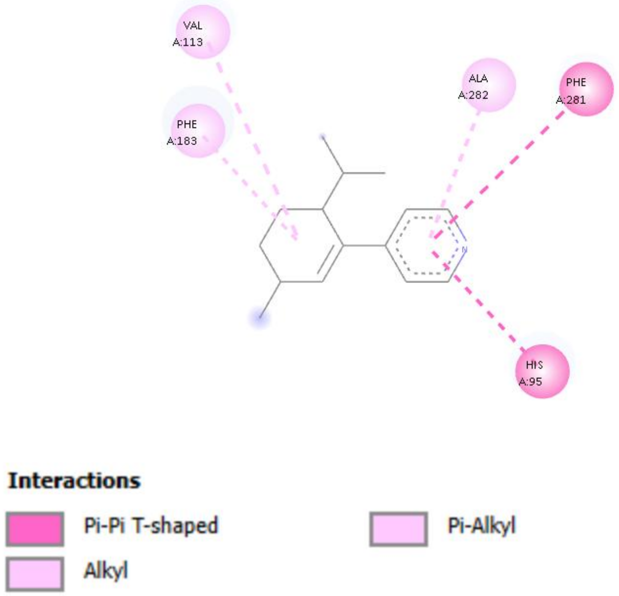
</td></tr>
<tr><td>Binding energy: -9.2</td><td>Binding energy: -8.0</td></tr>
</table>

<table>
<tr><th>6e (II)</th><th>ent-6e (II)</th></tr>
<tr><td>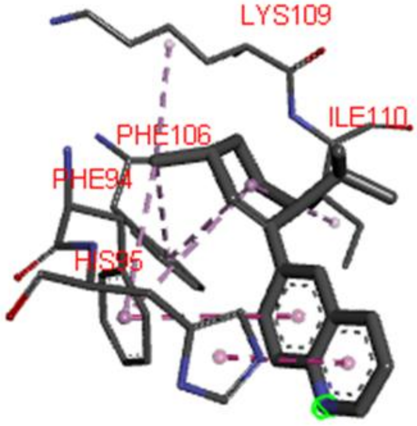
</td><td>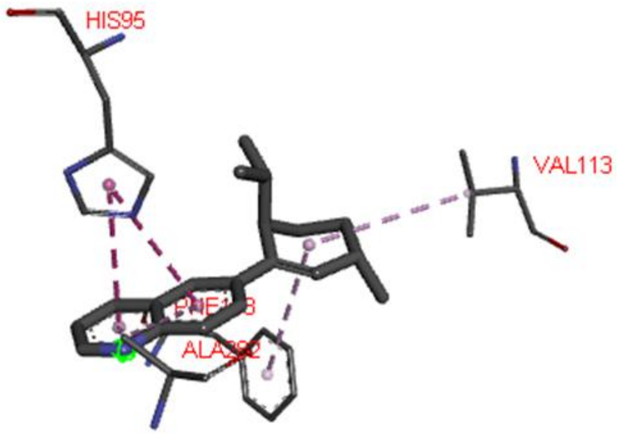
</td></tr>
<tr><td>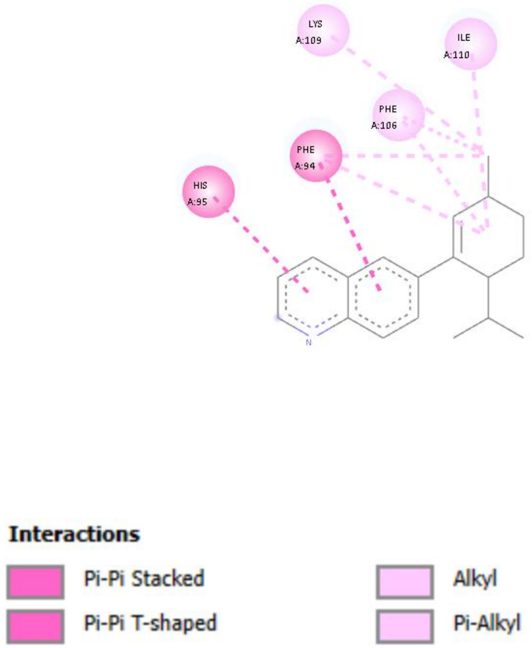
</td><td>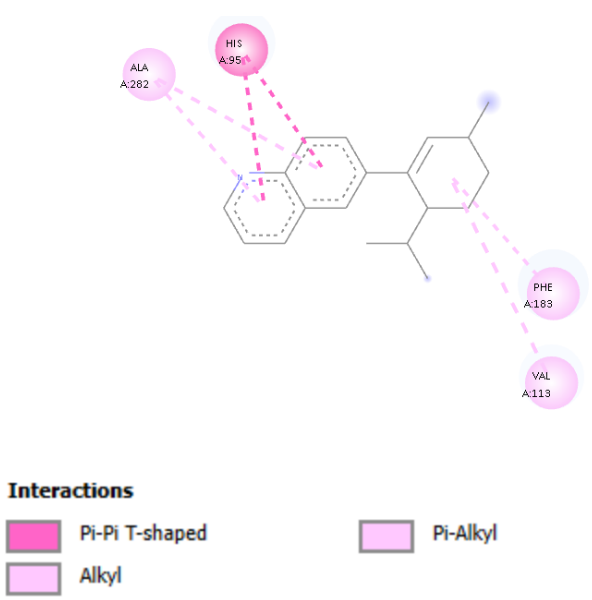
</td></tr>
<tr><td>Binding energy: -10.7</td><td>Binding energy: -9.8</td></tr>
</table>

<table>
<tr><th>6f (II)</th><th>ent-6f (II)</th></tr>
<tr><td>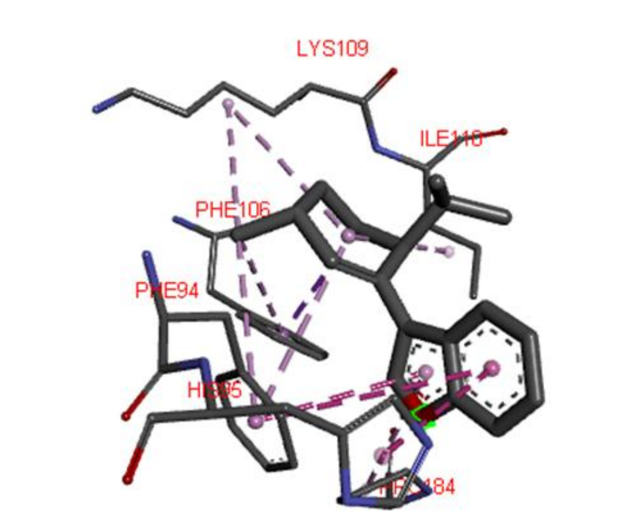
</td><td>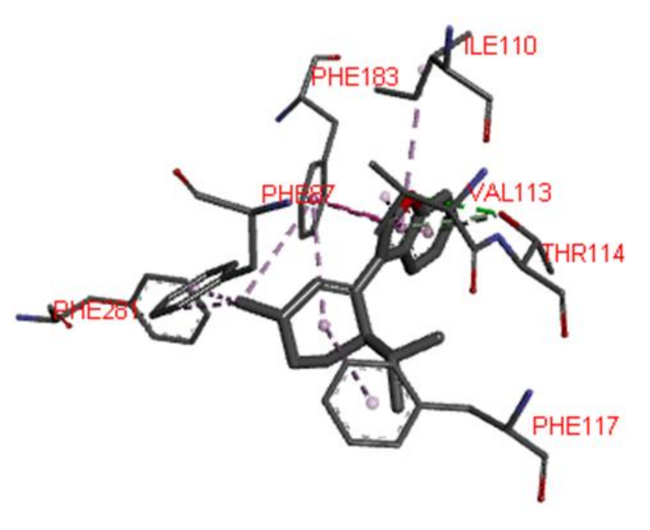
</td></tr>
<tr><td>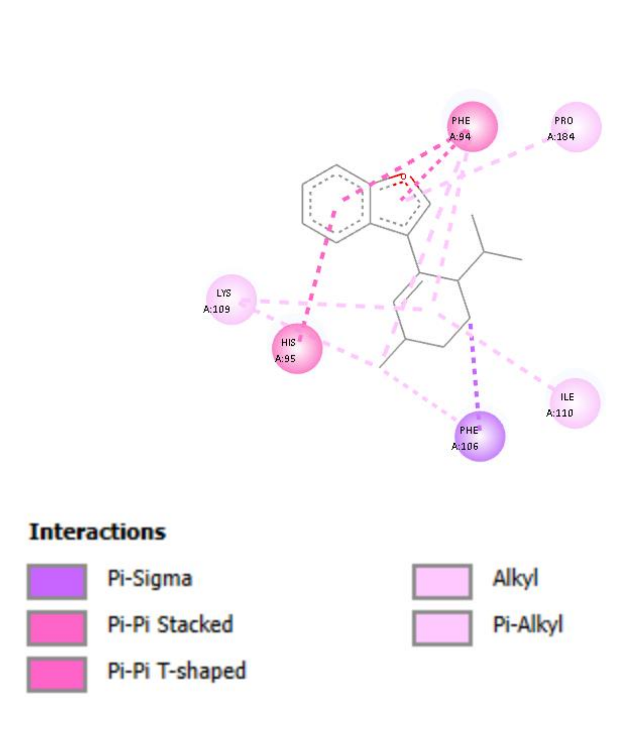
</td><td>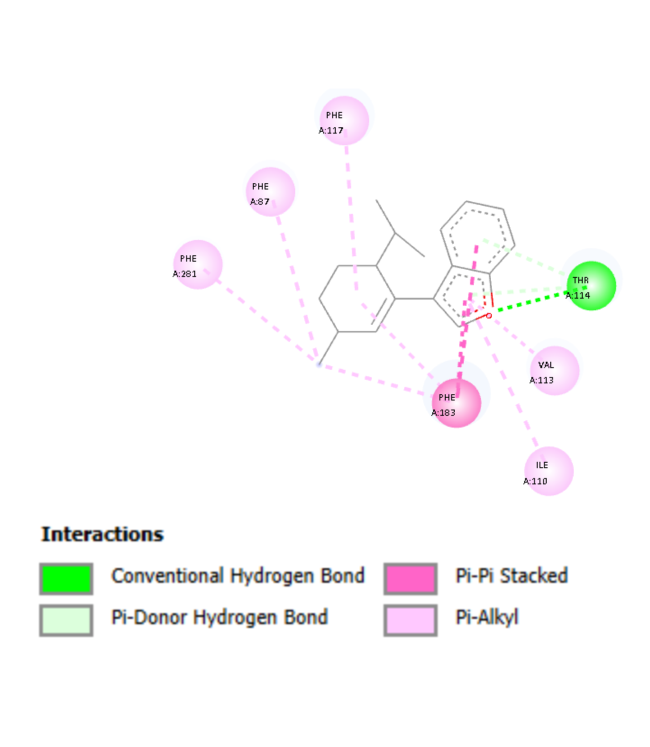
</td></tr>
<tr><td>Binding energy: -10.2</td><td>Binding energy: -9.5</td></tr>
</table>

• *Synthesized Ligands of Series **7**/ent-**7** with CB2 Receptor*

| **7a (I)** | **ent-7a (I)** |
|---|---|
| 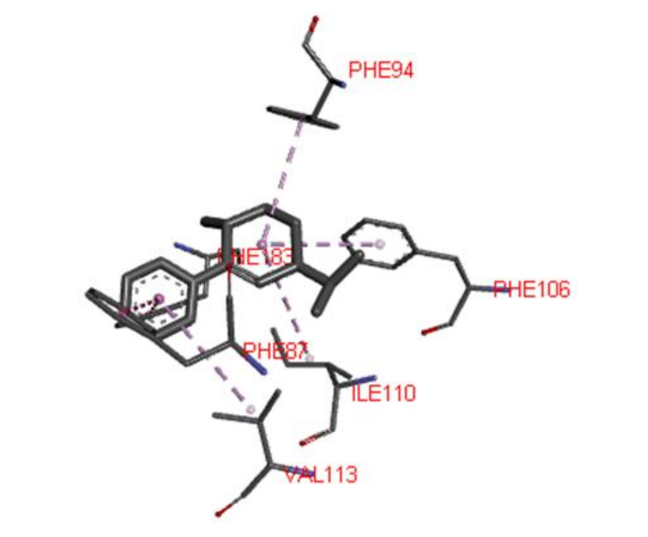 | 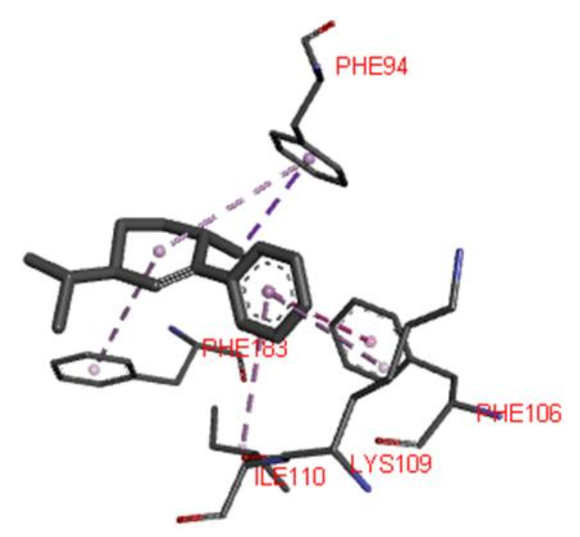 |
| 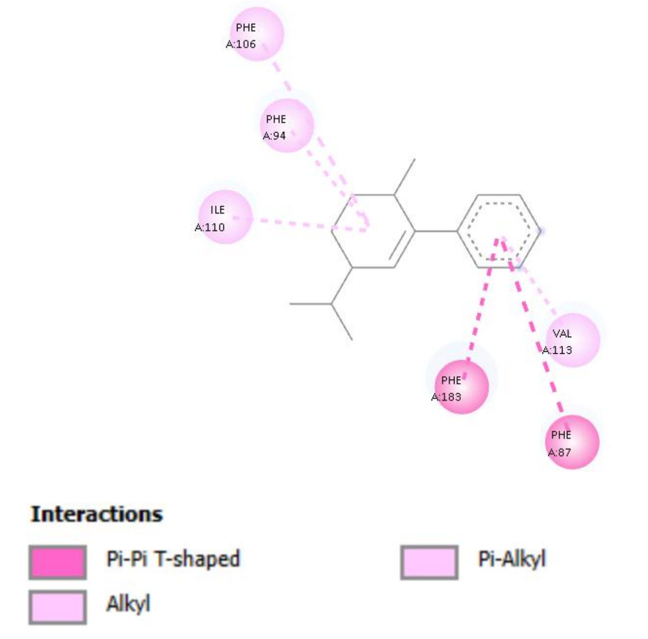 | 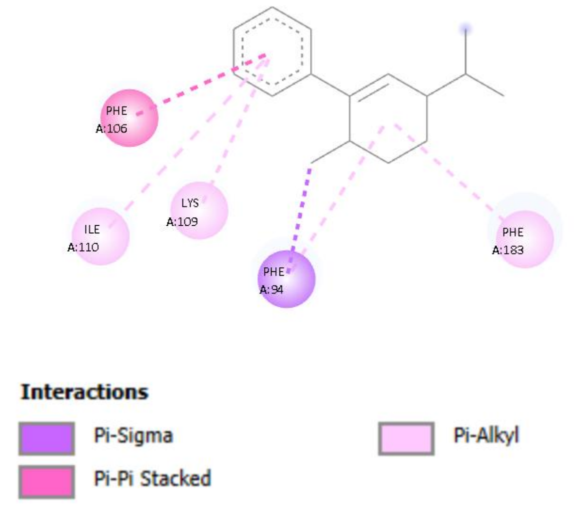 |
| **Binding energy: -9.5** | **Binding energy: -8.9** |

<table>
<tr><th>7a (II)</th><th>ent-7a (II)</th></tr>
<tr><td>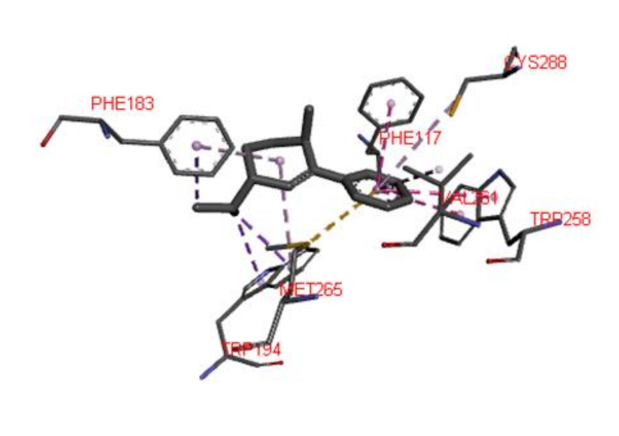
</td><td>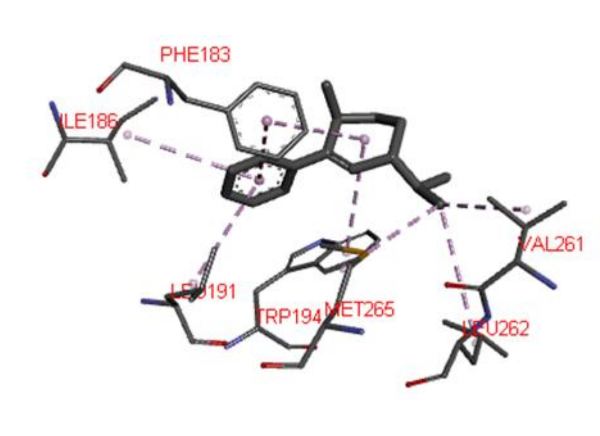
</td></tr>
<tr><td>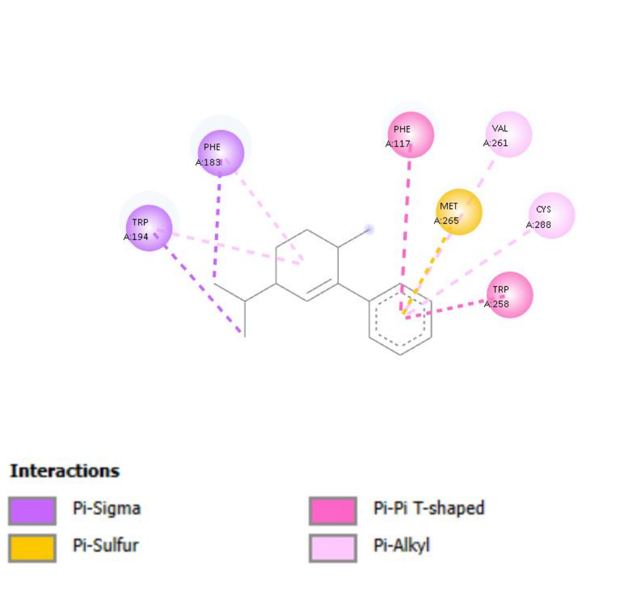
</td><td>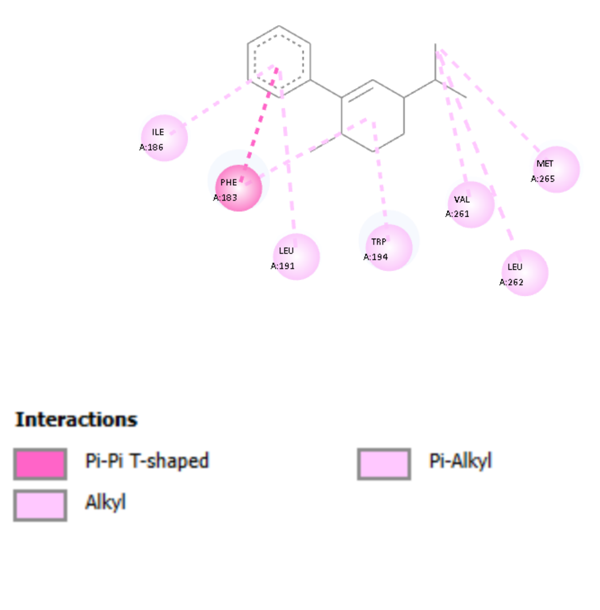
</td></tr>
<tr><td>Binding energy: -9.2</td><td>Binding energy: -9.2</td></tr>
</table>

<table>
<tr><th>7b (I)</th><th>ent-7b (I)</th></tr>
<tr><td>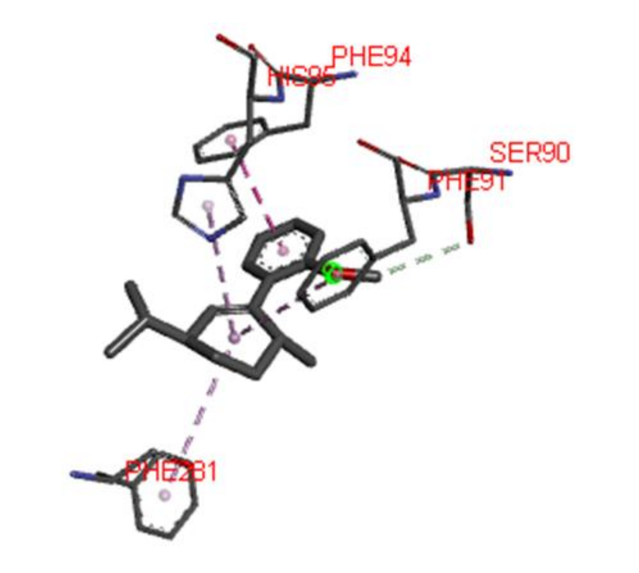
</td><td>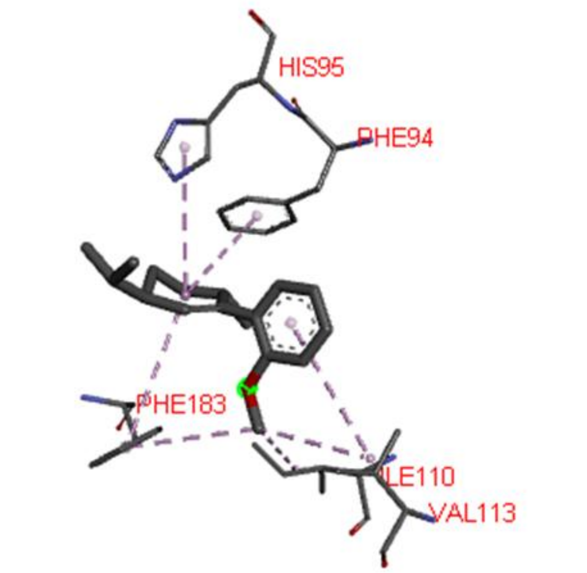
</td></tr>
<tr><td>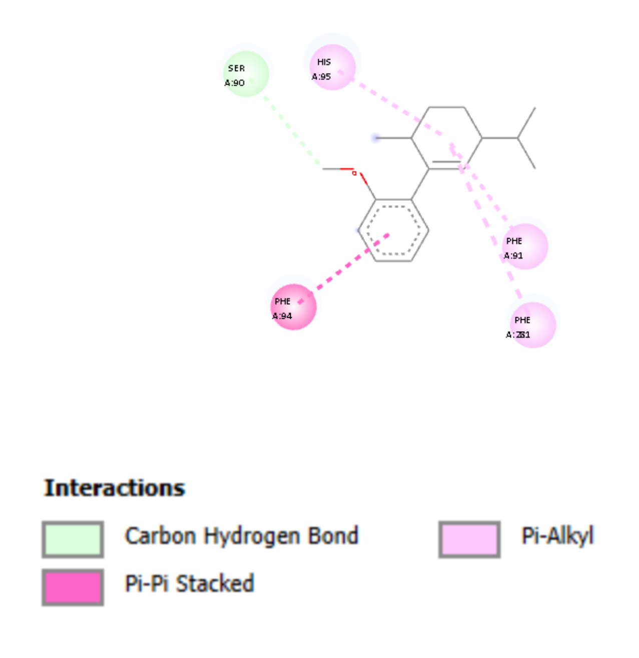
</td><td>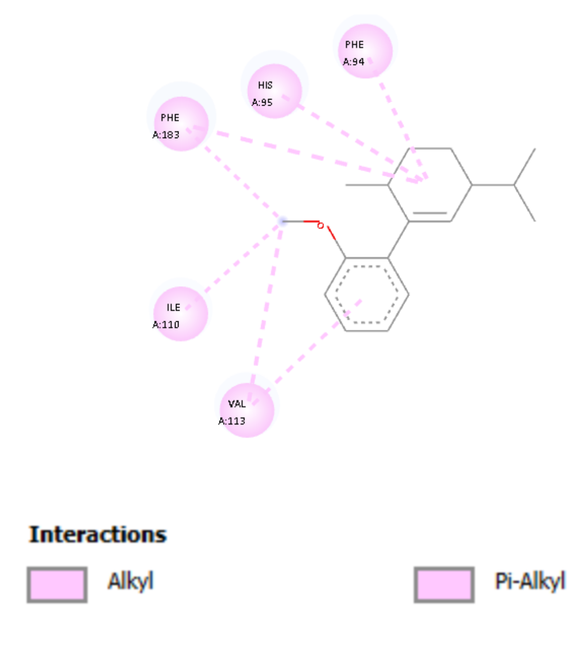
</td></tr>
<tr><td>Binding energy: -9.4</td><td>Binding energy: -8.2</td></tr>
</table>

<table>
<tr><th>7b (II)</th><th>ent-7b (II)</th></tr>
<tr><td>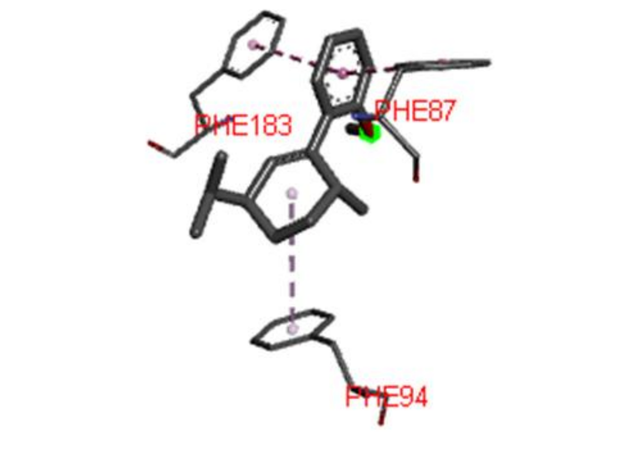
</td><td>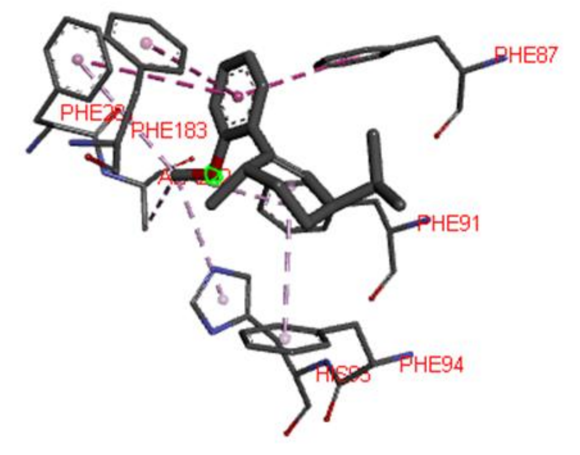
</td></tr>
<tr><td>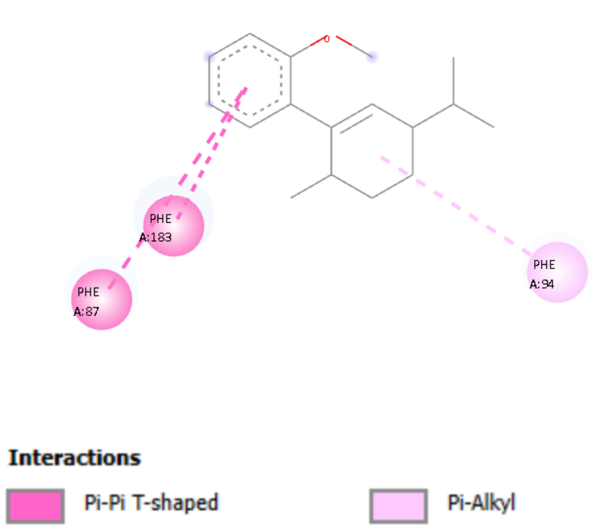
</td><td>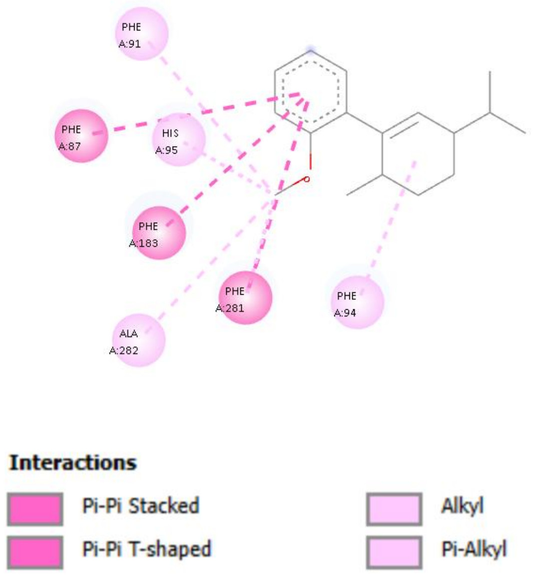
</td></tr>
<tr><td>Binding energy: -8.7</td><td>Binding energy: -8.7</td></tr>
</table>

<table>
<tr><th>7c (I)</th><th>ent-7c (I)</th></tr>
<tr><td>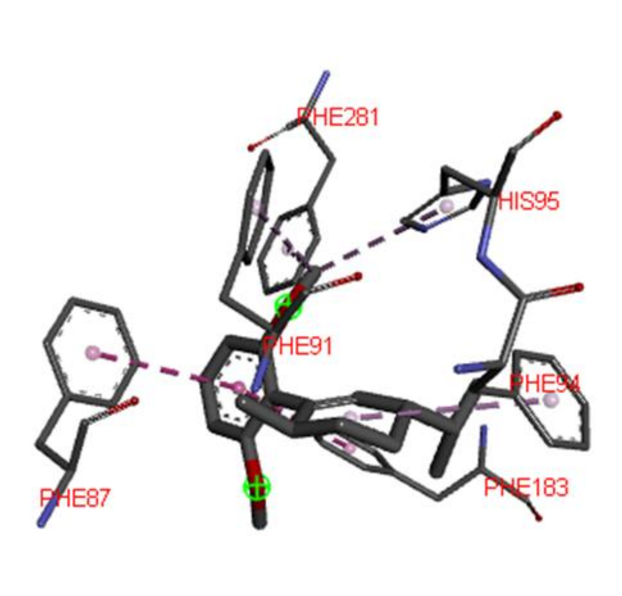
</td><td>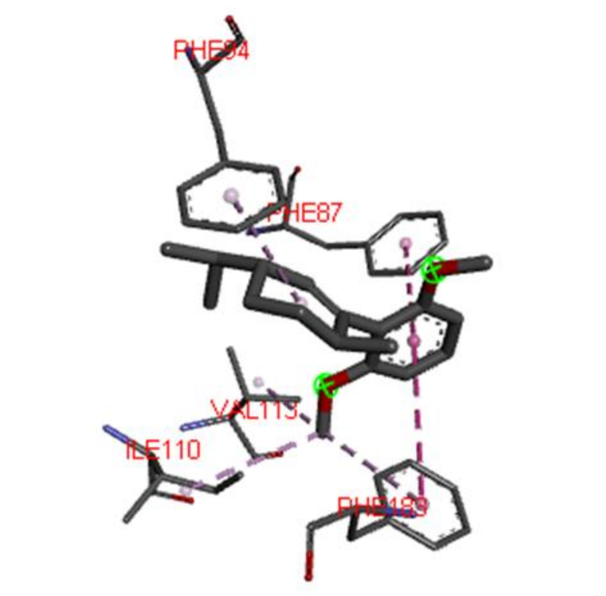
</td></tr>
<tr><td>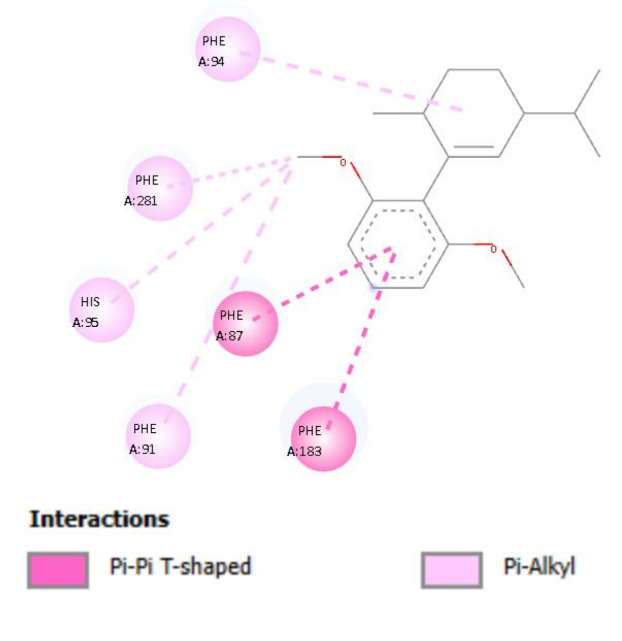
</td><td>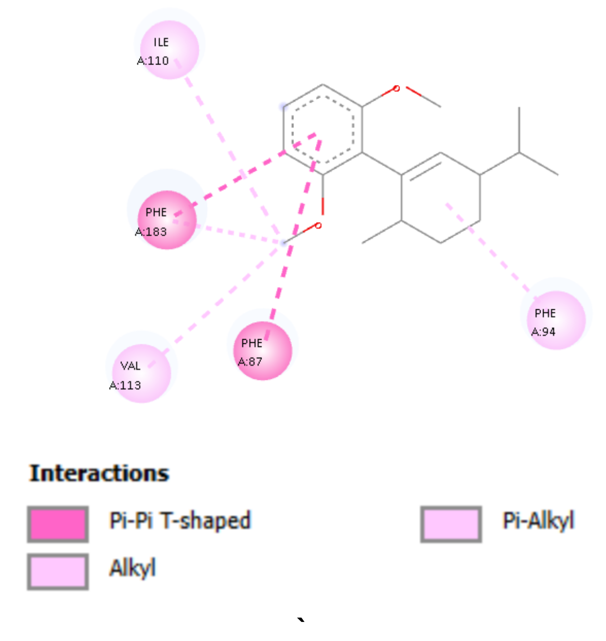
</td></tr>
<tr><td>Binding energy: -7.8</td><td>Binding energy: -8.2</td></tr>
</table>

| 7c (II) | ent-7c (II) |
| --- | --- |
| 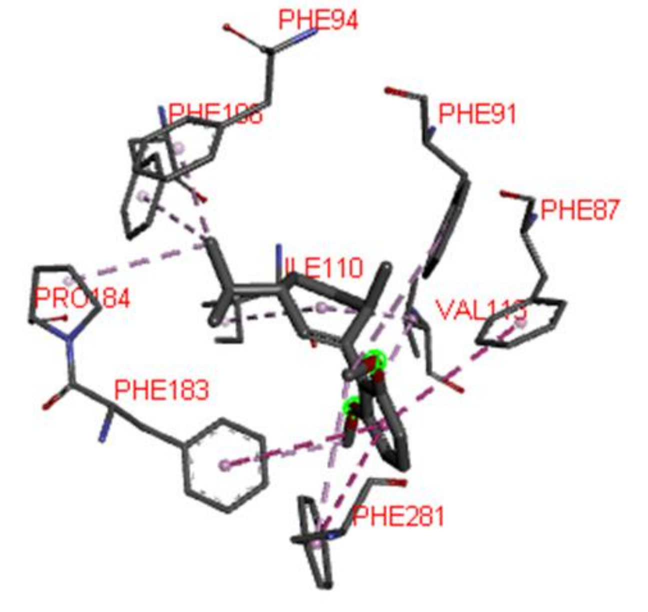 | 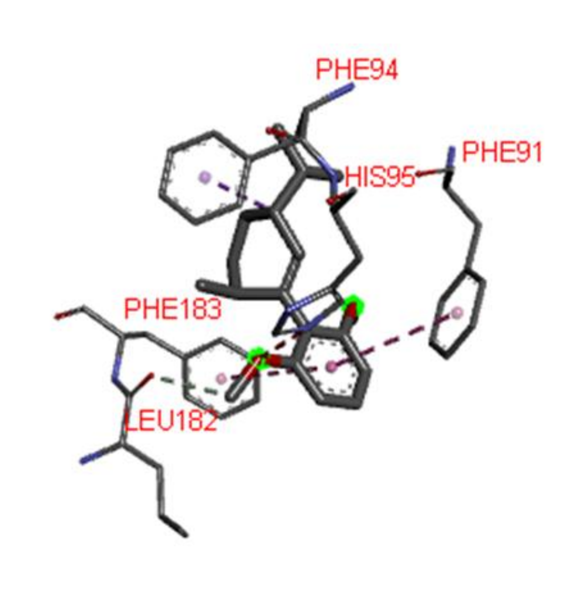 |
| 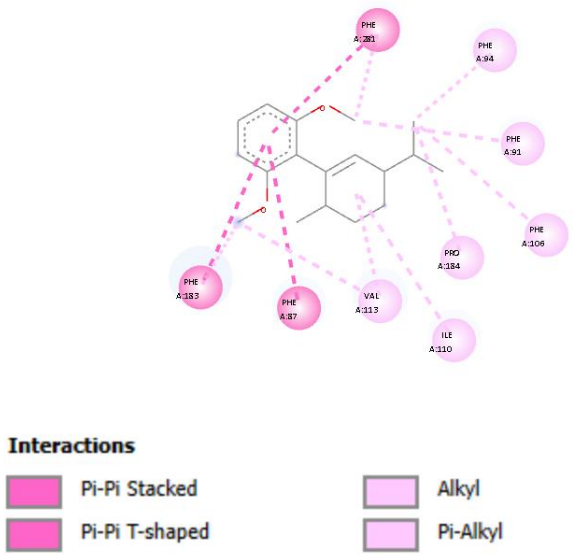 | 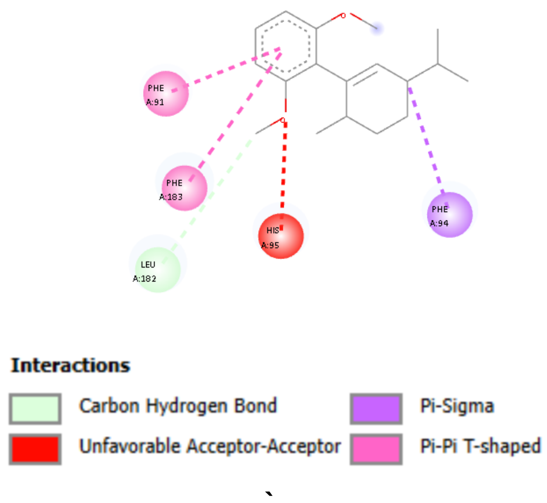 |
| **Binding energy: -8.0** | **Binding energy: -8.3** |

<table>
<tr><th>7d (I)</th><th>ent-7d (I)</th></tr>
<tr><td>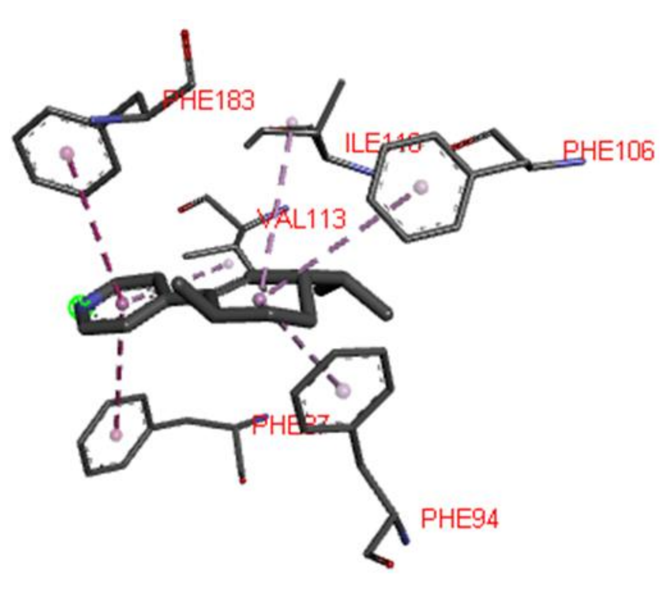
</td><td>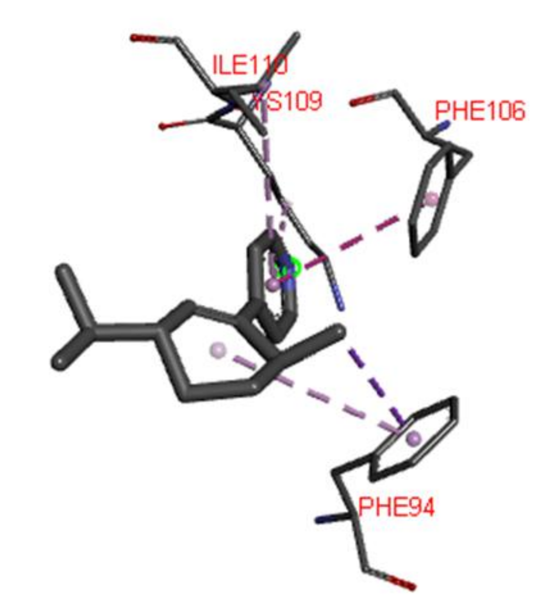
</td></tr>
<tr><td>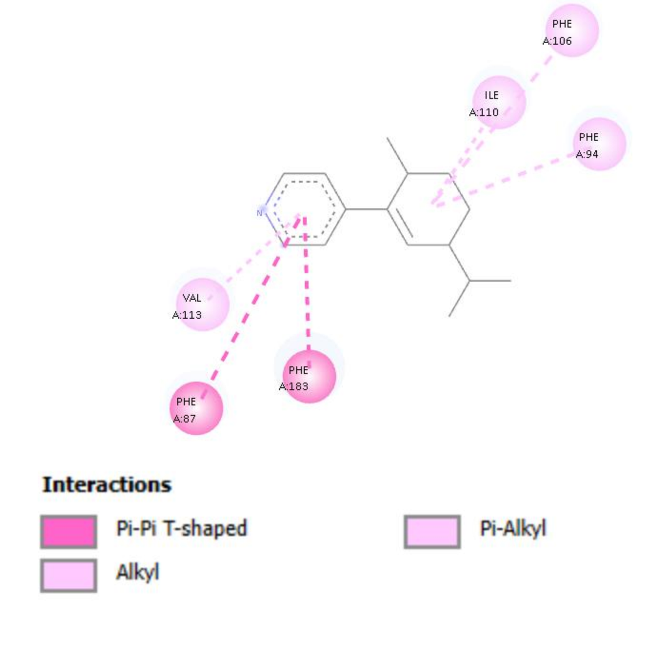
</td><td>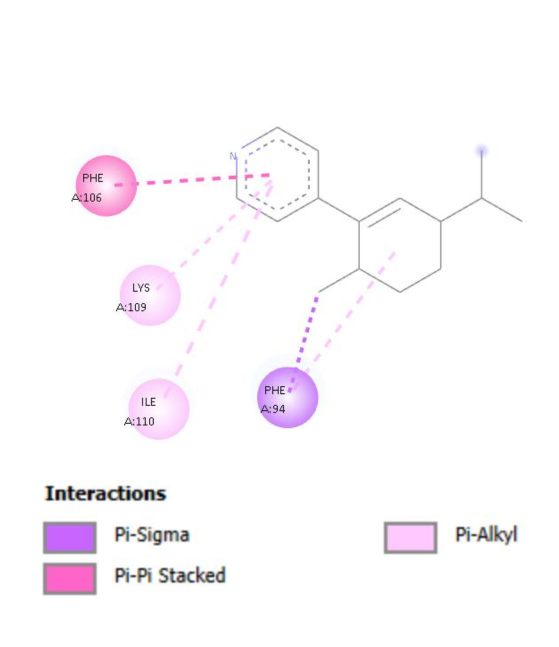
</td></tr>
<tr><td>Binding energy: -8.9</td><td>Binding energy: -8.3</td></tr>
</table>

| 7d (II) | ent-7d (II) |
| --- | --- |
| 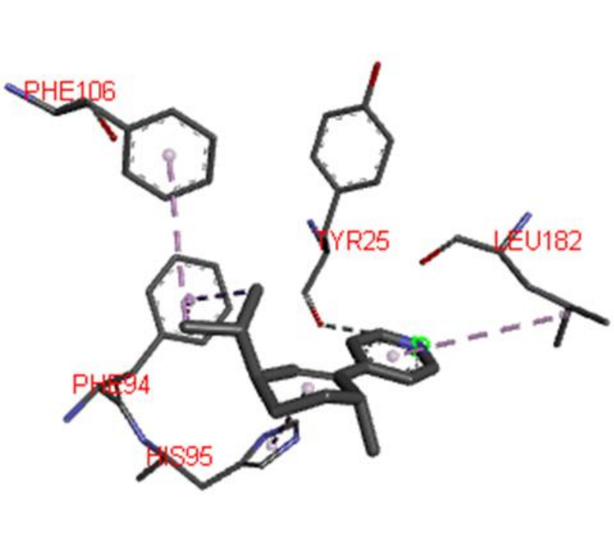 | 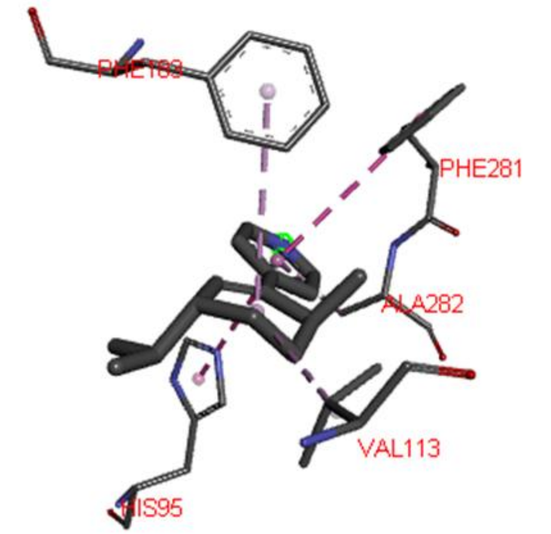 |
| 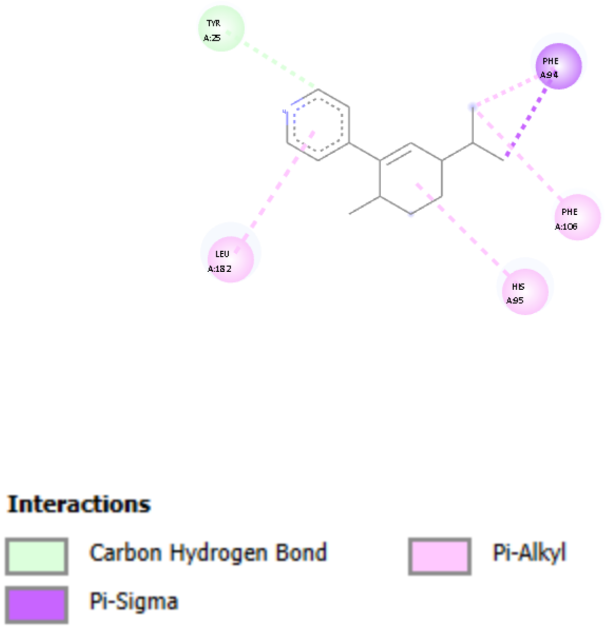 | 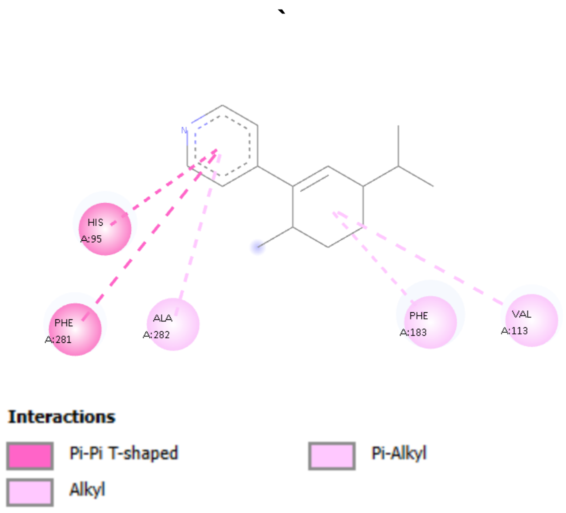 |
| **Binding energy: -8.2** | **Binding energy: -8.3** |

| 7e (I) | ent-7e (I) |
| --- | --- |
| 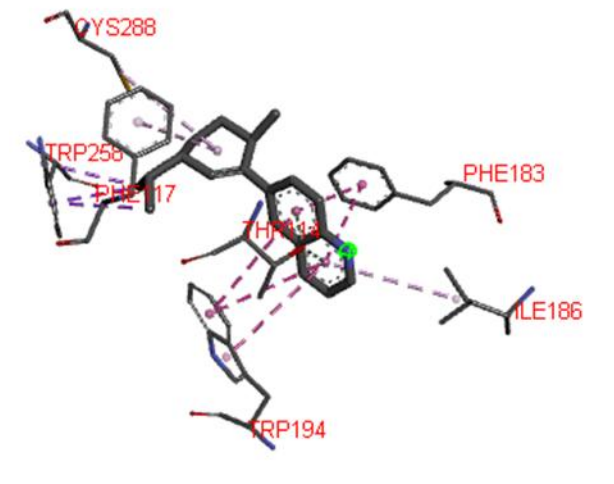 | 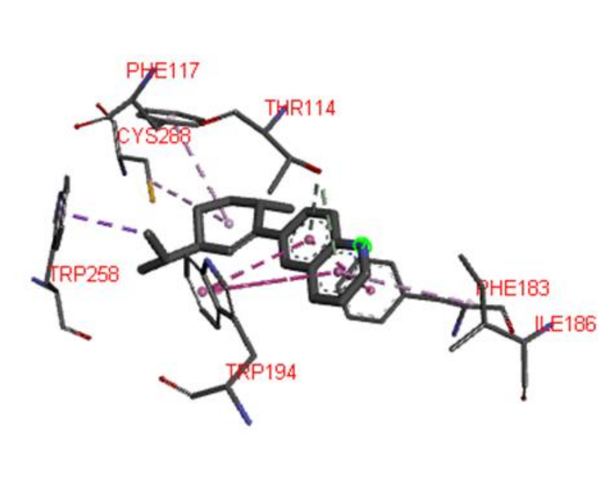 |
| 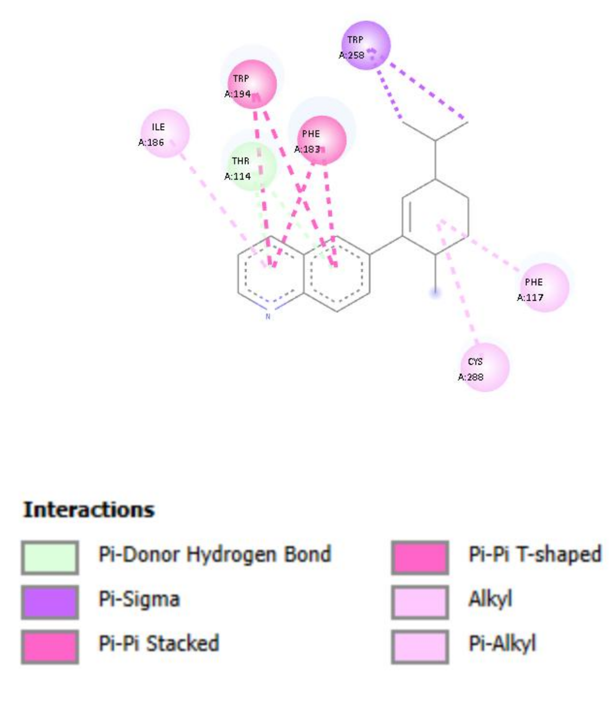 | 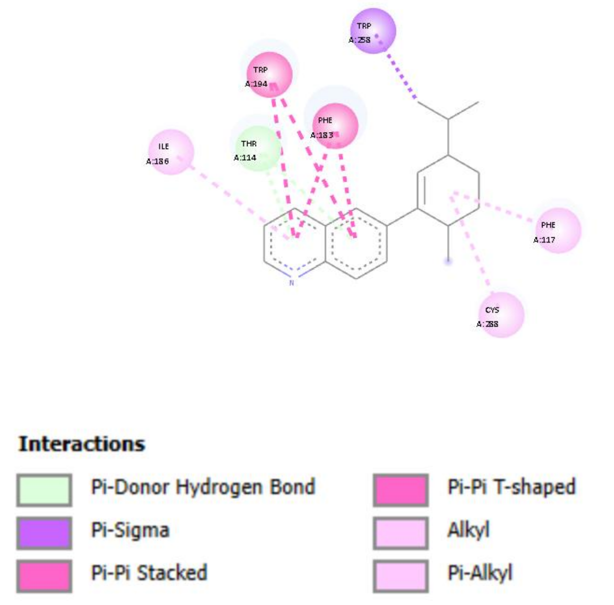 |
| **Binding energy: -10.6** | **Binding energy: -10.4** |

| 7e (II) | ent-7e (II) |
| --- | --- |
| 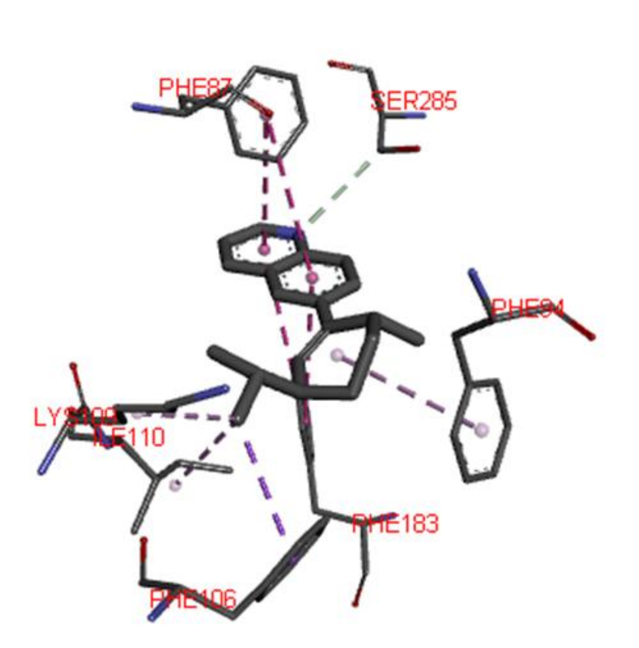 | 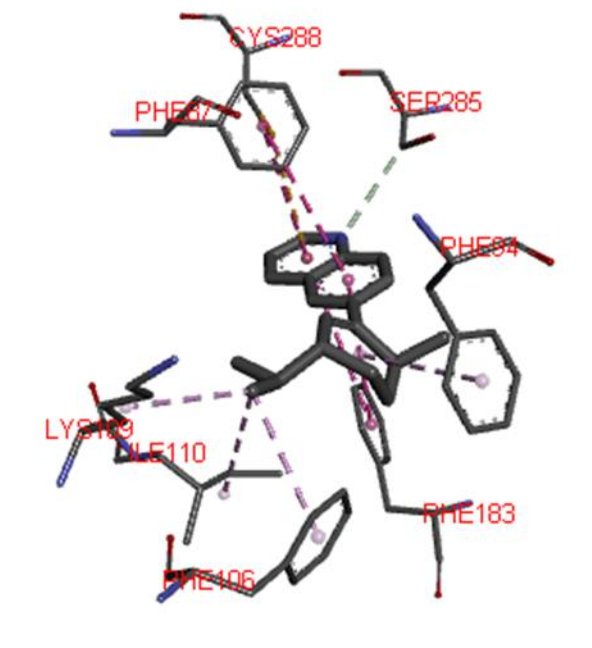 |
| 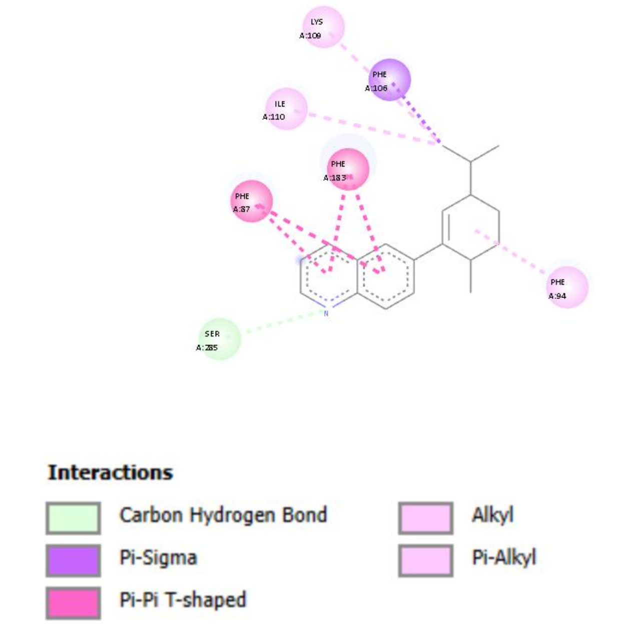 | 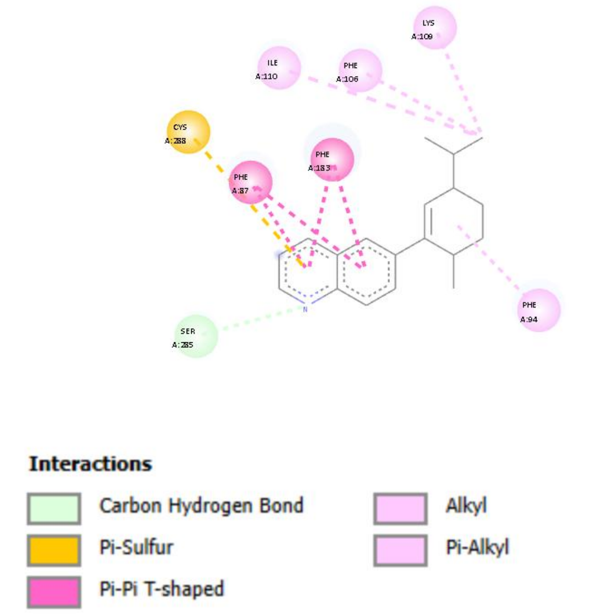 |
| **Binding energy: -10.2** | **Binding energy: -10.1** |

<table>
<tr><th>7f (I)</th><th>ent-7f (I)</th></tr>
<tr><td>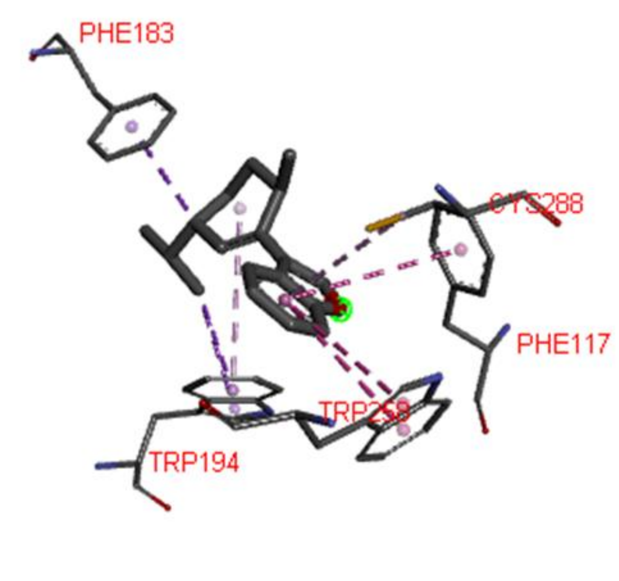
</td><td>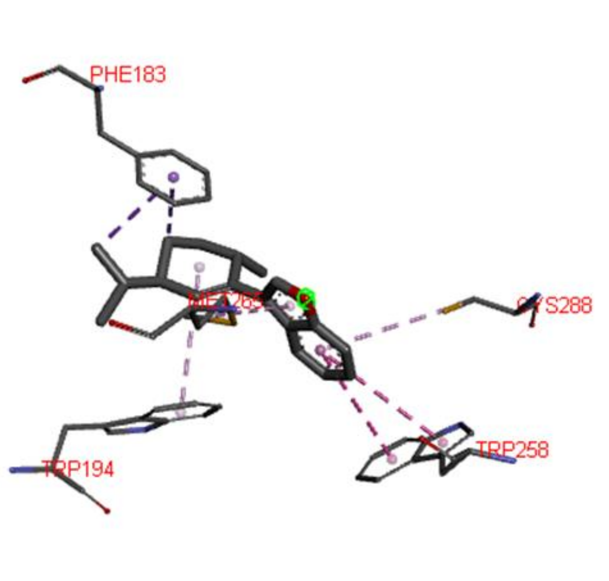
</td></tr>
<tr><td>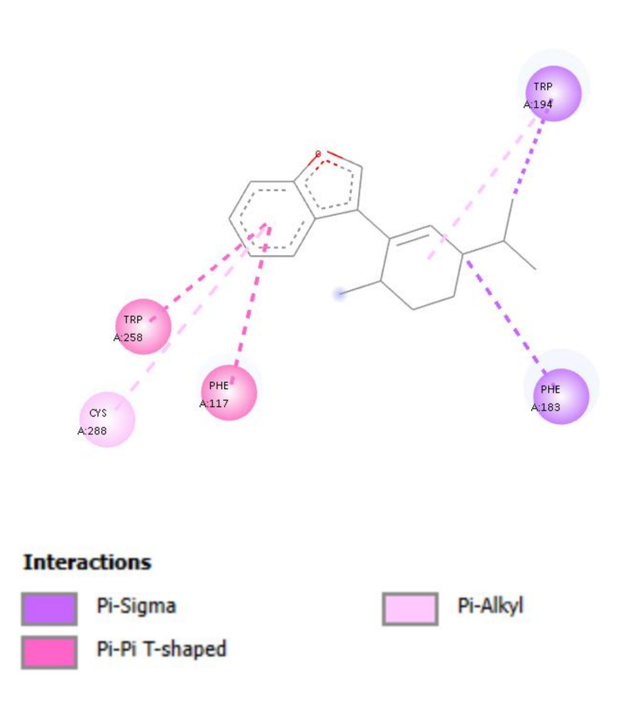
</td><td>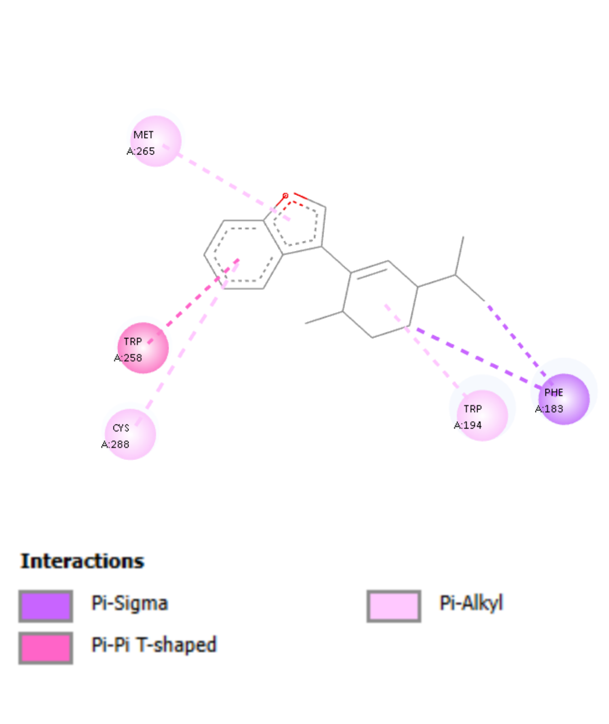
</td></tr>
<tr><td>Binding energy: -9.5</td><td>Binding energy: -9.7</td></tr>
</table>

<table>
<tr><th>7f (II)</th><th>ent-7f (II)</th></tr>
<tr><td>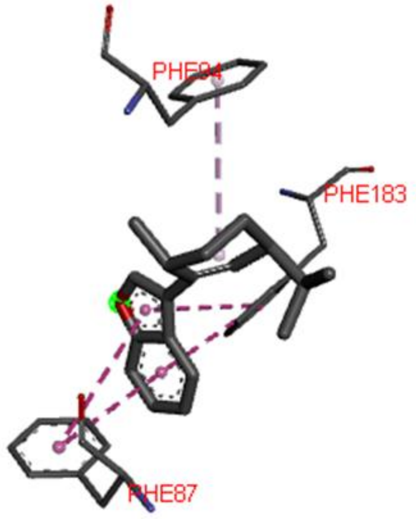
</td><td>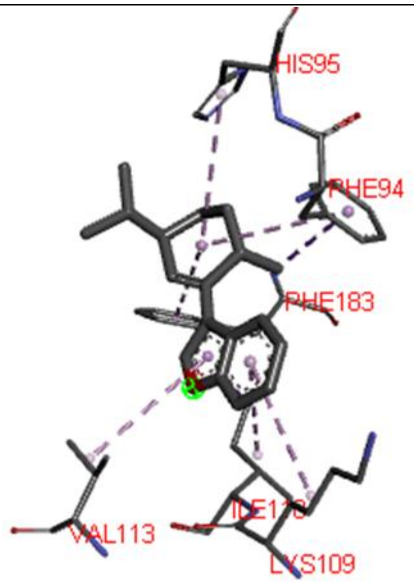
</td></tr>
<tr><td>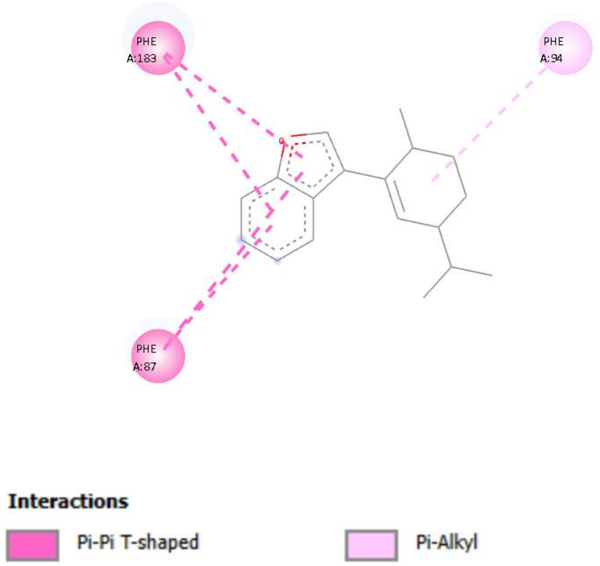
</td><td>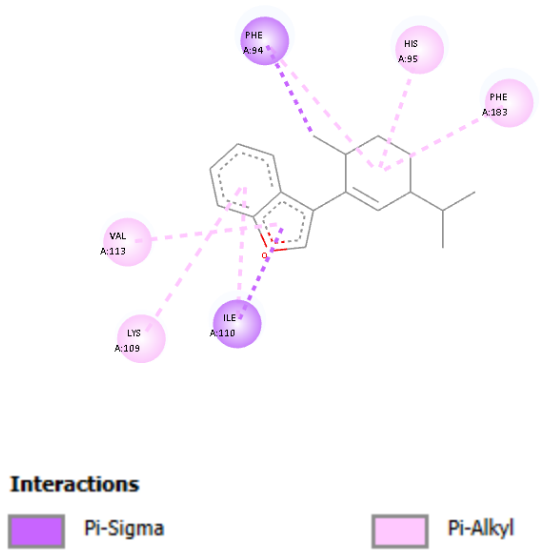
</td></tr>
<tr><td>Binding energy: -9.8</td><td>Binding energy: -9.8</td></tr>
</table>

• *Synthesized Ligands of Series **8**/ent-**8** with CB2 Receptor*

| 8a (l) | ent-8a (l) |
|---|---|
| 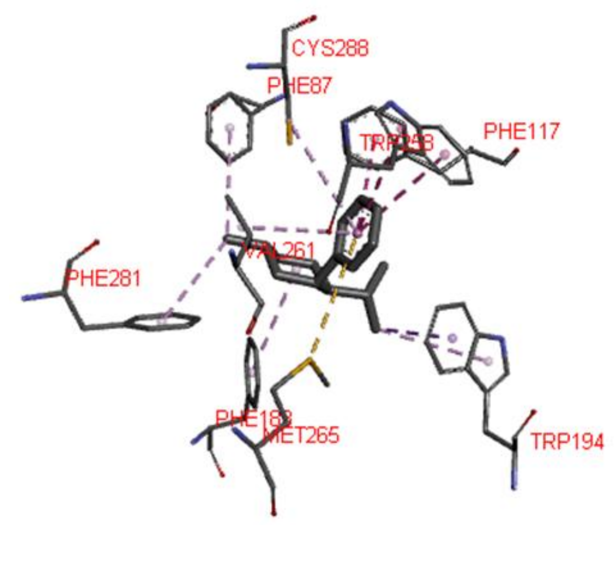 | 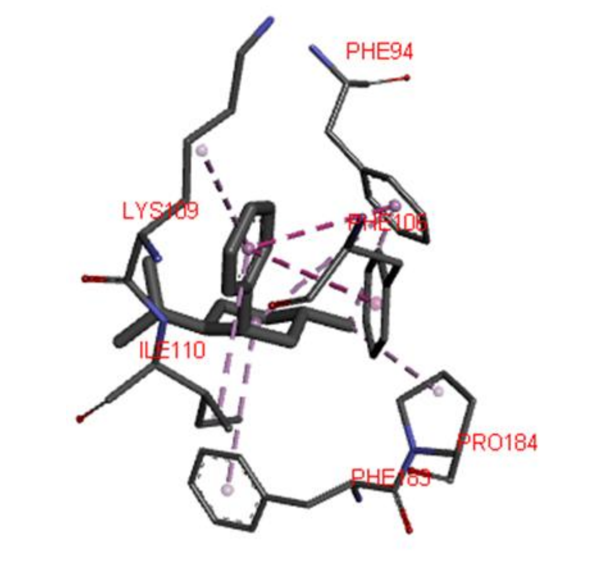 |
| 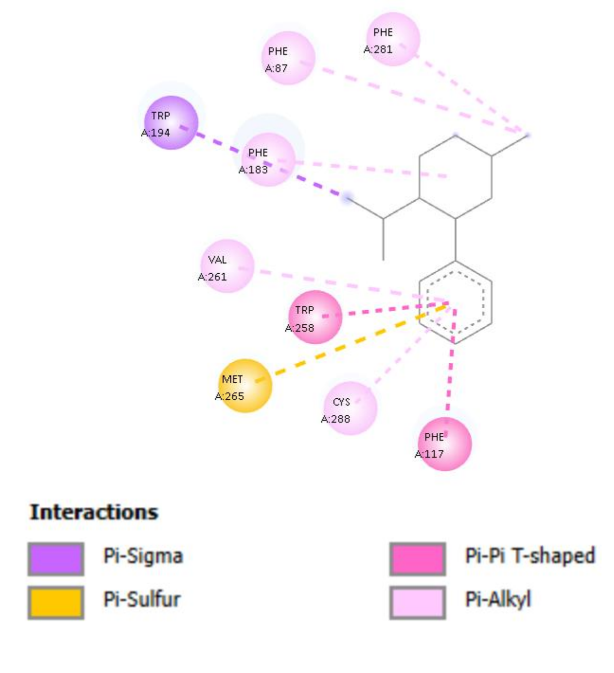 | 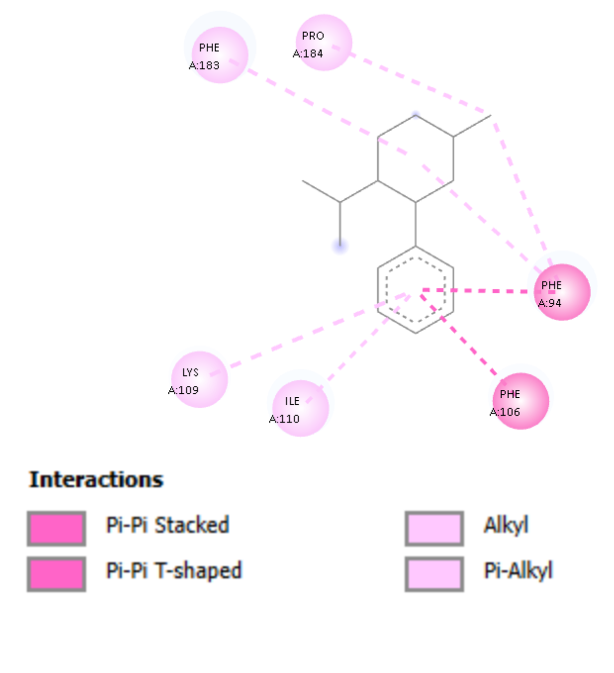 |
| **Binding energy: -8.8** | **Binding energy: -8.7** |

| 8a (II) | ent-8a (II) |
|---|---|
| 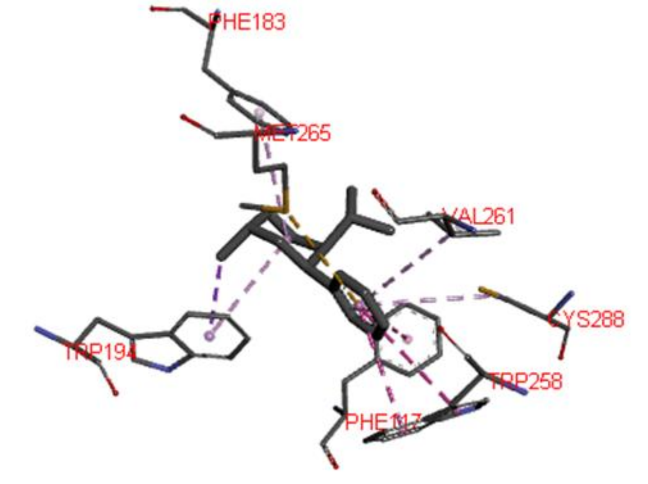 | 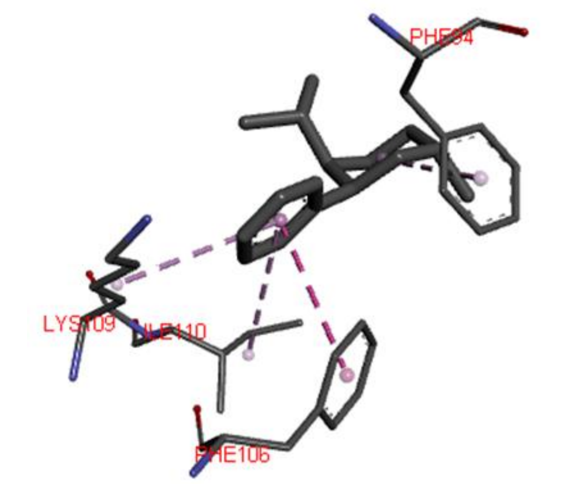 |
| 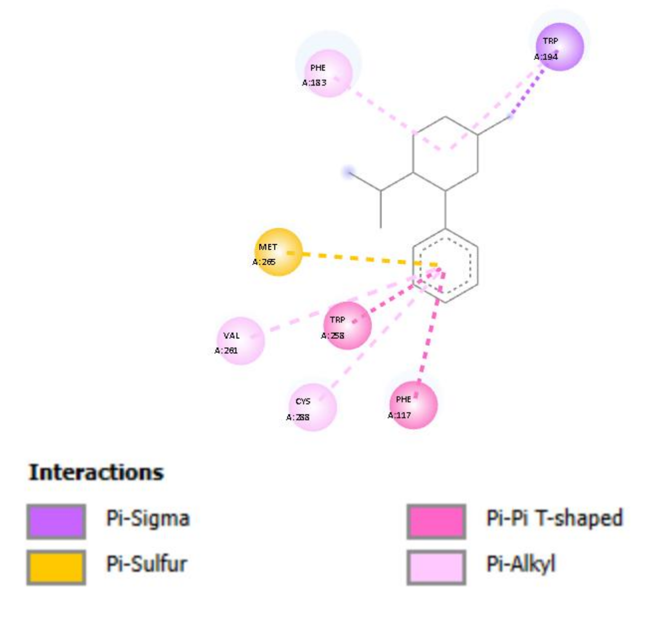 | 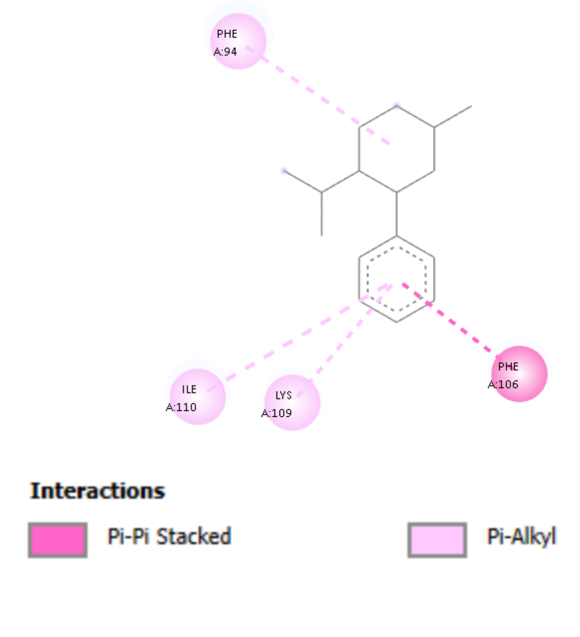 |
| **Binding energy: -9.0** | **Binding energy: -8.3** |

| 8b (I) | ent-8b (I) |
| --- | --- |
| 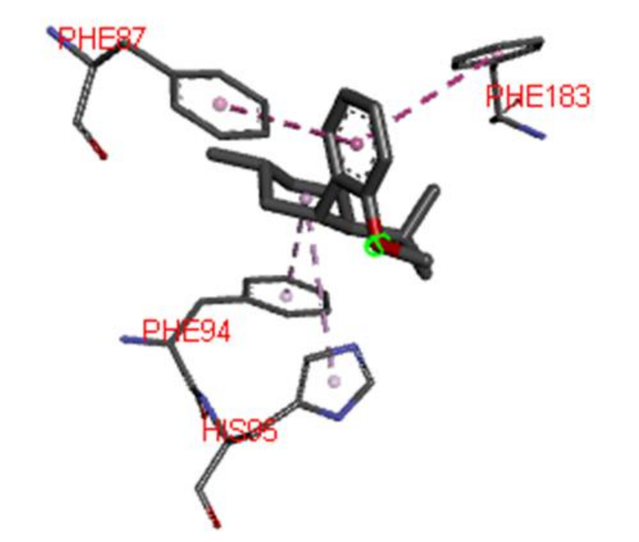  | 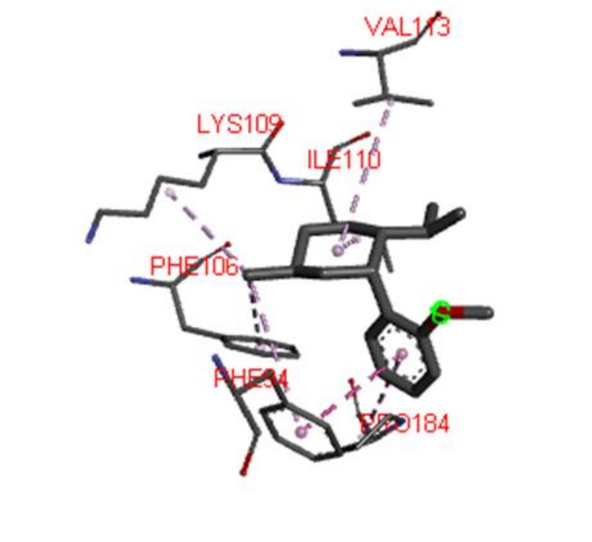  |
| 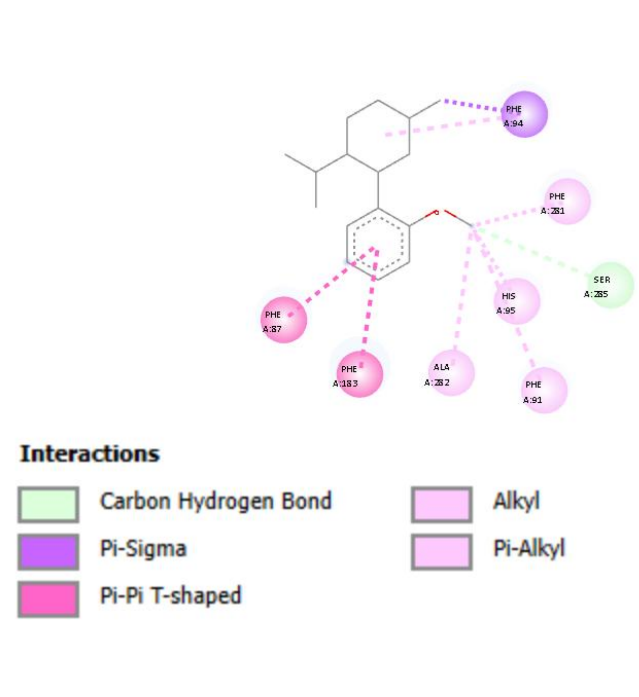  | 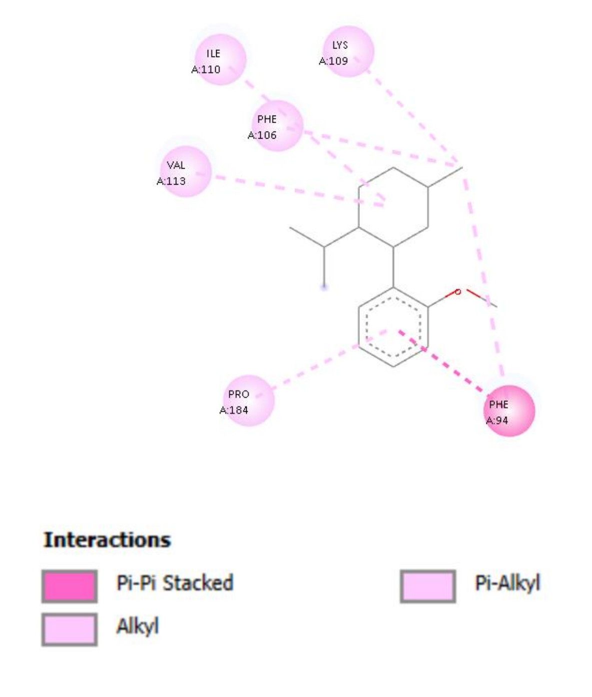  |
| **Binding energy: -8.2** | **Binding energy: -8.7** |

| 8b (II) | ent-8b (II) |
|---|---|
| 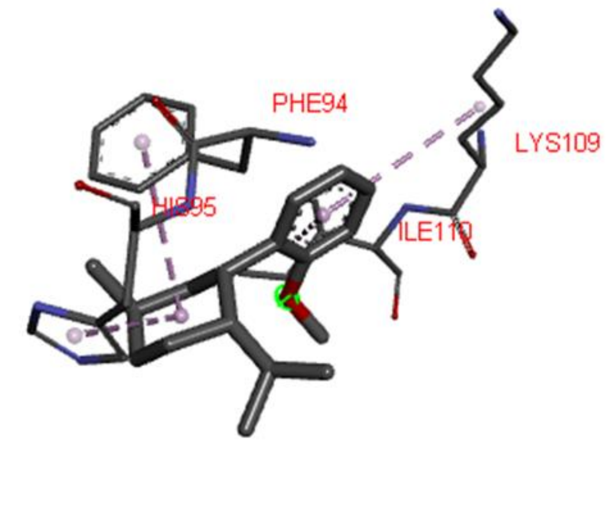 | 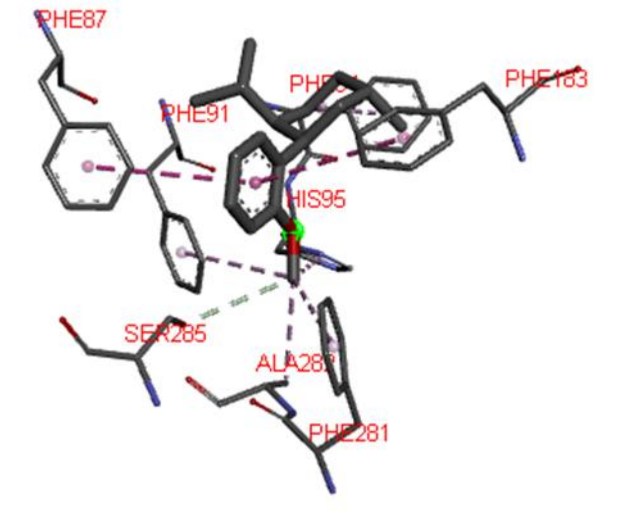 |
| 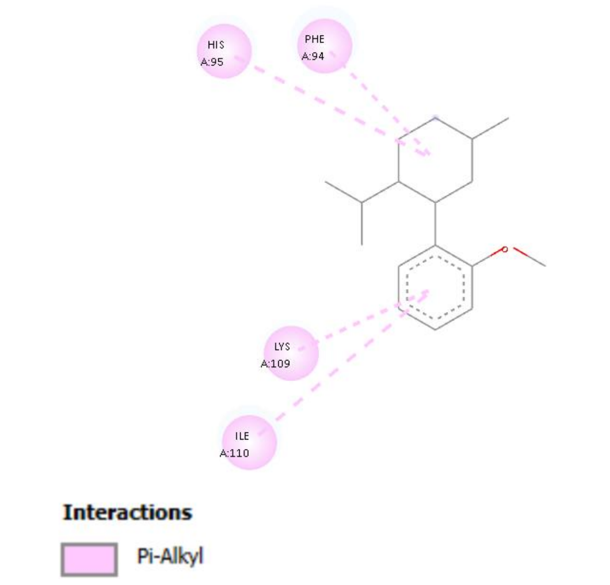 | 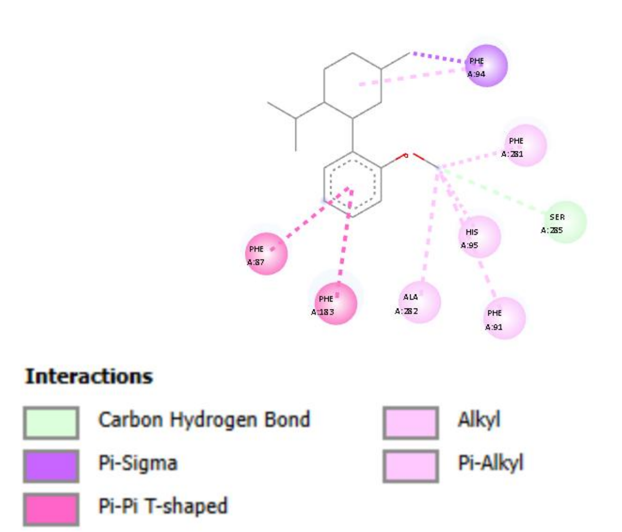 |
| **Binding energy: -8.5** | **Binding energy: -8.1** |

<table>
<tr><th>8c (I)</th><th>ent-8c (I)</th></tr>
<tr><td>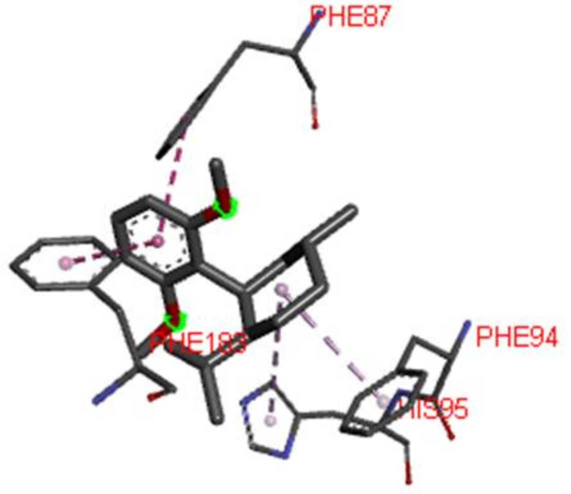
</td><td>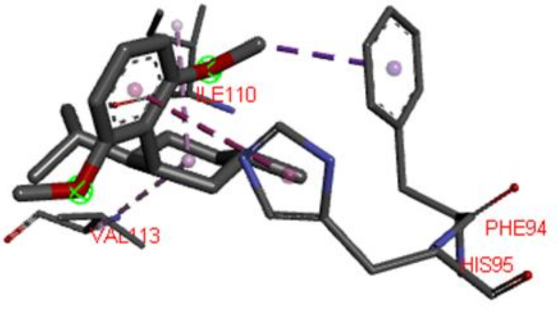
</td></tr>
<tr><td>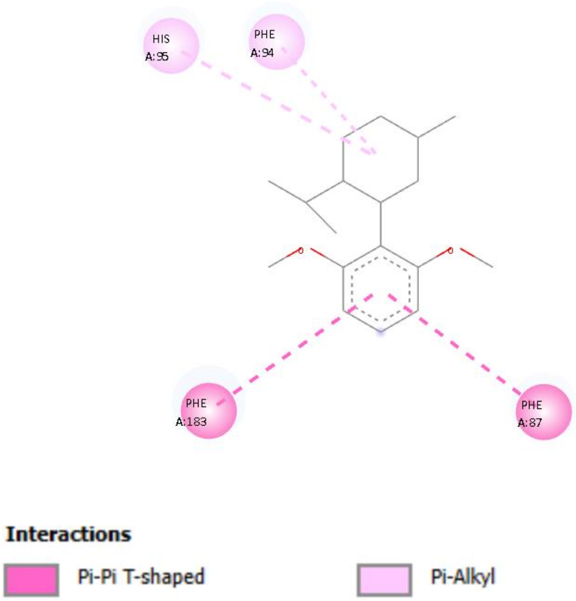
</td><td>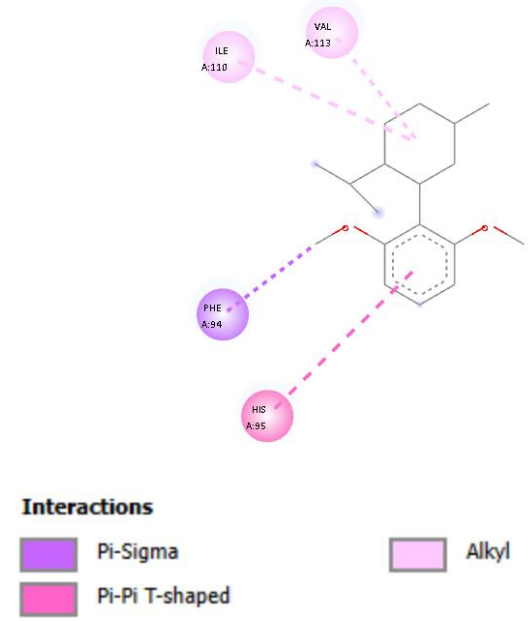
</td></tr>
<tr><td>Binding energy: -8.0</td><td>Binding energy: -7.3</td></tr>
</table>

| 8c (II) | ent-8c (II) |
| --- | --- |
| 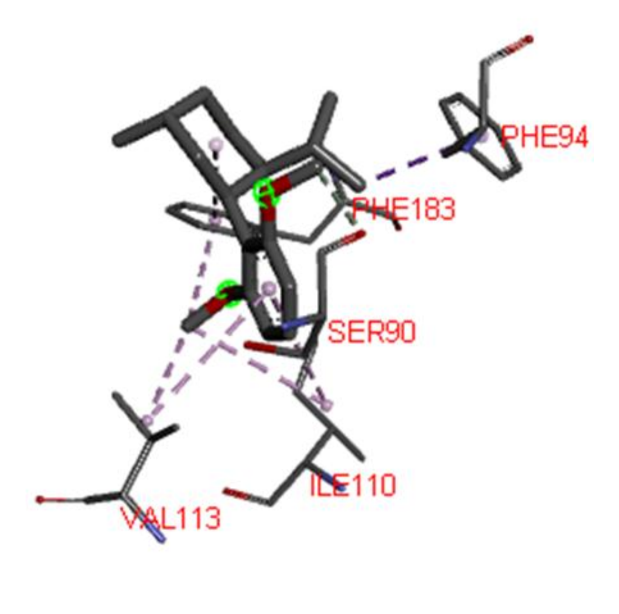 | 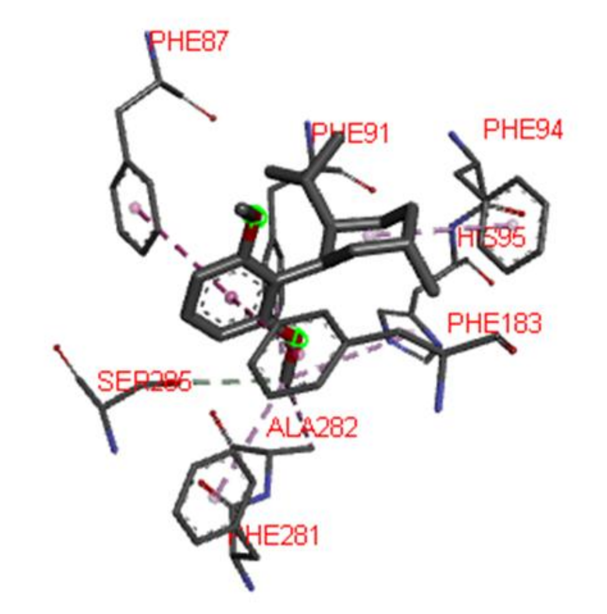 |
| 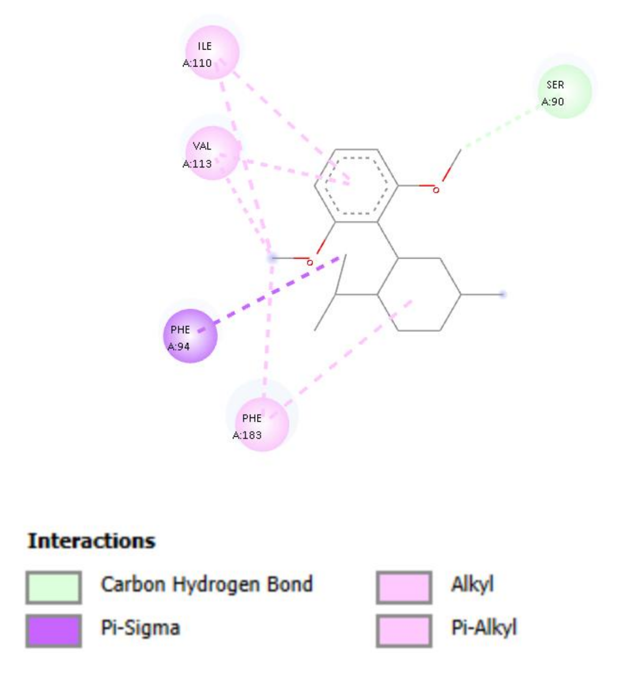 | 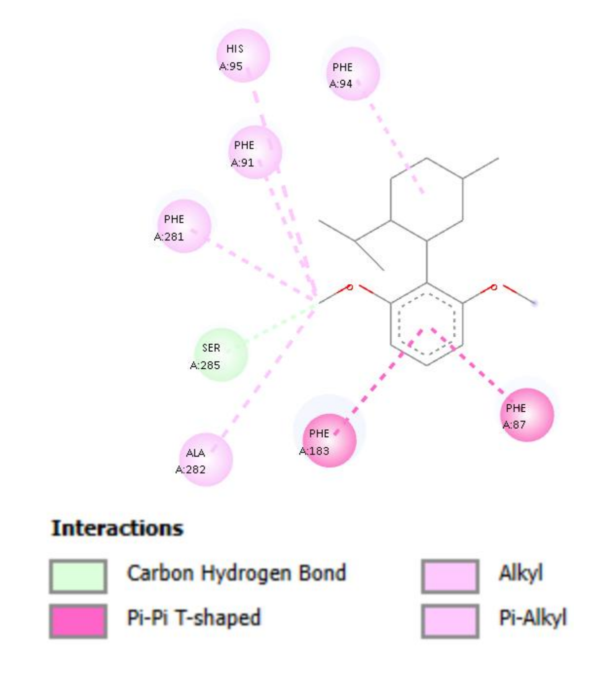 |
| **Binding energy: -7.6** | **Binding energy: -7.8** |

<table>
<tr><th>8d (I)</th><th>ent-8d (I)</th></tr>
<tr><td>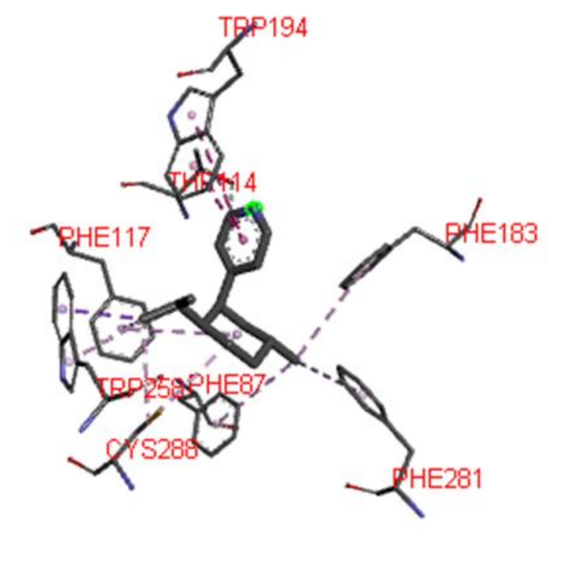
</td><td>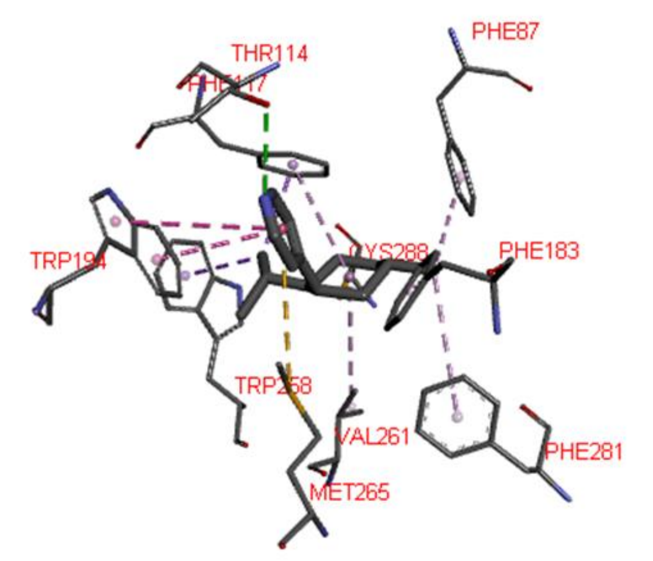
</td></tr>
<tr><td>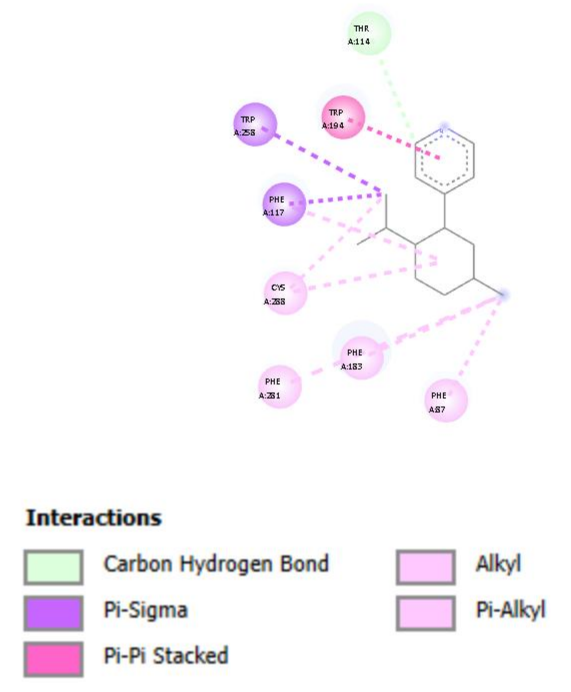
</td><td>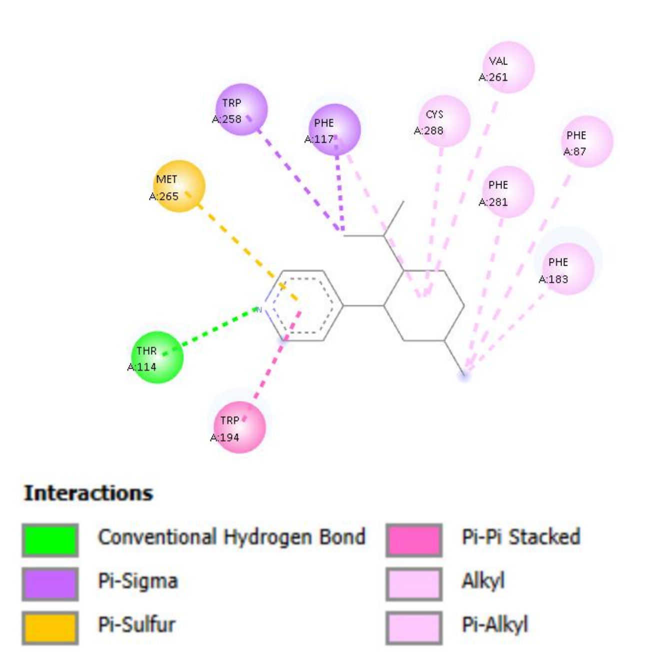
</td></tr>
<tr><td>Binding energy: -7.9</td><td>Binding energy: -8.3</td></tr>
</table>

| 8d (II) | ent-8d (II) |
| --- | --- |
| 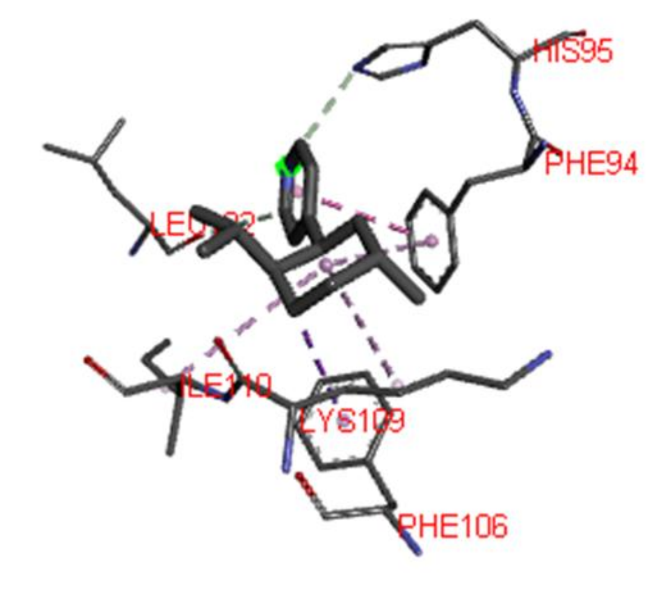 | 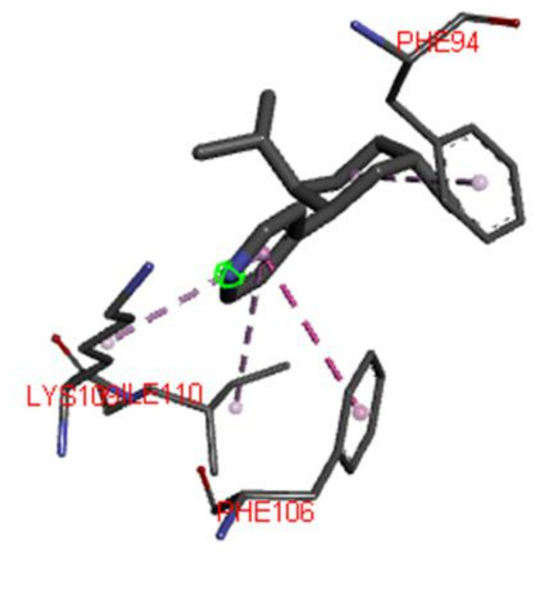 |
| 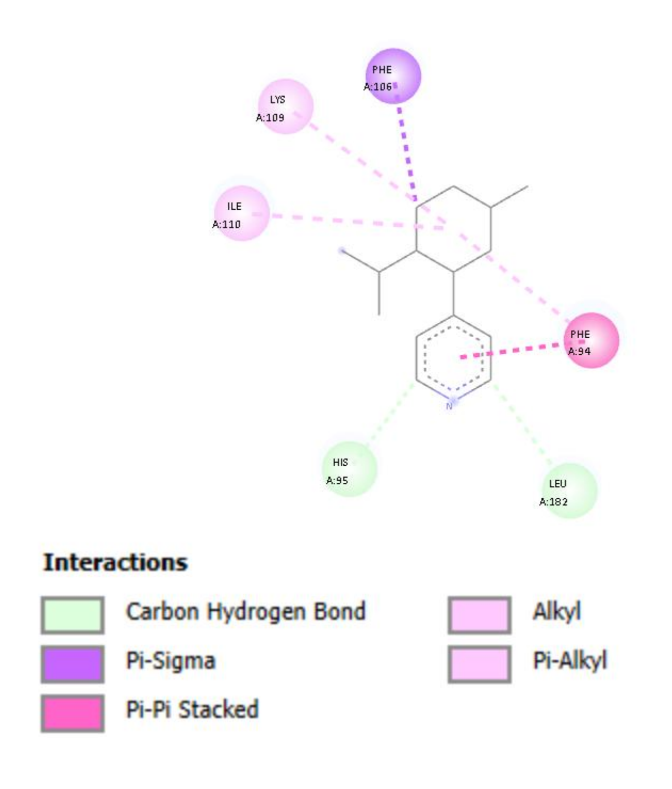 | 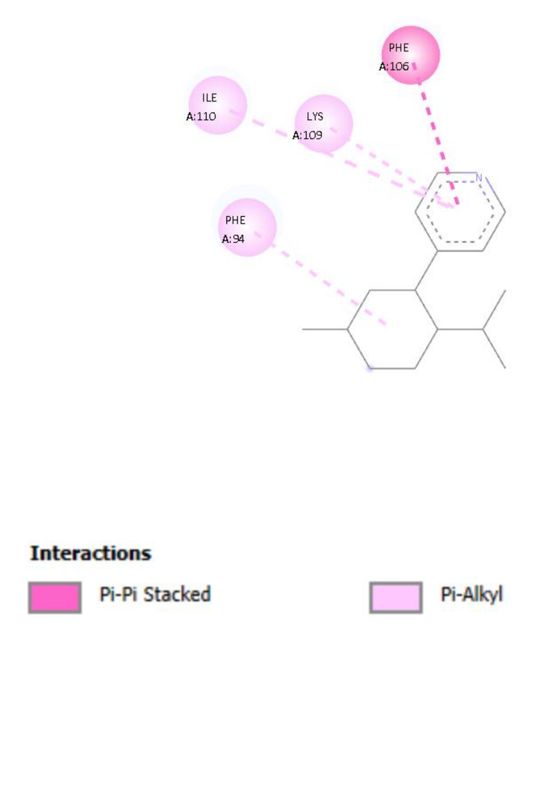 |
| **Binding energy: -8.3** | **Binding energy: -7.6** |

<table>
<tr><th>8e (I)</th><th>ent-8e (I)</th></tr>
<tr><td>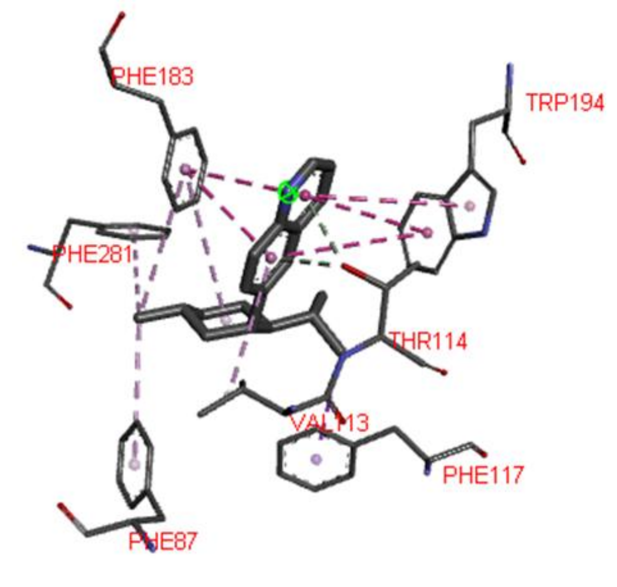
</td><td>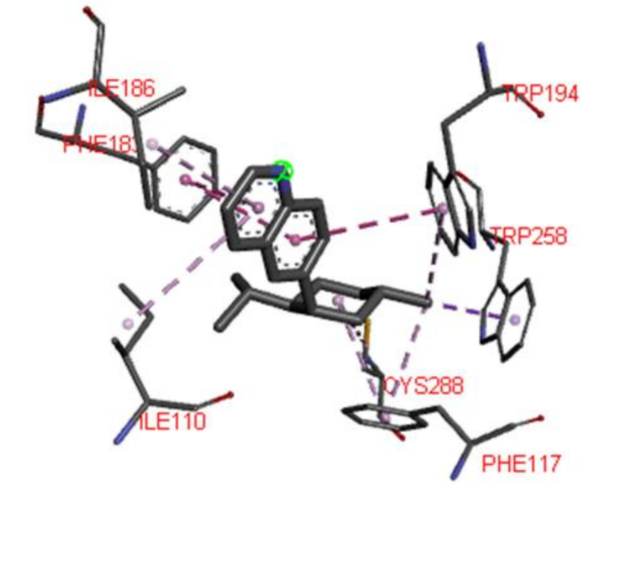
</td></tr>
<tr><td>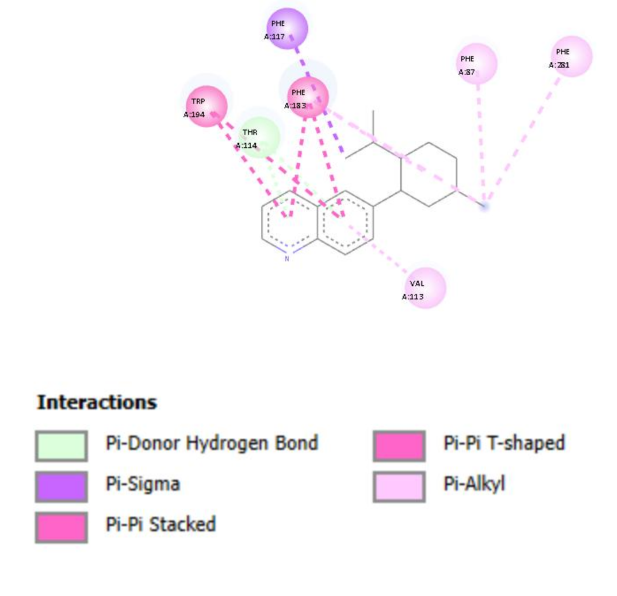
</td><td>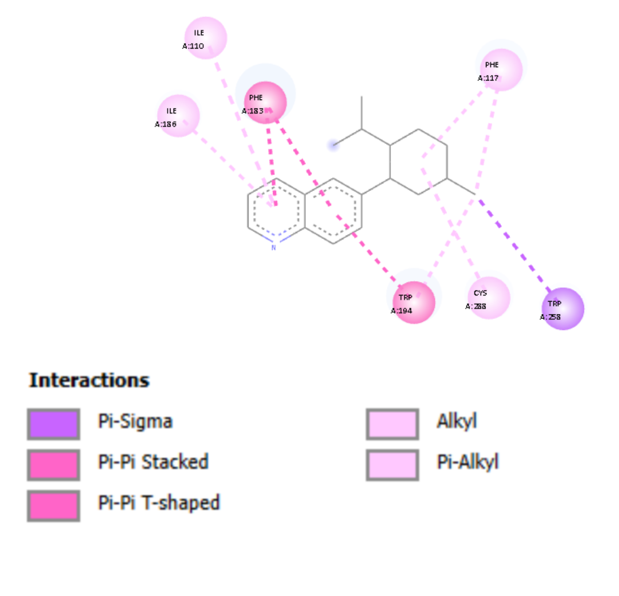
</td></tr>
<tr><td>Binding energy: -10.0</td><td>Binding energy: -9.6</td></tr>
</table>

| 8e (II) | ent-8e (II) |
| --- | --- |
| 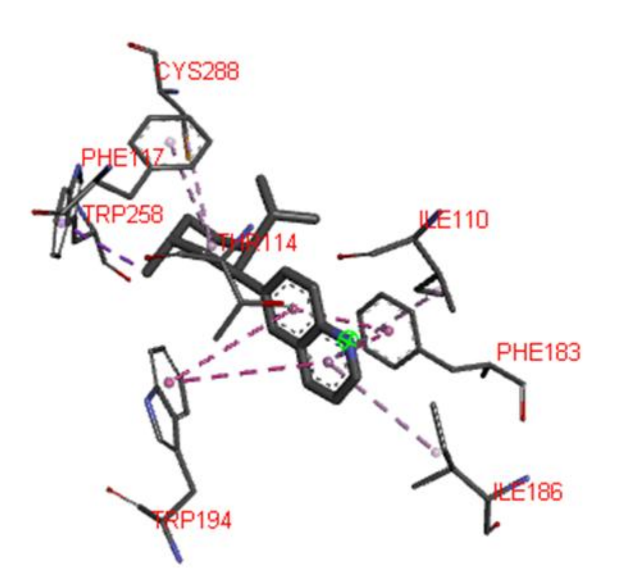 | 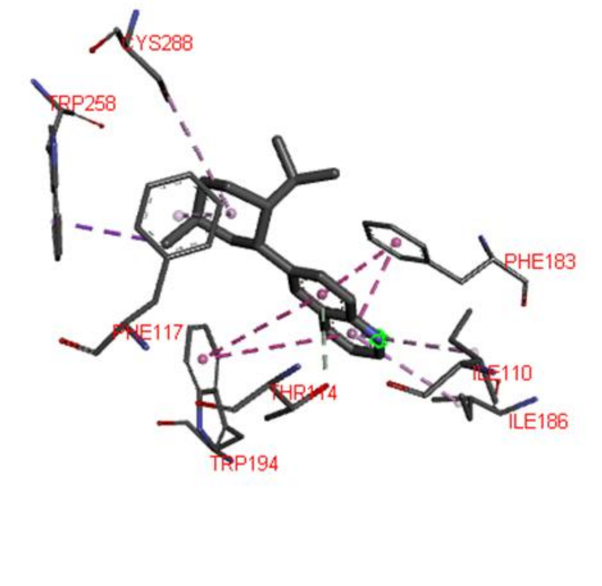 |
| 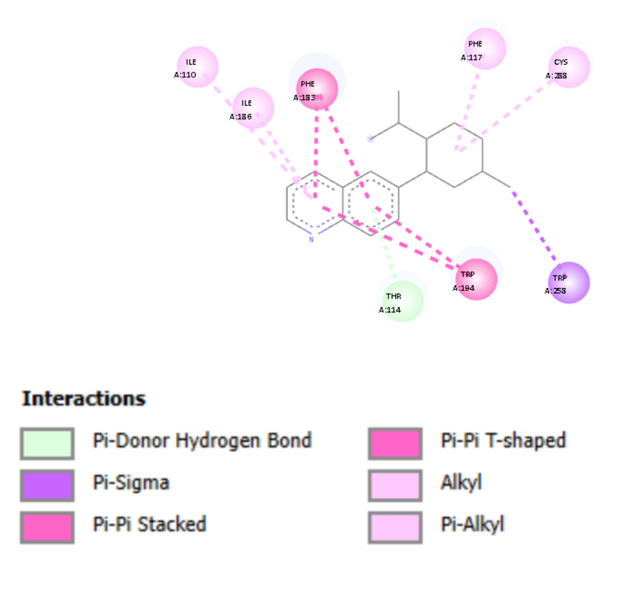 | 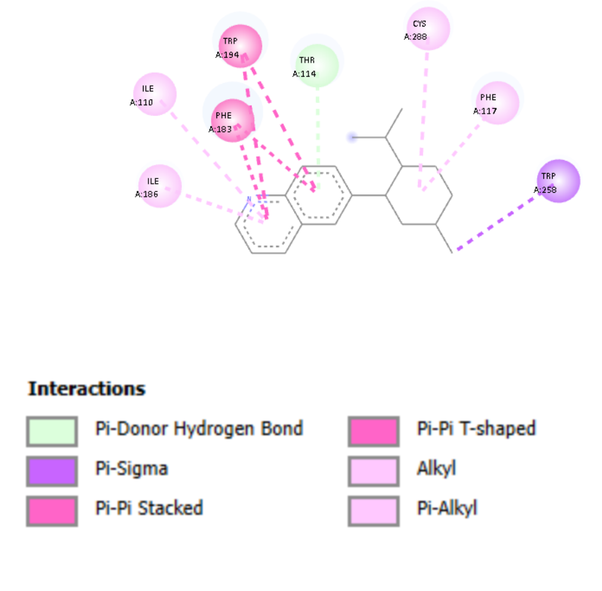 |
| **Binding energy: -10.0** | **Binding energy: -9.7** |

<table>
<tr><th>8f (I)</th><th>ent-8f (I)</th></tr>
<tr><td>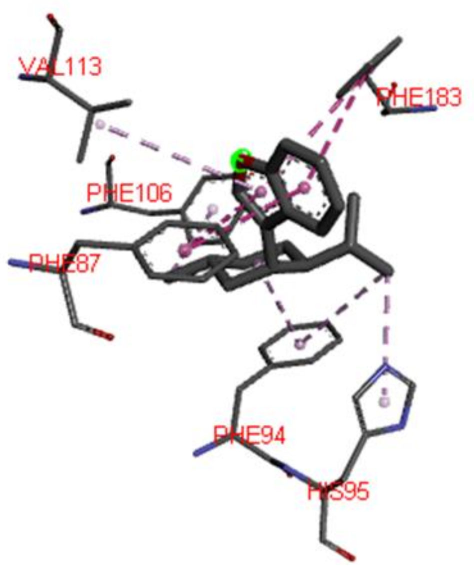
</td><td>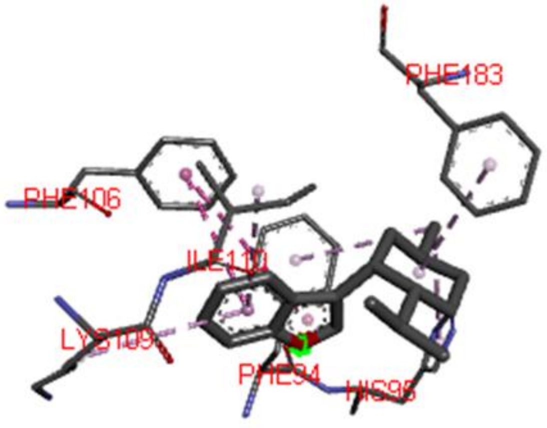
</td></tr>
<tr><td>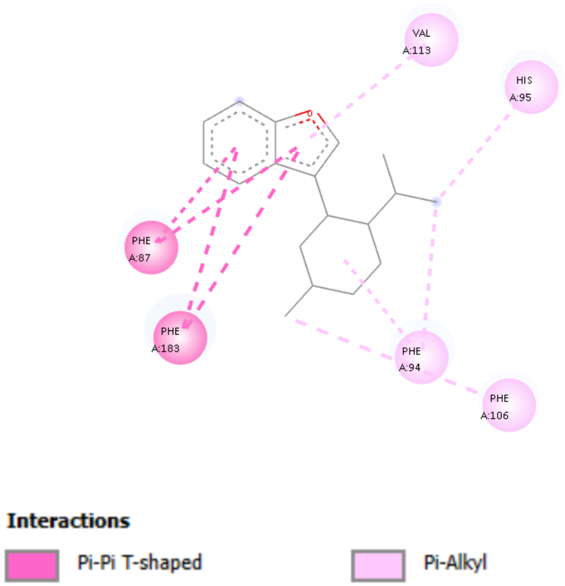
</td><td>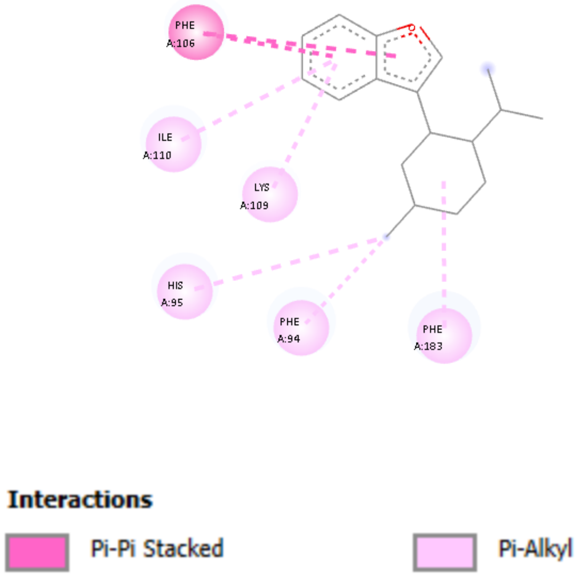
</td></tr>
<tr><td>Binding energy: -8.9</td><td>Binding energy: -9.2</td></tr>
</table>

<table>
<tr><th>8f (II)</th><th>ent-8f (II)</th></tr>
<tr><td>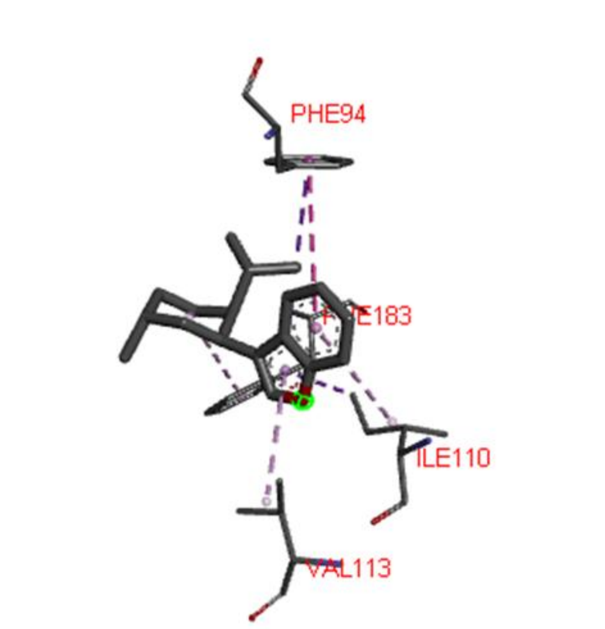
</td><td>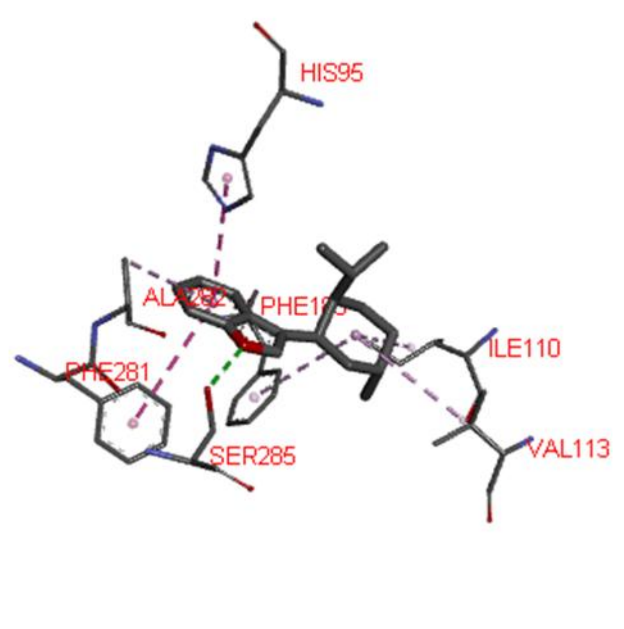
</td></tr>
<tr><td>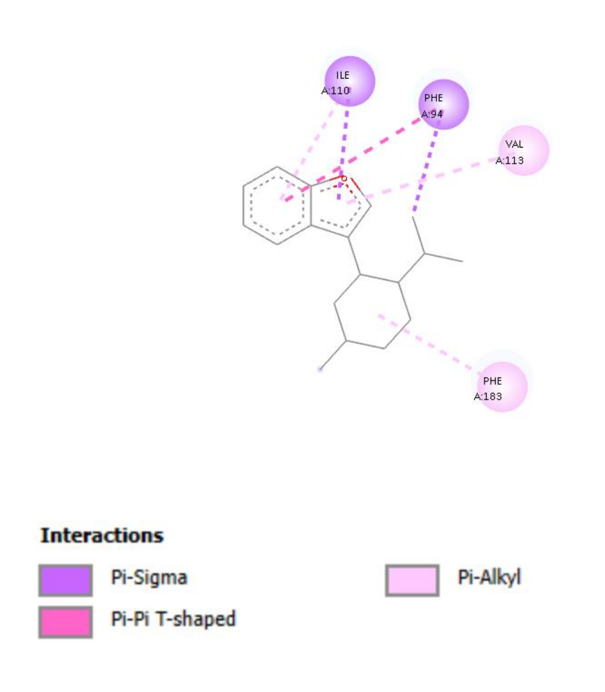
</td><td>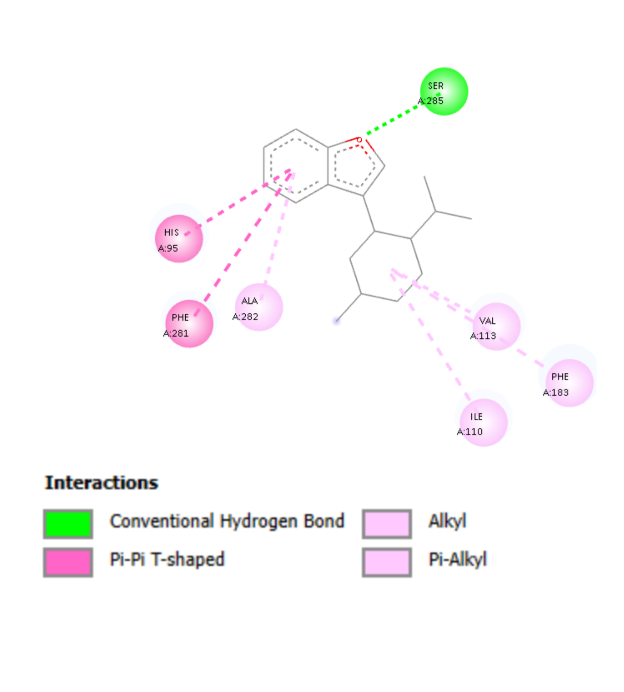
</td></tr>
<tr><td>Binding energy: -9.6</td><td>Binding energy: -8.9</td></tr>
</table>

• *Synthesized Ligands of Series **9**/ent-**9** with CB2 Receptor*

<table>
<tr><th>9a (I)</th><th>ent-9a (I)</th></tr>
<tr><td>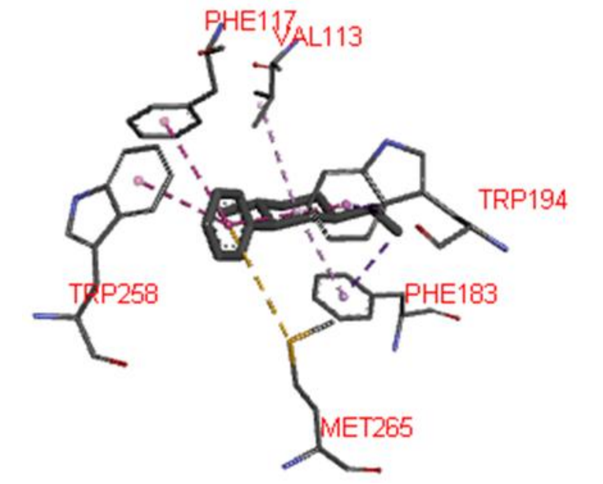
</td><td>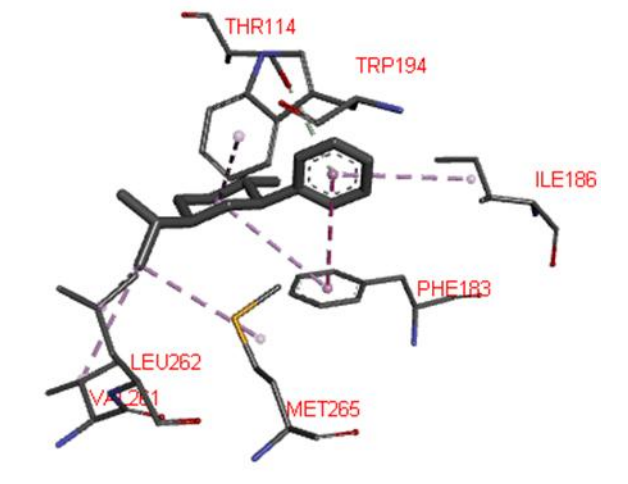
</td></tr>
<tr><td>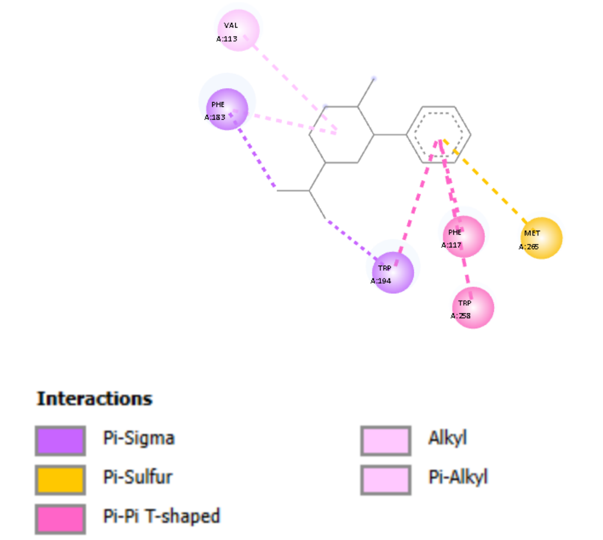
</td><td>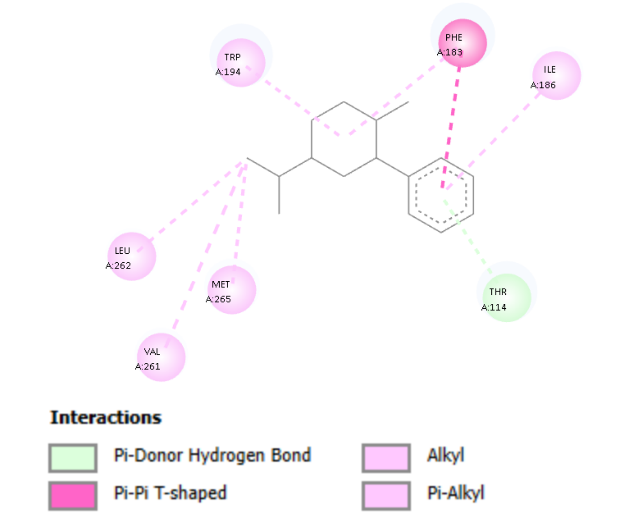
</td></tr>
<tr><td>Binding energy: -9.0</td><td>Binding energy: -8.9</td></tr>
</table>

| 9a (II) | ent-9a (II) |
|---|---|
| 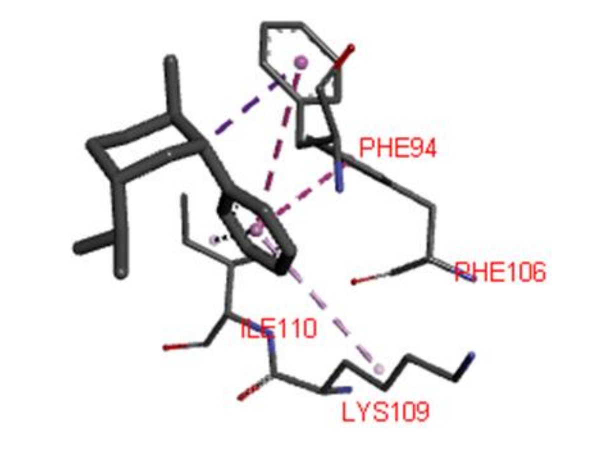 | 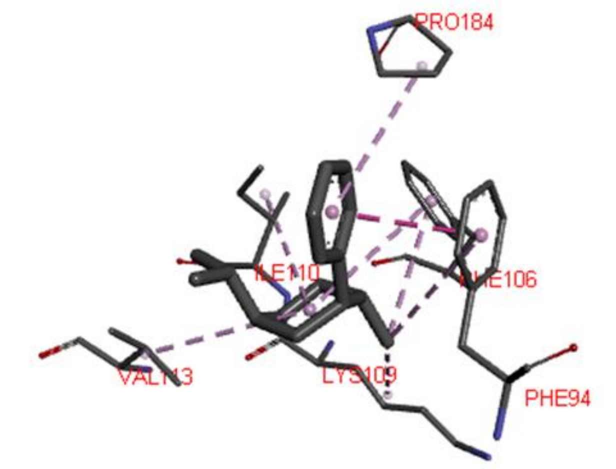 |
| 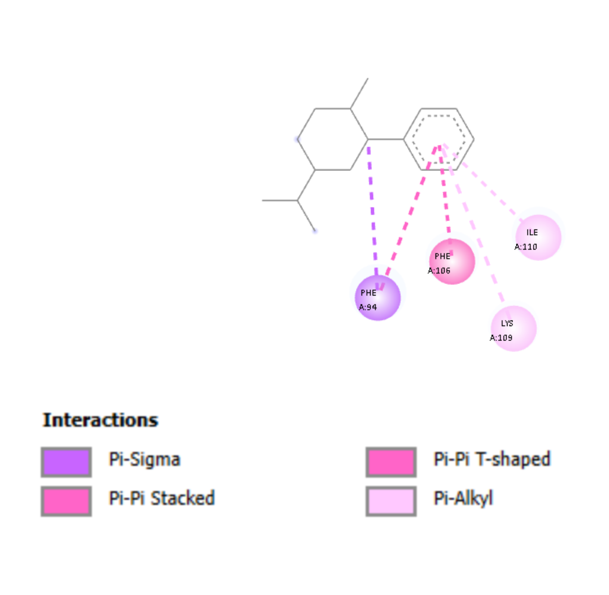 | 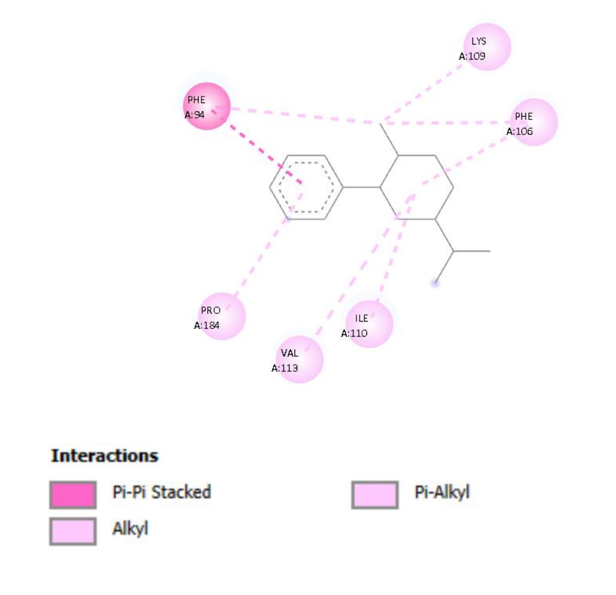 |
| **Binding energy: -9.2** | **Binding energy: -9.4** |

<table>
<tr><th>9b (I)</th><th>ent-9b (I)</th></tr>
<tr><td>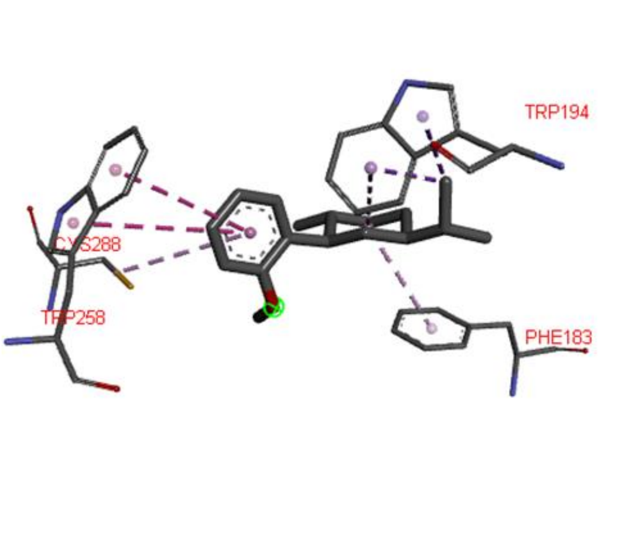
</td><td>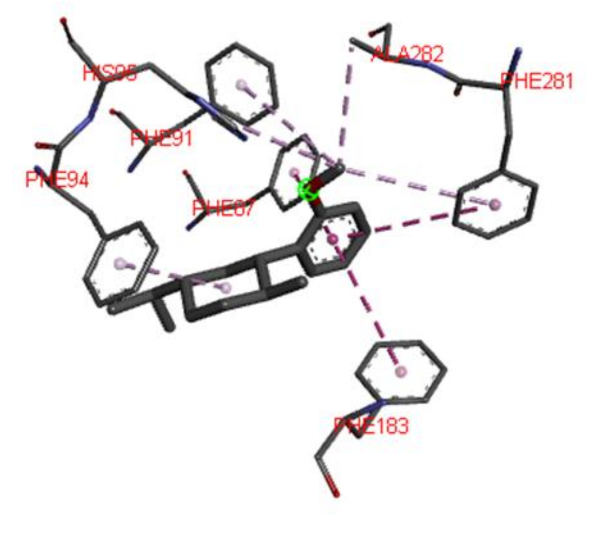
</td></tr>
<tr><td>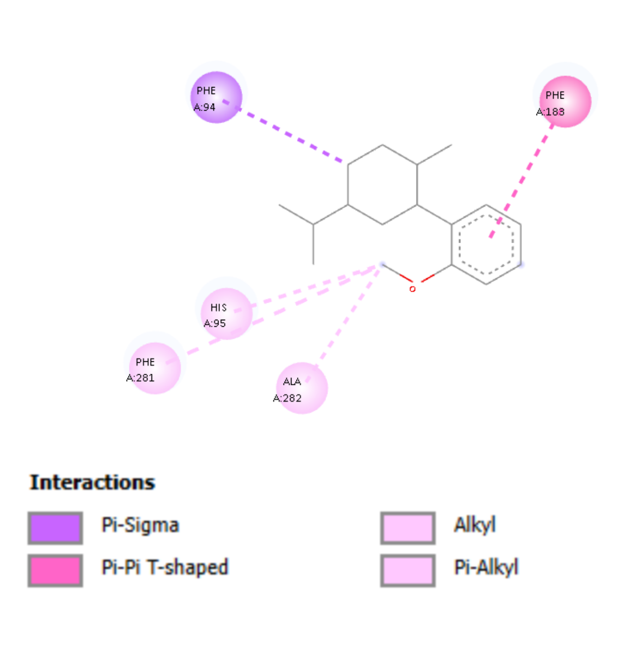
</td><td>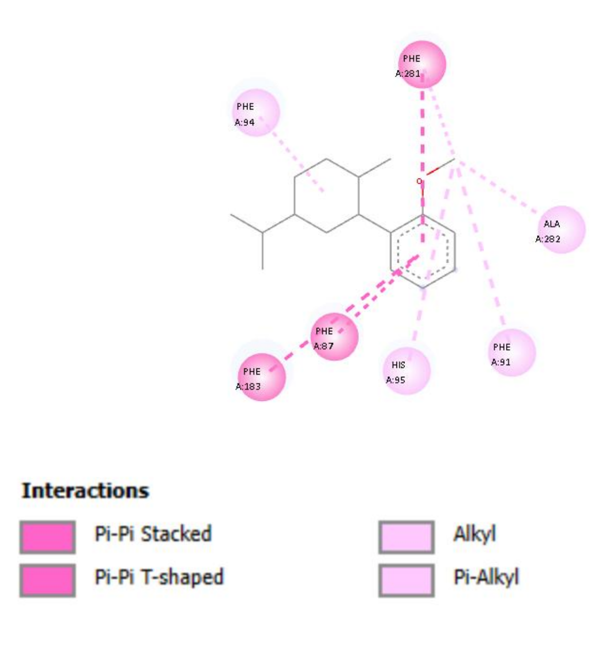
</td></tr>
<tr><td>Binding energy: -8.6</td><td>Binding energy: -8.6</td></tr>
</table>

<table>
<tr><th>9b (II)</th><th>ent-9b (II)</th></tr>
<tr><td>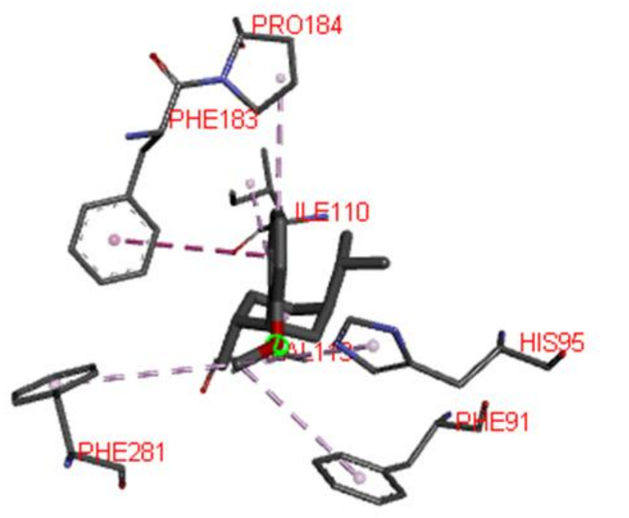
</td><td>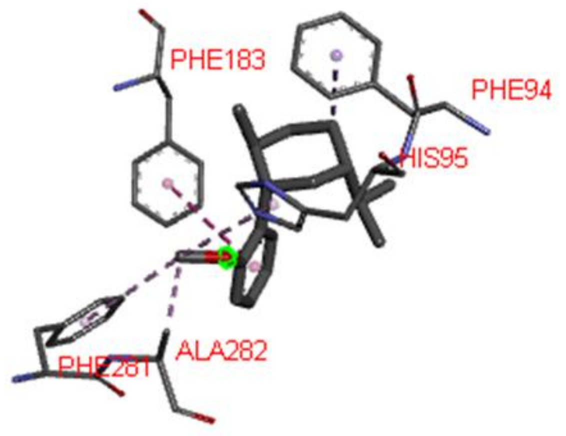
</td></tr>
<tr><td>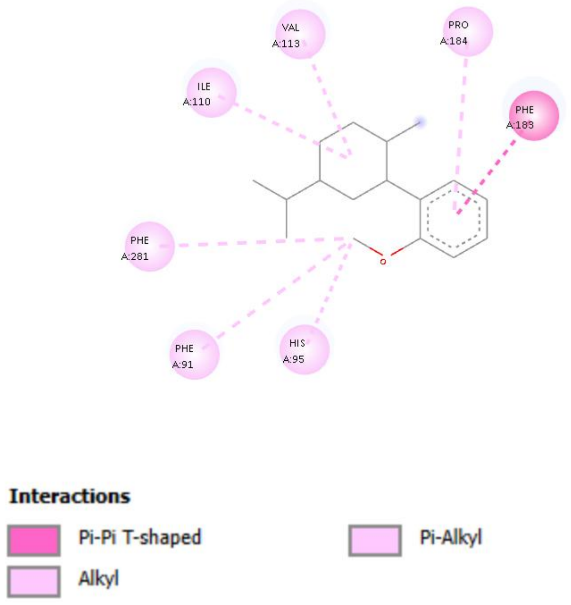
</td><td>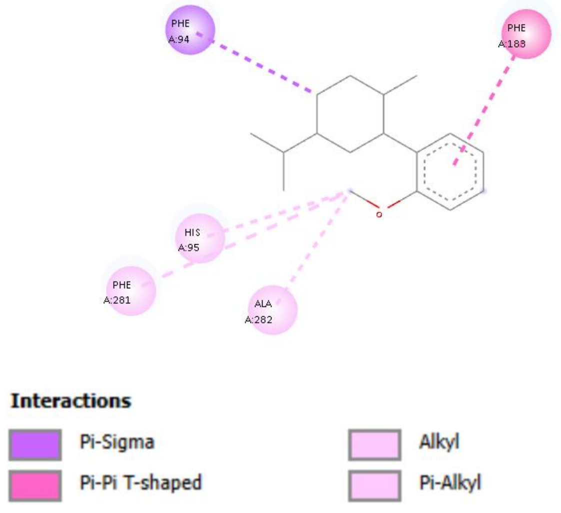
</td></tr>
<tr><td>Binding energy: -8.2</td><td>Binding energy: -8.6</td></tr>
</table>

<table>
<tr><th>9c (l)</th><th>ent-9c (l)</th></tr>
<tr><td>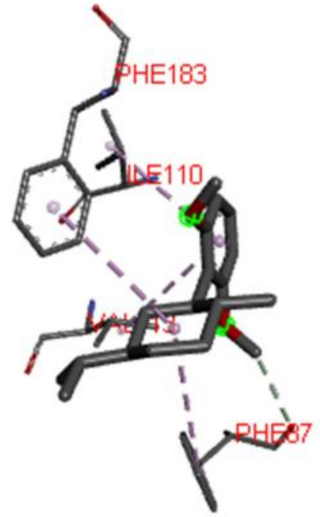
</td><td>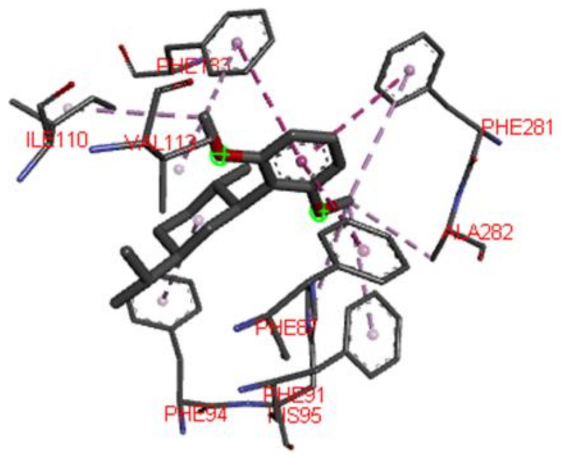
</td></tr>
<tr><td>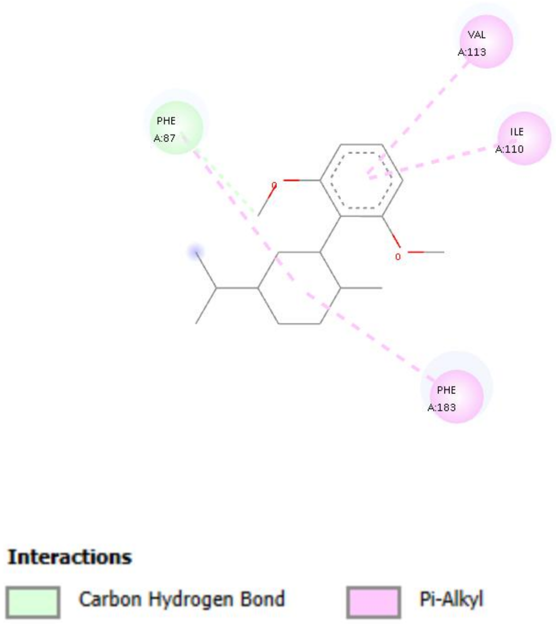
</td><td>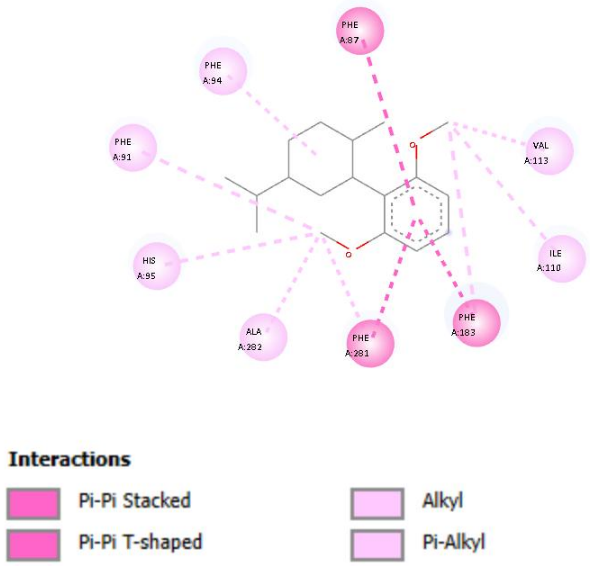
</td></tr>
<tr><td>Binding energy: -7.9</td><td>Binding energy: -8.3</td></tr>
</table>

| 9c (II) | ent-9c (II) |
|---|---|
| 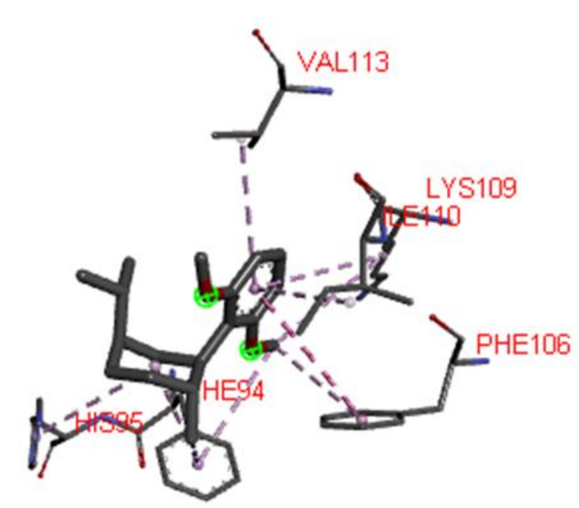 | 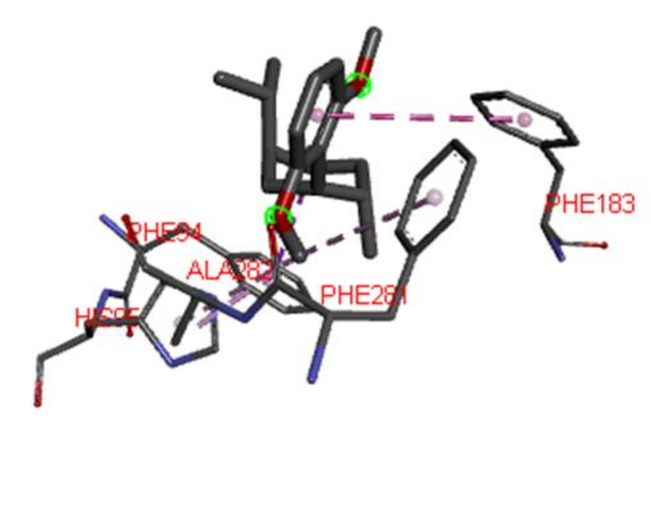 |
| 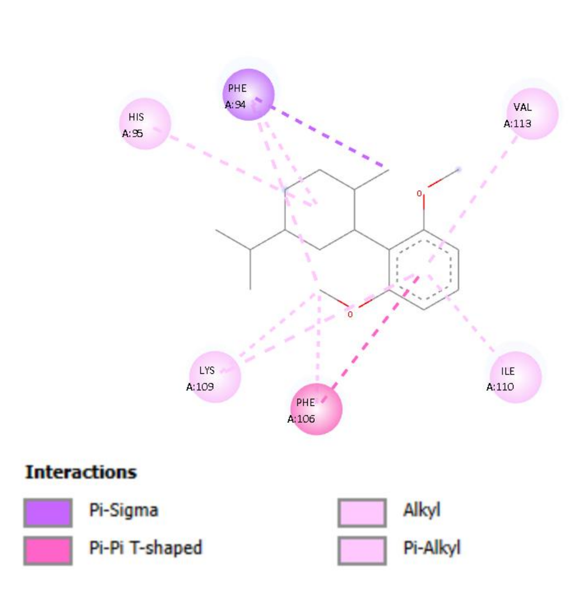 | 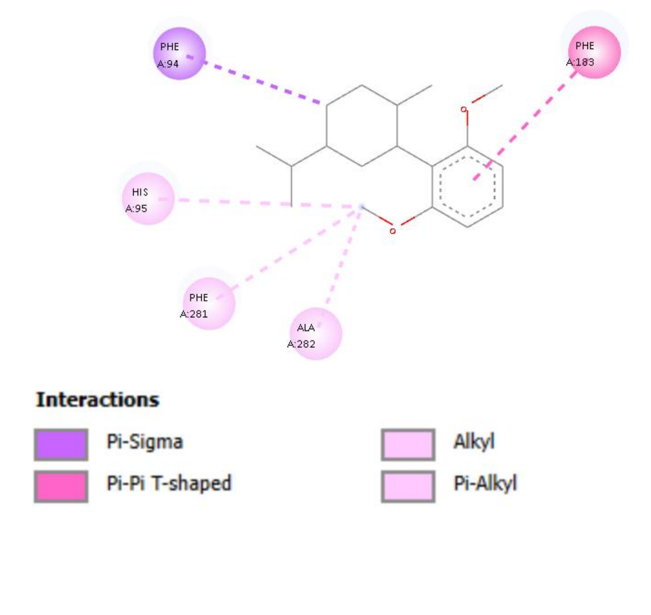 |
| **Binding energy: -8.0** | **Binding energy: -8.8** |

<table>
<tr><th>9d (I)</th><th>ent-9d (I)</th></tr>
<tr><td>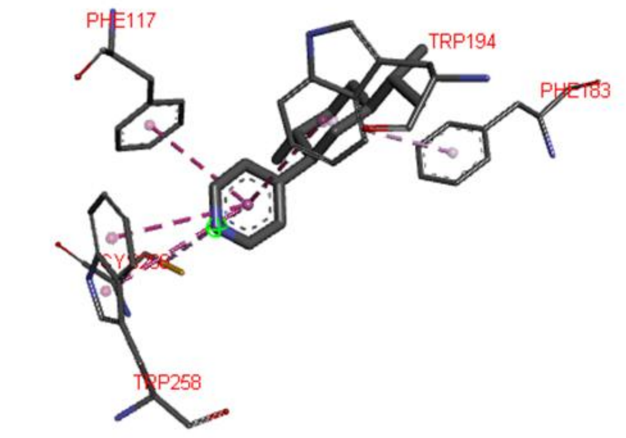
</td><td>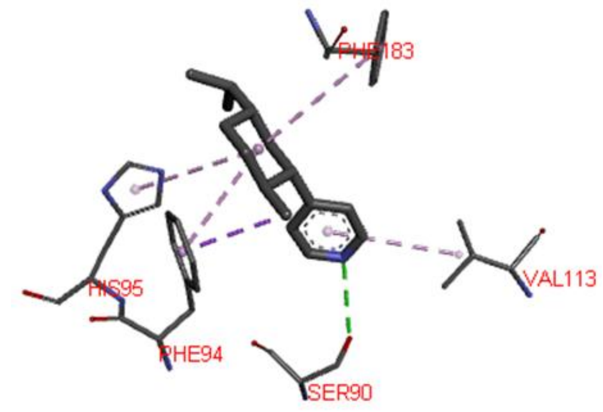
</td></tr>
<tr><td>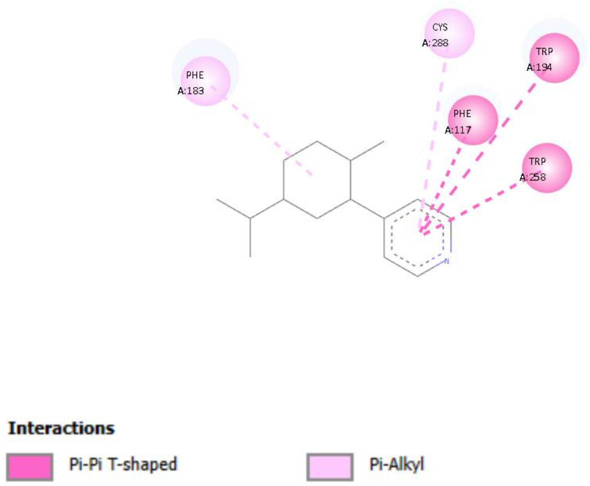
</td><td>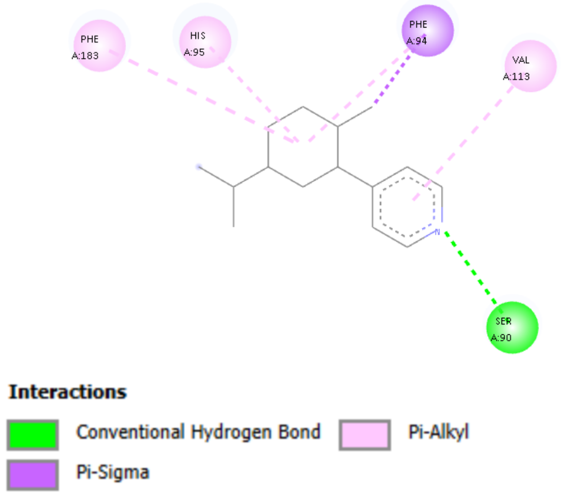
</td></tr>
<tr><td>Binding energy: -8.0</td><td>Binding energy: -8.1</td></tr>
</table>

<table>
<tr><th>9d (II)</th><th>ent-9d (II)</th></tr>
<tr><td>
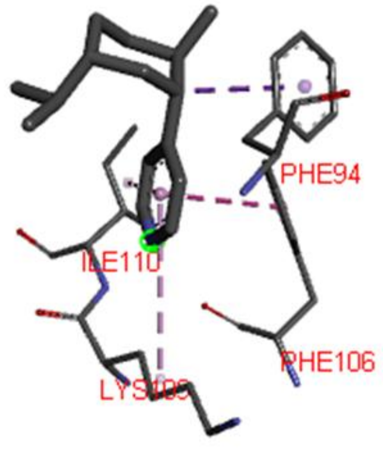

</td><td>
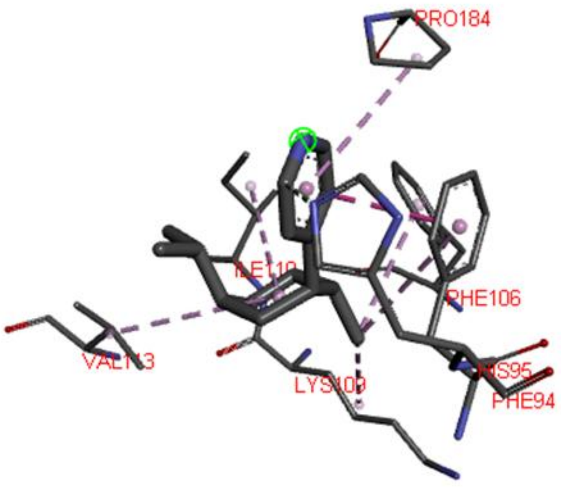

</td></tr>
<tr><td>
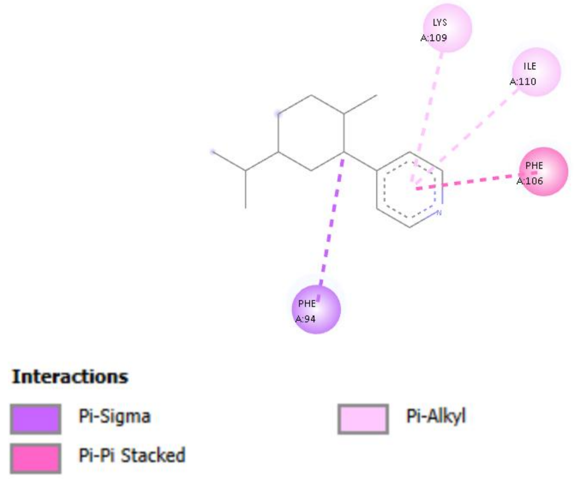

</td><td>
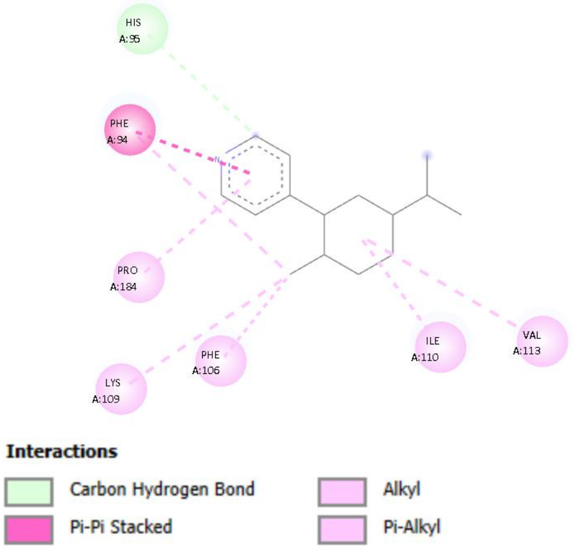

</td></tr>
<tr><td>Binding energy: -8.5</td><td>Binding energy: -9.0</td></tr>
</table>

<table>
<tr><th>9e (I)</th><th>ent-9e (I)</th></tr>
<tr><td>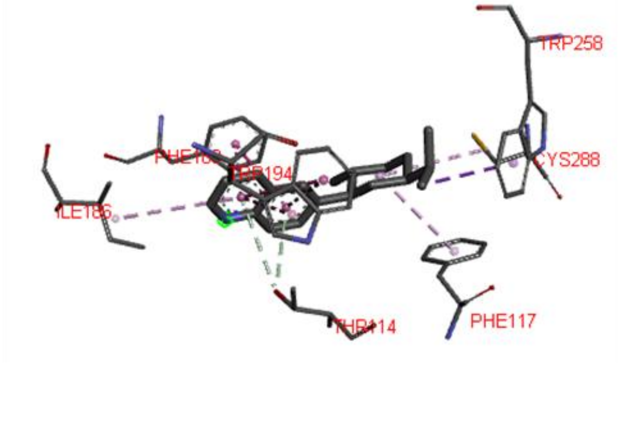
</td><td>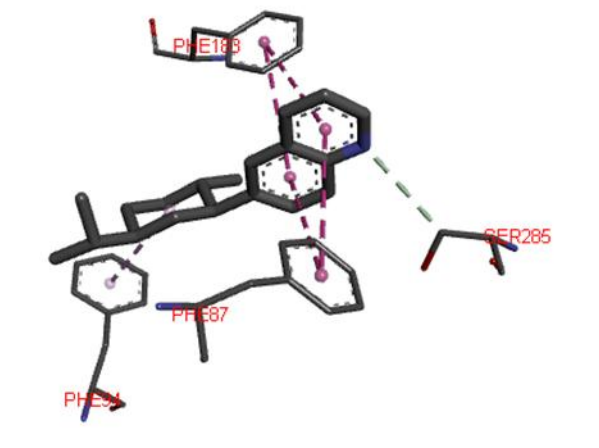
</td></tr>
<tr><td>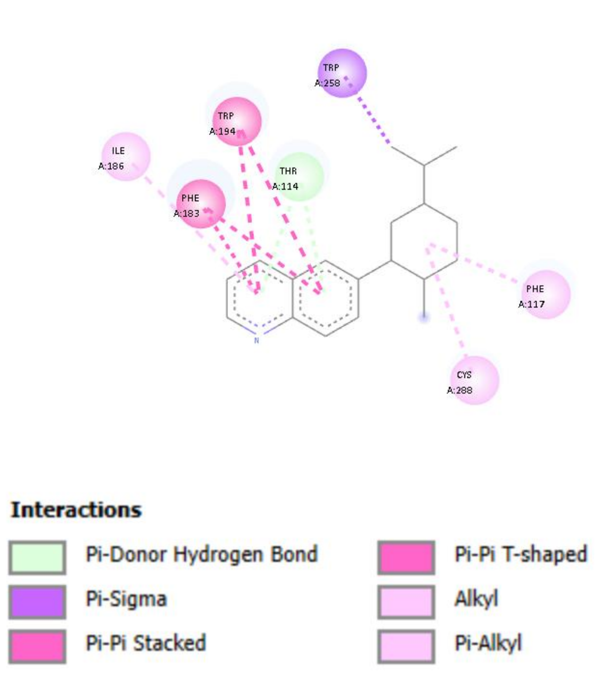
</td><td>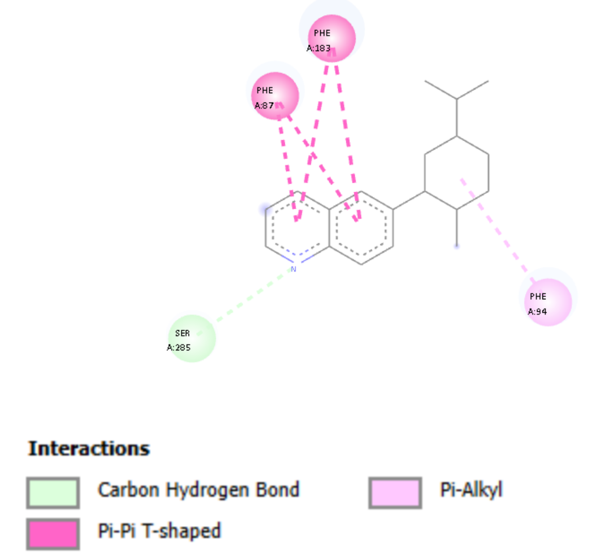
</td></tr>
<tr><td>Binding energy: -10.1</td><td>Binding energy: -10.0</td></tr>
</table>

<table>
<tr><th>9e (II)</th><th>ent-9e (II)</th></tr>
<tr><td>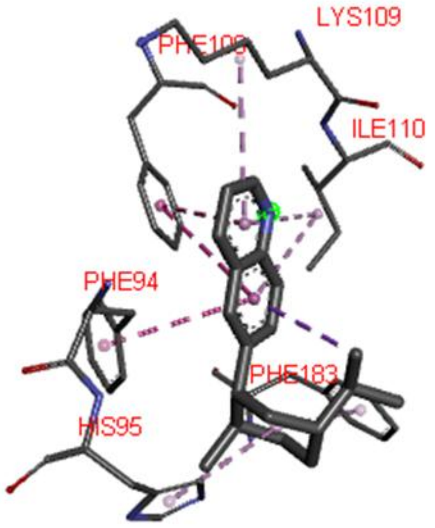<br></td><td>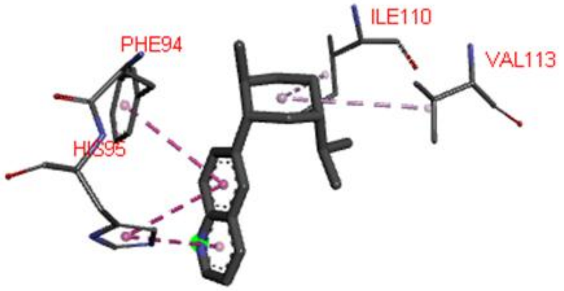<br></td></tr>
<tr><td>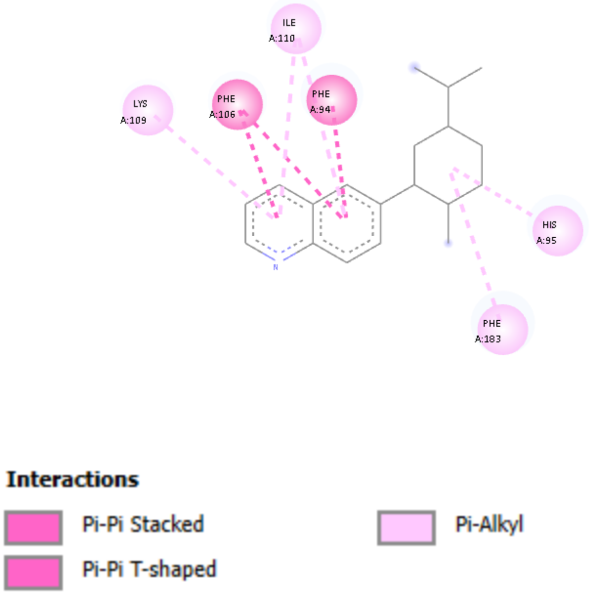<br></td><td>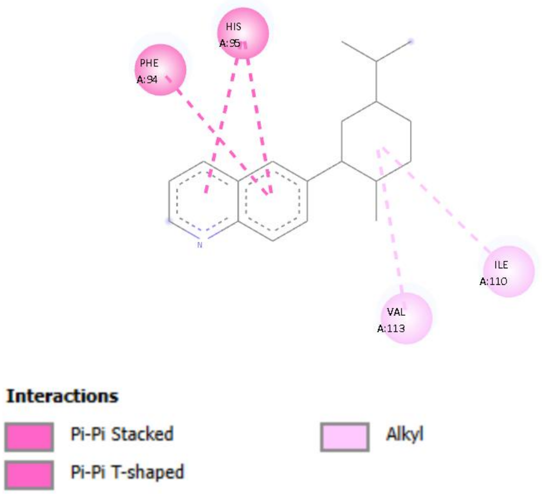<br></td></tr>
<tr><td>Binding energy: -9.4</td><td>Binding energy: -10.3</td></tr>
</table>

| 9f (I) | ent-9f (I) |
|---|---|
| 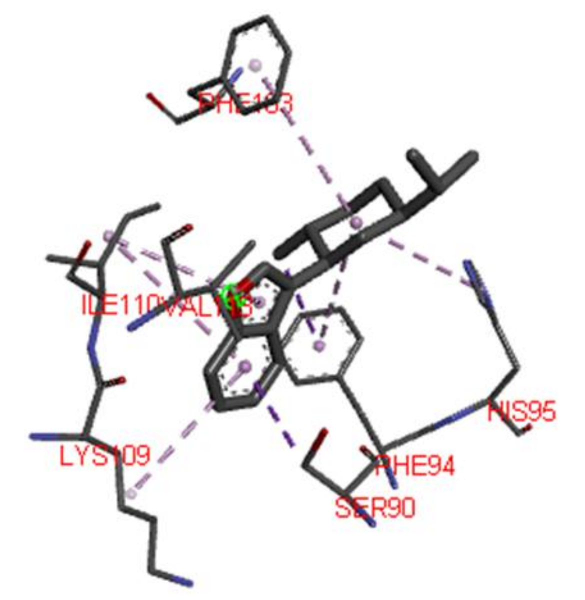 | 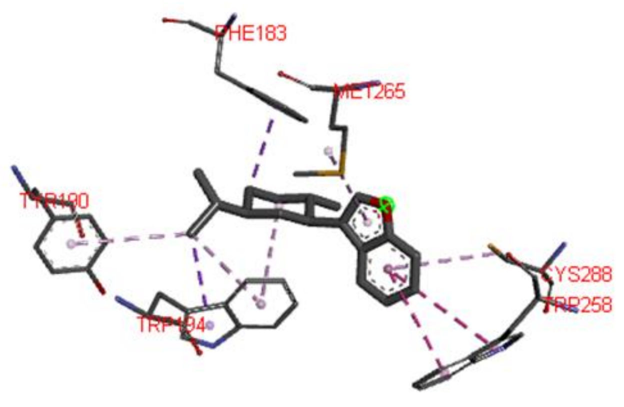 |
| 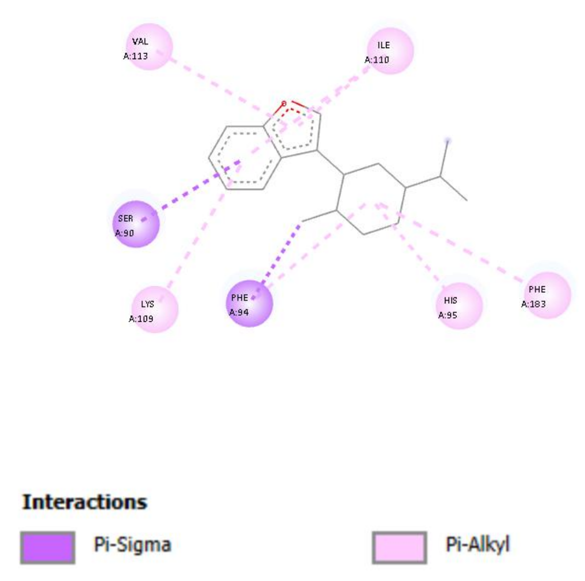 | 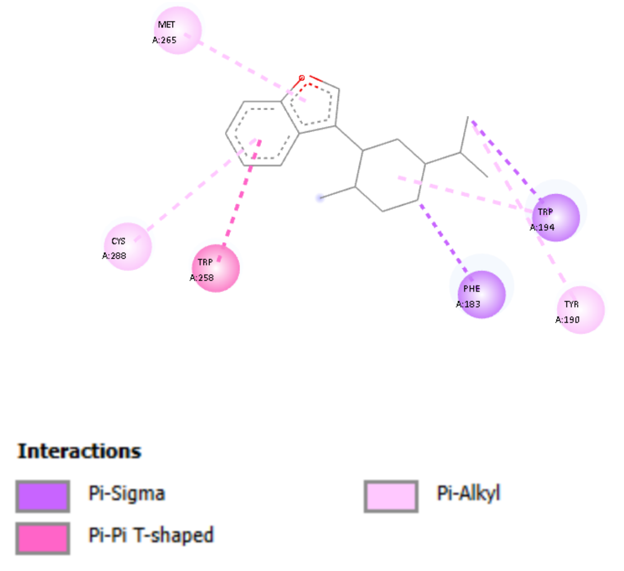 |
| **Binding energy: -9.5** | **Binding energy: -9.9** |

| 9f (II) | ent-9f (II) |
| --- | --- |
| 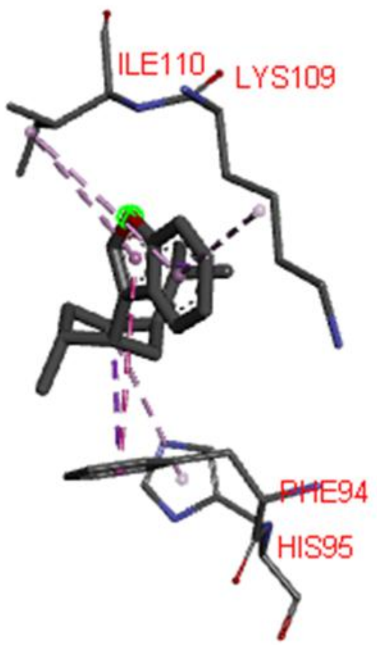 | 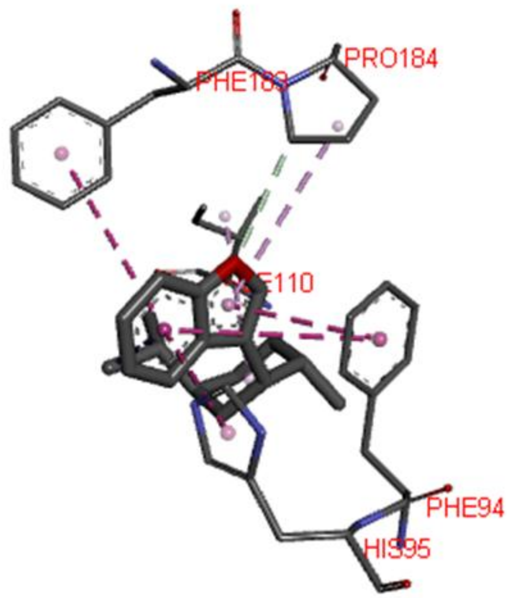 |
| 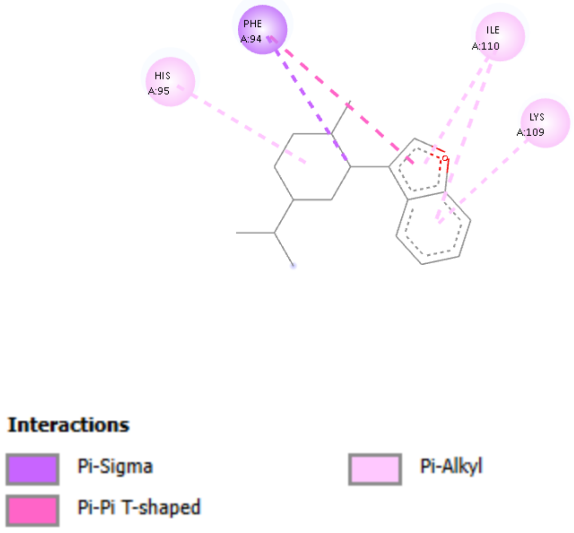 | 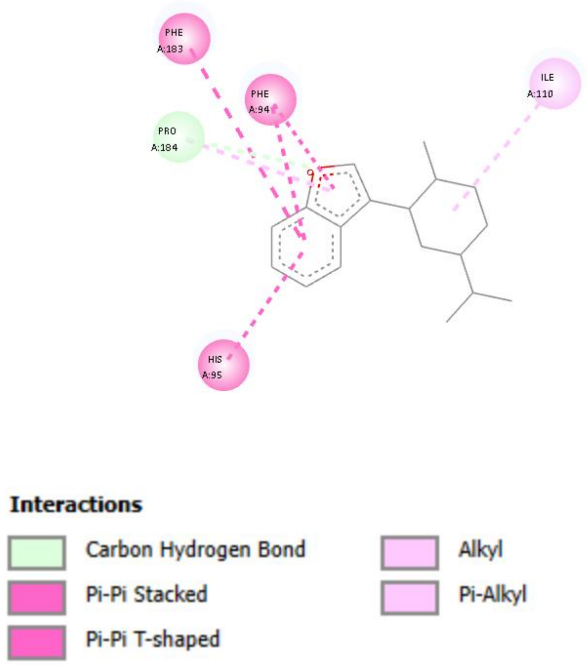 |
| **Binding energy: -9.1** | **Binding energy: -9.7** |

## VII. Molecular Docking of Known Modulators of Cannabinoid Receptors CB1 and CB2

• *CB1 Receptor Ligands*

| **Partial agonist: Tetrahydrocannabinol (THC)** |
|---|
| 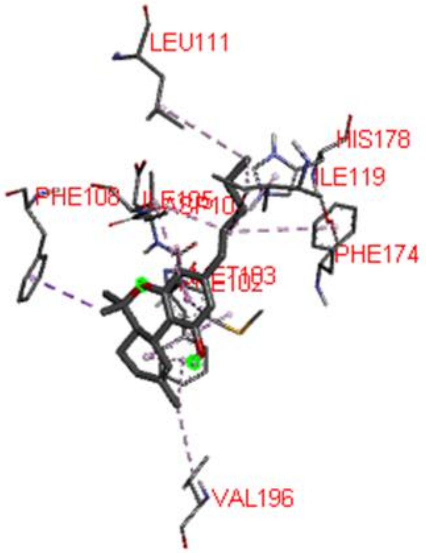  |
| 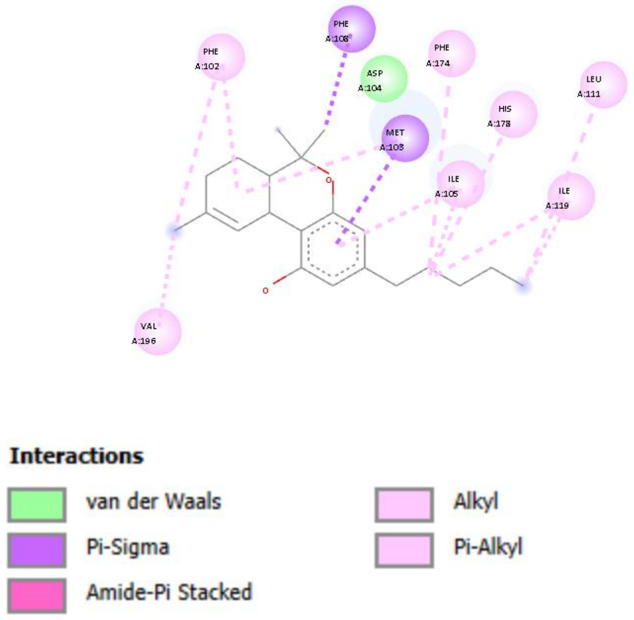  |
| **Binding energy: -9.6** |

| **Agonist: HU-210** |
| --- |
| 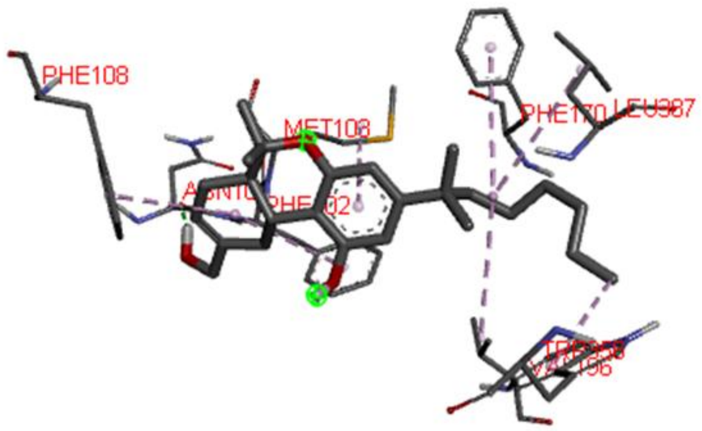 |
| 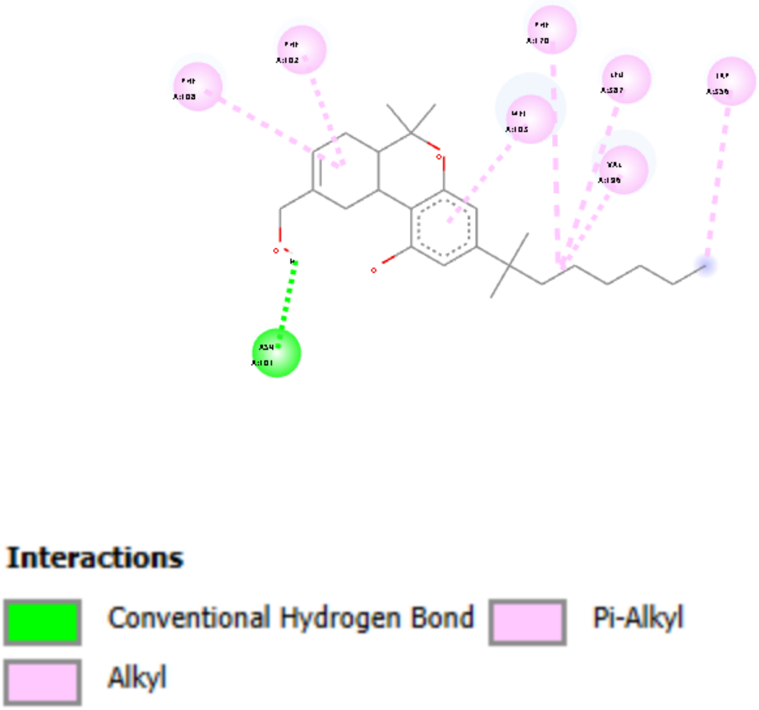 |
| **Binding energy: -9.7** |

**Antagonist: SR141716A (Rimonabant)**

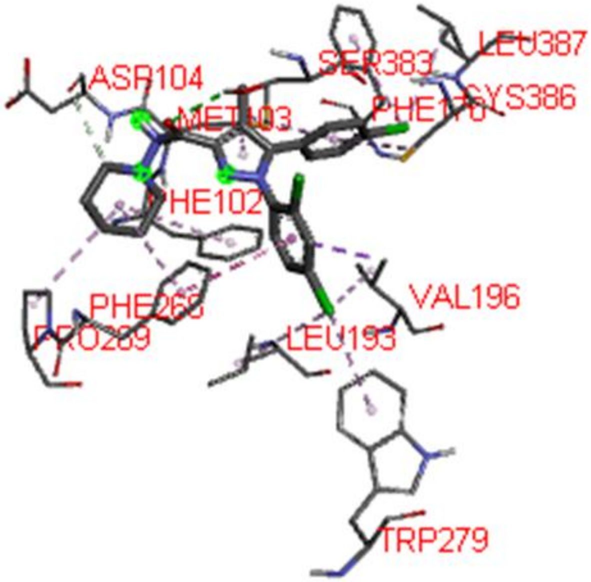


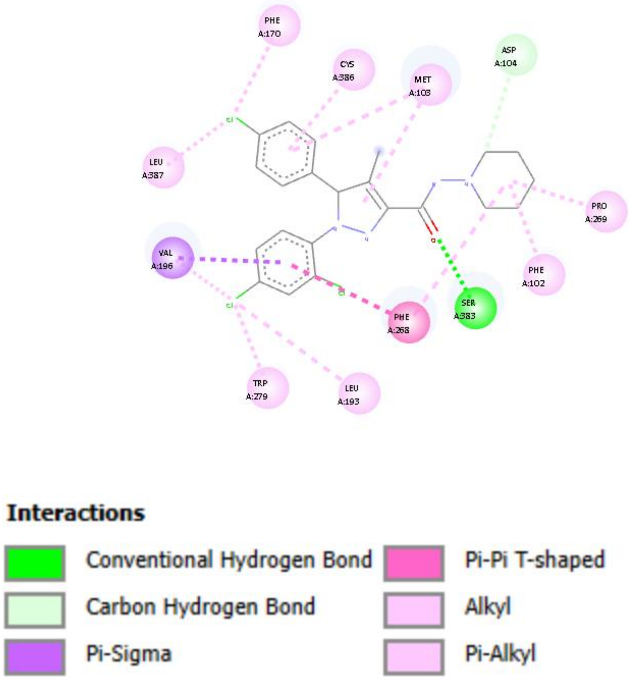


**Binding energy: -11.1**

• *CB2 Receptor Ligands*

**Partial agonist: Tetrahydrocannabinol (THC)**

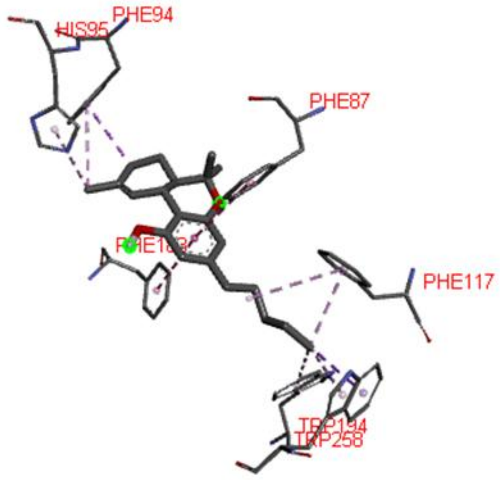


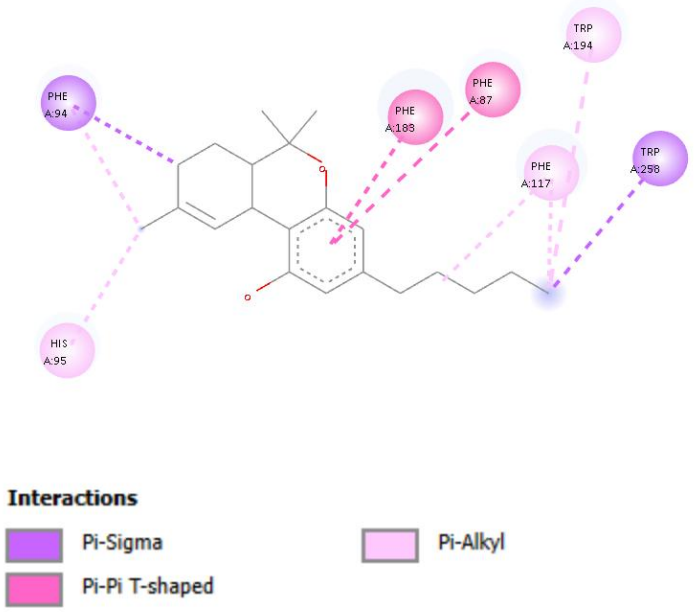


**Binding energy: -10.2**

**Agonist: HU-210**

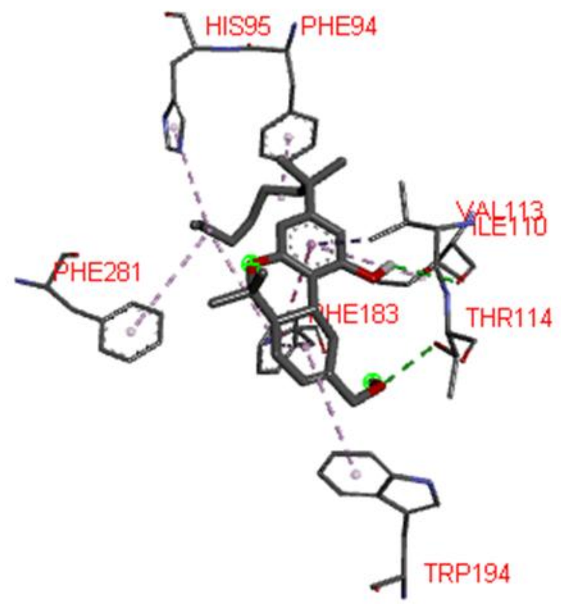


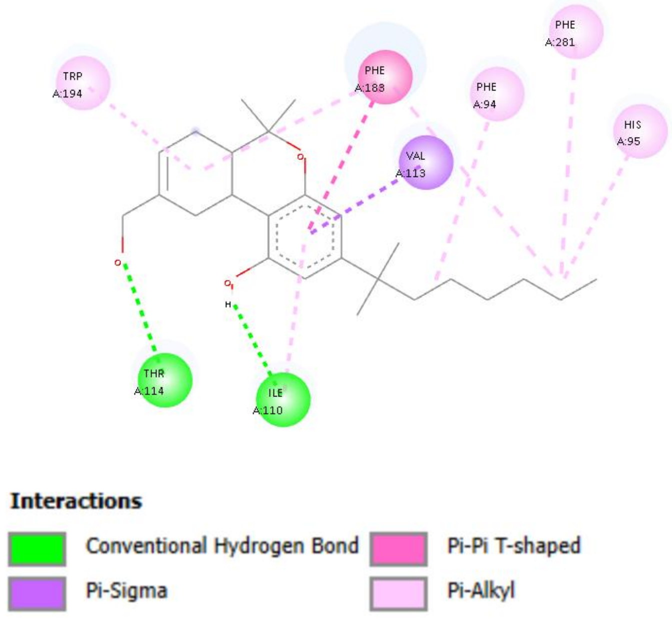


**Binding energy: -10.7**

**Antagonist: HY13439**

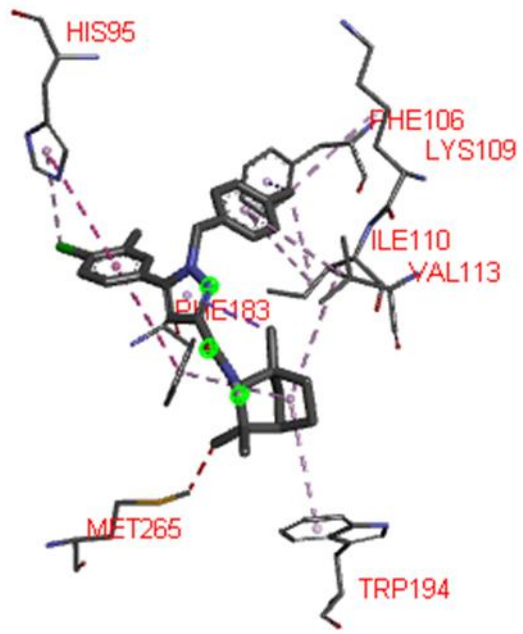


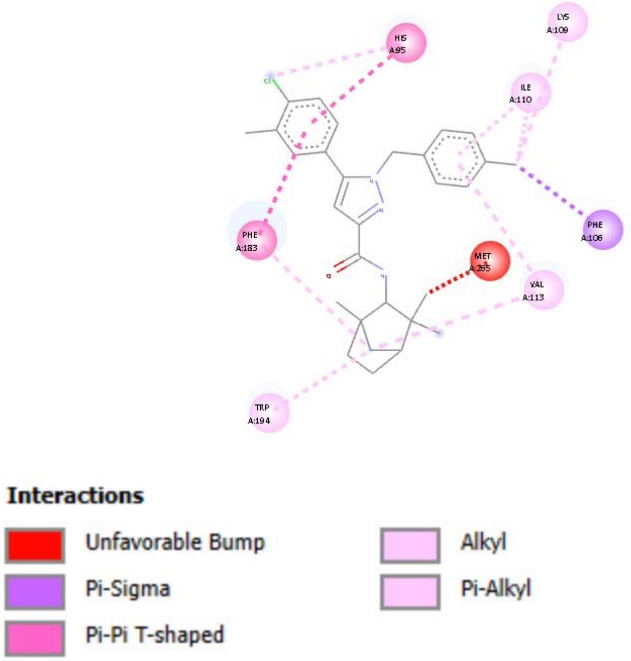


**Binding energy: -11.2**
